# Metal-free photosensitized radical relay 1,4-carboimination across two distinct olefins†

**DOI:** 10.1039/d2sc06497a

**Published:** 2023-02-03

**Authors:** Guangying Tan, Fritz Paulus, Alessia Petti, Maxim-Aleksa Wiethoff, Anna Lauer, Constantin Daniliuc, Frank Glorius

**Affiliations:** a Organisch-Chemisches Institut, Westfälische Wilhelms-Universität Münster Corrensstraße 36 48149 Münster Germany glorius@uni-muenster.de

## Abstract

Intermolecular carboamination of olefins offers a powerful platform for the rapid construction of structurally complex amines from abundant feedstocks. However, these reactions often require transition-metal catalysis, and are mainly limited to 1,2-carboamination. Herein, we report a novel radical relay 1,4-carboimination across two distinct olefins with alkyl carboxylic acid-derived bifunctional oxime esters *via* energy transfer catalysis. The reaction is highly chemo- and regioselective, and multiple C–C and C–N bonds were formed in a single orchestrated operation. This mild and metal-free method features a remarkably broad substrate scope with excellent tolerance of sensitive functional groups, therefore providing easy access to structurally diverse 1,4-carboiminated products. Moreover, the obtained imines could be easily converted into valuable biologically relevant free γ-amino acids.

## Abstract 2

Intermolecular carboamination of olefins offers a powerful platform for the rapid construction of structurally complex amines from abundant feedstocks.

## Graphical abstract

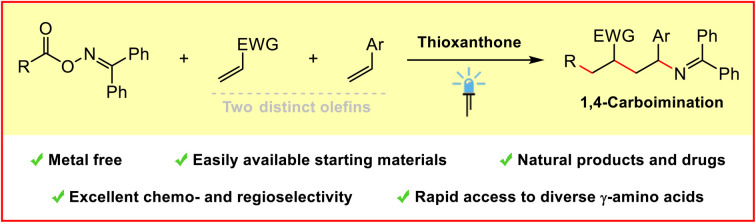

## Introduction

**Scheme 1:**
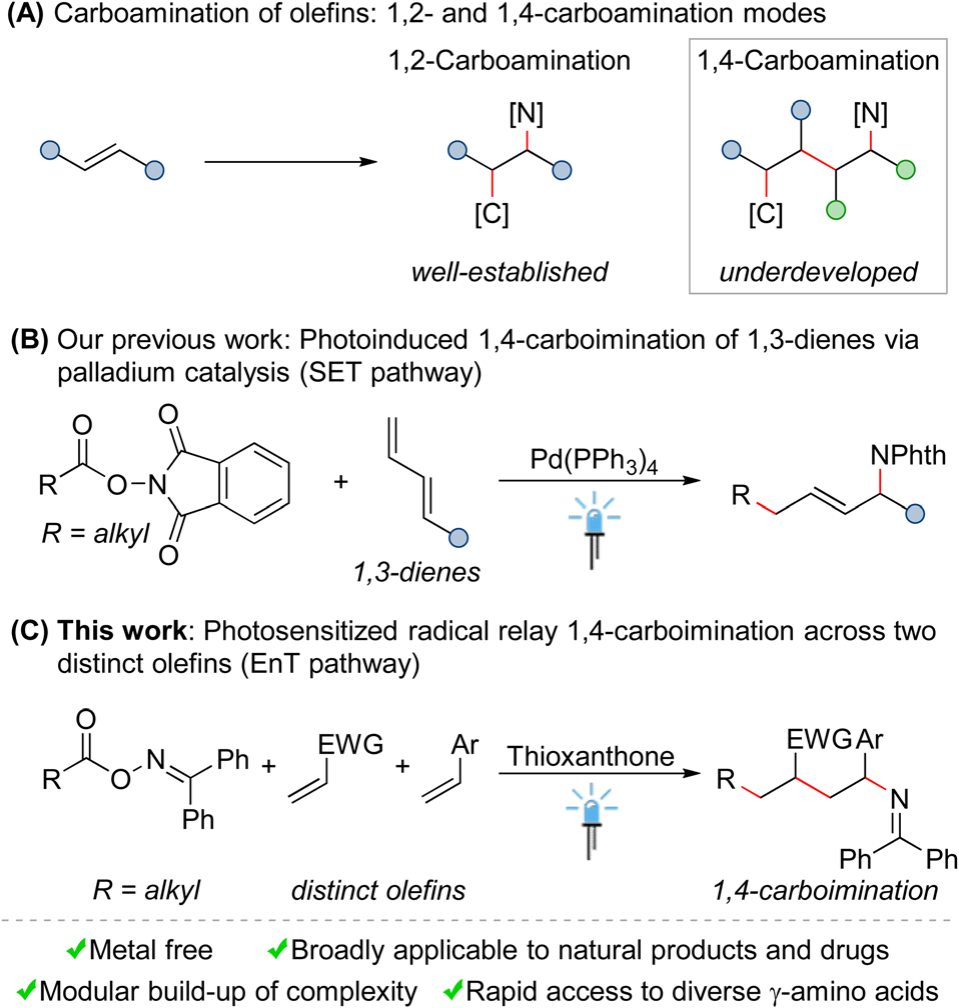
The development of 1,4-carboamination of olefins. (A) Carboamination of olefins: 1,2- and 1,4-carboamination modes. (B) Our previous work: photoinduced 1,4-carboimination of 1,3-dienes *via* palladium catalysis (SET pathway). (C) This work: photosensitized radical relay 1,4-carboimination across two distinct olefins (EnT pathway). SET: single electron transfer. EnT: energy transfer. NPhth: phthalimido.

Nitrogen-containing organic molecules are widely found in natural products, agrochemicals, pharmaceutical agents, and functional materials.^1^ Consequently, the selective incorporation of amine functionalities into small molecules has received considerable attention in recent years.^2^ In this context, the carboamination of olefins represents a vibrant research area in organic synthesis, as such transformations allow for the simultaneous installation of C–C and C–N bonds across simple olefins in a single operation, thereby constructing complex molecules in a modular manner from abundant feedstocks.^3,4^ However, despite significant achievements made over the past decades, some fundamental issues still remain to be solved in this field. First, these reactions often require transition-metal catalysis, rendering them costly and environmentally harmful. Second, the current studies are mainly limited to 1,2-carboamination of olefins, namely introducing carbon and amine functionalities across one olefin at its vicinal positions (Scheme 1A). Undoubtedly, from a synthetic perspective, the development of controllable remote 1,*n*-carboamination across more than one olefin to modularly access more structurally diverse amines, ideally under mild and metal-free conditions, is highly desirable.

Compared to the vigorous growth and abundant achievements in 1,2-carboamination chemistry of olefins, remote carboamination reactions are so far rarely developed. Comparatively few examples are transition-metal (Pd, Rh or Cu)-catalyzed 1,4-carboaminations of 1,3-dienes.^5^ Moreover, with the development of photochemistry,^6^ modern visible light-mediated methods for 1,4-carboaminations have recently gradually emerged.^7–9^ In a notable example, Sarlah and coworkers demonstrated an elegant strategy for the stepwise *syn*-1,4-carboamination of simple arenes by the combination of photoinduced cycloaddition and palladium-catalyzed ring-opening substitution.^7^ Very recently, we have realized a palladium-catalyzed 1,4-carboimination of 1,3-dienes with alkylcarboxylic acid-derived bifunctional reagents through a single electron transfer (SET) approach mediated by visible light (Scheme 1B).^8^ Based on this strategy, the palladium-catalyzed three component 1,4-carboamination of 1,3-dienes with haloalkanes and nitrogen nucleophiles has been subsequently achieved by other groups and us.^9^ Although these reactions are versatile, they require the use of transition metals (especially palladium), as catalysts. Moreover, the utilization of unstable 1,3-dienes as starting materials further restricts the general appeal of these protocols.

Seeking to address these limitations, we herein disclose an unprecedented radical relay 1,4-carboimination across two distinct olefins with alkyl carboxylic acid-derived bifunctional oxime esters *via* energy transfer (EnT) catalysis^10^ (Scheme 1C). This mild and metal-free reaction is highly chemo- and regioselective, and multiple C–C and C–N bonds were formed in a single operation in an orchestrated manner. Moreover, due to the direct utilization of two distinct olefins as starting materials, this method provides modular access to structurally diverse 1,4-carboiminated products, which could be easily converted into valuable biologically relevant free γ-amino acids.^11^

## Results and discussion

### Reaction development

Bifunctional reagents have gained a preeminent role within organic synthesis, thanks to their versatility, atom economy, and efficient activation modes.^12^ Recently, we and others have identified a class of benzophenone-based oxime esters as a suitable bifunctional source for supplying both carbon- and nitrogen-centered radicals through energy transfer catalysis.^13–15^ Our investigation commenced with the reaction of benzophenone *O*-pivaloyl oxime (1a), ethene-1,1-diyldibenzene (2a), and acrylonitrile (3a) under visible-light-sensitized conditions (Table 1).

**Table 1:** Optimization of the reaction conditions^a^^,^^b^

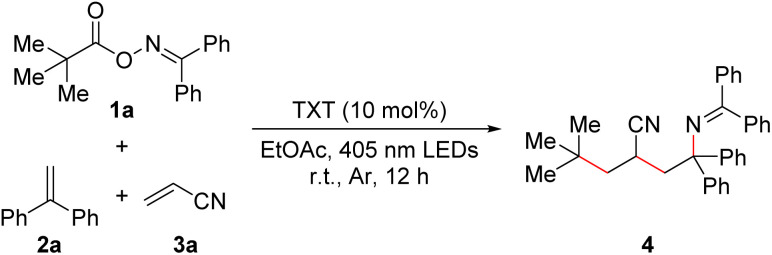			
Entry	Variation	Yield^b^ [%]	
1	None	63, 60^c^	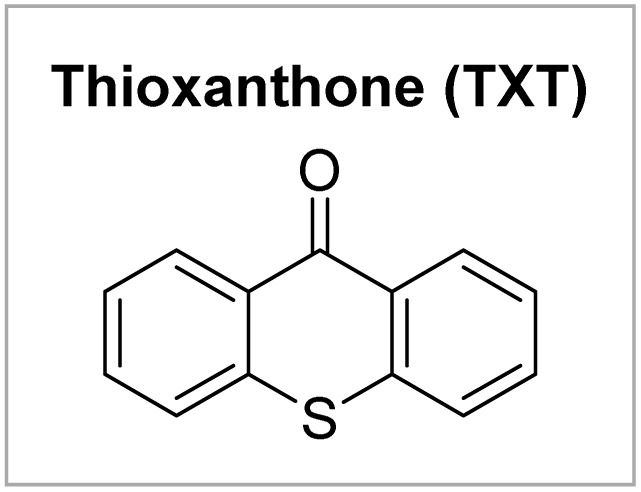
2	w/o TXT	N.R.	
3	w/o light	N.R.	
4	450 nm LEDs	N.R.	
5	CH_2_Cl_2_ as solvent	56	
6	Acetone as solvent	54	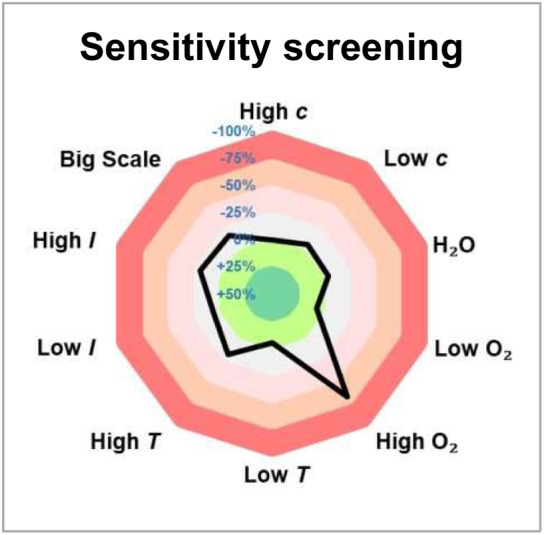
7	MeCN as solvent	57	
8	DCE as solvent	48	
9	[Ir–F]^d^ (1.0 mol%) instead of TXT and 450 nm LEDs	50	
10	EtOAc (0.05 M)	53	
11	1.5 equiv. of 3a	58	
12	1.0 equiv. of 1a, 2.0 equiv. of 2a and 3a	53	

a Standard conditions: 1a (0.3 mmol, 1.5 equiv.), 2a (0.2 mmol, 1.0 equiv.), 3a (0.4 mmol, 2.0 equiv.), and thioxanthone (10 mol%) in EtOAc (0.1 M, 2.0 mL), irradiation with 18 W blue LEDs (*λ*_max_ = 405 nm) under argon atmosphere at room temperature for 12 h.

b NMR yields are given.

c Isolated yield.

d [Ir–F] = [Ir(dF(CF_3_)ppy)_2_(dtbbpy)](PF_6_).

To our delight, after a systematic evaluation of the reaction parameters, the best yield for the desired product 4 reached 63% under the optimized reaction conditions: 1a (0.3 mmol, 1.5 equiv.), 2a (0.2 mmol, 1.0 equiv.), 3a (0.4 mmol, 2.0 equiv.), and thioxanthone (10 mol%) in EtOAc (0.1 M, 2.0 mL), and irradiation with 18 W blue LEDs (*λ*_max_ = 405 nm) under argon atmosphere at room temperature for 12 h (Table 1, entry 1). Control experiments demonstrated that both thioxanthone and blue light (*λ*_max_ = 405 nm) were indispensable for this reaction (Table 1, entries 2 and 3). As expected, the reaction did not proceed when switching to a blue light with a wavelength maximum of 450 nm (Table 1, entry 4). This protocol appeared to be relatively robust regarding the choice of solvents, as replacing EtOAc with CH_2_Cl_2_, acetone, MeCN, or DCE still provided 4 in similar yields (Table 1, entries 5–8). In addition, [Ir(dF(CF_3_)ppy)_2_(dtbbpy)](PF_6_) proved to be a suitable catalyst for the reaction as well, giving product 4 in 50% yield (Table 1, entry 9). Other variations from the standard conditions, such as decreasing the concentration of the reaction or changing the substrate ratio, led to slightly diminished yields of product 4 (Table 1, entries 10–12). Furthermore, a reaction condition-based sensitivity screening was conducted, showing that the reaction was relatively robust towards moisture, light intensity, scale-up, and small changes in concentration and temperature, but sensitive towards high oxygen level (Table 1, see ESI† for the details).^16^

### Mechanistic analysis

**Fig. 1:**
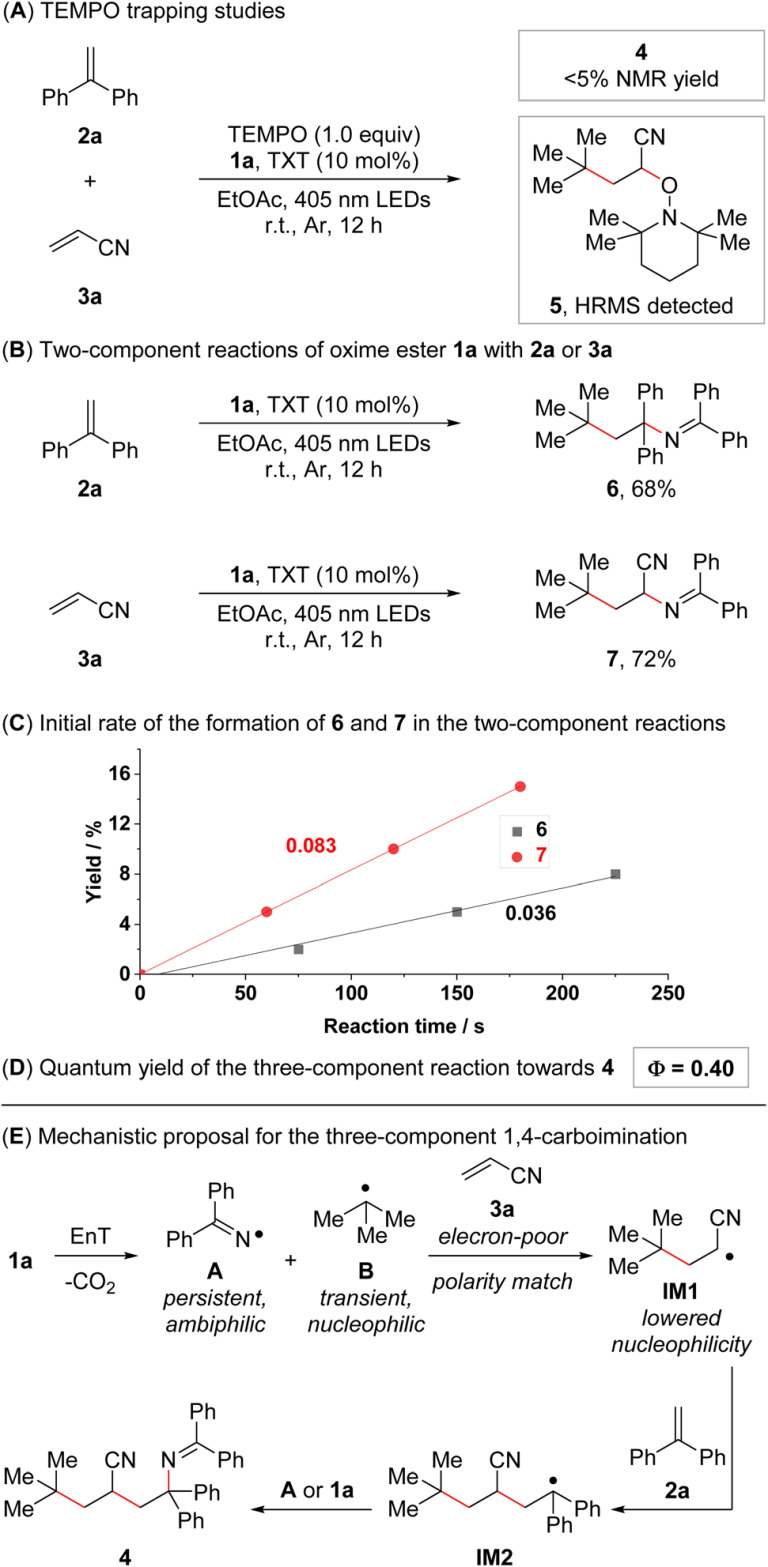
Mechanistic studies and mechanistic proposal. (A) TEMPO trapping experiment. Reaction conditions: 1a (0.15 mmol, 1.5 equiv.), 2a (0.1 mmol, 1.0 equiv.), 3a (0.2 mmol, 2.0 equiv.), thioxanthone (TXT, 10 mol%), and TEMPO (0.1 mmol, 1.0 equiv.) in EtOAc (0.1 M, 1.0 mL), 18 W blue LEDs (*λ*_max_ = 405 nm), r.t., 12 h. (B) Two-component reactions of 1a with 2a or 3a. Reaction conditions: 1a (0.3 mmol, 1.5 equiv.), 2a or 3a (0.2 mmol, 1.0 equiv.), and thioxanthone (10 mol%) in EtOAc (0.1 M, 2.0 mL), 18 W blue LEDs (*λ*_max_ = 405 nm), r.t., 12 h. Isolated yields given. (C) Initial course and rate of the reaction between 1a and 2a and the reaction between 1a and 3a (see ESI† for the details). (D) Quantum yield of the reaction between 1a, 2a, and 3a (see ESI† for the details). (E) Mechanistic proposal for the intermolecular 1,4-carboimination of 2a and 3a with 1a.

In order to obtain insights into the underlying reaction mechanism, we conducted a series of mechanistic experiments. Only trace amounts of 4 were observed when conducting the standard reaction in the presence of TEMPO ((2,2,6,6-tetramethylpiperidin-1-yl)oxyl), and compound 5 was detected in the reaction mixture by high-resolution mass spectrometry (Fig. 1A). Furthermore, the reaction was carried out under direct excitation conditions (irradiation at 365 nm instead of 405 nm; no photocatalyst), delivering product 4 in 38% NMR yield (see ESI† for the details). These results are consistent with an energy transfer-based radical pathway and an initial radical attack of the generated carbon-centered radical B to 3a. Subsequently, we turned our attention towards the corresponding two-component 1,2-carboiminations of alkenes 2a or 3a with oxime ester 1a (Fig. 1B). Both reactions delivered the corresponding 1,2-carboiminated products (6 and 7) in good yields, even though these compounds were only observed as minor side products in the three-component reaction of 1a, 2a, and 3a (see ESI† for the details). To further probe the origin of the regioselectivity observed for the assembly of 4, we conducted preliminary kinetic studies on both two-component reactions. The initial formation of 7 was found to be more than twice as fast than the formation of 6 (Fig. 1C), which is a hint that the formation of 4 might initiate with fast radical attack to 3a. Lastly, the quantum yield of the reaction of 1a, 2a, and 3a was determined. A value of 0.40 was obtained, rendering a pure radical chain mechanism unlikely (Fig. 1D).^15*c*^ However, a mixed radical chain/radical recombination mechanism cannot be ruled out.

Based on these experiments and our previous studies,^13,14^ a mechanistic analysis of the selective 1,4-carboimination of 1a, 2a, and 3a was developed (Fig. 1E, see ESI† for details). The reaction initiates with an energy transfer-enabled homolysis of 1a to provide the persistent N-centered iminyl radical A and the transient C-centered alkyl radical B, which are of ambiphilic and nucleophilic properties, respectively. Then, steered by polarity matching, persistent radical effects (PRE),^17^ and a higher concentration of 3a compared to 2a, the nucleophilic C-centered alkyl radical B preferentially adds to electron poor olefin 3a to form an α-cyano-containing C-centered alkyl radical with reduced nucleophilicity (IM1). Subsequently, addition of IM1 to 2a gives another benzyl stabilized C-centered alkyl radical (IM2), which is further trapped by the persistent N-centered iminyl radical A to furnish the desired 1,4-carboimination product 4*via* radical recombination. Alternatively, due to low concentrations of the radical species and a relatively high concentration of oxime ester 1a, IM2 can form product 4*via* radical attack to oxime ester 1a and subsequent fragmentation, representing a radical chain mechanism.^15*c*,18^

This mechanism is therefore related to our previous work, in which we used oxime carbonates as a source for electrophilic oxygen-centered radicals to achieve a three-component 1,4-oxyimination.^18^ In both cases, the desired regioselectivity was achieved by rational reaction design based on the philicity of the generated radicals and the polarity of the olefins. Notably, the different philicities of carbon- and oxygen-centered radicals cause a different order of building block assembly depending on the selected reagent.

### Synthetic scope

With the optimized reaction conditions in hand, we conducted an additive-based robustness screening to initially assess the reaction's functional group compatibility. The obtained results highlighted the outstanding tolerance of heterocycles and functional groups and already hinted towards the broadness of the substrate scope (see ESI† for the details).^19^ Next, the substrate scope with respect to the three reaction partners was examined (Table 2). First, a range of alkyl carboxylic acid-derived bifunctional oxime esters was explored. Tertiary alkyl carboxylic acids smoothly underwent this radical relay 1,4-carboimination reaction, providing the corresponding products in moderate to good yields (Table 2A, 4 and 8–12). Notably, these reactions were highly chemo- and regioselective, and two quaternary carbon centers were easily created, demonstrating the appeal of our method in building complex molecules. Then, a series of secondary and primary carboxylic acids was tested (Table 2B, 13–24). Various cyclic and acyclic carboxylic acids were all well compatible with this protocol, delivering the desired products in moderate to good yields. The structure of product 19 was further confirmed by X-ray crystallography (Table 2B).^20^ Subsequently, we turned our attention to the compatibility of olefins. As shown in Table 2C, diverse styrene derivatives with different electron-donating or electron-withdrawing substituents in various positions of the aromatic ring were found to be effective substrates for this 1,4-carboimination reaction, producing the corresponding products in moderate to good yields (Table 2C, 25–36). Functional groups such as bromide, acetoxy and an ester containing a tosyl group were well tolerated, offering opportunities for further functionalization. Moreover, a series of Michael acceptors such as acrylates and vinyl phosphate were also suitable for this transformation (Table 2D, 37–46). Satisfactorily, a variety of sensitive functional groups like alkenyl fluoride, trifluoromethyl, alkynyl, ester, phosphonate, and even a free alcohol were well tolerated in this method. Due to polar mismatch, electron rich and unactivated olefins were not suitable for this protocol. We further tested oxime esters with different imine moieties leading to products 47–50. Benzophenone-derived oxime esters containing fluoride, bromide, or methoxy groups were tolerated. Notably, an acetophenone-derived oxime ester delivered product 50 in moderate yield with well-controlled *E*/*Z*-selectivity.

**Table 2:** Scope of alkyl carboxylic acid-derived bifunctional oxime esters and olefins^a^^,^^b^

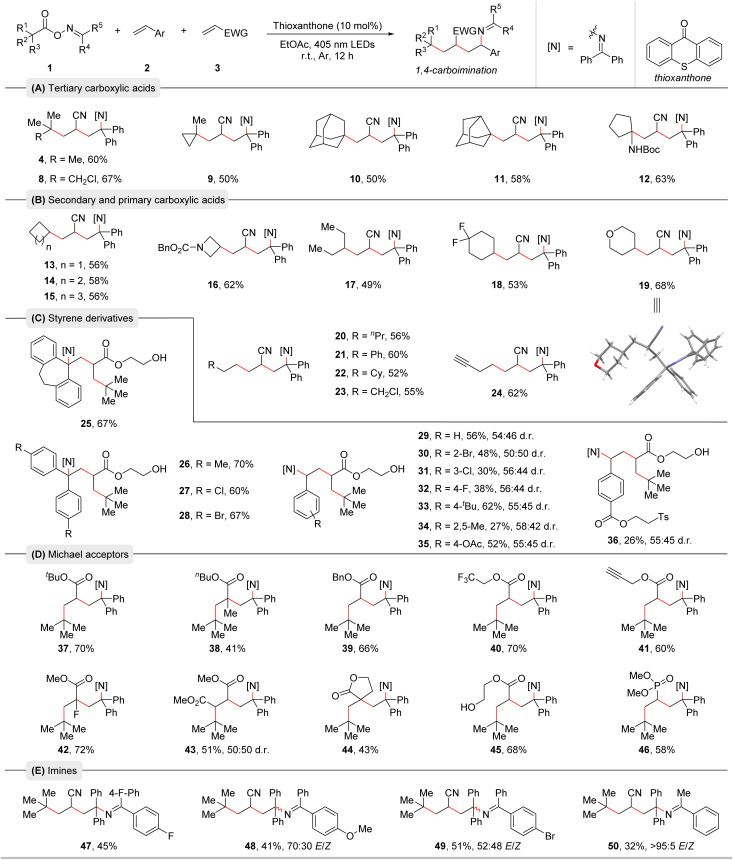

a Reaction conditions: Oxime esters 1 (0.3 mmol, 1.5 equiv.), styrene derivatives 2 (0.2 mmol, 1.0 equiv.), Michael acceptors 3 (0.4 mmol, 2.0 equiv.), and thioxanthone (10 mol%) in EtOAc (0.1 M, 2.0 mL), irradiation with 18 W blue LEDs (*λ*_max_ = 405 nm) under argon atmosphere at room temperature for 12 h.

b Isolated yields are given. The d.r. and *E*/*Z* values were determined by ^1^H NMR analysis.

Among various potential carbon-centered radical precursors, alkyl carboxylic acids represent an ideal starting material as these stable compounds are abundant, cheap, and are found in numerous natural products and pharmaceuticals.^21^ To demonstrate the broad applicability of the protocol, its capability to couple structurally complex natural products and pharmaceuticals with olefins 2a and 3a was investigated. As summarized in Table 3, many amino acids and peptides such as Z-Gly-OH (51), Z-D-Asp-OMe (52), Boc-Gly-Gly-OH (53), and Z-Gly-Gly-Gly-OH (54) were examined, giving the corresponding products in good yields. Moreover, a series of naturally occurring alkyl carboxylic acids including stearic acid (55), oleic acid (56), erucic acid (57), and linoleic acid (58) smoothly reacted with olefins 2a and 3a to afford good yields of the 1,4-carboimination products. Notably, the contained C–C double bonds remained untouched. (1*S*)-(−)-Camphanic acid (59) and diprogulic acid (63) were also smoothly engaged in the 1,4-carboimination reaction. Pharmaceuticals like sulbactam (60), gemfibrozil (61), and ciprofibrate (62) were successfully utilized as well, generating the desired imines in good yields. Finally, various complex steroid molecules, such as oleanolic acid (64), dehydroabietic acid (65), glycyrrhetinic acid (66), dehydrocholic acid (67), and lithocholic acid (68) were also suitable for this transformation. Overall, these examples clearly highlight the broadness of the developed reaction and its compatibility with complex molecules.

**Table 3:** Modification of natural products and drugs^a^^,^^b^

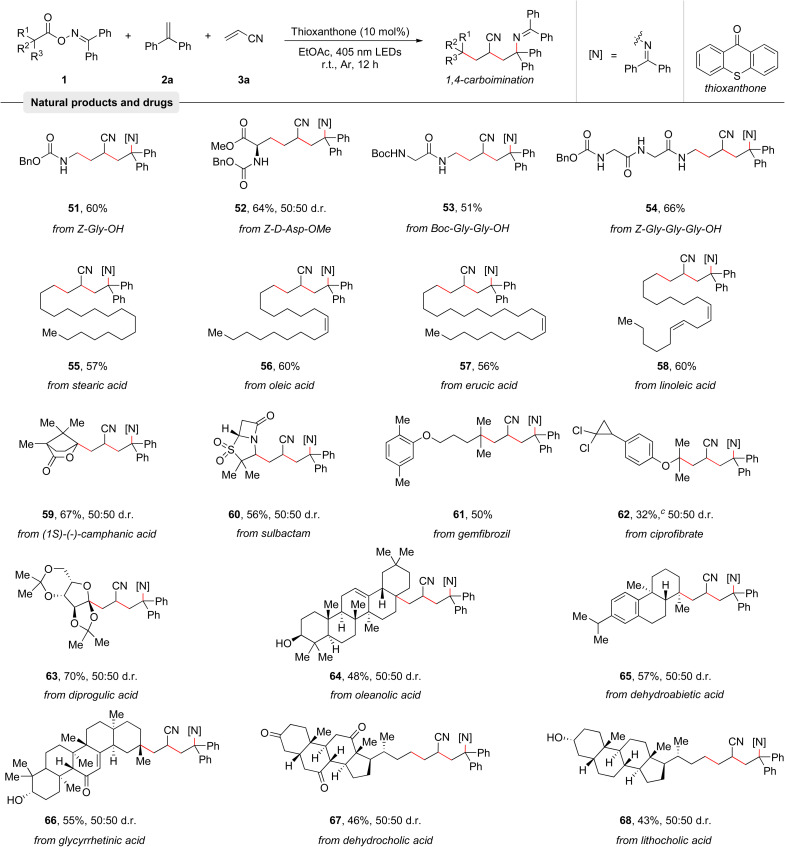

a Reaction conditions: oxime esters 1 (0.3 mmol, 1.5 equiv.), ethene-1,1-diyldibenzene 2a (0.2 mmol, 1.0 equiv.), acrylonitrile 3a (0.4 mmol, 2.0 equiv.), and thioxanthone (10 mol%) in EtOAc (0.1 M, 2.0 mL), irradiation with 18 W blue LEDs (*λ*_max_ = 405 nm) under argon atmosphere at room temperature for 12 h.

b Isolated yields are given. The d.r. values were determined by ^1^H NMR analysis.

c Oxime ester 1ad (0.2 mmol, 1.0 equiv.), ethene-1,1-diyldibenzene 2a (0.4 mmol, 2.0 equiv.), acrylonitrile 3a (0.4 mmol, 2.0 equiv.) were used.

**Scheme 2:**
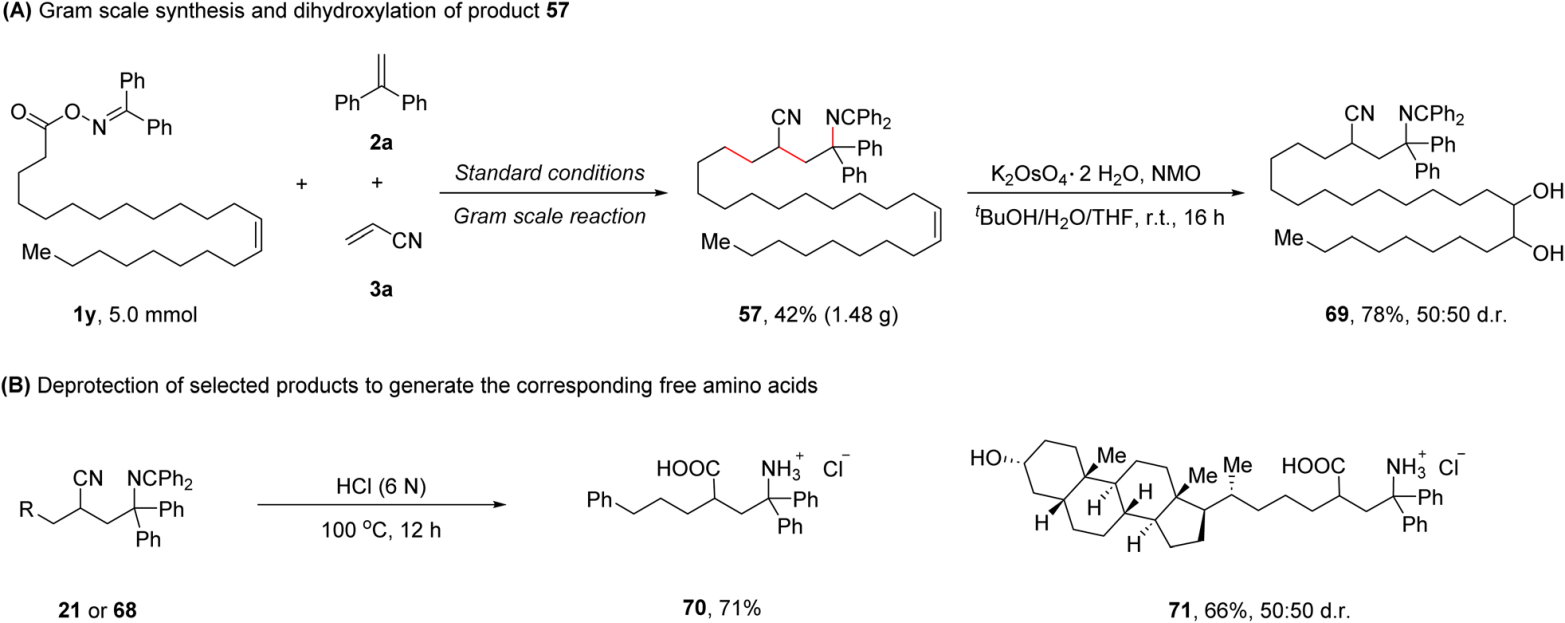
Synthetic applications. (A) Gram scale synthesis and dihydroxylation of product 57. (B) Deprotection of selected products to generate the corresponding free amino acids. NMO: *N*-methylmorpholine *N*-oxide.

To further demonstrate the synthetic practicality of this protocol, a gram scale reaction of 1y, 2a, and 3a was performed, affording the desired product 57 in 42% yield (1.48 g). Dihydroxylation of product 57 was conducted,^8^ furnishing product 69 in 78% yield (Scheme 2A). Treating selected 1,4-carboimination products (21 or 68) with 6 N HCl at 100 °C for 12 h provided the corresponding biologically relevant free γ-amino acids (70 and 71), further highlighting the utility of this method (Scheme 2B).

## Conclusions

In summary, we have developed a novel radical relay 1,4-carboimination across two distinct olefins with alkyl carboxylic acid-derived bifunctional oxime esters. Metal-free energy transfer catalysis enables the well-choreographed, highly chemo- and regioselective formation of three bonds in one single operation. The presented protocol is characterized by a remarkably broad substrate scope with excellent tolerance of sensitive functional groups, therefore allowing for the modular construction of a large variety of highly complex imines which could be easily converted into valuable biologically relevant free γ-amino acids. We believe that this work will be of interest to the synthetic community, and will inspire researchers to further explore other remote 1,*n*-difunctionalization reactions.

## Data availability

The authors declare that the data supporting the findings of this study are available within the paper and the ESI,† as well as from the authors upon request.

## Author contributions

G. T., F. P. and F. G. conceived the project; G. T., F. P. and A. P. performed the experiments and analyzed the data; M.-A. W. and A. L. prepared some of the starting materials; C. D. characterized the X-ray structure of 19; G. T., F. P. and F. G. wrote the manuscript with contributions from all authors; F. G. supervised the project.

## Conflicts of interest

There are no conflicts to declare.

## Supplementary Material

SC-014-D2SC06497A-s001

SC-014-D2SC06497A-s002
