# Supplementary material for: Metal-free photosensitized radical relay 1,4-carboimination across two distinct olefins
Source: Chem Sci. 2023 Feb 3;14(9):2447–54. doi: 10.1039/d2sc06497a (PMC9977457; doi:10.1039/d2sc06497a)
Supplement: SC-014-D2SC06497A-s001 [file SC-014-D2SC06497A-s001.pdf]

Electronic Supplementary Material (ESI) for Chemical Science.
This journal is © The Royal Society of Chemistry 2023

Supplementary Information for

Metal-Free Photosensitized Radical Relay 1,4-Carboimination Across Two Distinct Olefins

Guangying Tan,[+] Fritz Paulus,[+] Alessia Petti, Maxim-Aleksa Wiethoff, Anna Lauer, Constantin Daniliuc and Frank Glorius*

Westfälische Wilhelms-Universität Münster, Organisch-Chemisches Institut, Corrensstrasse 36, 48149 Münster, Germany.

[+]These authors contributed equally to this work

*Correspondence author. glorius@uni-muenster.de

Contents

1. General information 3

1.1. Reagents, solvents, and experimental conditions 3

1.2. Analytical techniques 3

1.3. Compound purification 4

1.4. Photochemical set-up 4

2. Synthesis of starting materials 4

2.1. Preparation of oximes 4

2.2. Preparation of oxime esters 7

2.3. Preparation of styrene derivatives 34

3. Reaction development 36

3.1. Optimization of the reaction conditions 36

3.2. Sensitivity assessment 37

3.3. Additive-based robustness screening 40

3.4. TEMPO trapping experiment 42

3.5. Two component reactions of 1a with 2a or 3a 42

3.6. Detection of side products 44

3.7. Direct excitation experiment 44

3.8. Quantum yield measurement 45

3.9. Determination of the initial reaction rate of the formation of 6 and 7 47

3.10. Mechanistic analysis for 1,4-carboimination of 1a, 2a, and 3a 49

3.11. General procedure for the 1,4-carboimination 49

4. Experimental data for the described substances 50

5. Synthetic application 94

5.1. Large scale synthesis of product 57 94

5.2. Dihydroxylation of product 57 95

5.3. Deprotection of product 21 and 68 95

6. X-Ray analysis 97

7. References 99

8. Copies of ^{1}H and ^{13}C NMR spectra 101

1. General information

1.1. Reagents, solvents, and experimental conditions

All reagents were purchased from Alfa Aesar, Sigma-Aldrich, Merck, TCI, Fluorochem, Combi-blocks, VWR and used without further purification, except otherwise stated. All the solvents were bought from Acros in AcroSeal® bottles and were directly stored under 3 or 4 Å molecular sieves, replacing the collected volume with argon. All reactions were carried out in an oven-dried glassware under an atmosphere of argon using standard Schlenk technique, unless otherwise noted. Solvents for chromatographic purification (pentane and EtOAc) were purchased as technical grade and purified by atmospheric pressure distillation. Reaction temperatures are referred to the temperature of the heating medium, unless otherwise stated.

Photosensitizer thioxanthone was purchased from Fluorochem and used directly without further purification.

1.2. Analytical techniques

NMR-spectra were recorded on a Bruker Avance II 300, Avance II 400, Agilent DD2 500 or DD2 600 spectrometers. All spectral data was acquired at 295 K, except otherwise stated. Deuterated solvents were purchased from Eurisotop ($CDCl_3$, deuteration > 99.8%). ^{1}H and ^{13}C chemicals shifts (δ) are quoted in parts per million (ppm) against tetramethylsilane (TMS, δ = 0.00 ppm) and were internally referenced to residual $CHCl_3$ (7.26 ppm for ^{1}H, 77.16 ppm for ^{13}C) or DMSO (2.50 ppm for ^{1}H, 39.52 ppm for ^{13}C). Coupling constants (*J*) are reported in Hertz (Hz) to the nearest 0.1 Hz. The following abbreviations (or combinations thereof) were used to explain multiplicities: s = singlet, d = doublet, t = triplet, m = multiplet.

High-resolution mass spectra (HRMS) were obtained by the MS service of the Organisch-Chemisches Institut, Westfälische Wilhelms Universität Münster, using electrospray ionisation (ESI) on a Bruker Daltonics, MicroToF spectrometer and calibrated using formate ion clusters.

Thin layer chromatography was carried out on Merck silica gel 60 F_{254} pre-coated aluminium sheets and were visualized using UV light (254 nm) and stained with basic aqueous potassium permanganate.

1.3. Compound purification

Flash chromatography was carried out using silica gel (Acros Organics, 0.035-0.070 mm, 60 Å) under a light positive pressure of argon, eluting with the specified solvent system as mentioned.

1.4. Photochemical set-up

Photochemical reactions, unless otherwise stated, were performed in a Hepatochem EvoluChem™ PhotoRedOx Box Duo device (Fig. S1) and irradiated with two EvoluChem™ Blue LEDs array (18 W, λ_{max} = 405 nm, HCK1012-01-010). The reaction temperature was measured not to exceed room temperature more than 9 °C, using this setup. A 10 mL Schlenk tube equipped with a PTFE-screw cap and a PTFE-coated rare-earth "extra power" oval stirring bar (10 x 5 mm) was used for small scale reactions.

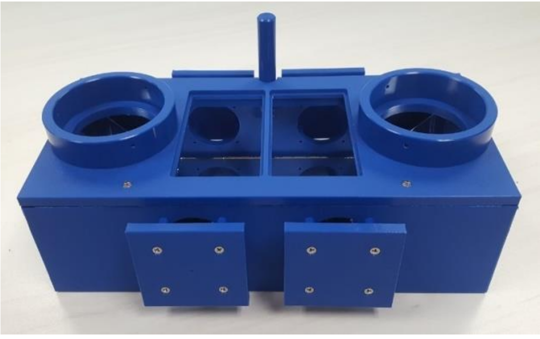

Fig. S1. Experimental set-ups: EvoluChem™ PhotoRedOx Duo (courtesy of EvoluChem™).

2. Synthesis of starting materials

2.1. Preparation of oximes

Diphenylmethanone oxime (S1)

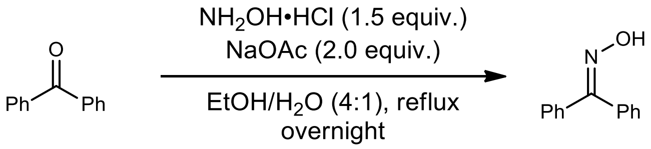

S1

The title compound was prepared similar to a reported procedure.[1] A round-bottom flask was charged with a magnetic stirring bar, sodium acetate (32.8 g, 400 mmol, 2.0 equiv), and hydroxylamine hydrochloride (20.8 g, 300 mmol, 1.5 equiv). A solution of benzophenone (36.4 g, 200 mmol, 1.0 equiv) in EtOH/H_2O (4/1; 400 mL, 0.50 M) was added and the mixture was stirred at 80 °C for 16 h. The mixture was cooled down to r.t. and EtOH was removed under reduced pressure. H_2O (200 mL) was added and the resulting mixture was extracted with EtOAc (3x 200 mL). The combined organic layers were washed with H_2O (2x 100 mL) and brine (100 mL) and were dried over $MgSO_4$. Diphenylmethanone oxime (**S1**; 38.6 g, 196 mmol, 98%) was obtained as a white solid after removal of the solvent under reduced pressure and drying under high vacuum. The isolated product was kept in a fridge under argon due to otherwise observed decomposition. ^{1}H NMR (400 MHz, $CDCl_3$) δ = 7.31 – 7.40 (m, 3H), 7.41 – 7.51 (m, 7H), 8.45 (s, 1H) ppm. ^{13}C NMR (101 MHz, $CDCl_3$) δ = 128.0, 128.4, 128.5, 129.2, 129.4, 129.7, 132.8, 136.3, 158.3 ppm. HRMS (ESI): *m/z* calculated for [$C_{13}H_{11}NONa$] [$M+Na^+$]: 220.0733; found: 220.0732.

(4-Methoxyphenyl)(phenyl)methanone oxime (S2)

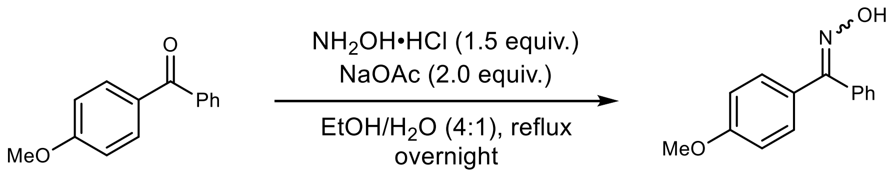

S2, 52:48 *E*/*Z*

The title compound was prepared following an adapted literature procedure.[1] A round-bottom flask was charged with a magnetic stirring bar, sodium acetate (3.28 g, 40.0 mmol, 2.0 equiv), and hydroxylamine hydrochloride (2.08 g, 29.9 mmol, 1.5 equiv). A solution of (4-methoxyphenyl)(phenyl)methanone (4.24 g, 20.0 mmol, 1.0 equiv) in EtOH/H_2O (4/1; 40 mL, 0.50 M) was added and the mixture was stirred at 80 °C for 16 h. The mixture was cooled down to r.t. and EtOH was removed under reduced pressure. H_2O (20 mL) was added and the resulting mixture was extracted with EtOAc (3x 20 mL). The combined organic layers were washed with H_2O (2x 10 mL) and brine (10 mL) and were dried over $MgSO_4$. Oxime **S2** was obtained after column chromatography (pentane/EtOAc = 9/1) as a white solid in 52:48 *E*/*Z*-ratio (3.49 g,

15.4 mmol, 77% yield). ^{1}H NMR (400 MHz, $CDCl_3$) δ = 3.79-3.90 (m, 3H), 6.78-6.92 (m, 1H), 6.94-7.05 (m, 1H), 7.30-7.52 (m, 7H), 8.69 (s, 1H) ppm. ^{13}C NMR (101 MHz, $CDCl_3$) δ = 55.4, 55.5, 113.7, 113.9, 124.8, 128.3, 128.4, 128.4, 128.8, 129.1, 129.3, 129.3, 129.5, 131.3, 133.0, 136.8, 157.7, 157.8, 160.2, 160.8 ppm. HRMS (ESI): *m/z* calculated for $[C_{14}H_{13}NO_2Na]^+$ $[M+Na]^+$: 250.0839; found: 250.0837.

1-Phenylethan-1-one oxime (S3)

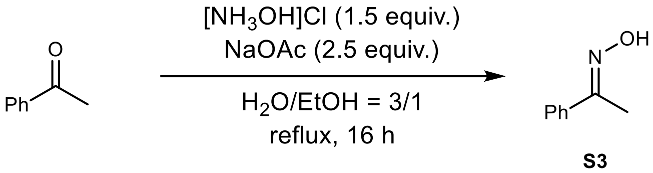

S3

The title compound was prepared similar to a reported procedure.[2] A round-bottom flask was charged with hydroxylammonium chloride (4.17 g, 60.0 mmol, 1.5 equiv), sodium acetate (8.20 g, 100 mmol, 2.5 equiv), acetophenone (4.81 g, 40.0 mmol, 1.0 equiv), distilled water (96 mL), and ethanol (32 mL). After heating to reflux for 7 h, the reaction mixture was cooled to 0 °C. The precipitate was collected by filtration and was then washed with water. The solid was dried and was then purified by column chromatography on silica gel (pentane/ethyl acetate = 3/1 to 1/1). The title compound was obtained as a white solid (4.58 g, 33.9 mmol, 85%). ^{1}H NMR (400 MHz, $CDCl_3$) δ = 2.32 (s, 3H), 7.01 (br, 1H), 7.37-7.44 (m, 3H), 7.60-7.67 (m, 2H) ppm. ^{13}C NMR (101 MHz, $CDCl_3$) δ = 12.5, 126.3, 128.7, 129.6, 136.4, 156.5 ppm.

2.2. Preparation of oxime esters

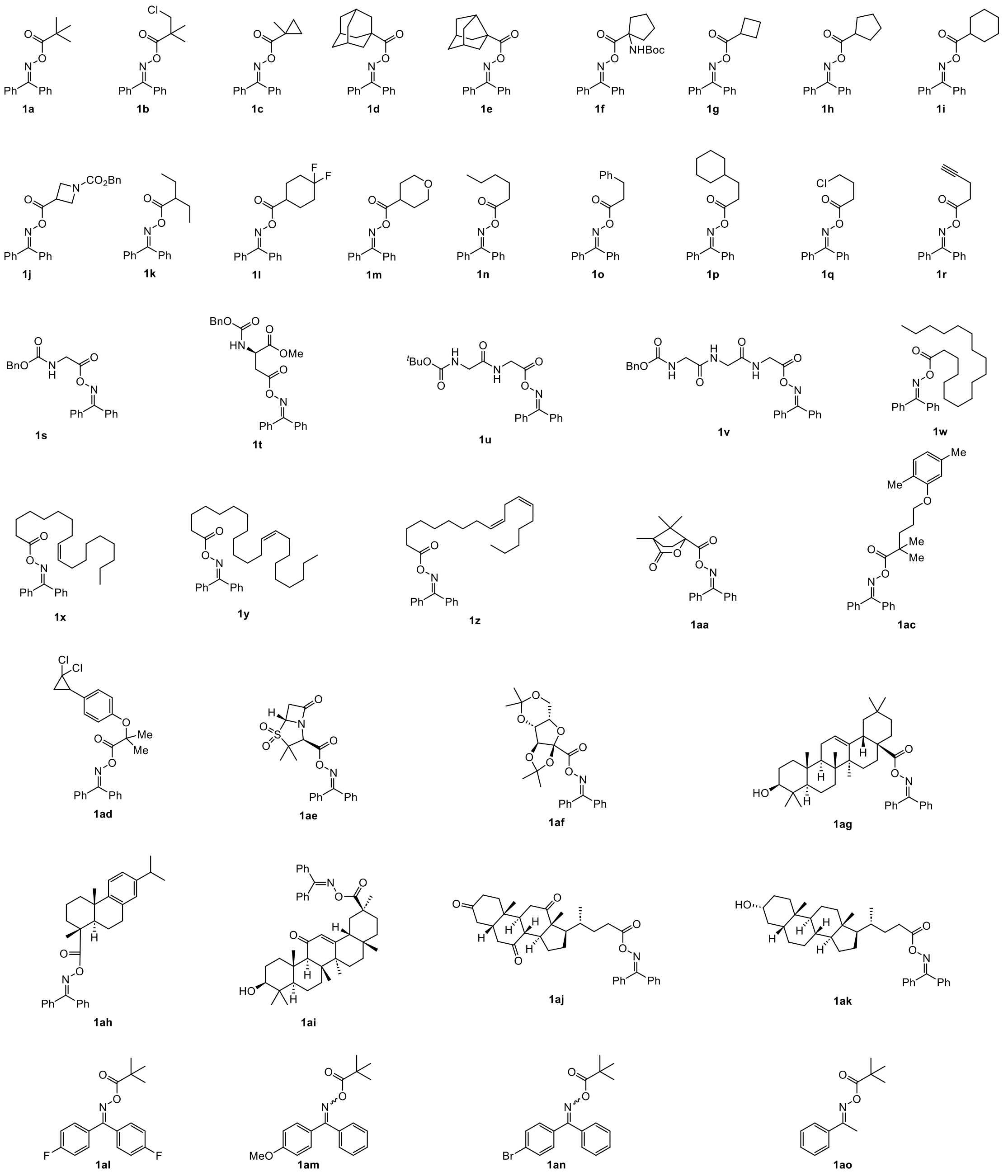

Fig. S2. Synthesized oxime esters.

Preparation of 1a:

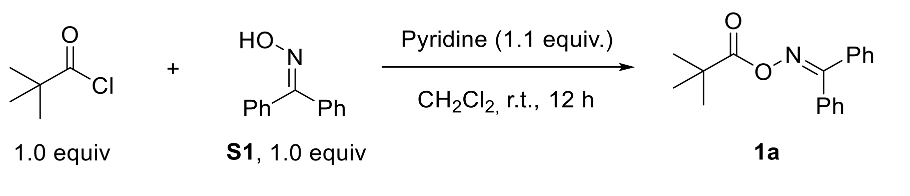

To a solution of diphenylmethanone oxime (**S1**; 1.97 g, 10 mmol, 1.0 equiv.) and pyridine (0.89 mL, 11 mmol, 1.1 equiv.) in dry CH_2Cl_2 (30 mL), pivaloyl chloride (1.23

mL, 10 mmol, 1.0 equiv.) was added dropwise at room temperature under inert atmosphere. The solution was stirred at room temperature overnight. The product was washed with water (100 mL) for three times. The combined organic layers were dried over $MgSO_4$ and the solvent was removed. The crude residue was purified by flash column chromatography on silica (pentane:EtOAc = 40:1, v/v), to afford **1a** as a white solid (2.56 g, 91% yield). ^{1}H NMR (400 MHz, $CDCl_3$) δ = 1.09 (s, 9H), 7.28-7.31 (m, 2H), 7.33-7.37 (m, 2H), 7.41-7.47 (m, 4H), 7.60-7.64 (m, 2H) ppm. ^{13}C NMR (101 MHz, $CDCl_3$) δ = 27.0, 38.5, 128.2, 128.4, 128.6, 129.0, 129.5, 131.0, 132.9, 134.6, 165.7, 175.2 ppm. HRMS (ESI): m/z calculated for [$C_{18}H_{19}NNaO_2$] [M+Na$^+$]: 304.1313, found: 304.1309.

Preparation of other oxime esters from oxime S1:

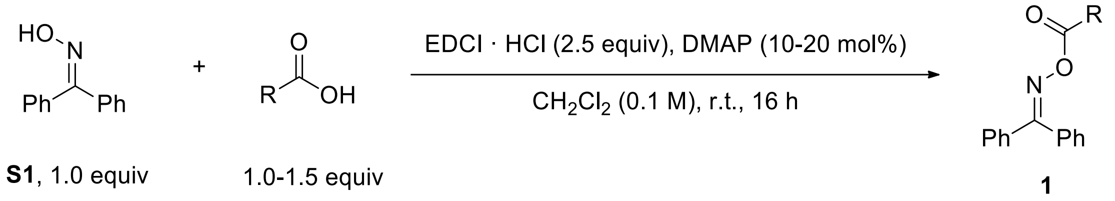

General procedure (**GP1**): Oxime esters **1** were prepared similar to a reported procedure by Glorius and coworkers.[3] An oven-dried Schlenk tube was charged with a PTFE-coated stirring bar and diphenylmethanone oxime (**S1**) (1.0 equiv). The tube was evacuated and backfilled with argon three times and then, dry CH_2Cl_2 (0.10 M) was added. Then, EDCI·HCl (2.5 equiv), carboxylic acid (1.0-1.5 equiv), and DMAP (10-20 mol%) were added successively. The reaction mixture was stirred at ambient temperature for 16 h. Distilled water (50 mL) was added and stirring was continued for 10 mins. The organic layer was separated and the aqueous layer was extracted with CH_2Cl_2 (2x30 mL). All organic layers were combined, dried over Na_2SO_4, and concentrated. Purification of the resulting residue by column chromatography on silica gel using the specified solvent system gave the pure oxime esters **1**.

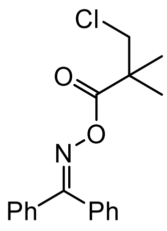

1b

Diphenylmethanone *O*-(3-chloro-2,2-dimethylpropanoyl) oxime (1b)

Following **general procedure GP1**, the title compound was prepared from diphenylmethanone oxime (**S1**) (986 mg, 5.00 mmol, 1.0 equiv), EDCI·HCl (2.40 g, 12.5 mmol, 2.5 equiv), 3-chloropivalic acid (683 mg, 5.00 mmol, 1.0 equiv.), and DMAP (122 mg, 1.00 mmol, 20 mol%) in CH_2Cl_2 (50 mL, 0.10 M). Oxime ester **1b** was obtained after column chromatography (*n*-pentane/EtOAc = 40/1) as a colorless solid (1.09 g, 3.45 mmol, 69%). ^{1}H NMR (400 MHz, $CDCl_3$) δ = 1.18 (s, 6H), 3.43 (s, 2H), 7.29 – 7.39 (m, 4H), 7.40 – 7.52 (m, 4H), 7.59 – 7.66 (m, 2H) ppm. ^{13}C NMR (101 MHz, $CDCl_3$) δ = 23.1, 44.5, 51.6, 128.3, 128.5, 128.5, 129.1, 129.7, 131.2, 132.7, 134.4, 166.2, 171.9 ppm. HRMS (ESI): *m/z* calculated for $[C_{18}H_{18}ClNO_2Na]^+$ $[M+Na]^+$: 338.0918; found: 338.0918.

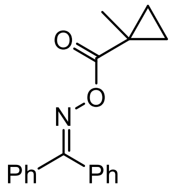

1c

Diphenylmethanone O-(1-methylcyclopropane-1-carbonyl) oxime (1c)

Following **general procedure GP1**, the title compound was prepared from diphenylmethanone oxime (**S1**) (986 mg, 5.00 mmol, 1.0 equiv), EDCI·HCl (2.40 g, 12.5 mmol, 2.5 equiv), 1-methylcyclopropane-1-carboxylic acid (500 mg, 4.99 mmol, 1.0 equiv), and DMAP (122 mg, 1.00 mmol, 20 mol%) in CH_2Cl_2 (50 mL, 0.10 M). Oxime ester **1c** was obtained after column chromatography (*n*-pentane/EtOAc = 30/1) as a colorless solid (879 mg, 3.15 mmol, 63%). ^{1}H NMR (400 MHz, $CDCl_3$) δ = 0.58 – 0.66 (m, 2H), 1.09 – 1.16 (m, 5H), 7.28 – 7.38 (m, 4H), 7.39 – 7.51 (m, 4H), 7.58 – 7.65 (m, 2H) ppm. ^{13}C NMR (101 MHz, $CDCl_3$) δ = 17.1, 18.0, 19.0, 128.2, 128.4,

128.6, 129.0, 129.5, 130.9, 132.9, 134.7, 164.9, 173.0 ppm. HRMS (ESI): *m/z* calculated for $[C_{18}H_{17}NO_2Na]^+$ $[M+Na]^+$: 302.1152; found: 302.1150.

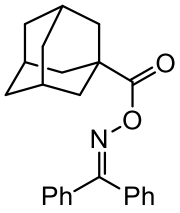

1d

Benzophenone O-((3*r*,5*r*,7*r*)-adamantane-1-carbonyl) oxime (1d)

Following **general procedure GP1**, the title compound was prepared from diphenylmethanone oxime (**S1**) (986 mg, 5.00 mmol, 1.0 equiv), EDCI·HCl (2.40 g, 12.5 mmol, 2.5 equiv), 1-adamantanecarboxylic acid (901 mg, 5.00 mmol, 1.0 equiv), and DMAP (122 mg, 1.00 mmol, 20 mol%) in CH_2Cl_2 (50 mL, 0.10 M). Oxime ester **1d** was obtained after column chromatography (*n*-pentane/EtOAc = 40/1) as a colorless solid (1.48 g, 4.12 mmol, 82%). ^{1}H NMR (400 MHz, $CDCl_3$) δ = 1.58 – 1.71 (m, 6H), 1.75 – 1.81 (m, 6H), 1.90 – 1.97 (m, 3H), 7.28 – 7.39 (m, 4H), 7.40 – 7.50 (m, 4H), 7.58 – 7.65 (m, 2H) ppm. ^{13}C NMR (101 MHz, $CDCl_3$) δ = 27.9, 36.5, 38.6, 40.6, 128.1, 128.4, 128.8, 129.1, 129.5, 131.0, 132.9, 134.8, 165.7, 174.4 ppm. HRMS (ESI): *m/z* calculated for $[C_{24}H_{25}NO_2Na]^+$ $[M+Na]^+$: 382.1778; found: 382.1777.

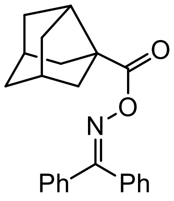

1e

Benzophenone O-((2*R*,3a*s*,5*S*,6a*s*)-octahydro-2,5-methanopentalene-3a-carbonyl) oxime (1e)

Following **general procedure GP1**, the title compound was prepared from diphenylmethanone oxime (**S1**) (986 mg, 5.00 mmol, 1.0 equiv), EDCI·HCl (2.40 g, 12.5 mmol, 2.5 equiv), 3-noradamantanecarboxylic acid (831 mg, 5.00 mmol, 1.0 equiv), and DMAP (122 mg, 1.00 mmol, 20 mol%) in CH_2Cl_2 (50 mL, 0.10 M). Oxime ester **1e** was obtained after column chromatography (*n*-pentane/EtOAc = 30/1) as a colorless solid (1.42 g, 4.11 mmol, 82%). ^{1}H NMR (400 MHz, $CDCl_3$) δ = 1.46 –

1.56 (m, 4H), 1.56 – 1.65 (m, 2H), 1.68 (dd, J = 10.8, 3.0 Hz, 2H), 1.87 – 1.97 (m, 2H), 2.16 – 2.23 (m, 2H), 2.56 (tt, J = 6.7, 1.5 Hz, 1H), 7.28 – 7.32 (m, 2H), 7.33 – 7.38 (m, 2H), 7.40 – 7.47 (m, 4H), 7.59 – 7.67 (m, 2H) ppm. ^{13}C NMR (101 MHz, $CDCl_3$) δ = 34.7, 37.4, 43.5, 44.2, 46.7, 53.1, 128.1, 128.4, 128.7, 129.0, 129.4, 130.9, 133.0, 134.8, 165.2, 174.3 ppm. HRMS (ESI): *m/z* calculated for $[C_{23}H_{23}NO_2Na]^+$ $[M+Na]^+$: 368.1621; found: 368.1619.

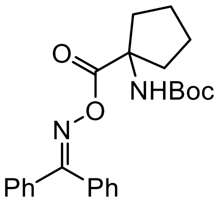

1f

***tert*-Butyl (1-((((diphenylmethylene)amino)oxy)carbonyl)cyclopentyl)carbamate (1f)**

Following **general procedure GP1**, the title compound was prepared from diphenylmethanone oxime (**S1**) (256 mg, 1.30 mmol, 1.0 equiv), EDCI·HCl (619 mg, 3.23 mmol, 2.5 equiv), 1-((*tert*-butoxycarbonyl)amino)cyclopentane-1-carboxylic acid (298 mg, 1.30 mmol, 1.0 equiv), and DMAP (31.8 mg, 0.26 mmol, 20 mol%) in CH_2Cl_2 (10 mL, 0.13 M). Oxime ester **1f** was obtained after column chromatography (*n*-pentane/EtOAc = 6/1) as a colorless solid (325 mg, 0.796 mmol, 61%). ^{1}H NMR (400 MHz, $CDCl_3$) δ = 1.40 (s, 9H), 1.44 – 1.56 (m, 2H), 1.58 – 1.72 (m, 2H), 1.74 – 1.89 (m, 2H), 2.01 – 2.12 (m, 2H), 4.85 (s, 1H), 7.28 – 7.38 (m, 4H), 7.40 – 7.47 (m, 4H), 7.57 – 7.64 (m, 2H) ppm. ^{13}C NMR (101 MHz, $CDCl_3$) δ = 24.6, 28.4, 37.7, 65.7, 80.0, 128.3, 128.5, 128.7, 129.2, 129.6, 131.0, 132.8, 134.6, 155.0, 165.5, 171.8 ppm. HRMS (ESI): *m/z* calculated for $[C_{24}H_{28}N_2O_4Na]^+$ $[M+Na]^+$: 431.1941; found: 431.1937.

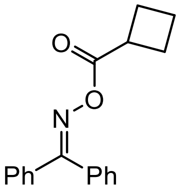

1g

Diphenylmethanone *O*-cyclobutanecarbonyl oxime (1g)

Following **general procedure GP1**, the title compound was prepared from diphenylmethanone oxime (**S1**) (986 mg, 5.00 mmol, 1.0 equiv), EDCI·HCl (2.40 g, 12.5 mmol, 2.5 equiv), cyclobutanecarboxylic acid (501 mg, 5.00 mmol, 1.0 equiv), and DMAP (61.1 mg, 0.500 mmol, 10 mol%) in CH_2Cl_2 (50 mL, 0.10 M). Oxime ester **1g** was obtained after column chromatography (100% *n*-pentane to *n*-pentane/EtOAc = 24/1) as a white solid (893 mg, 3.20 mmol, 64%). ^1H NMR (400 MHz, $CDCl_3$) δ = 1.80 – 1.99 (m, 2H), 2.09 – 2.19 (m, 2H), 2.21 – 2.33 (m, 2H), 3.16 (pd, J = 8.5, 1.1 Hz, 1H), 7.29 – 7.33 (m, 2H), 7.33 – 7.39 (m, 2H), 7.42 – 7.49 (m, 4H), 7.56 – 7.63 (m, 2H) ppm. ^{13}C NMR (101 MHz, $CDCl_3$) δ = 18.7, 25.3, 37.0, 128.2, 128.5, 128.9, 129.1, 129.6, 131.0, 132.8, 134.9, 165.2, 173.0 ppm. HRMS (ESI): *m/z* calculated for $[C_{18}H_{17}NO_2Na]^+$ $[M+Na]^+$: 302.1152; found: 302.1150.

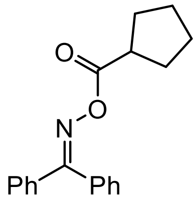

1h

Diphenylmethanone *O*-cyclopentanecarbonyl oxime (1h)

Following **general procedure GP1**, the title compound was prepared from diphenylmethanone oxime (**S1**) (986 mg, 5.00 mmol, 1.0 equiv), EDCI·HCl (2.40 g, 12.5 mmol, 2.5 equiv), cyclopentanecarboxylic acid (571 mg, 5.00 mmol, 1.0 equiv), and DMAP (61.1 mg, 0.500 mmol, 10 mol%) in CH_2Cl_2 (50 mL, 0.10 M). Oxime ester **1h** was obtained after column chromatography (100% *n*-pentane to *n*-pentane/EtOAc = 24/1) as a white solid (601 mg, 2.05 mmol, 41%). ^1H NMR (400 MHz, $CDCl_3$) δ = 1.47 – 1.68 (m, 4H), 1.71 – 1.86 (m, 4H), 2.72 (p, J = 7.9 Hz, 1H), 7.29 – 7.39 (m, 4H), 7.40 – 7.51 (m, 4H), 7.56 – 7.64 (m, 2H). ppm. ^{13}C NMR (101 MHz, $CDCl_3$) δ = 25.8, 29.9, 42.7, 128.3, 128.5, 128.8, 129.1, 129.6, 131.0, 132.9, 134.9, 165.2, 174.1 ppm. HRMS (ESI): *m/z* calculated for $[C_{19}H_{19}NO_2Na]^+$ $[M+Na]^+$: 316.1308; found: 316.1307.

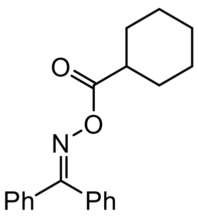

1i

Diphenylmethanone *O*-cyclohexanecarbonyl oxime (1i)

Following **general procedure GP1**, the title compound was prepared from diphenylmethanone oxime (**S1**) (986 mg, 5.00 mmol, 1.0 equiv), EDCI·HCl (2.40 g, 12.5 mmol, 2.5 equiv), cyclohexanecarboxylic acid (962 mg, 7.5 mmol, 1.5 equiv), and DMAP (122 mg, 1.00 mmol, 20 mol%) in CH_2Cl_2 (50 mL, 0.10 M). Oxime ester **1i** was obtained after column chromatography (*n*-pentane/EtOAc = 50/1 to 10/1) as a white solid (1.45 g, 4.72 mmol, 94%). ^{1}H NMR (400 MHz, $CDCl_3$) δ = 1.14 – 1.28 (m, 3H), 1.35 – 1.47 (m, 2H), 1.55 – 1.62 (m, 1H), 1.62 – 1.74 (m, 2H), 1.74 – 1.85 (m, 2H), 2.31 (tt, *J* = 11.1, 3.7 Hz, 1H), 7.28 – 7.39 (m, 4H), 7.41 – 7.51 (m, 4H), 7.56 – 7.64 (m, 2H) ppm. ^{13}C NMR (101 MHz, $CDCl_3$) δ = 25.4, 25.8, 28.8, 42.2, 128.3, 128.5, 128.8, 129.1, 129.6, 131.0, 132.9, 134.9, 165.4, 173.3 ppm. HRMS (ESI): *m/z* calculated for $[C_{20}H_{21}NO_2Na]^+$ $[M+Na]^+$: 330.1465; found: 330.1464.

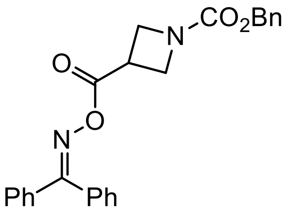

1j

Benzyl 3-((((diphenylmethylene)amino)oxy)carbonyl)azetidine-1-carboxylate (1j)

Following **general procedure GP1**, the title compound was prepared from diphenylmethanone oxime (**S1**) (128 mg, 0.649 mmol, 1.0 equiv), EDCI·HCl (309 mg, 1.61 mmol, 2.5 equiv), 1-((benzyloxy)carbonyl)azetidine-3-carboxylic acid (153 mg, 0.650 mmol, 1.0 equiv), and DMAP (15.9 mg, 0.130 mmol, 20 mol%) in CH_2Cl_2 (10 mL, 65 mM). Oxime ester **1j** was obtained after column chromatography (*n*-pentane/EtOAc = 3/1) as a colorless oil (142 mg, 0.343 mmol, 53%). ^{1}H NMR (400 MHz, $CDCl_3$) δ = 3.47 (p, *J* = 7.6 Hz, 1H), 4.09 – 4.20 (m, 4H), 5.09 (s, 2H), 7.26

– 7.31 (m, 2H), 7.31 – 7.41 (m, 7H), 7.42 – 7.50 (m, 4H), 7.54 – 7.62 (m, 2H) ppm. ^{13}C NMR (101 MHz, $CDCl_3$) δ = 31.8, 51.7, 66.9, 128.1, 128.3, 128.4, 128.6, 128.7, 129.2, 130.0, 131.4, 132.3, 134.4, 136.6, 156.2, 166.1, 170.2 ppm. HRMS (ESI): *m/z* calculated for $[C_{25}H_{22}N_2O_4Na]^+$ $[M+Na]^+$: 437.1472; found: 437.1464.

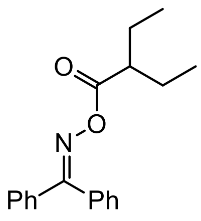

1k

Diphenylmethanone *O*-(2-ethylbutanoyl) oxime (1k)

Following **general procedure GP1**, the title compound was prepared from diphenylmethanone oxime (**S1**) (986 mg, 5.00 mmol, 1.0 equiv), EDCI·HCl (2.40 g, 12.5 mmol, 2.5 equiv), 2-ethylbutanoic acid (581 mg, 5.00 mmol, 1.0 equiv), and DMAP (61.1 mg, 0.500 mmol, 10 mol%) in CH_2Cl_2 (50 mL, 0.10 M). Oxime ester **1k** was obtained after column chromatography (*n*-pentane/EtOAc = 50/1 to 30/1) as a white solid (573 mg, 1.94 mmol, 39%). ^{1}H NMR (400 MHz, $CDCl_3$) δ = 0.84 (t, J = 7.4 Hz, 6H), 1.39 – 1.63 (m, 4H), 2.18 (tt, J = 8.6, 5.5 Hz, 1H), 7.28 – 7.33 (m, 2H), 7.33 – 7.39 (m, 2H), 7.41 – 7.49 (m, 4H), 7.58 – 7.65 (m, 2H) ppm. ^{13}C NMR (101 MHz, $CDCl_3$) δ = 11.8, 25.0, 48.0, 128.2, 128.5, 128.7, 129.1, 129.5, 131.0, 133.0, 134.8, 165.5, 173.3 ppm. HRMS (ESI): *m/z* calculated for $[C_{19}H_{21}NO_2Na]^+$ $[M+Na]^+$: 318.1465; found: 318.1462.

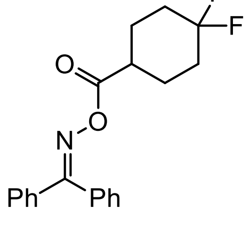

1l

Diphenylmethanone *O*-(4,4-difluorocyclohexane-1-carbonyl) oxime (1l)

Following **general procedure GP1**, the title compound was prepared from diphenylmethanone oxime (**S1**) (986 mg, 5.00 mmol, 1.0 equiv), EDCI·HCl (2.40 g, 12.5 mmol, 2.5 equiv), 4,4-difluorocyclohexane-1-carboxylic acid (1.23 g, 7.5 mmol,

1.5 equiv), and DMAP (122 mg, 1.00 mmol, 20 mol%) in CH_2Cl_2 (50 mL, 0.10 M). Oxime ester **1l** was obtained after column chromatography (*n*-pentane/EtOAc = 30/1 to 5/1) as a white solid (1.51 g, 4.40 mmol, 88%). ^{1}H NMR (400 MHz, $CDCl_3$) δ = 1.63 – 1.92 (m, 6H), 1.93 – 2.06 (m, 2H), 2.39 – 2.49 (m, 1H), 7.27 – 7.32 (m, 2H), 7.34 – 7.40 (m, 2H), 7.42 – 7.53 (m, 4H), 7.55 – 7.64 (m, 2H) ppm. ^{13}C NMR (101 MHz, $CDCl_3$) δ = 24.6 – 25.1 (m), 32.4 (t, J = 24.5 Hz), 39.5, 122.6 (t, J = 241.1 Hz), 128.4, 128.6, 128.6, 129.1, 129.8, 131.3, 132.8, 134.5, 166.1, 171.4 ppm. ^{19}F{^{1}H} NMR (377 MHz, $CDCl_3$) δ = -98.9 (d, J = 237.2 Hz), -95.02 (d, J = 236.9 Hz) ppm. HRMS (ESI): *m/z* calculated for $[C_{20}H_{19}NO_2F_2Na]^+$ $[M+Na]^+$: 366.1276; found: 366.1272.

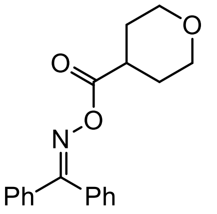

1m

Benzophenone *O*-tetrahydro-2*H*-pyran-4-carbonyl oxime (1m)

Following **general procedure GP1**, the title compound was prepared from diphenylmethanone oxime (**S1**) (986 mg, 5.00 mmol, 1.0 equiv), EDCI·HCl (2.40 g, 12.5 mmol, 2.5 equiv), tetrahydro-2*H*-pyran-4-carboxylic acid (0.98 g, 7.5 mmol, 1.5 equiv), and DMAP (122 mg, 1.00 mmol, 20 mol%) in CH_2Cl_2 (50 mL, 0.10 M). Oxime ester **1m** was obtained after column chromatography (*n*-pentane/EtOAc = 20/1 to 10/1) as a white solid (1.20 g, 78%). ^{1}H NMR (400 MHz, $CDCl_3$) δ = 1.68-1.73 (m, 4H), 2.51-2.57 (m, 1H), 3.31-3.38 (m, 2H), 3.83-3.89 (m, 2H), 7.26-7.31 (m, 2H), 7.32-7.37 (m, 2H), 7.41-7.48 (m, 4H), 7.57-7.60 (m, 2H) ppm. ^{13}C NMR (101 MHz, $CDCl_3$) δ = 28.3, 39.2, 66.9, 128.3, 128.5, 128.6, 129.0, 129.7, 131.1, 132.7, 134.6, 165.8, 171.5 ppm. HRMS (ESI): *m/z* calculated for $[C_{19}H_{19}NNaO_3]^+$ $[M+Na]^+$: 332.1263; found: 332.1263.

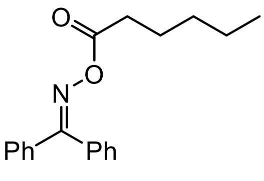

1n

Diphenylmethanone *O*-hexanoyl oxime (1n)

Following **general procedure GP1**, the title compound was prepared from diphenylmethanone oxime (**S1**) (986 mg, 5.00 mmol, 1.0 equiv), EDCI·HCl (2.40 g, 12.5 mmol, 2.5 equiv), hexanoic acid (582 mg, 5.01 mmol, 1.0 equiv), and DMAP (61.1 mg, 0.500 mmol, 10 mol%) in CH_2Cl_2 (50 mL, 0.10 M). Oxime ester **1n** was obtained after column chromatography (*n*-pentane/EtOAc = 50/1 → 30/1) as a white solid (744 mg, 2.52 mmol, 50%). ^{1}H NMR (400 MHz, $CDCl_3$) δ = 0.80 – 0.93 (m, 3H), 1.19 – 1.34 (m, 4H), 1.52 – 1.64 (m, 2H), 2.32 (t, J = 7.5 Hz, 2H), 7.29 – 7.40 (m, 4H), 7.42 – 7.49 (m, 4H), 7.56 – 7.64 (m, 2H) ppm. ^{13}C NMR (101 MHz, $CDCl_3$) δ = 14.0, 22.4, 24.5, 31.3, 33.1, 128.3, 128.5, 128.9, 129.2, 129.7, 131.0, 132.9, 134.9, 165.0, 171.4 ppm. HRMS (ESI): *m/z* calculated for $[C_{19}H_{21}NO_2Na]^+$ $[M+Na]^+$: 318.1465; found: 318.1460.

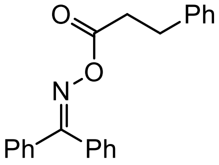

1o

Benzophenone *O*-(3-phenylpropanoyl) oxime (1o)

Following **general procedure GP1**, the title compound was prepared from diphenylmethanone oxime (**S1**) (986 mg, 5.00 mmol, 1.0 equiv), EDCI·HCl (2.40 g, 12.5 mmol, 2.5 equiv), 3-phenylpropanoic acid (1.13 g, 7.49 mmol, 1.5 equiv), and DMAP (122 mg, 1.00 mmol, 20 mol%) in CH_2Cl_2 (50 mL, 0.10 M). Oxime ester **1o** was obtained after column chromatography (*n*-pentane/EtOAc = 40/1 to 30/1) as a white solid (1.15 g, 70%). ^{1}H NMR (400 MHz, $CDCl_3$) δ = 2.65-2.69 (m, 2H), 2.93-2.97 (m, 2H), 7.16-7.31 (m, 7H), 7.35-7.39 (m, 2H), 7.42-7.50 (m, 4H), 7.58-7.62 (m, 2H) ppm. ^{13}C NMR (101 MHz, $CDCl_3$) δ = 30.8, 34.8, 126.4, 128.26, 128.27, 128.42, 128.46, 128.47, 128.6, 128.9, 129.1, 129.68, 129.70, 131.0, 132.6, 134.8, 140.3, 165.0, 170.5 ppm. HRMS (ESI): *m/z* calculated for $[C_{22}H_{19}NNaO_2]^+$ $[M+Na]^+$: 352.1313; found: 352.1313.

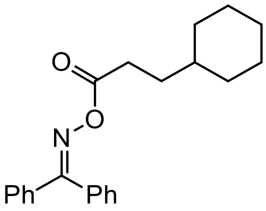

1p

Diphenylmethanone *O*-(3-cyclohexylpropanoyl) oxime (1p)

Following **general procedure GP1**, the title compound was prepared from diphenylmethanone oxime (**S1**) (986 mg, 5.00 mmol, 1.0 equiv), EDCI·HCl (2.40 g, 12.5 mmol, 2.5 equiv), 3-cyclohexylpropanoic acid (781 mg, 5.00 mmol, 1.0 equiv), and DMAP (122 mg, 1.00 mmol, 20 mol%) in CH_2Cl_2 (50 ml, 0.10 M). Oxime ester **1p** was obtained after column chromatography (*n*-pentane/EtOAc = 30/1) as a colorless solid (1.54 g, 4.59 mmol, 92%). ^{1}H NMR (400 MHz, $CDCl_3$) δ = 0.76 – 0.91 (m, 2H), 1.05 – 1.27 (m, 4H), 1.48 (q, J = 7.3 Hz, 2H), 1.57 – 1.72 (m, 5H), 2.33 (t, J = 7.7 Hz, 2H), 7.29 – 7.39 (m, 4H), 7.40 – 7.51 (m, 4H), 7.56 – 7.63 (m, 2H) ppm. ^{13}C NMR (101 MHz, $CDCl_3$) δ = 26.2, 26.6, 30.6, 32.1, 32.9, 37.0, 128.2, 128.4, 128.8, 129.1, 129.6, 130.9, 132.8, 134.9, 164.9, 171.5 ppm. HRMS (ESI): *m/z* calculated for $[C_{22}H_{25}NO_2Na]^+$ $[M+Na]^+$: 358.1778; found: 358.1780.

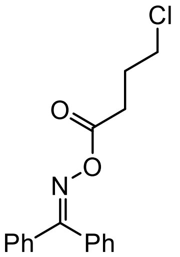

1q

Diphenylmethanone *O*-(4-chlorobutanoyl) oxime (1q)

Following **general procedure GP1**, the title compound was prepared from diphenylmethanone oxime (**S1**) (986 mg, 5.00 mmol, 1.0 equiv), EDCI·HCl (2.40 g, 12.5 mmol, 2.5 equiv), 4-chlorobutanoic acid (613 mg, 5.00 mmol, 1.0 equiv), and DMAP (61.1 mg, 0.500 mmol, 10 mol%) in CH_2Cl_2 (50 mL, 0.10 M). Oxime ester **1q** was obtained after column chromatography (*n*-pentane/EtOAc = 30/1 to 10/1) as a yellow oil (1.26 g, 4.18 mmol, 84%). ^{1}H NMR (400 MHz, $CDCl_3$) δ = 2.01 – 2.13 (m, 2H), 2.54 (t, J = 7.1 Hz, 2H), 3.54 (t, J = 6.3 Hz, 2H), 7.29 – 7.40 (m, 4H), 7.42 – 7.52

(m, 4H), 7.55 – 7.63 (m, 2H) ppm. ^{13}C NMR (101 MHz, $CDCl_3$) δ = 27.5, 30.1, 44.0, 128.4, 128.6, 128.8, 129.2, 129.8, 131.1, 132.7, 134.7, 165.4, 170.4 ppm. HRMS (ESI): *m/z* calculated for $[C_{17}H_{16}NO_2ClNa]^+$ $[M+Na]^+$: 324.0762; found: 324.0760.

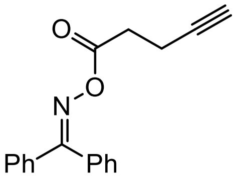

1r

Diphenylmethanone *O*-pent-4-ynoyl oxime (1r)

Following **general procedure GP1**, the title compound was prepared from diphenylmethanone oxime (**S1**) (986 mg, 5.00 mmol, 1.0 equiv), EDCI·HCl (2.40 g, 12.5 mmol, 2.5 equiv), pent-4-ynoic acid (491 mg, 5.01 mmol, 1.0 equiv), and DMAP (122 mg, 1.00 mmol, 20 mol%) in CH_2Cl_2 (50 ml, 0.10 M). Oxime ester **1r** was obtained after column chromatography (*n*-pentane/EtOAc = 40/1) as a colorless solid (690 mg, 2.49 mmol, 50%). ^{1}H NMR (400 MHz, $CDCl_3$) δ = 1.96 (t, J = 2.6 Hz, 1H), 2.42 – 2.53 (m, 2H), 2.55 – 2.64 (m, 2H), 7.30 – 7.39 (m, 4H), 7.41 – 7.52 (m, 4H), 7.55 – 7.63 (m, 2H). ppm. ^{13}C NMR (101 MHz, $CDCl_3$) δ = 14.3, 32.3, 69.4, 82.2, 128.3, 128.5, 128.9, 129.1, 129.8, 131.1, 132.5, 134.7, 165.3, 169.4 ppm. HRMS (ESI): *m/z* calculated for $[C_{18}H_{15}NO_2Na]^+$ $[M+Na]^+$: 300.0995; found: 300.0990.

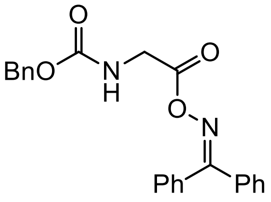

1s

Benzyl (2-(((diphenylmethylene)amino)oxy)-2-oxoethyl)carbamate (1s)

Following **general procedure GP1**, the title compound was prepared from diphenylmethanone oxime (**1**) (986 mg, 5.00 mmol, 1.0 equiv), EDCI·HCl (2.39 g, 12.5 mmol, 2.5 equiv), *N*-benzyloxycarbonyl glycine (1.05 g, 5.00 mmol, 1.0 equiv.) and DMAP (122 mg, 1.00 mmol, 0.2 equiv.) in CH_2Cl_2 (20 ml, 0.10 m). Oxime ester **1s** was obtained after column chromatography (pentane/EtOAc = 7/1 to 5/1, v/v) as a colorless oil (1.28 g, 3.30 mmol, 66%). ^{1}H NMR (400 MHz, $CDCl_3$): δ = 4.10-4.12 (m,

2H), 5.11 (s, 2H), 5.37-5.40 (m, 1H), 7.30-7.39 (m, 9H), 7.44-7.47 (m, 4H), 7.56-7.58 (m, 2H) ppm. ^{13}C NMR (101 MHz, CDCl$_3$): δ = 42.2, 67.2, 128.2, 128.3, 128.4, 128.5, 128.6, 128.9, 129.2, 130.0, 131.2, 132.1, 134.4, 136.3, 165.6, 168.6 ppm. HRMS (ESI): *m/z* calculated for [$C_{23}H_{20}N_2O_4$] [M+Na]$^+$: 411.1315; found: 411.1317.

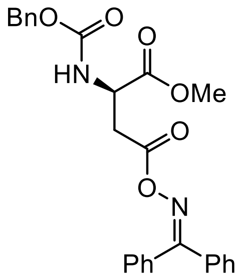

1t

Methyl (*R*)-2-(((benzyloxy)carbonyl)amino)-4-(((diphenylmethylene)amino)oxy)-4-oxobutanoate (1t)

Following **general procedure GP1**, the title compound was prepared from diphenylmethanone oxime (**S1**) (395 mg, 2.00 mmol, 1.0 equiv), EDCI·HCl (958 mg, 5.00 mmol, 2.5 equiv), Z-D-Asp-OMe (562 mg, 2.00 mmol, 1.0 equiv), and DMAP (48.9 mg, 0.400 mmol, 20 mol%) in CH_2Cl_2 (10 ml, 0.20 M). Oxime ester **1t** was obtained after column chromatography (*n*-pentane/EtOAc = 3/1) as a colorless solid (803 mg, 1.74 mmol, 87%). ^{1}H NMR (400 MHz, CDCl$_3$) δ = 2.93 (dd, J = 17.4, 4.5 Hz, 1H), 3.12 (dd, J = 17.5, 4.6 Hz, 1H), 3.73 (s, 3H), 4.64 (dt, J = 8.8, 4.5 Hz, 1H), 5.11 (s, 2H), 5.72 (d, J = 8.5 Hz, 1H), 7.27 – 7.40 (m, 9H), 7.40 – 7.51 (m, 4H), 7.53 – 7.60 (m, 2H) ppm. ^{13}C NMR (101 MHz, CDCl$_3$) δ = 35.6, 50.3, 53.0, 67.2, 128.2, 128.3, 128.4, 128.5, 128.6, 128.8, 129.1, 129.9, 131.2, 132.3, 134.5, 136.2, 156.0, 165.5, 169.1, 170.9 ppm. HRMS (ESI): *m/z* calculated for [$C_{26}H_{24}N_2O_6Na$]$^+$ [M+Na]$^+$: 483.1527; found: 483.1527.

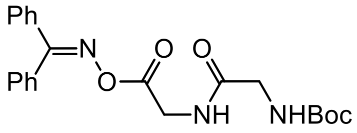

1u

***tert*-Butyl (2-((2-(((diphenylmethylene)amino)oxy)-2-oxoethyl)amino)-2-oxoethyl)carbamate (1u)**

Following **general procedure GP1**, the title compound was prepared from diphenylmethanone oxime (**S1**) (986 mg, 5.00 mmol, 1.0 equiv), EDCI·HCl (2.40 g, 12.5 mmol, 2.5 equiv), N-Boc-glycylglycine (1.16 g, 4.99 mmol, 1.0 equiv), and DMAP (61.1 mg, 0.500 mmol, 10 mol%) in CH_2Cl_2 (50 mL, 0.10 M). Oxime ester **1u** was obtained after column chromatography (100% *n*-pentane to *n*-pentane/EtOAc = 1/2) as a white solid (709 mg, 1.72 mmol, 34%). ^{1}H NMR (400 MHz, $CDCl_3$) δ = 1.46 (s, 9H), 3.80 (d, J = 5.9 Hz, 2H), 4.18 (d, J = 5.3 Hz, 2H), 5.10 (s, 1H), 6.64 (t, J = 5.3 Hz, 1H), 7.28 – 7.33 (m, 2H), 7.34 – 7.41 (m, 2H), 7.42 – 7.53 (m, 4H), 7.53 – 7.60 (m, 2H) ppm. ^{13}C NMR (101 MHz, $CDCl_3$) δ = 28.4, 40.6, 44.2, 80.5, 128.4, 128.6, 128.9, 129.2, 130.0, 131.3, 132.1, 134.4, 156.1, 165.7, 168.4, 169.9 ppm. HRMS (ESI): *m/z* calculated for $[C_{22}H_{25}N_3O_5Na]^+$ $[M+Na]^+$: 434.1686; found: 434.1687.

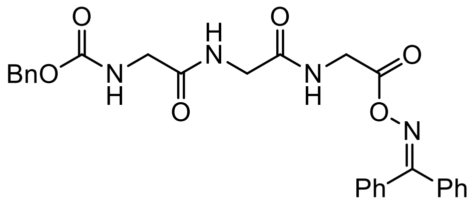

1v

Benzyl (4,7,10-trioxo-1,1-diphenyl-3-oxa-2,6,9-triazaundec-1-en-11-yl)carbamate (1v)

Following **general procedure GP1**, the title compound was prepared from diphenylmethanone oxime (**S1**) (296 mg, 1.50 mmol, 1.0 equiv), EDCI·HCl (719 mg, 3.75 mmol, 2.5 equiv), ((benzyloxy)carbonyl)glycylglycylglycine (485 mg, 1.50 mmol, 1.0 equiv.), and DMAP (36.7 mg, 0.300 mmol, 20 mol%) in CH_2Cl_2 (10 mL, 0.15 M). Oxime ester **1v** was obtained after column chromatography *n*-pentane/EtOAc = 1/6 to 1/9) as a colorless solid (563 mg, 1.12 mmol, 75%). ^{1}H NMR (400 MHz, $CDCl_3$) δ = 3.84 (d, J = 5.8 Hz, 2H), 3.93 (d, J = 5.7 Hz, 2H), 4.03 (d, J = 5.5 Hz, 2H), 5.06 (s, 2H), 5.97 (t, J = 5.7 Hz, 1H), 7.23 – 7.38 (m, 11H), 7.40 – 7.49 (m, 4H), 7.49 – 7.54 (m, 2H) ppm. ^{13}C NMR (101 MHz, $CDCl_3$) δ = 40.5, 42.8, 44.6, 67.3, 128.1, 128.3, 128.4, 128.6, 128.6, 129.0, 129.1, 130.0, 131.3, 132.0, 134.4, 136.3, 157.1, 165.7, 168.6, 169.7, 170.3 ppm. HRMS (ESI): *m/z* calculated for $[C_{27}H_{26}N_4O_6Na]^+$ $[M+Na]^+$: 525.1745; found: 525.1739.

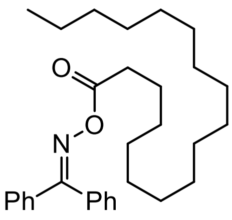

1w

Diphenylmethanone *O*-stearoyl oxime (1w)

Following **general procedure GP1**, the title compound was prepared from diphenylmethanone oxime (**S1**) (1.97 g, 9.99 mmol, 1.0 equiv), EDCI·HCl (4.79 g, 25.0 mmol, 2.5 equiv), stearic acid (2.84 g, 9.98 mmol, 1.0 equiv), and DMAP (244 mg, 2.00 mmol, 20 mol%) in CH_2Cl_2 (100 mL, 0.10 M). Oxime ester **1w** was obtained after column chromatography (*n*-pentane/EtOAc = 30/1) as a colorless solid (4.10 g, 8.84 mmol, 88%). ^{1}H NMR (400 MHz, $CDCl_3$) δ = 0.88 (t, 3H), 1.20 – 1.30 (m, 28H), 1.53 – 1.62 (m, 2H), 2.32 (t, J = 7.5 Hz, 2H), 7.28 – 7.33 (m, 2H), 7.33 – 7.38 (m, 2H), 7.42 – 7.48 (m, 4H), 7.57 – 7.61 (m, 2H) ppm. ^{13}C NMR (101 MHz, $CDCl_3$) δ = 14.2, 22.8, 24.8, 29.2, 29.3, 29.5, 29.5, 29.7, 29.8, 29.8, 29.8, 32.1, 33.2, 128.3, 128.5, 128.9, 129.1, 129.6, 131.0, 132.9, 134.9, 165.0, 171.3 ppm. HRMS (ESI): *m/z* calculated for $[C_{31}H_{45}NO_2Na]^+$ $[M+Na]^+$: 486.3343; found: 486.3341.

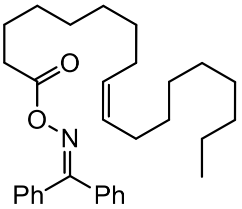

1x

Diphenylmethanone *O*-oleoyl oxime (1x)

Following **general procedure GP1**, the title compound was prepared from diphenylmethanone oxime (**S1**) (1.97 g, 9.99 mmol, 1.0 equiv), EDCI·HCl (4.79 g, 25.0 mmol, 2.5 equiv), oleic acid (2.82 g, 9.98 mmol, 1.0 equiv), and DMAP (244 mg, 2.00 mmol, 20 mol%) in CH_2Cl_2 (100 mL, 0.10 M). Oxime ester **1x** was obtained after column chromatography (*n*-pentane/EtOAc = 30/1) as a colorless solid (3.84 g, 8.32 mmol, 83%). ^{1}H NMR (400 MHz, $CDCl_3$) δ = 0.83 – 0.92 (m, 3H), 1.18 – 1.41 (m, 20H), 1.51 – 1.65 (m, 2H), 1.96 – 2.08 (m, 4H), 2.32 (t, J = 7.4 Hz, 2H), 5.29 –

5.43 (m, 2H), 7.29 – 7.38 (m, 4H), 7.40 – 7.49 (m, 4H), 7.57 – 7.63 (m, 2H) ppm. ^{13}C NMR (101 MHz, $CDCl_3$) δ = 14.2, 22.7, 24.7, 27.2, 27.3, 29.0, 29.1, 29.1, 29.4, 29.6, 29.7, 29.8, 31.9, 33.0, 128.2, 128.4, 128.8, 129.0, 129.5, 129.7, 130.0, 130.9, 132.7, 134.8, 164.9, 171.1 ppm. HRMS (ESI): *m/z* calculated for $[C_{31}H_{43}NO_2Na]^+$ $[M+Na]^+$: 484.3186; found: 484.3182.

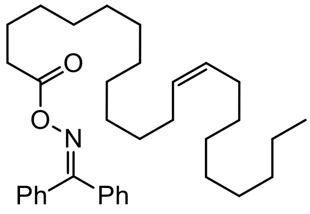

1y

(*Z*)-Diphenylmethanone *O*-docos-13-enoyl oxime (1y)

Following **general procedure GP1**, the title compound was prepared from diphenylmethanone oxime (**S1**) (1.97 g, 9.99 mmol, 1.0 equiv), EDCI·HCl (4.79 g, 25.0 mmol, 2.5 equiv), erucic acid (3.36 g, 9.92 mmol, 1.0 equiv), and DMAP (244 mg, 2.00 mmol, 20 mol%) in CH_2Cl_2 (100 mL, 0.10 M). Oxime ester **1y** was obtained after column chromatography (*n*-pentane/EtOAc = 40/1) as a colorless solid (3.43 g, 6.62 mmol, 66%). ^{1}H NMR (400 MHz, $CDCl_3$) δ = 0.85 – 0.91 (m, 3H), 1.16 – 1.39 (m, 28H), 1.53 – 1.62 (m, 2H), 1.94 – 2.09 (m, 4H), 2.32 (t, J = 7.5 Hz, 2H), 5.30 – 5.40 (m, 2H), 7.29 – 7.33 (m, 2H), 7.33 – 7.39 (m, 2H), 7.41 – 7.48 (m, 4H), 7.58 – 7.61 (m, 2H) ppm. ^{13}C NMR (101 MHz, $CDCl_3$) δ = 14.2, 22.8, 24.8, 27.3, 29.2, 29.3, 29.4, 29.5, 29.6, 29.7, 29.7, 29.7, 29.9, 29.9, 32.0, 33.2, 128.3, 128.5, 128.9, 129.1, 129.6, 130.0, 130.0, 131.0, 132.8, 134.9, 165.0, 171.3 ppm. HRMS (ESI): *m/z* calculated for $[C_{35}H_{51}NO_2Na]^+$ $[M+Na]^+$: 540.3812; found: 540.3811.

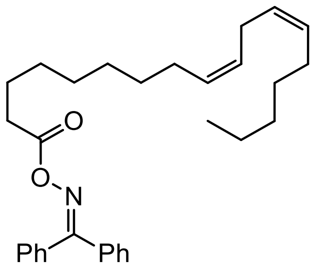

1z

Diphenylmethanone *O*-((9*Z*,12*Z*)-octadeca-9,12-dienoyl) oxime (1z)

Following **general procedure GP1**, the title compound was prepared from diphenylmethanone oxime (**S1**) (1.97 g, 9.99 mmol, 1.0 equiv), EDCI·HCl (4.79 g, 25.0 mmol, 2.5 equiv), linoleic acid (2.80 g, 9.98 mmol, 1.0 equiv), and DMAP (244 mg, 2.00 mmol, 20 mol%) in CH_2Cl_2 (100 mL, 0.10 M). Oxime ester **1z** was obtained after column chromatography (*n*-pentane/EtOAc = 40/1) as a colorless solid (3.91 g, 8.51 mmol, 85%). ^{1}H NMR (400 MHz, $CDCl_3$) δ = 0.83 – 0.96 (m, 3H), 1.19 – 1.42 (m, 14H), 1.54 – 1.64 (m, 2H), 1.98 – 2.12 (m, 4H), 2.32 (t, J = 7.5 Hz, 2H), 2.73 – 2.84 (m, 2H), 5.29 – 5.43 (m, 4H), 7.29 – 7.39 (m, 4H), 7.40 – 7.49 (m, 4H), 7.57 – 7.63 (m, 2H) ppm. ^{13}C NMR (101 MHz, $CDCl_3$) δ = 14.1, 22.6, 24.8, 25.7, 27.3, 27.3, 29.1, 29.1, 29.2, 29.4, 29.6, 31.6, 33.1, 128.0, 128.1, 128.2, 128.4, 128.8, 129.1, 129.6, 130.1, 130.3, 130.9, 132.8, 134.9, 164.9, 171.2 ppm. HRMS (ESI): *m/z* calculated for $[C_{31}H_{41}NO_2Na]^+$ $[M+Na]^+$: 482.2998; found: 482.3025.

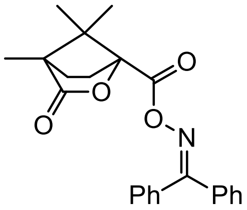

1aa

(1*S*,4*R*)-1-((((Diphenylmethylene)amino)oxy)carbonyl)-4,7,7-trimethyl-2-oxabicyclo[2.2.1]heptan-3-one (1aa)

Following **general procedure GP1**, the title compound was prepared from diphenylmethanone oxime (**S1**) (986 mg, 5.00 mmol, 1.0 equiv), EDCI·HCl (2.40 g, 12.5 mmol, 2.5 equiv), (1*S*)-(−)-camphanic acid (991 mg, 5.00 mmol, 1.0 equiv), and DMAP (122 mg, 1.00 mmol, 20 mol%) in CH_2Cl_2 (50 mL, 0.10 M). Oxime ester **1aa** was obtained after column chromatography (*n*-pentane/EtOAc = 10/1 to 8/1) as a colorless solid (946 mg, 2.51 mmol, 50%). ^{1}H NMR (400 MHz, $CDCl_3$) δ = 0.80 (s, 3H), 0.81 (s, 3H), 1.04 (s, 3H), 1.60 (ddd, J = 13.4, 9.4, 4.3 Hz, 1H), 1.81 (ddd, J = 13.1, 10.7, 4.5 Hz, 1H), 1.94 (ddd, J = 13.6, 9.3, 4.5 Hz, 1H), 2.22 (ddd, J = 13.5, 10.8, 4.3 Hz, 1H), 7.29 – 7.33 (m, 2H), 7.34 – 7.40 (m, 2H), 7.41 – 7.52 (m, 4H), 7.58 – 7.66 (m, 2H) ppm. ^{13}C NMR (101 MHz, $CDCl_3$) δ = 9.7, 16.4, 16.6, 28.9, 30.5, 54.3, 54.7,

90.6, 128.4, 128.6, 128.6, 129.2, 130.0, 131.5, 132.4, 134.0, 164.7, 167.2, 177.9 ppm. HRMS (ESI): *m/z* calculated for $[C_{23}H_{23}NO_4Na]^+$ $[M+Na]^+$: 400.1519; found: 400.1519.

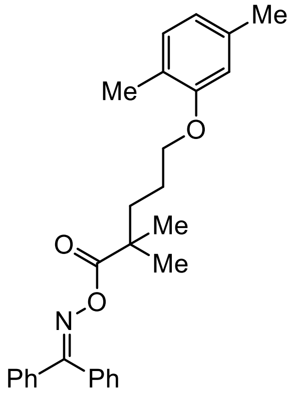

1ac

Diphenylmethanone *O*-(5-(2,5-dimethylphenoxy)-2,2-dimethylpentanoyl) oxime (1ac)

Following **general procedure GP1**, the title compound was prepared from diphenylmethanone oxime (**S1**) (986 mg, 5.00 mmol, 1.0 equiv), EDCI·HCl (2.40 g, 12.5 mmol, 2.5 equiv), gemfibrozil (1.25 g, 4.99 mmol, 1.0 equiv), and DMAP (61.1 mg, 0.500 mmol, 10 mol%) in CH_2Cl_2 (50 mL, 0.10 M). Oxime ester **1ac** was obtained after column chromatography (100% *n*-pentane to *n*-pentane/EtOAc = 24/1) as a colorless oil (1.03 g, 2.40 mmol, 48%). ^{1}H NMR (400 MHz, $CDCl_3$) δ = 1.12 (s, 6H), 1.52 – 1.72 (m, 4H), 2.15 (s, 3H), 2.31 (s, 3H), 3.78 (t, J = 6.1 Hz, 2H), 6.57 (s, 1H), 6.66 (d, J = 7.5 Hz, 1H), 7.00 (d, J = 7.4 Hz, 1H), 7.27 – 7.32 (m, 2H), 7.34 – 7.39 (m, 2H), 7.41 – 7.50 (m, 4H), 7.59 – 7.66 (m, 2H) ppm. ^{13}C NMR (101 MHz, $CDCl_3$) δ = 15.9, 21.6, 25.2, 25.2, 37.1, 42.1, 68.0, 112.1, 120.8, 123.7, 128.3, 128.5, 128.5, 129.1, 129.5, 130.4, 131.1, 133.0, 134.6, 136.6, 157.1, 165.8, 174.6 ppm. HRMS (ESI): *m/z* calculated for $[C_{28}H_{31}NO_3Na]^+$ $[M+Na]^+$: 452.2196; found: 452.2193.

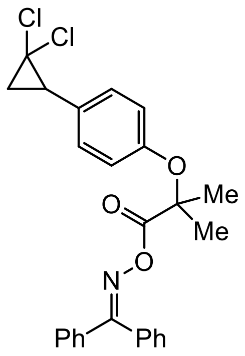

1ad

Diphenylmethanone *O*-(2-(4-(2,2-dichlorocyclopropyl)phenoxy)-2-methylpropanoyl) oxime (1ad)

Following **general procedure GP1**, the title compound was prepared from diphenylmethanone oxime (**S1**) (986 mg, 5.00 mmol, 1.0 equiv), EDCI·HCl (2.40 g, 12.5 mmol, 2.5 equiv), Ciprofibrate (1.88 g, 6.50 mmol, 1.3 equiv), and DMAP (122 mg, 1.00 mmol, 20 mol%) in CH_2Cl_2 (50 mL, 0.10 M). Oxime ester **1ad** was obtained after column chromatography (*n*-pentane /EtOAc = 50/1 to 20/1) as a colorless gum (1.74 g, 3.71 mmol, 74%). ^{1}H NMR (400 MHz, $CDCl_3$) δ = 1.50 (s, 6H), 1.78 (dd, J = 8.3, 7.4 Hz, 1H), 1.94 (dd, J = 10.7, 7.4 Hz, 1H), 2.83 (dd, J = 10.7, 8.3 Hz, 1H), 6.74 – 6.83 (m, 2H), 7.05 – 7.16 (m, 4H), 7.29 – 7.50 (m, 6H), 7.56 – 7.63 (m, 2H) ppm. ^{13}C NMR (101 MHz, $CDCl_3$) δ = 25.4, 25.5, 26.0, 35.0, 61.0, 79.0, 119.0, 128.2, 128.4, 128.6, 128.8, 129.3, 129.7, 129.8, 131.3, 132.3, 134.5, 154.9, 166.7, 171.3 ppm. HRMS (ESI): *m/z* calculated for $[C_{26}H_{23}NO_3Cl_2Na]^+$ $[M+Na]^+$: 490.0947; found: 490.0946.

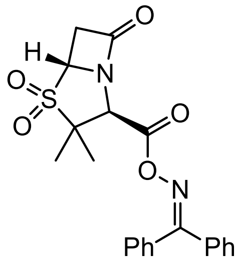

1ae

(2*S*,5*R*)-2-((((Diphenylmethylene)amino)oxy)carbonyl)-3,3-dimethyl-4-thia-1-azabicyclo[3.2.0]heptan-7-one 4,4-dioxide (1ae)

Following **general procedure GP1**, the title compound was prepared from diphenylmethanone oxime (**S1**) (1.97 g, 9.99 mmol, 1.0 equiv), EDCI·HCl (4.79 g,

25.0 mmol, 2.5 equiv), sulbactam (2.33 g, 9.99 mmol, 1.0 equiv.), and DMAP (244 mg, 2.00 mmol, 20 mol%) in CH_2Cl_2 (100 mL, 0.10 M). Oxime ester **1ae** was obtained after column chromatography (*n*-pentane/EtOAc = 30/1 to 3/1) as a colorless solid (2.12 g, 5.14 mmol, 51%). ^{1}H NMR (400 MHz, $CDCl_3$) δ = 1.17 (s, 3H), 1.33 (s, 3H), 3.33 – 3.47 (m, 2H), 4.33 (s, 1H), 4.45 (dd, J = 4.1, 2.3 Hz, 1H), 7.27 – 7.32 (m, 2H), 7.34 – 7.43 (m, 2H), 7.43 – 7.55 (m, 4H), 7.56 – 7.66 (m, 2H) ppm. ^{13}C NMR (101 MHz, $CDCl_3$) δ = 18.0, 20.5, 38.5, 61.1, 62.6, 62.7, 128.2, 128.7, 128.7, 129.1, 130.0, 131.8, 132.5, 133.5, 164.2, 167.3, 170.5 ppm. HRMS (ESI): *m/z* calculated for $[C_{21}H_{20}N_2O_5SNa]^+$ $[M+Na]^+$: 435.0985; found: 435.0984.

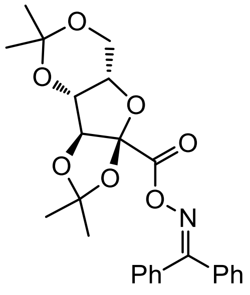

1af

Benzophenone*O*-((3*aS*,3*bR*,7*aS*,8*aR*)-2,2,5,5-tetramethyltetrahydro-3*aH*-[1,3]dioxolo[4',5':4,5]furo[3,2-*d*][1,3]dioxine-8a-carbonyl) oxime (1af)

Following **general procedure GP1**, the title compound was prepared from diphenylmethanone oxime (**S1**) (986 mg, 5.00 mmol, 1.0 equiv), EDCI·HCl (2.40 g, 12.5 mmol, 2.5 equiv), (−)-2,3:4,6-di-*O*-isopropylidene-2-keto-L-gulonic acid monohydrate (1.46 g, 5.00 mmol, 1.0 equiv), and DMAP (122 mg, 1.00 mmol, 20 mol%) in CH_2Cl_2 (50 mL, 0.10 M). Oxime ester **1af** was obtained after column chromatography (*n*-pentane/EtOAc = 5/1) as a colorless solid (548 mg, 1.21 mmol, 24%). ^{1}H NMR (400 MHz, $CDCl_3$) δ = 1.26 (s, 3H), 1.33 (s, 3H), 1.41 (s, 3H), 1.46 (s, 3H), 3.96 – 4.09 (m, 2H), 4.14 (q, J = 2.2 Hz, 1H), 4.28 (d, J = 2.5 Hz, 1H), 4.76 (s, 1H), 7.32 – 7.52 (m, 8H), 7.56 – 7.64 (m, 2H) ppm. ^{13}C NMR (101 MHz, $CDCl_3$) δ = 19.1, 25.6, 27.1, 28.8, 59.9, 72.6, 74.2, 88.0, 97.6, 110.2, 114.5, 128.2, 128.5, 129.5, 129.8, 130.0, 131.1, 132.2, 134.9, 164.2, 166.7 ppm. HRMS (ESI): *m/z* calculated for $[C_{25}H_{27}NO_7Na]^+$ $[M+Na]^+$: 476.1680; found: 476.1680.

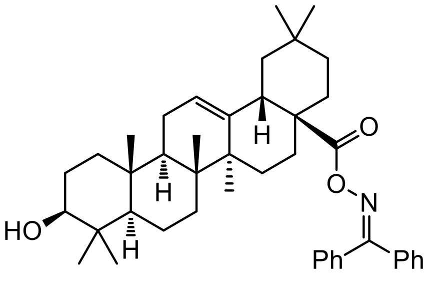

1ag

BenzophenoneO-((4*aS*,6*aS*,6*bR*,8*aR*,10*S*,12*aR*,12*bR*,14*bS*)-10-hydroxy-2,2,6*a*,6*b*,9,9,12*a*-heptamethyl-1,2,3,4,4*a*,5,6,6*a*,6*b*,7,8,8*a*,9,10,11,12,12*a*,12*b*,13,14*b*-icosahydropicene-4*a*-carbonyl) oxime (1ag)

Following **general procedure GP1**, the title compound was prepared from diphenylmethanone oxime (**S1**) (986 mg, 5.00 mmol, 1.0 equiv), EDCI·HCl (2.40 g, 12.5 mmol, 2.5 equiv), oleanolic acid (2.28 g, 4.99 mmol, 1.0 equiv.), and DMAP (122 mg, 1.00 mmol, 20 mol%) in CH_2Cl_2 (50 mL, 0.10 M). Oxime ester **1ag** was obtained after column chromatography (*n*-pentane/EtOAc = 10/1 to 6/1) as a colorless solid (389 mg, 0.612 mmol, 12%). ^{1}H NMR (400 MHz, $CDCl_3$) δ = 0.67 (s, 3H), 0.76 (s, 3H), 0.79 (s, 3H), 0.85 (s, 3H), 0.86 (s, 3H), 0.89 – 1.03 (m, 6H), 1.05 (s, 3H), 1.10 – 1.22 (m, 3H), 1.28 – 1.77 (m, 16H), 1.87 (td, *J* = 13.5, 4.1 Hz, 1H), 2.58 (dd, *J* = 13.7, 4.6 Hz, 1H), 3.19 (dd, *J* = 10.7, 5.0 Hz, 1H), 4.75 (t, *J* = 3.6 Hz, 1H), 7.27 – 7.37 (m, 4H), 7.39 – 7.49 (m, 4H), 7.59 – 7.63 (m, 2H) ppm. ^{13}C NMR (101 MHz, $CDCl_3$) δ = 15.4, 15.7, 17.2, 18.4, 23.1, 23.5, 23.5, 26.0, 27.3, 27.6, 28.2, 30.7, 32.6, 32.7, 33.1, 33.9, 37.1, 38.5, 38.9, 39.4, 40.8, 41.6, 45.9, 46.9, 47.7, 55.3, 79.1, 122.6, 128.2, 128.4, 128.6, 129.0, 129.4, 130.9, 133.3, 134.8, 143.4, 164.8, 174.6 ppm. HRMS (ESI): *m/z* calculated for $[C_{43}H_{57}NO_3Na]^+$ $[M+Na]^+$: 658.4231; found: 658.4229.

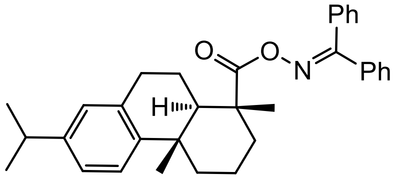

1ah

BenzophenoneO-((1*R*,4*aS*,10*aR*)-7-isopropyl-1,4*a*-dimethyl-1,2,3,4,4*a*,9,10,10*a*-octahydrophenanthrene-1-carbonyl) oxime (1ah)

Following **general procedure GP1**, the title compound was prepared from diphenylmethanone oxime (**S1**) (1.97 g, 9.99 mmol, 1.0 equiv), EDCI·HCl (4.80 g, 25.0 mmol, 2.5 equiv), dehydroabietic acid (3.90 g, 13.0 mmol, 1.3 equiv), and DMAP (244 mg, 2.00 mmol, 20 mol%) in CH_2Cl_2 (100 mL, 0.10 M). Oxime ester **1ah** was obtained after column chromatography (*n*-pentane /EtOAc = 100/1 to 30/1) as a white sticky solid (789 mg, 1.64 mmol, 16%). ^{1}H NMR (400 MHz, $CDCl_3$) δ = 1.12 – 1.18 (m, 6H), 1.25 (dd, *J* = 6.9, 1.1 Hz, 6H), 1.29 – 1.47 (m, 2H), 1.56 – 1.81 (m, 5H), 2.03 (dd, *J* = 12.5, 2.2 Hz, 1H), 2.21 – 2.29 (m, 1H), 2.62 (ddd, *J* = 17.6, 11.2, 7.6 Hz, 1H), 2.75 (dd, *J* = 17.0, 7.1 Hz, 1H), 2.84 (p, *J* = 6.9 Hz, 1H), 6.84 (d, *J* = 2.0 Hz, 1H), 6.98 (dd, *J* = 8.1, 2.0 Hz, 1H), 7.11 (d, *J* = 8.2 Hz, 1H), 7.24 – 7.40 (m, 7H), 7.40 – 7.49 (m, 1H), 7.60 – 7.67 (m, 2H) ppm. ^{13}C NMR (101 MHz, $CDCl_3$) δ = 16.3, 18.6, 21.7, 24.1, 24.2, 25.1, 29.8, 33.6, 36.8, 37.0, 38.0, 44.6, 47.7, 123.9, 124.1, 127.0, 128.2, 128.5, 128.5, 129.1, 129.4, 131.1, 133.0, 134.6, 134.8, 145.7, 146.9, 165.8, 175.5 ppm. HRMS (ESI): *m/z* calculated for $[C_{33}H_{37}NO_2Na]^+$ $[M+Na]^+$: 502.2717; found: 502.2715.

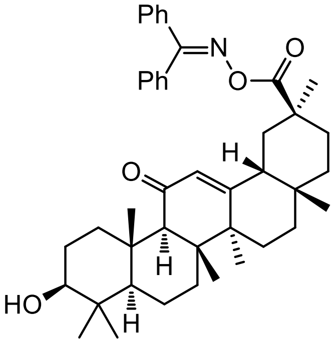

1ai

(2*S*,4*aS*,6*aS*,6*bR*,8*aR*,10*S*,12*aS*,12*bR*,14*bR*)-2-((((Diphenylmethylene)amino)oxy)carbonyl)-10-hydroxy-2,4*a*,6*a*,6*b*,9,9,12*a*-heptamethyl-1,3,4,4*a*,5,6,6*a*,6*b*,7,8,8*a*,9,10,11,12,12*a*,12*b*,14*b*-octadecahydropicen-13(2*H*)-one (1ai)

Following **general procedure GP1**, the title compound was prepared from diphenylmethanone oxime (**S1**) (493 mg, 2.50 mmol, 1.0 equiv), EDCI·HCl (1.19 g, 6.21 mmol, 2.5 equiv), enoxolone (1.18 g, 2.51 mmol, 1.0 equiv), and DMAP (61.1 mg, 0.500 mmol, 20 mol%) in CH_2Cl_2 (25 mL, 0.10 M). Oxime ester **1ai** was obtained after column chromatography (*n*-pentane/EtOAc = 4/1 to 2/1) as a colorless solid (1.04 g,

1.60 mmol, 64%). ^{1}H NMR (400 MHz, $CDCl_3$) δ = 0.63 – 0.71 (m, 1H), 0.76 (s, 3H), 0.80 (s, 3H), 0.91 – 1.01 (m, 5H), 1.06 (s, 3H), 1.09 (s, 3H), 1.12 (s, 3H), 1.14 – 1.32 (m, 7H), 1.34 – 1.84 (m, 11H), 1.95 (td, J = 13.6, 4.5 Hz, 1H), 2.06 (dd, J = 13.6, 3.6 Hz, 1H), 2.28 (s, 1H), 2.79 (dt, J = 13.4, 3.6 Hz, 1H), 3.22 (dd, J = 10.7, 5.7 Hz, 1H), 5.39 (s, 1H), 7.27 – 7.31 (m, 2H), 7.32 – 7.39 (m, 2H), 7.39 – 7.52 (m, 4H), 7.59 – 7.64 (m, 2H) ppm. ^{13}C NMR (101 MHz, $CDCl_3$) δ = 15.7, 16.5, 17.6, 18.8, 23.6, 26.5, 26.5, 27.4, 28.2, 28.4, 28.4, 31.0, 31.9, 32.9, 37.2, 37.7, 39.2, 39.3, 40.9, 43.2, 44.0, 45.4, 48.0, 55.1, 61.9, 78.9, 128.3, 128.5, 128.5, 128.6, 129.0, 129.7, 131.1, 132.8, 134.5, 165.9, 168.7, 173.5, 200.0 ppm. HRMS (ESI): m/z calculated for $[C_{43}H_{55}NO_4Na]^+$ $[M+Na]^+$: 672.4023; found: 672.4024

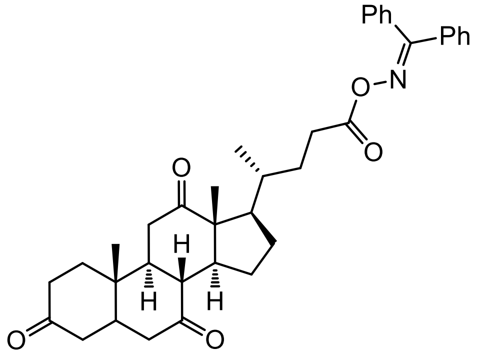

1aj

(8*R*,9*S*,10*S*,13*R*,14*S*,17*R*)-17-((*R*)-5-(((Diphenylmethylene)amino)oxy)-5-oxopentan-2-yl)-10,13-dimethyldecahydro-1*H*-cyclopenta[a]phenanthrene-3,7,12(2*H*,4*H*,8*H*)-trione (1aj)

Following **general procedure GP1**, the title compound was prepared from diphenylmethanone oxime (**S1**) (986 mg, 5.00 mmol, 1.0 equiv), EDCI·HCl (2.40 g, 12.5 mmol, 2.5 equiv), dehydrocholic acid (2.01 g, 4.99 mmol, 1.0 equiv), and DMAP (61.1 mg, 0.500 mmol, 10 mol%) in CH_2Cl_2 (50 mL, 0.10 M). Oxime ester **1aj** was obtained after column chromatography (100% *n*-pentane to 100% EtOAc) as a white solid (1.30 g, 2.23 mmol, 45%). ^{1}H NMR (400 MHz, $CDCl_3$) δ = 0.80 (d, J = 6.4 Hz, 3H), 1.04 (s, 3H), 1.19 – 1.37 (m, 4H), 1.39 (s, 3H), 1.61 (td, J = 14.1, 5.3 Hz, 1H), 1.76 – 2.48 (m, 16H), 2.78 – 2.96 (m, 3H), 7.28 – 7.40 (m, 4H), 7.41 – 7.49 (m, 4H), 7.55 – 7.62 (m, 2H) ppm. ^{13}C NMR (101 MHz, $CDCl_3$) δ = 12.0, 18.7, 22.0, 25.3, 27.6,

30.3, 30.4, 35.4, 35.4, 36.1, 36.6, 38.8, 42.9, 45.1, 45.7, 45.7, 47.0, 49.1, 51.9, 57.0, 128.3, 128.5, 128.9, 129.2, 129.7, 131.0, 132.8, 134.9, 165.0, 171.5, 208.8, 209.1, 212.1 ppm. HRMS (ESI): *m/z* calculated for $[C_{37}H_{43}NO_5Na]^+$ $[M+Na]^+$: 604.3033; found: 604.3032.

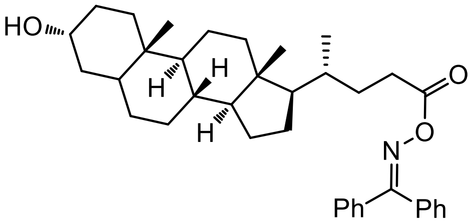

1ak

Benzophenone O-((4*R*)-4-((3*R*,8*R*,9*S*,10*S*,13*R*,14*S*,17*R*)-3-hydroxy-10,13-dimethylhexadecahydro-1*H*-cyclopenta[*a*]phenanthren-17-yl)pentanoyl) oxime (1ak)

Following **general procedure GP1**, the title compound was prepared from diphenylmethanone oxime (**S1**) (986 mg, 5.00 mmol, 1.0 equiv), EDCI·HCl (2.40 g, 12.5 mmol, 2.5 equiv), lithocholic acid (1.88 g, 4.99 mmol, 1.0 equiv), and DMAP (122 mg, 1.00 mmol, 20 mol%) in CH_2Cl_2 (50 mL, 0.10 M). Oxime ester **1ak** was obtained after column chromatography (*n*-pentane /EtOAc = 5/1 to 1/1) as a white solid (1.65 g, 2.97 mmol, 59%). ^{1}H NMR (400 MHz, $CDCl_3$) δ = 0.61 (s, 3H), 0.85 (d, *J* = 6.4 Hz, 3H), 0.91 (s, 3H), 0.95 – 1.44 (m, 17H), 1.47 – 1.58 (m, 3H), 1.62 – 1.89 (m, 6H), 1.93 (dt, *J* = 12.4, 3.0 Hz, 1H), 2.17 – 2.44 (m, 2H), 3.62 (tt, *J* = 11.1, 4.7 Hz, 1H), 7.29 – 7.39 (m, 4H), 7.41 – 7.50 (m, 4H), 7.55 – 7.63 (m, 2H) ppm. ^{13}C NMR (101 MHz, $CDCl_3$) δ = 12.2, 18.3, 20.9, 23.5, 24.3, 26.5, 27.3, 28.2, 30.1, 30.7, 30.9, 34.7, 35.4, 35.5, 36.0, 36.6, 40.3, 40.6, 42.2, 42.9, 56.0, 56.6, 72.0, 128.3, 128.5, 128.9, 129.2, 129.6, 131.0, 132.8, 134.9, 164.9, 171.8 ppm. HRMS (ESI): *m/z* calculated for $[C_{37}H_{49}NO_3Na]^+$ $[M+Na]^+$: 578.3605; found: 578.3602.

Bis(4-fluorophenyl)methanone *O*-pivaloyl oxime (1al)

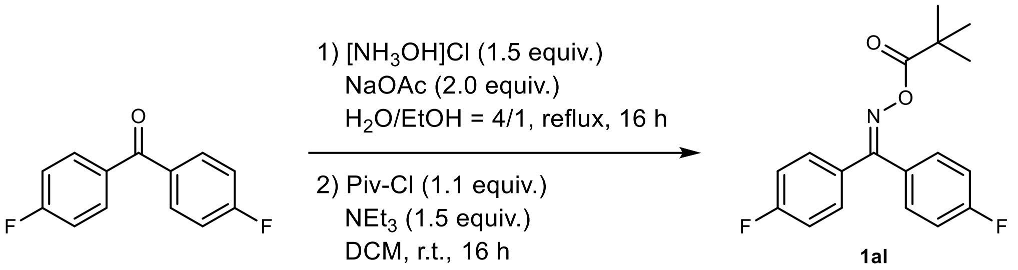

A round-bottom flask was charged with hydroxylammonium chloride (1.04 g, 15.0 mmol, 1.5 equiv), sodium acetate (1.64 g, 20.0 mmol, 2.0 equiv), and bis(4-fluorophenyl)methanone (2.18 g, 9.99 mmol, 1.0 equiv). Distilled water (4 mL), and ethanol (16 mL) were added and the resulting mixture was stirred at reflux for 18 h. After being cooled down to r.t., the ethanol was removed under reduced pressure. Distilled water (10 mL) was added and the mixture was extracted with CH_2Cl_2 (3x 20 mL). All organic layers were combined, washed with distilled water (2x 5 mL), dried over $MgSO_4$, and concentrated under reduced pressure. The residue was dried under vacuum and used for the next reaction step without further purification.

The residue was added to a Schlenk tube and the tube was evacuated and backfilled with argon three times. Dry CH_2Cl_2 (30 mL), triethylamine (1.52 g, 15.0 mmol, 1.5 equiv), and then pivaloyl chloride (dropwise; 1.33 g, 11.0 mmol, 1.1 equiv) were added at r.t., and the resulting mixture was stirred at r.t. for 16 h. Distilled water (20 mL) was added, and stirring was continued for 10 more minutes. The layers were separated and the aqueous layer was extracted with CH_2Cl_2 (3x 50 mL). All organic layers were combined, washed with brine (10 mL), dried over $MgSO_4$, and concentrated under reduced pressure. Column chromatography on silica gel (pentane/ethyl acetate = 50/1 to 15/1) gave the title compound as a white solid (2.39 g, 7.53 mmol, 75%). ^{1}H NMR (400 MHz, $CDCl_3$) δ = 1.11 (s, 9H), 7.01-7.11 (m, 2H), 7.12-7.22 (m, 2H), 7.27-7.34 (m, 2H), 7.55-7.65 (m, 2H) ppm. $^{13}C\{^1H,^{19}F\}$ NMR (126 MHz, $CDCl_3$) δ = 26.9, 38.5, 115.4, 115.6, 128.4, 130.6, 130.8, 131.0, 163.2, 163.5, 164.5, 175.0 ppm. HRMS (ESI): *m/z* calculated for [$C_{18}H_{17}NO_2F_2Na$] [M+Na$^+$]: 340.1120; found: 340.1113.

(4-Methoxyphenyl)(phenyl)methanone *O*-pivaloyl oxime (1am)

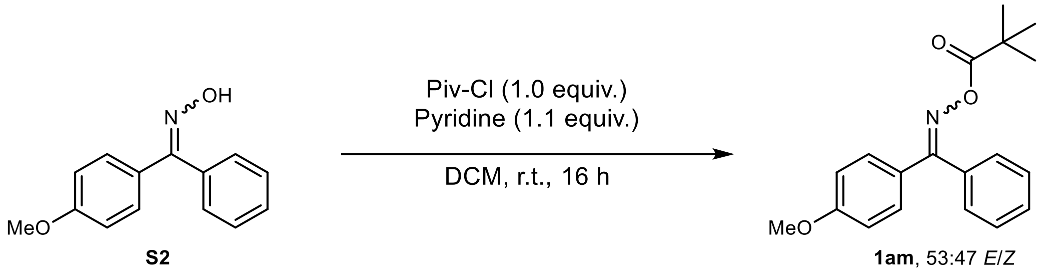

A Schlenk tube was charged with oxime **S2** (1.50 g, 6.60 mmol, 1.0 equiv) and was then evacuated and backfilled with argon three times. Dry CH_2Cl_2 (20 mL), pyridine (574 mg, 7.26 mmol, 1.1 equiv), and then pivaloyl chloride (dropwise; 796 mg, 6.60 mmol, 1.0 equiv) were added at r.t., and the resulting mixture was stirred at r.t. for 16 h. Distilled water (20 mL) was added, and stirring was continued for 10 more minutes. The layers were separated and the aqueous layer was extracted with CH_2Cl_2 (3x 50 mL). All organic layers were combined, washed with brine (10 mL), dried over $MgSO_4$, and concentrated under reduced pressure. The title compound was obtained without further purification as a white solid (1.96 g, 6.29 mmol, 95%) and with an *E*/*Z*-ratio of 53:47. Combined NMR data for both the isomers are reported here. ^{1}H NMR (400 MHz, $CDCl_3$) δ = 1.00-1.22 (m, 9H), 3.77-3.92 (m, 3H), 6.82-6.91 (m, 1H), 6.92-7.00 (m, 1H), 7.24-7.31 (m, 2H), 7.33-7.39 (m, 1H), 7.41-7.48 (m, 2H), 7.53-7.63 (m, 2H) ppm. ^{13}C NMR (101 MHz, $CDCl_3$) δ = 27.1, 27.2, 38.6, 38.7, 55.5, 55.5, 113.5, 113.9, 124.9, 127.0, 128.2, 128.4, 128.5, 129.3, 129.4, 130.7, 130.8, 131.0, 133.2, 135.4, 160.6, 162.0, 165.2, 165.5, 175.5, 175.5 ppm. HRMS (ESI): *m/z* calculated for [$C_{19}H_{21}NO_3Na$] [M+Na$^+$]: 334.1414; found: 334.1408.

(4-Bromophenyl)(phenyl)methanone *O*-pivaloyl oxime (1an)

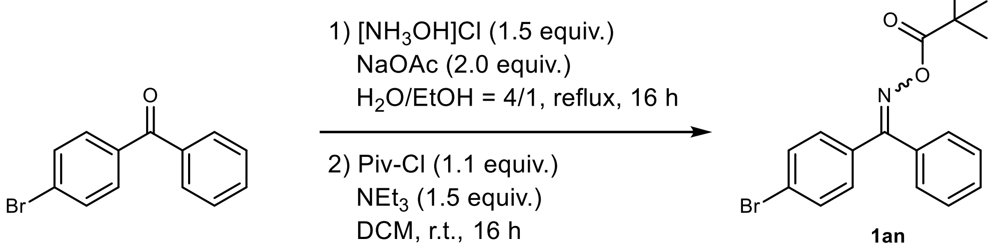

A round-bottom flask was charged with hydroxylammonium chloride (1.04 g, 15.0 mmol, 1.5 equiv), sodium acetate (1.64 g, 20.0 mmol, 2.0 equiv), and (4-

bromophenyl)(phenyl)methanone (2.61 g, 10.0 mmol, 1.0 equiv). Distilled water (4 mL), and ethanol (16 mL) were added and the resulting mixture was stirred at reflux for 18 h. After being cooled down to r.t., the ethanol was removed under reduced pressure. Distilled water (10 mL) was added and the mixture was extracted with CH_2Cl_2 (3x 20 mL). All organic layers were combined, washed with distilled water (2x 5 mL), dried over $MgSO_4$, and concentrated under reduced pressure. The residue was dried under vacuum and used for the next reaction step without further purification.

The residue was added to a Schlenk tube and the tube was evacuated and backfilled with argon three times. Dry CH_2Cl_2 (30 mL), triethylamine (1.52 g, 15.0 mmol, 1.5 equiv), and then pivaloyl chloride (dropwise; 1.33 g, 11.0 mmol, 1.1 equiv) were added at r.t., and the resulting mixture was stirred at r.t. for 16 h. Distilled water (20 mL) was added, and stirring was continued for 10 more minutes. The layers were separated and the aqueous layer was extracted with CH_2Cl_2 (3x 50 mL). All organic layers were combined, washed with brine (10 mL), dried over $MgSO_4$, and concentrated under reduced pressure. Column chromatography on silica gel (pentane/ethyl acetate = 50/1 to 10/1) gave the title compound as a white solid (1.70 g, 4.72 mmol, 47%). ^{1}H NMR (400 MHz, $CDCl_3$) δ = 1.12 (s, 9H), 7.16-7.24 (m, 2H), 7.34-7.40 (m, 2H), 7.43-7.49 (m, 1H), 7.55-7.65 (m, 4H) ppm. ^{13}C NMR (101 MHz, $CDCl_3$) δ = 27.1, 38.6, 124.0, 128.6, 129.0, 130.5, 131.3, 131.6, 131.7, 134.3, 164.5, 175.2 ppm. HRMS (ESI): *m/z* calculated for [$C_{18}H_{18}NO_2BrNa$] [M+Na$^+$]: 382.0413; found: 382.0410.

(*E*)-1-Phenylethan-1-one *O*-pivaloyl oxime (1ao)

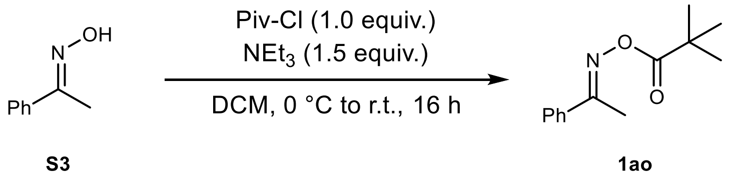

The title compound was prepared similar to a reported procedure.[4] A Schlenk tube was charged with oxime **S3** (676 mg, 5.00 mmol, 1.0 equiv) and was then evacuated and backfilled with argon three times. Then, dry CH_2Cl_2 (10 mL) was added and the mixture was cooled to 0 °C. Dry NEt_3 (759 mg, 7.50 mmol, 1.5 equiv) was added and then, a solution of pivaloyl chloride (603 mg, 5.00 mmol, 1.0 equiv) in dry CH_2Cl_2 (3 mL) was

added dropwise. The mixture was stirred at r.t. for 1 h and was then quenched by the addition of distilled water (10 mL). The phases were separated and the aqueous layer was extracted with CH_2Cl_2 (3x 30 mL). All organic phases were combined, washed with sat. aq. $NaHCO_3$ (10 mL) and brine (10 mL). After drying over $MgSO_4$ and removal of the solvent under reduced pressure, the crude was purified by column chromatography on silica gel (pentane/ethyl acetate = 10/1 to 7/1) to afford the title compound as a white solid (1.05 g, 4.79 mmol, 96%). ^{1}H NMR (400 MHz, $CDCl_3$) δ = 1.34 (s, 9H), 2.39 (s, 3H), 7.34-7.49 (m, 3H), 7.72-7.80 (m, 2H) ppm. ^{13}C NMR (101 MHz, $CDCl_3$) δ = 14.5, 27.5, 39.0, 127.2, 128.7, 130.7, 135.1, 163.3, 175.2 ppm.

2.3. Preparation of styrene derivatives

General procedure for the synthesis of olefins 2b-2e (GP2)

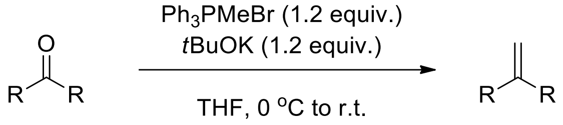

General procedure (**GP2**):[5] To a flame-dried 25 mL round-bottom flask, Ph_3PMeBr (1.2 equiv) was added. Under argon atmosphere, 10 mL of tetrahydrofuran and *t*-BuOK (1.2 equiv) were added at 0 °C. After 1 h, ketone (1.0 equiv) was added at room temperature. When the raw materials are exhausted, the reaction was quenched with ammonium chloride solution, extracted with petroleum ether (20 mL × 3), dried over anhydrous sodium sulfate, and then purified by flash column chromatography (pentane).

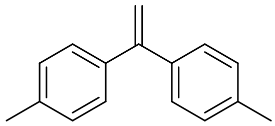

2b

4,4'-(Ethene-1,1-diyl)bis(methylbenzene) (2b)

Following the GP2, **2b** was obtained as a white solid (239 mg, 57% yield). ^{1}H NMR (400 MHz, $CDCl_3$): δ = 2.38 (s, 6H), 5.40 (s, 2H), 7.14 – 7.16 (m, 4H), 7.24 – 7.26 (m, 4H) ppm. ^{13}C NMR (101 MHz, $CDCl_3$) δ = 21.2, 113.0, 128.2, 128.8, 137.4, 138.8, 149.8 ppm.

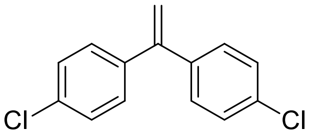

2c

4,4'-(Ethene-1,1-diyl)bis(chlorobenzene) (2c)

Following the GP2, **2c** was obtained as a white solid (502 mg, 73% yield). ^{1}H NMR (400 MHz, $CDCl_3$): δ = 5.46 (s, 2H), 7.23 – 7.26 (m, 4H), 7.29 – 7.32 (m, 4H) ppm. ^{13}C NMR (101 MHz, $CDCl_3$) δ = 115.2, 128.6, 129.6, 134.0, 139.5, 148.0 ppm.

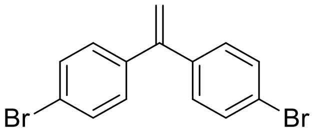

2d

4,4'-(Ethene-1,1-diyl)bis(bromobenzene) (2d)

Following the GP2, **2d** was obtained as a white solid (134 mg, 40% yield). ^{1}H NMR (400 MHz, $CDCl_3$): δ = 5.46 (s, 2H), 7.17 – 7.20 (m, 4H), 7.45 – 7.48 (m, 4H) ppm. ^{13}C NMR (101 MHz, $CDCl_3$) δ = 115.3, 122.2, 129.9, 131.6, 140.0, 148.1 ppm.

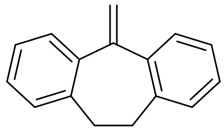

2e

5-Methylene-10,11-dihydro-5*H*-dibenzo[*a,d*][7]annulene (2e)

Following the GP2, **2e** was obtained as a white solid (274 mg, 66% yield). ^{1}H NMR (400 MHz, $CDCl_3$): δ = 3.26 (s, 4H), 5.54 (s, 2H), 7.22 – 7.24 (m, 2H), 7.27 – 7.34 (m, 4H), 7.46 – 7.49 (m, 2H) ppm. ^{13}C NMR (101 MHz, $CDCl_3$) δ = 33.4, 117.6, 126.3, 127.8, 128.3, 129.0, 138.5, 141.3, 152.0 ppm.

2-Tosylethyl 4-vinylbenzoate (2m)

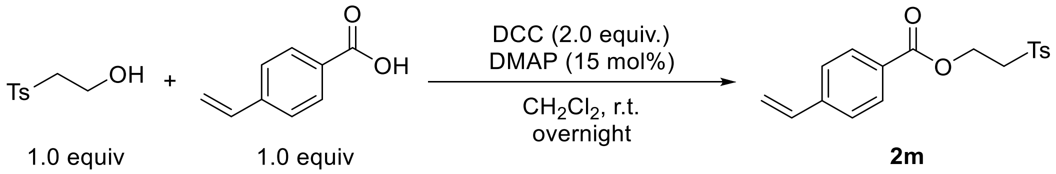

The title compound was prepared similar to an adapted literature procedure.[6] To a stirred solution of carboxylic acid (444 mg, 3.00 mmol, 1.0 equiv.) and DCC

(dicyclohexylcarbodiimide) (1.24 g, 6.01 mmol, 2.0 equiv.) in CH_2Cl_2 (25 mL) were added DMAP (4-dimethylaminopyridine) (55.0 mg, 0.450 mmol, 15 mol%) and 2-tosylethan-1-ol (600 mg, 3.00 mmol, 1.0 equiv.). The reaction mixture was stirred at room temperature overnight. After filtration, the filtrate was concentrated in vacuo and the residue was purified by column chromatography (pentane/EtOAc = 10/1) to afford ester **2m** as a white solid (510 mg, 1.54 mmol, 51% yield). ^1H NMR (400 MHz, $CDCl_3$) δ = 2.34 (s, 3H), 3.59 (t, J = 5.9 Hz, 2H), 4.64 (t, J = 5.9 Hz, 2H), 5.45 – 5.33 (m, 1H), 5.86 (dd, J = 17.6, 0.7 Hz, 1H), 6.73 (dd, J = 17.6, 10.9 Hz, 1H), 7.30 – 7.26 (m, 2H), 7.39 – 7.34 (m, 2H), 7.70 – 7.64 (m, 2H), 7.83 – 7.76 (m, 2H) ppm. ^{13}C NMR (101 MHz, $CDCl_3$) δ = 21.7, 55.4, 58.5, 117.0, 126.1, 128.2, 130.1, 130.2, 136.0, 136.7, 142.4, 145.1, 165.7 ppm. HRMS (ESI): *m/z* calculated for $[C_{18}H_{18}O_4SNa]^+$ $[M+Na]^+$: 353.0818; found: 353.0813.

3. Reaction development

3.1. Optimization of the reaction conditions

Table S1. Optimization of the reaction conditions[a,b]

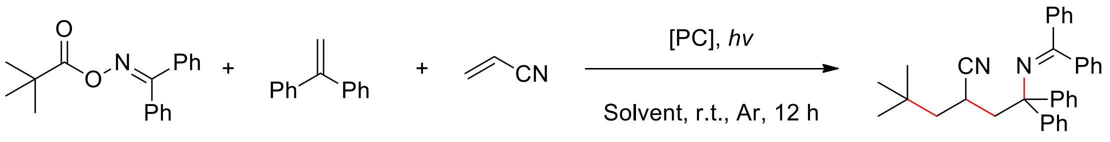

1a 2a 3a 4

Entry	Photocatalyst	Blue light (nm)	**1a** (mmol)	**2a** (mmol)	**3a** (mmol)	Solvent (0.1 M)	Yield of **4** (%)[b]
1	Thioxanthone	405	0.3	0.2	0.4	EtOAc	63, 60[c]
2	-	405	0.3	0.2	0.4	EtOAc	N.R.
3	Thioxanthone	-	0.3	0.2	0.4	EtOAc	N.R.
4	Thioxanthone	450	0.3	0.2	0.4	EtOAc	N.R.
5	Thioxanthone	405	0.3	0.2	0.4	CH_2Cl_2	56
6	Thioxanthone	405	0.3	0.2	0.4	Acetone	54
7	Thioxanthone	405	0.3	0.2	0.4	MeCN	57

8	Thioxanthone	405	0.3	0.2	0.4	DCE	48
9	[Ru (bpy)$_3$](PF$_6$)$_2$	450	0.3	0.2	0.4	EtOAc	N.R.
10	[Ir-F]	450	0.3	0.2	0.4	EtOAc	50
11	Thioxanthone	405	0.3	0.2	0.4	EtOAc (0.05 M)	53
12	Thioxanthone	405	0.3	0.2	0.3	EtOAc	58
13	Thioxanthone	405	0.2	0.4	0.4	EtOAc	53

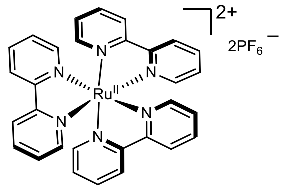

[Ru (bpy)$_3$](PF$_6$)$_2$

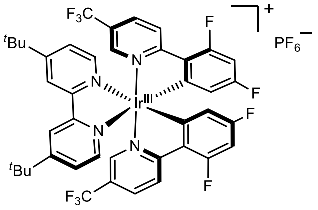

[Ir(dF(CF$_3$)ppy)$_2$(dtbbpy)](PF$_6$)

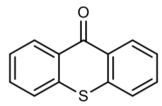

Thioxanthone

[a] Reaction conditions: A mixture of **1a**, **2a**, **3a**, photocatalyst (10 mol% for thioxanthone, 1 mol% for others), and solvent (0.1 or 0.05 M) was irradiated by blue LEDs ((18 W, λ_{max} = 405 nm; 30 w, λ_{max} = 450 nm) under argon at room temperature for 12 h. [b] NMR yields were given. [c] Isolated yields were given. [Ir-F]: [Ir(dF(CF$_3$)ppy)$_2$(dtbbpy)](PF$_6$).

3.2. Sensitivity assessment

To evaluate the sensitivity of the reaction with respect to small changes in the reaction conditions, a sensitivity assessment according to a procedure from Glorius was conducted.[7]

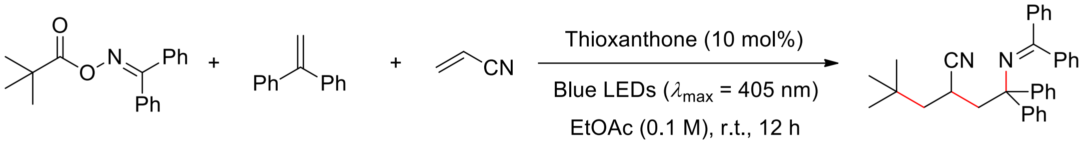

1a (1.5 equiv) **2a** (1.0 equiv) **3a** (2.0 equiv) **4**

Preparation of stock solution 1 (oxime ester 1a): A Schlenk tube was charged with a magnetic stirring bar and oxime ester **1a** (633 mg, 2.25 mmol, 1.5 equiv). The tube was evacuated and backfilled with argon three times and dry EtOAc (6.0 mL) was added.

The mixture was stirred until a clear solution was obtained. The effect of the oxime ester **1a** on the volume of the stock solution was neglected in the following.

Preparation of stock solution 2 (1,1-diphenylethylene (2a) and acrylonitrile (3a)): A Schlenk tube was charged with a magnetic stirring bar and was evacuated and backfilled with argon three times. 1,1-Diphenylethylene (**2a**, 271 mg, 1.50 mmol, 1.0 equiv), acrylonitrile (**3a,** 160 mg, 3.02 mmol, 2.0 equiv), and dry EtOAc (6.0 mL) were added and the solution was stirred for 5 minutes to ensure complete mixing. The effect of alkenes **2a** and **3a** on the volume of the stock solution was neglected in the following.

Reaction set-up: 11 Schlenk tubes were each charged with a magnetic stirring bar and thioxanthone (each 2.1 mg, 9.9 µmol, 10 mol%). The tubes were evacuated and backfilled with argon three times. To each tube, **stock solution 1** (0.40 mL), **stock solution 2** (0.40 mL), and dry EtOAc (0.20 mL) were added. The tubes were sealed and irradiated at 405 nm for 12 h (r.t.) in the standard photoreactor. Deviations from this procedure are described below. Then, the NMR yields were determined using CH_2Br_2 as internal standard.

Large scale reaction: A Schlenk tube was charged with a magnetic stirring bar, oxime ester **1a** (422 mg, 1.50 mmol, 1.5 equiv), and thioxanthone (21.2 mg, 99.9 µmol, 10 mol%). The Schlenk tube was evacuated and backfilled with argon three times and then, dry EtOAc (10 mL, 0.10 M), 1,1-diphenylethylene (**2a**, 181 mg, 1.00 mmol, 1.0 equiv), and acrylonitrile (**3a,** 106 mg, 2.00 mmol, 2.0 equiv) were added. The tube was sealed and irradiated at 405 nm for 12 h at r.t. (see picture below). After that, the NMR yield was determined using CH_2Br_2 as internal standard.

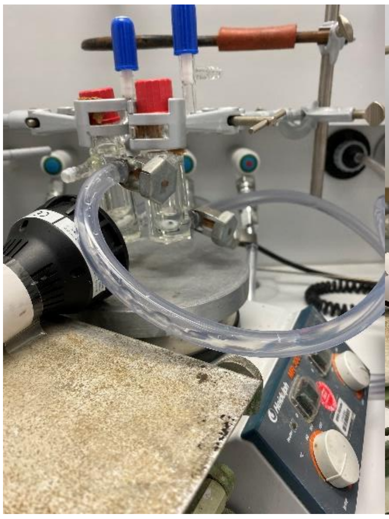
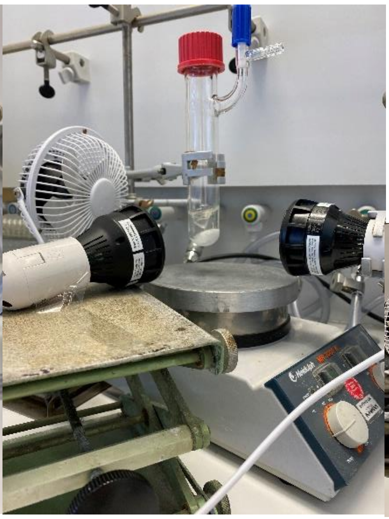
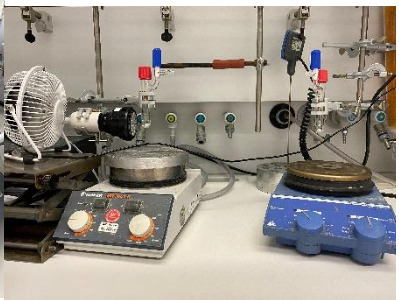

Fig. S3. Special experimental set-ups used for the low-temperature reaction (left), the large scale reaction (middle), and the high- and low-intensity reactions (right).

Table S2. Results of the sensitivity assessment.

Entry	Experiment	Notes	Relative difference in yield (%)
1	Standard	/	/
2	Low concentration	+100 µL EtOAc	-6
3	High concentration	-100 µL EtOAc	0
4	Low intensity	One lamp 32 cm away from reaction (see picture)	-7
5	High intensity	One lamp 2 cm away from reaction (see picture)	-20
6	High H_2O	+5 µL H_2O	-4
7	Low O_2	Freeze-pump-thaw 3x	+7
8	High O_2	Under air	-67
9	Low temperature	Water-cooled Schlenk tube (see picture); Yield is deviation from control reaction with water-cooled Schlenk tube filled with water but turned off flow	+5
10	High temperature	Standard photoreactor with fans turned off	-19
11	Large scale	Reaction on 1 mmol scale as described above and in the picture	-17

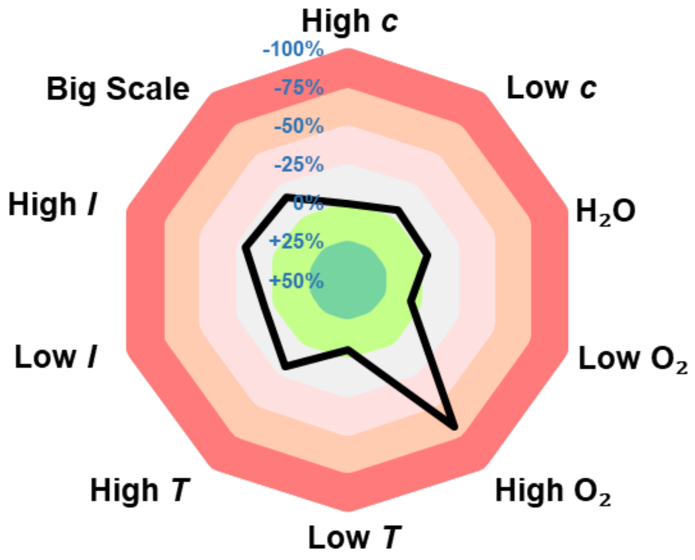

Fig. S4. Sensitivity screening radar diagram.

3.3. Additive-based robustness screening

The reaction's tolerance of functional groups and heterocycles was initially evaluated by conducting an additive-based robustness screening according to a simplified procedure from Glorius.[8]

Preparation of stock solution 3: A Schlenk tube was charged with a magnetic stirring bar and oxime ester **1a** (844 mg, 3.00 mmol, 1.5 equiv). The tube was evacuated and backfilled with argon three times. Then, dry EtOAc (20 mL, 0.10 M), 1,1-diphenylethylene (**2a**, 360 mg, 2.00 mmol, 1.0 equiv), and acrylonitrile (**3a,** 212 mg, 4.00 mmol, 2.0 equiv) were added. The mixture was stirred until a clear solution was obtained. The effect of oxime ester **1a**, 1,1-diphenylethylene (**2a**), and acrylonitrile (**3a**) on the volume of the stock solution was neglected in the following.

Reaction set-up: 16 Schlenk tubes were each charged with a magnetic stirring bar, thioxanthone (each 2.1 mg, 9.9 μmol, 10 mol%), and the additive (if solid; 0.10 mmol, 1.0 equiv). The Schlenk tubes were evacuated and backfilled with argon three times and then, the prepared **stock solution 3** (1.0 mL) was added to each Schlenk tube, followed by the additive (if liquid; 0.10 mmol, 1.0 equiv). The tubes were sealed and irradiated at 405 nm for 12 h at r.t. The additive recovery was then determined by GC-FID (one-point-calibration). The GC samples were prepared by adding mesitylene – as internal standard – to the rection mixture and then adding 40 μL of the respective reaction mixture to a short pad of silica – Celite® for the reaction with acetanilide – and eluting with EtOAc (2.0 mL). All GC samples were added back to the respective reaction mixture after measurement and the product yield was determined by ^{1}H NMR using CH_2Br_2 as internal standard.

Table S3. Results of the additive-based robustness screening.[a,b]

Entry	Additive	Yield Additive	Yield Product	
1	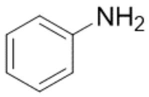	34	8	aniline
2	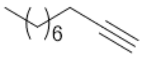	95	44	1-decyne

**3**	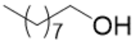	96	57	1-nonanol
**4**	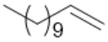	97	45	1-dodecene
**5**	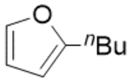	87	51	2-butylfuran
**6**	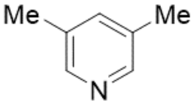	79	59	3,5-dimethylpyridine
**7**	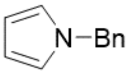	90	44	benzylpyrrole
**8**	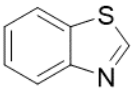	88	50	benzothiazol
**9**	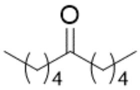	98	55	6-undecanone
**10**	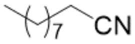	96	60	decanenitrile
**11**	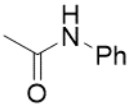	84	50	acetanilide
**12**	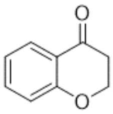	98	56	bromobenzene
**13**	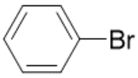	92	62	chromanone
**14**	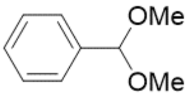	90	53	benzaldehyde dimethyl acetal
**15**	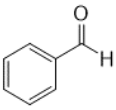	90	48	benzaldehyde
**16**	none	none	64	control
		**Average**	**Average**	
		88	49	

[a] Colorcoding for additive recovery: Green (>66%), yellow (33-66%), and red (<33%). [b] Colorcoding for product yield: Green (>42%), yellow (21-42%), and red (<21%).

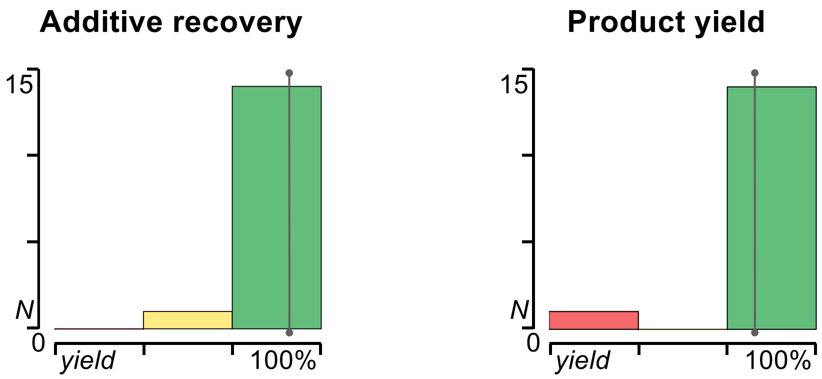

Fig. S5. Additive-based robustness screening results depicted as the typical bar charts.

3.4. TEMPO trapping experiment

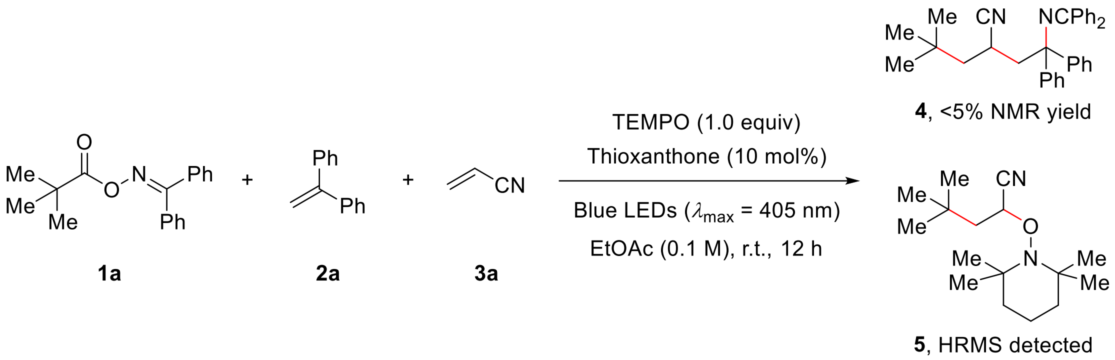

In an oven-dried 10 ml Schlenk tube equipped with a PTFE-coated rare-earth "extra power" oval stirring bar, oxime ester **1a** (42.2 mg, 0.15 mmol), thioxanthone (2.1 mg, 10 mol%), and 2,2,6,6-tetramethylpiperidine 1-oxyl (TEMPO; 15.7 mg, 0.1 mmol) were charged under air, then the vessel was evacuated and re-filled with argon for four times. Dry EtOAc (1.0 mL, 0.1 M), styrene **2a** (18.0 mg, 0.1 mmol), and Michael acceptor **3a** (10.6 mg, 0.2 mmol) were added under argon counter flow. The vessel was sealed with the screw cap, then irradiated at 405 nm using the described set-up for 12 hours. After irradiation, the reaction yield was determined by ^{1}H NMR spectroscopy using CH_2Br_2 as internal standard and the reaction was further analyzed by ESI-HRMS. HRMS (ESI) for **5**: m/z calculated for [$C_{16}H_{30}N_2ONa$] [$M+Na^+$]: 289.2250, found: 289.2247.

3.5. Two component reactions of 1a with 2a or 3a

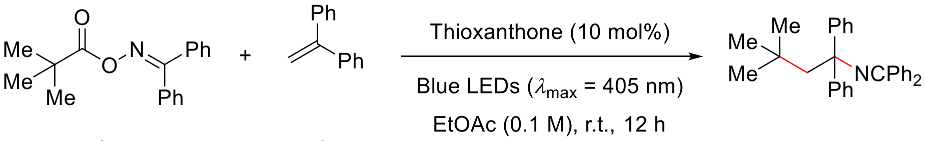

1a **2a** **6**, 68%

N-(3,3-Dimethyl-1,1-diphenylbutyl)-1,1-diphenylmethanimine (6)

In an oven-dried 10 ml Schlenk tube equipped with a PTFE-coated rare-earth "extra power" oval stirring bar, oxime ester **1a** (84.3 mg, 0.3 mmol) and thioxanthone (4.2 mg, 10 mol%) were charged under air, then the vessel was evacuated and re-filled with argon for four times. Dry EtOAc (2.0 mL, 0.1 M) and styrene **2a** (36.0 mg, 0.2 mmol) were added under argon counter flow. The vessel was sealed with the screw cap, then irradiated at 405 nm using the described set-up for 12 hours. After irradiation, the resulting homogenous solution was transferred to a 25 mL round bottom flask with aid of DCM (2 x 3 mL). NEt_3 (approx. 0.5 mL) and SiO_2 were added to this solution and the volatiles were removed under reduced pressure, affording a powder which was loaded on column. Purification by flash column chromatography on SiO_2 (pre-basified with NEt_3) with pentane as eluent afforded the title compound **6** (57 mg, 68%) as a colorless oil. ^{1}H NMR (400 MHz, $CDCl_3$): δ = 0.76 (s, 9H), 2.50 (s, 2H), 6.43-6.58 (m, 2H), 6.71-7.22 (m, 11H), 7.30-7.61 (m, 5H), 7.67-7.79 (m, 2H) ppm. ^{13}C NMR (101 MHz, $CDCl_3$): δ = 32.1, 32.3, 52.3, 68.8, 125.6, 126.6, 127.2, 127.6, 127.7, 128.1, 128.5, 129.7, 139.0, 142.2, 151.3, 164.8 ppm. HRMS (ESI): m/z calculated for [$C_{31}H_{31}NNa$] [$M+Na^+$]: 440.2349, found: 440.2347.

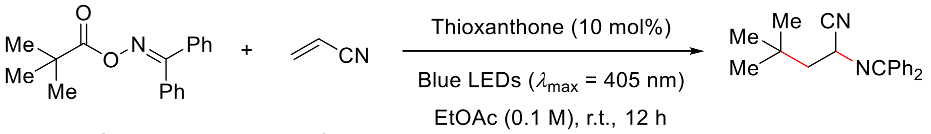

1a **3a** **7**, 72%

2-((Diphenylmethylene)amino)-4,4-dimethylpentanenitrile (7)

In an oven-dried 10 ml Schlenk tube equipped with a PTFE-coated rare-earth "extra power" oval stirring bar, oxime ester **1a** (84.3 mg, 0.3 mmol) and thioxanthone (4.2 mg, 10 mol%) were charged under air, then the vessel was evacuated and re-filled with argon for four times. Dry EtOAc (2.0 mL, 0.1 M) and Michael acceptor **3a** (10.6 mg, 0.20 mmol) were added under argon counter flow. The vessel was sealed with the screw cap, then irradiated at 405 nm using the described set-up for 12 hours. After irradiation, the resulting homogenous solution was transferred to a 25 mL round bottom flask with aid of DCM (2 x 3 mL). NEt_3 (approx. 0.5 mL) and SiO_2 were added to this solution

and the volatiles were removed under reduced pressure, affording a powder which was loaded on column. Purification by flash column chromatography on SiO_2 (pre-basified with NEt_3) with pentane/EtOAc (400:1 to 200:1, v/v) as eluent afforded the title compound **7** (42 mg, 72%) as a yellow oil. ^{1}H NMR (400 MHz, $CDCl_3$): δ = 0.85 (s, 9H), 1.82 (dd, J = 14.1, 6.1 Hz, 1H), 2.07 (dd, J = 14.1, 7.0 Hz, 1H), 4.29 (dd, J = 7.1, 6.1 Hz, 1H), 7.22-7.26 (m, 2H), 7.34-7.39 (m, 2H), 7.41-7.47 (m, 1H), 7.48-7.58 (m, 3H), 7.61-7.70 (m, 2H) ppm. ^{13}C NMR (101 MHz, $CDCl_3$): δ = 29.7, 30.7, 48.4, 50.5, 120.8, 127.5, 128.4, 129.1, 129.1, 129.5, 131.2, 135.5, 138.7, 172.2 ppm. HRMS (ESI): m/z calculated for [$C_{20}H_{22}N_2Na$] [$M+Na^+$]: 313.1675, found: 313.1672.

3.6. Detection of side products

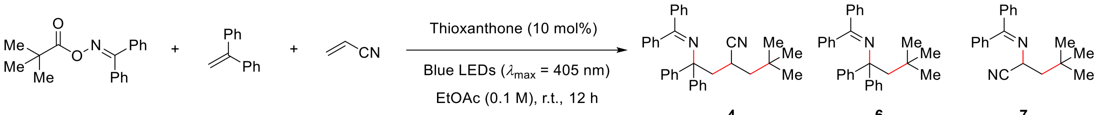

1a 2a 3a 4 62% NMR yield 6 < 5% NMR yield (traces) 7 23% NMR yield

In an oven-dried 10 ml Schlenk tube equipped with a PTFE-coated rare-earth “extra power” oval stirring bar, oxime ester **1a** (84.3 mg, 0.3 mmol) and thioxanthone (4.2 mg, 10 mol%) were charged under air, then the vessel was evacuated and re-filled with argon for four times. Dry EtOAc (2.0 mL, 0.1 M), styrene **2a** (36.0 mg, 0.2 mmol), and Michael acceptor **3a** (21.2 mg, 0.4 mmol) were added under argon counter flow. The vessel was sealed with the screw cap and then irradiated at 405 nm using the described set-up for 12 hours. After irradiation, the NMR yields for **4** (62%), **6** (< 5%), and **7** (23% with respect to **1a**) were determined using CH_2Br_2 as internal standard.

3.7. Direct excitation experiment

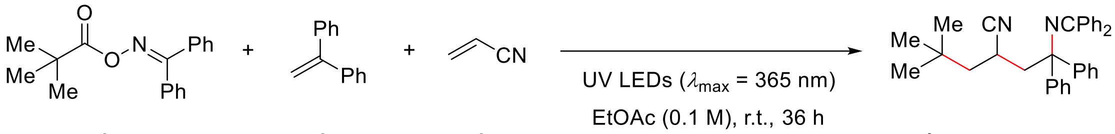

1a 2a 3a **4**, 38% NMR yield

In an oven-dried 10 ml Schlenk tube equipped with a PTFE-coated rare-earth “extra power” oval stirring bar, oxime ester **1a** (42.2 mg, 0.15 mmol) was charged under air, then the vessel was evacuated and re-filled with argon for four times. Dry EtOAc (1.0 mL, 0.1 M), styrene **2a** (18.0 mg, 0.1 mmol), and Michael acceptor **3a** (10.6 mg, 0.2 mmol) were added under argon counter flow. The vessel was sealed with the screw

cap, then irradiated at 365 nm with two 30 W LEDs (EvoluChem™, HCK1012-01-006) using the described set-up for 36 hours. After irradiation, the reaction yield (38%) was determined by ^{1}H NMR spectroscopy using CH_2Br_2 as internal standard.

3.8. Quantum yield measurement

Measurement of the photon flux

The photon flux was determined by ferrioxalate actinometry similar to a procedure by Yoon.[9] A 3 W violet LED (λ_{max} = 395 nm) was used for the quantum yield measurement instead of the standard 18 W blue LEDs (λ_{max} = 405 nm).

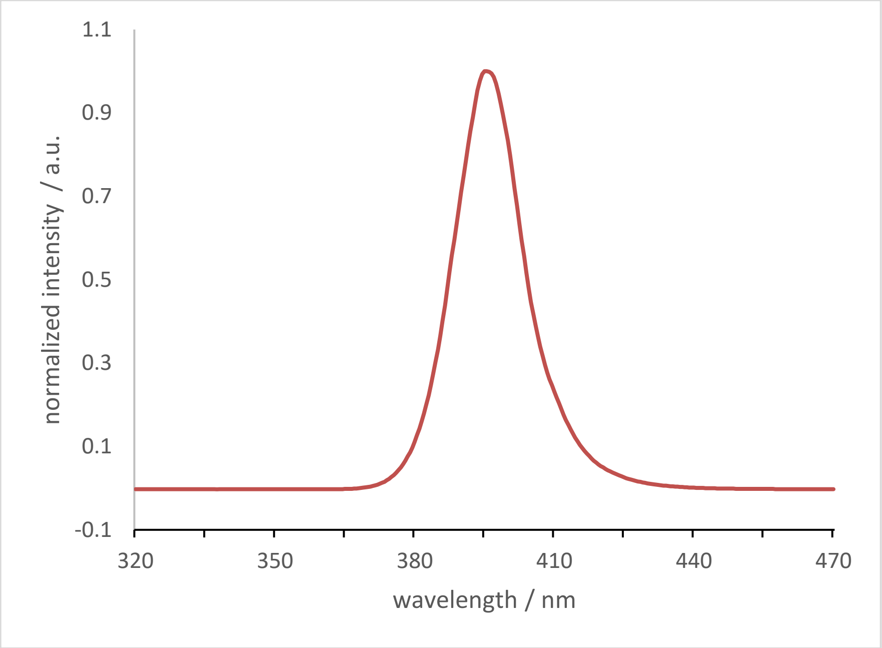

Fig. S6. Emission spectrum of the 3 W violet LED (λ_{max} = 395 nm) which was used for the quantum yield measurement.

A solution (10 mL, 0.15 M) of potassium ferrioxalate hydrate (737 mg, 1.50 mmol) in aq. H_2SO_4 (50 mM) and a solution (20 mL, 5.0 mM) of 1,10-phenanthroline monohydrate (20 mg, 0.10 mmol) and sodium acetate (4.50 g) in aq. H_2SO_4 (500 mM) were prepared and kept in the dark. All following steps were conducted in the dark as well.

Six Schlenk tubes were charged with the prepared ferrioxalate solution (1.0 mL) and three of these tubes were successively irradiated with a violet LED for 60 s at a distance of 5 cm, while three tubes were left in the dark. Then, to all six Schlenk tubes was added the prepared phenanthroline solution (175 µL each) and the reaction mixtures were

stirred for 60 mins. For all six solutions, the absorbance at 510 nm was measured and the difference between the average absorbance of the three irradiated samples and the average absorbance of the three control samples was determined ($\Delta A_{510\text{ nm}}$ = 2.672). The formed amount of Fe(II) was calculated based on the Lambert–Beer law (equation 1) with V = 1.175 mL, l = 1.0 cm, and ε = 11100 L·mol^{-1}·cm^{-1}.[10]

$$n_{Fe(II)} = \frac{V \cdot \Delta A_{510\text{ nm}}}{l \cdot \varepsilon} \quad (1)$$

The fraction of light which was absorbed by the actinometer at λ = 395 nm (f) was determined with equation 2 with the absorbance of the ferrioxalate stock solution at λ = 395 nm being $A_{395\text{ nm}} > 3$ ($f > 0.999$).

$$f = 1 - 10^{-A_{395\text{ nm}}} \quad (2)$$

The photonflux ϕ_q was finally determined using equation 3 with ϕ_F = 1.13 (at λ = 392 nm) and t = 60 s, giving ϕ_q = 4.172·10^{-9} mol·s^{-1}.[10,11]

$$\phi_\mathrm{q} = \frac{n_{Fe(II)}}{\phi_\mathrm{F} \cdot t \cdot f} \quad (3)$$

Reaction quantum yield

In an oven-dried 10 ml Schlenk tube equipped with a PTFE-coated rare-earth “extra power” oval stirring bar, oxime ester **1a** (42.2 mg, 0.15 mmol) and thioxanthone (2.1 mg, 10 mol%) were charged under air, then the vessel was evacuated and re-filled with argon for four times. Dry EtOAc (1.0 mL, 0.1 M), styrene **2a** (18.0 mg, 0.1 mmol), and Michael acceptor **3a** (10.6 mg, 0.2 mmol) were added under argon counter flow. The Schlenk tube was sealed and irradiated at 395 nm at ambient temperature for 30 minutes using the same setup as for the photon flux determination. Then, the NMR yield was determined (3%) using CH_2Br_2 as internal standard. The same procedure was repeated two more times giving the same yield.

The reaction’s quantum yield was determined using equation 4 with the determined photon flux ϕ_q, the irradiation time t = 1800 s, and the fraction of light absorbed

(f_R > 0.999; determined according to equation 2 with $A_{395\ \mathrm{nm}}$ > 3) by the reaction mixture.

$$\phi = \frac{n_{\text{product}}}{\phi_q \cdot t \cdot f_R} \tag{4}$$

The determined quantum yield was ϕ = 0.40.

3.9. Determination of the initial reaction rate of the formation of 6 and 7

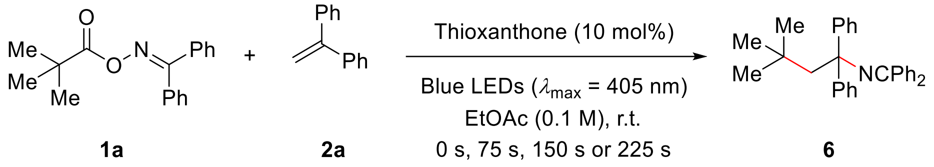

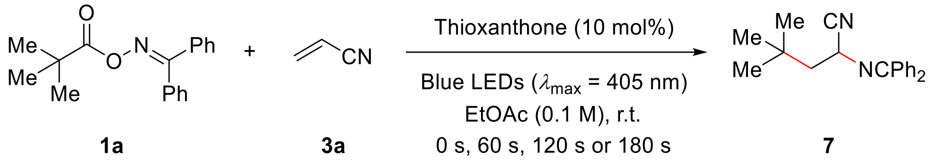

Preparation of stock solution 4: A Schlenk tube was charged with a magnetic stirring bar and **1a** (338 mg, 1.2 mmol). The vessel was then evacuated and re-filled with argon for four times and dry EtOAc (8.0 mL, 0.1 M) followed by **2a** (144 mg, 0.8 mmol) were added under argon counterflow. The solution was vigorously stirred for 10 minutues and kept under argon. The effect of oxime ester **1a** and 1,1-diphenylethylene (**2a**) on the volume of the stock solution was neglected in the following.

Preparation of stock solution 5: A Schlenk tube was charged with a magnetic stirring bar and **1a** (211 mg, 0.75 mmol). The vessel was then evacuated and re-filled with argon for four times and dry EtOAc (5.0 mL, 0.1 M) followed by **3a** (26.5 mg, 0.5 mmol) were added under argon counterflow. The solution was vigorously stirred for 10 minutues and kept under argon. The effect of oxime ester **1a** and acrylonitrile (**3a**) on the volume of the stock solution was neglected in the following.

Reaction set-up: 8 Schlenk tubes were each charged with a stirring bar and thioxanthone (2.1 mg, 10 mol%) under air. The Schlenk tubes were than evacuated and re-filled with argon for four times. To each Schlenk tube was then added either **stock solution 4** (1.0 mL) or **stock solution 5** (1.0 mL) under argon counterflow. The tubes were sealed and irradiated at 405 nm in the standard photosetup for the indicated

reaction time. Care was taken to place each reaction vessel in the same position within the photoreactor to ensure consistent light intensity. Then, the NMR yield was determined using CH_2Br_2 as internal standard.

Results: The obtained NMR yields after the respective reaction time are compiled in Table S4 and visualized in Fig. S7.

Table S4. Yields of **6** and **7** in the respective two-component reaction after the indicated reaction time.

Entry	Alkene/Product	Reaction time (s)	NMR Yield (%)
1	**2a/6**	0	0
2	**2a/6**	75	2
3	**2a/6**	150	5
4	**2a/6**	225	8
5	**3a/7**	0	0
6	**3a/7**	60	5
7	**3a/7**	120	10
8	**3a/7**	180	15

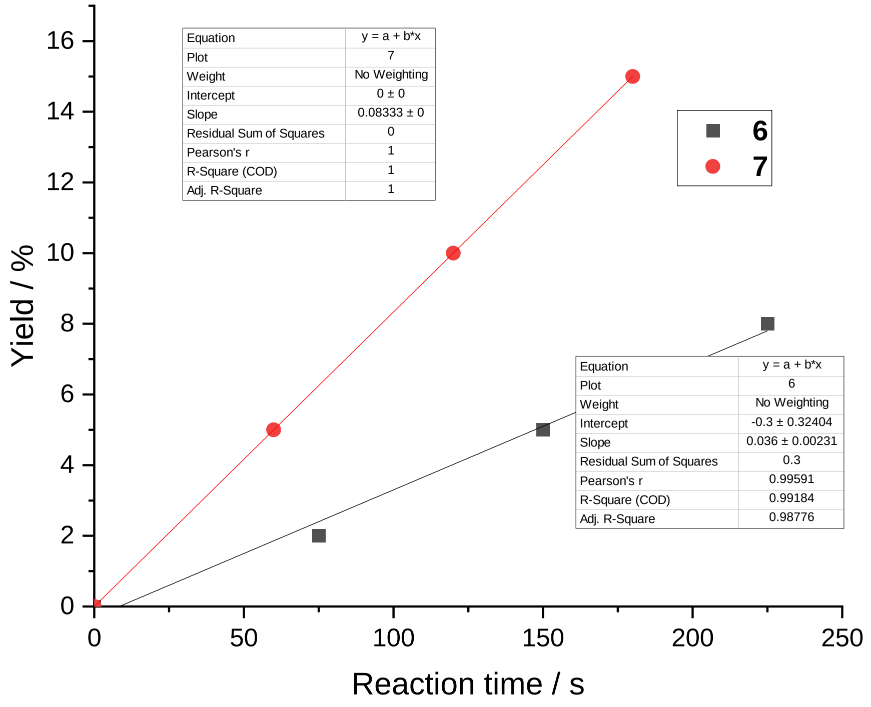

Fig. S7. Visualized initial course of the formation of **6** (black) and **7** (red) in the respective two-component reactions. NMR yields are used.

3.10. Mechanistic analysis for 1,4-carboimination of 1a, 2a, and 3a

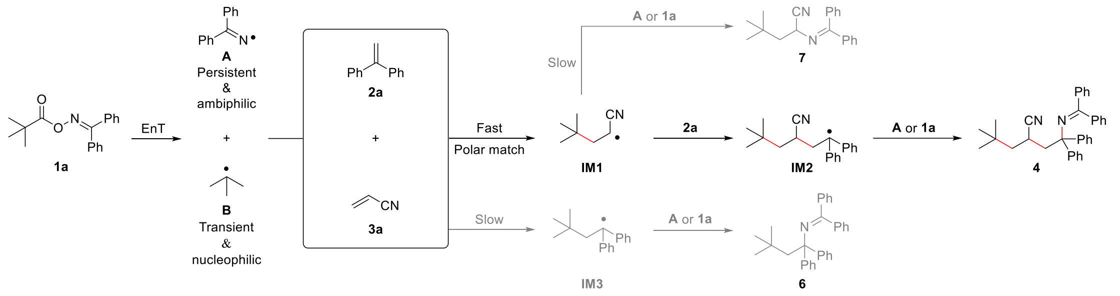

Based on previous studies and the carried out mechanistic experiments,[3,12] a mechanistic analysis of the selective 1,4-carboimination of **1a**, **2a**, and **3a** was developed. The reaction starts with an energy transfer-enabled homolysis of **1a** to provide a persistent N-centered iminyl radical **A** and a transient C-centered alkyl radical **B**, which are of ambiphilic and nucleophilic properties, respectively. Then, steered by polar and persistent radical effects (PRE),[13] the nucleophilic C-centered alkyl radical **B** preferentially adds to electron poor olefin **3a** to form an α-cyano-containing C-centered alkyl radical with reduced nucleophilicity (**IM1**). Subsequently, addition of **IM1** to **2a** gives another benzyl stabilized C-centered alkyl radical (**IM2**), which is further trapped by the persistent N-centered iminyl radical **A** to furnish the desired 1,4-carboimination product **4**. Alternatively, radical addition of **IM2** onto **1a** and subsequent fragmentation can form **4** in a radical chain mechanism.[12b,c] The competing 1,2-carboiminations of **2a** or **3a** were observed as minor side reactions.

3.11. General procedure for the 1,4-carboimination

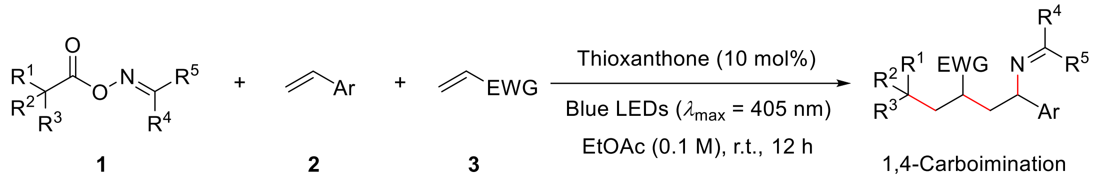

In an oven-dried 10 ml Schlenk tube equipped with a PTFE-coated rare-earth “extra power” oval stirring bar, oxime ester **1** (0.3 mmol, 1.5 equiv., unless otherwise stated), styrene derivatives (0.2 mmol, 1.0 equiv., if solid), Michael acceptors (0.4 mmol, 2.0 equiv., if solid), and thioxanthone photosensitizer (4.2 mg, 10 mol%) were charged under air, then the vessel was evacuated and re-filled with argon for four times. Dry EtOAc (2.0 mL, 0.1 M), appropriate styrene derivatives (0.2 mmol, 1.0 equiv., if liquid),

and Michael acceptors (0.4 mmol, 2.0 equiv., if liquid) were added under argon counter flow. The vessel was sealed with the screw cap, then irradiated at 405 nm using the described set-up for 12 hours, unless otherwise stated. After irradiation, the resulting homogenous solution was transferred to a 25 mL round bottom flask with aid of DCM (2 x 3 mL). NEt_3 (approx. 0.5 mL) and SiO_2 were added to this solution and the volatiles were removed under reduced pressure, affording a powder which was loaded on column. Purification by flash column chromatography on SiO_2, pre-basified with NEt_3 using pentane: EtOAc mixtures afforded the corresponding 1,4-carboimination products.

Note: *NEt_3 was used as the product can be partially hydrolyzed by acidic silica, causing a slight decrease in the yield (approx. 5-10%). The amount of Et_3N added to the eluent was approximately 1 ml for 300 ml of solvent. When Et_3N was used for TLC analysis, one Pasteur pipette drop was used for 10 ml of eluent. "Pre-basified" silica refers to silica gel, which have been soaked with 2-3% triethylamine in pentane prior to use.*

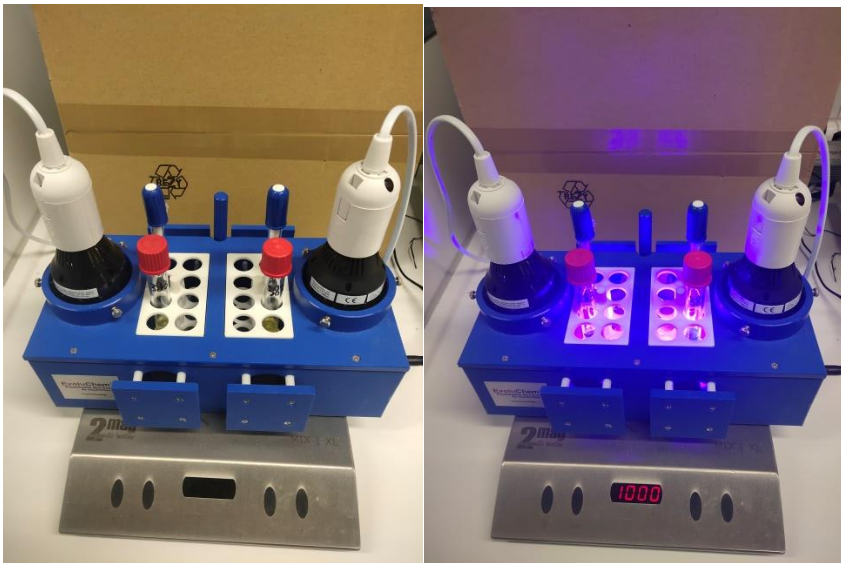

Fig. S8. Experimental set-up featuring two 18W Blue LEDs (*405 nm*).

4. Experimental data for the described substances

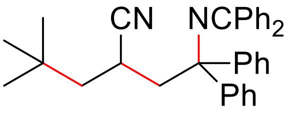

4

2-(2-((Diphenylmethylene)amino)-2,2-diphenylethyl)-4,4-dimethylpentanenitrile (4)

Following the general procedure, benzophenone *O*-pivaloyl oxime **1a** (84.3 mg, 0.3 mmol), ethene-1,1-diyldibenzene **2a** (36.0 mg, 0.2 mmol), and acrylonitrile **3a** (21.2 mg, 0.4 mmol) were used. Purification by column chromatography using pre-basified silica with pentane/EtOAc (400:1 to 200:1, v/v) as eluent afforded **4** as colorless oil (56 mg, 60% yield). ^{1}H NMR (400 MHz, $CDCl_3$): δ = 0.76 (s, 9H), 1.12-1.16 (m, 1H), 1.63-1.70 (m, 1H), 2.34-2.38 (m, 1H), 2.58-2.64 (m, 1H), 2.97-3.02 (m, 1H), 6.58-6.60 (m, 2H), 7.00-7.19 (m, 9H), 7.23-7.27 (m, 2H), 7.33-7.42 (m, 3H), 7.47-7.53 (m, 2H), 7.71-7.74 (m, 2H) ppm. ^{13}C NMR (101 MHz, $CDCl_3$): δ = 22.4, 29.3, 31.0, 46.1, 48.4, 68.7, 124.5, 126.4, 126.5, 126.9, 127.2, 127.4, 128.00, 128.04, 128.12, 128.15, 128.7, 130.2, 130.2, 138.6, 141.6, 148.1, 148.4, 168.8 ppm. HRMS (ESI): m/z calculated for [$C_{34}H_{34}N_2Na$] [$M+Na^+$]: 493.2620, found: 493.2632.

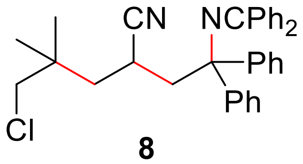

8

5-Chloro-2-(2-((diphenylmethylene)amino)-2,2-diphenylethyl)-4,4-dimethylpentanenitrile (8)

Following the general procedure, benzophenone *O*-(3-chloro-2,2-dimethylpropanoyl) oxime **1b** (94.5 mg, 0.3 mmol), ethene-1,1-diyldibenzene **2a** (36.0 mg, 0.2 mmol), and acrylonitrile **3a** (21.2 mg, 0.4 mmol) were used. Purification by column chromatography using pre-basified silica with pentane/EtOAc (400:1 to 200:1, v/v) as eluent afforded **8** as colorless oil (67 mg, 67% yield). ^{1}H NMR (400 MHz, $CDCl_3$): δ = 0.80 (s, 3H), 0.92 (s, 3H), 1.32-1.36 (m, 1H), 1.73-1.79 (m, 1H), 2.37-2.41 (m, 1H), 2.59-2.66 (m, 1H), 2.98-3.03 (m, 1H), 3.07-3.10 (m, 1H), 3.19-3.22 (m, 1H), 6.57-6.59 (m, 2H), 7.00-7.20 (m, 9H), 7.24-7.28 (m, 2H), 7.33-7.42 (m, 3H), 7.48-7.50 (m, 2H), 7.70-7.73 (m, 2H) ppm. ^{13}C NMR (101 MHz, $CDCl_3$): δ = 22.0, 24.8, 25.2, 35.7, 43.4, 46.0, 55.0, 68.6, 123.8, 126.5, 126.6, 127.0, 127.2, 127.5, 128.04, 128.06, 128.08, 128.2, 128.8, 130.3, 138.6, 141.6, 147.8, 148.2, 169.0 ppm. HRMS (ESI): m/z calculated for [$C_{34}H_{33}ClN_2Na$] [$M+Na^+$]: 527.2230, found: 527.2226.

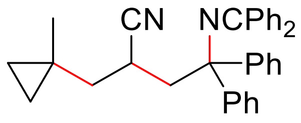

9

4-((Diphenylmethylene)amino)-2-((1-methylcyclopropyl)methyl)-4,4-diphenylbutanenitrile (9)

Following the general procedure, benzophenone *O*-(1-methylcyclopropanecarbonyl) oxime **1c** (83.7 mg, 0.3 mmol), ethene-1,1-diyldibenzene **2a** (36.0 mg, 0.2 mmol), and acrylonitrile **3a** (21.2 mg, 0.4 mmol) were used. Purification by column chromatography using pre-basified silica with pentane/EtOAc (500:1 to 300:1, v/v) as eluent afforded **9** as colorless oil (47 mg, 50% yield). ^{1}H NMR (400 MHz, $CDCl_3$): δ = 0.06-0.21 (m, 2H), 0.27 (dt, *J* = 9.8, 5.1 Hz, 1H), 0.42 (dt, *J* = 9.6, 5.0 Hz, 1H), 0.75 (s, 3H), 1.24-1.43 (m, 2H), 2.40 (dd, *J* = 13.4, 3.5 Hz, 1H), 2.71-2.81 (m, 1H), 2.86 (dd, *J* = 13.4, 8.0 Hz, 1H), 6.55 (s, 2H), 6.97-7.19 (m, 9H), 7.21-7.26 (m, 2H), 7.31-7.41 (m, 3H), 7.50 (d, *J* = 18.6 Hz, 2H), 7.67-7.75 (m, 2H) ppm. ^{13}C NMR (101 MHz, $CDCl_3$): δ = 12.8, 13.4, 13.8, 22.0, 24.6, 43.7, 44.0, 68.6, 123.5, 126.47, 126.52, 126.9, 127.2, 127.5, 127.6, 128.02, 128.07, 128.11, 128.2, 128.38, 128.44, 128.7, 129.1, 130.3, 138.6, 141.6, 148.0, 148.4, 168.8 ppm. HRMS (ESI): m/z calculated for [$C_{34}H_{32}N_2Na$] [$M+Na^+$]: 491.2463, found: 491.2453.

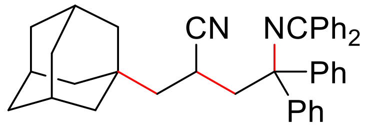

10

2-((3*r*,5*r*,7*r*)-Adamantan-1-ylmethyl)-4-((diphenylmethylene)amino)-4,4-diphenylbutanenitrile (10)

Following the general procedure, benzophenone *O*-((3*r*,5*r*,7*r*)-adamantane-1-carbonyl) oxime **1d** (107.7 mg, 0.3 mmol), ethene-1,1-diyldibenzene **2a** (36.0 mg, 0.2 mmol), and acrylonitrile **3a** (21.2 mg, 0.4 mmol) were used. Purification by column chromatography using pre-basified silica with pentane/EtOAc (400:1 to 200:1, v/v) as eluent afforded **10** as a white solid (55 mg, 50% yield). ^{1}H NMR (400 MHz, $CDCl_3$): δ = 1.01-1.05 (m, 1H), 1.22-1.36 (m, 6H), 1.43-1.71 (m, 7H), 1.84-1.91 (m, 3H), 2.31-

2.36 (m, 1H), 2.64-2.70 (m, 1H), 2.95-3.00 (m, 1H), 6.58-6.60 (m, 2H), 7.00-7.20 (m, 9H), 7.23-7.27 (m, 2H), 7.33-7.42 (m, 3H), 7.45-7.55 (m, 2H), 7.72-7.74 (m, 2H) ppm. ^{13}C NMR (101 MHz, $CDCl_3$): δ = 20.4, 28.49, 28.52, 32.8, 36.8, 42.0, 46.1, 49.2, 68.7, 124.7, 126.41, 126.44, 126.9, 127.2, 127.4, 127.97, 128.01, 128.1, 128.2, 128.7, 130.2, 138.6, 141.6, 148.1, 148.4, 168.7 ppm. HRMS (ESI): m/z calculated for [$C_{40}H_{40}N_2Na$] [M+Na$^+$]: 571.3089, found: 571.3082.

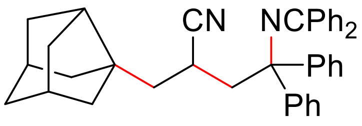

11

4-((Diphenylmethylene)amino)-2-(((2*R*,3*ar*,5*S*,6*as*)-octahydro-2,5-methanopentalen-3*a*-yl)methyl)-4,4-diphenylbutanenitrile (11)

Following the general procedure, benzophenone *O*-((2*R*,3*as*,5*S*,6*as*)-octahydro-2,5-methanopentalene-3a-carbonyl) oxime **1e** (103.5 mg, 0.3 mmol), ethene-1,1-diyldibenzene **2a** (36.0 mg, 0.2 mmol), and acrylonitrile **3a** (21.2 mg, 0.4 mmol) were used. Purification by column chromatography using pre-basified silica with pentane/EtOAc (400:1 to 200:1, v/v) as eluent afforded **11** as a white solid (62 mg, 58% yield). ^{1}H NMR (400 MHz, $CDCl_3$): δ = 0.84-0.91 (m, 1H), 1.17-1.22 (m, 1H), 1.41-1.62 (m, 9H), 1.78-1.81 (m, 1H), 1.84-1.90 (m, 1H), 2.09-2.12 (m, 2H), 2.37-2.41 (m, 1H), 2.59-2.65 (m, 1H), 2.92-2.97 (m, 1H), 6.57-6.58 (m, 2H), 6.99-7.17 (m, 9H), 7.21-7.28 (m, 2H), 7.32-7.41 (m, 3H), 7.45-7.49 (m, 2H), 7.69-7.71 (m, 2H) ppm. ^{13}C NMR (101 MHz, $CDCl_3$): δ = 23.8, 35.0, 37.5, 37.9, 43.6, 43.7, 44.3, 44.7, 45.4, 48.2, 48.3, 48.4, 68.7, 124.3, 126.5, 126.9, 127.3, 127.5, 128.03, 128.06, 128.13, 128.15, 128.7, 128.8, 129.5, 130.2, 138.6, 141.6, 148.2, 148.4, 168.7 ppm. HRMS (ESI): m/z calculated for [$C_{39}H_{38}N_2Na$] [M+Na$^+$]: 557.2933, found: 557.2926.

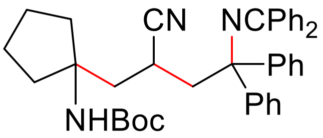

12

***tert*-Butyl (1-(2-cyano-4-((diphenylmethylene)amino)-4,4-diphenylbutyl)cyclopentyl)carbamate (12)**

Following the general procedure, *tert*-butyl (1-((((diphenylmethylene)amino)oxy)carbonyl)cyclopentyl)carbamate **1f** (122.4 mg, 0.3 mmol), ethene-1,1-diyldibenzene **2a** (36.0 mg, 0.2 mmol), and acrylonitrile **3a** (21.2 mg, 0.4 mmol) were used. Purification by column chromatography using pre-basified silica with pentane/EtOAc (40:1 to 30:1, v/v) as eluent afforded **12** as a white solid (62 mg, 63% yield). Combined NMR data for both the rotamers are reported here. ^{1}H NMR (400 MHz, $CDCl_3$): δ = 1.22-1.85 (m, 18H), 2.12-2.18 (m, 1H), 2.32-2.44 (m, 1H), 2.56-2.69 (m, 1H), 2.88-2.93 (m, 1H), 4.24 (s, 1H), 6.57 (m, 2H), 6.99-7.17 (m, 9H), 7.21-7.25 (m, 2H), 7.31-7.39 (m, 3H), 7.42-7.54 (m, 2H), 7.62-7.72 (m, 2H) ppm. ^{13}C NMR (101 MHz, $CDCl_3$): δ = 23.0, 23.1, 23.4, 23.5, 28.46, 28.56, 38.2, 38.6, 41.2, 44.5, 50.6, 62.4, 62.9, 68.4, 79.2, 120.2, 123.5, 126.45, 126.49, 127.0, 127.3, 127.47, 127.52, 128.00, 128.03, 128.1, 128.2, 128.4, 128.5, 128.8, 129.2, 129.5, 130.2, 131.3, 135.4, 138.6, 141.6, 148.3, 148.6, 154.5, 168.9 ppm. HRMS (ESI): m/z calculated for [$C_{40}H_{43}N_3NaO_2$] [M+Na$^+$]: 620.3253, found: 620.3256.

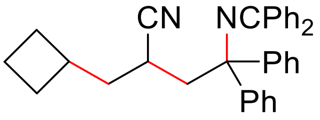

13

2-(Cyclobutylmethyl)-4-((diphenylmethylene)amino)-4,4-diphenylbutanenitrile (13)

Following the general procedure, benzophenone *O*-cyclobutanecarbonyl oxime **1g** (83.8 mg, 0.3 mmol), ethene-1,1-diyldibenzene **2a** (36.0 mg, 0.2 mmol), and acrylonitrile **3a** (21.2 mg, 0.4 mmol) were used. Purification by column chromatography using pre-basified silica with pentane/EtOAc (400:1 to 200:1, v/v) as eluent afforded **13** as a white solid (52 mg, 56% yield). ^{1}H NMR (400 MHz, $CDCl_3$): δ = 1.24-1.52 (m, 3H), 1.55-2.02 (m, 5H), 2.24-2.40 (m, 2H), 2.49 (m, 1H), 2.86 (dd, J = 13.5, 8.4 Hz, 1H), 6.56 (d, J = 5.4 Hz, 2H), 7.00-7.20 (m, 9H), 7.23-7.27 (m, 3H), 7.32-7.42 (m, 3H), 7.45-7.48 (m, 2H), 7.67-7.76 (m, 2H) ppm. ^{13}C NMR (101 MHz, $CDCl_3$): δ = 18.5, 24.9, 27.9, 28.3, 33.6, 41.1, 43.6, 68.5, 123.0, 126.5, 126.5, 127.0, 127.3,

127.4, 128.0, 128.0, 128.1, 128.2, 128.7, 130.3, 138.6, 141.6, 148.0, 148.4, 168.7 ppm. HRMS (ESI): m/z calculated for [$C_{34}H_{32}N_2Na$] [M+Na^+]: 491.2458, found: 491.2459.

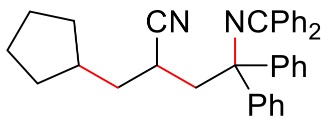

14

2-(Cyclopentylmethyl)-4-((diphenylmethylene)amino)-4,4-diphenylbutanenitrile (14)

Following the general procedure, benzophenone *O*-cyclopentanecarbonyl oxime **1h** (88.0 mg, 0.3 mmol), ethene-1,1-diyldibenzene **2a** (36.0 mg, 0.2 mmol), and acrylonitrile **3a** (21.2 mg, 0.4 mmol) were used. Purification by column chromatography using pre-basified silica with pentane/EtOAc (400:1 to 200:1, v/v) as eluent afforded **14** as a white solid (56 mg, 58% yield). ^{1}H NMR (400 MHz, $CDCl_3$): δ = 0.67-0.98 (m, 2H), 1.20 (m, 1H), 1.37-1.54 (m, 5H), 1.56-1.67 (m, 2H), 1.74-1.90 (m, 1H), 2.36 (dd, *J* = 13.6, 3.6 Hz, 1H), 2.60 (m, 1H), 2.89 (dd, *J* = 13.6, 8.0 Hz, 1H), 6.56 (d, *J* = 5.3 Hz, 2H), 6.99-7.19 (m, 9H), 7.22-7.26 (m, 2H), 7.32-7.42 (m, 3H), 7.45-7.49 (m, 2H), 7.67-7.75 (m, 2H) ppm. ^{13}C NMR (101 MHz, $CDCl_3$): δ = 25.0, 25.0, 26.0, 31.8, 32.7, 37.7, 40.6, 44.0, 68.5, 123.3, 126.5, 126.5, 126.9, 127.2, 127.5, 128.0, 128.0, 128.1, 128.1, 128.7, 130.3, 138.6, 141.6, 148.0, 148.4, 168.7 ppm. HRMS (ESI): m/z calculated for [$C_{35}H_{34}N_2Na$] [M+Na^+]: 505.2614, found: 505.2616.

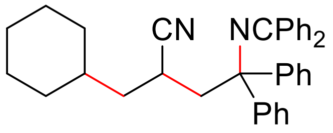

15

2-(Cyclohexylmethyl)-4-((diphenylmethylene)amino)-4,4-diphenylbutanenitrile (15)

Following the general procedure, benzophenone *O*-cyclohexanecarbonyl oxime **1i** (92.2 mg, 0.3 mmol), ethene-1,1-diyldibenzene **2a** (36.0 mg, 0.2 mmol), and acrylonitrile **3a** (21.2 mg, 0.4 mmol) were used. Purification by column chromatography using pre-basified silica with pentane/EtOAc (400:1 to 200:1, v/v) as eluent afforded **15** as a white solid (55 mg, 56% yield). ^{1}H NMR (400 MHz, $CDCl_3$): δ

= 0.53-0.76 (m, 2H), 0.96-1.22 (m, 4H), 1.23-1.36 (m, 2H), 1.39-1.66 (m, 5H), 2.32 (dd, *J* = 13.6, 3.3 Hz, 1H), 2.69 (m, 1H), 2.88 (dd, *J* = 13.5, 8.2 Hz, 1H), 6.57 (d, *J* = 7.4 Hz, 2H), 6.99-7.19 (m, 9H), 7.22-7.26 (m, 2H), 7.32-7.41 (m, 3H), 7.45-7.47 (m, 2H) 7.67-7.75 (m, 2H) ppm. ^{13}C NMR (101 MHz, $CDCl_3$): δ = 23.9, 26.0, 26.1, 26.4, 32.2, 33.3, 35.0, 41.9, 44.0, 68.6, 123.3, 126.5, 126.9, 127.2, 127.5, 128.0, 128.0, 128.1, 128.1, 128.2, 128.7, 130.3, 138.6, 141.6, 148.0, 148.5, 168.7 ppm. HRMS (ESI): m/z calculated for [$C_{36}H_{36}N_2Na$] [$M+Na^+$]: 519.2771, found: 519.2772.

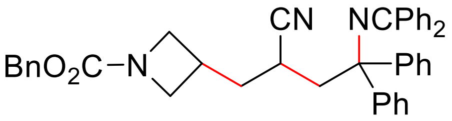

16

Benzyl 3-(2-cyano-4-((diphenylmethylene)amino)-4,4-diphenylbutyl)azetidine-1-carboxylate (16)

Following the general procedure, benzyl 3-((((diphenylmethylene)amino)oxy)carbonyl)azetidine-1-carboxylate **1j** (124.2 mg, 0.3 mmol), ethene-1,1-diyldibenzene **2a** (36.0 mg, 0.2 mmol), and acrylonitrile **3a** (21.2 mg, 0.4 mmol) were used. Purification by column chromatography using pre-basified silica with pentane/EtOAc (20:1 to 10:1, v/v) as eluent afforded **16** as a white solid (75 mg, 62% yield). ^{1}H NMR (400 MHz, $CDCl_3$): δ = 1.55-1.83 (m, 2H), 1.94-2.62 (m, 3H), 2.85-2.95 (m, 1H), 3.26-3.70 (m, 2H), 3.94-4.26 (m, 2H), 5.08-5.09 (m, 2H), 6.53-6.55 (m, 2H), 6.99-7.24 (m, 10H), 7.30-7.54 (m, 11H), 7.62-7.70 (m, 2H) ppm. ^{13}C NMR (101 MHz, $CDCl_3$): δ = 25.1, 27.3, 38.5, 39.1, 43.9, 66.7, 68.5, 122.1, 126.6, 126.8, 127.07, 127.13, 127.5, 127.9, 128.08, 128.10, 128.12, 128.17, 128.3, 128.6, 128.7, 129.1, 129.3, 130.4, 136.8, 138.4, 141.4, 147.5, 147.9, 156.3, 169.1 ppm. HRMS (ESI): m/z calculated for [$C_{41}H_{37}N_3NaO_2$] [$M+Na^+$]: 626.2783, found: 626.2781.

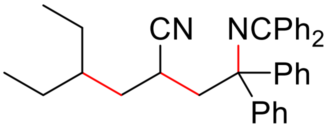

17

2-(2-((Diphenylmethylene)amino)-2,2-diphenylethyl)-4-ethylhexanenitrile (17)

Following the general procedure, benzophenone *O*-(2-ethylbutanoyl) oxime **1k** (88.5 mg, 0.3 mmol), ethene-1,1-diyldibenzene **2a** (36.0 mg, 0.2 mmol), and acrylonitrile **3a** (21.2 mg, 0.4 mmol) were used. Purification by column chromatography using pre-basified silica with pentane/EtOAc (400:1 to 200:1, v/v) as eluent afforded **17** as a white solid (47 mg, 49% yield). ^{1}H NMR (400 MHz, $CDCl_3$): δ = 0.63 (t, *J* = 7.4 Hz, 3H), 0.72 (t, *J* = 7.4 Hz, 3H), 0.95-1.24 (m, 5H), 1.24-1.36 (m, 1H), 1.47-1.54 (m, 1H), 2.34 (dd, *J* = 13.6, 3.3 Hz, 1H), 2.65 (m, 1H), 2.92 (dd, *J* = 13.6, 8.2 Hz, 1H), 6.58 (d, *J* = 5.7 Hz, 2H), 6.99-7.19 (m, 9H), 7.23-7.27 (m, 2H), 7.32-7.43 (m, 3H), 7.44-7.53 (m, 2H), 7.69-7.76 (m, 2H) ppm. ^{13}C NMR (101 MHz, $CDCl_3$): δ = 9.7, 10.7, 24.0, 24.7, 25.3, 37.7, 38.1, 44.3, 68.6, 123.2, 126.4, 126.5, 126.9, 127.2, 127.5, 128.00, 128.04, 128.2, 128.7, 130.2, 138.6, 141.6, 148.0, 148.4, 168.8 ppm. HRMS (ESI): m/z calculated for [$C_{35}H_{36}N_2Na$] [M+Na$^+$]: 507.2771, found: 507.2767.

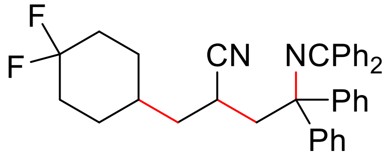

18

2-((4,4-Difluorocyclohexyl)methyl)-4-((diphenylmethylene)amino)-4,4-diphenylbutanenitrile (18)

Following the general procedure, benzophenone *O*-(4,4-difluorocyclohexanecarbonyl) oxime **1l** (103.0 mg, 0.3 mmol), ethene-1,1-diyldibenzene **2a** (36.0 mg, 0.2 mmol), and acrylonitrile **3a** (21.2 mg, 0.4 mmol) were used. Purification by column chromatography using pre-basified silica with pentane/EtOAc (400:1 to 100:1, v/v) as eluent afforded **18** as a white solid (56 mg, 53% yield). ^{1}H NMR (400 MHz, $CDCl_3$): δ = 0.87-1.20 (m, 3H), 1.32-1.43 (m, 2H), 1.47-1.54 (m, 2H), 1.56-1.70 (m, 2H), 1.85-2.04 (m, 2H), 2.33 (dd, *J* = 13.6, 3.5 Hz, 1H), 2.69 (m, 1H), 2.94 (dd, *J* = 13.6, 7.8 Hz, 1H), 6.56 (d, *J* = 7.5 Hz, 2H), 6.99-7.20 (m, 9H), 7.23-7.27 (m, 2H), 7.33-7.42 (m, 3H), 7.45-7.47 (m, 2H), 7.65-7.74 (m, 2H) ppm. ^{13}C{^{1}H,^{19}F} NMR (126 MHz, $CDCl_3$): δ = 24.5, 27.8, 29.1, 33.1, 33.3, 33.3, 40.1, 44.2, 68.6, 122.8, 123.4, 126.6, 126.6, 127.0, 127.2, 127.5, 128.0, 128.1, 128.1, 128.2, 128.7, 130.4, 138.5, 141.5, 147.7, 148.2, 169.0 ppm. ^{19}F{^{1}H} NMR (376 MHz, $CDCl_3$) δ = -102.08 (d, *J* = 235.2 Hz), -92.04 (d, *J* =

235.5 Hz) ppm. HRMS (ESI): m/z calculated for [$C_{36}H_{34}F_2N_2Na$] [M+Na$^+$]: 555.2582, found: 555.2584.

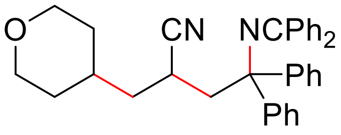

19

4-((Diphenylmethylene)amino)-4,4-diphenyl-2-((tetrahydro-2*H*-pyran-4-yl)methyl)butanenitrile (19)

Following the general procedure, benzophenone *O*-tetrahydro-2*H*-pyran-4-carbonyl oxime **1m** (92.7 mg, 0.3 mmol), ethene-1,1-diyldibenzene **2a** (36.0 mg, 0.2 mmol), and acrylonitrile **3a** (21.2 mg, 0.4 mmol) were used. Purification by column chromatography using pre-basified silica with pentane/EtOAc (100:1 to 30:1, v/v) as eluent afforded **19** as a white solid (68 mg, 68% yield). ^{1}H NMR (400 MHz, $CDCl_3$): δ = 0.88-1.02 (m, 1H), 1.04-1.29 (m, 3H), 1.36-1.42 (m, 1H), 1.47-1.58 (m, 2H), 2.31-2.36 (m, 1H), 2.69-2.76 (m, 1H), 2.91-2.96 (m, 1H), 3.19-3.31 (m, 2H), 3.78-3.92 (m, 2H), 6.57 (m, 2H), 6.99-7.21 (m, 9H), 7.23-7.27 (m, 2H), 7.30-7.52 (m, 5H), 7.69-7.72 (m, 2H) ppm. ^{13}C NMR (101 MHz, $CDCl_3$): δ = 23.5, 31.9, 32.5, 32.8, 41.2, 44.0, 67.68, 67.73, 68.5, 123.0, 126.51, 126.56, 127.0, 127.1, 127.5, 128.0, 128.1, 128.2, 128.7, 130.3, 138.5, 141.5, 147.7, 148.2, 168.9 ppm. HRMS (ESI): m/z calculated for [$C_{35}H_{34}N_2NaO$] [M+Na$^+$]: 521.2569, found: 521.2565.

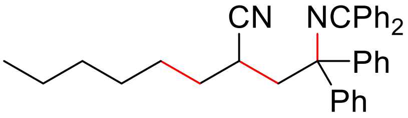

20

2-(2-((diphenylmethylene)amino)-2,2-diphenylethyl)octanenitrile (20)

Following the general procedure, benzophenone *O*-hexanoyl oxime **1n** (88.5 mg, 0.3 mmol), ethene-1,1-diyldibenzene **2a** (36.0 mg, 0.2 mmol), and acrylonitrile **3a** (21.2 mg, 0.4 mmol) were used. Purification by column chromatography using pre-basified silica with pentane/EtOAc (400:1 to 100:1, v/v) as eluent afforded **20** as a white solid (47 mg, 56% yield). ^{1}H NMR (400 MHz, $CDCl_3$): δ = 0.84 (t, *J* = 7.1 Hz, 3H), 1.04-1.47 (m, 10H), 2.35 (dd, *J* = 13.6, 3.7 Hz, 1H), 2.58 (m, 1H), 2.86 (dd, *J* = 13.6, 8.3 Hz,

1H), 6.55 (d, J = 7.2 Hz, 2H), 6.97-7.25 (m, 11H), 7.31-7.49 (m, 5H), 7.66-7.74 (m, 2H) ppm. ^{13}C NMR (101 MHz, $CDCl_3$): δ = 14.1, 22.6, 26.7, 26.7, 28.6, 31.5, 34.1, 43.6, 68.5, 122.9, 126.5, 126.5, 127.0, 127.3, 127.5, 128.04, 128.08, 128.1, 128.2, 128.7, 130.3, 138.6, 141.6, 148.1, 148.5, 168.7 ppm. HRMS (ESI): m/z calculated for $[C_{35}H_{36}N_2Na]$ $[M+Na^+]$: 507.2771, found: 507.2768.

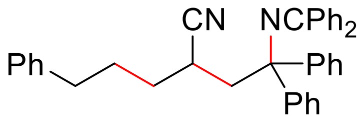

21

2-(2-((Diphenylmethylene)amino)-2,2-diphenylethyl)-5-phenylpentanenitrile (21)

Following the general procedure, benzophenone *O*-(3-phenylpropanoyl) oxime **1o** (98.7 mg, 0.3 mmol), ethene-1,1-diyldibenzene **2a** (36.0 mg, 0.2 mmol), and acrylonitrile **3a** (21.2 mg, 0.4 mmol) were used. Purification by column chromatography using pre-basified silica with pentane/EtOAc (400:1 to 200:1, v/v) as eluent afforded **21** as colorless oil (47 mg, 60% yield). ^{1}H NMR (400 MHz, $CDCl_3$): δ = 1.34 (ddt, J = 13.5, 10.9, 5.8 Hz, 1H), 1.46 (dtd, J = 14.4, 9.3, 5.1 Hz, 1H), 1.57-1.76 (m, 2H), 2.31 (dd, J = 13.6, 3.6 Hz, 1H), 2.35-2.55 (m, 2H), 2.56-2.67 (m, 1H), 2.85 (dd, J = 13.5, 8.4 Hz, 1H), 6.50 (s, 2H), 6.94-7.25 (m, 16H), 7.32-7.46 (m, 5H), 7.67-7.73 (m, 2H) ppm. ^{13}C NMR (101 MHz, $CDCl_3$): δ = 26.5, 28.2, 33.2, 34.9, 43.5, 68.3, 122.6, 126.0, 126.5, 127.0, 127.2, 127.4, 127.9, 128.02, 128.07, 128.2, 128.5, 128.7, 130.3, 138.5, 141.4, 141.5, 148.0, 148.5, 168.7 ppm. HRMS (ESI): m/z calculated for $[C_{38}H_{34}N_2Na]$ $[M+Na^+]$: 541.2620, found: 541.2615.

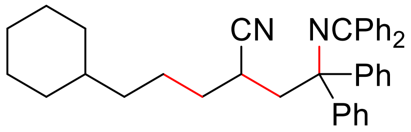

22

5-Cyclohexyl-2-(2-((diphenylmethylene)amino)-2,2-diphenylethyl)pentanenitrile (22)

Following the general procedure, benzophenone *O*-(3-cyclohexylpropanoyl) oxime **1p** (100.5 mg, 0.3 mmol), ethene-1,1-diyldibenzene **2a** (36.0 mg, 0.2 mmol), and acrylonitrile **3a** (21.2 mg, 0.4 mmol) were used. Purification by column

chromatography using pre-basified silica with pentane/EtOAc (400:1 to 300:1, v/v) as eluent afforded **22** as pale yellow oil (54 mg, 52% yield). ^{1}H NMR (400 MHz, $CDCl_3$): δ = 0.74-1.13 (m, 6H), 1.15-1.46 (m, 6H), 1.56-1.68 (m, 5H), 2.33-2.38 (m, 1H), 2.55-2.62 (m, 1H), 2.84-2.90 (m, 1H), 6.54-6.56 (m, 2H), 7.00-7.19 (m, 9H), 7.21-7.27 (m, 2H), 7.32-7.40 (m, 3H), 7.41-7.53 (m, 2H), 7.69-7.72 (m, 2H) ppm. ^{13}C NMR (101 MHz, $CDCl_3$): δ = 24.0, 28.5, 26.7, 26.8, 33.3, 33.4, 34.3, 36.6, 37.3, 43.6, 68.4, 122.9, 126.48, 126.49, 127.0, 127.3, 127.5, 128.00, 128.03, 128.08, 128.2, 128.7, 129.4, 130.3, 138.6, 141.6, 148.1, 148.5, 168.7 ppm. HRMS (ESI): m/z calculated for [$C_{38}H_{40}N_2Na$] [$M+Na^+$]: 547.3089, found: 547.3084.

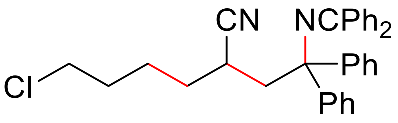

23

6-Chloro-2-(2-((diphenylmethylene)amino)-2,2-diphenylethyl)hexanenitrile (23)

Following the general procedure, benzophenone *O*-(4-chlorobutanoyl) oxime **1q** (90.3 mg, 0.3 mmol), ethene-1,1-diyldibenzene **2a** (36.0 mg, 0.2 mmol), and acrylonitrile **3a** (21.2 mg, 0.4 mmol) were used. Purification by column chromatography using pre-basified silica with pentane/EtOAc (400:1 to 200:1, v/v) as eluent afforded **23** as a white solid (54 mg, 55% yield). ^{1}H NMR (400 MHz, $CDCl_3$): δ = 1.25-1.71 (m, 6H), 2.37 (dd, *J* = 13.6, 3.9 Hz, 1H), 2.62 (m, 1H), 2.90 (dd, *J* = 13.6, 7.9 Hz, 1H), 3.39 (m, 2H), 6.55 (d, *J* = 7.4 Hz, 2H), 6.99-7.20 (m, 9H), 7.22-7.26 (m, 2H), 7.33-7.41 (m, 3H), 7.42-7.46 (m, 2H), 7.67-7.74 (m, 2H) ppm. ^{13}C NMR (101 MHz, $CDCl_3$): δ = 24.2, 26.6, 31.8, 33.2, 43.6, 44.4, 68.4, 122.6, 126.6, 126.6, 127.0, 127.2, 127.5, 127.99, 128.02, 128.07, 128.12, 128.2, 128.7, 130.4, 138.5, 141.5, 147.9, 148.3, 168.8 ppm. HRMS (ESI): m/z calculated for [$C_{33}H_{31}N_2ClNa$] [$M+Na^+$]: 513.2068, found: 513.2071.

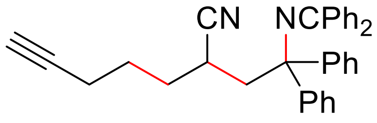

24

2-(2-((Diphenylmethylene)amino)-2,2-diphenylethyl)hept-6-ynenitrile (24)

Following the general procedure, benzophenone *O*-pent-4-ynoyl oxime **1r** (83.1 mg, 0.3 mmol), ethene-1,1-diyldibenzene **2a** (36.0 mg, 0.2 mmol), and acrylonitrile **3a** (21.2 mg, 0.4 mmol) were used. Purification by column chromatography using pre-basified silica with pentane/EtOAc (400:1 to 200:1, v/v) as eluent afforded **24** as colorless oil (58 mg, 62% yield). ^{1}H NMR (400 MHz, $CDCl_3$): δ = 1.46-1.67 (m, 4H), 1.87 (t, *J* = 2.4 Hz, 1H), 2.04-2.08 (m, 2H), 2.35-2.40 (m, 1H), 2.60-2.67 (m, 1H), 2.82-2.91 (m, 1H), 6.48-6.56 (m, 2H), 6.99-7.19 (m, 9H), 7.20-7.28 (m, 2H), 7.30-7.44 (m, 5H), 7.68-7.72 (m, 2H) ppm. ^{13}C NMR (101 MHz, $CDCl_3$): δ = 17.9, 25.5, 26.4, 33.0, 43.7, 68.4, 69.2, 83.3, 122.4, 126.6, 127.0, 127.2, 127.40, 127.41, 127.49, 127.67, 127.70, 128.0, 128.1, 128.22, 128.28, 128.29, 128.8, 130.3, 138.5, 141.5, 147.9, 148.4, 168.8 ppm. HRMS (ESI): m/z calculated for [$C_{34}H_{30}N_2Na$] [M+Na^+]: 489.2307, found: 489.2300.

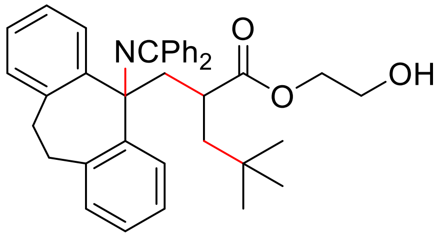

25

2-Hydroxyethyl 2-((5-((diphenylmethylene)amino)-10,11-dihydro-5*H*-dibenzo[*a,d*][7]annulen-5-yl)methyl)-4,4-dimethylpentanoate (25)

Following the general procedure, benzophenone *O*-pivaloyl oxime **1a** (84.3 mg, 0.3 mmol), 5-methylene-10,11-dihydro-5*H*-dibenzo[a,d][7]annulene **2e** (41.2 mg, 0.2 mmol), and 2-hydroxyethyl acrylate **3b** (46.4 mg, 0.4 mmol) were used. Purification by column chromatography using pre-basified silica with pentane/EtOAc (10:1 to 8:1, v/v) as eluent afforded **25** as colorless oil (75 mg, 67% yield). ^{1}H NMR (400 MHz, $CDCl_3$): δ = 0.69 (s, 9H), 1.24-1.32 (m, 1H), 1.49-1.54 (m, 1H), 1.85-1.87 (m, 1H), 2.28-2.39 (m, 3H), 2.68-2.85 (m, 3H), 3.24-3.30 (m, 1H), 3.66 (m, 2H), 3.96-4.16 (m, 2H), 6.02-6.05 (m, 2H), 6.69-6.76 (m, 3H), 6.84-6.86 (m, 1H), 6.91-6.99 (m, 2H), 7.06-7.18 (m, 3H), 7.31-7.41 (m, 3H), 7.54-7.61 (m, 4H) ppm. ^{13}C NMR (101 MHz, $CDCl_3$): δ = 29.3, 31.3, 36.1, 36.2, 37.8, 48.7, 54.9, 61.5, 65.8, 72.8, 125.6, 125.97, 126.04, 126.55, 126.66, 126.70, 127.2, 128.0, 128.8, 129.1, 129.3, 130.0, 132.2, 132.3, 139.2, 141.7,

141.8, 141.9, 145.3, 146.8, 169.6, 178.5 ppm. HRMS (ESI): m/z calculated for [$C_{38}H_{41}NNaO_3$] [M+Na$^+$]: 582.2984, found: 582.2984.

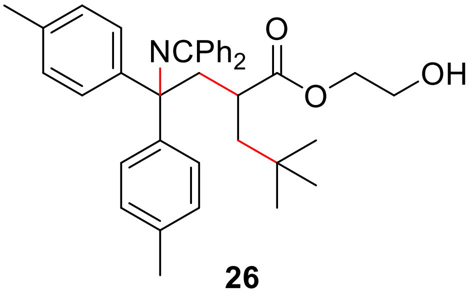

26

2-Hydroxyethyl 2-(2-((diphenylmethylene)amino)-2,2-di-*p*-tolylethyl)-4,4-dimethylpentanoate (26)

Following the general procedure, benzophenone *O*-pivaloyl oxime **1a** (84.3 mg, 0.3 mmol), 4,4'-(ethene-1,1-diyl)bis(methylbenzene) **2b** (41.6 mg, 0.2 mmol), and 2-hydroxyethyl acrylate **3b** (46.4 mg, 0.4 mmol) were used. Purification by column chromatography using pre-basified silica with pentane/EtOAc (10:1 to 8:1, v/v) as eluent afforded **26** as colorless oil (78 mg, 70% yield). ^{1}H NMR (400 MHz, $CDCl_3$): δ = 0.69 (s, 9H), 1.22 (dd, *J* = 14.0 Hz, 3.6 Hz, 1H), 1.66-1.77 (m, 2H), 2.21 (s, 3H), 2.27-2.31 (m, 1H), 2.33 (s, 3H), 2.55-2.60 (m, 1H), 2.98-3.04 (m, 1H), 3.42-3.47 (m, 1H), 3.49-3.59 (m, 2H), 3.67-3.72 (m, 1H), 6.67-6.76 (m, 4H), 6.84-6.86 (m, 2H), 6.96-7.00 (m, 2H), 7.05-7.11 (m, 3H), 7.30-7.39 (m, 5H), 7.64-7.67 (m, 2H) ppm. ^{13}C NMR (101 MHz, $CDCl_3$): δ = 21.0, 21.1, 29.4, 31.1, 37.6, 47.1, 49.8, 61.1, 66.0, 68.8, 126.4, 127.0, 127.2, 127.9, 128.0, 128.2, 128.5, 128.6, 129.9, 135.1, 135.3, 139.0, 142.4, 145.6, 146.7, 167.4, 178.3 ppm. HRMS (ESI): m/z calculated for [$C_{38}H_{43}NNaO_3$] [M+Na$^+$]: 584.3141, found: 584.3141.

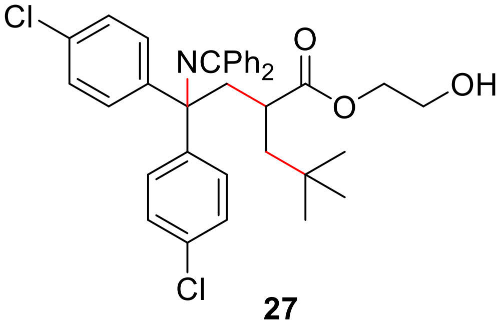

27

2-Hydroxyethyl 2-(2,2-bis(4-chlorophenyl)-2-((diphenylmethylene)amino)ethyl)-4,4-dimethylpentanoate (27)

Following the general procedure, benzophenone *O*-pivaloyl oxime **1a** (84.3 mg, 0.3 mmol), 4,4'-(ethene-1,1-diyl)bis(chlorobenzene) **2c** (49.6 mg, 0.2 mmol), and 2-hydroxyethyl acrylate **3b** (46.4 mg, 0.4 mmol) were used. Purification by column chromatography using pre-basified silica with pentane/EtOAc (10:1 to 8:1, v/v) as eluent afforded **27** as colorless oil (72 mg, 60% yield). ^{1}H NMR (400 MHz, $CDCl_3$): δ = 0.68 (s, 9H), 1.19 (dd, *J* = 14.4 Hz, 3.6 Hz, 1H), 1.60-1.70 (m, 2H), 2.20-2.24 (m, 1H), 2.45-2.51 (m, 1H), 3.00-3.06 (m, 1H), 3.34-3.39 (m, 1H), 3.49-3.58 (m, 2H), 3.65-3.70 (m, 1H), 6.52-6.90 (m, 6H), 7.05 (m, 2H), 7.12-7.16 (m, 1H), 7.24-7.26 (m, 2H), 7.30-7.51 (m, 5H), 7.59-7.62 (m, 2H) ppm. ^{13}C NMR (101 MHz, $CDCl_3$): δ = 29.3, 31.1, 37.5, 47.1, 49.9, 61.1, 66.2, 68.6, 127.0, 127.2, 127.4, 127.7, 128.1, 128.7, 129.5, 130.3, 132.0, 132.2, 138.7, 141.8, 146.5, 147.8, 168.5, 178.0 ppm. HRMS (ESI): m/z calculated for [$C_{36}H_{37}Cl_2NNaO_3$] [M+Na$^+$]: 624.2048, found: 624.2043.

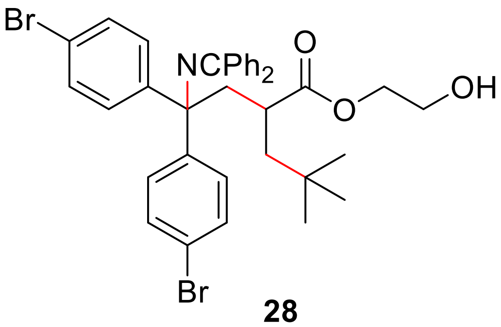

28

2-Hydroxyethyl 2-(2,2-bis(4-bromophenyl)-2-((diphenylmethylene)amino)ethyl)-4,4-dimethylpentanoate (28)

Following the general procedure, benzophenone *O*-pivaloyl oxime **1a** (84.3 mg, 0.3 mmol), 4,4'-(ethene-1,1-diyl)bis(bromobenzene) **2d** (67.0 mg, 0.2 mmol), and 2-hydroxyethyl acrylate **3b** (46.4 mg, 0.4 mmol) were used. Purification by column chromatography using pre-basified silica with pentane/EtOAc (10:1 to 8:1, v/v) as eluent afforded **28** as colorless oil (92 mg, 67% yield). ^{1}H NMR (400 MHz, $CDCl_3$): δ = 0.69 (s, 9H), 1.20 (dd, *J* = 14.0 Hz, 3.2 Hz, 1H), 1.66-1.71 (m, 2H), 2.20-2.24 (m, 1H), 2.46-2.52 (m, 1H), 3.00-3.05 (m, 1H), 3.34-3.40 (m, 1H), 3.48-3.59 (m, 2H), 3.65-3.71 (m, 1H), 6.74-6.76 (m, 4H), 7.04-7.07 (m, 4H), 7.13-7.18 (m, 1H), 7.30-7.47 (m,

7H), 7.60-7.63 (m, 2H) ppm. ^{13}C NMR (101 MHz, $CDCl_3$): δ = 29.3, 31.1, 37.4, 47.0, 49.9, 61.0, 66.2, 68.6, 120.2, 120.3, 127.0, 127.1, 127.4, 128.0, 128.6, 129.9, 130.3, 130.6, 131.0, 138.6, 141.7, 146.9, 148.2, 168.5, 177.9 ppm. HRMS (ESI): m/z calculated for [$C_{36}H_{37}Br_2NNaO_3$] [$M+Na^+$]: 712.1038, found: 712.1038.

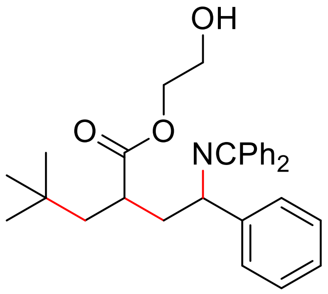

29, 54:46 d.r.

2-Hydroxyethyl 2-(2-((diphenylmethylene)amino)-2-phenylethyl)-4,4-dimethylpentanoate (29)

Following the general procedure, benzophenone *O*-pivaloyl oxime **1a** (84.3 mg, 0.3 mmol), styrene **2f** (20.8 mg, 0.2 mmol), and 2-hydroxyethyl acrylate **3b** (46.4 mg, 0.4 mmol) were used. Purification by column chromatography using pre-basified silica with pentane/EtOAc (10:1 to 8:1, v/v) as eluent afforded **29** as colorless oil in 54:46 diastereomeric ratio (51 mg, 56% yield). Combined NMR data for both the isomers are reported here. ^{1}H NMR (400 MHz, $CDCl_3$): δ = 0.78-0.84 (m, 9H), 1.18-1.26 (m, 1H), 1.66-1.73 (m, 1H), 1.87-2.13 (m, 2H), 2.18-2.53 (m, 2H), 3.60-3.64 (m, 2H), 3.88-4.06 (m, 2H), 4.32-4.37 (m, 1H), 7.02-7.09 (m, 2H), 7.21-7.44 (m, 11H), 7.63-7.68 (m, 2H) ppm. ^{13}C NMR (101 MHz, $CDCl_3$): δ = 29.50, 29.52, 30.97, 31.10, 39.2, 39.6, 44.7, 45.0, 47.0, 61.1, 61.4, 64.9, 65.3, 66.1, 126.9, 127.1, 127.2, 127.4, 128.0, 128.07, 128.12, 128.14, 128.3, 128.47, 128.50, 128.52, 128.56, 128.73, 128.74, 130.12, 130.16, 136.9, 137.1, 139.9, 140.1, 144.3, 145.0, 167.6, 167.9, 177.59, 177.65 ppm. HRMS (ESI): m/z calculated for [$C_{30}H_{35}NNaO_3$] [$M+Na^+$]: 480.2515, found: 480.2518.

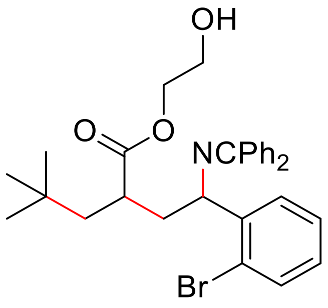

30, 50:50 d.r.

2-Hydroxyethyl 2-(2-(2-bromophenyl)-2-((diphenylmethylene)amino)ethyl)-4,4-dimethylpentanoate (30 and 30')

Following the general procedure, benzophenone *O*-pivaloyl oxime **1a** (84.3 mg, 0.3 mmol), 1-bromo-2-vinylbenzene **2g** (36.4 mg, 0.2 mmol), and 2-hydroxyethyl acrylate **3b** (46.4 mg, 0.4 mmol) were used. Purification by column chromatography using pre-basified silica with pentane/EtOAc (10:1 to 8:1, v/v) as eluent afforded two separable diastereomers (**30** and **30')** as colorless oil in 50:50 diastereomeric ratio (**30**: 26 mg, **30'**: 26 mg, 48% combined yield). Diastereomer **30**: ^{1}H NMR (400 MHz, $CDCl_3$): δ = 0.87 (s, 9H), 1.28-1.32 (m, 1H), 1.72-1.77 (m, 1H), 1.97 (t, *J* = 6.0 Hz, 1H), 2.03-2.09 (m, 1H), 2.15-2.21 (m, 1H), 2.59-2.66 (m, 1H), 3.62-3.66 (m, 2H), 3.93-4.06 (m, 2H), 4.70-4.73 (m, 1H), 6.93-6.95 (m, 2H), 7.03-7.08 (m, 1H), 7.25-7.45 (m, 8H), 7.58-7.71 (m, 3H) ppm. ^{13}C NMR (101 MHz, $CDCl_3$): δ = 29.6, 31.2, 39.4, 43.9, 47.2, 61.6, 63.2, 66.5, 122.3, 127.8, 127.9, 128.19, 128.21, 128.4, 128.6, 128.8, 129.6, 130.3, 132.7, 136.9, 140.0, 144.6, 169.5, 177.4 ppm. Diastereomer **30'**: ^{1}H NMR (400 MHz, $CDCl_3$): δ = 0.82 (s, 9H), 1.29-1.30 (m, 1H), 1.64-1.71 (m, 1H), 1.87-1.93 (m, 1H), 2.04-2.07 (m, 1H), 2.31-2.39 (m, 1H), 2.47-2.53 (m, 1H), 3.66 (m, 2H), 3.93-3.99 (m, 1H), 4.06-4.12 (m, 1H), 4.81-4.84 (m, 1H), 6.96-6.98 (m, 2H), 7.04-7.08 (m, 1H), 7.25-7.45 (m, 8H), 7.66-7.71 (m, 3H) ppm. ^{13}C NMR (101 MHz, $CDCl_3$): δ = 29.6, 31.0, 40.0, 43.1, 46.2, 61.3, 63.5, 66.3, 122.4, 127.8, 127.9, 128.2, 128.3, 128.58, 128.64, 128.9, 129.6, 130.4, 132.6, 136.9, 139.8, 144.4, 169.5, 177.6 ppm. HRMS (ESI): m/z calculated for [$C_{30}H_{34}BrNNaO_3$] [M+Na^+]: 558.1620, found: 558.1621.

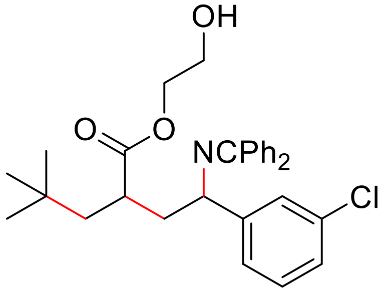

31, 56:44 d.r.

2-Hydroxyethyl 2-(2-(3-chlorophenyl)-2-((diphenylmethylene)amino)ethyl)-4,4-dimethylpentanoate (31)

Following the general procedure, benzophenone *O*-pivaloyl oxime **1a** (84.3 mg, 0.3 mmol), 1-chloro-3-vinylbenzene **2h** (27.7 mg, 0.2 mmol), and 2-hydroxyethyl acrylate **3b** (46.4 mg, 0.4 mmol) were used. Purification by column chromatography using pre-basified silica with pentane/EtOAc (10:1 to 6:1, v/v) as eluent afforded **31** as a colorless sticky solid in 56:44 diastereomeric ratio (30 mg, 30% yield). Combined NMR data for both the isomers are reported here. ^{1}H NMR (400 MHz, $CDCl_3$): δ = 0.74-0.89 (m, 9H), 1.15-1.27 (m, 1H), 1.63-1.75 (m, 1H), 1.80 (s, 1H), 1.89-2.53 (m, 3H), 3.55-3.73 (m, 2H), 3.86-4.10 (m, 2H), 4.27-4.38 (m, 1H), 6.98-7.24 (m, 5H), 7.26-7.48 (m, 7H), 7.60-7.74 (m, 2H) ppm. ^{13}C NMR (101 MHz, $CDCl_3$): δ = 29.5, 29.5, 31.0, 31.1, 39.1, 39.5, 44.7, 45.0, 47.0, 47.0, 61.1, 61.4, 64.4, 64.8, 66.1, 66.1, 125.3, 125.6, 127.1, 127.3, 127.3, 127.6, 127.9, 128.0, 128.2, 128.2, 128.4, 128.6, 128.6, 128.7, 128.8, 129.8, 130.3, 130.4, 134.3, 134.3, 136.7, 136.9, 139.6, 139.8, 146.4, 147.1, 168.1, 168.6, 177.4, 177.5 ppm. HRMS (ESI): m/z calculated for [$C_{30}H_{34}NO_3ClNa$] [$M+Na^+$]: 514.2119, found: 514.2120.

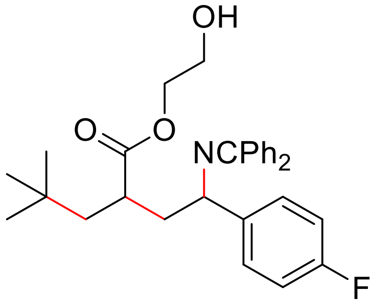

32, 56:44 d.r.

2-Hydroxyethyl 2-(2-((diphenylmethylene)amino)-2-(4-fluorophenyl)ethyl)-4,4-dimethylpentanoate (32)

Following the general procedure, benzophenone *O*-pivaloyl oxime **1a** (84.3 mg, 0.3 mmol), 1-fluoro-4-vinylbenzene **2i** (24.4 mg, 0.2 mmol), and 2-hydroxyethyl acrylate **3b** (46.4 mg, 0.4 mmol) were used. Purification by column chromatography using pre-basified silica with pentane/EtOAc (10:1 to 6:1, v/v) as eluent afforded **32** as a colorless sticky solid in 56:44 diastereomeric ratio (36 mg, 38% yield). Combined NMR data for both the isomers are reported here. ^{1}H NMR (400 MHz, $CDCl_3$): δ = 0.75-0.88 (m, 9H), 1.15-1.26 (m, 1H), 1.69 (dt, *J* = 14.1, 8.5 Hz, 1H), 1.85-2.52 (m, 4H), 3.53-3.73 (m, 2H), 3.86-4.10 (m, 2H), 4.25-4.36 (m, 1H), 6.93-7.10 (m, 4H), 7.17-7.25 (m, 2H), 7.29-7.47 (m, 6H), 7.60-7.72 (m, 2H) ppm. ^{13}C{^{1}H,^{19}F} NMR (151 MHz, $CDCl_3$): δ = 29.5, 29.5, 31.0, 31.1, 39.1, 39.5, 44.8, 45.1, 47.0, 47.1, 61.1, 61.3, 64.1, 64.6, 66.1, 66.1, 115.3, 115.3, 127.9, 128.0, 128.2, 128.2, 128.3, 128.5, 128.6, 128.6, 128.7, 128.9, 130.2, 130.3, 136.8, 137.0, 139.8, 139.9, 140.0, 140.7, 161.8, 161.9, 167.6, 168.1, 177.5, 177.5 ppm. HRMS (ESI): m/z calculated for [$C_{30}H_{34}NO_3FNa$] [M+Na$^+$]: 498.2415, found: 498.2406.

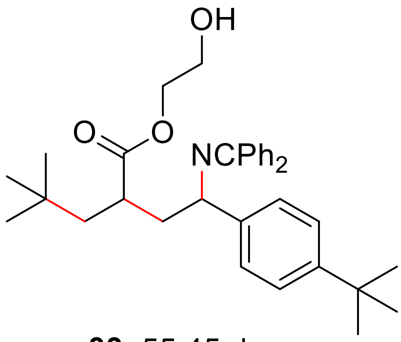

33, 55:45 d.r.

2-Hydroxyethyl 2-(2-(4-(*tert*-butyl)phenyl)-2-((diphenylmethylene)amino)ethyl)-4,4-dimethylpentanoate (33)

Following the general procedure, benzophenone *O*-pivaloyl oxime **1a** (84.3 mg, 0.3 mmol), 1-(*tert*-butyl)-4-vinylbenzene **2j** (32.0 mg, 0.2 mmol), and 2-hydroxyethyl acrylate **3b** (46.4 mg, 0.4 mmol) were used. Purification by column chromatography using pre-basified silica with pentane/EtOAc (10:1 to 8:1, v/v) as eluent afforded **33** as colorless oil in 55:45 diastereomeric ratio (64 mg, 62% yield). Combined NMR data

for both the isomers are reported here. ^{1}H NMR (400 MHz, $CDCl_3$): δ = 0.78-0.84 (m, 9H), 1.31 (s, 9H), 1.65-2.11 (m, 4H), 2.18-2.52 (m, 2H), 3.57-3.68 (m, 2H), 3.85-3.93 (m, 1H), 3.96-4.03 (m, 1H), 4.31-4.37 (m, 1H), 7.04-7.12 (m, 2H), 7.18-7.21 (m, 2H), 7.28-7.46 (m, 8H), 7.63-7.67 (m, 2H) ppm. ^{13}C NMR (101 MHz, $CDCl_3$): δ = 29.51, 29.52, 31.0, 31.1, 31.5, 34.56, 34.58, 39.2, 39.6, 44.7, 44.9, 46.98, 47.00, 61.1, 61.4, 64.6, 65.1, 66.0, 125.33, 125.36, 126.8, 127.0, 128.07, 128.08, 128.10, 128.18, 128.3, 128.4, 128.5, 128.72, 128.75, 130.02, 130.07, 136.9, 137.2, 140.0, 140.2, 141.2, 141.7, 149.7, 149.8, 167.2, 167.5, 177.6, 177.7 ppm. HRMS (ESI): m/z calculated for [$C_{34}H_{43}NNaO_3$] [M+Na$^+$]: 536.3141, found: 536.3138.

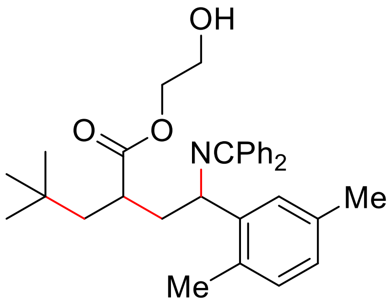

34, 58:42 d.r.

2-Hydroxyethyl 2-(2-(2,5-dimethylphenyl)-2-((diphenylmethylene)amino)ethyl)-4,4-dimethylpentanoate (34 and 34')

Following the general procedure, benzophenone *O*-pivaloyl oxime **1a** (84.3 mg, 0.3 mmol), 1,4-dimethyl-2-vinylbenzene **2k** (26.4 mg, 0.2 mmol), and 2-hydroxyethyl acrylate **3b** (46.4 mg, 0.4 mmol) were used. Purification by column chromatography using pre-basified silica with pentane/EtOAc (10:1 to 8:1, v/v) as eluent afforded two separable diastereomers (**34** and **34')** as colorless oils in 58:42 diastereomeric ratio (**34**: 11 mg, **34'**: 15 mg, 27% combined yield). Diastereomer **34**: ^{1}H NMR (400 MHz, $CDCl_3$): δ = 0.81 (s, 9H), 1.21 (dd, *J* = 14.1, 2.9 Hz, 1H), 1.64-1.83 (m, 5H), 2.08 (s, 1H), 2.28 (s, 3H), 2.35-2.56 (m, 2H), 3.61-3.73 (m, 2H), 3.91-4.01 (m, 1H), 4.05-4.15 (m, 1H), 4.54 (dd, *J* = 9.3, 3.9 Hz, 1H), 6.86-7.05 (m, 4H), 7.30-7.42 (m, 7H), 7.64-7.71 (m, 2H) ppm. ^{13}C NMR (101 MHz, $CDCl_3$): δ = 18.5, 21.3, 29.6, 31.0, 40.1, 43.7, 46.0, 60.9, 61.3, 66.3, 127.3, 127.8, 128.2, 128.2, 128.5, 128.8, 130.1, 130.8, 135.8, 137.5, 140.0, 143.6, 168.3, 177.8 ppm. HRMS (ESI): m/z calculated for [$C_{32}H_{39}NO_3Na$] [M+Na$^+$]: 508.2822, found: 508.2823. Diastereomer **34'**: ^{1}H NMR (400 MHz, $CDCl_3$):

δ = 0.90 (s, 9H), 1.32 (dd, *J* = 14.1, 3.6 Hz, 1H), 1.71 (dd, *J* = 14.1, 8.5 Hz, 1H), 1.79 (s, 3H), 1.92 (t, *J* = 6.1 Hz, 1H), 2.01 (ddd, *J* = 13.6, 10.7, 2.8 Hz, 1H), 2.16 (ddd, *J* = 13.7, 10.2, 3.4 Hz, 1H), 2.27 (s, 3H), 2.60-2.71 (m, 1H), 3.61-3.71 (m, 2H), 3.89-4.14 (m, 2H), 4.35 (dd, *J* = 10.2, 2.8 Hz, 1H), 6.86-6.99 (m, 4H), 7.28-7.46 (m, 7H), 7.63-7.78 (m, 2H) ppm. ^{13}C NMR (101 MHz, $CDCl_3$): δ = 18.3, 21.3, 29.6, 31.2, 39.8, 44.7, 47.5, 60.8, 61.5, 66.4, 127.1, 127.7, 128.0, 128.2, 128.2, 128.4, 128.7, 130.1, 130.1, 130.8, 135.7, 137.4, 140.2, 144.2, 168.5, 177.9 ppm. HRMS (ESI): m/z calculated for [$C_{32}H_{39}NO_3Na$] [M+Na$^+$]: 508.2822, found: 508.2820.

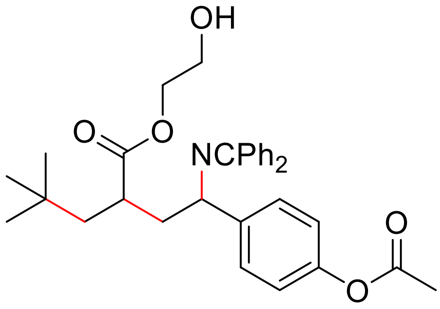

35, 55:45 d.r.

2-Hydroxyethyl 2-(2-(4-acetoxyphenyl)-2-((diphenylmethylene)amino)ethyl)-4,4-dimethylpentanoate (35)

Following the general procedure, benzophenone *O*-pivaloyl oxime **1a** (84.3 mg, 0.3 mmol), 4-vinylphenyl acetate **2l** (32.4 mg, 0.2 mmol), and 2-hydroxyethyl acrylate **3b** (46.4 mg, 0.4 mmol) were used. Purification by column chromatography using pre-basified silica with pentane/EtOAc (8:1 to 4:1, v/v) as eluent afforded **35** as colorless oil in 55:45 diastereomeric ratio (54 mg, 52% yield). Combined NMR data for both the isomers are reported here. ^{1}H NMR (400 MHz, $CDCl_3$): δ = 0.79-0.84 (m, 9H), 1.17-1.26 (m, 1H), 1.66-1.73 (m, 2H), 1.91-2.08 (m, 2H), 2.19-2.50 (m, 5H), 3.60-3.64 (m, 2H), 3.85-3.91 (m, 1H), 3.95-4.01 (m, 1H), 4.33-4.39 (m, 1H), 6.99-7.09 (m, 4H), 7.25-7.45 (m, 8H), 7.62-7.67 (m, 2H) ppm. ^{13}C NMR (101 MHz, $CDCl_3$): δ = 21.3, 29.5, 31.0, 31.1, 39.2, 39.4, 44.7, 45.0, 47.0, 47.1, 61.1, 61.3, 64.5, 64.7, 66.1, 121.49, 121.50, 127.9, 128.0, 128.1, 128.2, 128.3, 128.36, 128.40, 128.5, 128.6, 128.71, 128.73, 130.20, 130.24, 136.8, 137.0, 139.8, 139.9, 141.8, 142.3, 149.5, 149.6, 167.7, 167.9, 169.7,

169.8, 177.4, 177.5 ppm. HRMS (ESI): m/z calculated for [$C_{32}H_{37}NNaO_5$] [M+Na$^+$]: 538.2569, found: 538.2569.

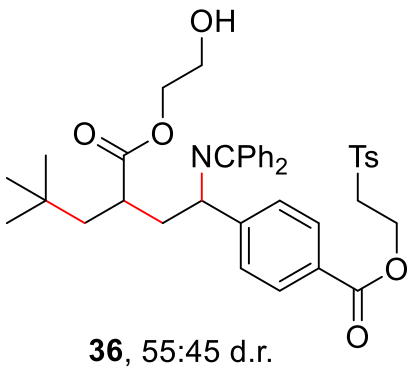

36, 55:45 d.r.

2-Tosylethyl 4-(1-((diphenylmethylene)amino)-3-((2-hydroxyethoxy)carbonyl)-5,5-dimethylhexyl)benzoate (36)

Following the general procedure, benzophenone *O*-pivaloyl oxime **1a** (84.3 mg, 0.3 mmol), 2-tosylethyl 4-vinylbenzoate **2m** (66.1 mg, 0.2 mmol), and 2-hydroxyethyl acrylate **3b** (46.4 mg, 0.4 mmol) were used. Purification by column chromatography using pre-basified silica with pentane/EtOAc (10/1 to 1/3, v/v) as eluent afforded **36** as a white solid in 55:45 diastereomeric ratio (35 mg, 26% yield). Combined NMR data for both the isomers are reported here. ^{1}H NMR (400 MHz, $CDCl_3$): δ = 0.75-0.88 (m, 9H), 1.13-1.27 (m, 1H), 1.65-1.73 (m, 1H), 1.74-2.58 (m, 7H), 3.53-3.72 (m, 4H), 3.85-4.09 (m, 2H), 4.30-4.43 (m, 1H), 4.55-4.67 (m, 2H), 6.92-7.07 (m, 2H), 7.22-7.48 (m, 10H), 7.60-7.70 (m, 4H), 7.77-7.84 (m, 2H) ppm. ^{13}C NMR (101 MHz, $CDCl_3$): δ = 21.7, 21.7, 29.5, 29.5, 31.0, 31.1, 39.1, 39.5, 44.5, 44.8, 47.0, 47.0, 55.4, 58.4, 61.1, 61.3, 64.6, 65.0, 66.1, 66.1, 127.0, 127.3, 127.6, 127.8, 127.9, 128.2, 128.4, 128.6, 128.7, 130.0, 130.2, 130.4, 136.6, 136.6, 145.1, 145.1, 165.8, 177.3, 177.3 ppm. HRMS (ESI): m/z calculated for [$C_{40}H_{45}NO_7SNa$] [M+Na$^+$]: 706.2809, found: 706.2813.

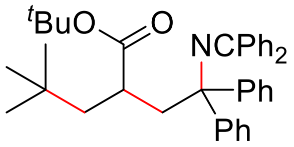

37

***tert*-Butyl 2-(2-((diphenylmethylene)amino)-2,2-diphenylethyl)-4,4-dimethylpentanoate (37)**

Following the general procedure, benzophenone *O*-pivaloyl oxime **1a** (84.3 mg, 0.3

mmol), ethene-1,1-diyldibenzene **2a** (36.0 mg, 0.2 mmol), and *tert*-butyl acrylate **3c** (51.2 mg, 0.4 mmol) were used. Purification by column chromatography using pre-basified silica with pentane/EtOAc (400:1 to 200:1, v/v) as eluent afforded **37** as colorless oil (76 mg, 70% yield). ^{1}H NMR (400 MHz, $CDCl_3$): δ = 0.63 (s, 9H), 1.11-1.15 (m, 1H), 1.24 (s, 9H), 1.51-1.59 (m, 1H), 2.32-2.37 (m, 1H), 2.47-2.53 (m, 1H), 2.88-2.92 (m, 1H), 6.68-6.69 (m, 2H), 6.97-7.03 (m, 5H), 7.06-7.14 (m, 4H), 7.16-7.20 (m, 2H), 7.31-7.44 (m, 5H), 7.67-7.70 (m, 2H) ppm. ^{13}C NMR (101 MHz, $CDCl_3$): δ = 27.9, 29.5, 30.9, 38.7, 46.1, 47.7, 69.3, 79.7, 125.8, 125.9, 126.5, 127.2, 127.4, 127.6, 127.7, 127.9, 128.4, 128.6, 128.8, 129.8, 139.2, 142.3, 149.3, 149.8, 167.3, 177.4 ppm. HRMS (ESI): m/z calculated for [$C_{38}H_{43}NNaO_2$] [$M+Na^+$]: 568.3191, found: 568.3189.

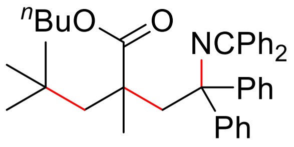

38

Butyl 2-(2-((diphenylmethylene)amino)-2,2-diphenylethyl)-2,4,4-trimethylpentanoate (38)

Following the general procedure, benzophenone *O*-pivaloyl oxime **1a** (84.3 mg, 0.3 mmol), ethene-1,1-diyldibenzene **2a** (36.0 mg, 0.2 mmol), and butyl methacrylate **3d** (56.8 mg, 0.4 mmol) were used. Purification by column chromatography using pre-basified silica with pentane/EtOAc (400:1 to 200:1, v/v) as eluent afforded **38** as colorless oil (46 mg, 41% yield). ^{1}H NMR (400 MHz, $CDCl_3$): δ = 0.83 (s, 9H), 0.85-0.89 (m, 3H), 1.08 (s, 3H), 1.19-1.29 (m, 2H), 1.31-1.44 (m, 2H), 1.96 (d, J = 14.4 Hz, 1H), 2.57 (d, J = 14.0 Hz, 1H), 3.11-3.23 (m, 2H), 3.38-3.44 (m, 1H), 6.64 (m, 2H), 6.93-7.01 (m, 5H), 7.05-7.13 (m, 5H), 7.30-7.37 (m, 6H), 7.66-7.68 (m, 2H) ppm. ^{13}C NMR (101 MHz, $CDCl_3$): δ = 14.0, 19.4, 22.4, 30.4, 31.2, 31.9, 45.8, 52.5, 56.3, 64.5, 68.5, 125.6, 125.7, 126.7, 127.2, 127.66, 127.7, 128.0, 128.7, 129.8, 138.9, 142.4, 148.9, 151.4, 177.3 ppm. HRMS (ESI): m/z calculated for [$C_{39}H_{45}NNaO_2$] [$M+Na^+$]: 582.3348, found: 582.3347.

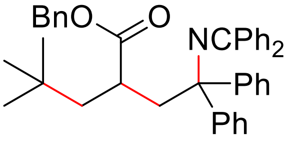

39

Benzyl 2-(2-((diphenylmethylene)amino)-2,2-diphenylethyl)-4,4-dimethylpentanoate (39)

Following the general procedure, benzophenone *O*-pivaloyl oxime **1a** (84.3 mg, 0.3 mmol), ethene-1,1-diyldibenzene **2a** (36.0 mg, 0.2 mmol), and benzyl acrylate **3e** (64.8 mg, 0.4 mmol) were used. Purification by column chromatography using pre-basified silica with pentane/EtOAc (400:1 to 200:1, v/v) as eluent afforded **39** as colorless oil (76 mg, 66% yield). ^{1}H NMR (400 MHz, $CDCl_3$): δ = 0.58 (s, 9H), 1.12 (dd, *J* = 14.0 Hz, 3.6 Hz, 1H), 1.58-1.63 (m, 1H), 2.20-2.24 (m, 1H), 2.47-2.53 (m, 1H), 2.98-3.04 (m, 1H), 4.24-4.27 (m, 1H), 4.56-4.59 (m, 1H), 6.61 (m, 2H), 6.84-6.92 (m, 7H), 6.98-7.03 (m, 1H), 7.08-7.14 (m, 3H), 7.16-7.30 (m, 8H), 7.40-7.62 (m, 4H) ppm. ^{13}C NMR (101 MHz, $CDCl_3$): δ = 29.3, 31.0, 37.7, 46.9, 49.6, 66.1, 69.2, 125.8, 126.0, 126.5, 127.2, 127.3, 127.5, 127.79, 127.82, 127.95, 128.00, 128.2, 128.4, 128.7, 129.9, 136.2, 138.9, 142.2, 148.6, 149.8, 167.6, 177.5 ppm. HRMS (ESI): m/z calculated for [$C_{41}H_{41}NNaO_2$] [M+Na$^+$]: 602.3035, found: 602.3027.

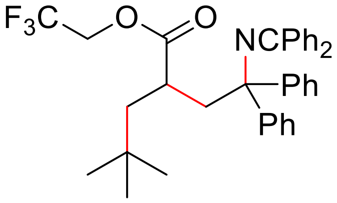

40

2,2,2-Trifluoroethyl 2-(2-((diphenylmethylene)amino)-2,2-diphenylethyl)-4,4-dimethylpentanoate (40)

Following the general procedure, benzophenone *O*-pivaloyl oxime **1a** (84.3 mg, 0.3 mmol), ethene-1,1-diyldibenzene **2a** (36.0 mg, 0.2 mmol), and 2,2,2-trifluoroethyl acrylate **3f** (61.6 mg, 0.4 mmol) were used. Purification by column chromatography using pre-basified silica with pentane/EtOAc (400:1 to 200:1, v/v) as eluent afforded **40** as colorless oil (80 mg, 70% yield). ^{1}H NMR (400 MHz, $CDCl_3$): δ = 0.65 (s, 9H),

1.19-1.28 (m, 1H), 1.65 (dd, J = 14.2, 8.2 Hz, 1H), 2.29 (dd, J = 13.4, 2.5 Hz, 1H), 2.48-2.59 (m, 1H), 3.06 (dd, J = 13.3, 10.7 Hz, 1H), 3.37 (dq, J = 12.7, 8.7 Hz, 1H), 3.91 (dq, J = 12.7, 8.6 Hz, 1H), 6.67 (s, 2H), 6.89-7.02 (m, 6H), 7.07 (tt, J = 7.4, 1.3 Hz, 1H), 7.19 (qt, J = 7.2, 1.5 Hz, 2H), 7.26-7.41 (m, 5H), 7.44-7.71 (m, 4H) ppm. ^{13}C NMR (101 MHz, $CDCl_3$): δ = 29.2, 31.1, 37.3, 47.3, 49.6, 60.6 (q, J = 36.0 Hz), 69.3, 125.9, 126.2, 126.7, 127.2, 127.3, 127.6, 127.9, 128.1, 128.6, 130.1, 138.8, 142.2, 148.1, 149.5, 168.1, 176.2 ppm. HRMS (ESI): m/z calculated for [$C_{36}H_{36}F_3NNaO_2$] [M+Na$^+$]: 594.2596, found: 594.2590.

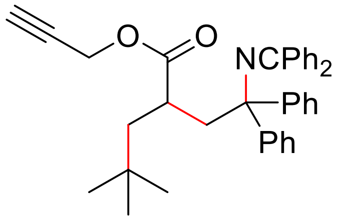

41

Prop-2-yn-1-yl 2-(2-((diphenylmethylene)amino)-2,2-diphenylethyl)-4,4-dimethylpentanoate (41)

Following the general procedure, benzophenone *O*-pivaloyl oxime **1a** (84.3 mg, 0.3 mmol), ethene-1,1-diyldibenzene **2a** (36.0 mg, 0.2 mmol), and prop-2-yn-1-yl acrylate **3g** (44.0 mg, 0.4 mmol) were used. Purification by column chromatography using pre-basified silica with pentane/EtOAc (400:1 to 200:1, v/v) as eluent afforded **41** as colorless oil (63 mg, 60% yield). ^{1}H NMR (400 MHz, $CDCl_3$): δ = 0.68 (s, 9H), 1.17-1.21 (m, 1H), 1.65-1.71 (m, 1H), 2.27-2.31 (m, 1H), 2.36 (t, J = 2.4 Hz, 1H), 2.50-2.56 (m, 1H), 3.02-3.08 (m, 1H), 3.82-3.87 (m, 1H), 4.07-4.12 (m, 1H), 6.69 (m, 2H), 6.92-7.00 (m, 7H), 7.06-7.10 (m, 1H), 7.15-7.20 (m, 1H), 7.24-7.40 (m, 5H), 7.48-7.64 (m, 2H), 7.63-7.69 (m, 2H) ppm. ^{13}C NMR (101 MHz, $CDCl_3$): δ = 29.4, 31.1, 37.5, 47.0, 49.6, 51.8, 69.2, 74.5, 78.1, 125.8, 126.1, 126.6, 127.26, 127.32, 127.5, 127.9, 128.0, 128.2, 128.4, 128.6, 130.0, 138.8, 142.0, 148.4, 149.8, 167.6, 176.8 ppm. HRMS (ESI): m/z calculated for [$C_{37}H_{37}NNaO_2$] [M+Na$^+$]: 550.2722, found: 550.2716.

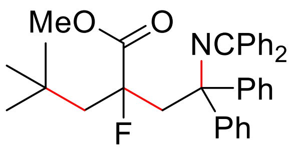

42

Methyl 2-(2-((diphenylmethylene)amino)-2,2-diphenylethyl)-2-fluoro-4,4-dimethylpentanoate (42)

Following the general procedure, benzophenone *O*-pivaloyl oxime **1a** (84.3 mg, 0.3 mmol), ethene-1,1-diyldibenzene **2a** (36.0 mg, 0.2 mmol), and methyl 2-fluoroacrylate **3h** (41.6 mg, 0.4 mmol) were used. Purification by column chromatography using pre-basified silica with pentane/EtOAc (400:1 to 200:1, v/v) as eluent afforded **42** as colorless oil (75 mg, 72% yield). ^{1}H NMR (400 MHz, $CDCl_3$): δ = 0.86 (s, 9H), 1.73-1.78 (m, 1H), 1.84-1.97 (m, 1H), 2.88-2.93 (m, 1H), 3.04-3.15 (m, 1H), 3.17 (s, 3H), 6.60-6.62 (m, 2H), 7.02-7.12 (m, 5H), 7.13-7.21 (m, 4H), 7.27-7.41 (m, 7H), 7.71-7.74 (m, 2H) ppm. ^{13}C NMR (101 MHz, $CDCl_3$): δ = 30.3, 30.4, 31.2, 48.4, 48.6, 52.0, 52.8, 53.0, 66.9, 94.9, 96.8, 125.91, 125.94, 127.0, 127.3, 127.5, 127.7, 127.8, 128.0, 128.5, 128.8, 129.9, 139.1, 142.5, 148.5, 150.8, 167.1, 171.9, 172.1 ppm. HRMS (ESI): m/z calculated for [$C_{35}H_{36}FNNaO_2$] [$M+Na^+$]: 544.2628, found: 544.2625.

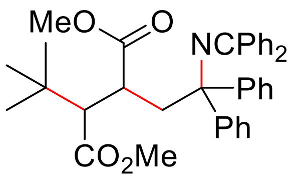

43, 50:50 d.r.

Dimethyl 2-(*tert*-butyl)-3-(2-((diphenylmethylene)amino)-2,2-diphenylethyl)succinate (43)

Following the general procedure, benzophenone *O*-pivaloyl oxime **1a** (84.3 mg, 0.3 mmol), ethene-1,1-diyldibenzene **2a** (36.0 mg, 0.2 mmol), and dimethyl maleate **3i** (57.6 mg, 0.4 mmol) were used. Purification by column chromatography using pre-basified silica with pentane/EtOAc (200:1 to 100:1, v/v) as eluent afforded **43** as colorless oil in 50:50 diastereomeric ratio (57 mg, 51% yield). Combined NMR data for both the isomers are reported here. ^{1}H NMR (400 MHz, $CDCl_3$): δ = 0.75 (s, 9H),

2.34-2.37 (m, 1H), 2.47-2.49 (m, 1H), 2.68-2.78 (m, 1H), 3.01-3.07 (m, 4H), 3.58 (s, 3H), 6.75-6.77 (m, 3H), 6.81-7.06 (m, 7H), 7.13-7.39 (m, 8H), 7.62-7.65 (m, 2H) ppm. ^{13}C NMR (101 MHz, $CDCl_3$): δ = 28.1, 33.4, 40.0, 43.2, 51.2, 51.7, 59.0, 69.4, 125.8, 126.1, 126.4, 127.23, 127.25, 127.4, 127.6, 127.9, 128.5, 128.8, 129.9, 139.0, 142.4, 148.2, 149.3, 167.9, 173.3, 175.6 ppm. HRMS (ESI): m/z calculated for [$C_{37}H_{39}NNaO_4$] [M+Na$^+$]: 584.2777, found: 584.2777.

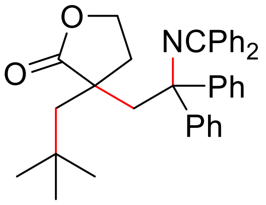

44

3-(2-((Diphenylmethylene)amino)-2,2-diphenylethyl)-3-neopentyldihydrofuran-2(3*H*)-one (44)

Following the general procedure, benzophenone *O*-pivaloyl oxime **1a** (84.3 mg, 0.3 mmol), ethene-1,1-diyldibenzene **2a** (36.0 mg, 0.2 mmol), and 3-methylenedihydrofuran-2(3*H*)-one **3j** (39.2 mg, 0.4 mmol) were used. Purification by column chromatography using pre-basified silica with pentane/EtOAc (200:1 to 100:1, v/v) as eluent afforded **44** as colorless oil (44 mg, 43% yield). ^{1}H NMR (400 MHz, $CDCl_3$): δ = 0.96 (s, 9H), 1.61-1.64 (m, 1H), 1.72-1.85 (m, 2H), 2.05-2.12 (m, 1H), 2.94-2.98 (m, 1H), 3.08-3.12 (m, 1H), 3.98-4.05 (m, 1H), 4.12-4.17 (m, 1H), 6.74 (m, 2H), 6.93-6.99 (m, 5H), 7.03-7.07 (m, 2H), 7.10-7.17 (m, 1H), 7.18-7.23 (m, 3H), 7.30-7.40 (m, 5H), 7.62-7.64 (m, 2H) ppm. ^{13}C NMR (101 MHz, $CDCl_3$): δ = 30.2, 31.5, 32.0, 45.5, 48.2, 53.0, 65.5, 68.6, 125.9, 126.1, 126.7, 127.3, 127.6, 127.7, 128.0, 128.1, 128.4, 128.6, 130.1, 130.2, 138.5, 141.7, 148.2, 150.3, 168.0, 182.0 ppm. HRMS (ESI): m/z calculated for [$C_{36}H_{37}NNaO_2$] [M+Na$^+$]: 538.2722, found: 538.2722.

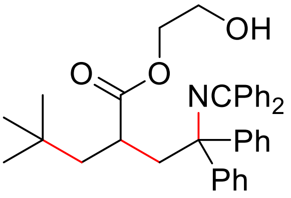

45

2-Hydroxyethyl 2-(2-((diphenylmethylene)amino)-2,2-diphenylethyl)-4,4-dimethylpentanoate (45)

Following the general procedure, benzophenone *O*-pivaloyl oxime **1a** (84.3 mg, 0.3 mmol), ethene-1,1-diyldibenzene **2a** (36.0 mg, 0.2 mmol), and 2-hydroxyethyl acrylate **3b** (46.4 mg, 0.4 mmol) were used. Purification by column chromatography using pre-basified silica with pentane/EtOAc (20:1 to 10:1, v/v) as eluent afforded **45** as colorless oil (72 mg, 68% yield). ^{1}H NMR (400 MHz, $CDCl_3$): δ = 0.67 (s, 9H), 1.20 (dd, *J* = 14.0 Hz, 3.2 Hz, 1H), 1.66-1.76 (m, 2H), 2.30-2.34 (m, 1H), 2.52-2.58 (m, 1H), 3.03-3.09 (m, 1H), 3.40-3.45 (m, 1H), 3.48-3.60 (m, 2H), 3.66-3.72 (m, 1H), 6.68 (m, 2H), 6.92-7.00 (m, 7H), 7.06-7.10 (m, 1H), 7.15-7.20 (m, 1H), 7.24-7.29 (m, 2H), 7.31-7.40 (m, 3H), 7.54-7.63 (m, 2H), 7.65-7.68 (m, 2H) ppm. ^{13}C NMR (101 MHz, $CDCl_3$): δ = 29.3, 31.0, 37.5, 47.0, 49.8, 61.1, 66.0, 69.2, 125.8, 126.1, 126.6, 127.2, 127.3, 127.5, 127.8, 128.0, 128.1, 128.3, 128.6, 130.0, 138.8, 142.3, 148.4, 149.6, 167.8, 178.3 ppm. HRMS (ESI): m/z calculated for [$C_{36}H_{39}NNaO_3$] [M+Na$^+$]: 556.2828, found: 556.2822.

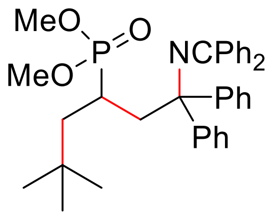

46

Dimethyl (1-((diphenylmethylene)amino)-5,5-dimethyl-1,1-diphenylhexan-3-yl)phosphonate (46)

Following the general procedure, benzophenone *O*-pivaloyl oxime **1a** (84.3 mg, 0.3 mmol), ethene-1,1-diyldibenzene **2a** (36.0 mg, 0.2 mmol), and dimethyl vinylphosphonate **3k** (54.4 mg, 0.4 mmol) were used. Purification by column chromatography using pre-basified silica with pentane/EtOAc (6:1 to 2:1, v/v) as eluent afforded **46** as colorless oil (64 mg, 58% yield). ^{1}H NMR (400 MHz, $CDCl_3$): δ = 0.69 (s, 9H), 1.46-1.72 (m, 2H), 2.10-2.21 (m, 1H), 2.43-2.51 (m, 1H), 3.08-3.17 (m, 1H), 3.48-3.54 (m, 6H), 6.64-6.66 (m, 2H), 6.96-7.00 (m, 2H), 7.02-7.18 (m, 9H), 7.25-7.38 (m, 5H), 7.66-7.68 (m, 2H) ppm. ^{13}C NMR (101 MHz, $CDCl_3$): δ = 28.0, 29.4, 30.0, 30.6 (d, *J* = 2.0 Hz), 43.2 (d, *J* = 2.0 Hz), 44.3 (d, *J* = 4.0 Hz), 52.1 (d, *J* = 7.0 Hz), 52.3

(d, *J* = 6.0 Hz), 69.6 (d, *J* = 11.0 Hz), 125.94, 125.96, 126.5, 127.25, 127.32, 127.6, 127.7, 127.9, 128.6, 128.7, 129.9, 139.0, 142.2, 148.8, 149.1, 167.2 ppm. HRMS (ESI): m/z calculated for [$C_{35}H_{40}NNaO_3P$] [M+Na$^+$]: 576.2644, found: 576.2647.

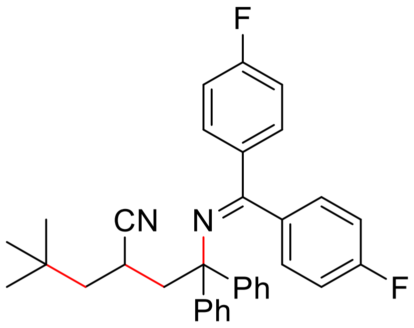

47

2-(2-((Bis(4-fluorophenyl)methylene)amino)-2,2-diphenylethyl)-4,4-dimethylpentanenitrile (47)

Following the general procedure, oxime ester **1al** (95.2 mg, 0.3 mmol), ethene-1,1-diyldibenzene **2a** (36.0 mg, 0.2 mmol), and acrylonitrile **3a** (21.2 mg, 0.4 mmol) were used. Purification by column chromatography using pre-basified silica with pentane/EtOAc (500:1 to 200:1, v/v) as eluent afforded **47** as a white solid (46 mg, 45% yield). ^{1}H NMR (400 MHz, $CDCl_3$): δ = 0.72 (s, 9H), 1.09 (dd, *J* = 13.9, 2.9 Hz, 1H), 1.69 (dd, *J* = 13.9, 10.3 Hz, 1H), 2.29 (dd, *J* = 13.6, 3.3 Hz, 1H), 2.54 (ddt, *J* = 10.2, 8.4, 3.1 Hz, 1H), 2.93 (dd, *J* = 13.6, 8.5 Hz, 1H), 6.55 (s, 2H), 6.71 (t, *J* = 8.6 Hz, 2H), 6.91-6.98 (m, 2H), 6.99-7.11 (m, 5H), 7.17-7.23 (m, 1H), 7.26-7.32 (m, 2H), 7.42-7.57 (m, 2H), 7.64-7.71 (m, 2H) ppm. $^{13}C\{^1H,^{19}F\}$ NMR (126 MHz, $CDCl_3$): δ = 22.3, 29.2, 31.0, 46.3, 48.7, 68.9, 114.6, 115.0, 115.7, 124.6, 126.6, 126.7, 128.0, 128.1, 128.3, 129.0, 130.8, 132.6, 134.3, 137.7, 147.9, 148.0, 161.7, 164.4, 166.9 ppm. HRMS (ESI): m/z calculated for [$C_{34}H_{32}N_2F_2Na$] [M+Na$^+$]: 529.2426, found: 529.2421.

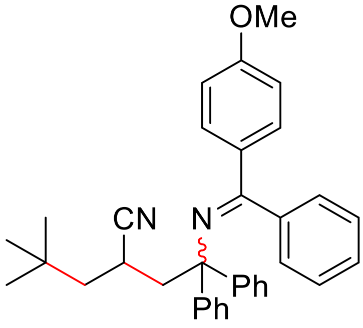

48, 70:30 *E*/*Z*

2-(2-(((4-Methoxyphenyl)(phenyl)methylene)amino)-2,2-diphenylethyl)-4,4-dimethylpentanenitrile (48)

Following the general procedure, oxime ester **1am** (93.4 mg, 0.3 mmol), ethene-1,1-diyldibenzene **2a** (36.0 mg, 0.2 mmol), and acrylonitrile **3a** (21.2 mg, 0.4 mmol) were used. Purification by column chromatography using pre-basified silica with pentane/EtOAc (500:1 to 25:1, v/v) as eluent afforded **48** as a white solid (41 mg, 41% yield) with an *E*/*Z*-ratio of 70:30. Combined NMR data for both the isomers are reported here. ^{1}H NMR (400 MHz, $CDCl_3$): δ = 0.69-0.76 (m, 9H), 1.06-1.17 (m, 1H), 1.58-1.71 (m, 1H), 2.28-2.38 (m, 1H), 2.52-2.63 (m, 1H), 2.91-3.02 (m, 1H), 3.76-3.84 (m, 3H), 6.37-6.69 (m, 2H), 6.81-7.20 (m, 9H), 7.20-7.29 (m, 2H), 7.32-7.40 (m, 1H), 7.48 (s, 2H), 7.62-7.75 (m, 2H) ppm. ^{13}C NMR (101 MHz, $CDCl_3$): δ = 22.4, 22.4, 29.3, 31.0, 31.0, 45.8, 46.2, 48.4, 55.3, 55.4, 68.4, 68.6, 112.9, 113.3, 124.5, 124.6, 126.3, 126.4, 126.5, 126.8, 127.2, 127.4, 127.9, 128.0, 128.0, 128.1, 128.2, 128.6, 128.8, 130.2, 130.4, 130.9, 134.6, 138.8, 142.0, 148.2, 148.3, 148.4, 148.5, 158.4, 161.4, 168.0, 169.0 ppm. HRMS (ESI): m/z calculated for [$C_{35}H_{36}N_2ONa$] [M+Na$^+$]: 523.2720, found: 523.2717.

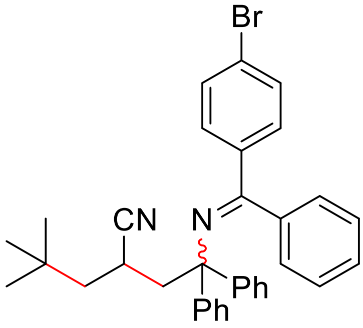

49, 52:48 E/*Z*

2-(2-(((4-bromophenyl)(phenyl)methylene)amino)-2,2-diphenylethyl)-4,4-dimethylpentanenitrile (49)

Following the general procedure, oxime ester **1an** (108 mg, 0.3 mmol), ethene-1,1-diyldibenzene **2a** (36.0 mg, 0.2 mmol), and acrylonitrile **3a** (21.2 mg, 0.4 mmol) were used. Purification by column chromatography using pre-basified silica with pentane/EtOAc (500:1 to 150:1, v/v) as eluent afforded **49** as a white solid (56 mg, 51% yield) with an *E*/*Z*-ratio of 52:48. Combined NMR data for both the isomers are reported here. ^{1}H NMR (400 MHz, $CDCl_3$): δ = 0.73 (s, 9H), 1.05-1.14 (m, 1H), 1.62-1.74 (m, 1H), 2.25-2.39 (m, 1H), 2.47-2.63 (m, 1H), 2.85-3.09 (m, 1H), 6.32-6.73 (m, 2H), 6.91-7.23 (m, 8H), 7.23-7.31 (m, 2H), 7.32-7.62 (m, 6H), 7.66-7.72 (m, 1H) ppm. ^{13}C NMR (101 MHz, $CDCl_3$): δ = 22.3, 22.3, 29.2, 31.0, 31.0, 46.0, 46.4, 48.6, 68.8, 68.9, 121.3, 124.5, 124.6, 125.1, 126.5, 126.6, 126.7, 126.7, 127.1, 127.6, 128.0, 128.0, 128.1, 128.1, 128.2, 128.2, 128.6, 128.7, 128.9, 130.1, 130.3, 130.5, 130.6, 131.2, 131.7, 131.8, 137.6, 138.1, 140.5, 141.0, 147.8, 147.9, 147.9, 148.2, 167.7, 168.0 ppm. HRMS (ESI): m/z calculated for [$C_{34}H_{33}N_2BrNa$] [$M+Na^+$]: 571.1719, found: 571.1720.

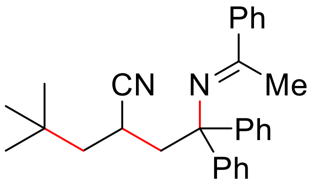

50, >95:5 *E*/*Z*

(*E*)-2-(2,2-Diphenyl-2-((1-phenylethylidene)amino)ethyl)-4,4-dimethylpentanenitrile (50)

Following the general procedure, (*E*)-1-phenylethan-1-one *O*-pivaloyl oxime **1ao** (65.8 mg, 0.3 mmol), ethene-1,1-diyldibenzene **2a** (36.0 mg, 0.2 mmol), and acrylonitrile **3a** (21.2 mg, 0.4 mmol) were used. Purification by column chromatography using pre-basified silica with pentane/EtOAc (500:1 to 50:1, v/v) as eluent afforded **50** as a colorless oil (26 mg, 32% yield) in >95:5 *E*/*Z* ratio. ^{1}H NMR (400 MHz, $CDCl_3$): δ = 0.73 (s, 9H), 1.19 (dd, *J* = 13.9, 2.8 Hz, 1H), 1.62-1.74 (m, 1H), 1.83 (s, 3H), 2.51-2.64 (m, 2H), 3.04-3.14 (m, 1H), 7.17-7.25 (m, 2H), 7.28-7.36 (m, 4H), 7.37-7.48 (m, 5H), 7.52-7.60 (m, 2H), 7.94-8.03 (m, 2H) ppm. ^{13}C NMR (101 MHz, $CDCl_3$): δ = 20.8, 22.6, 29.3, 31.0, 47.1, 48.7, 68.6, 124.6, 126.7, 126.8, 127.2,

127.5, 127.8, 128.3, 128.5, 128.6, 130.0, 142.1, 146.9, 147.1, 167.9 ppm. HRMS (ESI): m/z calculated for [$C_{29}H_{32}N_2Na$] [M+Na$^+$]: 431.2458, found: 431.2457.

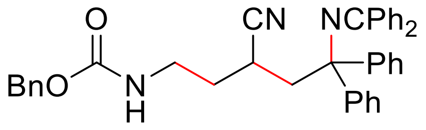

51

Benzyl (3-cyano-5-((diphenylmethylene)amino)-5,5-diphenylpentyl)carbamate (51)

Following the general procedure, benzyl (2-(((diphenylmethylene)amino)oxy)-2-oxoethyl)carbamate **1s** (116.4 mg, 0.3 mmol), ethene-1,1-diyldibenzene **2a** (36.0 mg, 0.2 mmol), and acrylonitrile **3a** (21.2 mg, 0.4 mmol) were used. Purification by column chromatography using pre-basified silica with pentane/EtOAc (10:1 to 8:1, v/v) as eluent afforded **51** as a white solid (69 mg, 60% yield). ^{1}H NMR (400 MHz, $CDCl_3$): δ = 1.60-1.74 (m, 2H), 2.35-2.39 (m, 1H), 2.66-2.73 (m, 1H), 2.89-2.95 (m, 1H), 3.06-3.22 (m, 2H), 4.22-4.57 (m, 1H), 5.02-5.10 (m, 2H), 6.55-6.57 (m, 2H), 7.00-7.19 (m, 9H), 7.21-7.27 (m, 2H), 7.30-7.42 (m, 8H), 7.46-7.51 (m, 2H), 7.71-7.74 (m, 2H) ppm. ^{13}C NMR (101 MHz, $CDCl_3$): δ = 24.0, 33.9, 38.4, 43.3, 66.8, 68.4, 122.2, 126.57, 126.65, 127.0, 127.1, 127.5, 127.98, 128.04, 128.08, 128.11, 128.22, 128.27, 128.6, 128.7, 130.3, 136.6, 138.4, 141.4, 147.6, 148.2, 156.2, 169.1 ppm. HRMS (ESI): m/z calculated for [$C_{39}H_{35}N_3NaO_2$] [M+Na$^+$]: 600.2627, found: 600.2615.

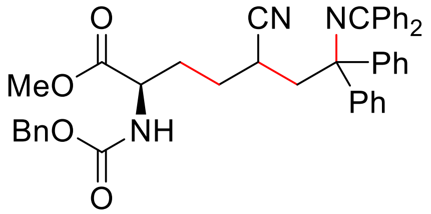

52, 50:50 d.r.

(2*R*)-Methyl 2-(((benzyloxy)carbonyl)amino)-5-cyano-7-((diphenylmethylene)amino)-7,7-diphenylheptanoate (52)

Following the general procedure, (*R*)-methyl 2-(((benzyloxy)carbonyl)amino)-4-(((diphenylmethylene)amino)oxy)-4-oxobutanoate **1t** (138.0 mg, 0.3 mmol), ethene-1,1-diyldibenzene **2a** (36.0 mg, 0.2 mmol), and acrylonitrile **3a** (21.2 mg, 0.4 mmol) were used. Purification by column chromatography using pre-basified silica with

pentane/EtOAc (6:1 to 4:1, v/v) as eluent afforded **52** as a white solid in 50:50 diastereomeric ratio (83 mg, 64% yield). Combined NMR data for both the isomers are reported here. ^{1}H NMR (400 MHz, $CDCl_3$): δ = 1.30-1.62 (m, 2H), 1.65-2.00 (m, 2H), 2.31-2.36 (m, 1H), 2.59-2.66 (m, 1H), 2.84-2.92 (m, 1H), 3.69-3.75 (m, 3H), 4.23-4.38 (m, 1H), 5.09-5.16 (m, 3H), 6.52-6.54 (m, 2H), 7.00-7.12 (m, 7H), 7.13-7.20 (m, 2H), 7.21-7.25 (m, 2H), 7.30-7.45 (m, 10H), 7.69-7.72 (m, 2H) ppm. ^{13}C NMR (101 MHz, $CDCl_3$): δ = 26.6, 26.7, 29.9, 30.2, 43.6, 52.60, 52.63, 53.4, 67.2, 68.27, 68.30, 122.0, 122.1, 126.57, 126.63, 127.07, 127.14, 127.49, 127.54, 127.90, 127.92, 127.97, 128.04, 128.09, 128.22, 128.25, 128.4, 128.66, 128.67, 128.70, 129.0, 129.4, 130.4, 136.2, 138.41, 138.42, 141.43, 141.44, 147.66, 147.70, 148.09, 148.17, 155.9, 168.9, 172.3 ppm. HRMS (ESI): m/z calculated for [$C_{42}H_{39}N_3NaO_4$] [M+Na$^+$]: 672.2838, found: 672.2839.

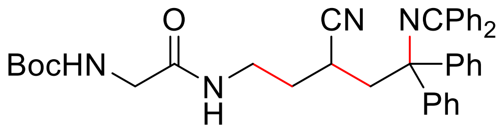

53

***tert*-Butyl (2-((3-cyano-5-((diphenylmethylene)amino)-5,5-diphenylpentyl)amino)-2-oxoethyl)carbamate (53)**

Following the general procedure, *tert*-butyl (2-((2-(((diphenylmethylene)amino)oxy)-2-oxoethyl)amino)-2-oxoethyl)carbamate **1u** (123.3 mg, 0.3 mmol), ethene-1,1-diyldibenzene **2a** (36.0 mg, 0.2 mmol), and acrylonitrile **3a** (21.2 mg, 0.4 mmol) were used. Purification by column chromatography using pre-basified silica with pentane/EtOAc (2:1 to 1:1, v/v) as eluent afforded **53** as a white solid (61 mg, 51% yield). ^{1}H NMR (400 MHz, $CDCl_3$): δ = 1.45-1.46 (m, 9H), 1.62-1.74 (m, 2H), 2.34-2.38 (m, 1H), 2.59-2.66 (m, 1H), 2.87-2.93 (m, 1H), 3.08-3.29 (m, 2H), 3.62-3.63 (m, 2H), 5.01 (s, 1H), 5.95-5.97 (m, 1H), 6.53-6.55 (m, 2H), 6.99-7.14 (m, 7H), 7.17-7.30 (m, 4H), 7.32-7.47 (m, 5H), 7.68-7.71 (m, 2H) ppm. ^{13}C NMR (101 MHz, $CDCl_3$): δ = 24.2, 28.4, 33.4, 36.9, 43.2, 44.3, 68.4, 80.4, 122.4, 126.58, 126.61, 127.06, 127.11, 127.5, 128.02, 128.06, 128.09, 128.3, 128.7, 130.4, 138.4, 141.4, 147.6, 148.4, 169.1, 169.6 ppm. HRMS (ESI): m/z calculated for [$C_{38}H_{40}N_4NaO_3$] [M+Na$^+$]: 623.2998,

found: 623.3009.

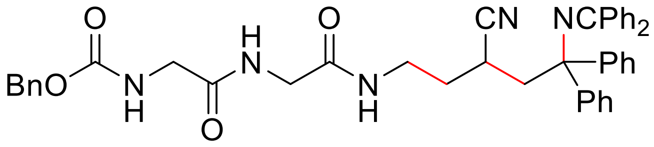

54

Benzyl (2-((2-((3-cyano-5-((diphenylmethylene)amino)-5,5-diphenylpentyl)amino)-2-oxoethyl)amino)-2-oxoethyl)carbamate (54)

Following the general procedure, benzyl (4,7,10-trioxo-1,1-diphenyl-3-oxa-2,6,9-triazaundec-1-en-11-yl)carbamate **1v** (150.6 mg, 0.3 mmol), ethene-1,1-diyldibenzene **2a** (36.0 mg, 0.2 mmol), and acrylonitrile **3a** (21.2 mg, 0.4 mmol) were used. Purification by column chromatography using pre-basified silica with pentane/EtOAc (1:1, v/v) as eluent afforded **54** as a white solid (91 mg, 66% yield). ^{1}H NMR (400 MHz, $CDCl_3$): δ = 1.56-1.70 (m, 2H), 2.32-2.36 (m, 1H), 2.60-2.66 (m, 1H), 2.86-2.91 (m, 1H), 3.12-3.26 (m, 2H), 3.75-3.78 (m, 4H), 5.07-5.09 (m, 2H), 5.72 (t, *J* = 5.6 Hz, 1H), 6.48-6.53 (m, 2H), 6.85 (t, *J* = 5.6 Hz, 1H), 6.98-7.18 (m, 10H), 7.21-7.25 (m, 2H), 7.27-7.39 (m, 8H), 7.42-7.44 (m, 2H), 7.67-7.70 (m, 2H) ppm. ^{13}C NMR (101 MHz, $CDCl_3$): δ = 24.4, 33.2, 36.9, 43.0, 43.3, 44.7, 67.4, 68.4, 122.6, 126.56, 126.60, 127.1, 127.5, 127.96, 127.99, 128.04, 128.07, 128.2, 128.3, 128.4, 128.67, 128.72, 130.4, 136.2, 138.4, 141.4, 147.5, 148.2, 157.0, 168.8, 169.1, 169.8 ppm. HRMS (ESI): m/z calculated for [$C_{43}H_{41}N_5NaO_4$] [M+Na$^+$]: 714.3056, found: 714.3048.

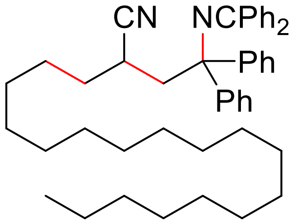

55

2-(2-((Diphenylmethylene)amino)-2,2-diphenylethyl)icosanenitrile (55)

Following the general procedure, benzophenone *O*-stearoyl oxime **1w** (138.9 mg, 0.3 mmol), ethene-1,1-diyldibenzene **2a** (36.0 mg, 0.2 mmol), and acrylonitrile **3a** (21.2 mg, 0.4 mmol) were used. Purification by column chromatography using pre-basified silica with pentane/EtOAc (400:1 to 200:1, v/v) as eluent afforded **55** as a white solid

(74 mg, 57% yield). ^{1}H NMR (400 MHz, $CDCl_3$): δ = 0.90 (t, J = 7.2 Hz, 3H), 1.11-1.48 (m, 35H), 2.34-2.39 (m, 1H), 2.57-2.63 (m, 1H), 2.86-2.92 (m, 1H), 6.56-6.58 (m, 2H), 7.01-7.20 (m, 9H), 7.22-7.27 (m, 2H), 7.33-7.42 (m, 3H), 7.43-7.53 (m, 2H), 7.71-7.74 (m, 2H) ppm. ^{13}C NMR (101 MHz, $CDCl_3$): δ = 14.3, 22.8, 26.6, 26.8, 29.0, 29.4, 29.5, 29.6, 29.7, 29.78, 29.79, 29.82, 29.84, 32.1, 34.0, 43.6, 68.4, 122.9, 126.46, 126.48, 127.0, 127.3, 127.4, 127.98, 128.02, 128.06, 128.2, 128.7, 129.1, 130.2, 138.6, 141.6, 148.1, 148.5, 168.7 ppm. HRMS (ESI): m/z calculated for [$C_{47}H_{60}N_2Na$] [$M+Na^+$]: 675.4654, found: 675.4651.

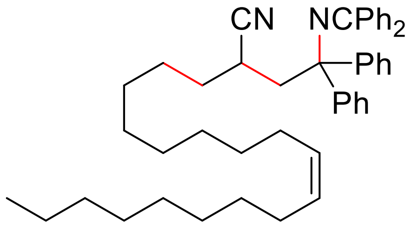

56

(*Z*)-2-(2-((Diphenylmethylene)amino)-2,2-diphenylethyl)icos-11-enenitrile (56)

Following the general procedure, (*Z*)-benzophenone *O*-oleoyl oxime **1x** (138.3 mg, 0.3 mmol), ethene-1,1-diyldibenzene **2a** (36.0 mg, 0.2 mmol), and acrylonitrile **3a** (21.2 mg, 0.4 mmol) were used. Purification by column chromatography using pre-basified silica with pentane/EtOAc (400:1 to 200:1, v/v) as eluent afforded **56** as a white solid (78 mg, 60% yield). ^{1}H NMR (400 MHz, $CDCl_3$): δ = 0.87-0.90 (m, 3H), 1.06-1.46 (m, 26H), 1.99-2.05 (m, 4H), 2.33-2.37 (m, 1H), 2.55-2.61 (m, 1H), 2.85-2.90 (m, 1H), 5.30-5.40 (m, 2H), 6.54-6.56 (m, 2H), 7.00-7.18 (m, 9H), 7.22-7.26 (m, 2H), 7.32-7.40 (m, 3H), 7.42-7.52 (m, 2H), 7.70-7.72 (m, 2H) ppm. ^{13}C NMR (101 MHz, $CDCl_3$): δ = 14.3, 22.8, 26.7, 26.8, 27.35, 27.37, 29.0, 29.34, 29.37, 29.46, 29.52, 29.7, 29.88, 29.91, 32.0, 34.0, 43.6, 68.4, 122.9, 126.47, 126.49, 127.0, 127.3, 127.5, 127.99, 128.03, 128.07, 128.2, 128.7, 130.0, 130.1, 130.3, 132.4, 138.6, 141.6, 148.1, 148.5, 168.7 ppm. HRMS (ESI): m/z calculated for [$C_{47}H_{58}N_2Na$] [$M+Na^+$]: 673.4498, found: 673.4505.

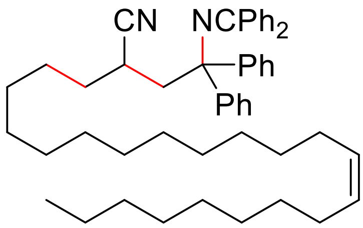

57

(*Z*)-2-(2-((Diphenylmethylene)amino)-2,2-diphenylethyl)tetracos-15-enenitrile (57)

Following the general procedure, (*Z*)-benzophenone *O*-docos-13-enoyl oxime **1y** (155.1 mg, 0.3 mmol), ethene-1,1-diyldibenzene **2a** (36.0 mg, 0.2 mmol), and acrylonitrile **3a** (21.2 mg, 0.4 mmol) were used. Purification by column chromatography using pre-basified silica with pentane/EtOAc (400:1 to 200:1, v/v) as eluent afforded **57** as a white solid (79 mg, 56% yield). ^{1}H NMR (400 MHz, $CDCl_3$): δ = 0.89 (t, *J* = 6.8 Hz, 3H), 1.07-1.46 (m, 34H), 2.00-2.05 (m, 4H), 2.33-2.37 (m, 1H), 2.55-2.61 (m, 1H), 2.84-2.90 (m, 1H), 5.32-5.50 (m, 2H), 6.54-6.56 (m, 2H), 6.99-7.18 (m, 9H), 7.21-7.25 (m, 2H), 7.32-7.40 (m, 3H), 7.41-7.47 (m, 2H), 7.69-7.72 (m, 2H) ppm. ^{13}C NMR (101 MHz, $CDCl_3$): δ = 14.3, 22.8, 26.7, 26.8, 27.4, 29.0, 29.38, 29.47, 29.48, 29.63, 29.67, 29.72, 29.74, 29.79, 29.92, 29.94, 32.0, 34.1, 43.6, 68.4, 122.9, 126.48, 126.49, 127.0, 127.3, 127.5, 128.00, 128.03, 128.07, 128.2, 128.7, 130.04, 130.06, 130.3, 138.6, 141.6, 148.1, 148.5, 168.7 ppm. HRMS (ESI): m/z calculated for [$C_{51}H_{66}N_2Na$] [M+Na^+]: 729.5124, found: 729.5118.

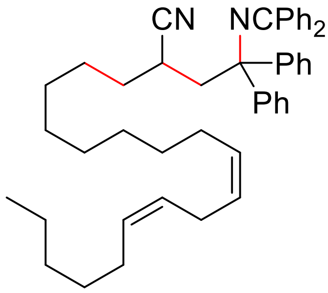

58

(11*Z*,14*Z*)-2-(2-((diphenylmethylene)amino)-2,2-diphenylethyl)icosa-11,14-dienenitrile (58)

Following the general procedure, benzophenone *O*-((9*Z*,12*Z*)-octadeca-9,12-dienoyl) oxime **1z** (137.7 mg, 0.3 mmol), ethene-1,1-diyldibenzene **2a** (36.0 mg, 0.2 mmol), and

acrylonitrile **3a** (21.2 mg, 0.4 mmol) were used. Purification by column chromatography using pre-basified silica with pentane/EtOAc (400:1 to 200:1, v/v) as eluent afforded **58** as a white solid (78 mg, 60% yield). ^{1}H NMR (400 MHz, $CDCl_3$): δ = 0.88-0.92 (m, 3H), 1.06-1.46 (m, 20H), 2.03-2.09 (m, 4H), 2.33-2.38 (m, 1H), 2.55-2.62 (m, 1H), 2.76-2.81 (m, 2H), 2.85-2.90 (m, 1H), 5.31-5.44 (m, 4H), 6.55-6.57 (m, 2H), 7.00-7.12 (m, 7H), 7.13-7.19 (m, 2H), 7.22-7.26 (m, 2H), 7.32-7.41 (m, 3H), 7.41-7.51 (m, 2H), 7.70-7.72 (m, 2H) ppm. ^{13}C NMR (101 MHz, $CDCl_3$): δ = 14.2, 22.7, 25.8, 26.7, 26.8, 27.35, 27.37, 29.0, 29.33, 29.37, 29.49, 29.52, 29.8, 31.7, 34.0, 43.6, 68.4, 122.9, 126.47, 16.49, 127.0, 127.3, 127.5, 127.99, 128.02, 128.07, 128.15, 128.16, 128.7, 130.24, 130.27, 130.35, 138.6, 141.6, 148.1, 148.5, 168.7 ppm. HRMS (ESI): m/z calculated for [$C_{47}H_{56}N_2Na$] [$M+Na^+$]: 671.4341, found: 671.4341.

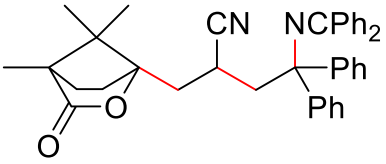

59, 50:50 d.r.

4-((Diphenylmethylene)amino)-4,4-diphenyl-2-((4,7,7-trimethyl-3-oxo-2-oxabicyclo[2.2.1]heptan-1-yl)methyl)butanenitrile (59)

Following the general procedure, 1-((((diphenylmethylene)amino)oxy)carbonyl)-4,7,7-trimethyl-2-oxabicyclo[2.2.1]heptan-3-one **1aa** (113.1 mg, 0.3 mmol), ethene-1,1-diyldibenzene **2a** (36.0 mg, 0.2 mmol), and acrylonitrile **3a** (21.2 mg, 0.4 mmol) were used. Purification by column chromatography using pre-basified silica with pentane/EtOAc (40:1 to 30:1, v/v) as eluent afforded **59** as a white solid in 50:50 diastereomeric ratio (76 mg, 67% yield). Combined NMR data for both the isomers are reported here. ^{1}H NMR (400 MHz, $CDCl_3$): δ = 0.67 (s, 3H), 0.73 (s, 3H), 1.02 (s, 3H), 1.39-1.45 (m, 2H), 1.55-1.73 (m, 2H), 1.76-1.87 (m, 2H), 2.64-2.68 (m, 1H), 2.87-2.95 (m, 2H), 6.56-6.58 (m, 2H), 6.99-7.02 (m, 2H), 7.04-7.10 (m, 5H), 7.12-7.16 (m, 1H), 7.21-7.28 (m, 3H), 7.29-7.43 (m, 3H), 7.42-7.53 (m, 2H), 7.71-7.74 (m, 2H) ppm. ^{13}C NMR (101 MHz, $CDCl_3$): δ = 10.0, 16.2, 16.4, 23.0, 28.9, 29.2, 33.5, 44.1, 52.3, 53.5, 68.5, 92.5, 123.3, 126.55, 126.59, 127.0, 127.2, 127.5, 127.8, 128.04, 128.09, 128.13, 128.40, 128.48, 128.8, 129.0, 129.5, 130.3, 138.6, 141.5, 147.7, 148.1, 169.0, 179.5

ppm. HRMS (ESI): m/z calculated for [$C_{39}H_{38}N_2NaO_2$] [M+Na$^+$]: 589.2831, found: 589.2833.

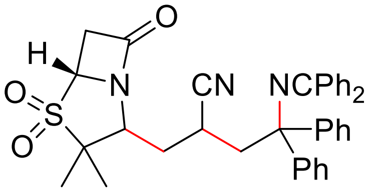

60, 50:50 d.r.

2-(((5*R*)-3,3-Dimethyl-4,4-dioxido-7-oxo-4-thia-1-azabicyclo[3.2.0]heptan-2-yl)methyl)-4-((diphenylmethylene)amino)-4,4-diphenylbutanenitrile (60)

Following the general procedure, (2*S*,5*R*)-2-((((diphenylmethylene)amino)oxy)carbonyl)-3,3-dimethyl-4-thia-1-azabicyclo[3.2.0]heptan-7-one 4,4-dioxide **1ae** (123.6 mg, 0.3 mmol), ethene-1,1-diyldibenzene **2a** (36.0 mg, 0.2 mmol), and acrylonitrile **3a** (21.2 mg, 0.4 mmol) were used. Purification by column chromatography using pre-basified silica with pentane/EtOAc (6:1 to 4:1, v/v) as eluent afforded **60** as a white solid in 50:50 diastereomeric ratio (67 mg, 56% yield). Combined NMR data for both the isomers are reported here. ^{1}H NMR (400 MHz, $CDCl_3$): δ = 1.24-1.32 (m, 1H), 1.61-1.71 (m, 6H), 2.01-2.16 (m, 2H), 2.32-2.43 (m, 1H), 2.55-2.67 (m, 1H), 2.93-3.07 (m, 2H), 3.75-3.81 (m, 1H), 5.46-5.52 (m, 1H), 6.52-6.54 (m, 2H), 6.98-7.03 (m, 3H), 7.05-7.14 (m, 3H), 7.16-7.28 (m, 3H), 7.29-7.48 (m, 7H), 7.53-7.69 (m, 2H) ppm. ^{13}C NMR (101 MHz, $CDCl_3$): δ = 18.7, 22.7, 23.9, 28.8, 37.7, 42.1, 44.6, 51.1, 68.6, 115.6, 120.7, 121.9, 126.7, 126.9, 127.09, 127.12, 127.48, 127.55, 128.0, 128.17, 128.20, 128.4, 128.6, 128.7, 129.0, 129.1, 129.19, 129.25, 129.27, 130.5, 138.4, 141.3, 147.1, 147.6, 164.2, 169.3 ppm. HRMS (ESI): m/z calculated for [$C_{37}H_{35}N_3NaO_3S$] [M+Na$^+$]: 624.2297, found: 624.2297.

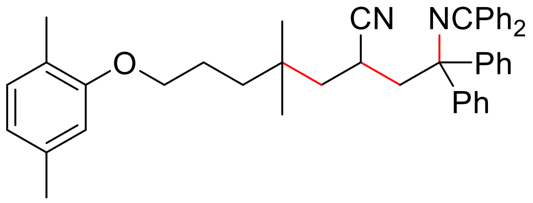

61

7-(2,5-Dimethylphenoxy)-2-(2-((diphenylmethylene)amino)-2,2-diphenylethyl)-4,4-dimethylheptanenitrile (61)

Following the general procedure, benzophenone *O*-(5-(2,5-dimethylphenoxy)-2,2-dimethylpentanoyl) oxime **1ac** (128.7 mg, 0.3 mmol), ethene-1,1-diyldibenzene **2a** (36.0 mg, 0.2 mmol), and acrylonitrile **3a** (21.2 mg, 0.4 mmol) were used. Purification by column chromatography using pre-basified silica with pentane/EtOAc (40:1 to 30:1, v/v) as eluent afforded **61** as a white solid (62 mg, 50% yield). ^{1}H NMR (400 MHz, $CDCl_3$): δ = 0.78-0.79 (m, 3H), 0.82-0.83 (m, 3H), 1.14-1.34 (m, 3H), 1.44-1.52 (m, 1H), 1.58-1.64 (m, 1H), 1.71-1.78 (m, 1H), 2.18-2.20 (m, 3H), 2.34-2.41 (m, 4H), 2.63-2.68 (m, 1H), 3.02-3.08 (m, 1H), 3.75-3.86 (m, 2H), 6.60-6.70 (m, 4H), 7.02-7.18 (m, 10H), 7.24-7.28 (m, 2H), 7.34-7.40 (m, 3H), 7.49-7.55 (m, 2H), 7.73-7.76 (m, 2H) ppm. ^{13}C NMR (101 MHz, $CDCl_3$): δ = 15.9, 21.5, 22.0, 24.2, 26.8, 27.2, 33.2, 38.1, 46.2, 46.3, 68.3, 68.6, 112.1, 120.7, 123.6, 124.4, 126.4, 126.5, 126.9, 127.2, 127.4, 128.00, 128.04, 128.08, 128.2, 128.7, 130.2, 130.4, 136.5, 138.6, 141.6, 148.0, 148.4, 157.1, 168.9 ppm. HRMS (ESI): m/z calculated for [$C_{44}H_{46}N_2NaO$] [M+Na$^+$]: 641.3508, found: 641.3500.

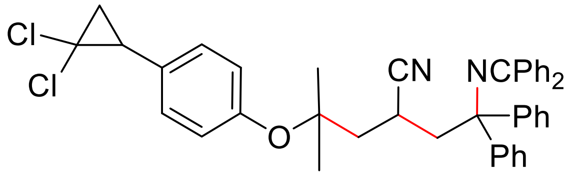

62, 50:50 d.r.

4-(4-(2,2-Dichlorocyclopropyl)phenoxy)-2-(2-((diphenylmethylene)amino)-2,2-diphenylethyl)-4-methylpentanenitrile (62)

Following the general procedure, benzophenone *O*-(2-(4-(2,2-dichlorocyclopropyl)phenoxy)-2-methylpropanoyl) oxime **1ad** (93.4 mg, 0.2 mmol), ethene-1,1-diyldibenzene **2a** (72.0 mg, 0.4 mmol), and acrylonitrile **3a** (21.2 mg, 0.4 mmol) were used. Purification by column chromatography using pre-basified silica with pentane/EtOAc (40:1 to 30:1, v/v) as eluent afforded **62** as a white solid in 50:50 diastereomeric ratio (62 mg, 32% yield). Combined NMR data for both the isomers are reported here. ^{1}H NMR (400 MHz, $CDCl_3$): δ = 1.12 (s, 3H), 1.14 (s, 3H), 1.71 (dd, *J* = 14.4, 3.1 Hz, 1H), 1.78 (dd, *J* = 8.4, 7.4 Hz, 1H), 1.90-2.07 (m, 2H), 2.50-2.61 (m,

1H), 2.84 (dd, J = 10.7, 8.3 Hz, 1H), 2.89-3.03 (m, 2H), 6.57 (d, J = 5.3 Hz, 2H), 6.70-6.78 (m, 2H), 6.98-7.18 (m, 11H), 7.20-7.25 (m, 2H), 7.29-7.39 (m, 3H), 7.43-7.53 (m, 2H), 7.68-7.75 (m, 2H) ppm. ^{13}C NMR (101 MHz, $CDCl_3$): δ = 22.3, 25.9, 26.0, 26.9, 35.0, 45.2, 46.9, 61.0, 68.6, 79.4, 123.5, 123.5, 123.8, 126.5, 126.5, 126.9, 127.3, 127.4, 128.0, 128.1, 128.2, 128.8, 129.5, 129.6, 129.6, 130.3, 138.6, 141.6, 148.1, 148.5, 154.2, 168.8 ppm. HRMS (ESI): m/z calculated for [$C_{42}H_{38}N_2OCl_2Na$] [$M+Na^+$]: 679.2253, found: 679.2257.

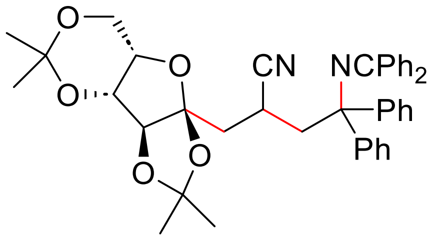

63, 50:50 d.r.

4-((Diphenylmethylene)amino)-4,4-diphenyl-2-(((3*aS*,3*bR*,7*aS*,8*aS*)-2,2,5,5-tetramethyltetrahydro-3*aH*-[1,3]dioxolo[4',5':4,5]furo[3,2-*d*][1,3]dioxin-8*a*-yl)methyl)butanenitrile (63)

Following the general procedure, benzophenone *O*-((3*aS*,3*bR*,7*aS*,8*aR*)-2,2,5,5-tetramethyltetrahydro-3*aH*-[1,3]dioxolo[4',5':4,5]furo[3,2-*d*][1,3]dioxine-8*a*-carbonyl) oxime **1af** (135.9 mg, 0.3 mmol), ethene-1,1-diyldibenzene **2a** (36.0 mg, 0.2 mmol), and acrylonitrile **3a** (21.2 mg, 0.4 mmol) were used. Purification by column chromatography using pre-basified silica with pentane/EtOAc (20:1 to 10:1, v/v) as eluent afforded **63** as a white solid in 50:50 diastereomeric ratio (90 mg, 70% yield). Combined NMR data for both the isomers are reported here. ^{1}H NMR (400 MHz, $CDCl_3$): δ = 1.13-1.29 (m, 6H), 1.36-1.44 (m, 6H), 1.98-2.03 (m, 1H), 2.26-2.37 (m, 1H), 2.60-2.69 (m, 1H), 2.90-2.99 (m, 1H), 3.02-3.10 (m, 1H), 3.75-4.01 (m, 3H), 4.17-4.21 (m, 2H), 6.57-6.63 (m, 2H), 6.98-7.04 (m, 2H), 7.09-7.22 (m, 9H), 7.30-7.42 (m, 5H), 7.71-7.74 (m, 2H) ppm. ^{13}C NMR (101 MHz, $CDCl_3$): δ = 18.8, 19.0, 22.6, 22.7, 26.58, 26.64, 27.39, 27.43, 28.6, 28.9, 41.4, 42.1, 44.48, 44.53, 60.29, 60.31, 68.4, 68.5, 72.4, 73.4, 73.6, 86.3, 86.4, 97.4, 97.5, 111.5, 111.6, 113.7, 123.0, 123.1, 126.29, 126.39, 126.42, 126.5, 126.8, 127.0, 127.25, 127.28, 127.36, 127.44, 127.91, 127.96, 127.98, 128.04, 128.06, 128.09, 128.1, 128.80, 128.81, 130.1, 130.2, 138.6, 138.7,

141.6, 141.7, 148.0, 148.1, 148.3, 148.4, 168.6, 168.7 ppm. HRMS (ESI): m/z calculated for [$C_{41}H_{42}N_2NaO_5$] [$M+Na^+$]: 665.2991, found: 665.2998.

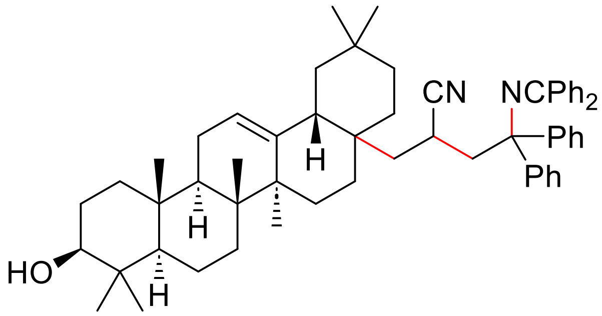

64, 50:50 d.r.

4-((Diphenylmethylene)amino)-2-(((6*aS*,6*bR*,8*aR*,10*S*,12*aR*,12*bR*,14*bR*)-10-hydroxy-2,2,6*a*,6*b*,9,9,12*a*-heptamethyl-1,2,3,4,4a,5,6,6*a*,6*b*,7,8,8*a*,9,10,11,12,12*a*,12*b*,13,14*b*-icosahydropicen-4*a*-yl)methyl)-4,4-diphenylbutanenitrile (64)

Following the general procedure, benzophenone *O*-((4*aS*,6*aS*,6*bR*,8*aR*,10*S*,12*aR*,12*bR*,14b*S*)-10-hydroxy-2,2,6*a*,6*b*,9,9,12*a*-heptamethyl-1,2,3,4,4*a*,5,6,6*a*,6*b*,7,8,8*a*,9,10,11,12,12*a*,12*b*,13,14*b*-icosahydropicene-4*a*-carbonyl) oxime **1ag** (190.5 mg, 0.3 mmol), ethene-1,1-diyldibenzene **2a** (36.0 mg, 0.2 mmol), and acrylonitrile **3a** (21.2 mg, 0.4 mmol) were used. Purification by column chromatography using pre-basified silica with pentane/EtOAc (30:1 to 10:1, v/v) as eluent afforded **64** as a white solid in 50:50 diastereomeric ratio (79 mg, 48% yield). Combined NMR data for both the isomers are reported here. ^{1}H NMR (400 MHz, $CDCl_3$): δ = 0.69-0.75 (m, 3H), 0.78-0.85 (m, 9H), 0.86-1.19 (m, 20H), 1.25-1.66 (m, 9H), 1.73-1.89 (m, 4H), 1.99-2.06 (m, 1H), 2.09-2.13 (m, 1H), 2.30-2.34 (m, 1H), 2.38-2.58 (m, 1H), 2.89-3.03 (m, 1H), 3.19-3.23 (m, 1H), 4.95-5.18 (m, 1H), 6.57 (m, 2H), 6.84-6.88 (m, 1H), 6.96-7.22 (m, 8H), 7.25-7.40 (m, 5H), 7.42-7.74 (m, 4H) ppm. ^{13}C NMR (101 MHz, $CDCl_3$): δ = 15.58, 15.62, 15.69, 15.70, 16.93, 16.96, 18.4, 20.7, 20.8, 22.9, 23.0, 23.6, 23.7, 25.5, 25.9, 26.1, 26.3, 27.3, 28.17, 28.19, 30.78, 30.82, 32.2, 32.4, 33.2, 33.3, 34.2, 34.3, 35.6, 36.1, 36.96, 36.98, 38.7, 38.85, 38.87, 39.9, 40.0, 41.49, 41.54, 43.8, 45.0, 46.1, 46.4, 46.7, 47.0, 47.4, 47.55, 47.62, 55.2, 68.55, 68.64, 79.00, 79.05, 122.5, 122.9, 124.0, 124.4, 126.29, 126.39, 126.45, 126.48, 126.7, 126.9, 127.29, 127.34, 127.39, 127.88, 127.95, 127.99,

128.03, 128.10, 128.13, 128.2, 128.7, 128.8, 130.1, 130.2, 138.6, 138.8, 141.6, 144.0, 144.3, 148.3, 148.5, 148.6, 149.2, 168.6, 169.0 ppm. HRMS (ESI): m/z calculated for [$C_{59}H_{72}N_2NaO$] [M+Na$^+$]: 847.5542, found: 847.5537.

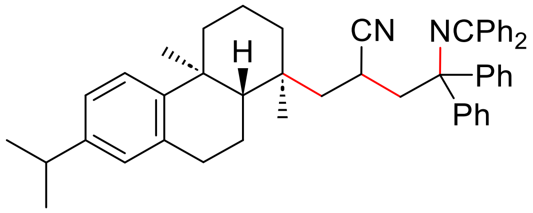

65, 50:50 d.r.

4-((Diphenylmethylene)amino)-2-(((1*S*,4*aS*,10*aS*)-7-isopropyl-1,4*a*-dimethyl-1,2,3,4,4*a*,9,10,10*a*-octahydrophenanthren-1-yl)methyl)-4,4-diphenylbutanenitrile (65)

Following the general procedure, benzophenone *O*-((1*R*,4*aS*,10*aR*)-7-isopropyl-1,4*a*-dimethyl-1,2,3,4,4*a*,9,10,10*a*-octahydrophenanthrene-1-carbonyl) oxime **1ah** (143.7 mg, 0.3 mmol), ethene-1,1-diyldibenzene **2a** (36.0 mg, 0.2 mmol), and acrylonitrile **3a** (21.2 mg, 0.4 mmol) were used. Purification by column chromatography using pre-basified silica with pentane/EtOAc (400:1 to 20:1, v/v) as eluent afforded **65** as a white solid in 50:50 diastereomeric ratio (76 mg, 57% yield). Combined NMR data for both the isomers are reported here. ^{1}H NMR (400 MHz, $CDCl_3$): δ = 0.67-0.93 (m, 3H), 1.00-1.10 (m, 1H), 1.13-1.17 (m, 3H), 1.19-1.35 (m, 3H), 1.47-1.67 (m, 4H), 1.75-1.85 (m, 1H), 2.17-2.22 (m, 1H), 2.34-2.49 (m, 1H), 2.58-2.72 (m, 1H), 2.74-2.93 (m, 2H), 2.98-3.08 (m, 1H), 6.59 (m, 2H), 6.83-6.86 (m, 1H), 6.97-7.16 (m, 11H), 7.17-7.25 (m, 2H), 7.29-7.40 (m, 3H), 7.41-7.52 (m, 2H), 7.69-7.73 (m, 2H) ppm. ^{13}C NMR (101 MHz, $CDCl_3$): δ = 18.8, 18.9, 19.0, 20.0, 21.0, 21.1, 21.5, 24.08, 24.13, 24.15, 25.4, 25.6, 29.7, 30.2, 33.5, 33.6, 36.5, 36.7, 36.86, 36.92, 37.6, 37.7, 38.1, 38.2, 46.2, 46.8, 47.1, 47.9, 48.6, 48.8, 68.57, 68.61, 123.8, 123.9, 124.1, 124.3, 124.4, 124.5, 126.40, 126.42, 126.44, 126.9, 127.0, 127.2, 127.4, 127.9, 128.01, 128.04, 128.09, 128.19, 128.21, 128.8, 130.22, 130.25, 134.6, 135.0, 138.6, 138.7, 141.5, 141.6, 145.6, 147.2, 147.4, 148.14, 148.16, 148.4, 148.5, 168.8, 168.9 ppm. HRMS (ESI): m/z calculated for [$C_{49}H_{52}N_2Na$] [M+Na$^+$]: 691.4028, found: 691.4024.

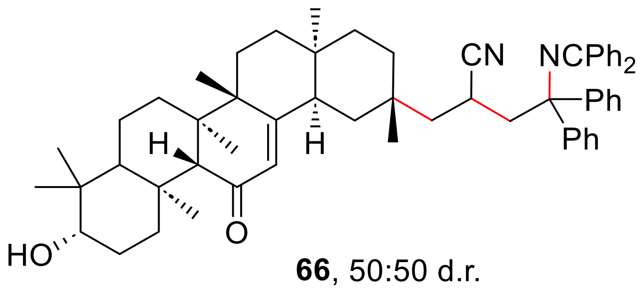

66, 50:50 d.r.

4-((Diphenylmethylene)amino)-2-(((2*S*,4*aR*,6*aS*,6*bR*,10*S*,12*aS*,12*bR*,14*bR*)-10-hydroxy-2,4*a*,6*a*,6*b*,9,9,12*a*-heptamethyl-13-oxo-1,2,3,4,4*a*,5,6,6*a*,6*b*,7,8,8*a*,9,10,11,12,12*a*,12*b*,13,14*b*-icosahydropicen-2-yl)methyl)-4,4-diphenylbutanenitrile (66)

Following the general procedure, (2*S*,4*aS*,6*aS*,6*bR*,8*aR*,10*S*,12*aS*,12*bR*,14*bR*)-2-((((diphenylmethylene)amino)oxy)carbonyl)-10-hydroxy-2,4*a*,6*a*,6*b*,9,9,12*a*-heptamethyl-1,3,4,4*a*,5,6,6*a*,6*b*,7,8,8*a*,9,10,11,12,12*a*,12*b*,14*b*-octadecahydropicen-13(2*H*)-one **1ai** (194.7 mg, 0.3 mmol), ethene-1,1-diyldibenzene **2a** (36.0 mg, 0.2 mmol), and acrylonitrile **3a** (21.2 mg, 0.4 mmol) were used. Purification by column chromatography using pre-basified silica with pentane/EtOAc (8:1 to 3:1, v/v) as eluent afforded **66** as a white solid in 50:50 diastereomeric ratio (92 mg, 55% yield). Combined NMR data for both the isomers are reported here. ^{1}H NMR (400 MHz, $CDCl_3$): δ = 0.58-0.65 (m, 3H), 0.71-0.76 (m, 6H), 0.85-0.96 (m, 6H), 1.00-1.09 (m, 9H), 1.15-1.36 (m, 12H), 1.48-1.61 (m, 4H), 1.65-1.95 (m, 3H), 2.21-2.31 (m, 2H), 2.41-2.62 (m, 1H), 2.67-2.77 (m, 1H), 2.87-3.01 (m, 1H), 3.12-3.18 (m, 1H), 5.42-5.49 (m, 1H), 6.48 (m, 2H), 6.88-7.10 (m, 9H), 7.12-7.19 (m, 2H), 7.23-7.33 (m, 3H), 7.34-7.45 (m, 2H), 7.60-7.64 (m, 2H) ppm. ^{13}C NMR (101 MHz, $CDCl_3$): δ = 15.7, 16.4, 16.5, 17.6, 18.79, 18.82, 18.84, 20.7, 20.8, 21.2, 21.3, 23.4, 23.5, 26.4, 27.4, 28.2, 28.58, 28.62, 28.69, 29.8, 32.1, 32.5, 32.6, 32.9, 33.8, 34.2, 34.3, 35.75, 35.81, 37.2, 39.2, 39.3, 42.8, 42.9, 43.35, 43.37, 43.39, 44.0, 45.47, 45.51, 46.1, 46.2, 46.4, 46.9, 47.0, 50.1, 50.3, 55.0, 61.8, 61.9, 68.5, 68.6, 78.9, 124.2, 124.4, 126.4, 126.5, 126.6, 126.9, 127.0, 127.1, 127.2, 127.4, 127.99, 128.03, 128.05, 128.10, 128.15, 128.18, 128.29, 128.34, 128.68, 128.70, 128.73, 130.3, 138.5, 138.6, 141.5, 148.0, 148.3, 148.6, 168.84, 168.88, 169.2, 169.67, 169.70, 200.2, 200.3 ppm. HRMS (ESI): m/z calculated for

[$C_{59}H_{70}N_2NaO_2$] [M+Na^+]: 861.5335, found: 861.5327.

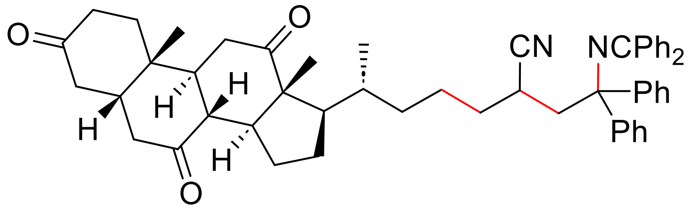

67, 50:50 d.r.

(6*R*)-6-((5*S*,8*R*,9S,10*S*,13*R*,14*S*,17*R*)-10,13-Dimethyl-3,7,12-trioxohexadecahydro-1*H*-cyclopenta[*a*]phenanthren-17-yl)-2-(2-((diphenylmethylene)amino)-2,2-diphenylethyl)heptanenitrile (67)

Following the general procedure, (5*S*,8*R*,9*S*,10*S*,13*R*,14*S*,17*R*)-17-((*R*)-5-(((diphenylmethylene)amino)oxy)-5-oxopentan-2-yl)-10,13-dimethyldecahydro-1*H*-cyclopenta[*a*]phenanthrene-3,7,12(2*H*,4*H*,8*H*)-trione **1aj** (174.3 mg, 0.3 mmol), ethene-1,1-diyldibenzene **2a** (36.0 mg, 0.2 mmol), and acrylonitrile **3a** (21.2 mg, 0.4 mmol) were used. Purification by column chromatography using pre-basified silica with pentane/EtOAc (10:1 to 8:1, v/v) as eluent afforded **67** as a white solid in 50:50 diastereomeric ratio (71 mg, 46% yield). Combined NMR data for both the isomers are reported here. ^{1}H NMR (400 MHz, $CDCl_3$): δ = 0.75-0.77 (m, 3H), 1.02-1.05 (m, 4H), 1.39 (m, 6H), 1.51-1.65 (m, 2H), 1.70-2.04 (m, 6H), 2.07-2.16 (m, 2H), 2.19-2.37 (m, 9H), 2.54-2.62 (m, 1H), 2.80-2.94 (m, 5H), 6.54 (m, 2H), 6.99-7.13 (m, 6H), 7.15-7.25 (m, 5H), 7.27-7.40 (m, 4H), 7.41-7.47 (m, 2H), 7.69-7.72 (m, 2H) ppm. ^{13}C NMR (101 MHz, $CDCl_3$): δ = 11.90, 11.92, 18.90, 18.98, 22.0, 23.89, 24.02, 25.2, 26.6, 26.7, 27.9, 34.25, 34.32, 34.7, 35.3, 35.9, 36.0, 36.1, 36.6, 38.7, 42.9, 43.4, 43.6, 45.1, 45.6, 45.9, 46.0, 46.9, 51.9, 56.9, 68.33, 68.35, 122.8, 122.9, 126.4, 126.5, 127.0, 127.2, 127.4, 127.5, 127.9, 127.98, 128.03, 128.12, 128.14, 128.4, 128.7, 128.8, 129.4, 130.2, 138.46, 138.48, 141.50, 141.51, 148.02, 148.03, 148.40, 148.43, 168.61, 168.63, 208.88, 208.90, 209.2, 212.0 ppm. HRMS (ESI): m/z calculated for [$C_{53}H_{58}N_2NaO_3$] [M+Na^+]: 793.4345, found: 793.4341.

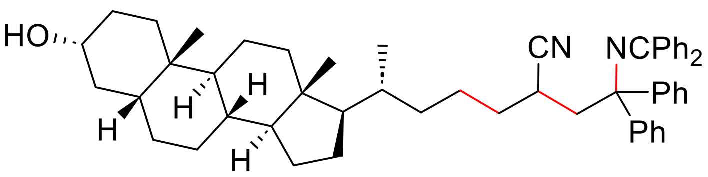

68, 50:50 d.r.

(6*R*)-2-(2-((Diphenylmethylene)amino)-2,2-diphenylethyl)-6-((3*R*,5*R*,8*R*,9*S*,10*S*,13*R*,14*S*,17*R*)-3-hydroxy-10,13-dimethylhexadecahydro-1*H*-cyclopenta[*a*]phenanthren-17-yl)heptanenitrile (68)

Following the general procedure, benzophenone *O*-((*R*)-4-((3*R*,5*R*,8*R*,9*S*,10*S*,13*R*,14*S*,17*R*)-3-hydroxy-10,13-dimethylhexadecahydro-1*H*-cyclopenta[*a*]phenanthren-17-yl)pentanoyl) oxime **1ak** (166.5 mg, 0.3 mmol), ethene-1,1-diyldibenzene **2a** (36.0 mg, 0.2 mmol), and acrylonitrile **3a** (21.2 mg, 0.4 mmol) were used. Purification by column chromatography using pre-basified silica with pentane/EtOAc (10:1 to 8:1, v/v) as eluent afforded **68** as a white solid in 50:50 diastereomeric ratio (64 mg, 43% yield). Combined NMR data for both the isomers are reported here. ^{1}H NMR (400 MHz, $CDCl_3$): δ = 0.61-0.62 (m, 3H), 0.82-0.84 (m, 4H), 0.92-0.93 (m, 3H), 0.97-1.44 (m, 18H), 1.48-1.57 (m, 3H), 1.64-1.97 (m, 9H), 2.33-2.38 (m, 1H), 2.55-2.62 (m, 1H), 2.84-2.90 (m, 1H), 3.58-3.66 (m, 1H), 6.56 (m, 2H), 7.00-7.19 (m, 9H), 7.22-7.26 (m, 2H), 7.33-7.42 (m, 3H), 7.44-7.46 (m, 2H), 7.70-7.72 (m, 2H) ppm. ^{13}C NMR (101 MHz, $CDCl_3$): δ = 12.1, 18.5, 18.6, 20.9, 23.4, 23.5, 24.3, 26.5, 26.7, 26.8, 27.3, 28.4, 30.6, 34.4, 34.5, 34.7, 35.1, 35.3, 35.46, 35.53, 35.6, 36.0, 36.6, 40.3, 40.5, 42.2, 42.8, 43.5, 43.6, 56.3, 56.6, 68.36, 68.38, 72.0, 122.8, 122.9, 126.43, 126.45, 127.0, 127.2, 127.4, 127.9, 127.96, 127.99, 128.04, 128.1, 128.7, 130.2, 138.5, 141.5, 148.07, 148.10, 148.4, 168.6 ppm. HRMS (ESI): m/z calculated for [$C_{53}H_{64}N_2NaO$] [M+Na^+]: 767.4916, found: 767.4909.

5. Synthetic application

5.1. Large scale synthesis of product 57

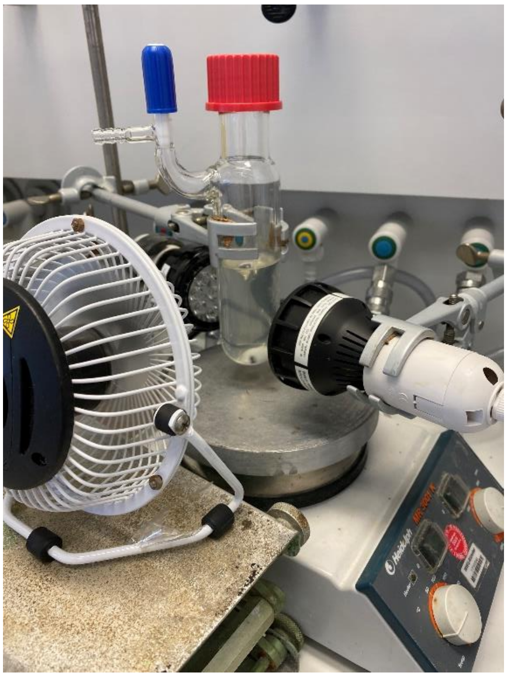

Fig. S9. Reaction setup used for the large scale synthesis of **57** featuring two 18 W blue LEDs (λ_{max} = 405 nm).

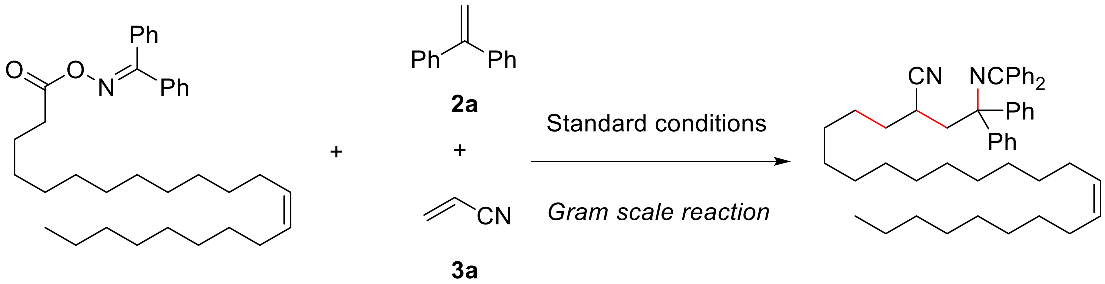

1y, 5.0 mmol

57, 42% (1.48 g)

A 100 mL Schlenk tube was charged with a magnetic stirring bar, thioxanthone (106 mg, 0.499 mmol, 10 mol%), and oxime ester **1y** (2.59 g, 5.00 mmol, 1.0 equiv). The Schlenk tube was evacuated and backfilled with argon three times and was then charged with dry EtOAc (50 mL, 0.10 M), 1,1-diphenylethylene (**2a**,1.81 g, 10.0 mmol, 2.0 equiv), and acrylonitrile (**3a**, 531 mg, 10.0 mmol, 2.0 equiv). The reaction mixture was then irradiated at 405 nm for 12 h using the setup shown above. The solvent was removed under reduced pressure and column chromatography on silica gel (prebasified using *n*-pentane/CH_2Cl_2/NEt_3 = 5/1/0.03; *n*-pentane/CH_2Cl_2 = 5/1 to 4/1) gave **57** as colorless oil (1.48 g, 2.09 mmol, 42% yield).

5.2. Dihydroxylation of product 57

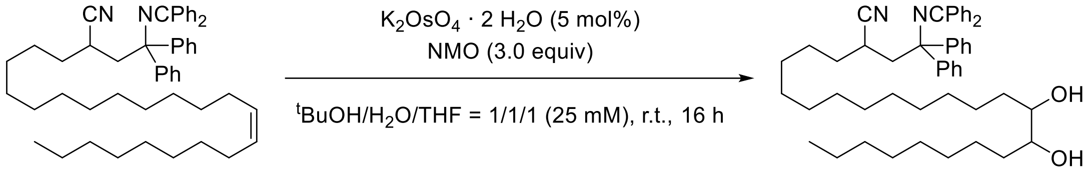

69, 78%, 50:50 d.r.

A 20 mL vial was charged with **57** (70.7 mg, 0.100 mmol, 1.0 equiv), ^{t}BuOH/H_2O/THF (1/1/1, 4.0 mL, 25 mM), $K_2OsO_4 \cdot H_2O$ (1.8 mg, 0.0049 mmol, 5 mol%), and NMO (35.1 mg, 0.300 mmol, 3.0 equiv). The resulting mixture was stirred at r.t. for 16 h and was then filtered through a short pad of silica which has been prebasified (EtOAc + 2% NEt_3), eluting with EtOAc (50 mL). Concentration under reduced pressure followed by column chromatography (silica prebasified with CH_2Cl_2 + 0.3% NEt_3; CH_2Cl_2 + 1% MeOH) gave dihydroxylated product **69** as a white solid in 50:50 diastereomeric ratio (57.5 mg, 0.0776 mmol, 78%). Combined NMR data for both the isomers are reported here. ^{1}H NMR (400 MHz, $CDCl_3$): δ = 0.86-0.92 (m, 3H), 1.02-1.58 (m, 38H), 1.89 (s, 2H), 2.35 (dd, J = 13.6, 3.6 Hz, 1H), 2.58 (tt, J = 8.4, 4.0 Hz, 1H), 2.87 (dd, J = 13.6, 8.3 Hz, 1H), 3.60 (t, J = 5.3 Hz, 2H), 6.55 (d, J = 7.5 Hz, 2H), 6.97-7.20 (m, 9H), 7.20-7.26 (m, 2H), 7.31-7.51 (m, 5H), 7.67-7.74 (m, 2H) ppm. ^{13}C NMR (101 MHz, $CDCl_3$): δ = 14.2, 22.8, 26.2, 26.7, 26.8, 28.9, 29.3, 29.4, 29.6, 29.7, 29.7, 29.7, 29.8, 31.3, 32.0, 34.0, 43.6, 68.4, 74.8, 122.9, 126.5, 126.5, 127.0, 127.2, 127.5, 128.0, 128.0, 128.1, 128.2, 128.7, 130.3, 138.6, 141.6, 148.1, 148.5, 168.7 ppm. HRMS (ESI): m/z calculated for $[C_{51}H_{68}N_2O_2Na]^+$ $[M+Na^+]$: 763.5173, found: 763.5173.

5.3. Deprotection of product 21 and 68

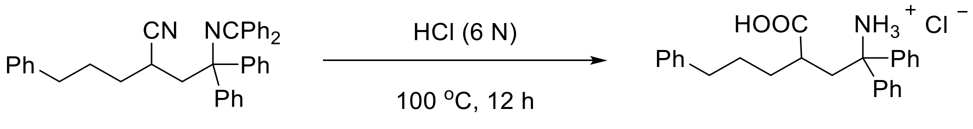

21

70, 71%

A 10 mL tube was charged with compound **21** (51.8 mg, 0.1 mmol) and 6 N HCl (2.0 mL), and the solution was stirred at 100 °C for 12 h. After cooling the mixture to room temperature, Et_2O (1 mL) was added into the tube. Then, stirring the resulting mixture and carefully remove the upper organic phase with a dropper. After repeating this

operation once, the water layer was combined and the tube was washed with MeOH (5 mL). Next, water and MeOH were combined, and the volatile was removed in vacuo, and the product **70** was obtained as a white solid (29 mg, 71% yield). *Note: the product **70** is slightly soluble in water and Et_2O, but soluble in methanol.* ^{1}H NMR (400 MHz, DMSO-d_6): δ = 1.18-1.46 (m, 1H), 1.50-1.76 (m, 2H), 1.79-1.98 (m, 1H), 2.46-2.61 (m, 3H), 3.05-3.23 (m, 2H), 7.12-7.21 (m, 3H), 7.23-7.32 (m, 4H), 7.31-7.48 (m, 8H), 8.96-9.06 (m, 1H), 9.42 (s, 1H), 11.8 (s, 1H) ppm. ^{13}C NMR (101 MHz, DMSO-d_6): δ = 28.1, 29.3, 34.9, 41.3, 41.5, 72.6, 125.6, 125.76, 125.79, 127.4, 127.5, 128.18, 128.25, 128.57, 128.62, 141.6, 143.7, 144.5, 172.4 ppm.

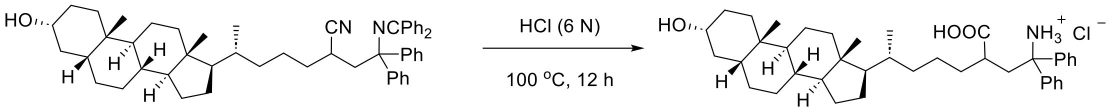

68 **71**, 66%, 50:50 d.r.

A 10 mL tube was charged with compound **68** (51.8 mg, 0.1 mmol) and 6 N HCl (2.0 mL), and the solution was stirred at 100 °C for 12 h. After cooling the mixture to room temperature, Et_2O (1 mL) was added into the tube. Then, stirring the resulting mixture and carefully remove the upper organic phase with a dropper. After repeating this operation once, the water layer was combined and the tube was washed with MeOH (5 mL). Next, water and MeOH were combined, and the volatile was removed in vacuo, and the product **71** was obtained as a white solid in 50:50 diastereomeric ratio (42 mg, 66% yield). *Note: the product **71** is insoluble in water and Et_2O, but soluble in methanol.* Combined NMR data for both the isomers are reported here. ^{1}H NMR (400 MHz, CD_3OD): δ = 0.64-0.66 (m, 3H), 0.88-1.63 (m, 31H), 1.68-1.99 (m, 6H), 2.63 (m, 1H), 3.18 (m, 2H), 3.54 (m, 1H), 7.13-7.52 (m, 10H) ppm. ^{13}C NMR (101 MHz, CD_3OD): δ = 12.53, 12.55, 19.1, 19.2, 21.9, 23.9, 24.2, 24.3, 25.2, 27.6, 28.3, 29.4, 31.1, 31.6, 31.7, 35.6, 36.4, 36.6, 36.77, 36.81, 37.08, 37.13, 41.5, 41.8, 43.4, 43.6, 43.8, 57.35, 57.39, 57.8, 72.3, 74.4, 127.3, 127.4, 129.02, 129.08, 129.87, 129.91, 144.2, 144.3, 145.0, 174.0 ppm.

6. X-Ray analysis

X-Ray diffraction: Data sets for compound **19** were collected with a Bruker D8 Venture Photon III Diffractometer. Programs used: data collection: *APEX4* Version 2021.4-0[14] (Bruker AXS Inc., **2021**); cell refinement: *SAINT* Version 8.40B (Bruker AXS Inc., **2021**); data reduction: *SAINT* Version 8.40B (Bruker AXS Inc., **2021**); absorption correction, *SADABS* Version 2016/2 (Bruker AXS Inc., **2021**); structure solution *SHELXT*-Version 2018-3 [15] (Sheldrick, G. M. *Acta Cryst.*, **2015**, *A71,* 3-8); structure refinement *SHELXL*- Version 2018-3[16] (Sheldrick, G. M. *Acta Cryst.*, **2015**, *C71* (1), 3-8) and graphics, *XP*[17] (Version 5.1, Bruker AXS Inc., Madison, Wisconsin, USA, **1998**). *R*-values are given for observed reflections, and $w\mathrm{R}^2$ values are given for all reflections.

X-ray crystal structure analysis of 19 (glo10386): A colorless, plate-like specimen of $C_{35}H_{34}N_2O$, approximate dimensions 0.052 mm x 0.065 mm x 0.202 mm, was used for the X-ray crystallographic analysis. The X-ray intensity data were measured on a single crystal diffractometer Bruker D8 Venture Photon III system equipped with a micro focus tube Mo ImS (MoKα, λ = 0.71073 Å) and a MX mirror monochromator. A total of 576 frames were collected. The total exposure time was 4.80 hours. The frames were integrated with the Bruker SAINT software package using a narrow-frame algorithm. The integration of the data using a monoclinic unit cell yielded a total of 59448 reflections to a maximum θ angle of 26.74° (0.79 Å resolution), of which 5803 were independent (average redundancy 10.244, completeness = 99.7%, R_{int} = 9.05%, R_{sig} = 3.93%) and 4626 (79.72%) were greater than $2\sigma(F^2)$. The final cell constants of a = 9.3198(3) Å, b = 11.1833(4) Å, c = 26.6506(7) Å, β = 99.8120(10)°, volume = 2737.06(15) $Å^3$, are based upon the refinement of the XYZ-centroids of 7272 reflections above 20 σ(I) with 4.784° < 2θ < 53.40°. Data were corrected for absorption effects using the Multi-Scan method (SADABS). The ratio of minimum to maximum apparent transmission was 0.923. The calculated minimum and maximum transmission coefficients (based on crystal size) are 0.9860 and 0.9960. The structure was solved and refined using the Bruker SHELXTL Software Package, using the space group $P2_1/n$, with Z = 4 for the formula unit, $C_{35}H_{34}N_2O$. The final anisotropic full-

matrix least-squares refinement on F^2 with 343 variables converged at R1 = 4.23%, for the observed data and wR2 = 10.06% for all data. The goodness-of-fit was 1.059. The largest peak in the final difference electron density synthesis was 0.308 $e^-/Å^3$ and the largest hole was -0.221 $e^-/Å^3$ with an RMS deviation of 0.043 $e^-/Å^3$. On the basis of the final model, the calculated density was 1.210 g/cm^3 and F(000), 1064 e^-. CCDC Nr.: 2207448.

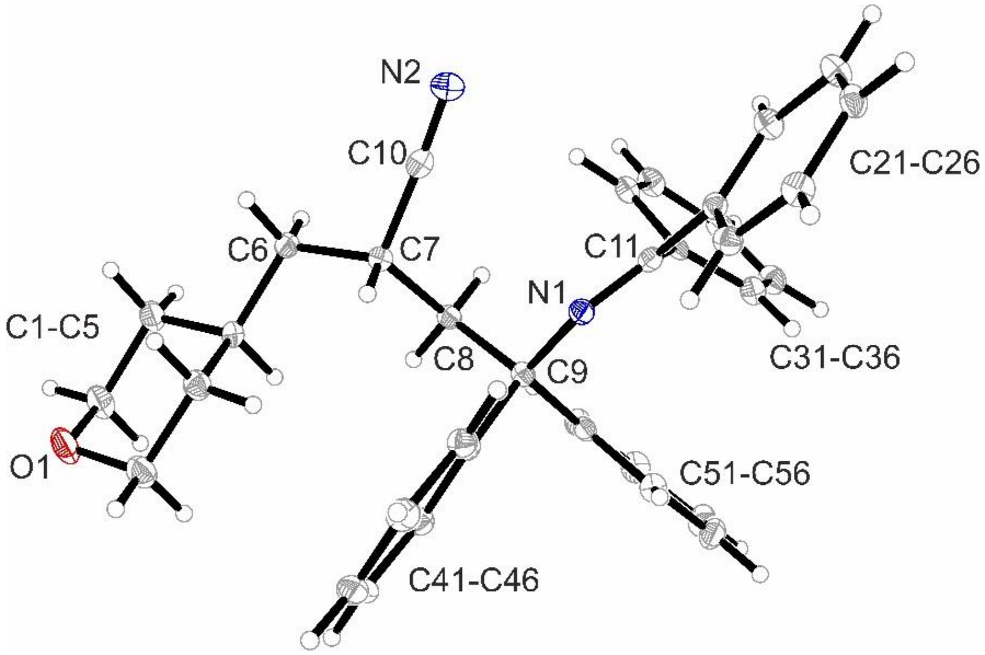

Fig. S10. Crystal structure of compound **19**. Thermal ellipsoids are shown at 50% probability.

7. References

[1] X. Mo, T. D. R. Morgan, H. T. Ang, D. G. Hall, *J. Am. Chem. Soc.* **2018**, *140*, 5264.

[2] R. S. Phatake, P. Patel, C. V. Ramana, *Org. Lett.* **2016**, 18, 292.

[3] T. Patra, P. Bellotti, F. Strieth-Kalthoff, F. Glorius, *Angew. Chem. Int. Ed.* **2020**, *59*, 3172.

[4] X. Yang, S. Liu, S. Yu, L. Kong, Y. Lan, X. Li, *Org. Lett.* **2018**, 20, 2698.

[5] J. Huang, G. Hu, S. An, D. Chen, M.Li, P. Li, *J. Org. Chem.* **2019**, *84*, 9758.

[6] Y.-Y. Cheng, J.-X. Yu, T. Lei, H.-Y. Hou, B. Chen, C.-H. Tung, L.-Z. Wu, *Angew. Chem. Int. Ed.* **2021**, *60*, 26822.

[7] L. Pitzer, F. Schäfers, F. Glorius, *Angew. Chem. Int. Ed.* **2019**, *58*, 8572.

[8] T. Gensch, M. Teders, F. Glorius, *J. Org. Chem.* **2017**, *82*, 9154.

[9] M. A. Cismesia, T. P. Yoon, *Chem. Sci.* **2015**, *6*, 5426.

[10] H. Heath, *Proc. R. Soc. London. Ser. A. Math. Phys. Sci.* **1956**, *235*, 518.

[11] E. E. Wegner, A. W. Adamson, *J. Am. Chem. Soc.* **1966**, *88*, 394.

[12] a) G. Tan, M. Das, H. Keum P. Bellotti, C. Daniliuc, F. Glorius, *Nat. Chem.* **2022**, *14*, 1174. b) J. Majhi, R. K. Dhungana, Á. Rentería-Gómez, M. Sharique, L. Li, W. Dong, O. Gutierrez, G. A. Molander, *J. Am. Chem. Soc.* **2022**, *144*, 15871. c) G. Tan, F. Paulus, Á. Rentería-Gómez, R. F. Lalisse, C. G. Daniliuc, O. Gutierrez, F. Glorius, *J. Am. Chem. Soc.* **2022**, *144*, 21664.

[13] a) H. Fische, *Chem. Rev.* **2001**, *101*, 3581; b) D. Leifert, A. Studer, *Angew. Chem. Int. Ed.* **2020**, *59*, 74.

[14] Bruker AXS (2019) APEX3 Version 2019.1-0, SAINT Version 8.40A and SADABS Bruker AXS area detector scaling and absorption correction Version 2016/2, Bruker AXS Inc., Madison, Wisconsin, USA.

[15] G. M. Sheldrick, SHELXT-Integrated space-group and crystal-structure determination, *Acta Cryst.* **A71**, 3–8 (2015).

[16] G. M. Sheldrick, Crystal structure refinement with SHELXL, *Acta Cryst.* **C71** (1), 3–8 (2015).

[17] Bruker AXS (1998) XP-Interactive molecular graphics, Version 5.1, Bruker AXS Inc., Madison, Wisconsin, USA.

8. Copies of ^{1}H and ^{13}C NMR spectra

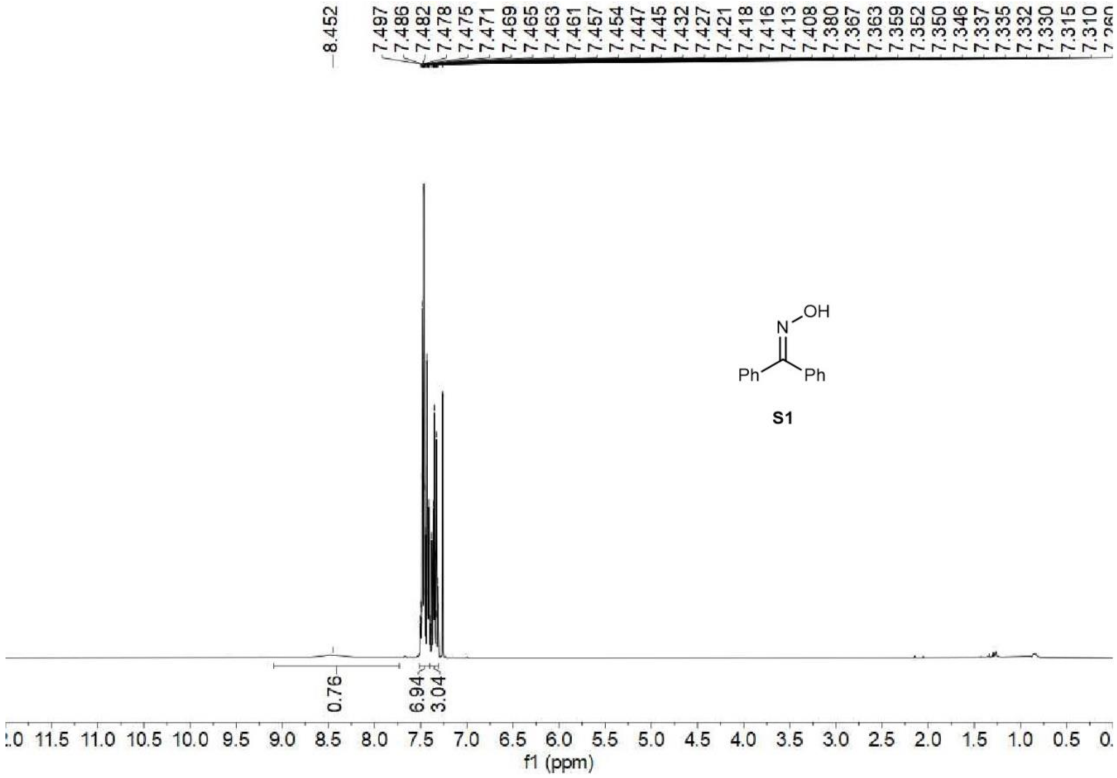

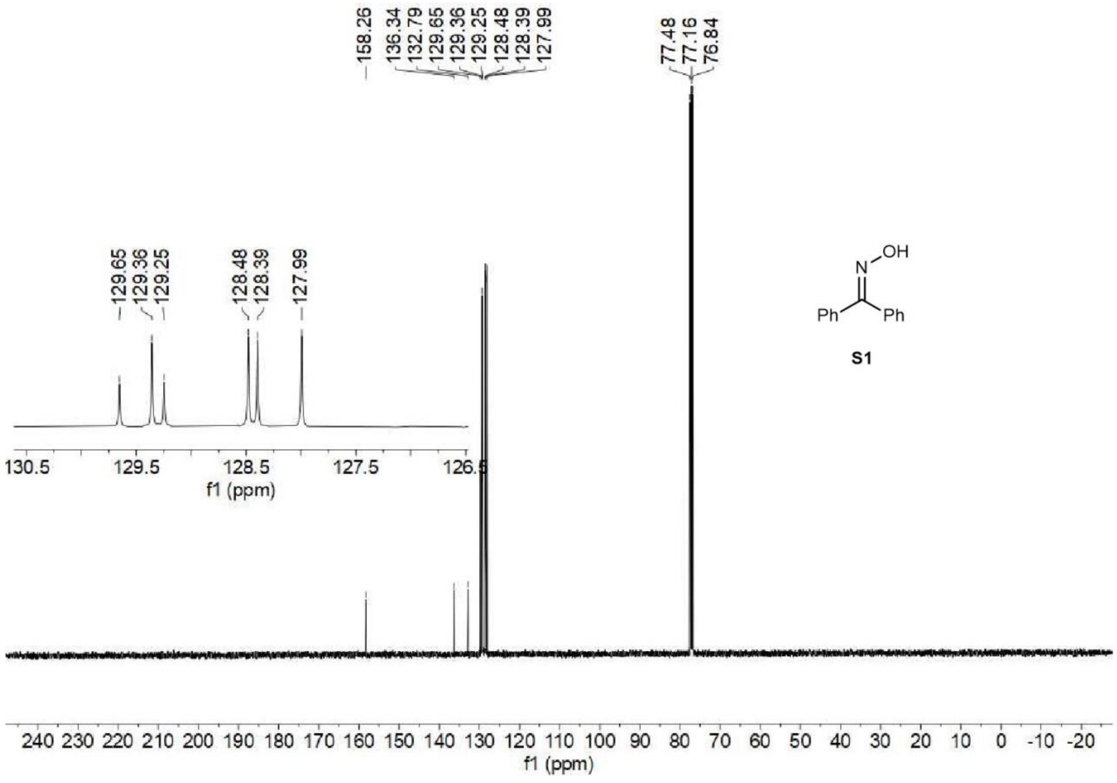

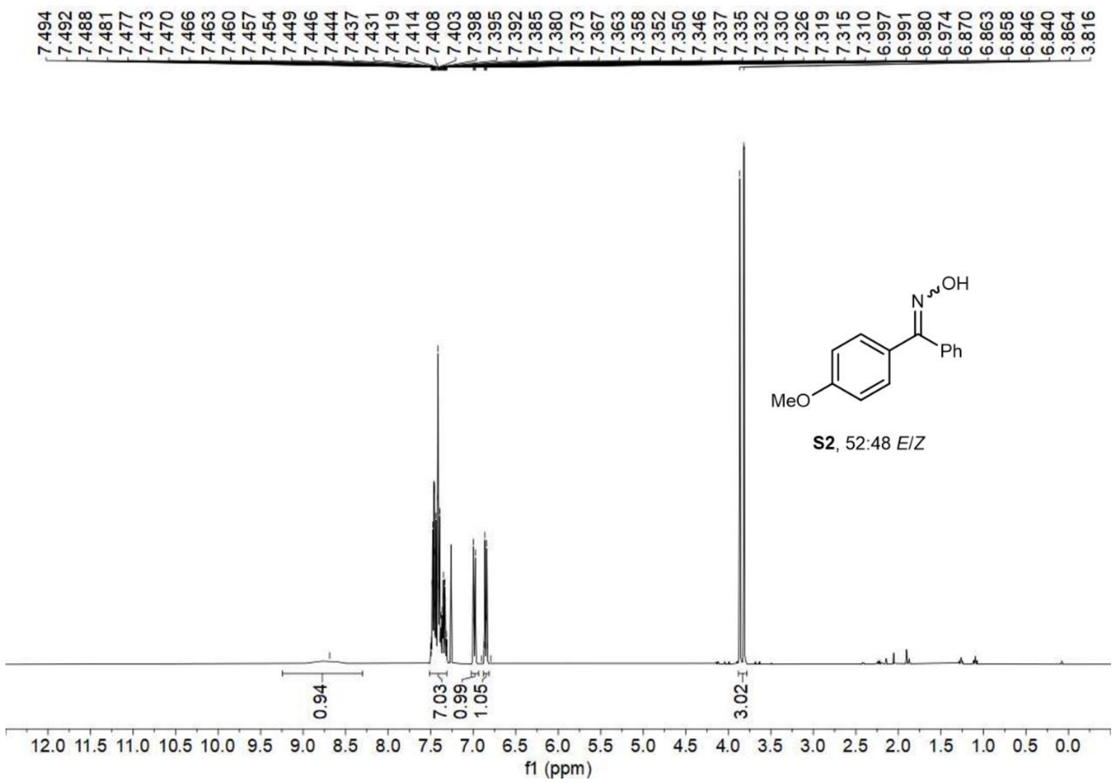
7.494 7.492 7.488 7.481 7.477 7.473 7.470 7.466 7.463 7.460 7.457 7.454 7.449 7.446 7.444 7.437 7.431 7.419 7.414 7.408 7.403 7.398 7.395 7.392 7.385 7.380 7.373 7.367 7.363 7.358 7.352 7.350 7.346 7.337 7.335 7.332 7.330 7.326 7.319 7.315 7.310 6.997 6.991 6.980 6.974 6.870 6.863 6.858 6.846 6.840 3.864 3.816
OH
N
Ph
MeO
S2, 52:48 E/Z
0.94
7.03
0.99
1.05
3.02
12.0 11.5 11.0 10.5 10.0 9.5 9.0 8.5 8.0 7.5 7.0 6.5 6.0 5.5 5.0 4.5 4.0 3.5 3.0 2.5 2.0 1.5 1.0 0.5 0.0
f1 (ppm)

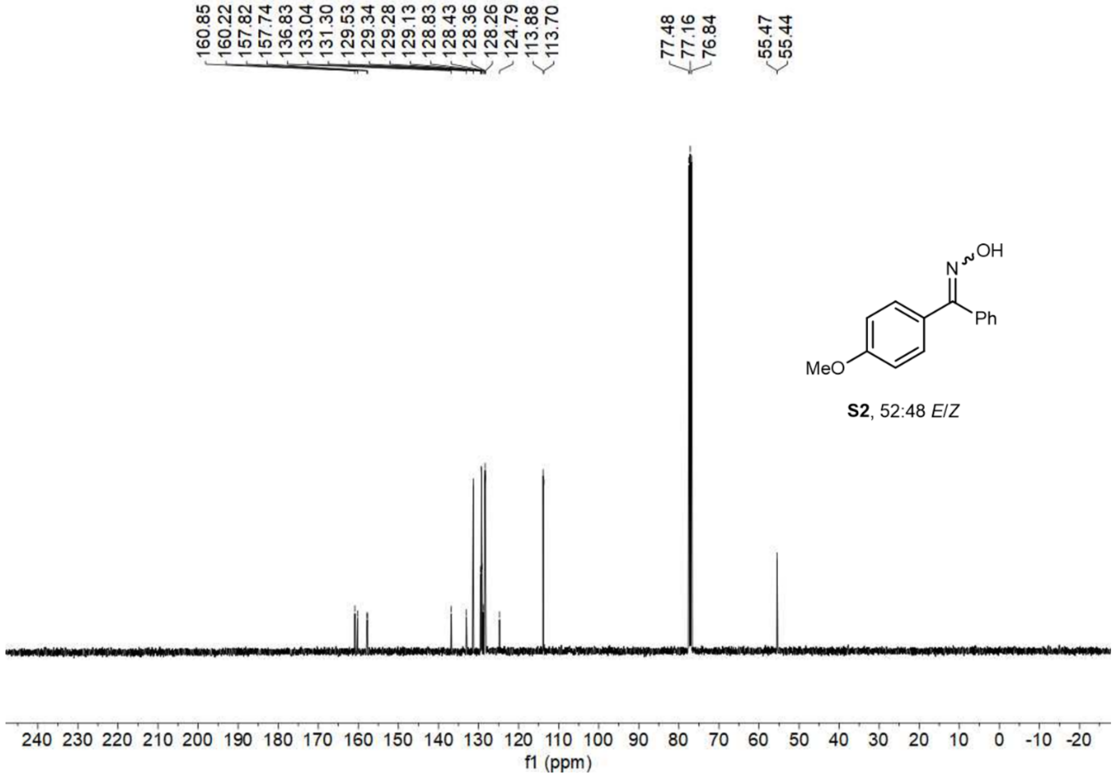
160.85 160.22 157.82 157.74 136.83 133.04 131.30 129.53 129.34 129.28 129.13 128.83 128.43 128.36 128.26 124.79 113.88 113.70
77.48 77.16 76.84
55.47 55.44
OH
N
Ph
MeO
S2, 52:48 E/Z
240 230 220 210 200 190 180 170 160 150 140 130 120 110 100 90 80 70 60 50 40 30 20 10 0 -10 -20
f1 (ppm)

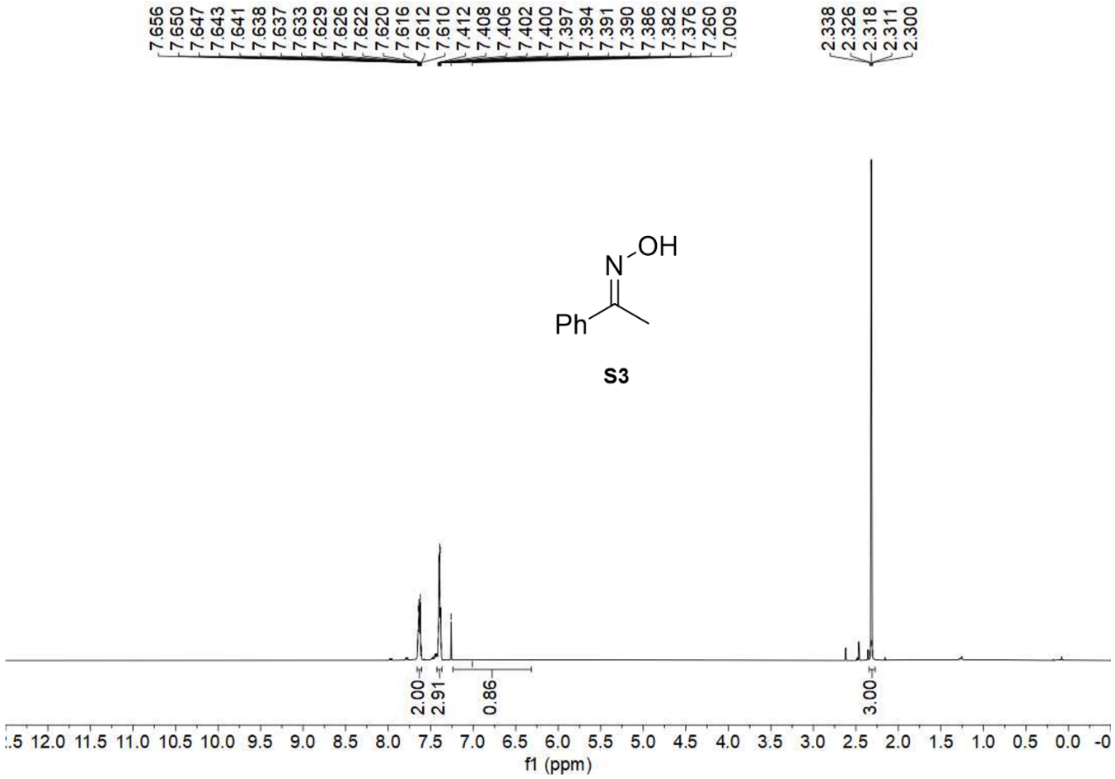
7.656 7.650 7.647 7.643 7.641 7.638 7.637 7.633 7.629 7.626 7.622 7.620 7.616 7.612 7.610 7.412 7.408 7.406 7.402 7.400 7.397 7.394 7.391 7.390 7.386 7.382 7.376 7.260 7.009
2.338 2.326 2.318 2.311 2.300
N
OH
Ph
S3
2.00
2.91
0.86
3.00
f1 (ppm)

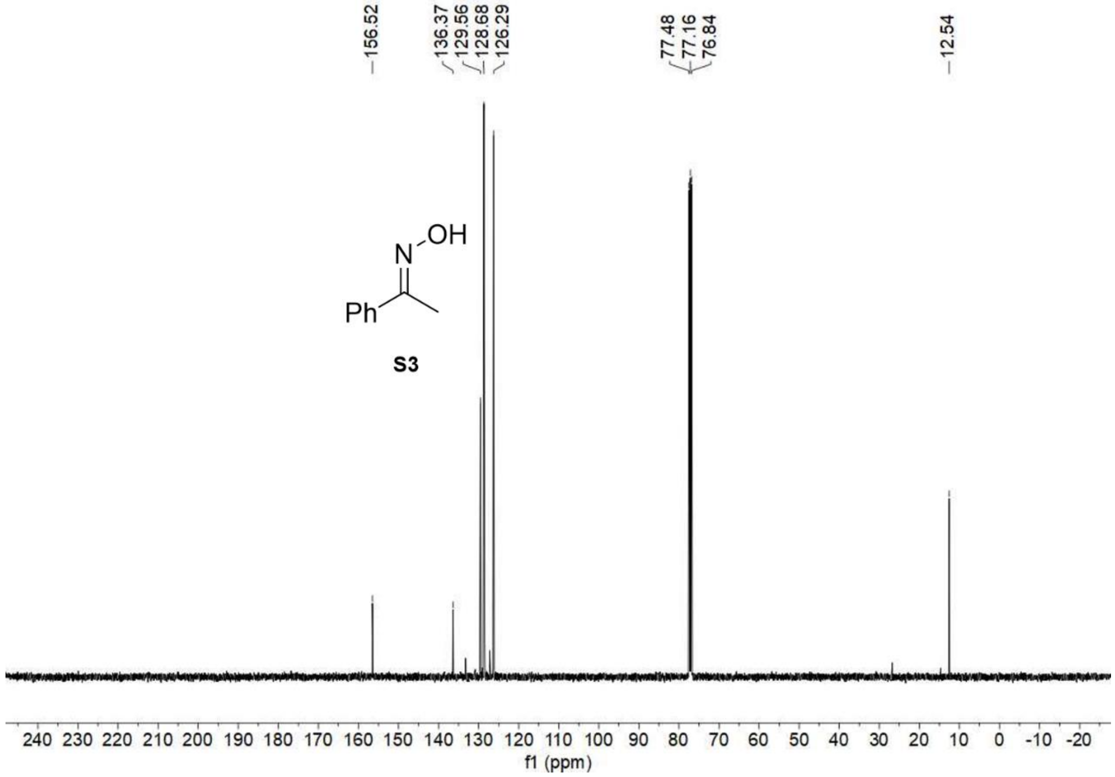
156.52
136.37 129.56 128.68 126.29
77.48 77.16 76.84
12.54
N
OH
Ph
S3
f1 (ppm)

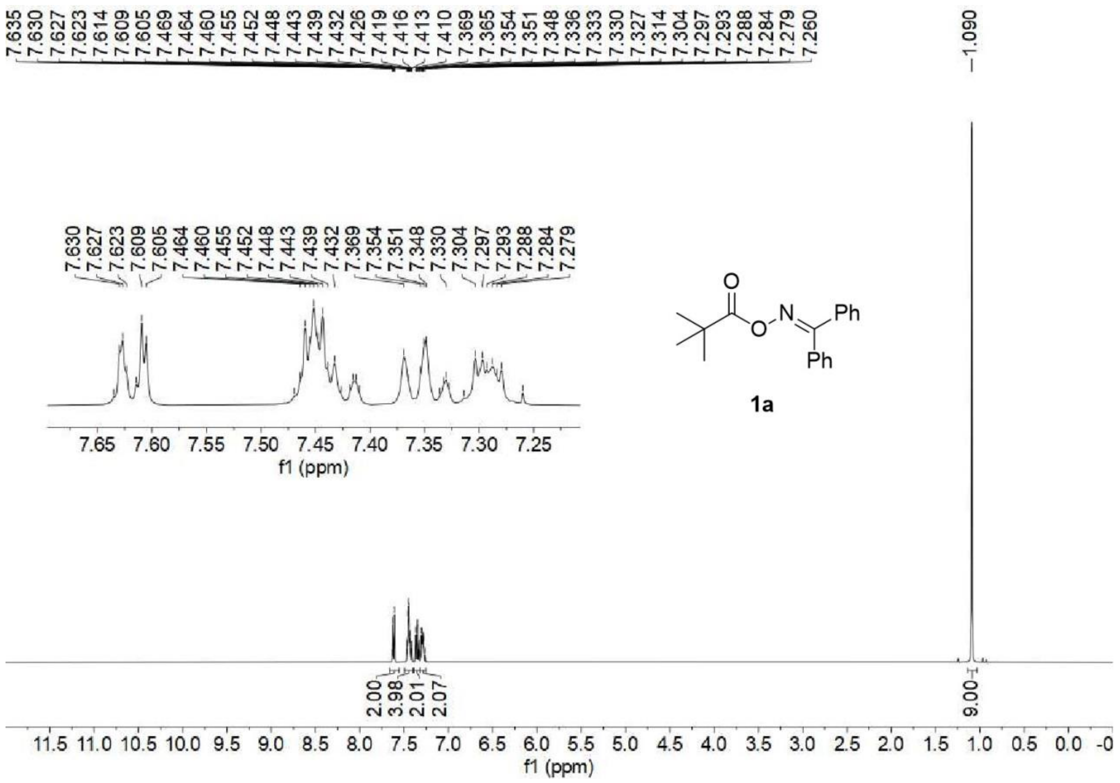
7.635 7.630 7.627 7.623 7.614 7.609 7.605 7.469 7.464 7.460 7.455 7.452 7.448 7.443 7.439 7.432 7.426 7.419 7.416 7.413 7.410 7.369 7.365 7.354 7.351 7.348 7.336 7.333 7.330 7.327 7.314 7.304 7.297 7.293 7.288 7.284 7.279 7.260
1.090
7.630 7.627 7.623 7.609 7.605 7.464 7.460 7.455 7.452 7.448 7.443 7.439 7.432 7.369 7.354 7.351 7.348 7.330 7.304 7.297 7.293 7.288 7.284 7.279
7.65 7.60 7.55 7.50 7.45 7.40 7.35 7.30 7.25
f1 (ppm)
O
N
O
Ph
Ph
1a
2.00
3.98
2.01
2.07
9.00
11.5 11.0 10.5 10.0 9.5 9.0 8.5 8.0 7.5 7.0 6.5 6.0 5.5 5.0 4.5 4.0 3.5 3.0 2.5 2.0 1.5 1.0 0.5 0.0 -0
f1 (ppm)

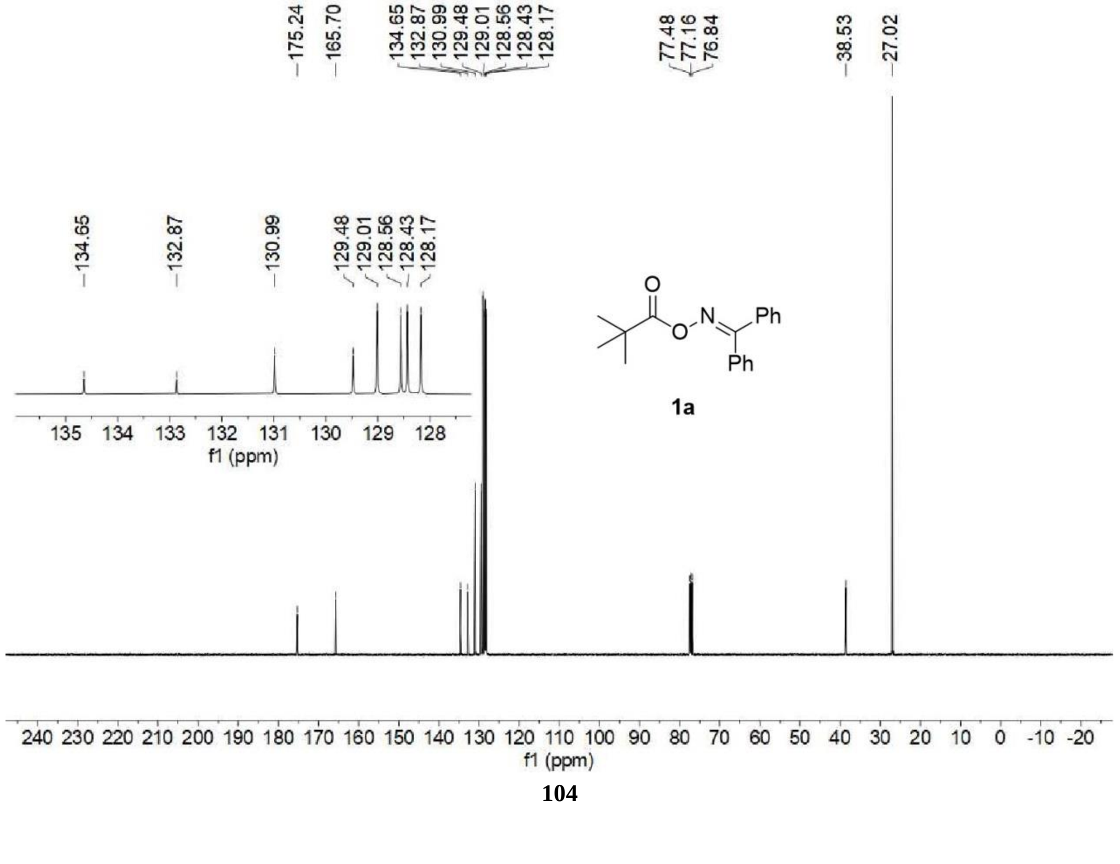
175.24
165.70
134.65 132.87 130.99 129.48 129.01 128.56 128.43 128.17
77.48 77.16 76.84
38.53
27.02
134.65
132.87
130.99
129.48 129.01 128.56 128.43 128.17
135 134 133 132 131 130 129 128
f1 (ppm)
O
N
O
Ph
Ph
1a
240 230 220 210 200 190 180 170 160 150 140 130 120 110 100 90 80 70 60 50 40 30 20 10 0 -10 -20
f1 (ppm)

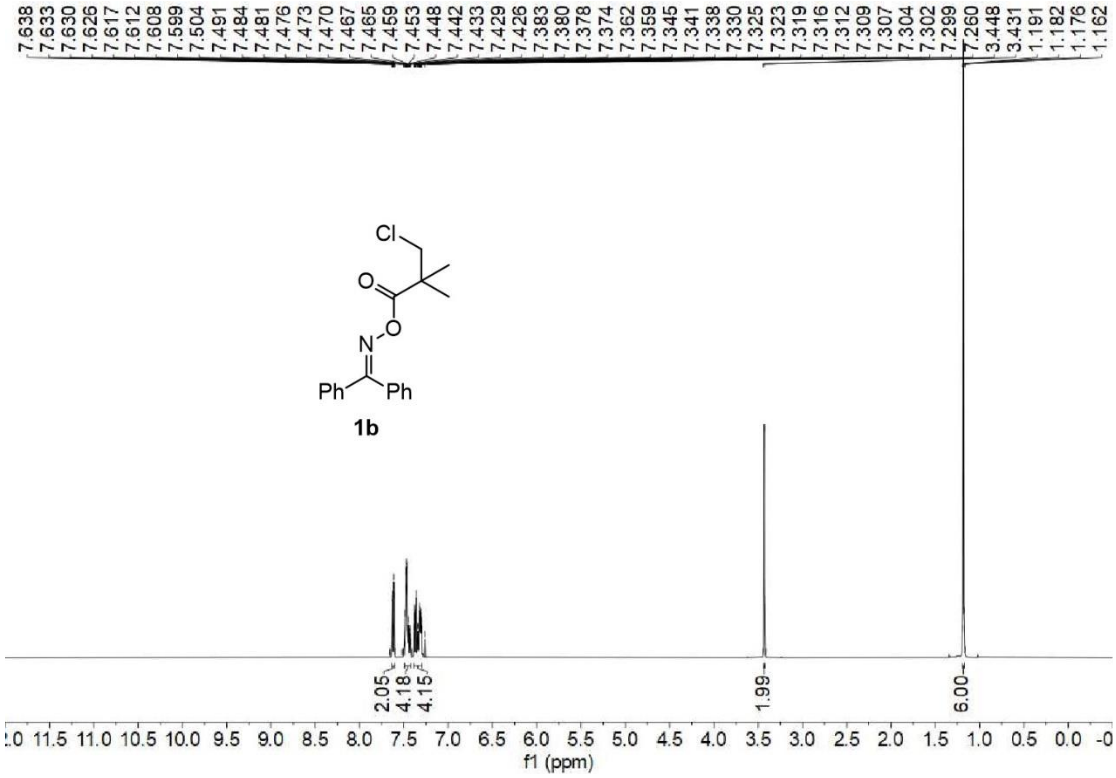
7.638
7.633
7.630
7.626
7.617
7.612
7.608
7.599
7.504
7.491
7.484
7.481
7.476
7.473
7.470
7.467
7.465
7.459
7.453
7.448
7.442
7.433
7.429
7.426
7.383
7.380
7.378
7.374
7.362
7.359
7.345
7.341
7.338
7.330
7.325
7.323
7.319
7.316
7.312
7.309
7.307
7.304
7.302
7.299
7.260
3.448
3.431
1.191
1.182
1.176
1.162
Cl
O
O
N
Ph
Ph
1b
2.05
4.18
4.15
1.99
6.00
f1 (ppm)

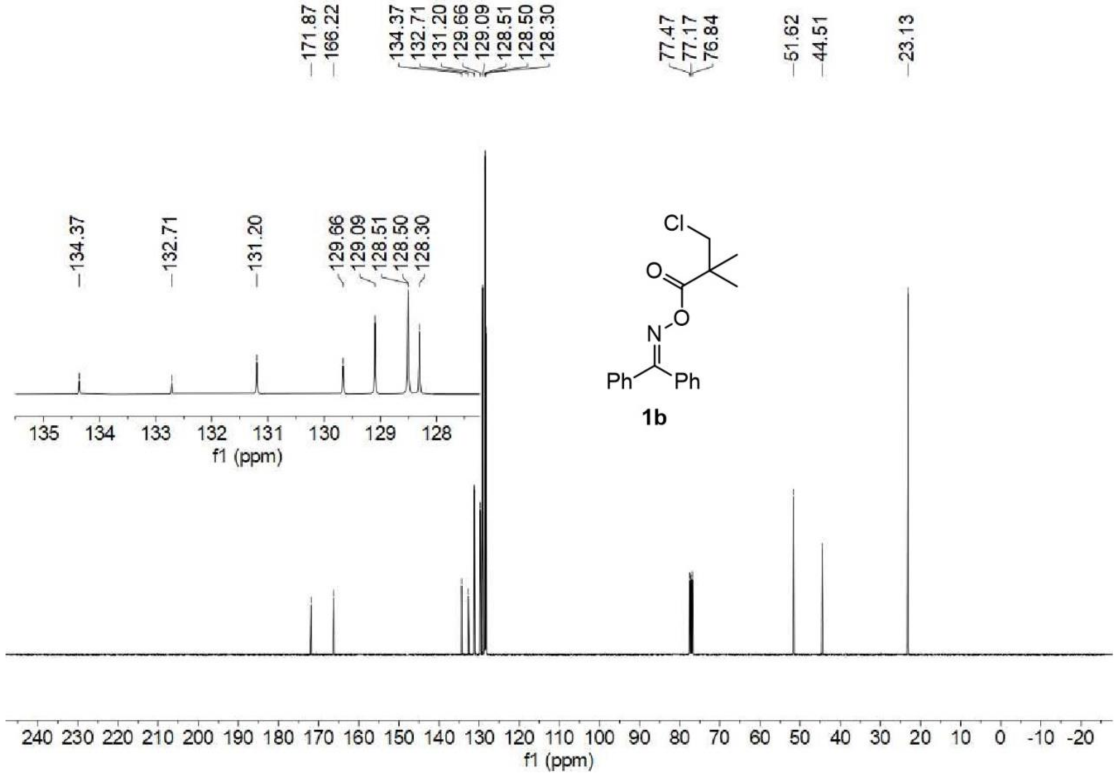
171.87
166.22
134.37
132.71
131.20
129.66
129.09
128.51
128.50
128.30
77.47
77.17
76.84
51.62
44.51
23.13
Cl
O
O
N
Ph
Ph
1b
f1 (ppm)

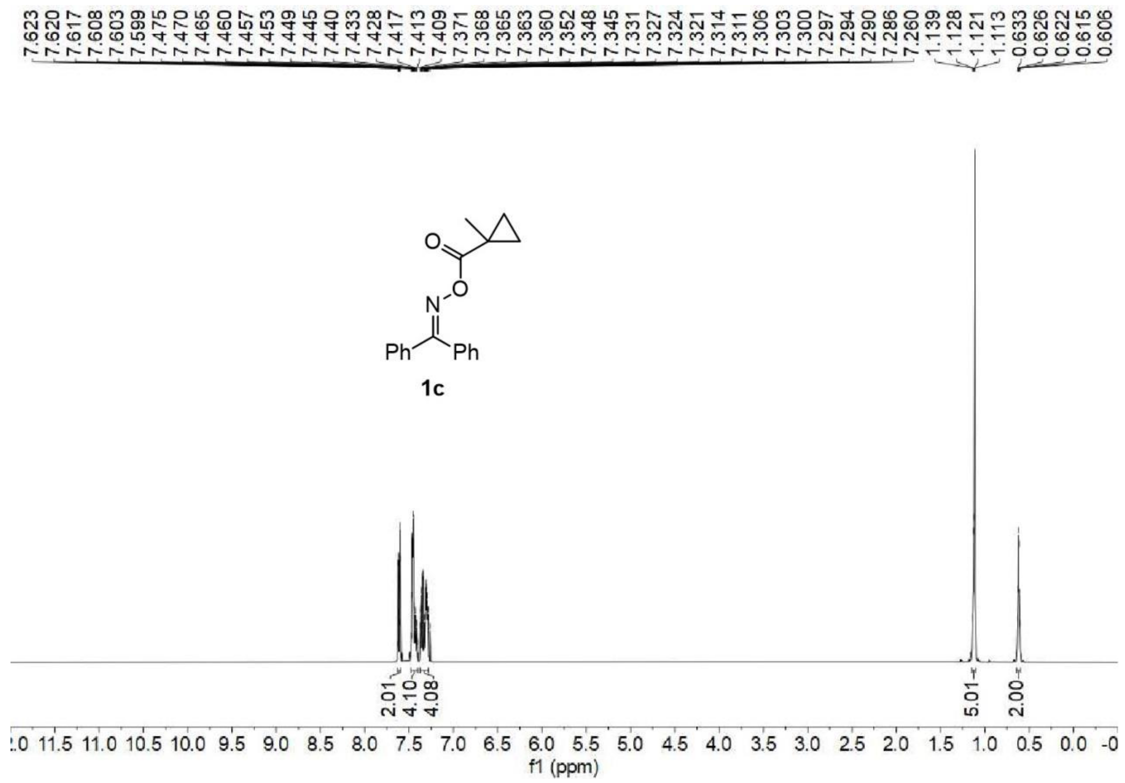
7.623 7.620 7.617 7.608 7.603 7.599 7.475 7.470 7.465 7.460 7.457 7.453 7.449 7.445 7.440 7.433 7.428 7.417 7.413 7.409 7.371 7.368 7.365 7.363 7.360 7.352 7.348 7.345 7.331 7.327 7.324 7.321 7.314 7.311 7.306 7.303 7.300 7.297 7.294 7.290 7.286 7.260 1.139 1.128 1.121 1.113 0.633 0.626 0.622 0.615 0.606
O
O
N
Ph
Ph
1c
2.01
4.10
4.08
5.01
2.00
f1 (ppm)

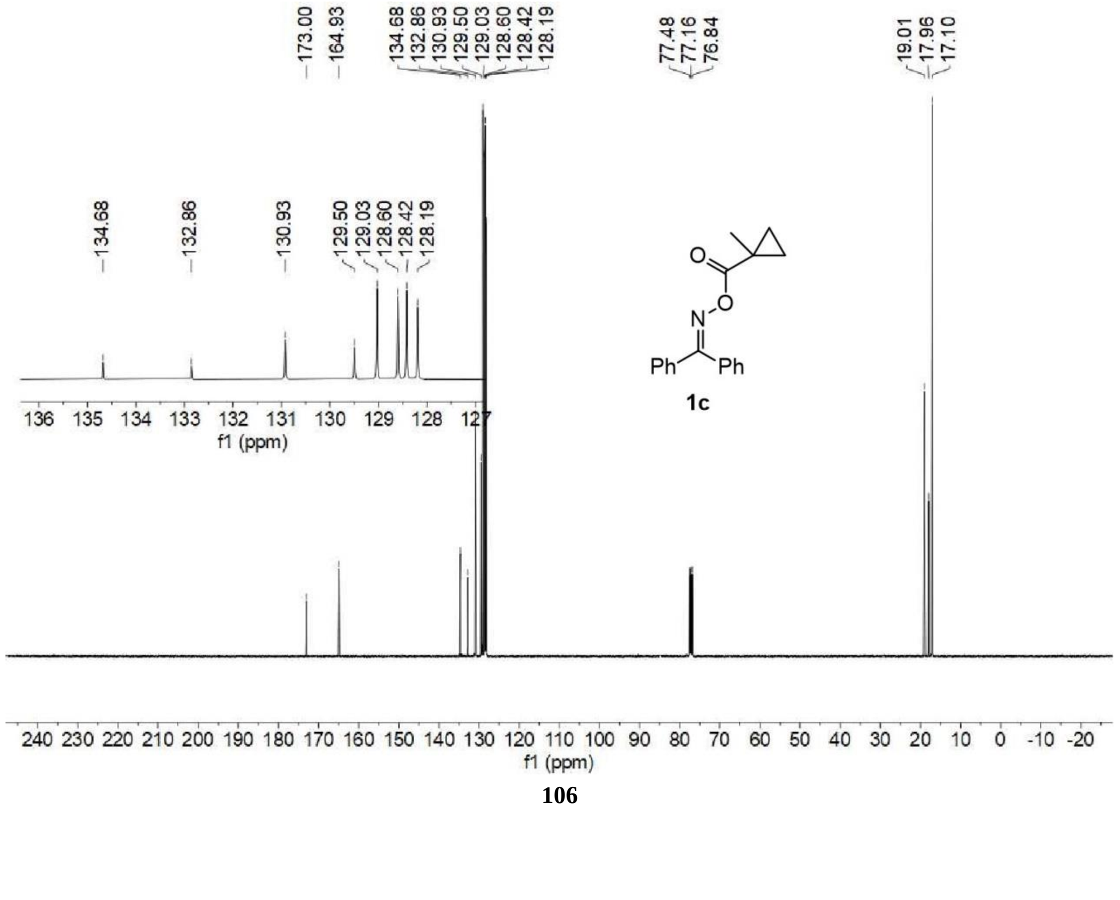
173.00
164.93
134.68 132.86 130.93 129.50 129.03 128.60 128.42 128.19
77.48 77.16 76.84
19.01 17.96 17.10
134.68
132.86
130.93
129.50 129.03 128.60 128.42 128.19
f1 (ppm)
O
O
N
Ph
Ph
1c
f1 (ppm)

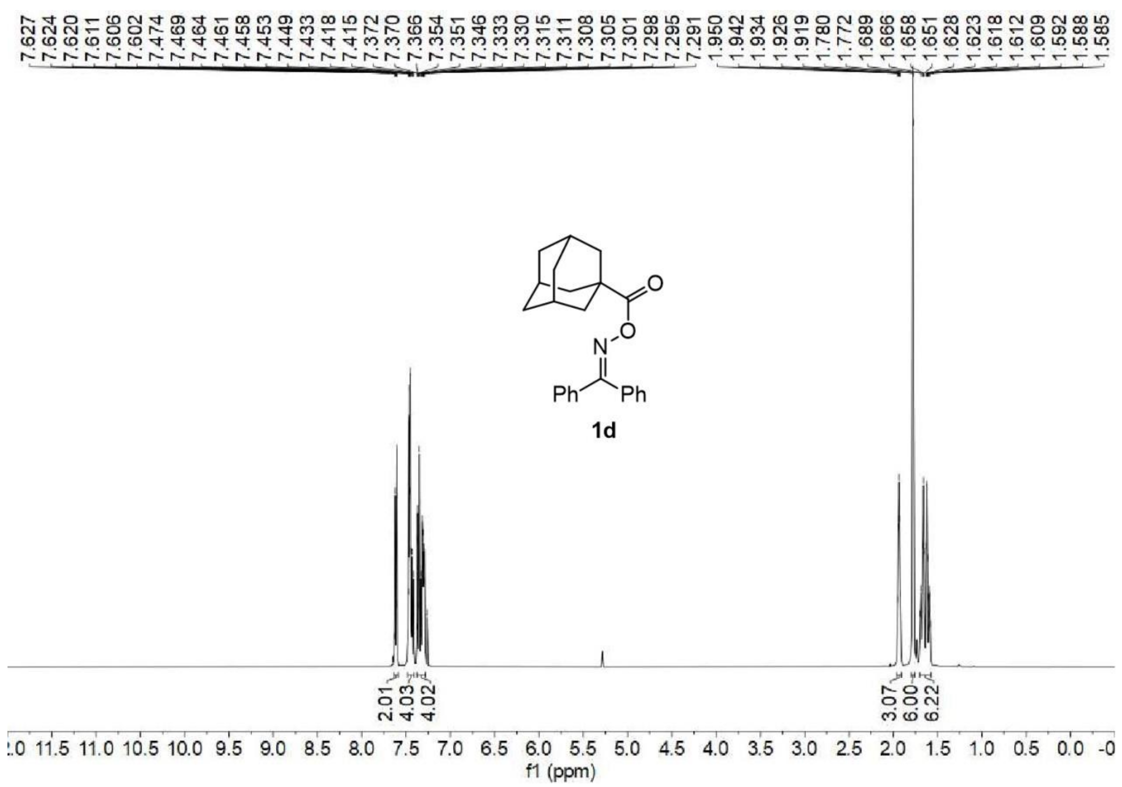
7.627 7.624 7.620 7.611 7.606 7.602 7.474 7.469 7.464 7.461 7.458 7.453 7.449 7.433 7.418 7.415 7.372 7.370 7.366 7.354 7.351 7.346 7.333 7.330 7.315 7.311 7.308 7.305 7.301 7.298 7.295 7.291 1.950 1.942 1.934 1.926 1.919 1.780 1.772 1.689 1.666 1.658 1.651 1.628 1.623 1.618 1.612 1.609 1.592 1.588 1.585
O
N
O
Ph
Ph
1d
2.01 4.03 4.02
3.07 6.00 6.22
f1 (ppm)

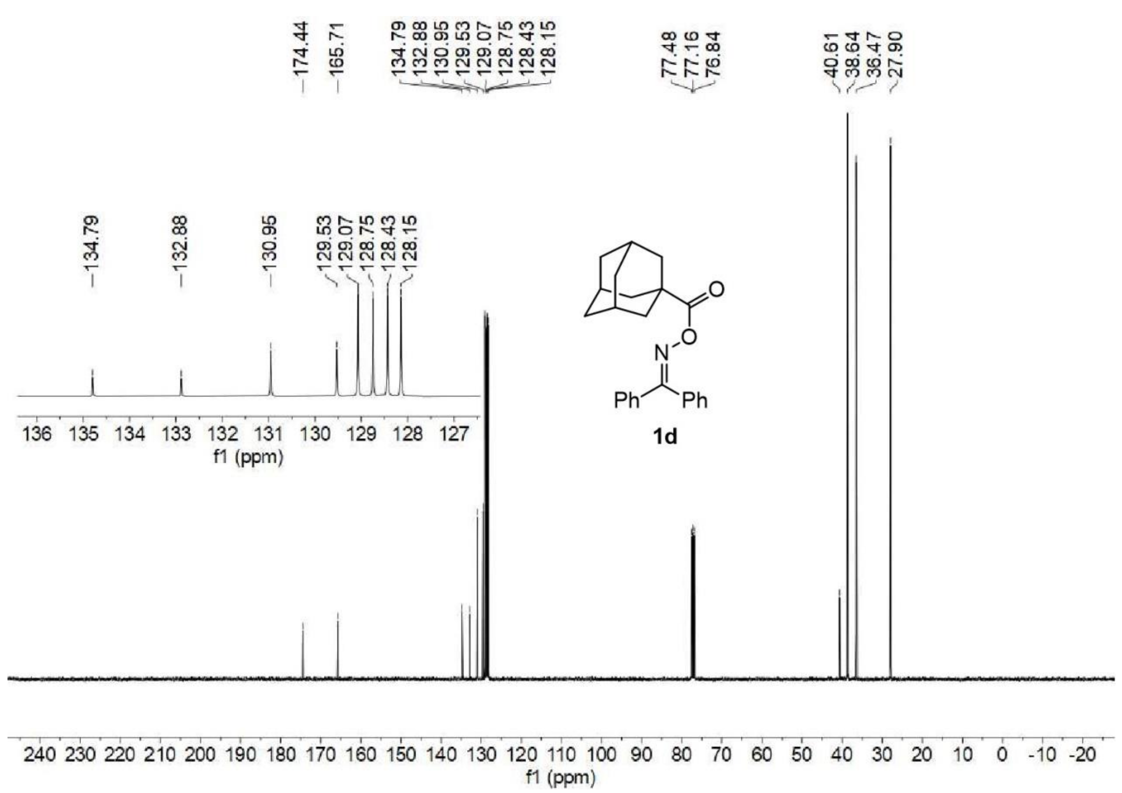
174.44 165.71 134.79 132.88 130.95 129.53 129.07 128.75 128.43 128.15 77.48 77.16 76.84 40.61 38.64 36.47 27.90
134.79 132.88 130.95 129.53 129.07 128.75 128.43 128.15
f1 (ppm)
O
N
O
Ph
Ph
1d
f1 (ppm)

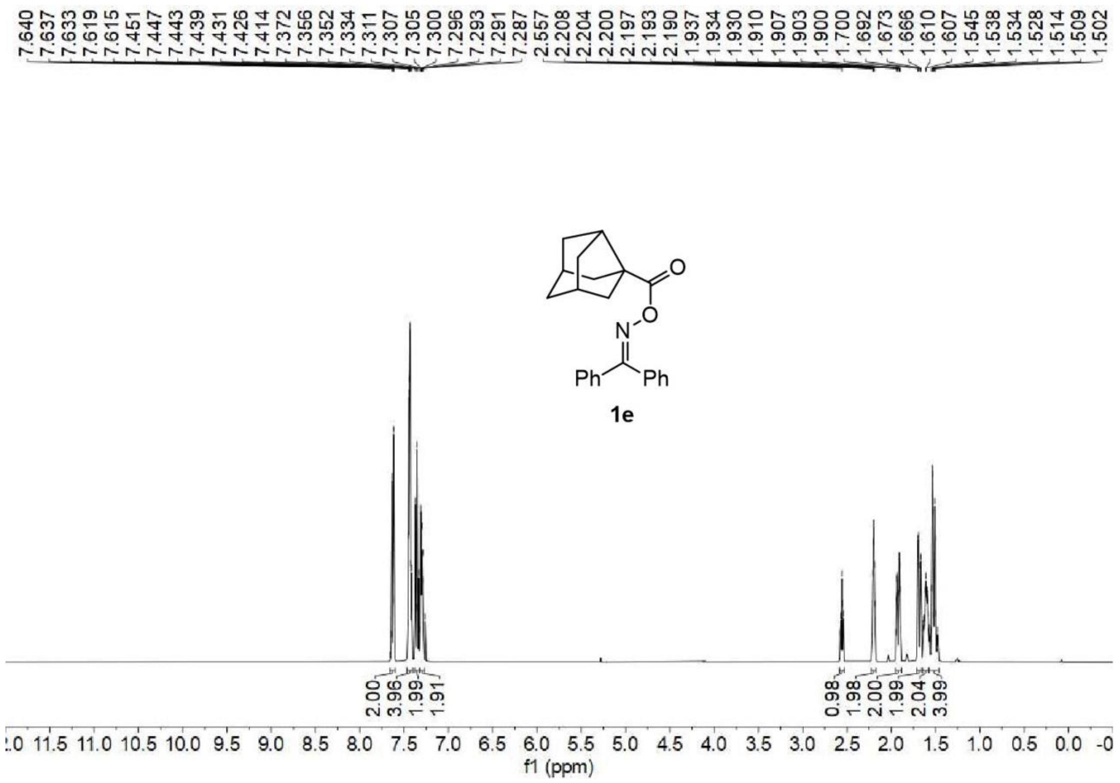
7.640 7.637 7.633 7.619 7.615 7.451 7.447 7.443 7.439 7.431 7.426 7.414 7.372 7.356 7.352 7.334 7.311 7.307 7.305 7.300 7.296 7.293 7.291 7.287 2.557 2.208 2.204 2.200 2.197 2.193 2.190 1.937 1.934 1.930 1.910 1.907 1.903 1.900 1.700 1.692 1.673 1.666 1.610 1.607 1.545 1.538 1.534 1.528 1.514 1.509 1.502
O
O
N
Ph
Ph
1e
2.00 3.96 1.99 1.91
0.98 1.98 2.00 1.99 2.04 3.99
f1 (ppm)

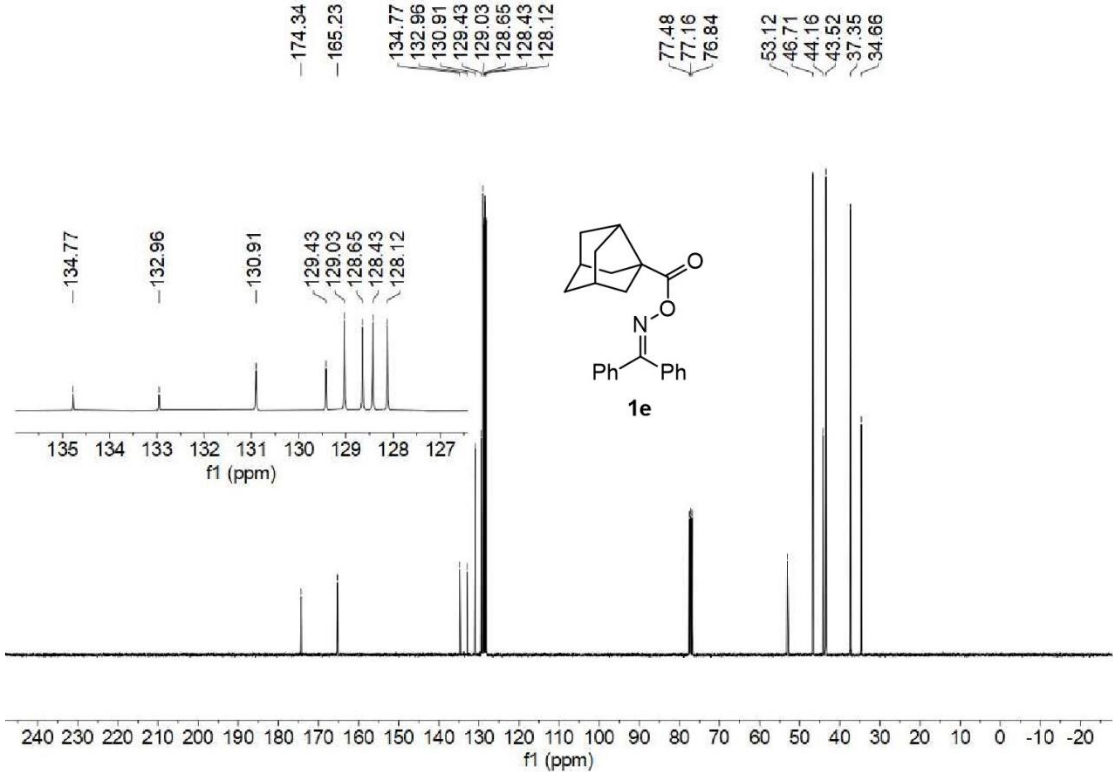
174.34 165.23 134.77 132.96 130.91 129.43 129.03 128.65 128.43 128.12 77.48 77.16 76.84 53.12 46.71 44.16 43.52 37.35 34.66
O
O
N
Ph
Ph
1e
f1 (ppm)
f1 (ppm)

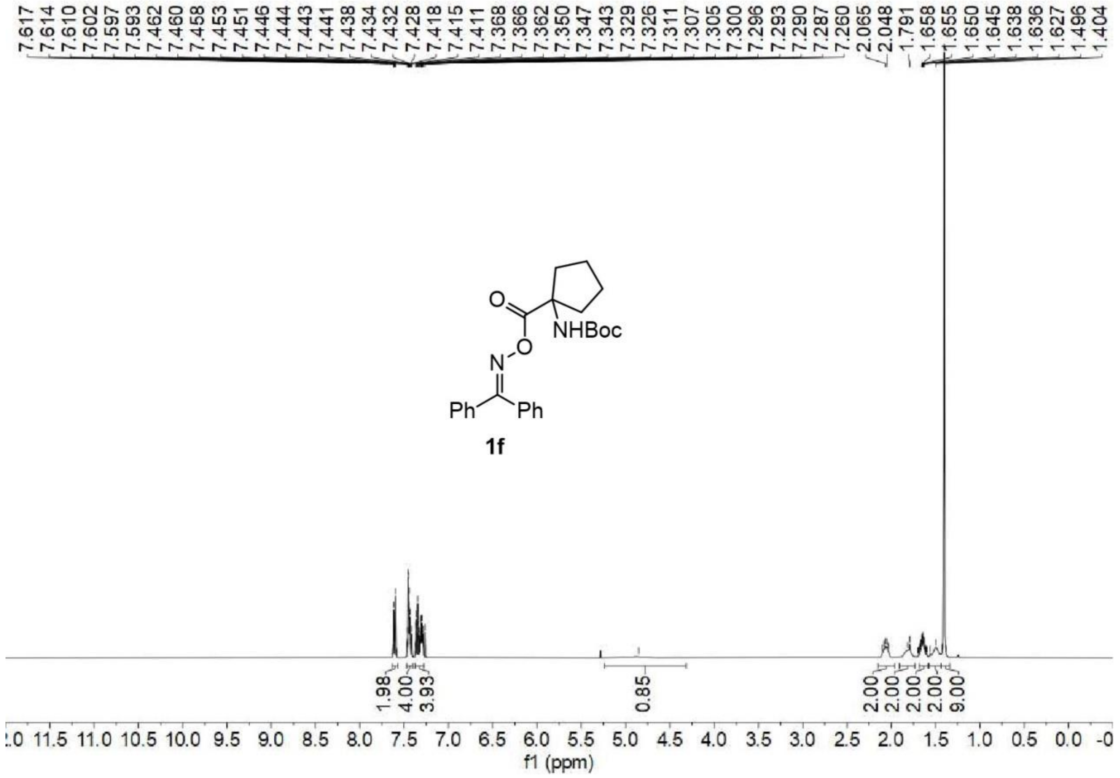
7.617 7.614 7.610 7.602 7.597 7.593 7.462 7.460 7.458 7.453 7.451 7.446 7.444 7.443 7.441 7.438 7.434 7.432 7.428 7.418 7.415 7.411 7.368 7.366 7.362 7.350 7.347 7.343 7.329 7.326 7.311 7.307 7.305 7.300 7.296 7.293 7.290 7.287 7.260 2.065 2.048 1.791 1.658 1.655 1.650 1.645 1.638 1.636 1.627 1.496 1.404
O
NHBoc
N
O
Ph
Ph
1f
1.98
4.00
3.93
0.85
2.00
2.00
2.00
2.00
9.00
f1 (ppm)

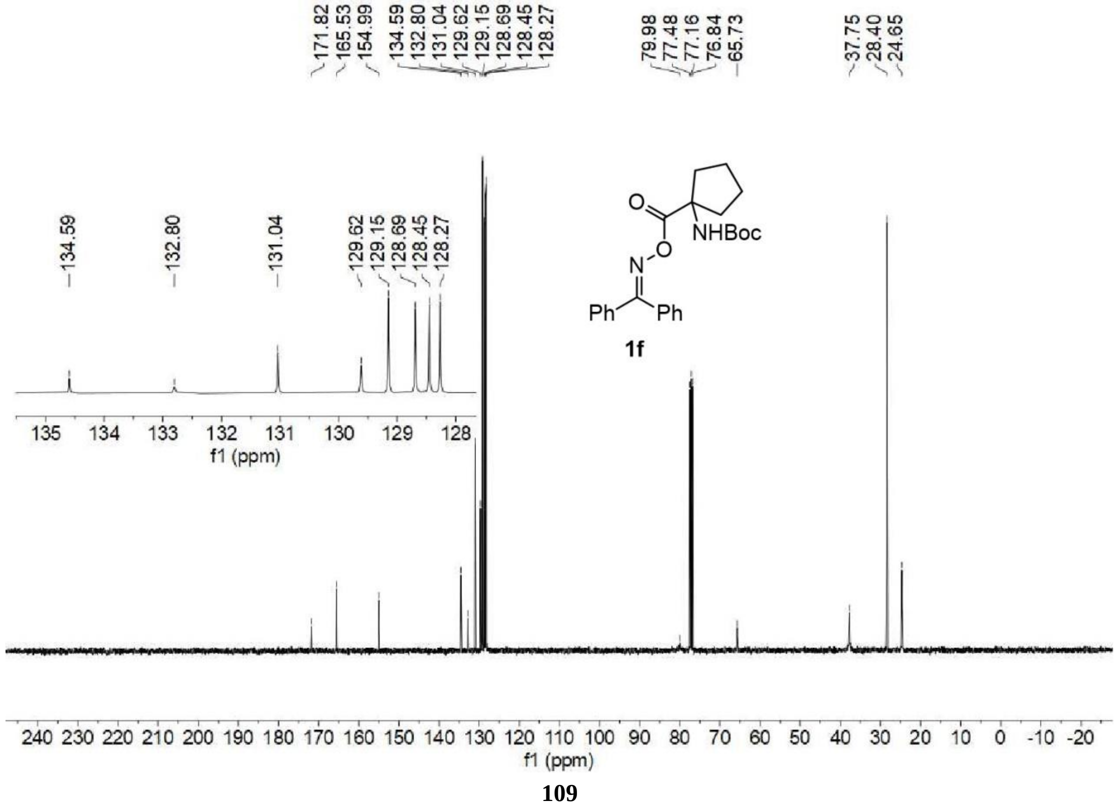
171.82 165.53 154.99 134.59 132.80 131.04 129.62 129.15 128.69 128.45 128.27
79.98 77.48 77.16 76.84 65.73
37.75 28.40 24.65
134.59
132.80
131.04
129.62 129.15 128.69 128.45 128.27
f1 (ppm)
O
NHBoc
N
O
Ph
Ph
1f
f1 (ppm)

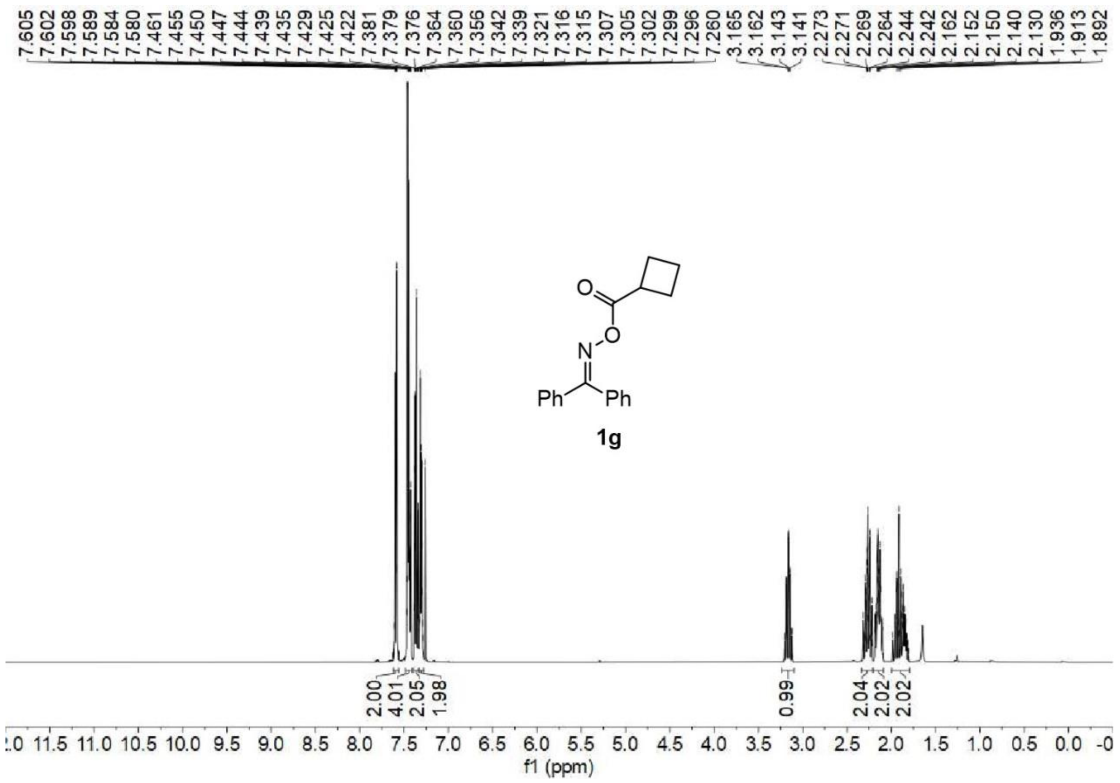
7.605 7.602 7.598 7.589 7.584 7.580 7.461 7.455 7.450 7.447 7.444 7.439 7.435 7.429 7.425 7.422 7.381 7.379 7.376 7.364 7.360 7.356 7.342 7.339 7.321 7.316 7.315 7.307 7.305 7.302 7.299 7.296 7.260 3.165 3.162 3.143 3.141 2.273 2.271 2.269 2.264 2.244 2.242 2.162 2.152 2.150 2.140 2.130 1.936 1.913 1.892
Ph
Ph
N
O
O
1g
2.00
4.01
2.05
1.98
0.99
2.04
2.02
2.02
f1 (ppm)

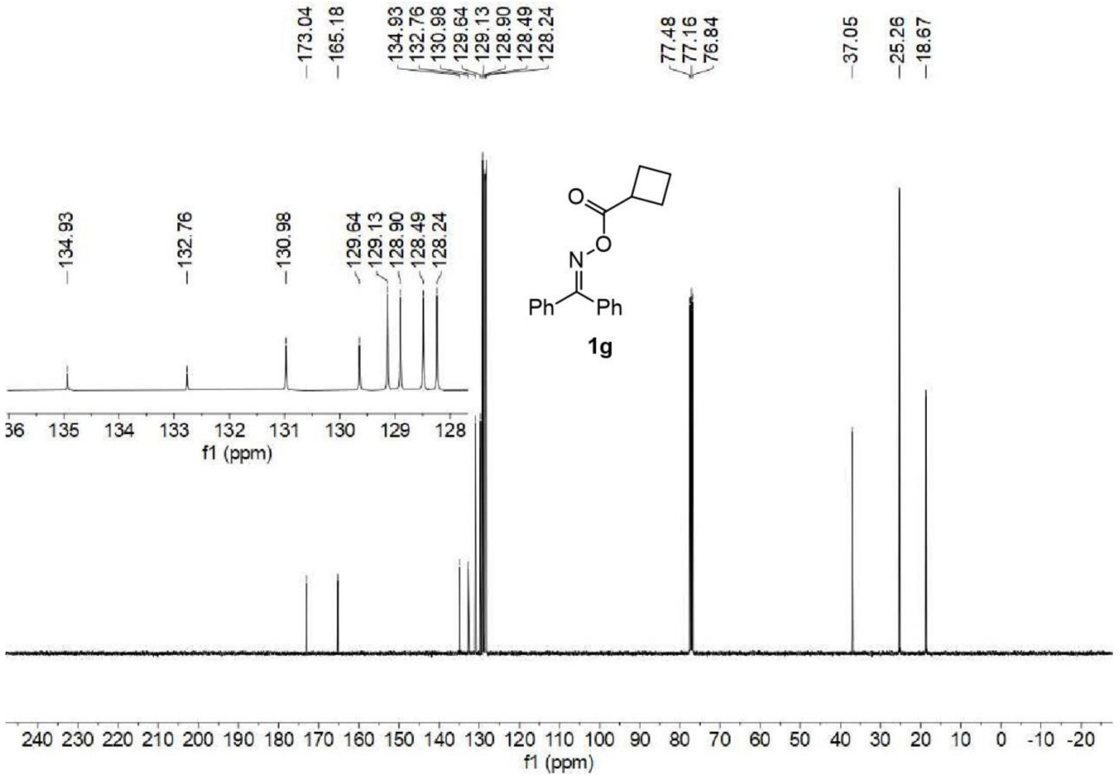
173.04 165.18 134.93 132.76 130.98 129.64 129.13 128.90 128.49 128.24 77.48 77.16 76.84 37.05 25.26 18.67
Ph
Ph
N
O
O
1g
f1 (ppm)
f1 (ppm)

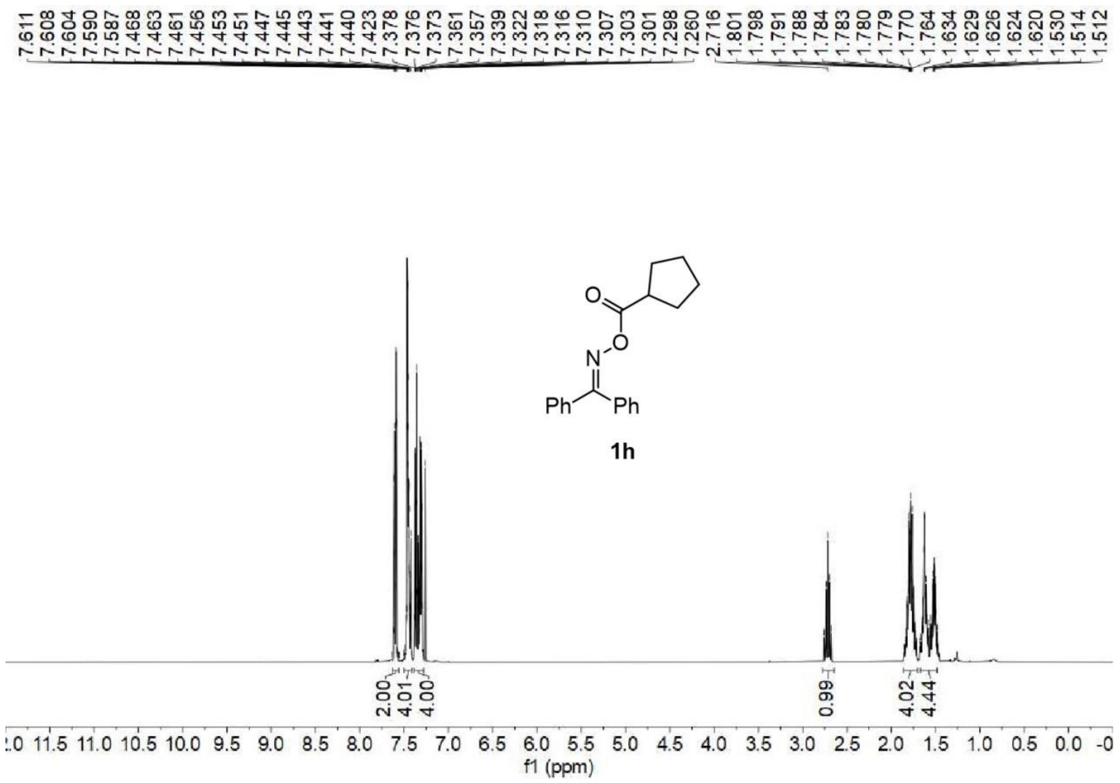

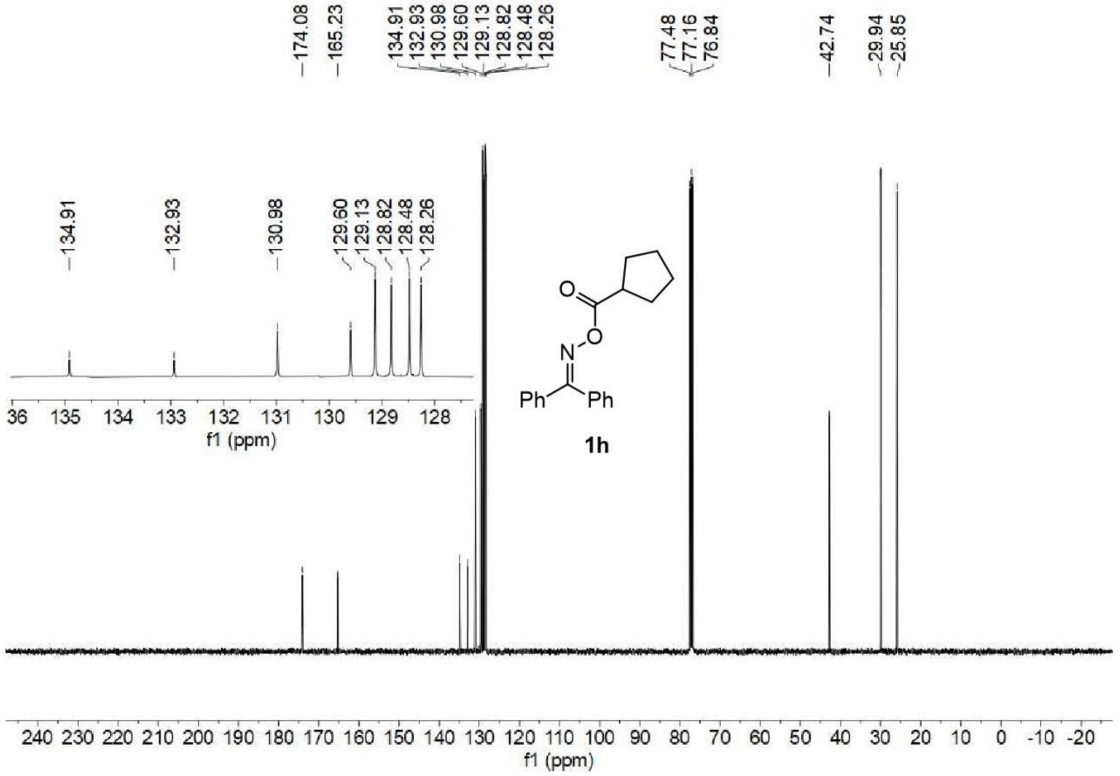

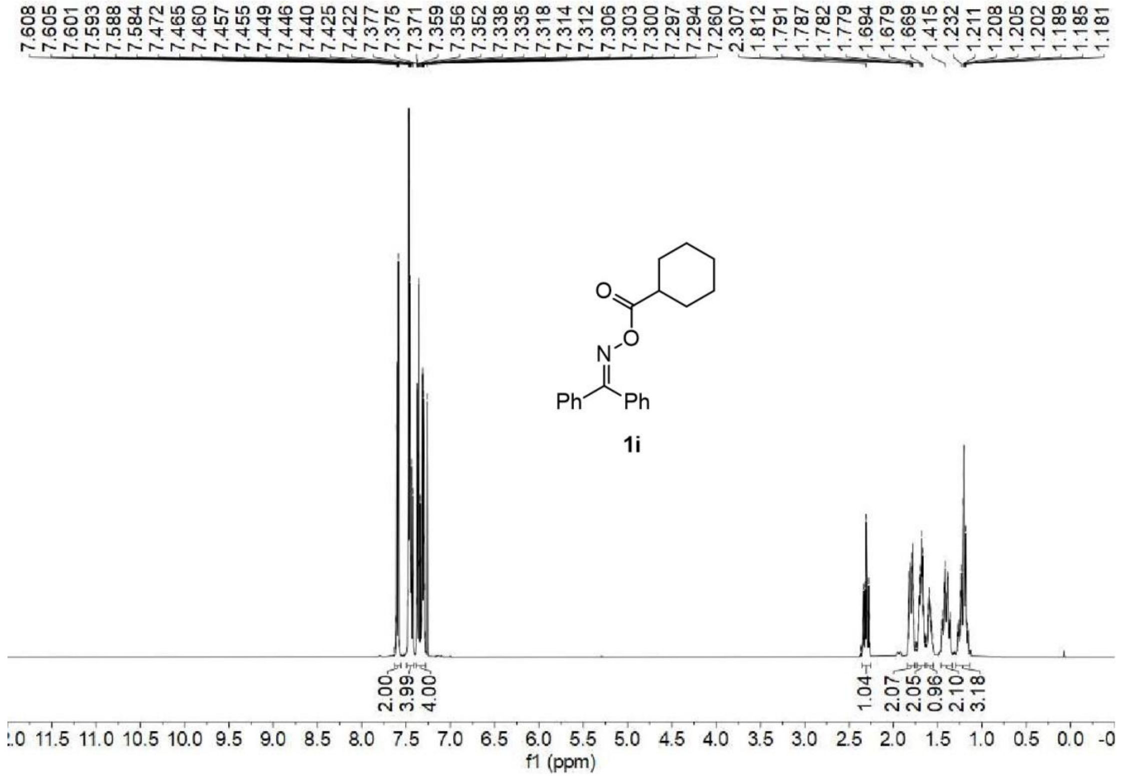
7.608 7.605 7.601 7.593 7.588 7.584 7.472 7.465 7.460 7.457 7.455 7.449 7.446 7.440 7.425 7.422 7.377 7.375 7.371 7.359 7.356 7.352 7.338 7.335 7.318 7.314 7.312 7.306 7.303 7.300 7.297 7.294 7.260 2.307 1.812 1.791 1.787 1.782 1.779 1.694 1.679 1.669 1.415 1.232 1.211 1.208 1.205 1.202 1.189 1.185 1.181
O
O
N
Ph
Ph
1i
2.00 3.99 4.00
1.04 2.07 2.05 0.96 2.10 3.18
f1 (ppm)

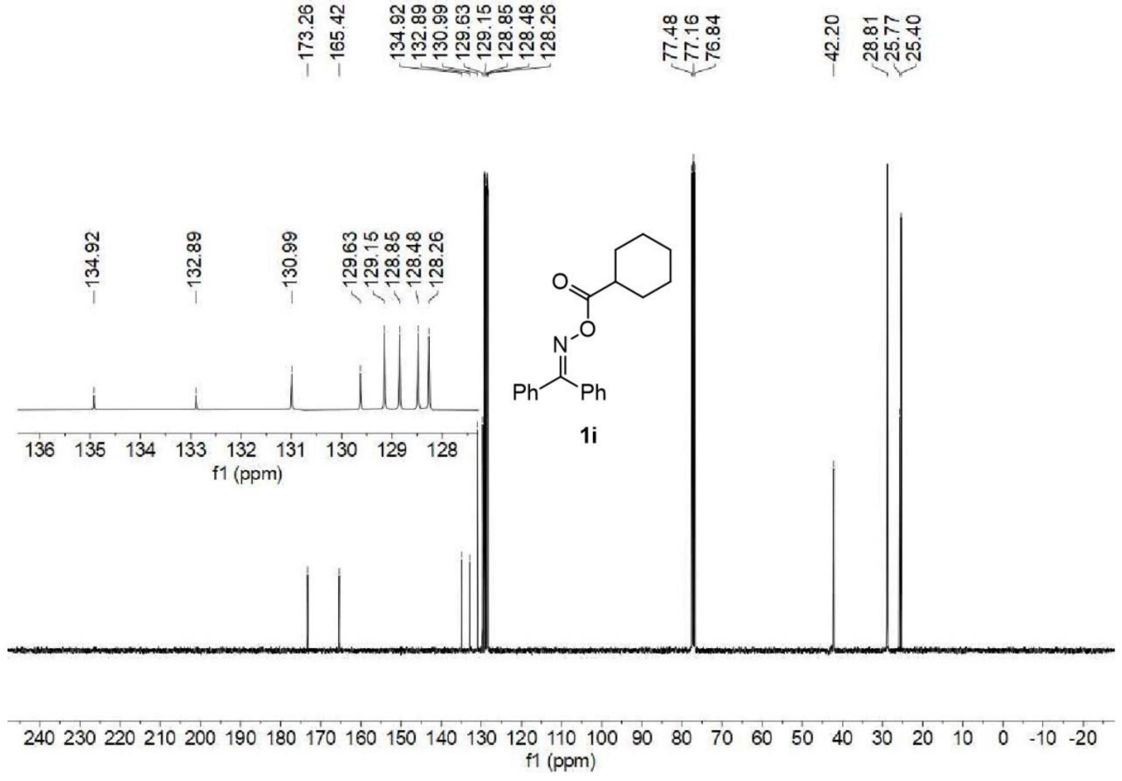
173.26
165.42
134.92 132.89 130.99 129.63 129.15 128.85 128.48 128.26
77.48 77.16 76.84
42.20
28.81 25.77 25.40
O
O
N
Ph
Ph
1i
f1 (ppm)
f1 (ppm)

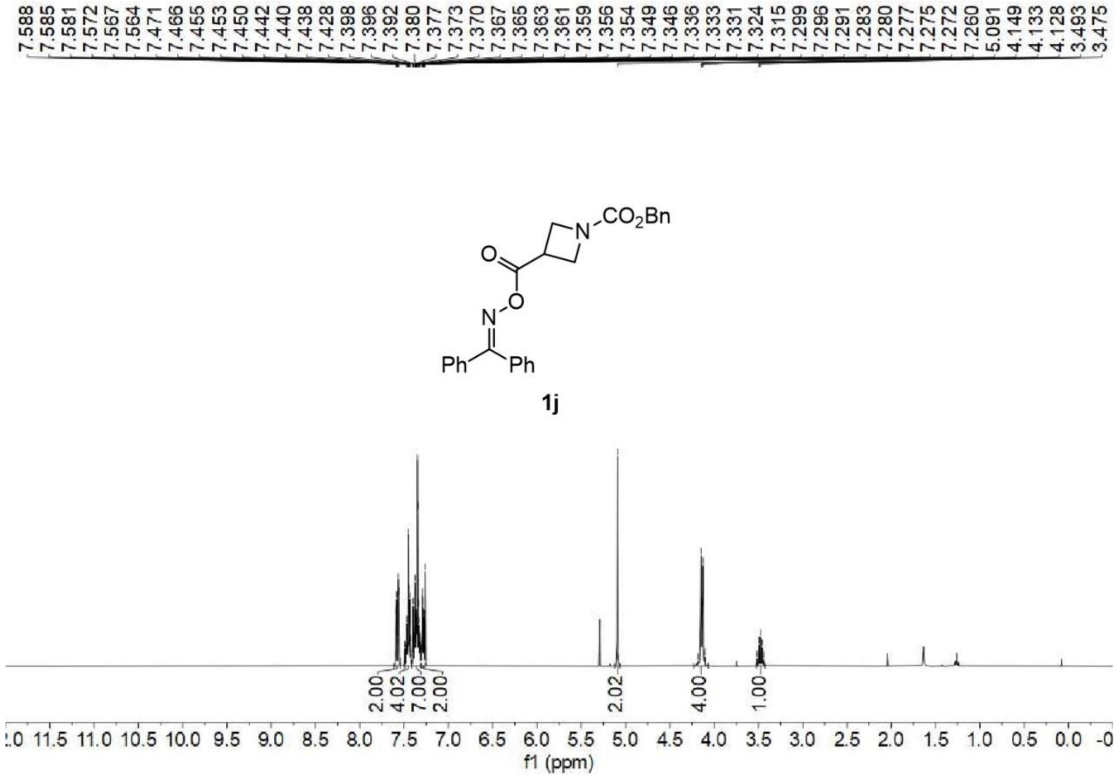
7.588 7.585 7.581 7.572 7.567 7.564 7.471 7.466 7.455 7.453 7.450 7.442 7.440 7.438 7.428 7.398 7.396 7.392 7.380 7.377 7.373 7.370 7.367 7.365 7.363 7.361 7.359 7.356 7.354 7.349 7.346 7.336 7.333 7.331 7.324 7.315 7.299 7.296 7.291 7.283 7.280 7.277 7.275 7.272 7.260 5.091 4.149 4.133 4.128 3.493 3.475
CO2Bn
N
O
O
N
Ph
Ph
1j
2.00
4.02
7.00
2.00
2.02
4.00
1.00
f1 (ppm)

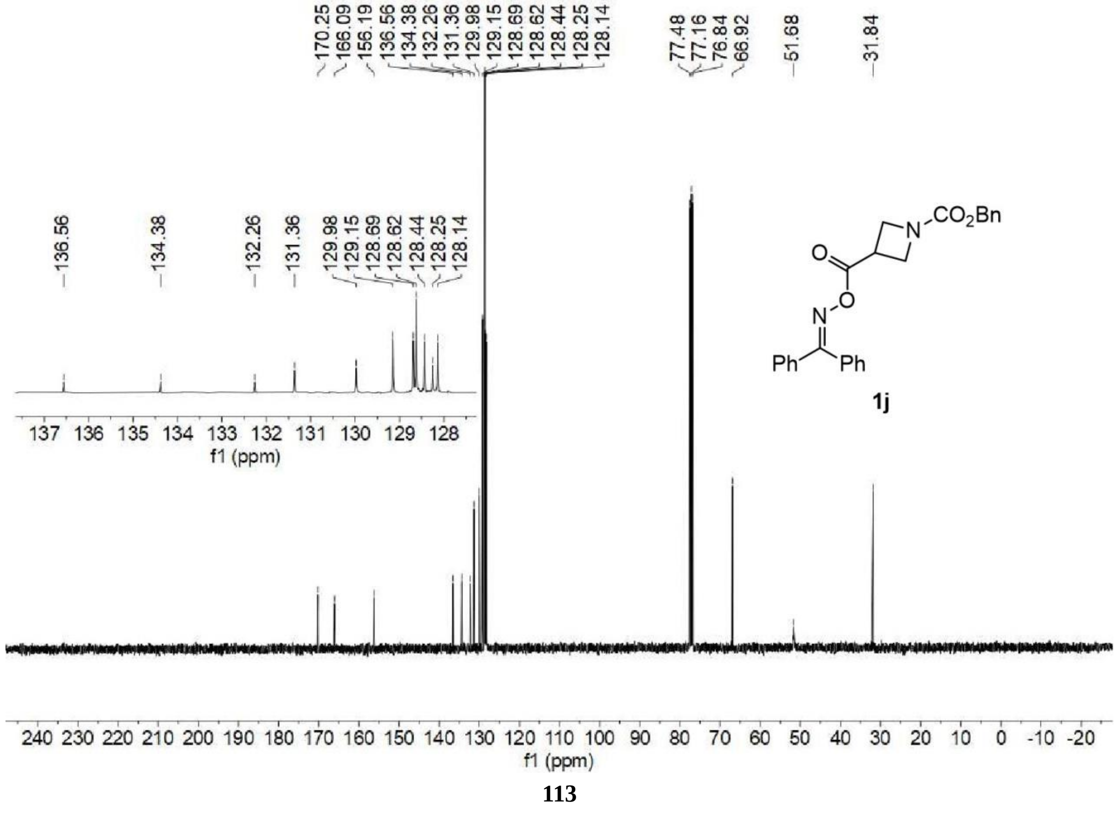
170.25 166.09 156.19 136.56 134.38 132.26 131.36 129.98 129.15 128.69 128.62 128.44 128.25 128.14
77.48 77.16 76.84 66.92
51.68
31.84
136.56
134.38
132.26
131.36
129.98 129.15 128.69 128.62 128.44 128.25 128.14
f1 (ppm)
CO2Bn
N
O
O
N
Ph
Ph
1j
f1 (ppm)

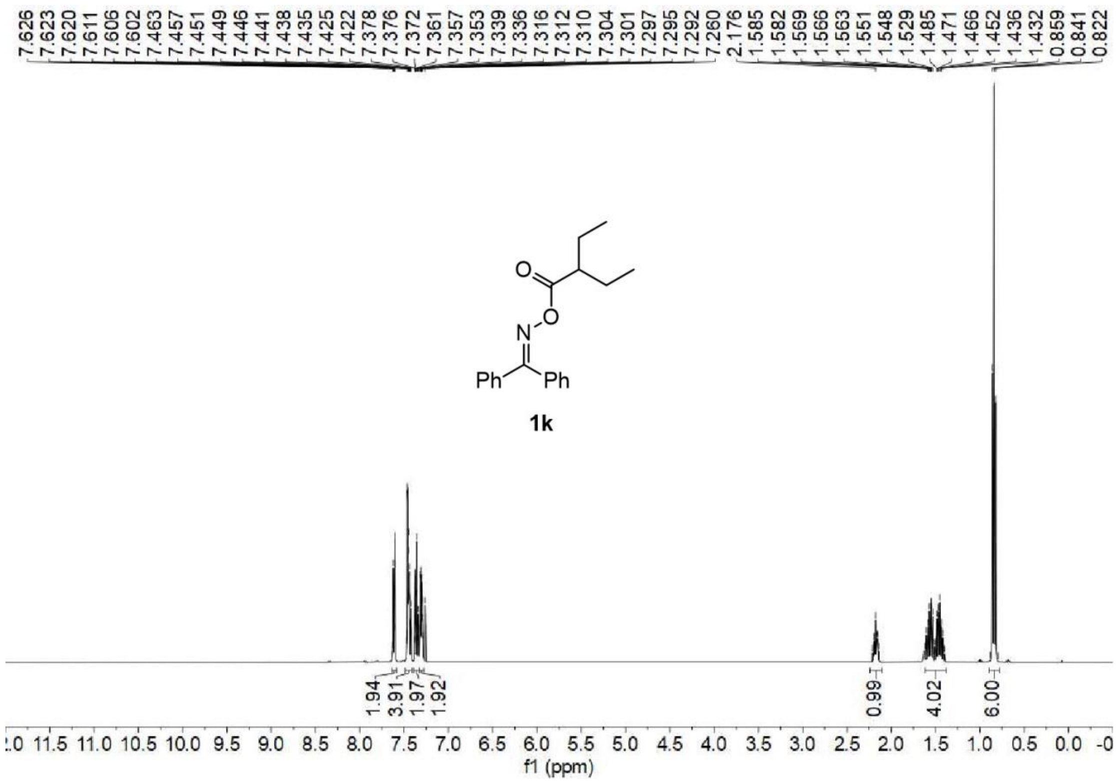

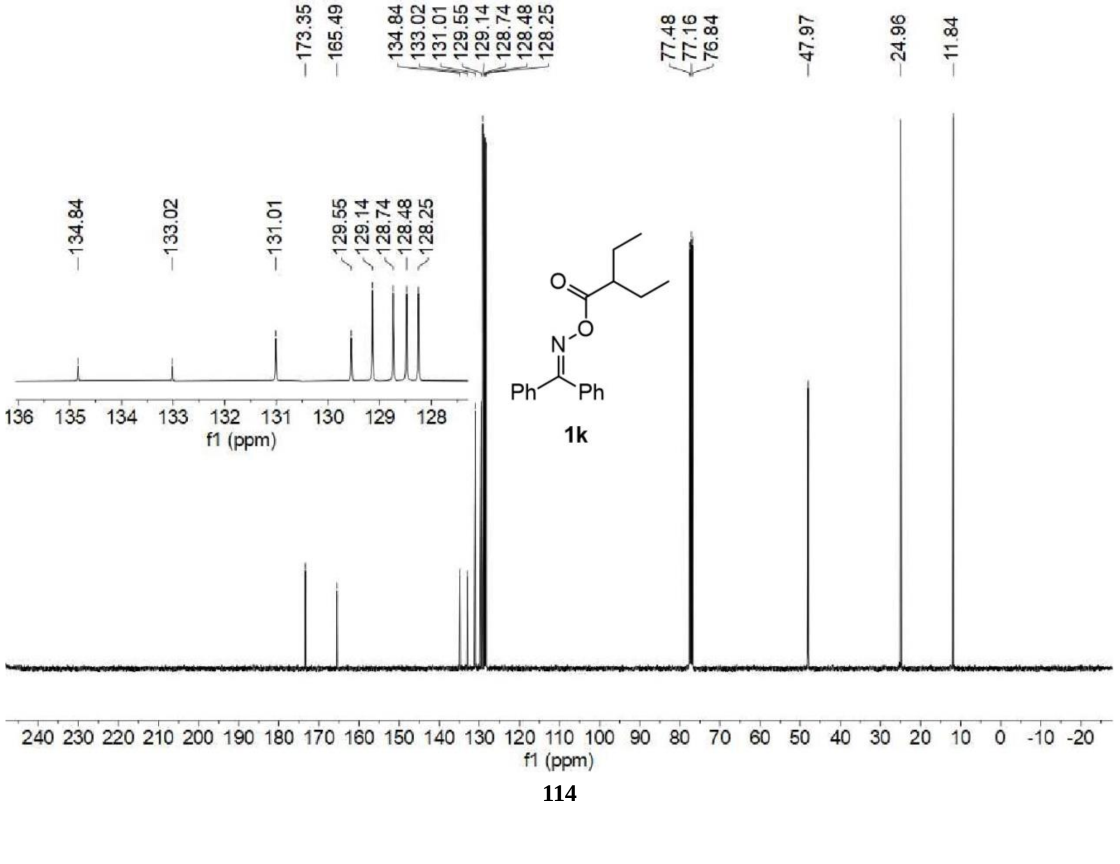

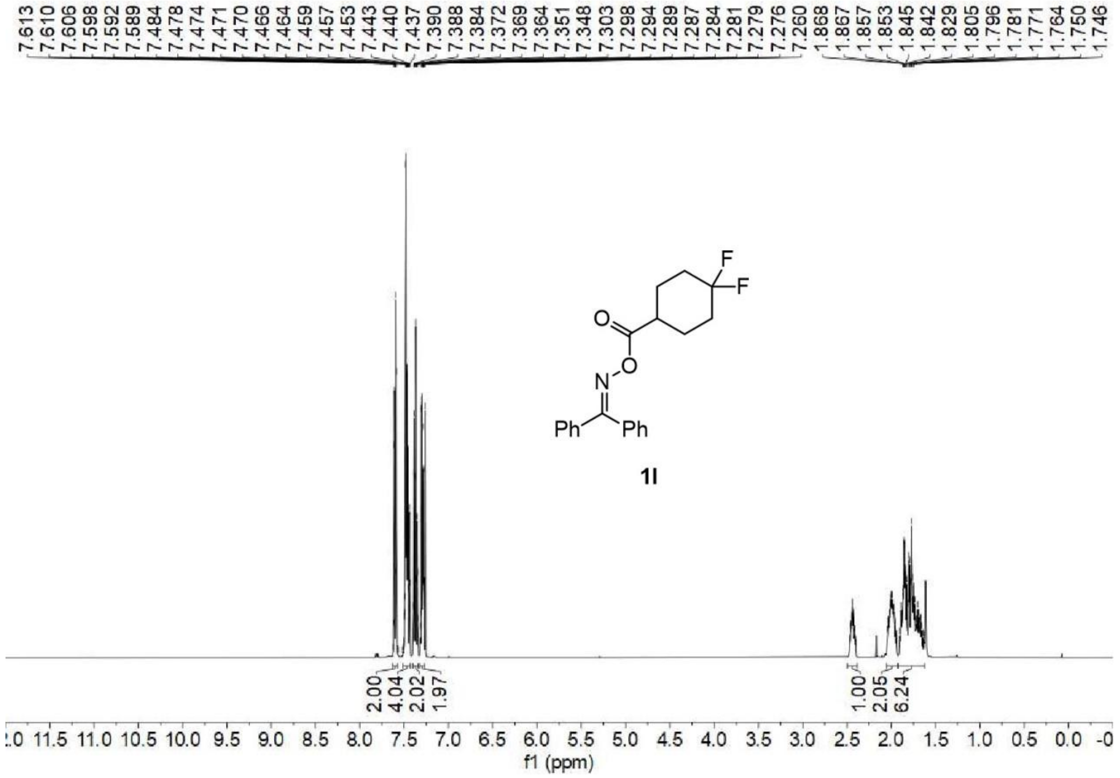
7.613 7.610 7.606 7.598 7.592 7.589 7.484 7.478 7.474 7.471 7.470 7.466 7.464 7.459 7.457 7.453 7.443 7.440 7.437 7.390 7.388 7.384 7.372 7.369 7.364 7.351 7.348 7.303 7.298 7.294 7.289 7.287 7.284 7.281 7.279 7.276 7.260 1.868 1.867 1.857 1.853 1.845 1.842 1.829 1.805 1.796 1.781 1.771 1.764 1.750 1.746
F
F
O
O
N
Ph
Ph
1l
2.00
4.04
2.02
1.97
1.00
2.05
6.24
f1 (ppm)

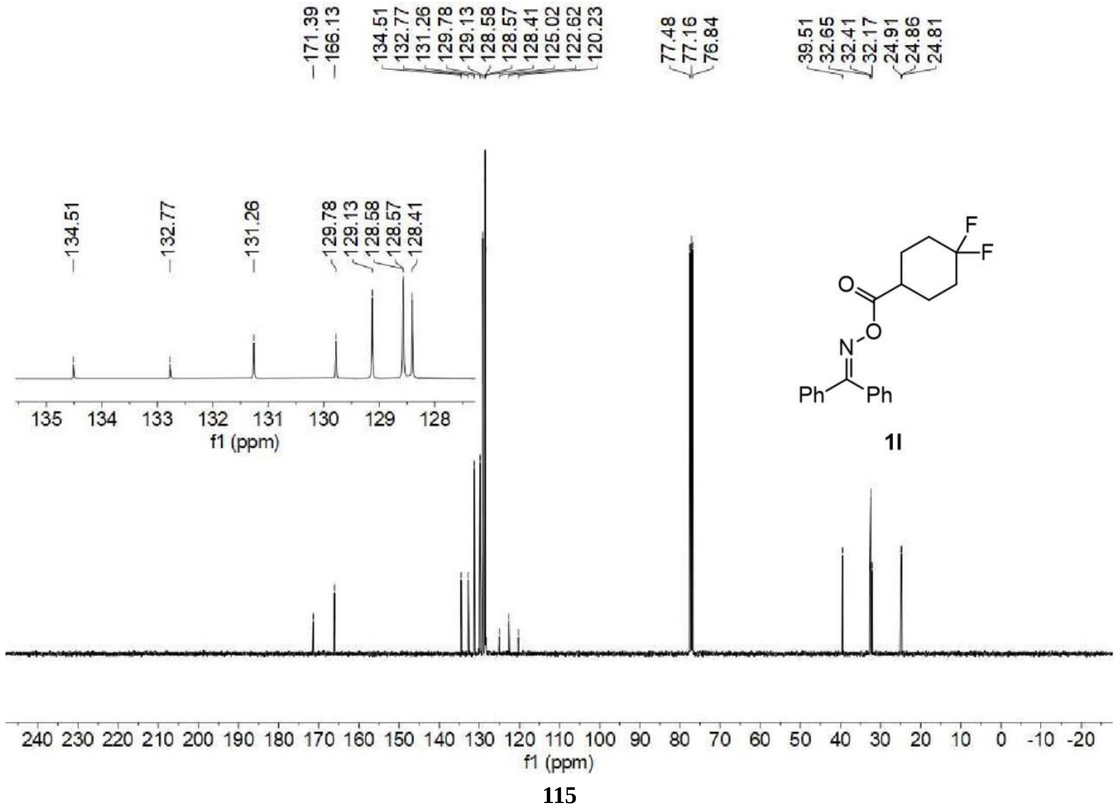
171.39
166.13
134.51 132.77 131.26 129.78 129.13 128.58 128.57 128.41 125.02 122.62 120.23
77.48 77.16 76.84
39.51 32.65 32.41 32.17 24.91 24.86 24.81
134.51
132.77
131.26
129.78 129.13 128.58 128.57 128.41
f1 (ppm)
F
F
O
O
N
Ph
Ph
1l
f1 (ppm)

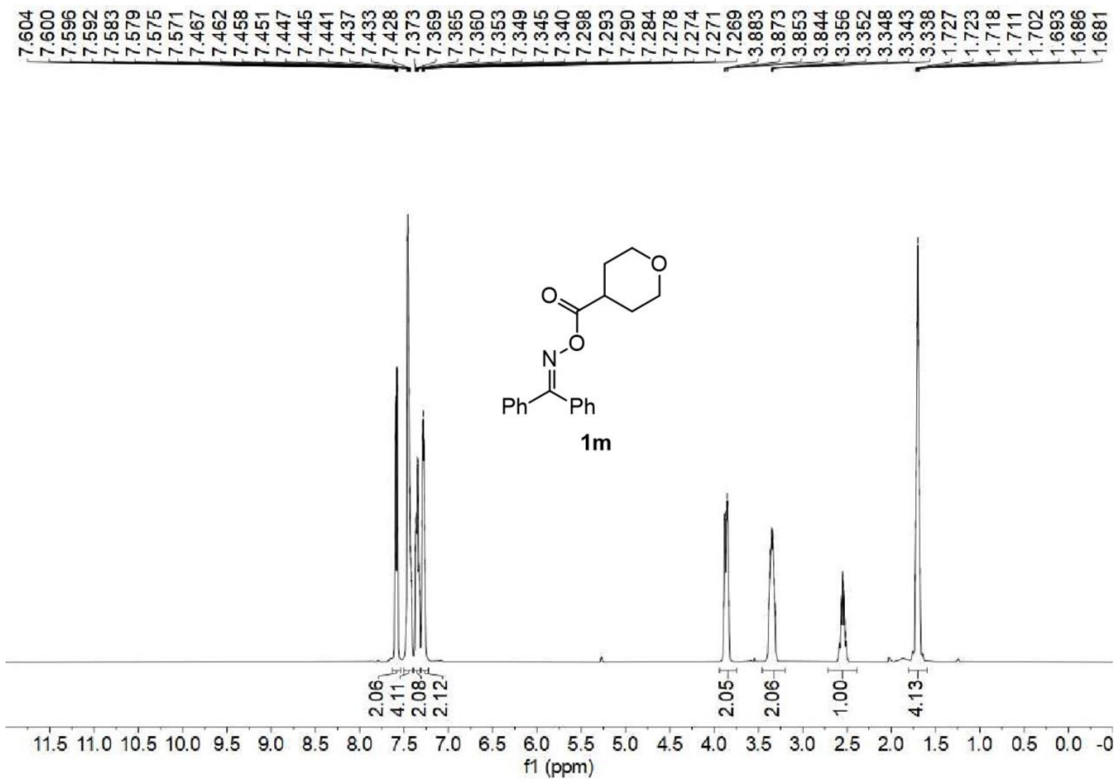

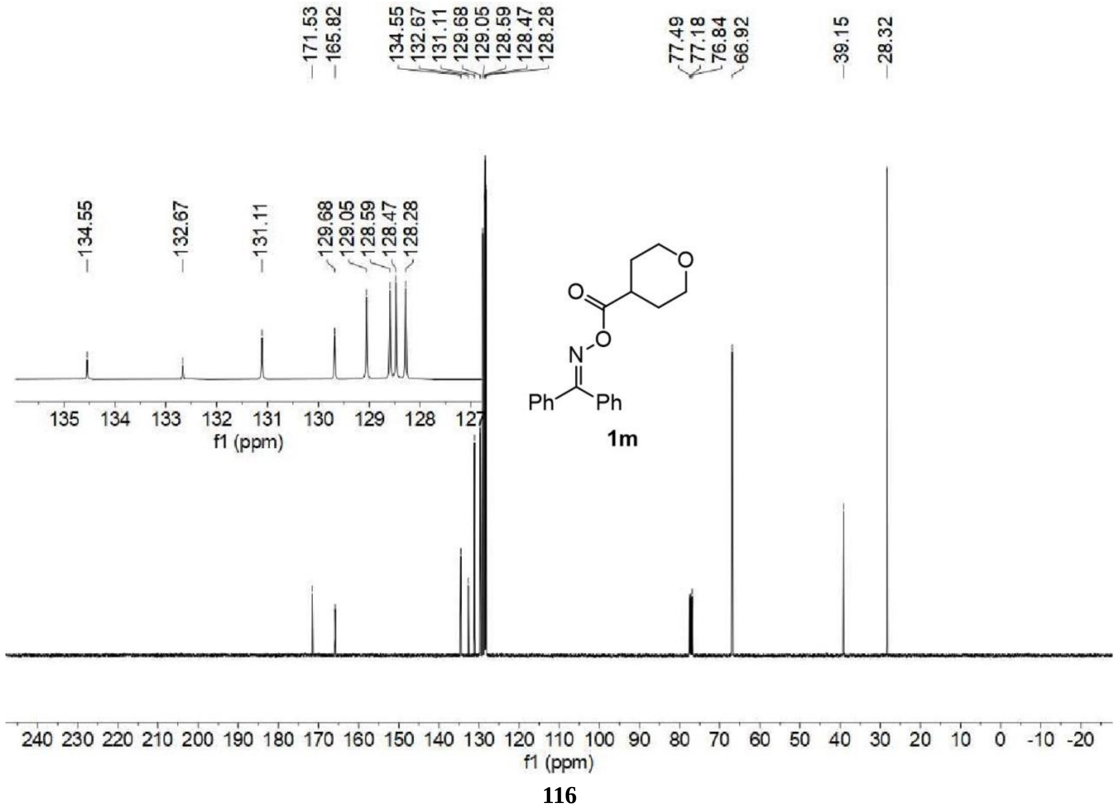

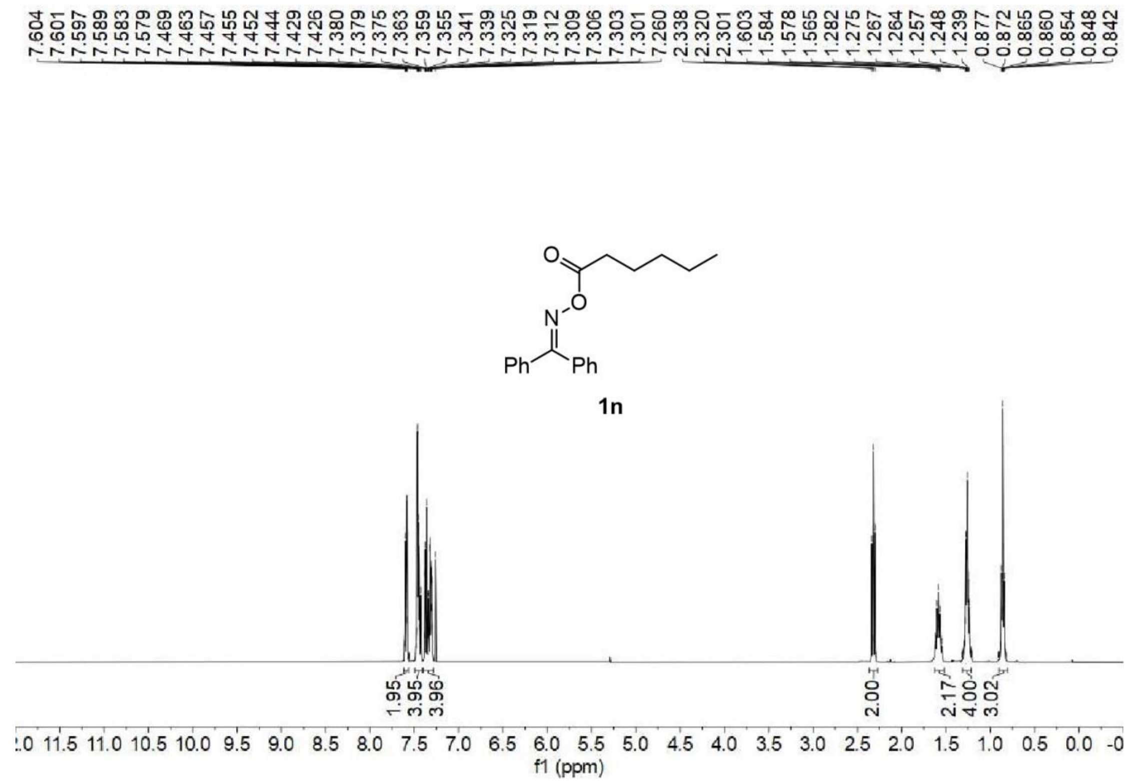

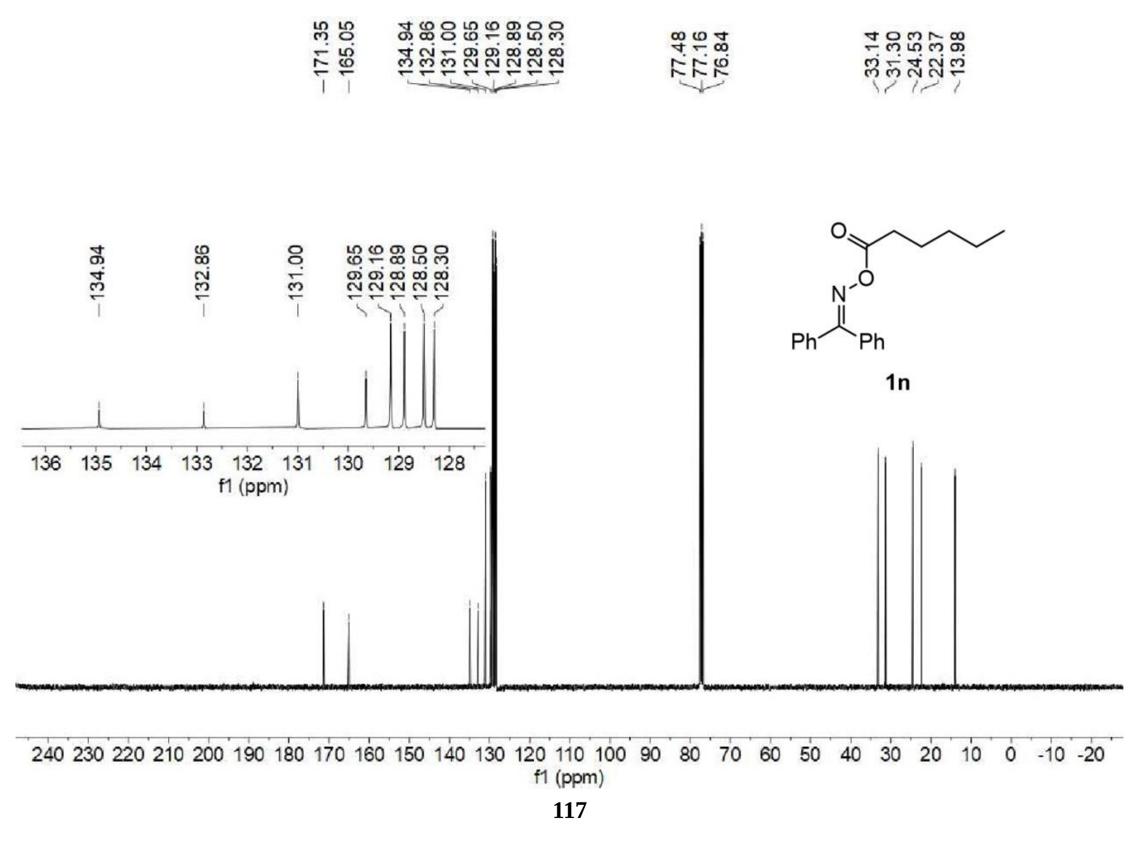

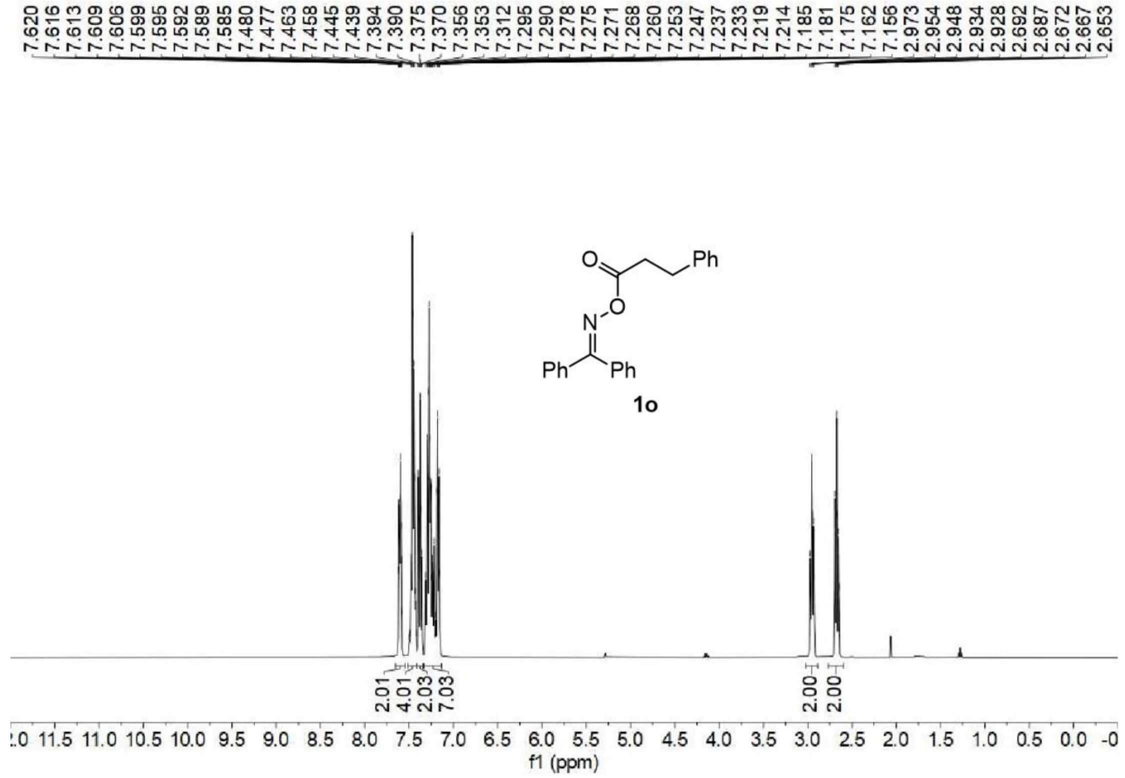

7.620 7.616 7.613 7.609 7.606 7.599 7.595 7.592 7.589 7.585 7.480 7.477 7.463 7.458 7.445 7.439 7.394 7.390 7.375 7.370 7.356 7.353 7.312 7.295 7.290 7.278 7.275 7.271 7.268 7.260 7.253 7.247 7.237 7.233 7.219 7.214 7.185 7.181 7.175 7.162 7.156 2.973 2.954 2.948 2.934 2.928 2.692 2.687 2.672 2.667 2.653
O
Ph
O
N
Ph
Ph
1o
2.01
4.01
2.03
7.03
2.00
2.00
f1 (ppm)

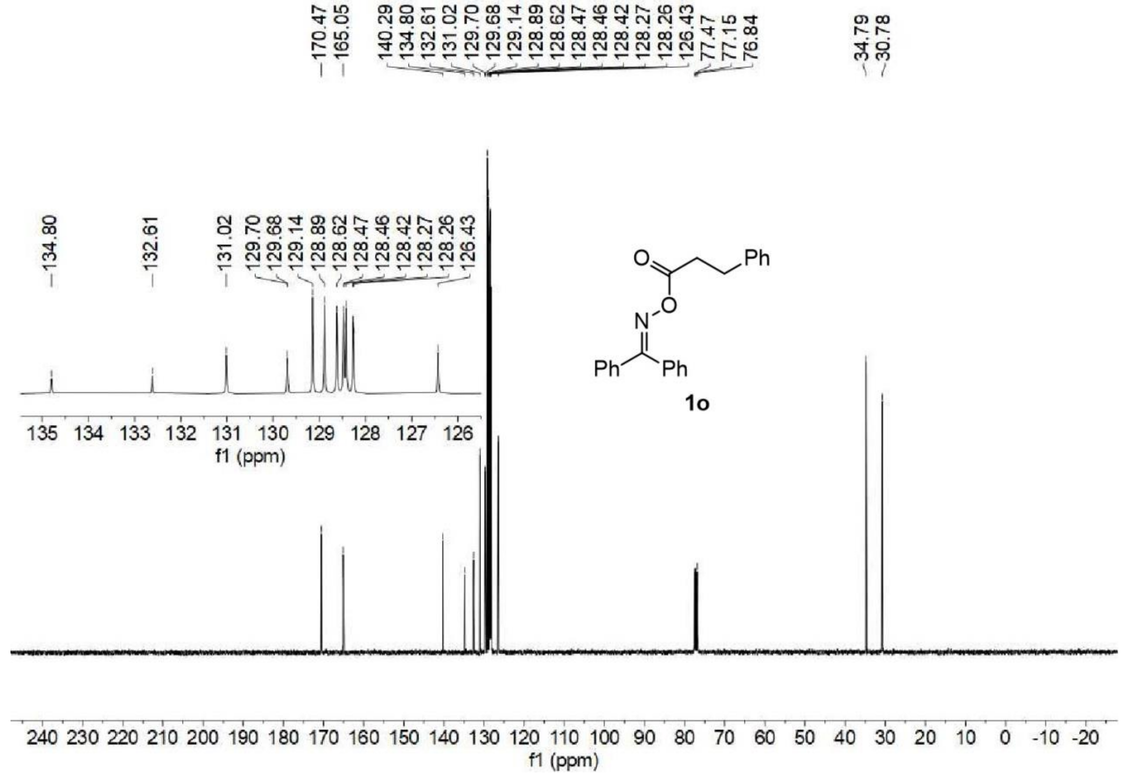

170.47 165.05 140.29 134.80 132.61 131.02 129.70 129.68 129.14 128.89 128.62 128.47 128.46 128.42 128.27 128.26 126.43 77.47 77.15 76.84 34.79 30.78
134.80 132.61 131.02 129.70 129.68 129.14 128.89 128.62 128.47 128.46 128.42 128.27 128.26 126.43
O
Ph
O
N
Ph
Ph
1o
f1 (ppm)
f1 (ppm)

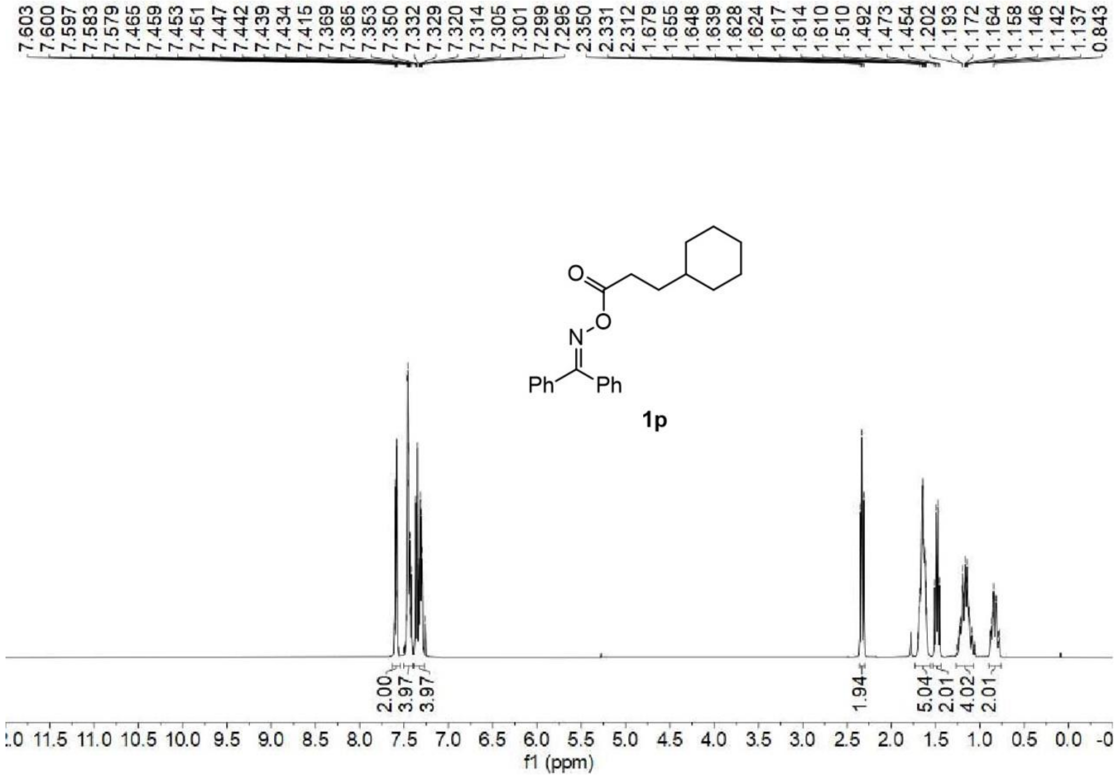
7.603 7.600 7.597 7.583 7.579 7.465 7.459 7.453 7.451 7.447 7.442 7.439 7.434 7.415 7.369 7.365 7.353 7.350 7.332 7.329 7.320 7.314 7.305 7.301 7.299 7.295 2.350 2.331 2.312 1.679 1.655 1.648 1.639 1.628 1.624 1.617 1.614 1.610 1.510 1.492 1.473 1.454 1.202 1.193 1.172 1.164 1.158 1.146 1.142 1.137 0.843
O
N
O
Ph
Ph
1p
2.00
3.97
3.97
1.94
5.04
2.01
4.02
2.01
f1 (ppm)

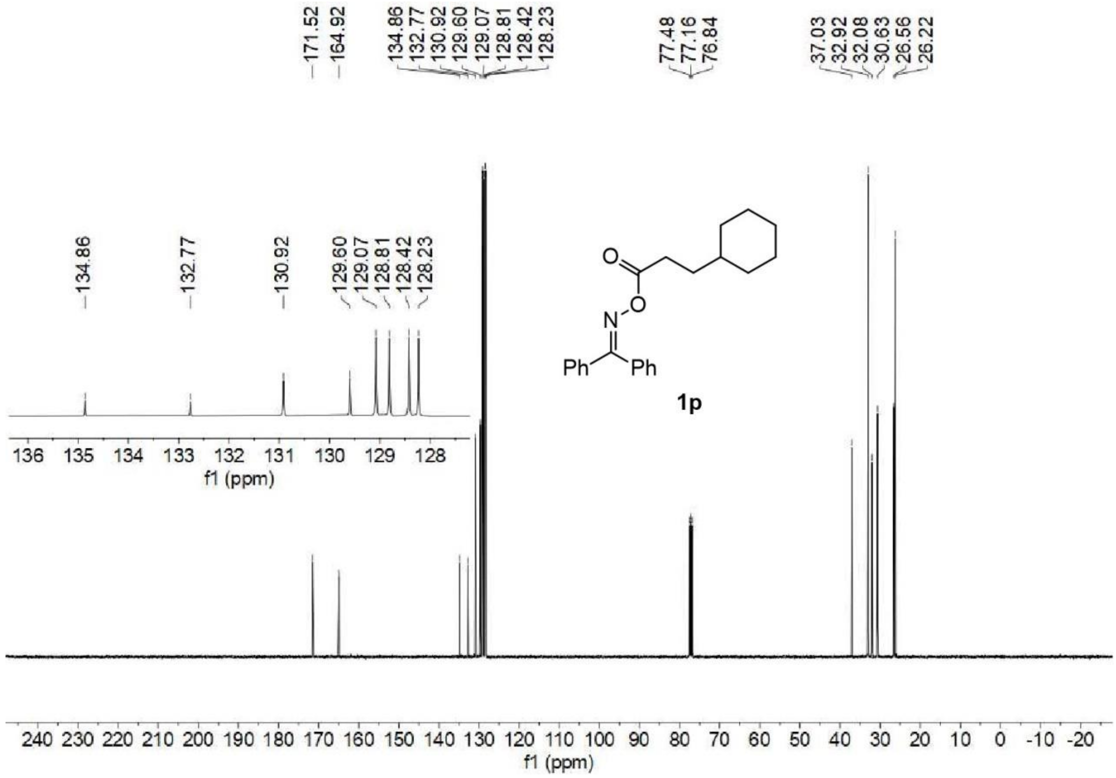
171.52 164.92
134.86 132.77 130.92 129.60 129.07 128.81 128.42 128.23
77.48 77.16 76.84
37.03 32.92 32.08 30.63 26.56 26.22
O
N
O
Ph
Ph
1p
f1 (ppm)

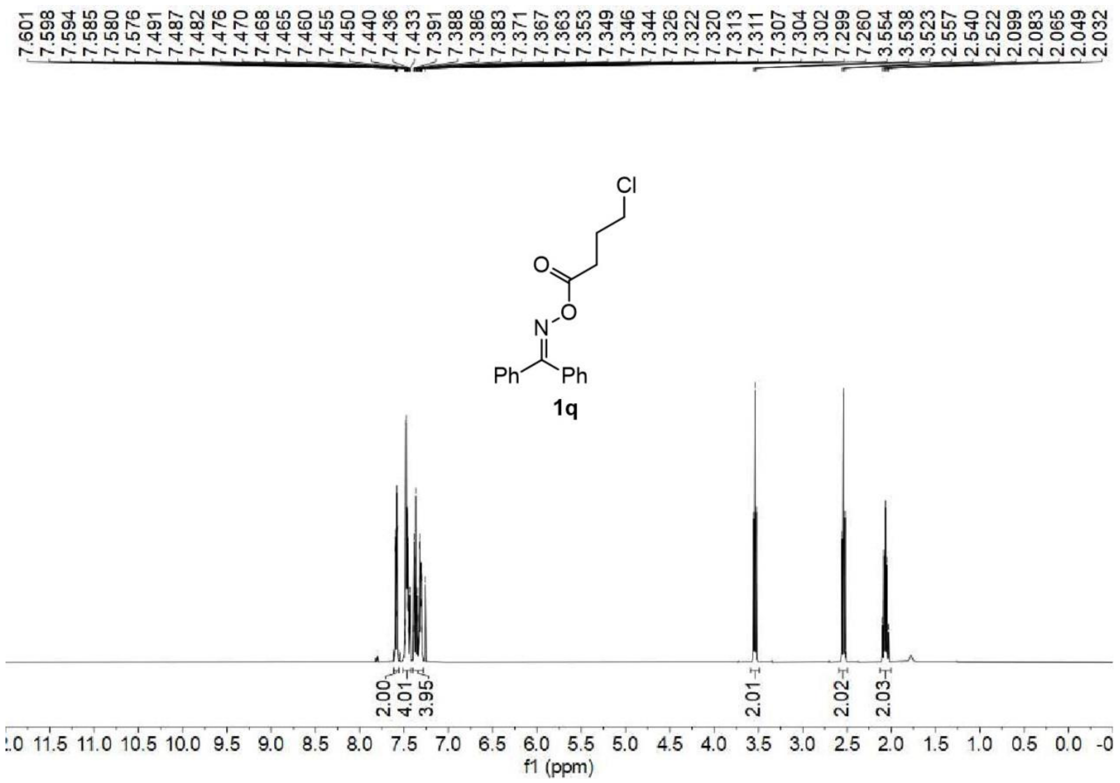
7.601
7.598
7.594
7.585
7.580
7.576
7.491
7.487
7.482
7.476
7.470
7.468
7.465
7.460
7.455
7.450
7.440
7.436
7.433
7.391
7.388
7.386
7.383
7.371
7.367
7.363
7.353
7.349
7.346
7.344
7.326
7.322
7.320
7.313
7.311
7.307
7.304
7.302
7.299
7.260
3.554
3.538
3.523
2.557
2.540
2.522
2.099
2.083
2.065
2.049
2.032
Cl
O
O
N
Ph
Ph
1q
2.00
4.01
3.95
2.01
2.02
2.03
f1 (ppm)

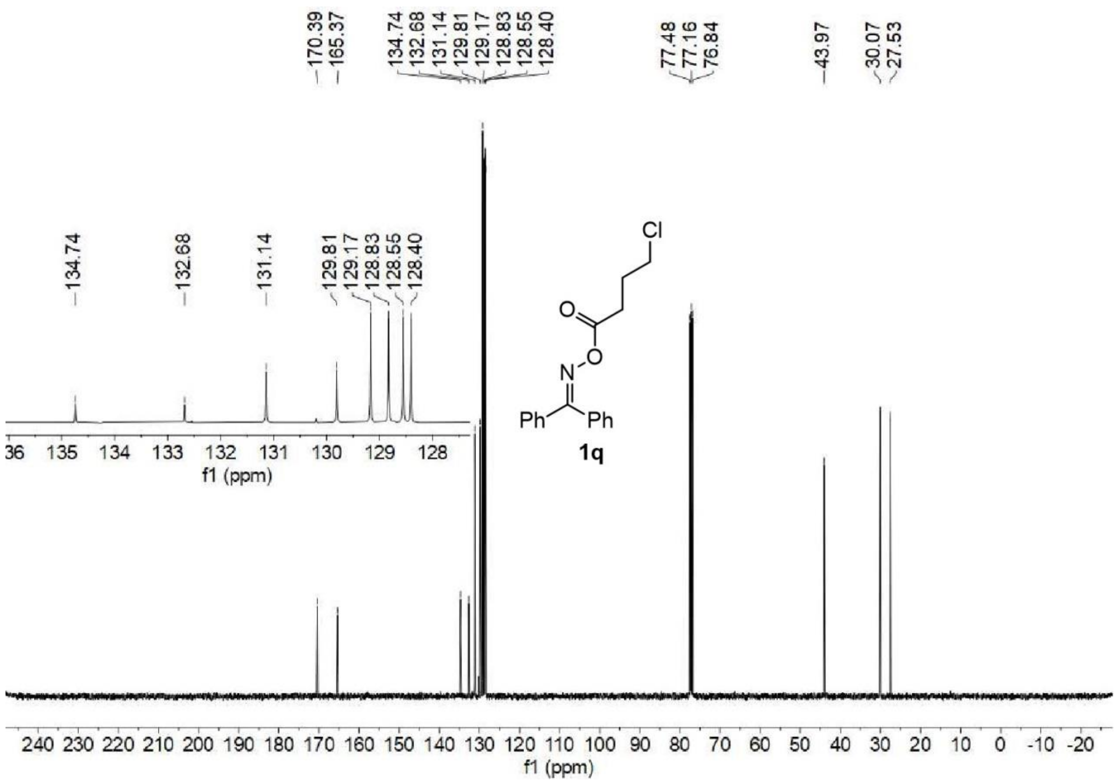
170.39
165.37
134.74
132.68
131.14
129.81
129.17
128.83
128.55
128.40
77.48
77.16
76.84
43.97
30.07
27.53
Cl
O
O
N
Ph
Ph
1q
f1 (ppm)

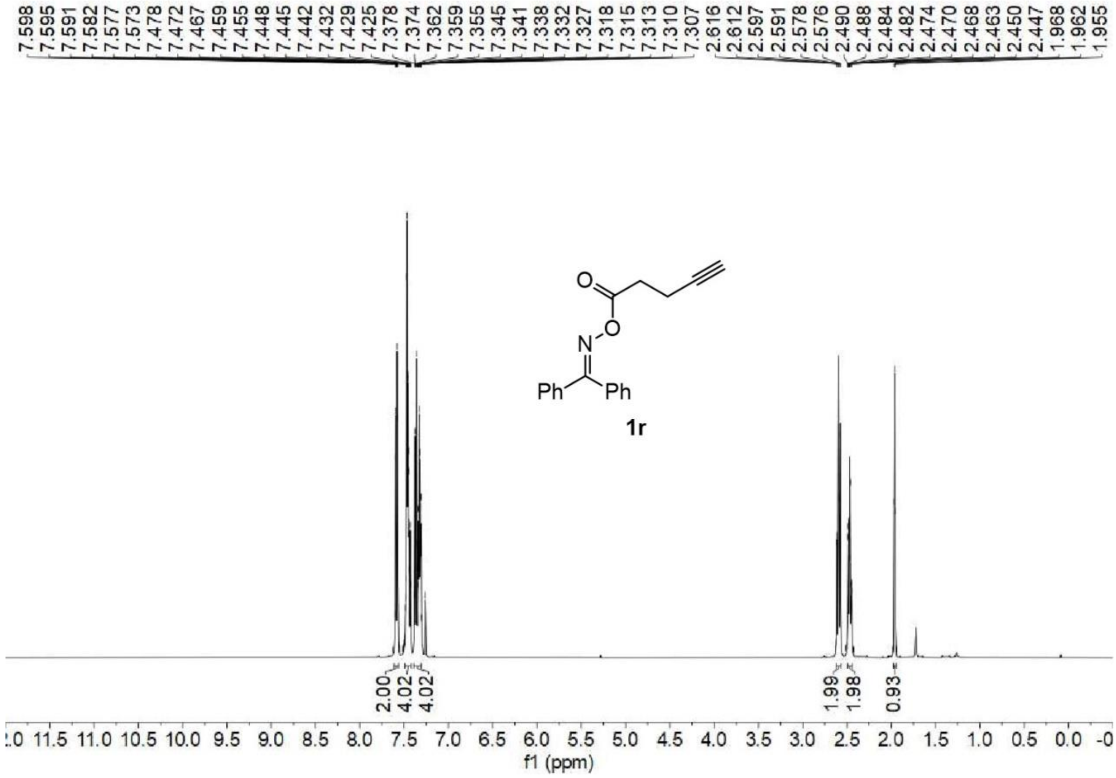
7.598 7.595 7.591 7.582 7.577 7.573 7.478 7.472 7.467 7.459 7.455 7.448 7.445 7.442 7.432 7.429 7.425 7.378 7.374 7.362 7.359 7.355 7.345 7.341 7.338 7.332 7.327 7.318 7.315 7.313 7.310 7.307 2.616 2.612 2.597 2.591 2.578 2.576 2.490 2.488 2.484 2.482 2.474 2.470 2.468 2.463 2.450 2.447 1.968 1.962 1.955
O
N
O
Ph
Ph
1r
2.00
4.02
4.02
1.99
1.98
0.93
f1 (ppm)

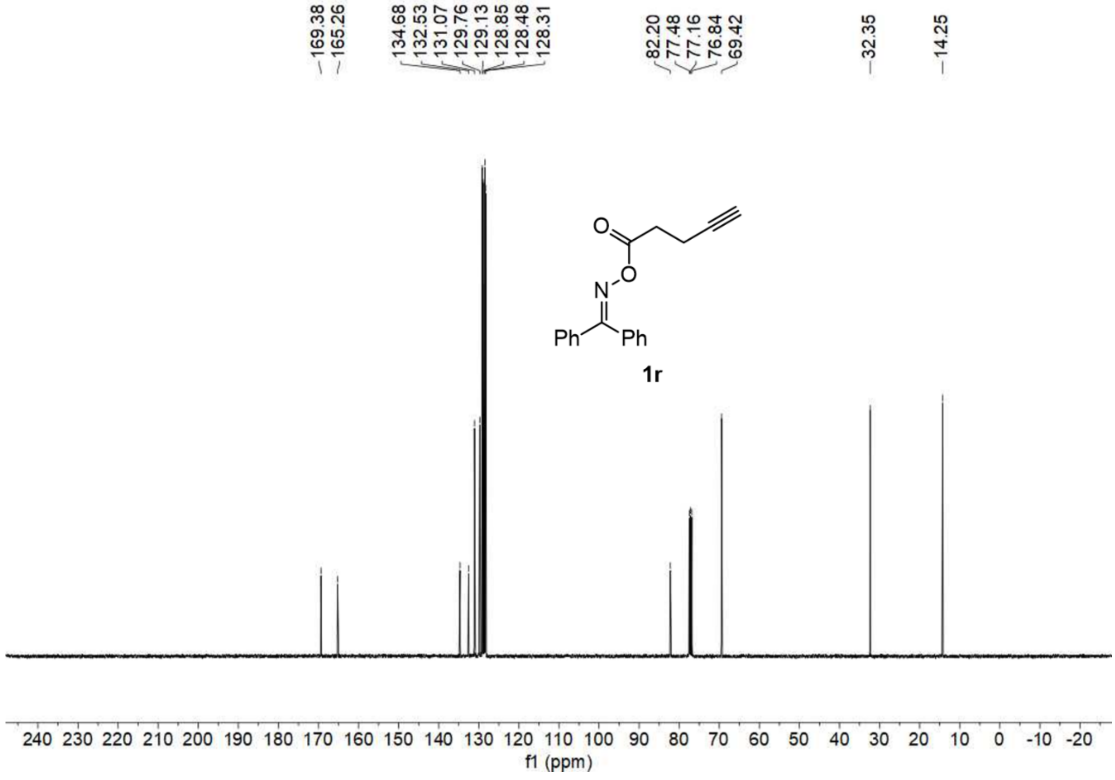
169.38
165.26
134.68 132.53 131.07 129.76 129.13 128.85 128.48 128.31
82.20 77.48 77.16 76.84 69.42
32.35
14.25
O
N
O
Ph
Ph
1r
f1 (ppm)

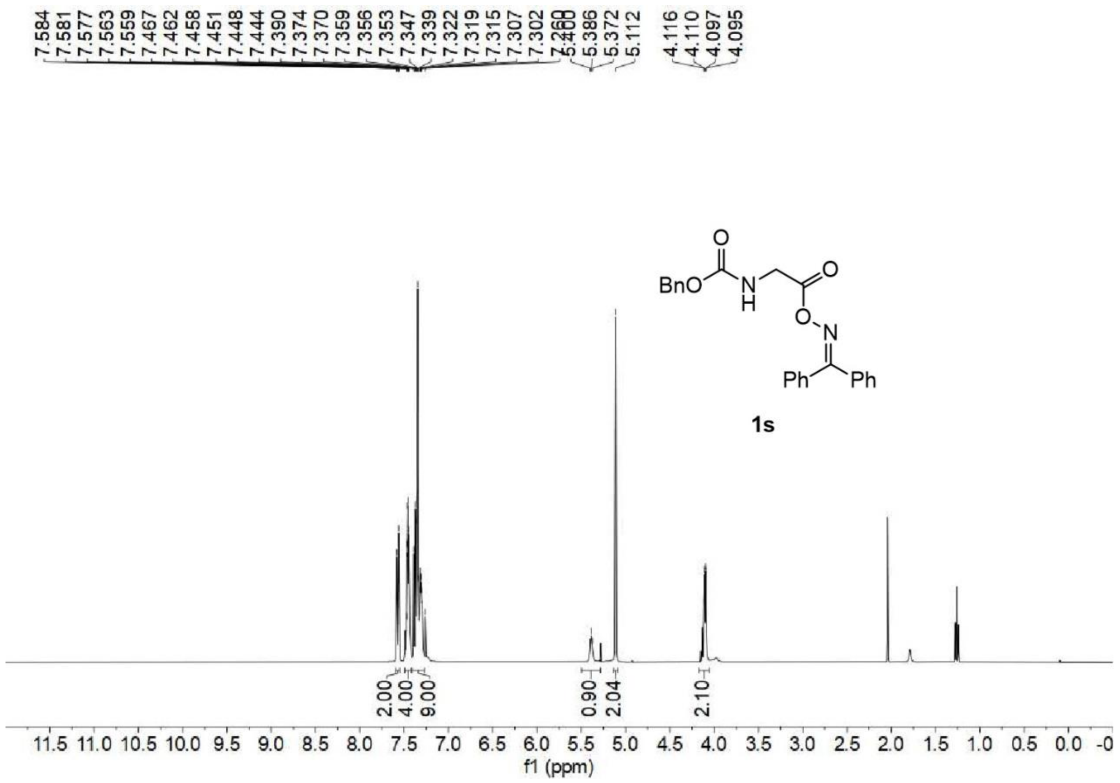
7.584
7.581
7.577
7.563
7.559
7.467
7.462
7.458
7.451
7.448
7.444
7.390
7.374
7.370
7.359
7.356
7.353
7.347
7.339
7.322
7.319
7.315
7.307
7.302
5.386
5.372
5.112
4.116
4.110
4.097
4.095
BnO
N
H
O
O
O
N
Ph
Ph
1s
2.00
4.00
9.00
0.90
2.04
2.10
11.5 11.0 10.5 10.0 9.5 9.0 8.5 8.0 7.5 7.0 6.5 6.0 5.5 5.0 4.5 4.0 3.5 3.0 2.5 2.0 1.5 1.0 0.5 0.0
f1 (ppm)

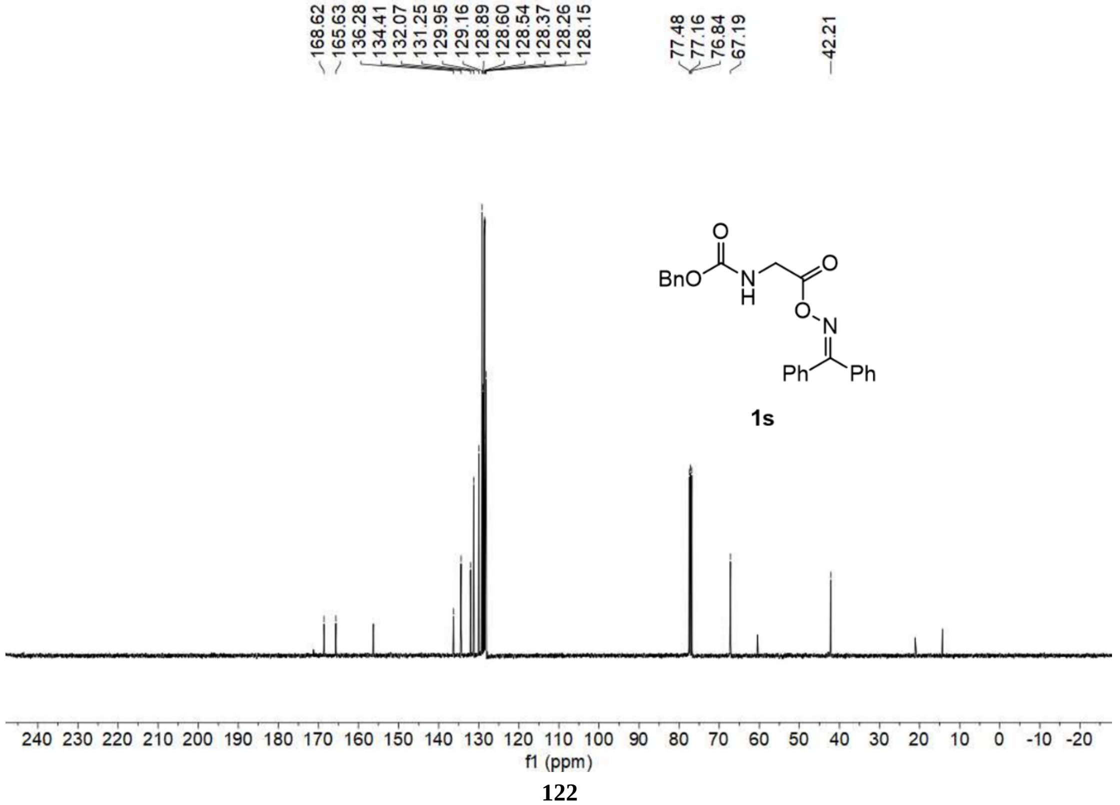
168.62
165.63
136.28
134.41
132.07
131.25
129.95
129.16
128.89
128.60
128.54
128.37
128.26
128.15
77.48
77.16
76.84
67.19
42.21
BnO
N
H
O
O
O
N
Ph
Ph
1s
240 230 220 210 200 190 180 170 160 150 140 130 120 110 100 90 80 70 60 50 40 30 20 10 0 -10 -20
f1 (ppm)

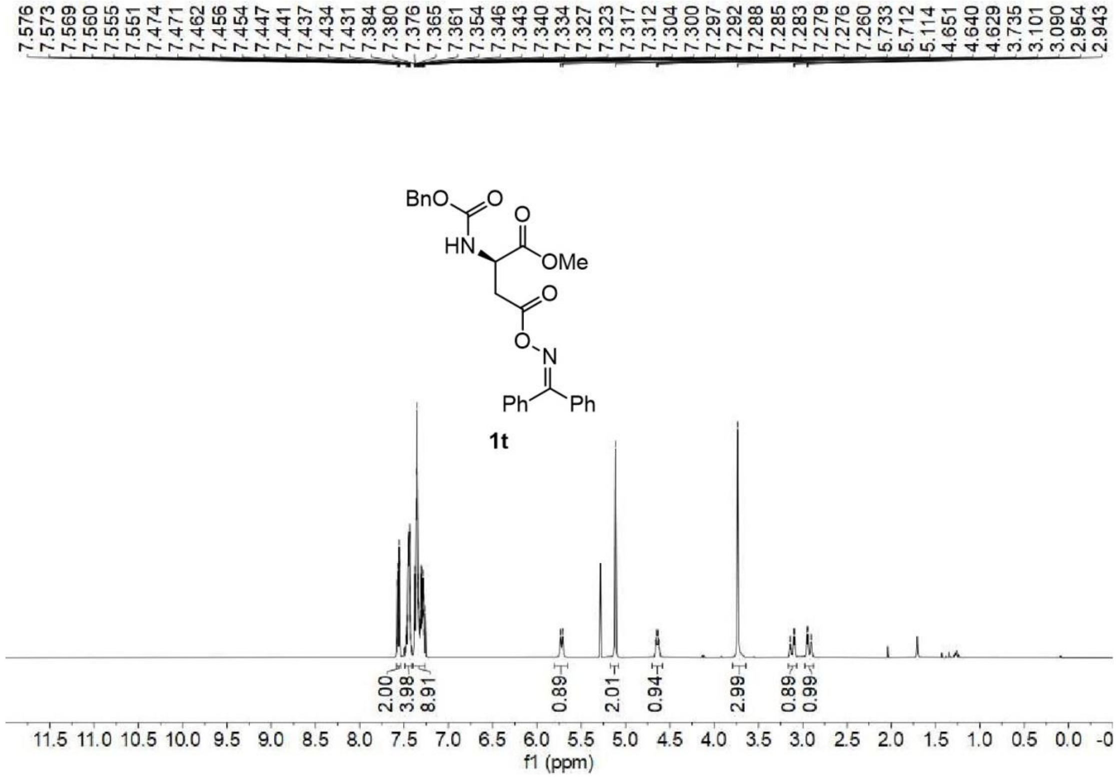
7.576 7.573 7.569 7.560 7.555 7.551 7.474 7.471 7.462 7.456 7.454 7.447 7.441 7.437 7.434 7.431 7.384 7.380 7.376 7.365 7.361 7.354 7.346 7.343 7.340 7.334 7.327 7.323 7.317 7.312 7.304 7.300 7.297 7.292 7.288 7.285 7.283 7.279 7.276 7.260 5.733 5.712 5.114 4.651 4.640 4.629 3.735 3.101 3.090 2.954 2.943
BnO O HN O OMe O O N Ph Ph
1t
2.00 3.98 8.91 0.89 2.01 0.94 2.99 0.89 0.99
11.5 11.0 10.5 10.0 9.5 9.0 8.5 8.0 7.5 7.0 6.5 6.0 5.5 5.0 4.5 4.0 3.5 3.0 2.5 2.0 1.5 1.0 0.5 0.0 -0
f1 (ppm)

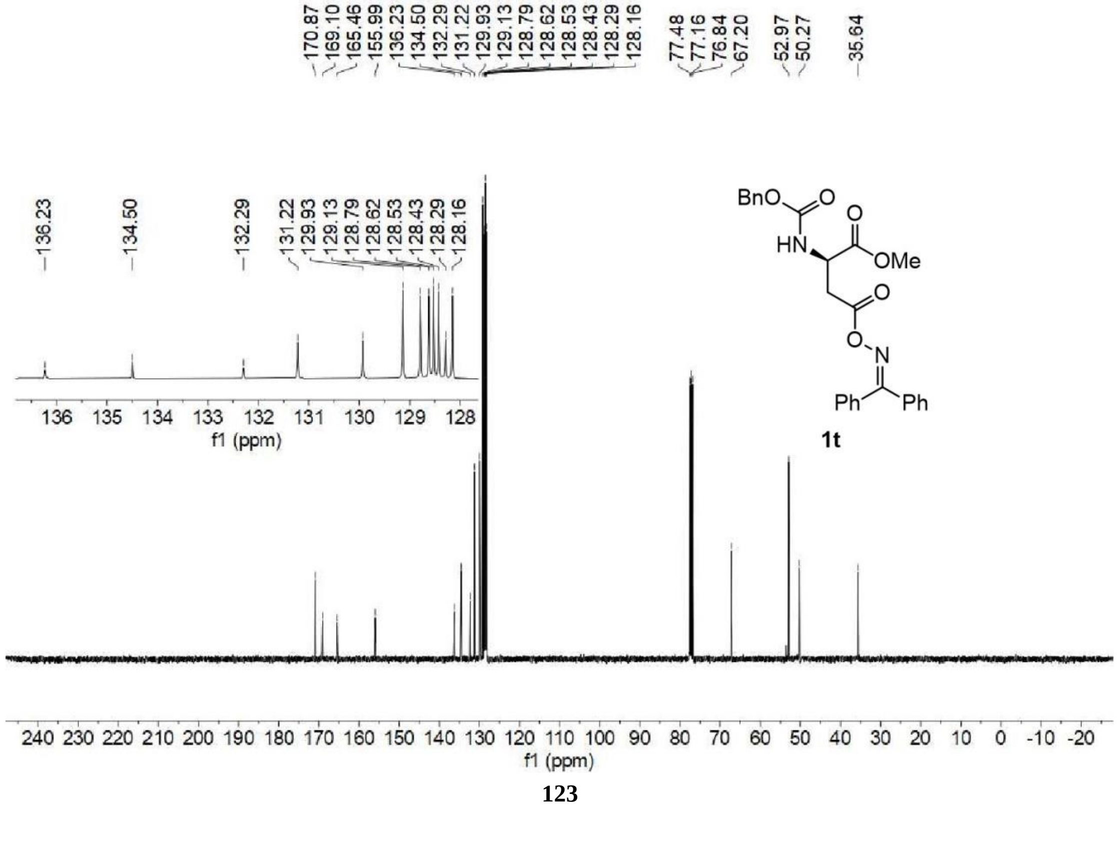
170.87 169.10 165.46 155.99 136.23 134.50 132.29 131.22 129.93 129.13 128.79 128.62 128.53 128.43 128.29 128.16 77.48 77.16 76.84 67.20 52.97 50.27 35.64
136.23 134.50 132.29 131.22 129.93 129.13 128.79 128.62 128.53 128.43 128.29 128.16
136 135 134 133 132 131 130 129 128
f1 (ppm)
BnO O HN O OMe O O N Ph Ph
1t
240 230 220 210 200 190 180 170 160 150 140 130 120 110 100 90 80 70 60 50 40 30 20 10 0 -10 -20
f1 (ppm)

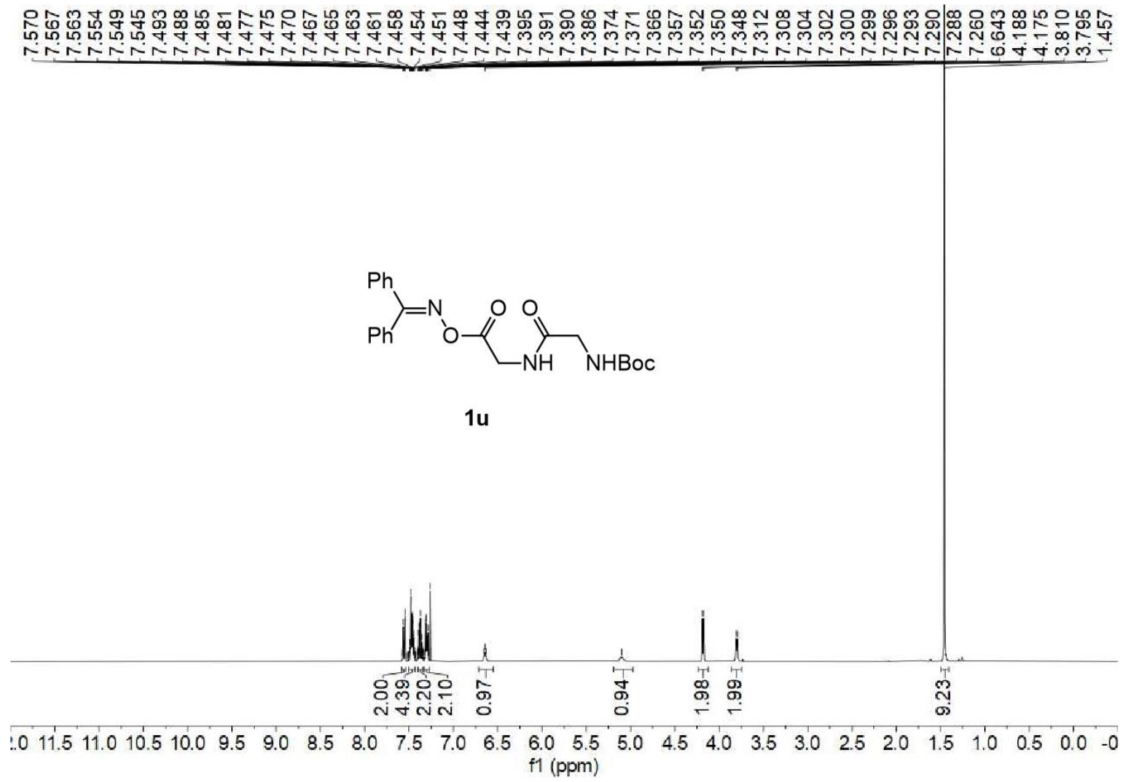
7.570 7.567 7.563 7.554 7.549 7.545 7.493 7.488 7.485 7.481 7.477 7.475 7.470 7.467 7.465 7.463 7.461 7.458 7.454 7.451 7.448 7.444 7.439 7.395 7.391 7.390 7.386 7.374 7.371 7.366 7.357 7.352 7.350 7.348 7.312 7.308 7.304 7.302 7.300 7.299 7.296 7.293 7.290 7.288 7.260 6.643 4.188 4.175 3.810 3.795 1.457
Ph
N
O
Ph
O
O
NH
NHBoc
1u
2.00
4.39
2.20
2.10
0.97
0.94
1.98
1.99
9.23
f1 (ppm)

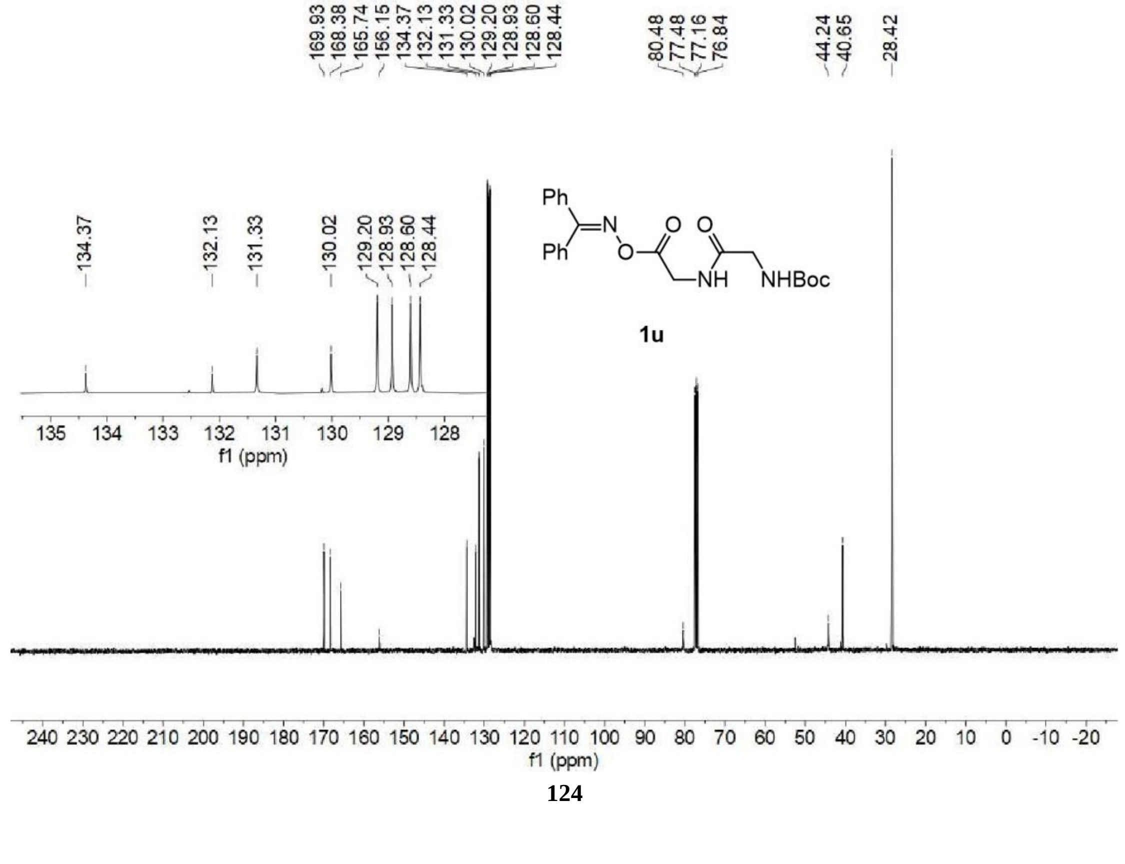
169.93 168.38 165.74 156.15 134.37 132.13 131.33 130.02 129.20 128.93 128.60 128.44
80.48 77.48 77.16 76.84
44.24 40.65
28.42
134.37
132.13
131.33
130.02
129.20
128.93
128.60
128.44
f1 (ppm)
Ph
N
O
Ph
O
O
NH
NHBoc
1u
f1 (ppm)

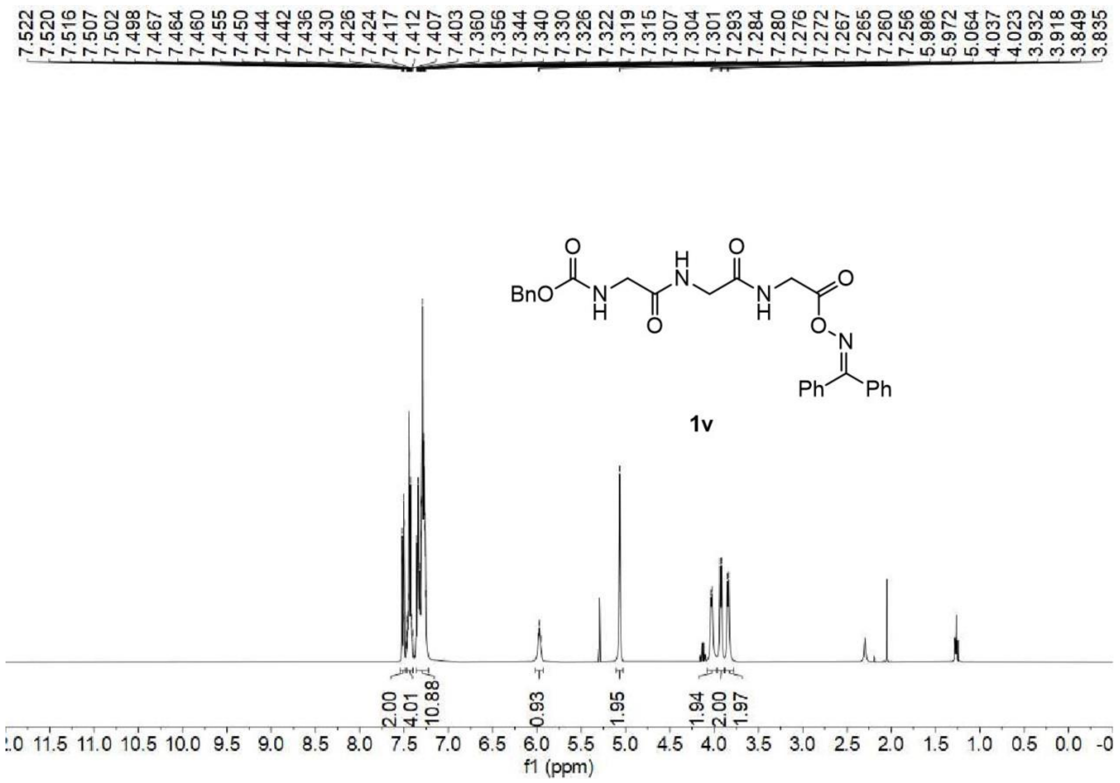

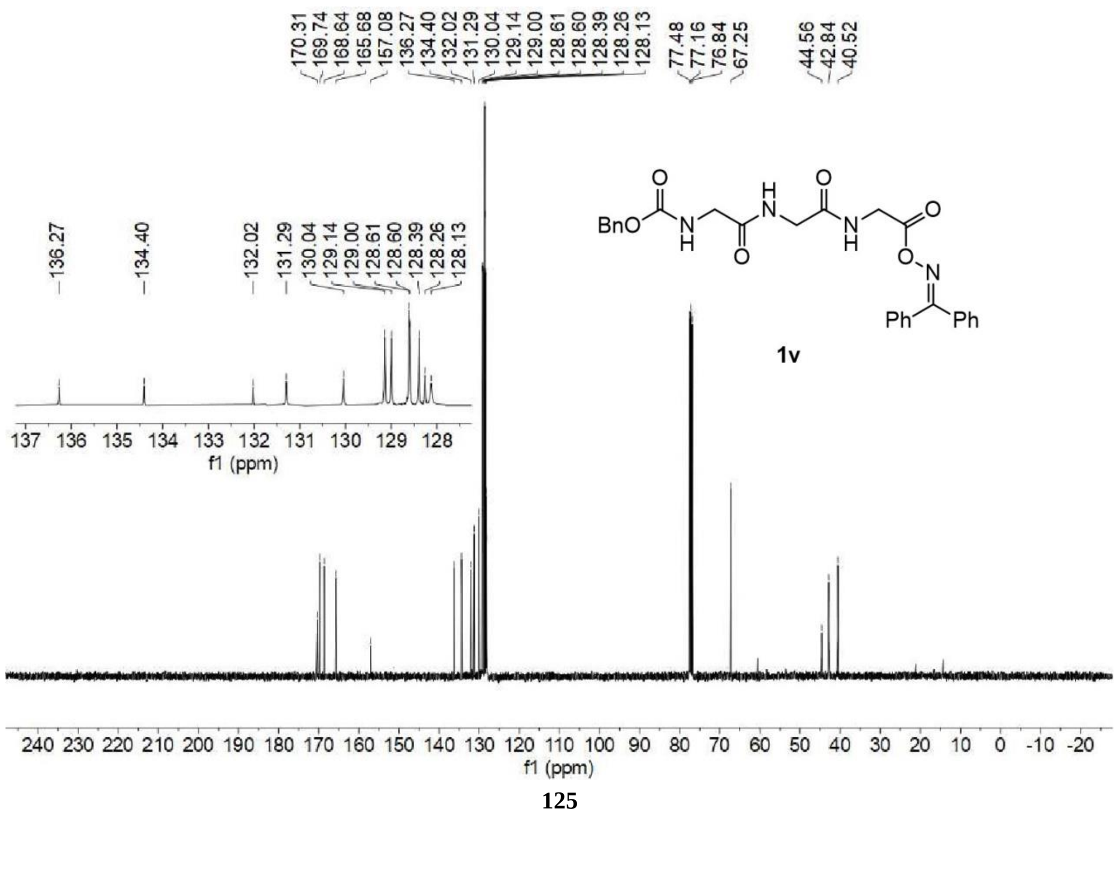

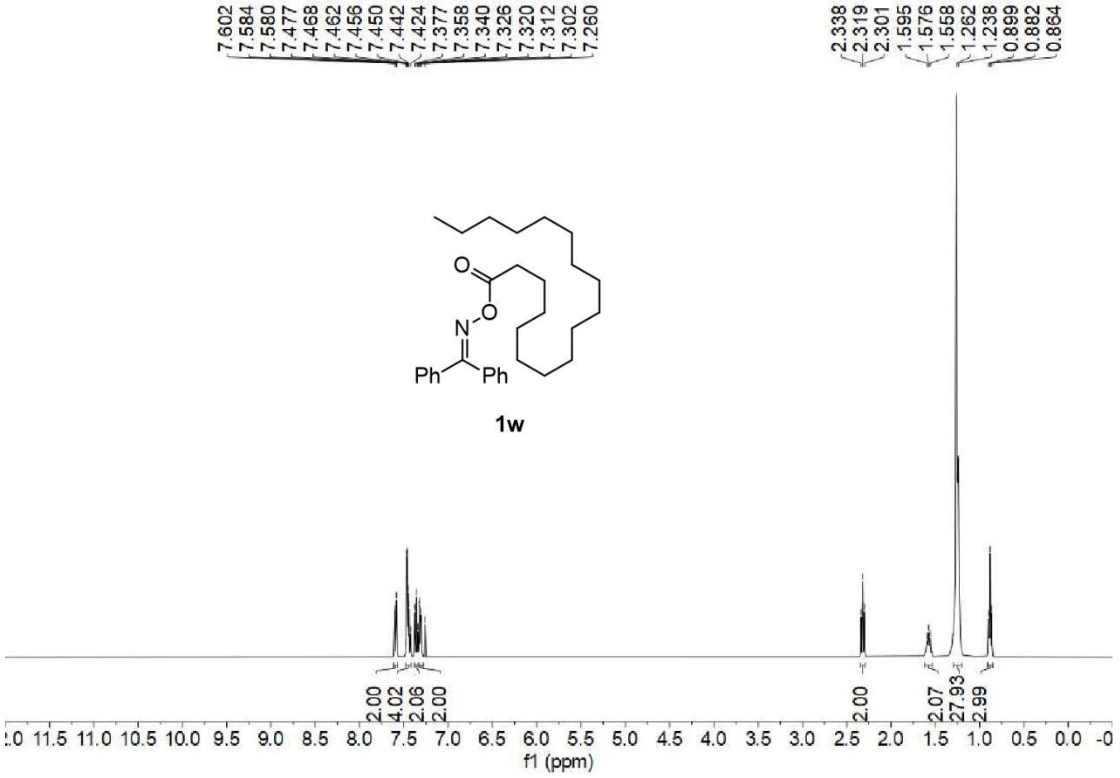
7.602 7.584 7.580 7.477 7.468 7.462 7.456 7.450 7.442 7.424 7.377 7.358 7.340 7.326 7.320 7.312 7.302 7.260
2.338 2.319 2.301 1.595 1.576 1.558 1.262 1.238 0.899 0.882 0.864
O
O
N
Ph
Ph
1w
2.00 4.02 2.06 2.00
2.00
2.07 27.93 2.99
f1 (ppm)

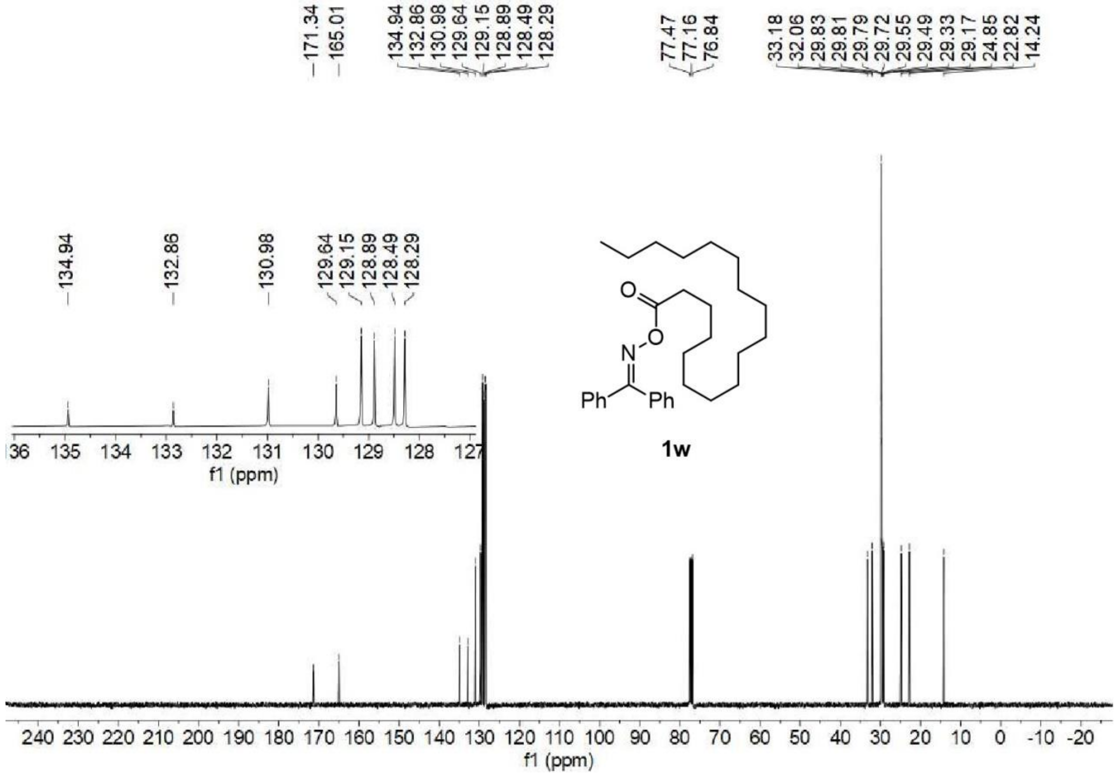
171.34 165.01
134.94 132.86 130.98 129.64 129.15 128.89 128.49 128.29
77.47 77.16 76.84
33.18 32.06 29.83 29.81 29.79 29.72 29.55 29.49 29.33 29.17 24.85 22.82 14.24
O
O
N
Ph
Ph
1w
f1 (ppm)

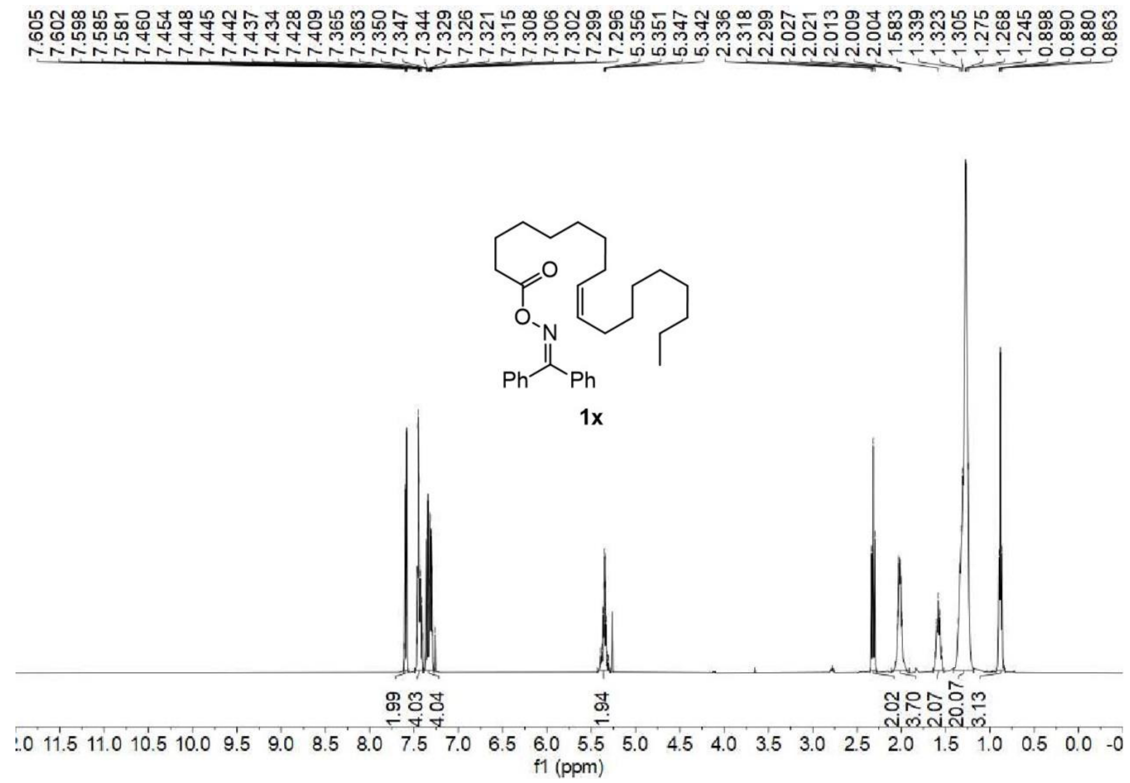
7.605
7.602
7.598
7.585
7.581
7.460
7.454
7.448
7.445
7.442
7.437
7.434
7.428
7.409
7.365
7.363
7.350
7.347
7.344
7.329
7.326
7.321
7.315
7.308
7.306
7.302
7.299
7.296
5.356
5.351
5.347
5.342
2.336
2.318
2.299
2.027
2.021
2.013
2.009
2.004
1.583
1.339
1.323
1.305
1.275
1.268
1.245
0.898
0.890
0.880
0.863
O
O
N
Ph
Ph
1x
1.99
4.03
4.04
1.94
2.02
3.70
2.07
20.07
3.13
f1 (ppm)

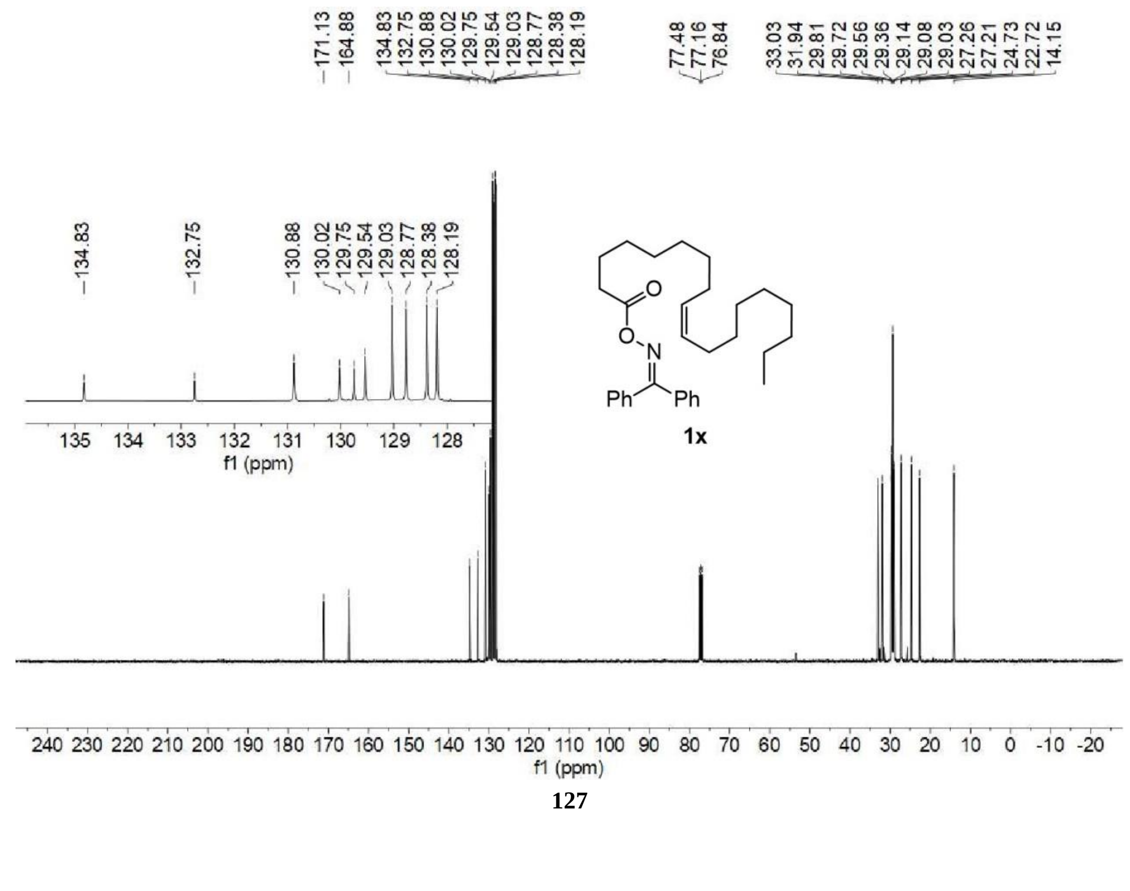
171.13
164.88
134.83
132.75
130.88
130.02
129.75
129.54
129.03
128.77
128.38
128.19
77.48
77.16
76.84
33.03
31.94
29.81
29.72
29.56
29.36
29.14
29.08
29.03
27.26
27.21
24.73
22.72
14.15
O
O
N
Ph
Ph
1x
f1 (ppm)
f1 (ppm)

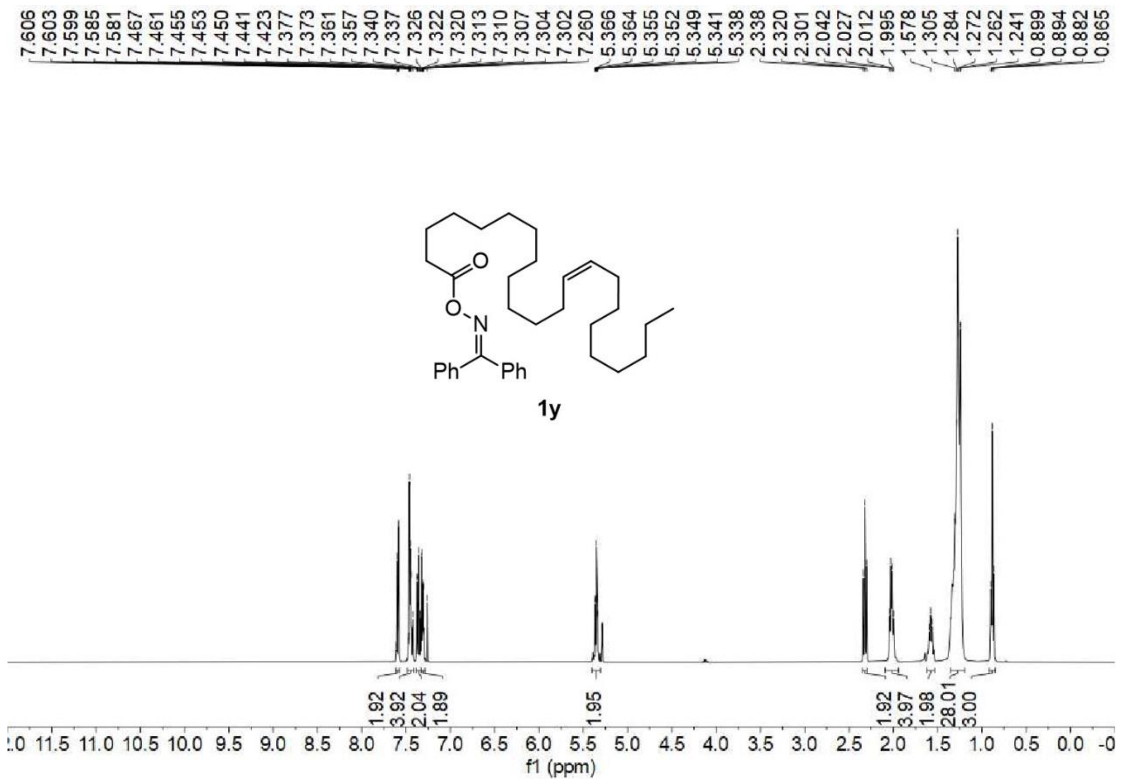
7.606 7.603 7.599 7.585 7.581 7.467 7.461 7.455 7.453 7.450 7.441 7.423 7.377 7.373 7.361 7.357 7.340 7.337 7.326 7.322 7.320 7.313 7.310 7.307 7.304 7.302 7.260 5.366 5.364 5.355 5.352 5.349 5.341 5.338 2.338 2.320 2.301 2.042 2.027 2.012 1.995 1.578 1.305 1.284 1.272 1.262 1.241 0.899 0.894 0.882 0.865
O
O
N
Ph
Ph
1y
1.92 3.92 2.04 1.89
1.95
1.92 3.97 1.98 28.01 3.00
f1 (ppm)

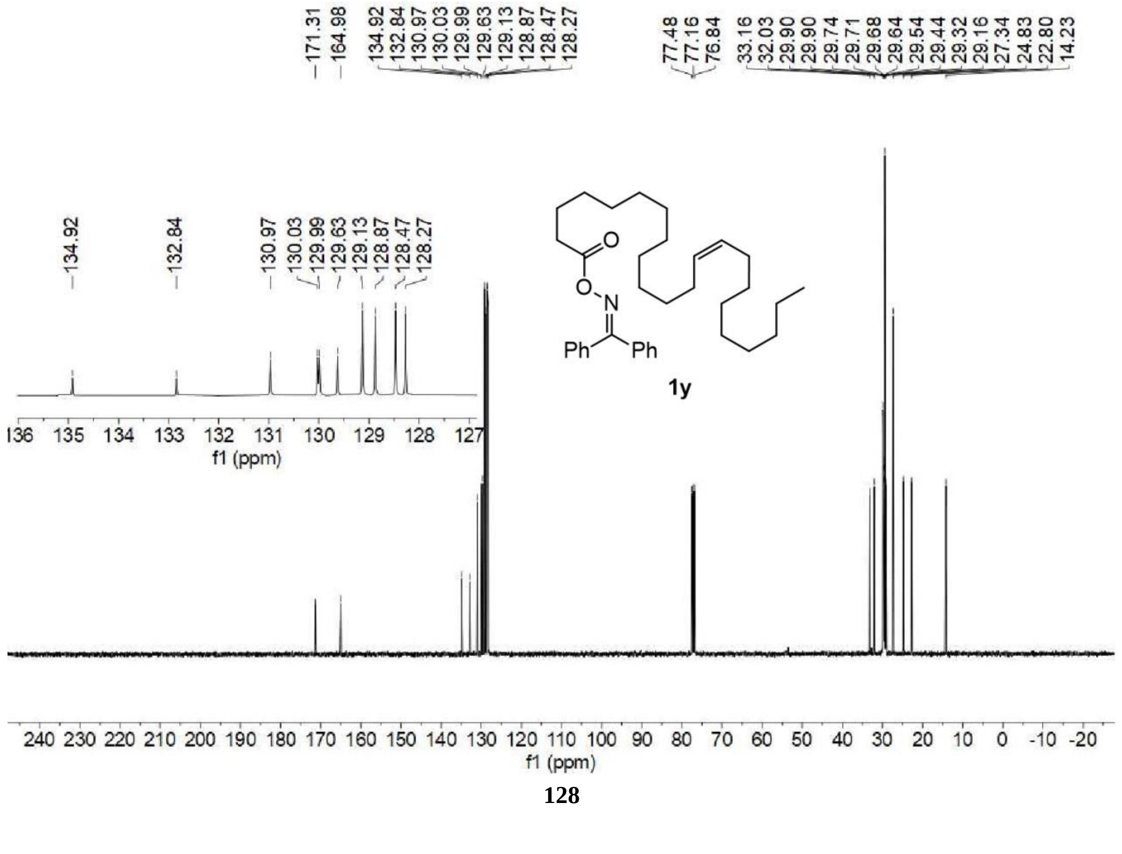
171.31 164.98 134.92 132.84 130.97 130.03 129.99 129.63 129.13 128.87 128.47 128.27
77.48 77.16 76.84 33.16 32.03 29.90 29.90 29.74 29.71 29.68 29.64 29.54 29.44 29.32 29.16 27.34 24.83 22.80 14.23
134.92 132.84 130.97 130.03 129.99 129.63 129.13 128.87 128.47 128.27
f1 (ppm)
O
O
N
Ph
Ph
1y
f1 (ppm)

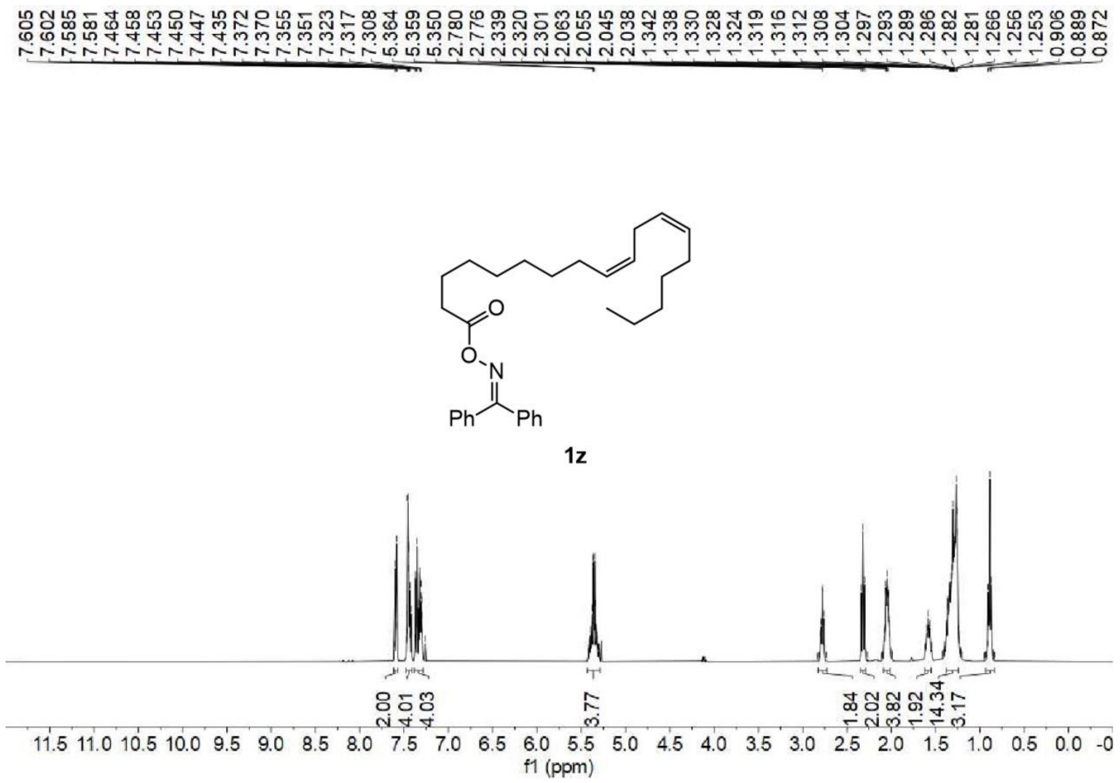

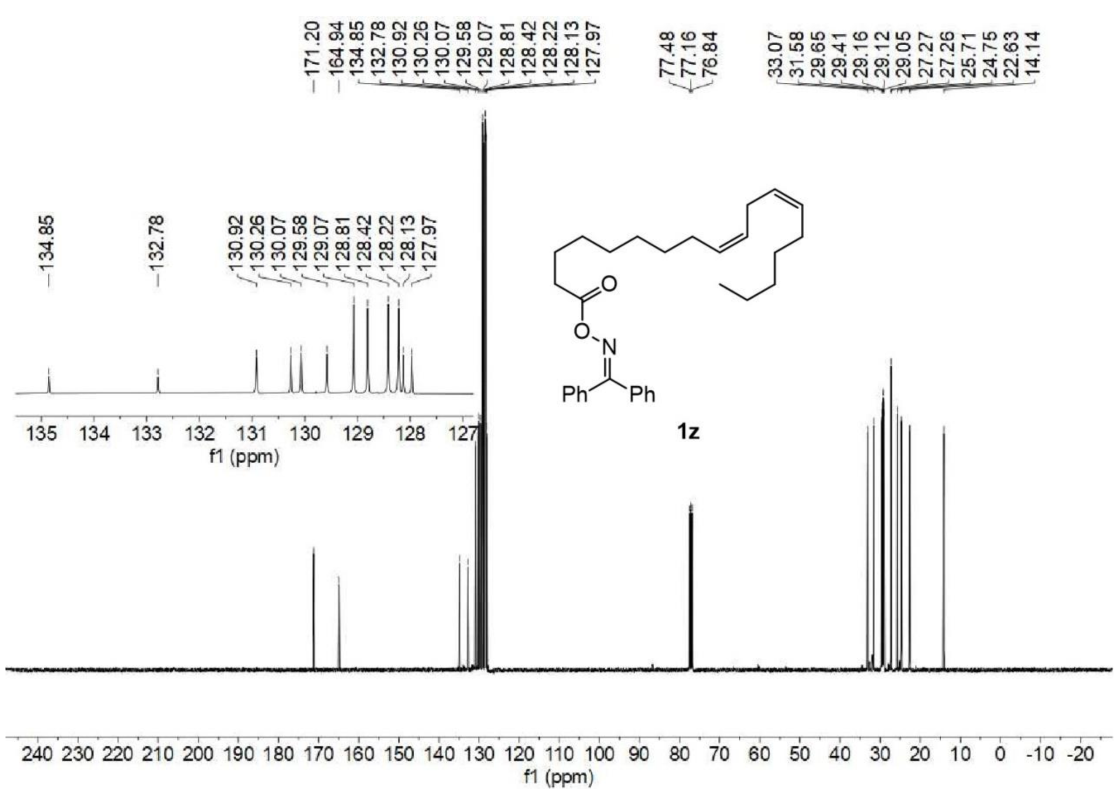

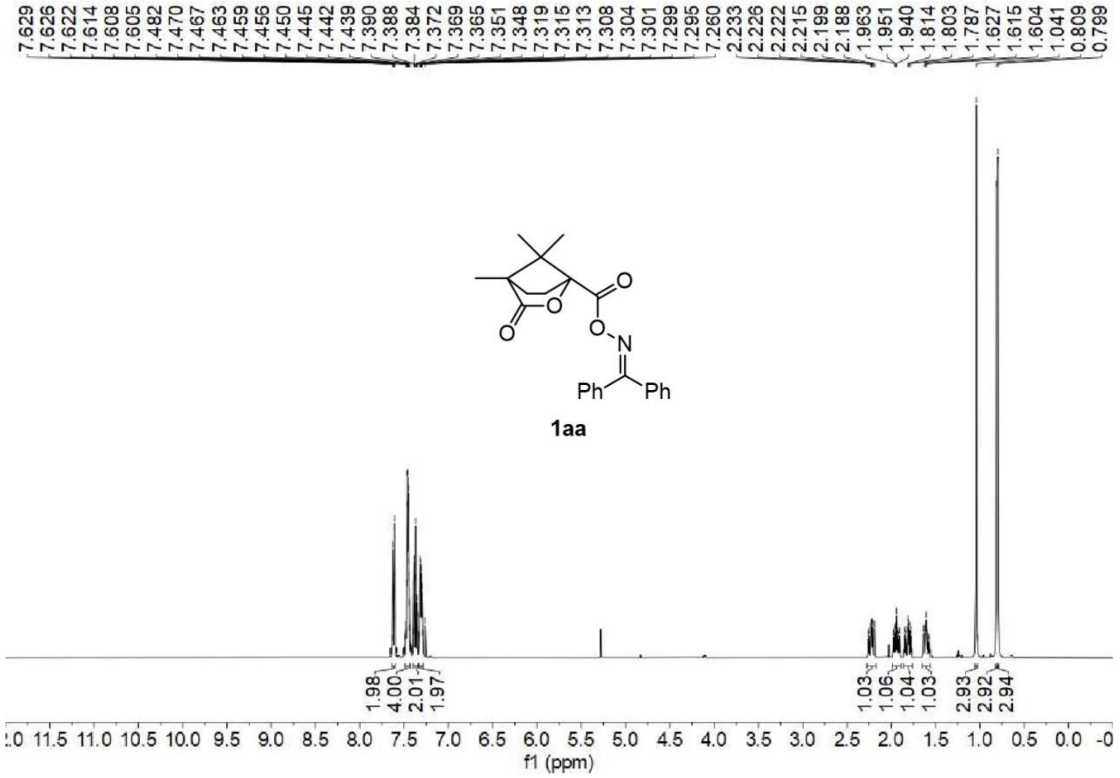
7.629 7.626 7.622 7.614 7.608 7.605 7.482 7.470 7.467 7.463 7.459 7.456 7.450 7.445 7.442 7.439 7.390 7.388 7.384 7.372 7.369 7.365 7.351 7.348 7.319 7.315 7.313 7.308 7.304 7.301 7.299 7.295 7.260 2.233 2.226 2.222 2.215 2.199 2.188 1.963 1.951 1.940 1.814 1.803 1.787 1.627 1.615 1.604 1.041 0.809 0.799
Ph
Ph
1aa
1.98
4.00
2.01
1.97
1.03
1.06
1.04
1.03
2.93
2.92
2.94
f1 (ppm)

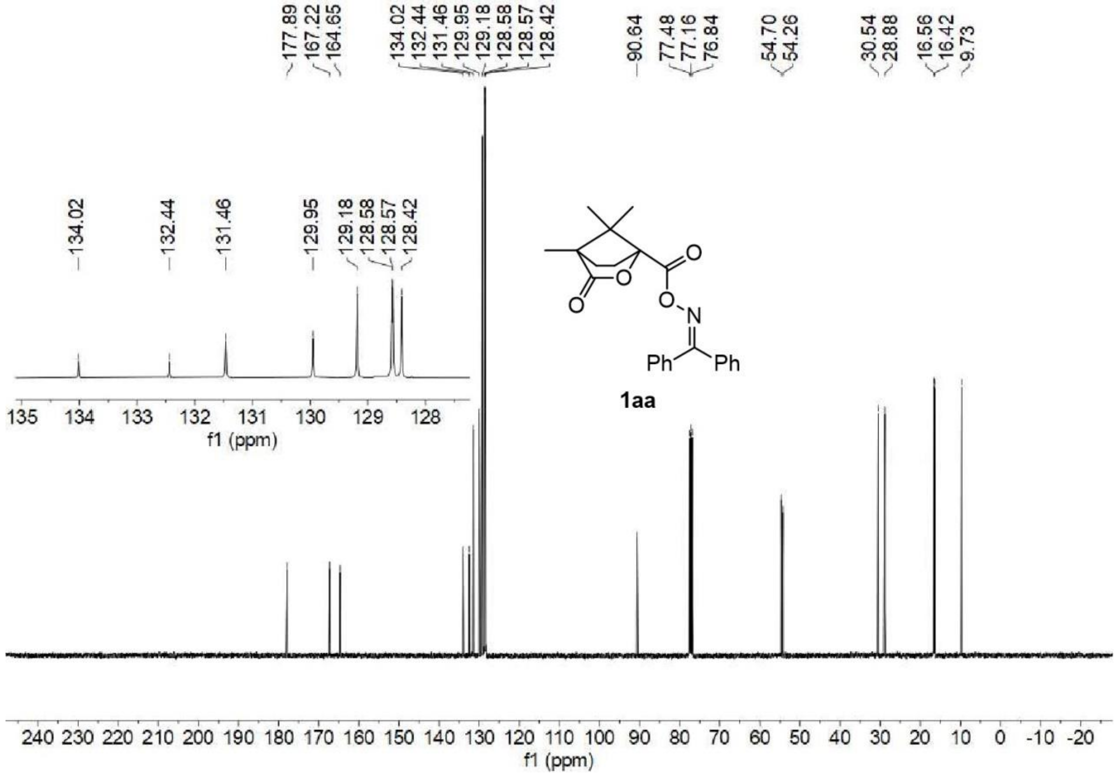
177.89 167.22 164.65 134.02 132.44 131.46 129.95 129.18 128.58 128.57 128.42 90.64 77.48 77.16 76.84 54.70 54.26 30.54 28.88 16.56 16.42 9.73
134.02 132.44 131.46 129.95 129.18 128.58 128.57 128.42
f1 (ppm)
Ph
Ph
1aa
f1 (ppm)

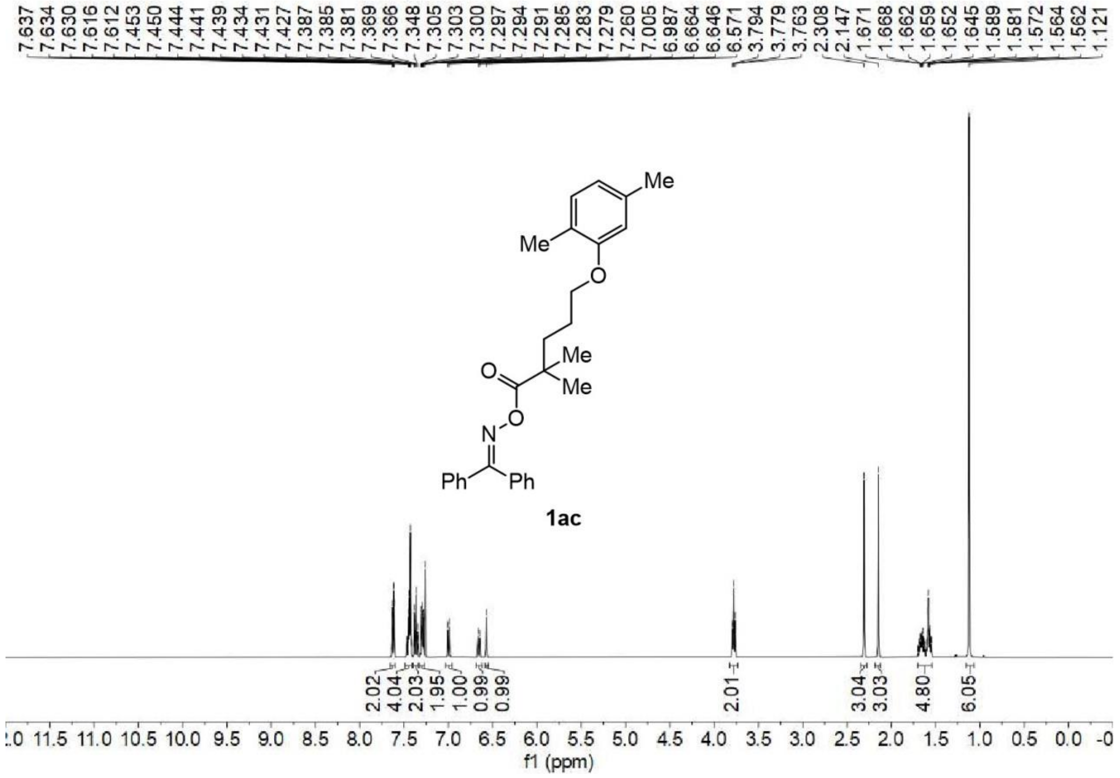

7.637 7.634 7.630 7.616 7.612 7.453 7.450 7.444 7.441 7.439 7.434 7.431 7.427 7.387 7.385 7.381 7.369 7.366 7.348 7.305 7.303 7.300 7.297 7.294 7.291 7.285 7.283 7.279 7.260 7.005 6.987 6.664 6.646 6.571 3.794 3.779 3.763 2.308 2.147 1.671 1.668 1.662 1.659 1.652 1.645 1.589 1.581 1.572 1.564 1.562 1.121
Me
Me
O
Me
Me
O
O
N
Ph
Ph
1ac
2.02 4.04 2.03 1.95 1.00 0.99 0.99 2.01 3.04 3.03 4.80 6.05
f1 (ppm)

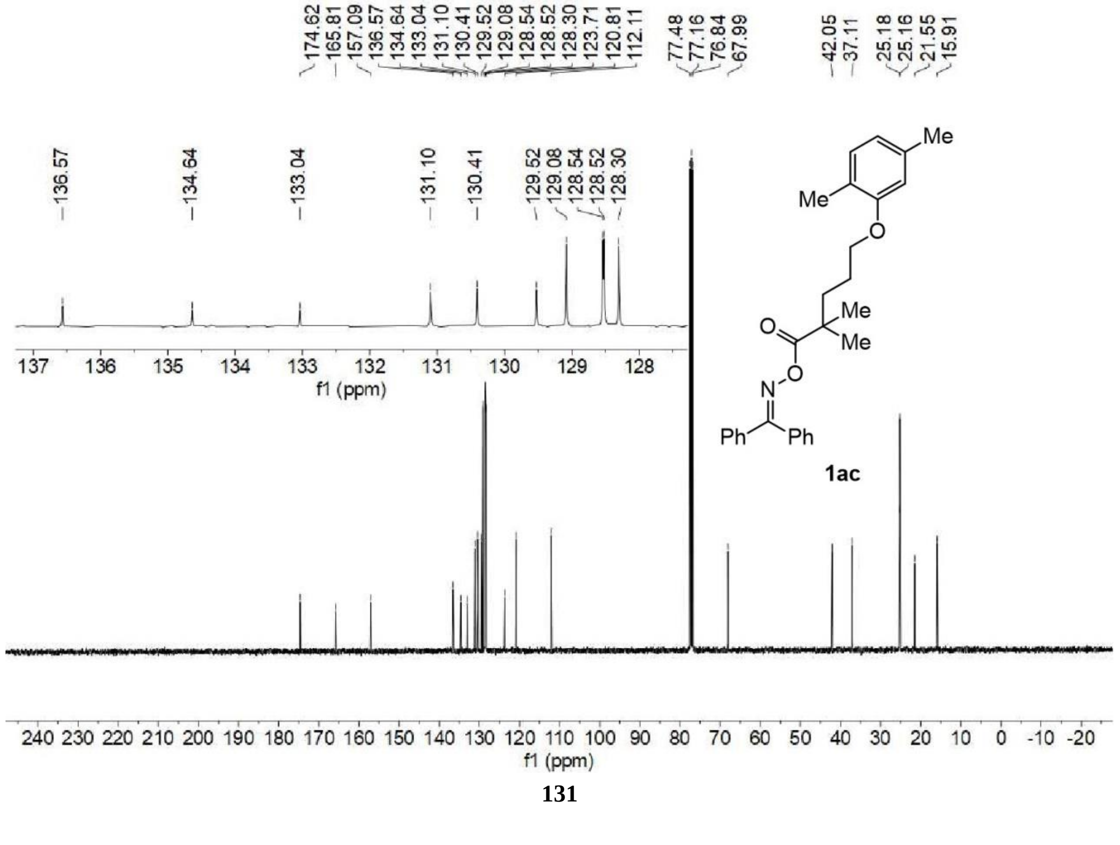

174.62 165.81 157.09 136.57 134.64 133.04 131.10 130.41 129.52 129.08 128.54 128.52 128.30 123.71 120.81 112.11 77.48 77.16 76.84 67.99 42.05 37.11 25.18 25.16 21.55 15.91
136.57 134.64 133.04 131.10 130.41 129.52 129.08 128.54 128.52 128.30
f1 (ppm)
Me
Me
O
Me
Me
O
O
N
Ph
Ph
1ac
f1 (ppm)

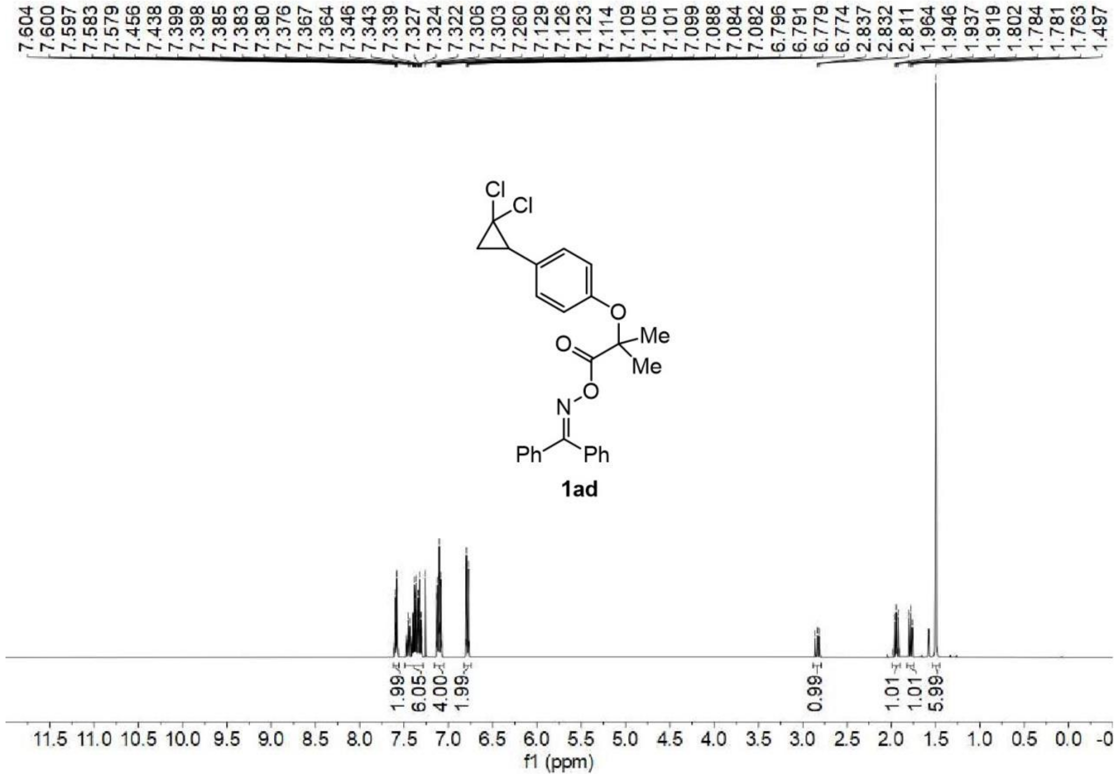
7.604
7.600
7.597
7.583
7.579
7.456
7.438
7.399
7.398
7.385
7.383
7.380
7.376
7.367
7.364
7.346
7.343
7.339
7.327
7.324
7.322
7.306
7.303
7.260
7.129
7.126
7.123
7.114
7.109
7.105
7.101
7.099
7.088
7.084
7.082
6.796
6.791
6.779
6.774
2.837
2.832
2.811
1.964
1.946
1.937
1.919
1.802
1.784
1.781
1.763
1.497
Cl
Cl
O
O
Me
Me
O
N
Ph
Ph
1ad
1.99
6.05
4.00
1.99
0.99
1.01
1.01
5.99
f1 (ppm)

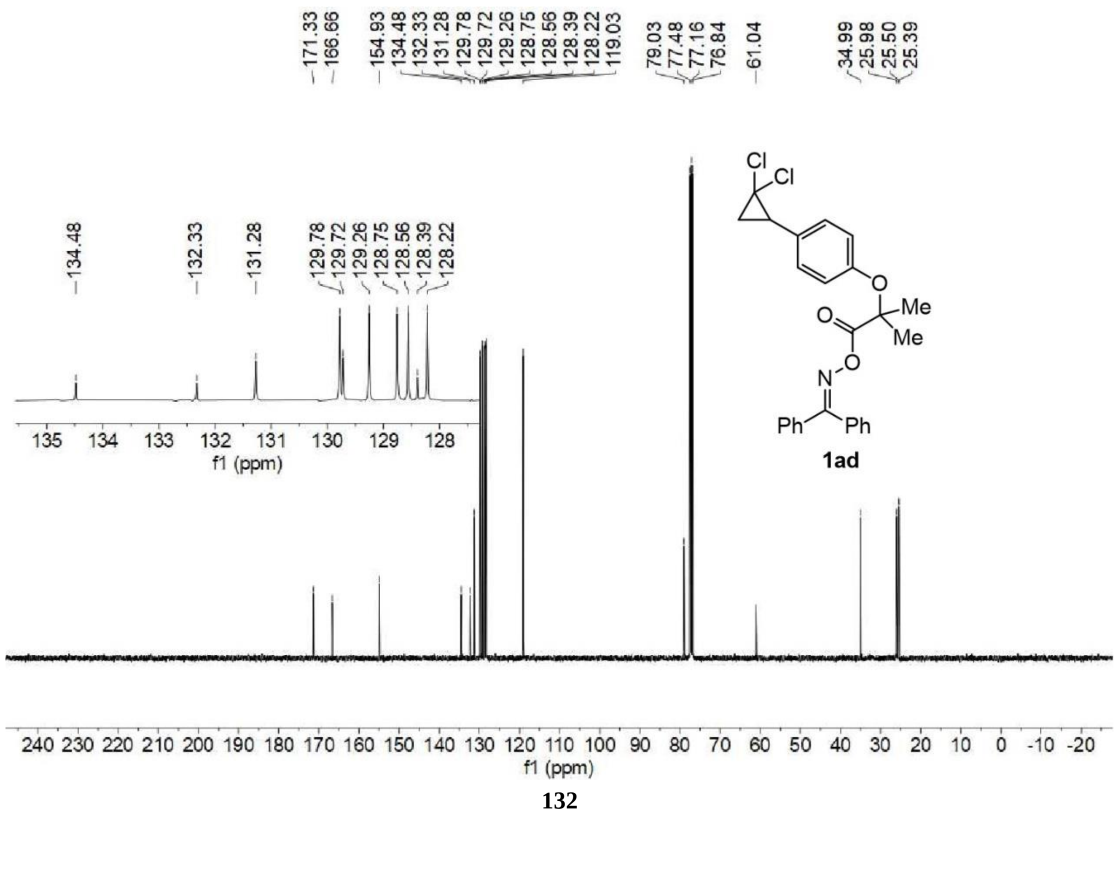
171.33
166.66
154.93
134.48
132.33
131.28
129.78
129.72
129.26
128.75
128.56
128.39
128.22
119.03
79.03
77.48
77.16
76.84
61.04
34.99
25.98
25.50
25.39
Cl
Cl
O
O
Me
Me
O
N
Ph
Ph
1ad
f1 (ppm)

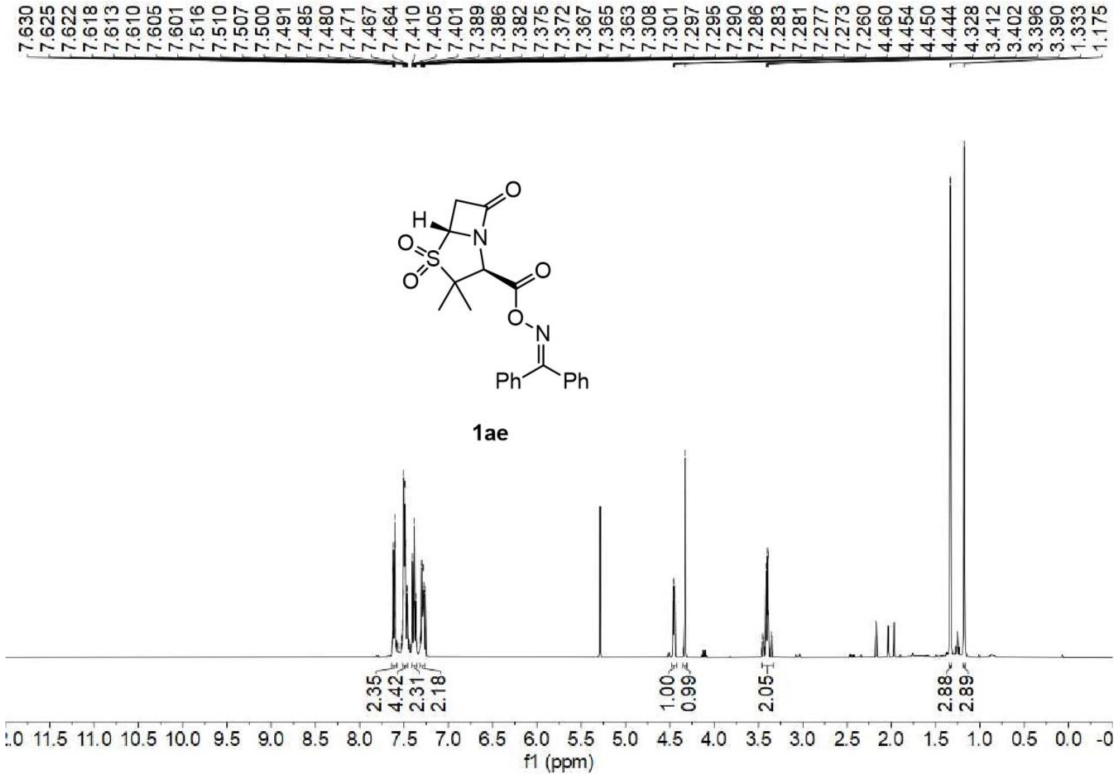
7.630
7.625
7.622
7.618
7.613
7.610
7.605
7.601
7.516
7.510
7.507
7.500
7.491
7.485
7.480
7.471
7.467
7.464
7.410
7.405
7.401
7.389
7.386
7.382
7.375
7.372
7.367
7.365
7.363
7.308
7.301
7.297
7.295
7.290
7.286
7.283
7.281
7.277
7.273
7.260
4.460
4.454
4.450
4.444
4.328
3.412
3.402
3.396
3.390
1.333
1.175
H
O
N
O
S
O
O
O
N
Ph
Ph
1ae
2.35
4.42
2.31
2.18
1.00
0.99
2.05
2.88
2.89
f1 (ppm)

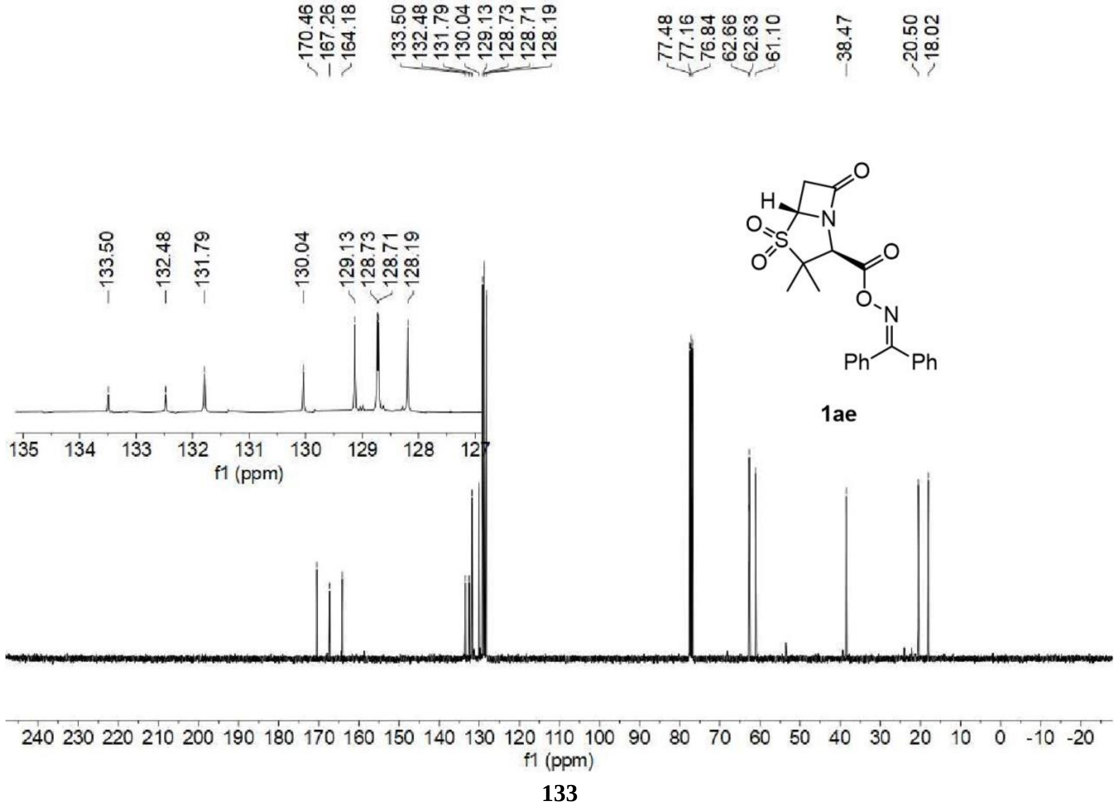
170.46
167.26
164.18
133.50
132.48
131.79
130.04
129.13
128.73
128.71
128.19
77.48
77.16
76.84
62.66
62.63
61.10
38.47
20.50
18.02
H
O
N
O
S
O
O
O
N
Ph
Ph
1ae
f1 (ppm)
f1 (ppm)

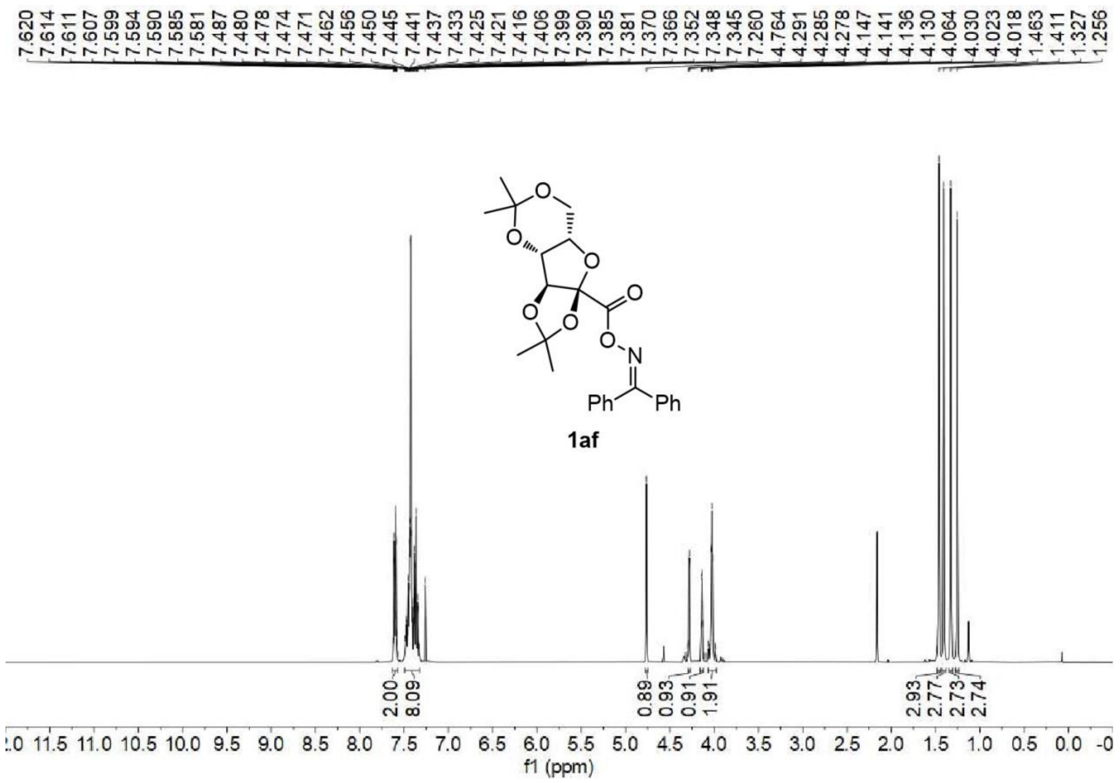
7.620 7.614 7.611 7.607 7.599 7.594 7.590 7.585 7.581 7.487 7.480 7.478 7.474 7.471 7.462 7.456 7.450 7.445 7.441 7.437 7.433 7.425 7.421 7.416 7.406 7.399 7.390 7.385 7.381 7.370 7.366 7.352 7.348 7.345 7.260 4.764 4.291 4.285 4.278 4.147 4.141 4.136 4.130 4.064 4.030 4.023 4.018 1.463 1.411 1.327 1.256
O
O
O
O
O
O
N
Ph
Ph
1af
2.00
8.09
0.89
0.93
0.91
1.91
2.93
2.77
2.73
2.74
f1 (ppm)

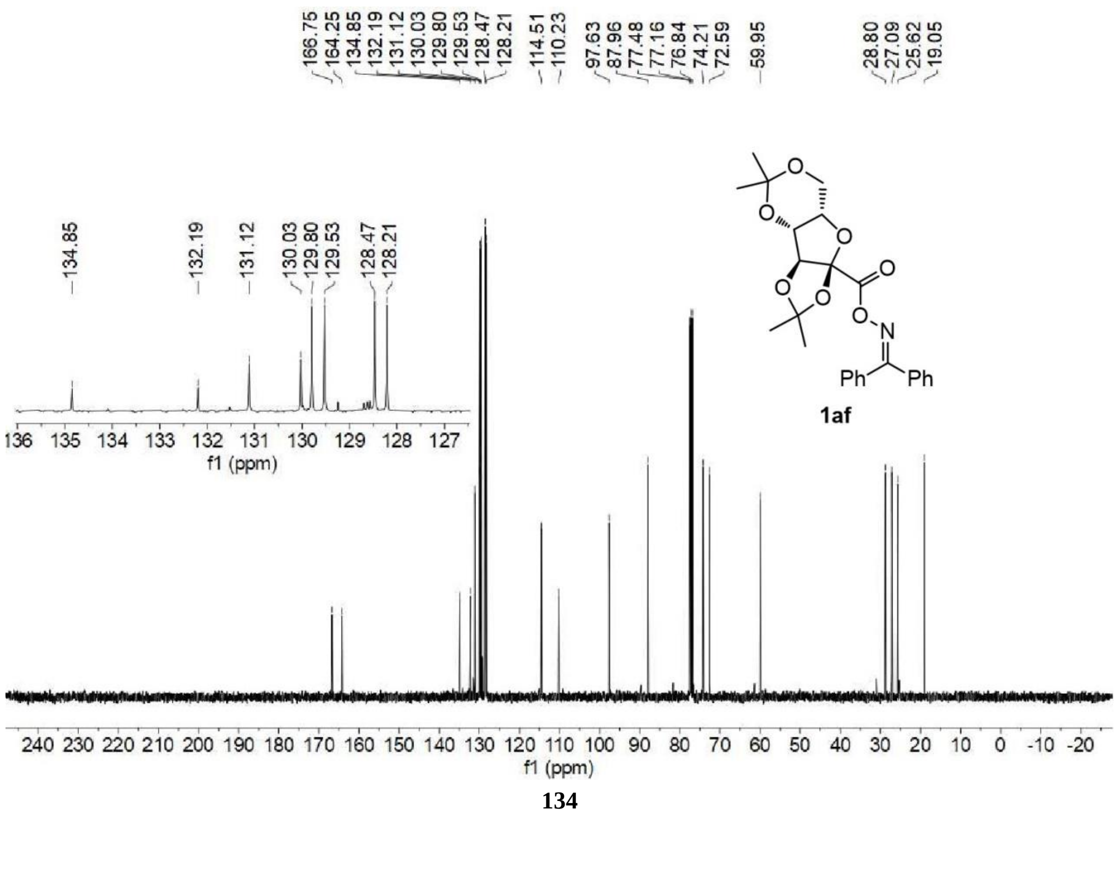
166.75 164.25 134.85 132.19 131.12 130.03 129.80 129.53 128.47 128.21
114.51 110.23
97.63 87.96 77.48 77.16 76.84 74.21 72.59
59.95
28.80 27.09 25.62 19.05
134.85
132.19
131.12
130.03 129.80 129.53
128.47 128.21
f1 (ppm)
O
O
O
O
O
O
N
Ph
Ph
1af
f1 (ppm)

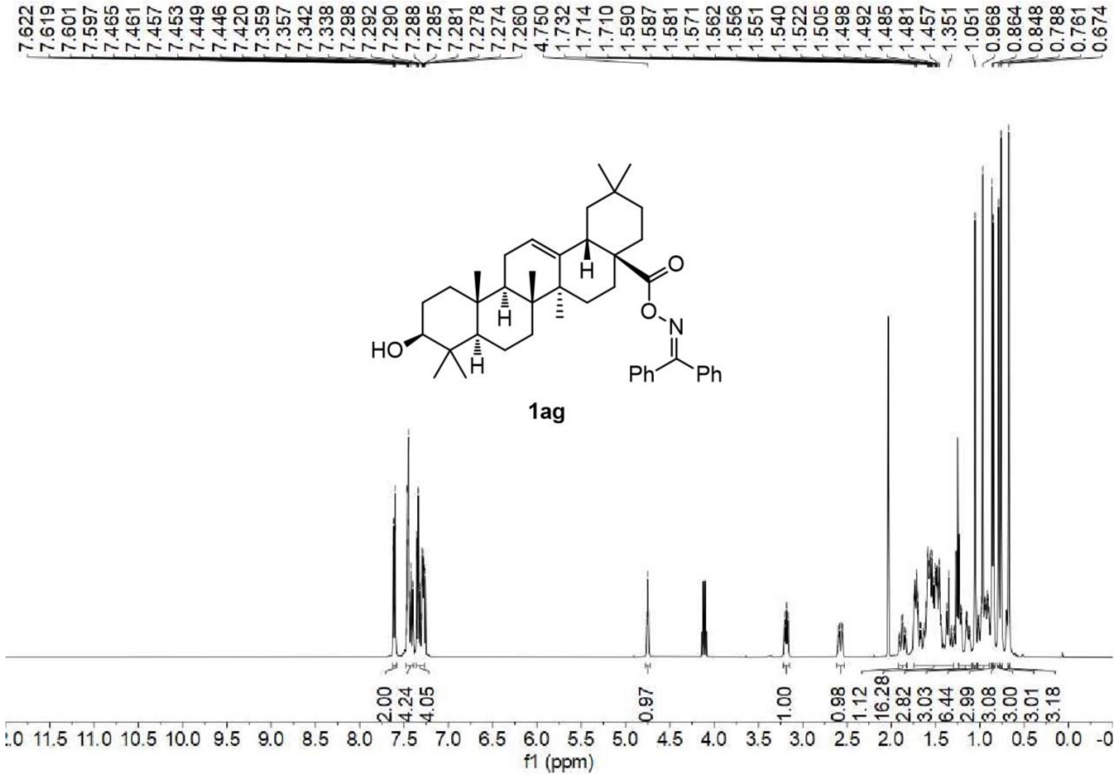

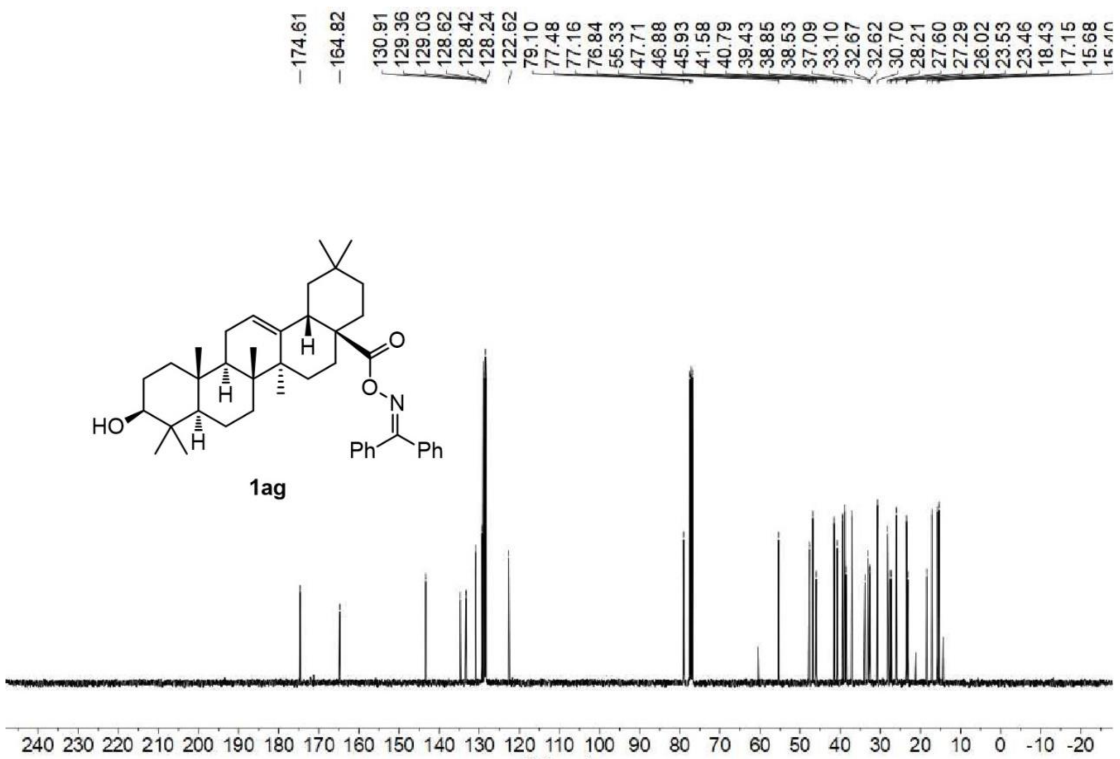

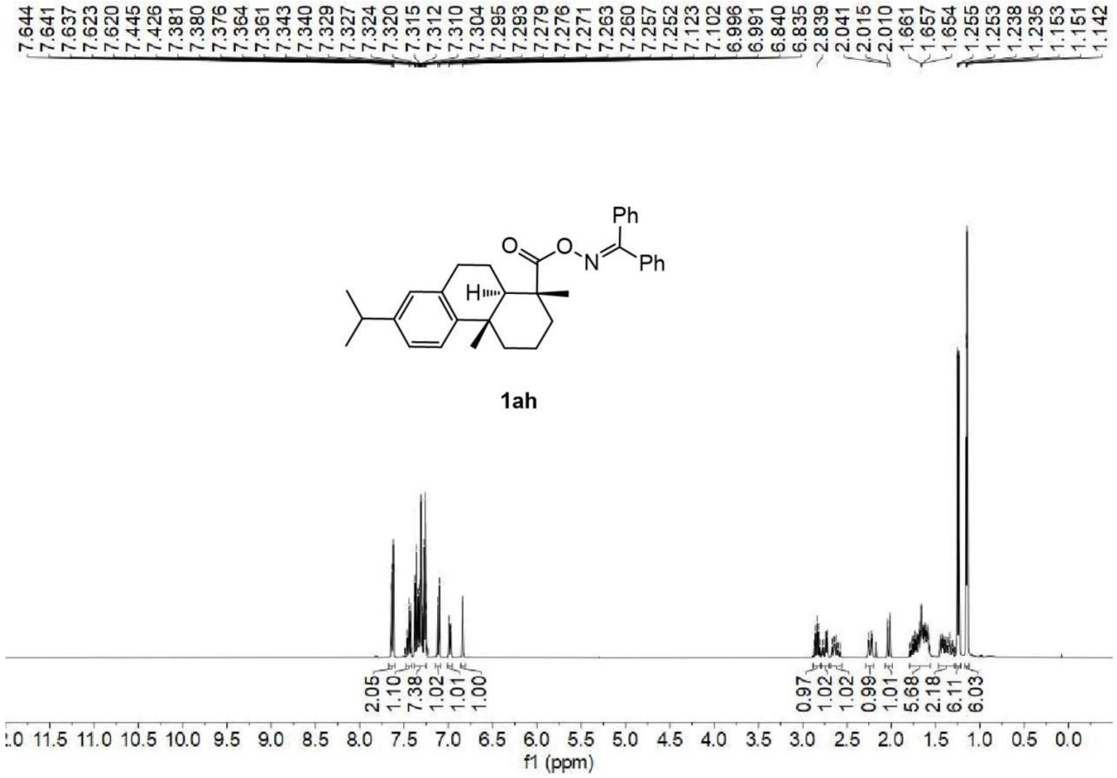
7.644 7.641 7.637 7.623 7.620 7.445 7.426 7.381 7.380 7.376 7.364 7.361 7.343 7.340 7.329 7.327 7.324 7.320 7.315 7.312 7.310 7.304 7.295 7.293 7.279 7.276 7.271 7.263 7.260 7.257 7.252 7.123 7.102 6.996 6.991 6.840 6.835 2.839 2.041 2.015 2.010 1.661 1.657 1.654 1.255 1.253 1.238 1.235 1.153 1.151 1.142
Ph
O
O
N
Ph
H
1ah
2.05 1.10 7.38 1.02 1.01 1.00
0.97 1.02 1.02 0.99 1.01 5.68 2.18 6.11 6.03
f1 (ppm)

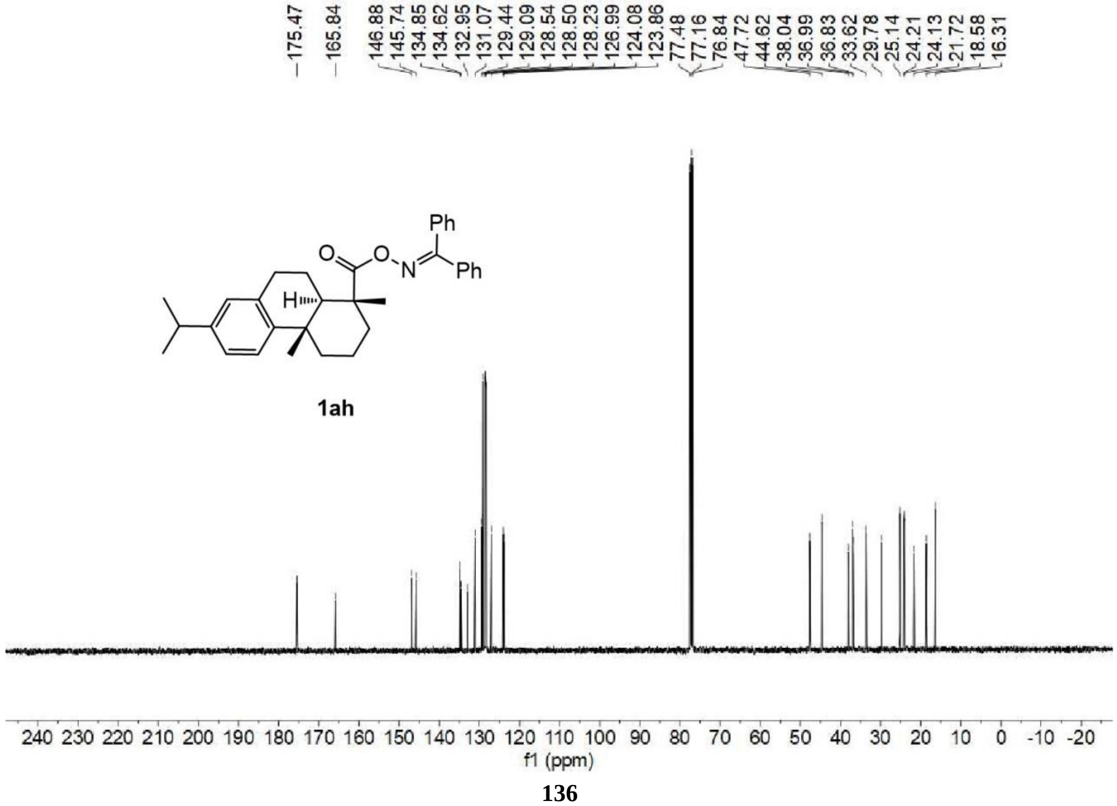
175.47
165.84
146.88 145.74 134.85 134.62 132.95 131.07 129.44 129.09 128.54 128.50 128.23 126.99 124.08 123.86 77.48 77.16 76.84 47.72 44.62 38.04 36.99 36.83 33.62 29.78 25.14 24.21 24.13 21.72 18.58 16.31
Ph
O
O
N
Ph
H
1ah
f1 (ppm)

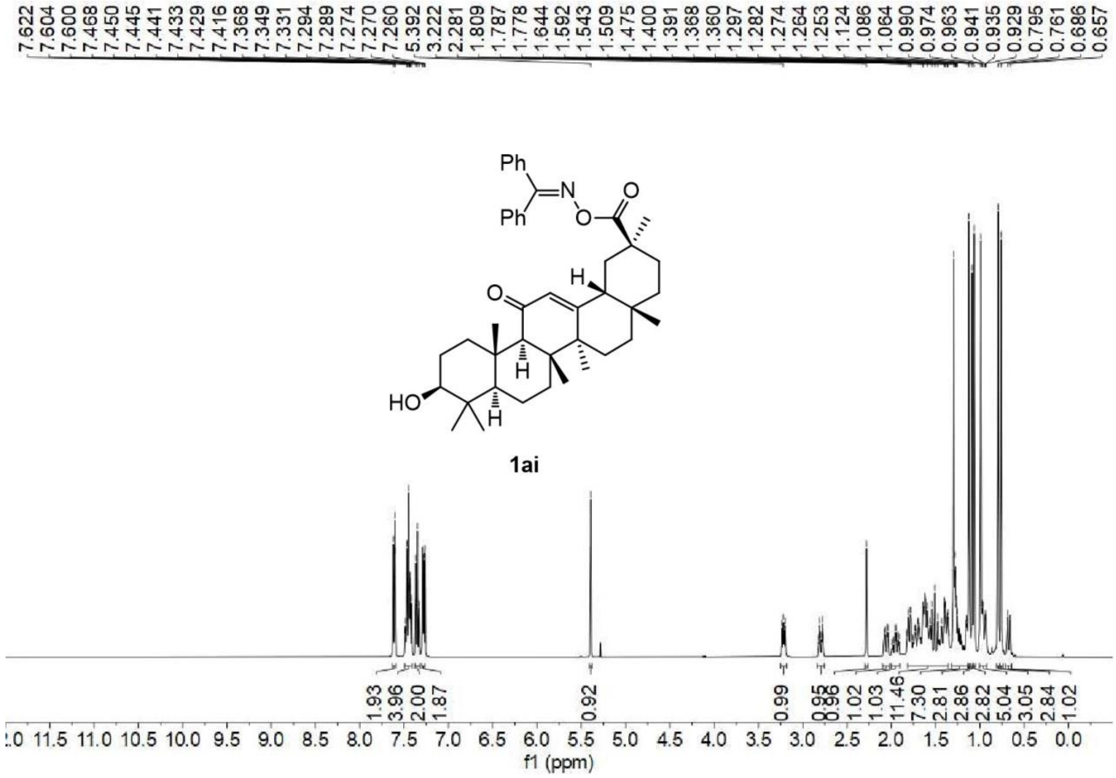
7.622 7.604 7.600 7.468 7.450 7.445 7.441 7.433 7.429 7.416 7.368 7.349 7.331 7.294 7.289 7.274 7.270 7.260 5.392 3.222 2.281 1.809 1.787 1.778 1.644 1.592 1.543 1.509 1.475 1.400 1.391 1.368 1.360 1.297 1.282 1.274 1.264 1.253 1.124 1.086 1.064 0.990 0.974 0.963 0.941 0.935 0.929 0.795 0.761 0.686 0.657
Ph
Ph
N
O
O
H
O
HO
H
H
1ai
1.93 3.96 2.00 1.87
0.92
0.99
1.02 1.03 11.46 7.30 2.81 2.86 2.82 5.04 3.05 2.84 1.02
f1 (ppm)

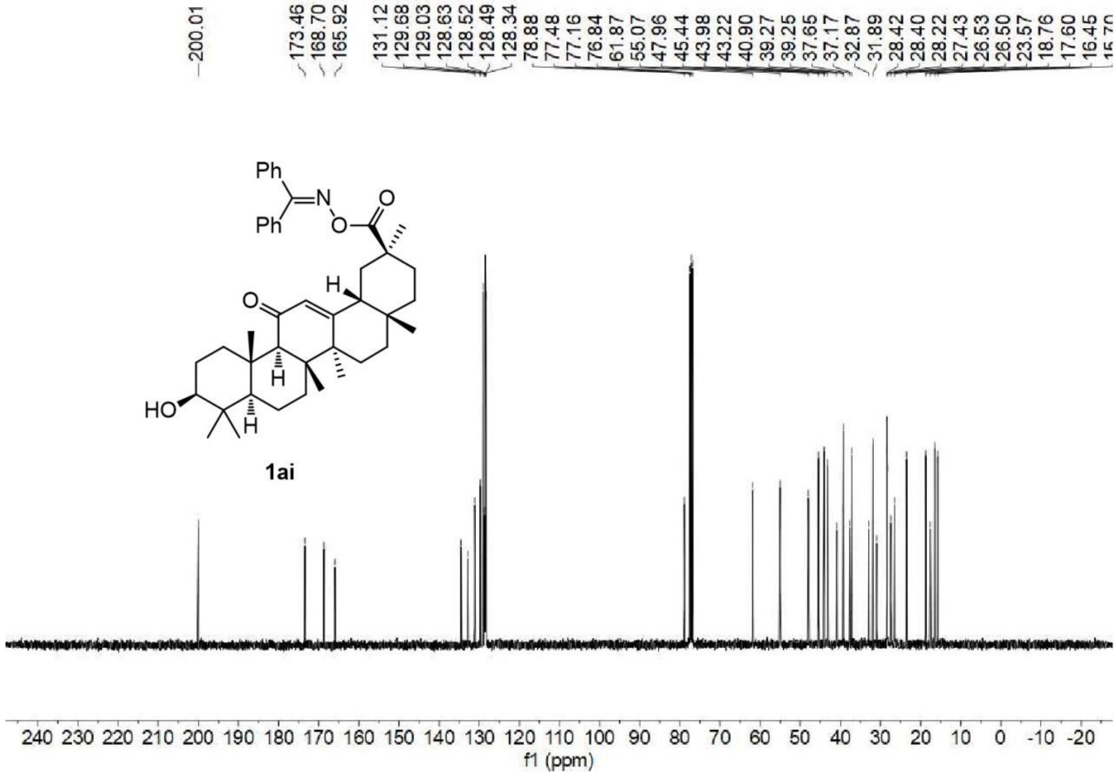
200.01
173.46 168.70 165.92
131.12 129.68 129.03 128.63 128.52 128.49 128.34
78.88 77.48 77.16 76.84 61.87 55.07 47.96 45.44 43.98 43.22 40.90 39.27 39.25 37.65 37.17 32.87 31.89 28.42 28.40 28.22 27.43 26.53 26.50 23.57 18.76 17.60 16.45
Ph
Ph
N
O
O
H
O
HO
H
H
1ai
240 230 220 210 200 190 180 170 160 150 140 130 120 110 100 90 80 70 60 50 40 30 20 10 0 -10 -20
f1 (ppm)

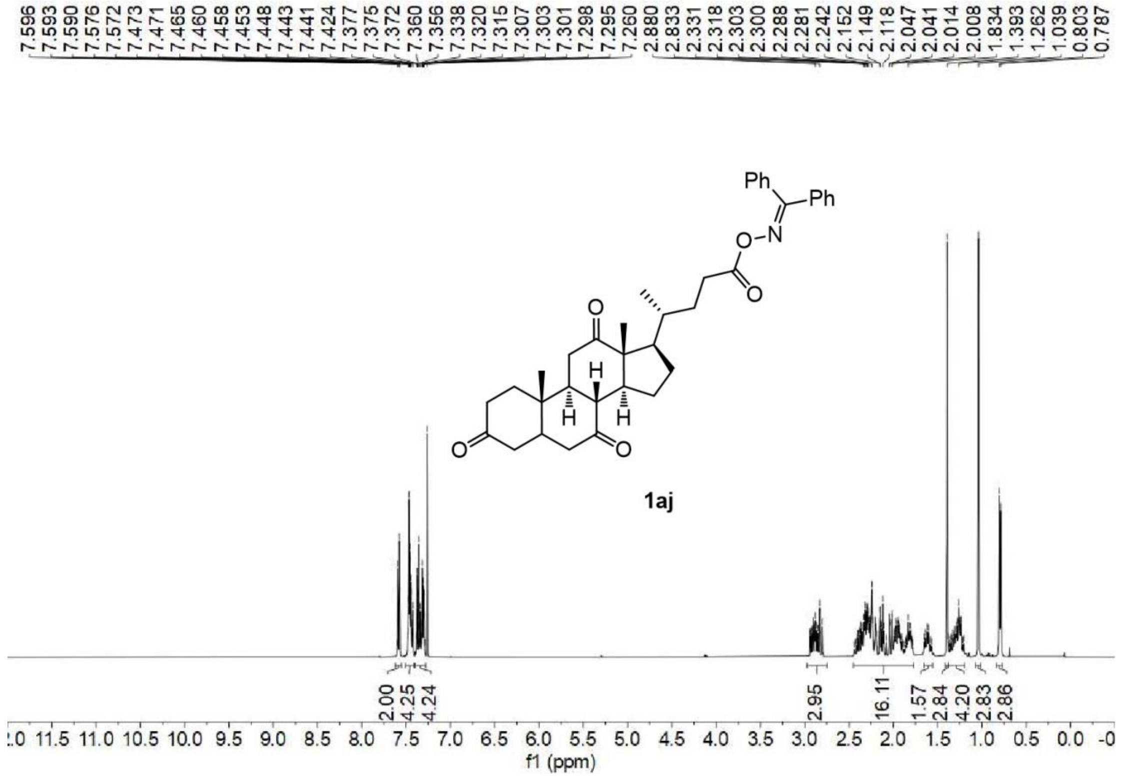

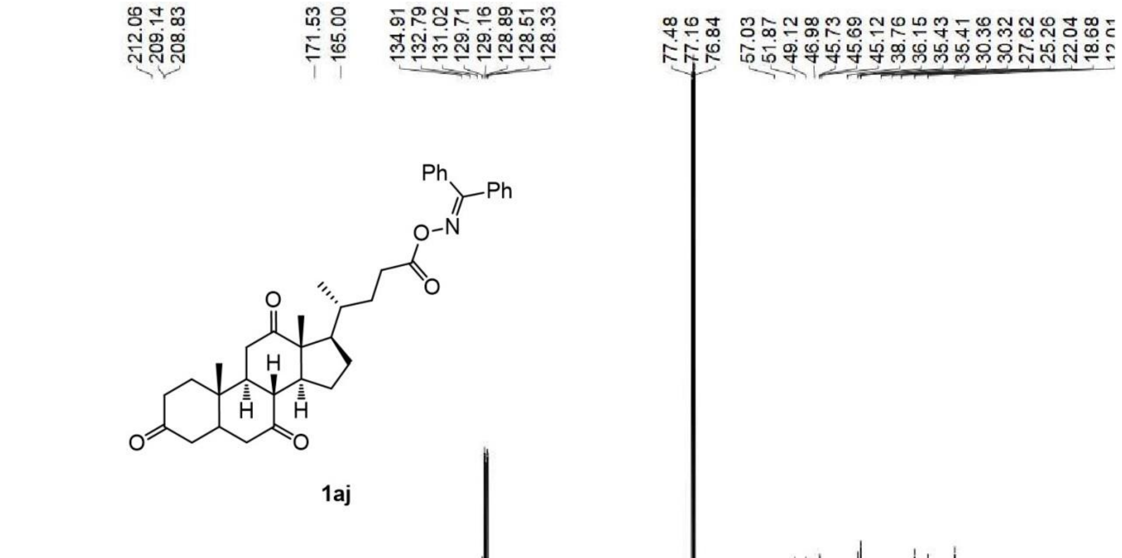

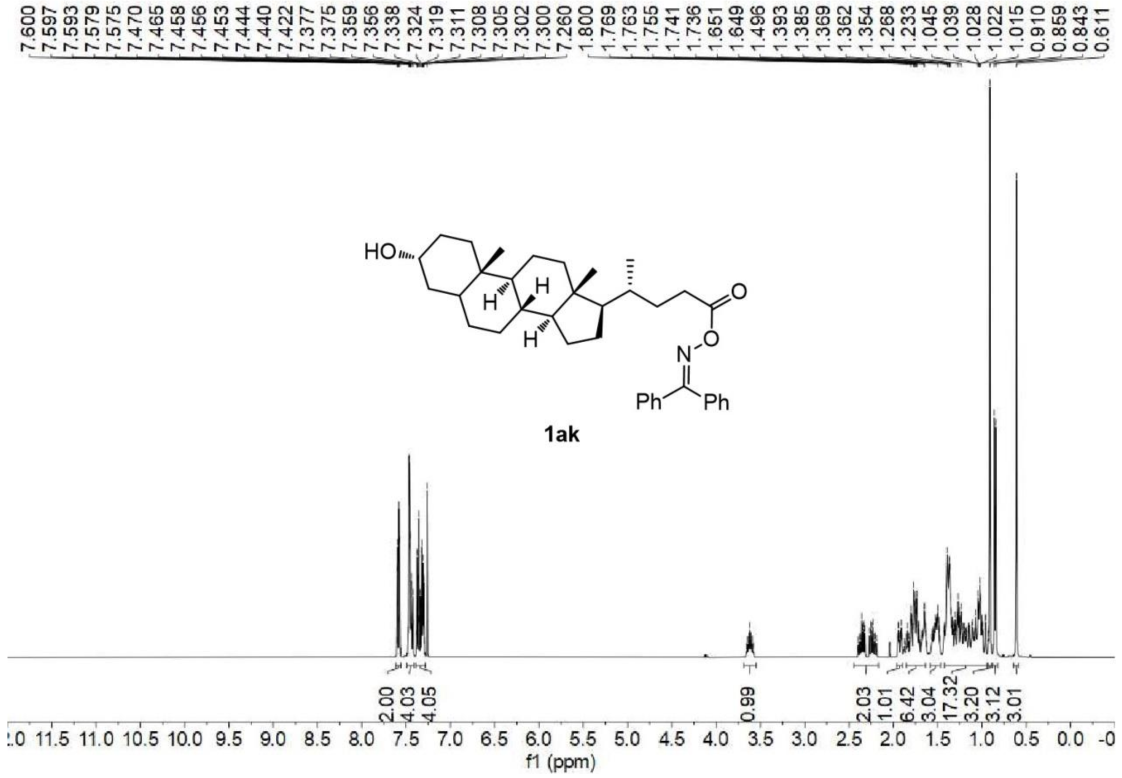
7.600 7.597 7.593 7.579 7.575 7.470 7.465 7.458 7.456 7.453 7.444 7.440 7.422 7.377 7.375 7.359 7.356 7.338 7.324 7.319 7.311 7.308 7.305 7.302 7.300 7.260 1.800 1.769 1.763 1.755 1.741 1.736 1.651 1.649 1.496 1.393 1.385 1.369 1.362 1.354 1.268 1.233 1.045 1.039 1.028 1.022 1.015 0.910 0.859 0.843 0.611
HO
H
H
H
O
O
N
Ph
Ph
1ak
2.00
4.03
4.05
0.99
2.03
1.01
6.42
3.04
17.32
3.20
3.12
3.01
f1 (ppm)

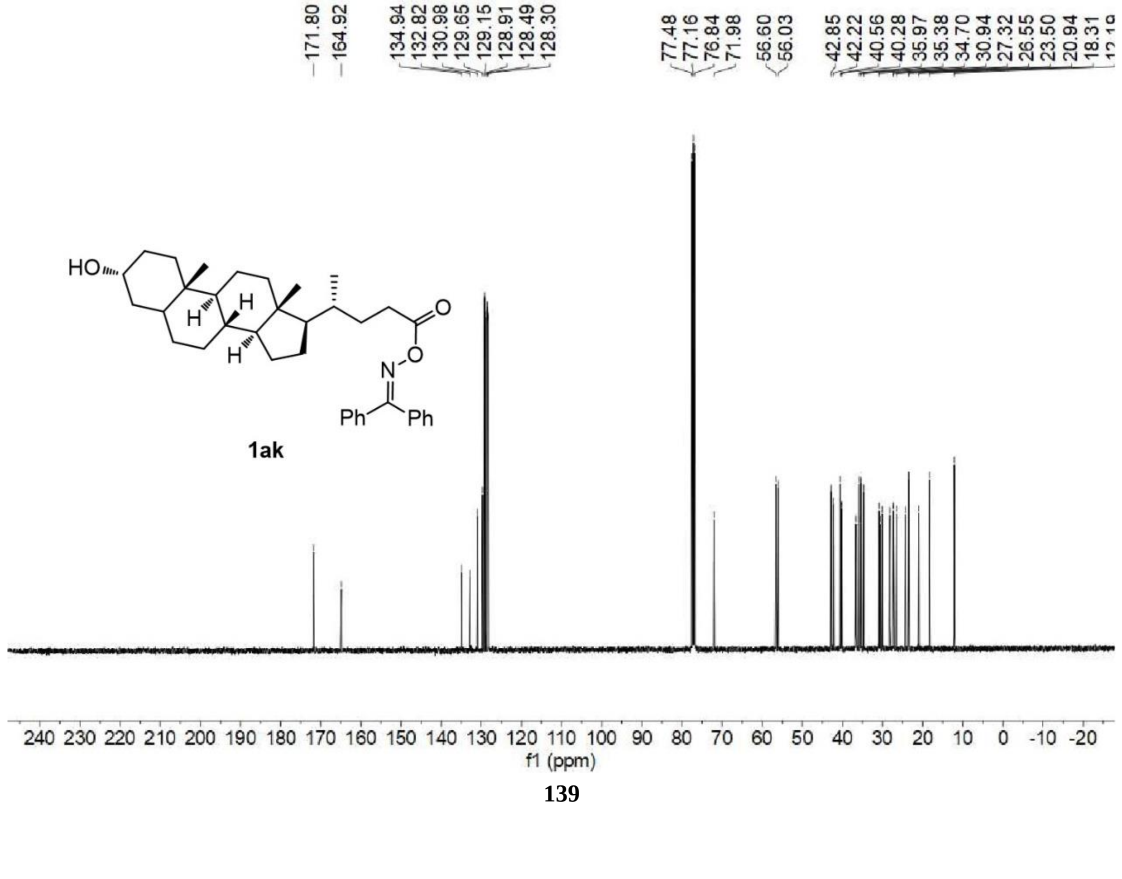
171.80
164.92
134.94 132.82 130.98 129.65 129.15 128.91 128.49 128.30
77.48 77.16 76.84 71.98
56.60 56.03
42.85 42.22 40.56 40.28 35.97 35.38 34.70 30.94 27.32 26.55 23.50 20.94 18.31
HO
H
H
H
O
O
N
Ph
Ph
1ak
240 230 220 210 200 190 180 170 160 150 140 130 120 110 100 90 80 70 60 50 40 30 20 10 0 -10 -20
f1 (ppm)

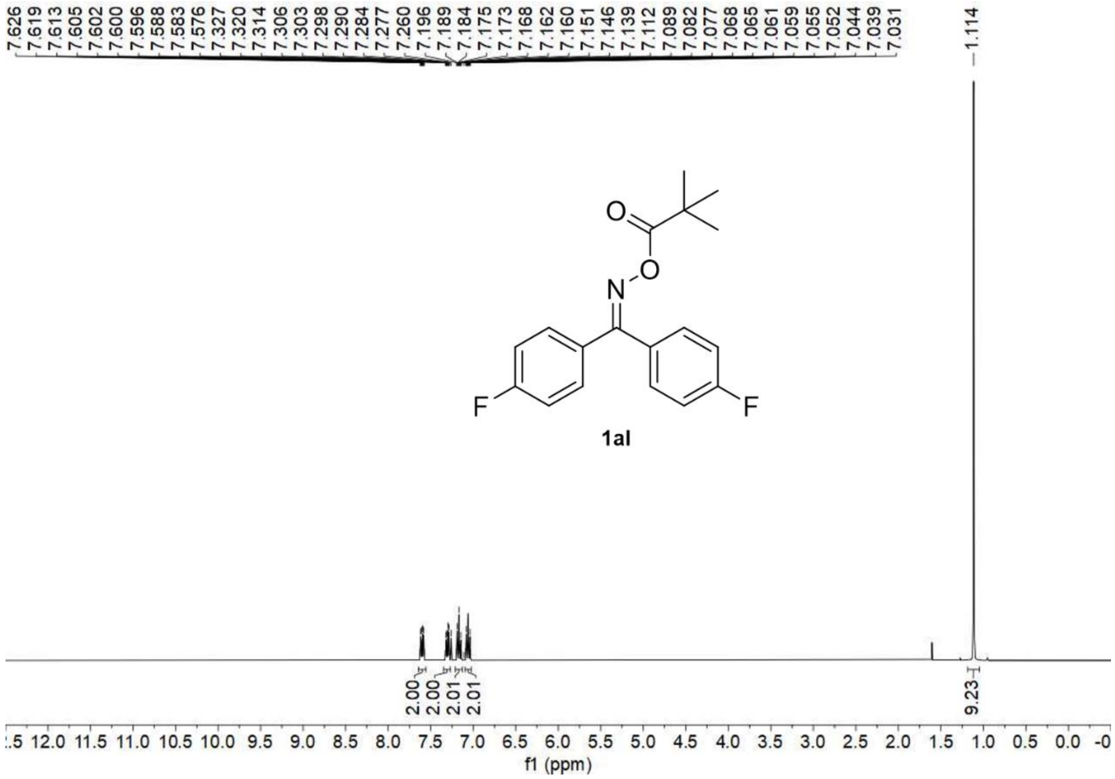

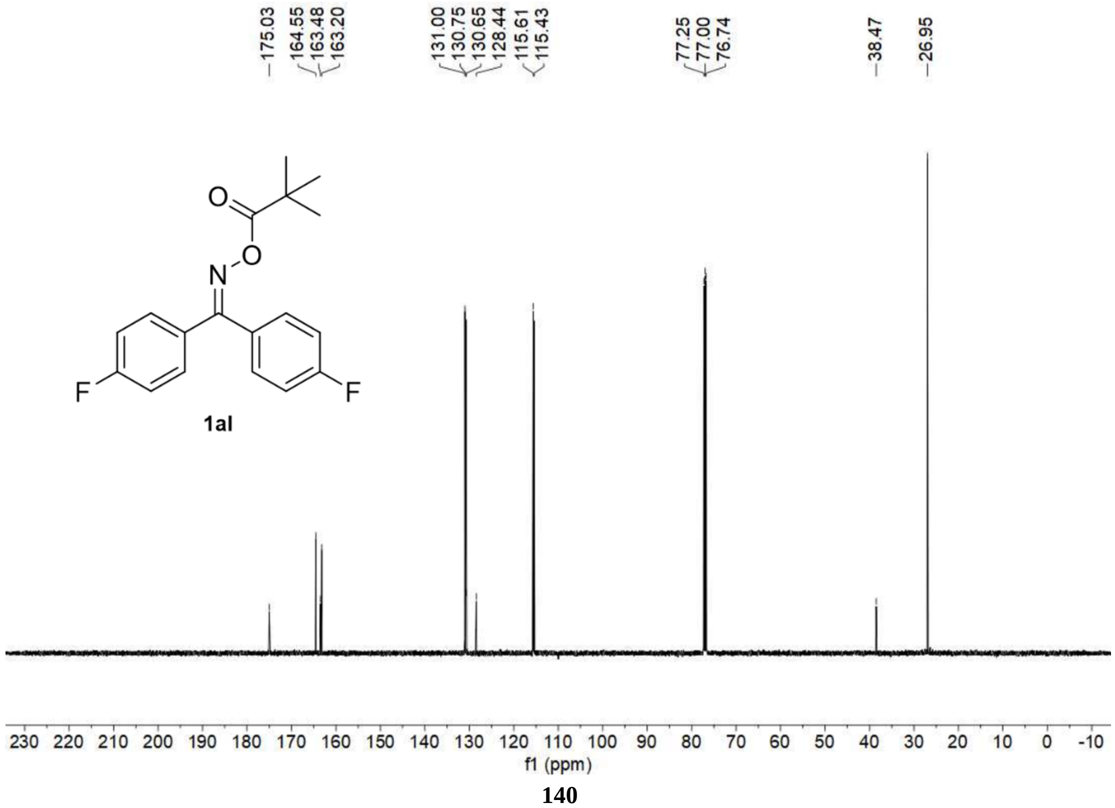

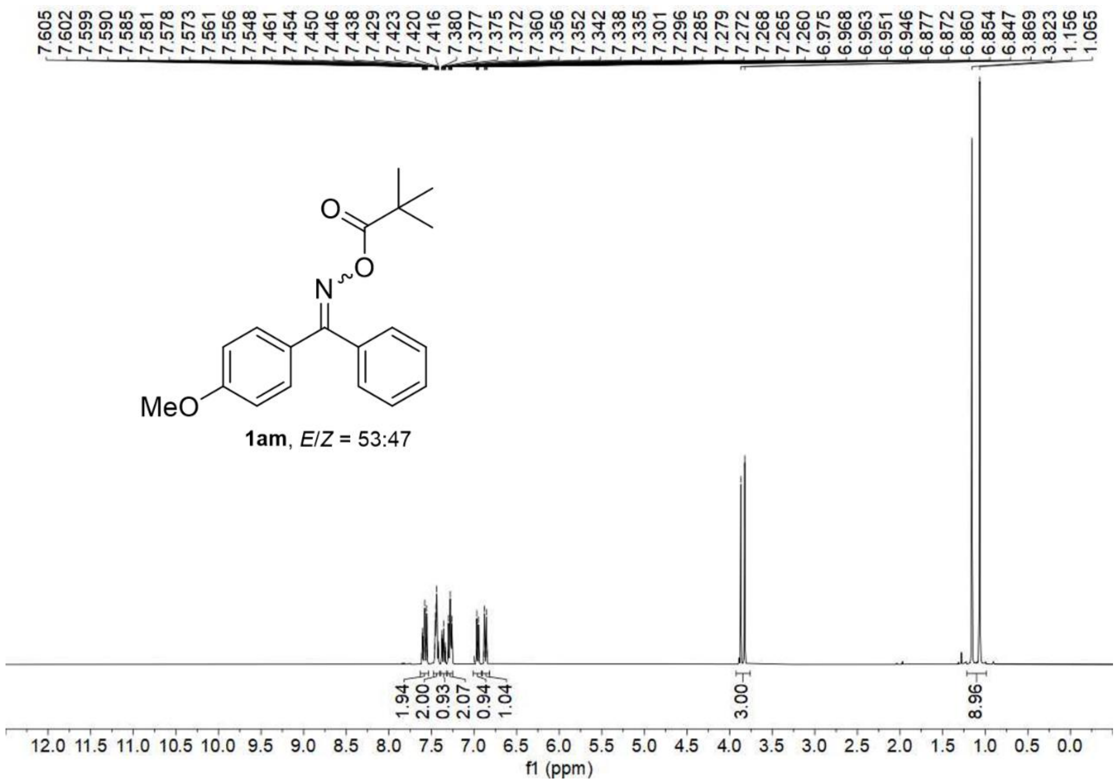
7.605 7.602 7.599 7.590 7.585 7.581 7.578 7.573 7.561 7.556 7.548 7.461 7.454 7.450 7.446 7.438 7.429 7.423 7.420 7.416 7.380 7.377 7.375 7.372 7.360 7.356 7.352 7.342 7.338 7.335 7.301 7.296 7.285 7.279 7.272 7.268 7.265 7.260 6.975 6.968 6.963 6.951 6.946 6.877 6.872 6.860 6.854 6.847 3.869 3.823 1.156 1.065
MeO
1am, E/Z = 53:47
1.94 2.00 0.93 2.07 0.94 1.04
3.00
8.96
f1 (ppm)

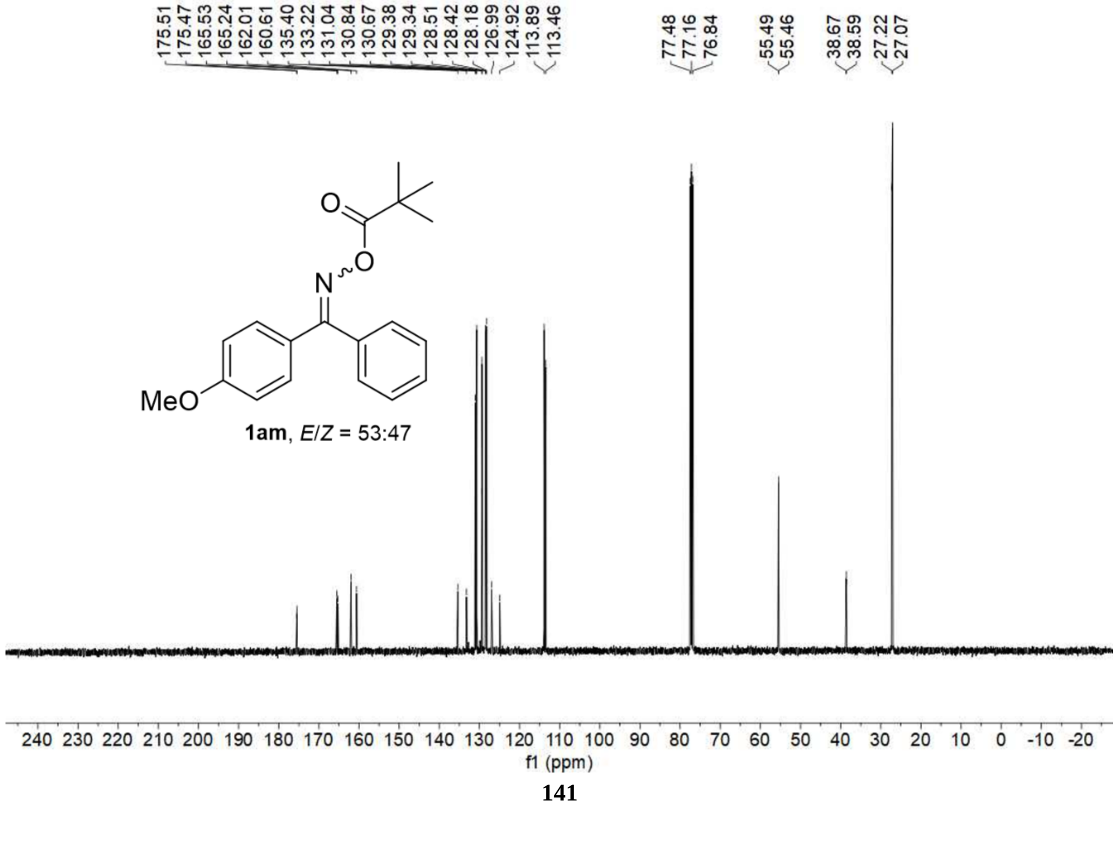
175.51 175.47 165.53 165.24 162.01 160.61 135.40 133.22 131.04 130.84 130.67 129.38 129.34 128.51 128.42 128.18 126.99 124.92 113.89 113.46
77.48 77.16 76.84
55.49 55.46
38.67 38.59
27.22 27.07
MeO
1am, E/Z = 53:47
f1 (ppm)

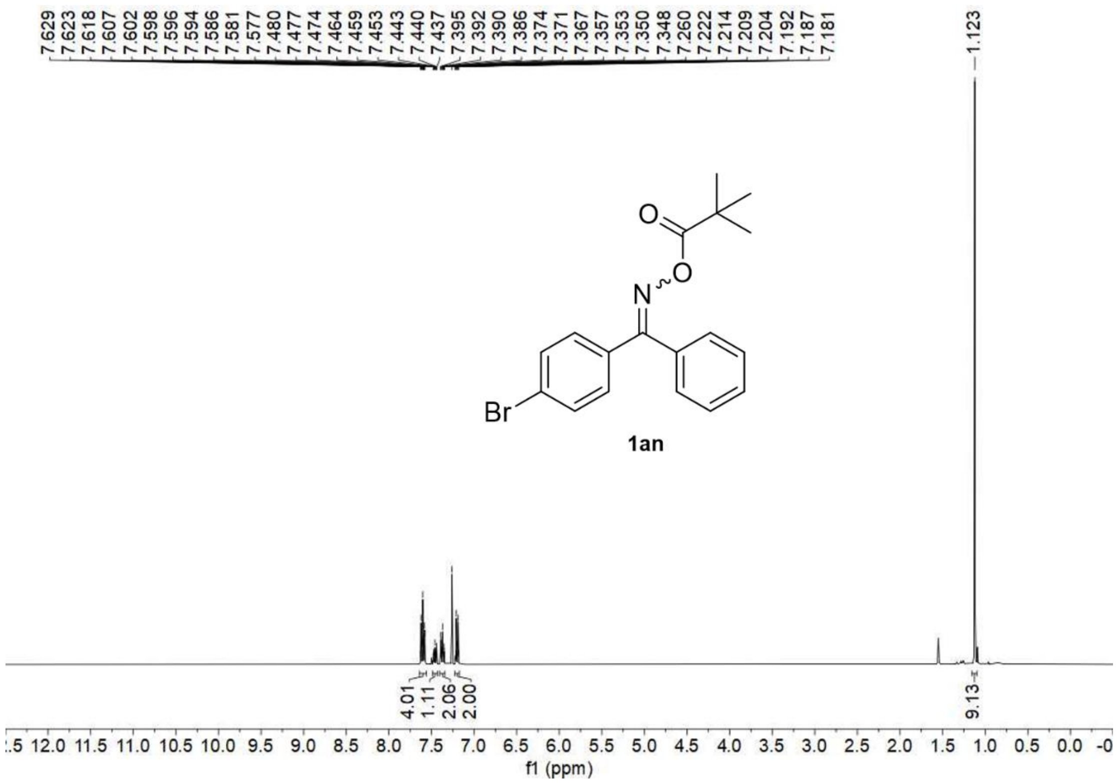
7.629
7.623
7.618
7.607
7.602
7.598
7.596
7.594
7.586
7.581
7.577
7.480
7.477
7.474
7.464
7.459
7.453
7.443
7.440
7.437
7.395
7.392
7.390
7.386
7.374
7.371
7.367
7.357
7.353
7.350
7.348
7.260
7.222
7.214
7.209
7.204
7.192
7.187
7.181
1.123
O
O
N
Br
4.01
1.11
2.06
2.00
9.13
f1 (ppm)

1an

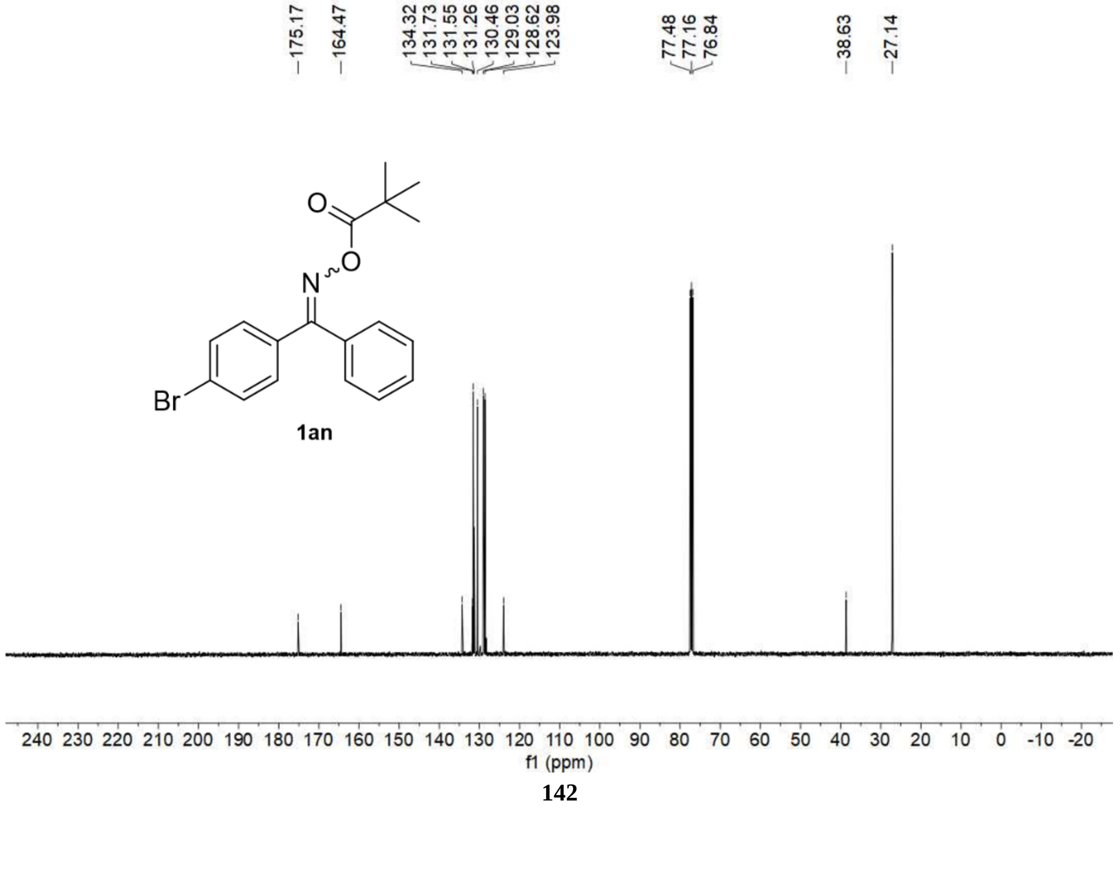
175.17
164.47
134.32
131.73
131.55
131.26
130.46
129.03
128.62
123.98
77.48
77.16
76.84
38.63
27.14
O
O
N
Br
f1 (ppm)

1an

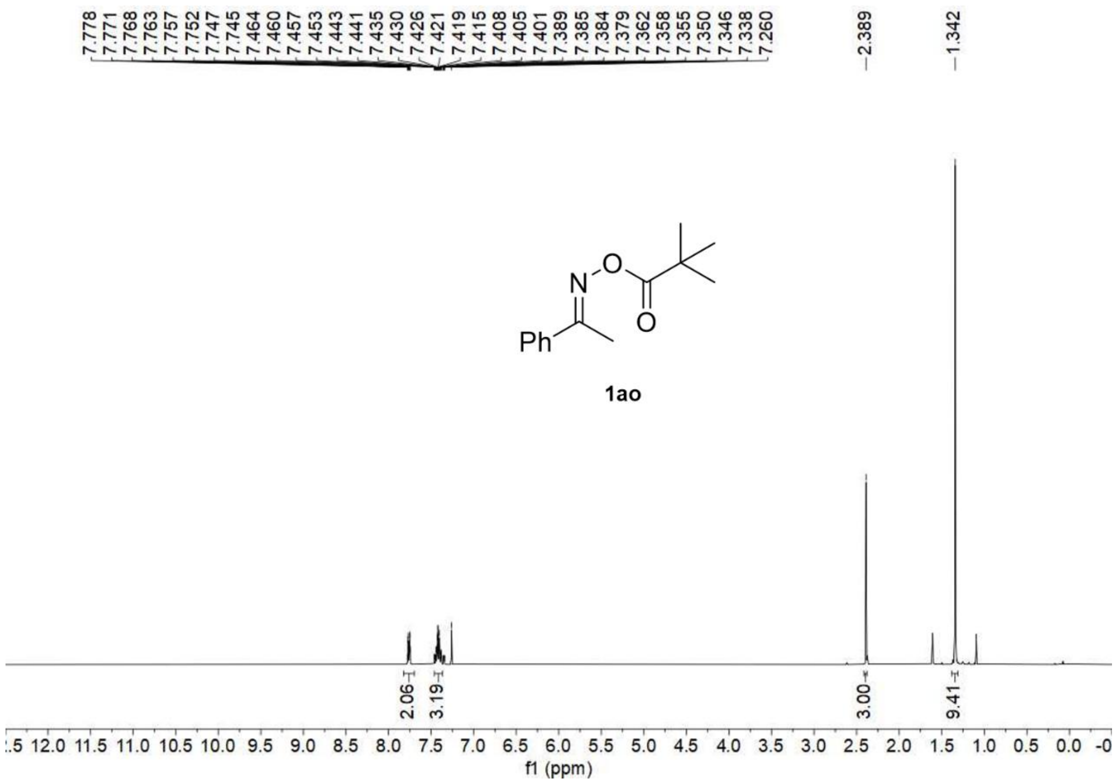

7.778
7.771
7.768
7.763
7.757
7.752
7.747
7.745
7.464
7.460
7.457
7.453
7.443
7.441
7.435
7.430
7.426
7.421
7.419
7.415
7.408
7.405
7.401
7.389
7.385
7.384
7.379
7.362
7.358
7.355
7.350
7.346
7.338
7.260
2.389
1.342
N
O
O
Ph
1ao
2.06
3.19
3.00
9.41
f1 (ppm)

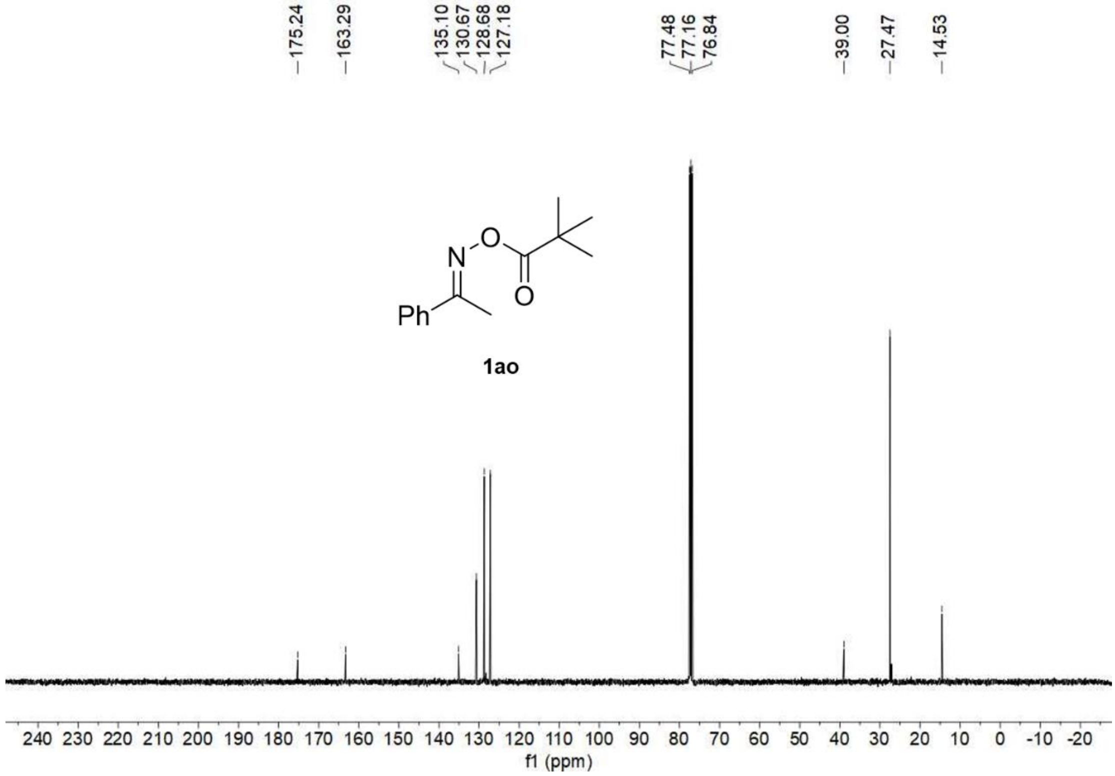

175.24
163.29
135.10
130.67
128.68
127.18
77.48
77.16
76.84
39.00
27.47
14.53
N
O
O
Ph
1ao
f1 (ppm)

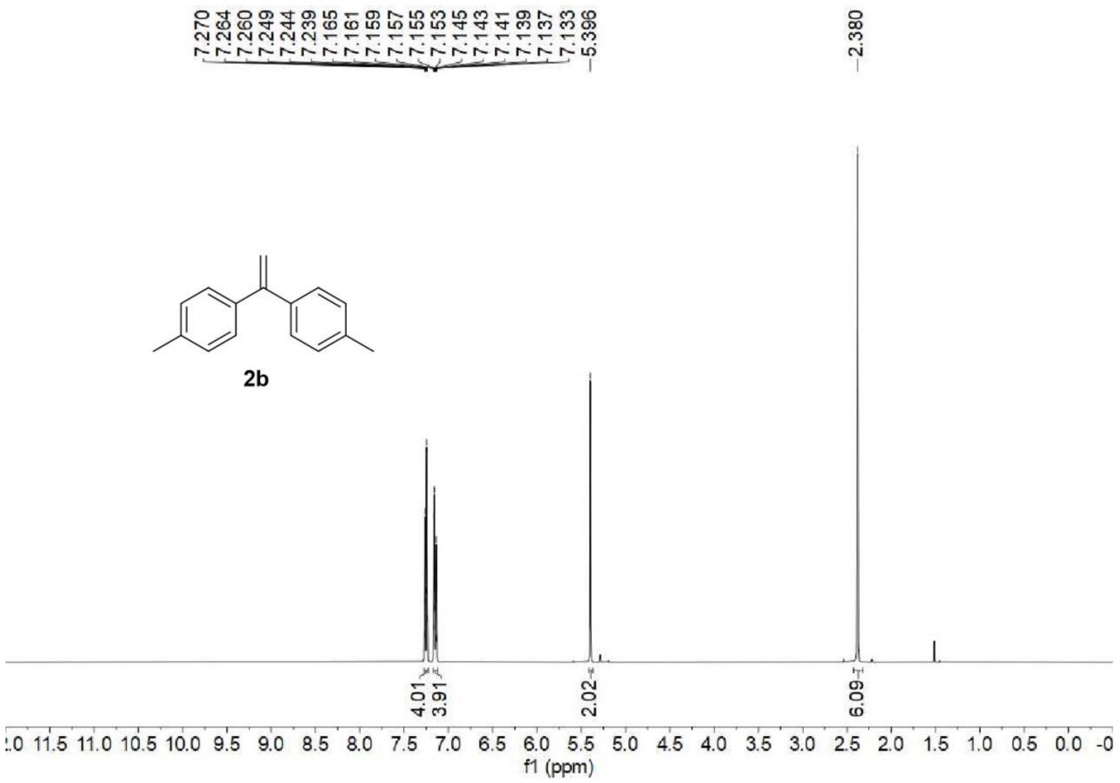
7.270
7.264
7.260
7.249
7.244
7.239
7.165
7.161
7.159
7.157
7.155
7.153
7.145
7.143
7.141
7.139
7.137
7.133
5.396
2.380
2b
4.01
3.91
2.02
6.09
f1 (ppm)

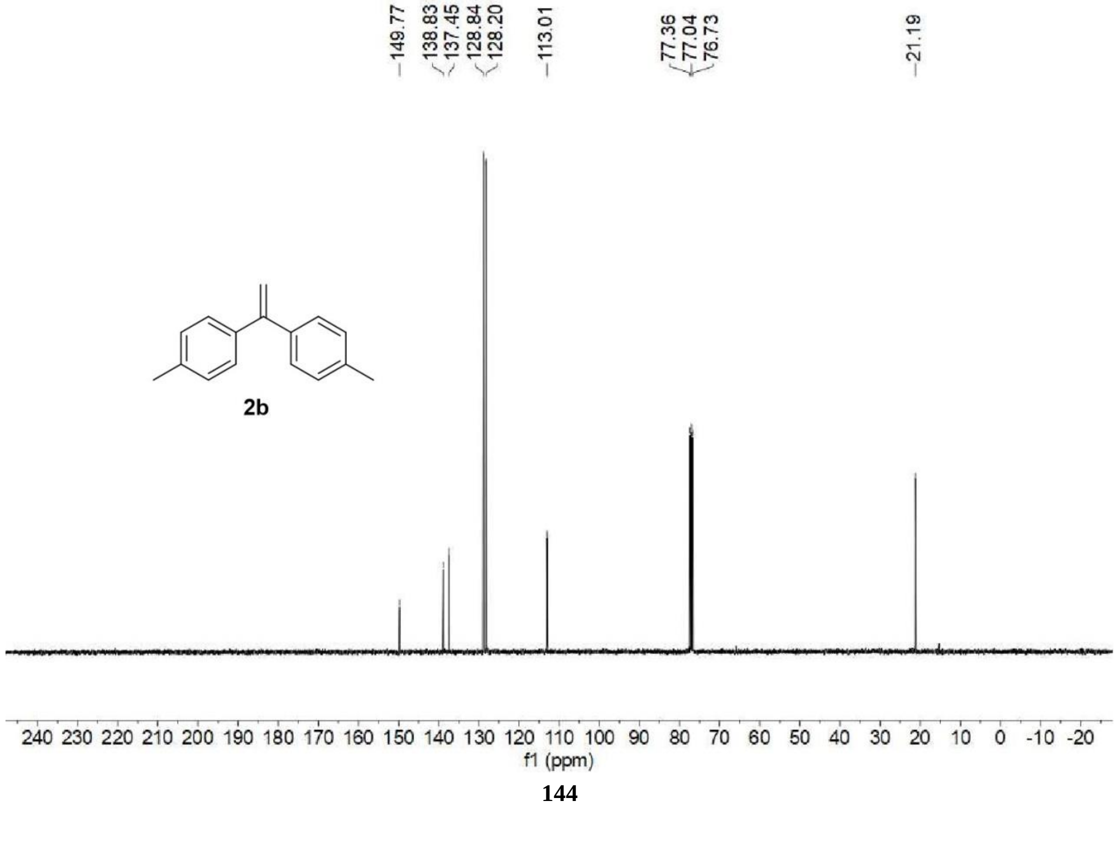
149.77
138.83
137.45
128.84
128.20
113.01
77.36
77.04
76.73
21.19
2b
f1 (ppm)

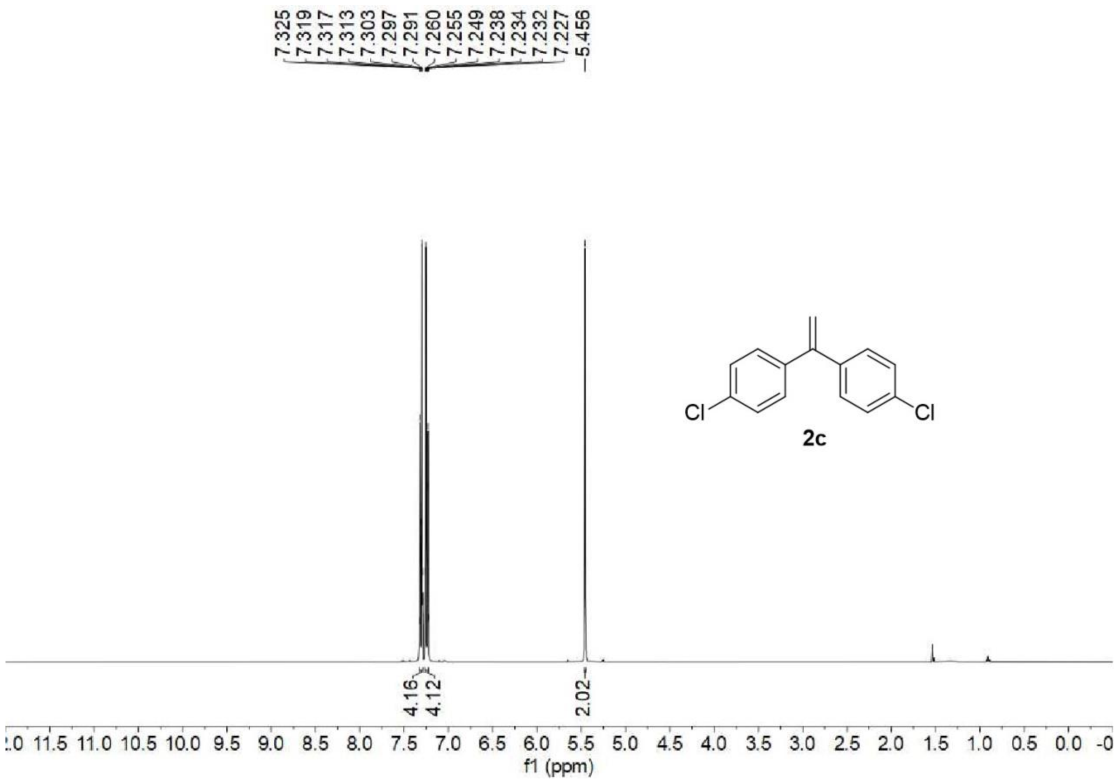
7.325
7.319
7.317
7.313
7.303
7.297
7.291
7.260
7.255
7.249
7.238
7.234
7.232
7.227
5.456
4.16
4.12
2.02
Cl
Cl
2c
f1 (ppm)

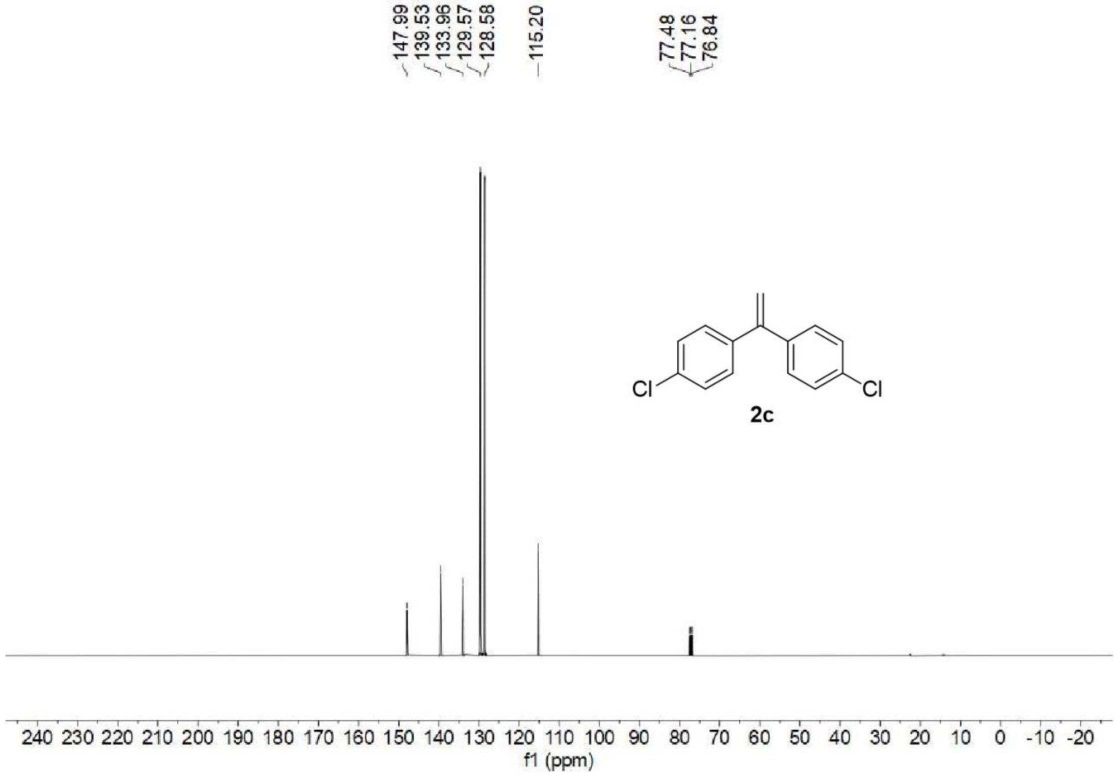
147.99
139.53
133.96
129.57
128.58
115.20
77.48
77.16
76.84
Cl
Cl
2c
f1 (ppm)

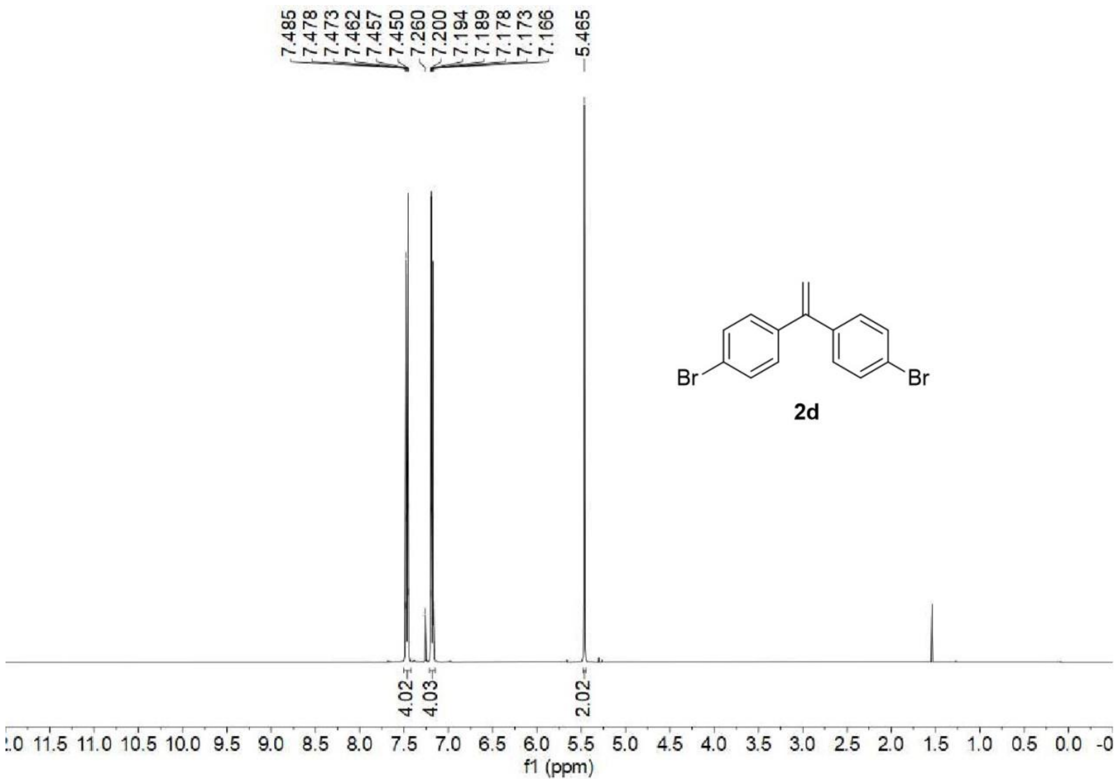
7.485
7.478
7.473
7.462
7.457
7.450
7.260
7.200
7.194
7.189
7.178
7.173
7.166
5.465
Br
Br
2d
4.02
4.03
2.02
f1 (ppm)

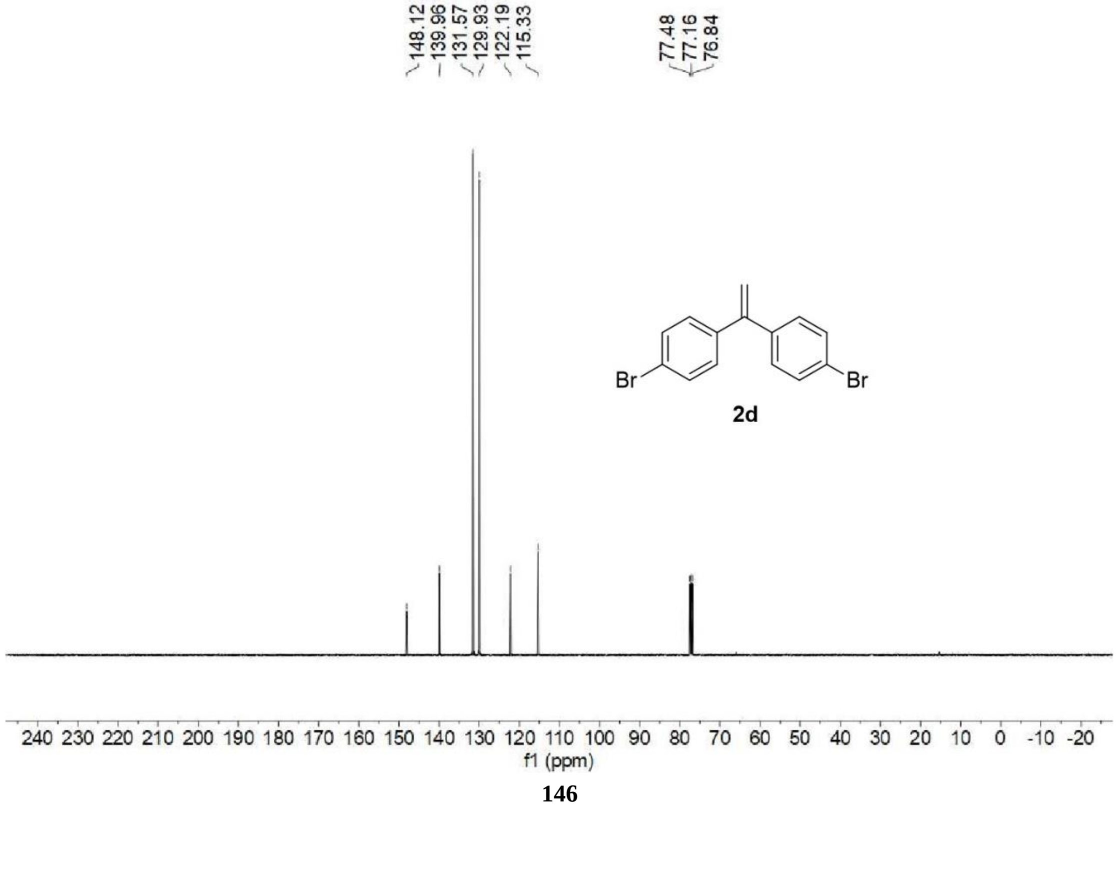
148.12
139.96
131.57
129.93
122.19
115.33
77.48
77.16
76.84
Br
Br
2d
f1 (ppm)

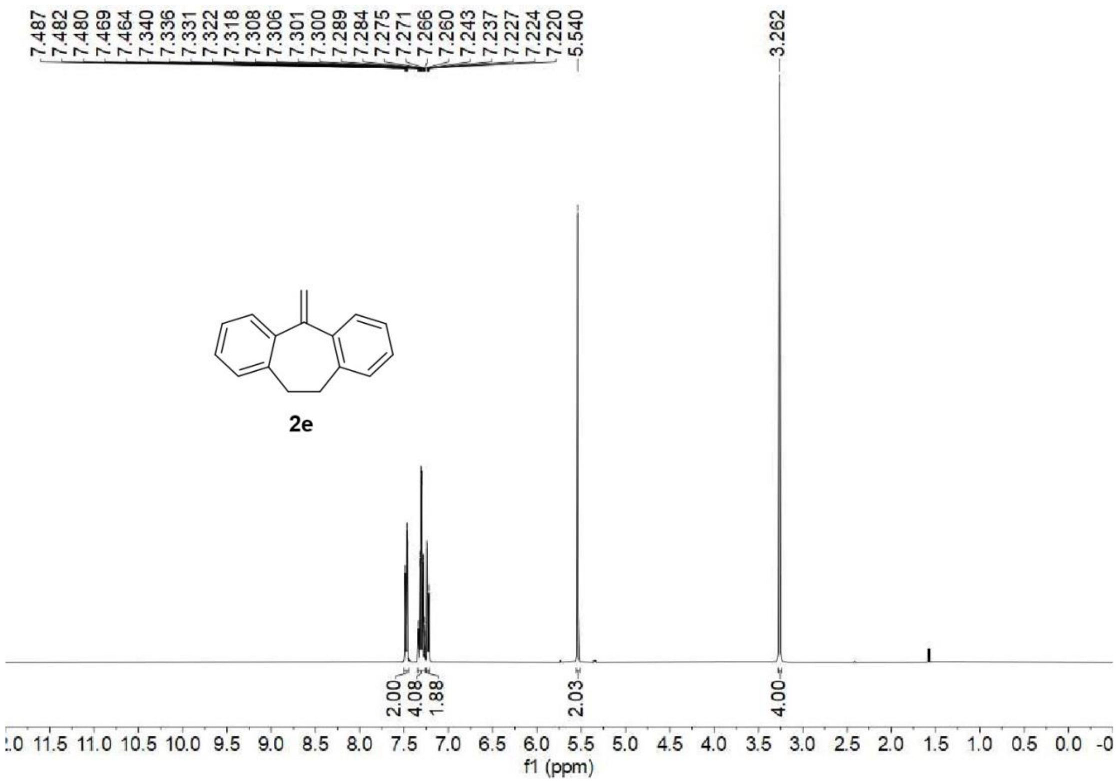
7.487
7.482
7.480
7.469
7.464
7.340
7.336
7.331
7.322
7.318
7.308
7.306
7.301
7.300
7.289
7.284
7.275
7.271
7.266
7.260
7.243
7.237
7.227
7.224
7.220
5.540
3.262
2e
2.00
4.08
1.88
2.03
4.00
f1 (ppm)

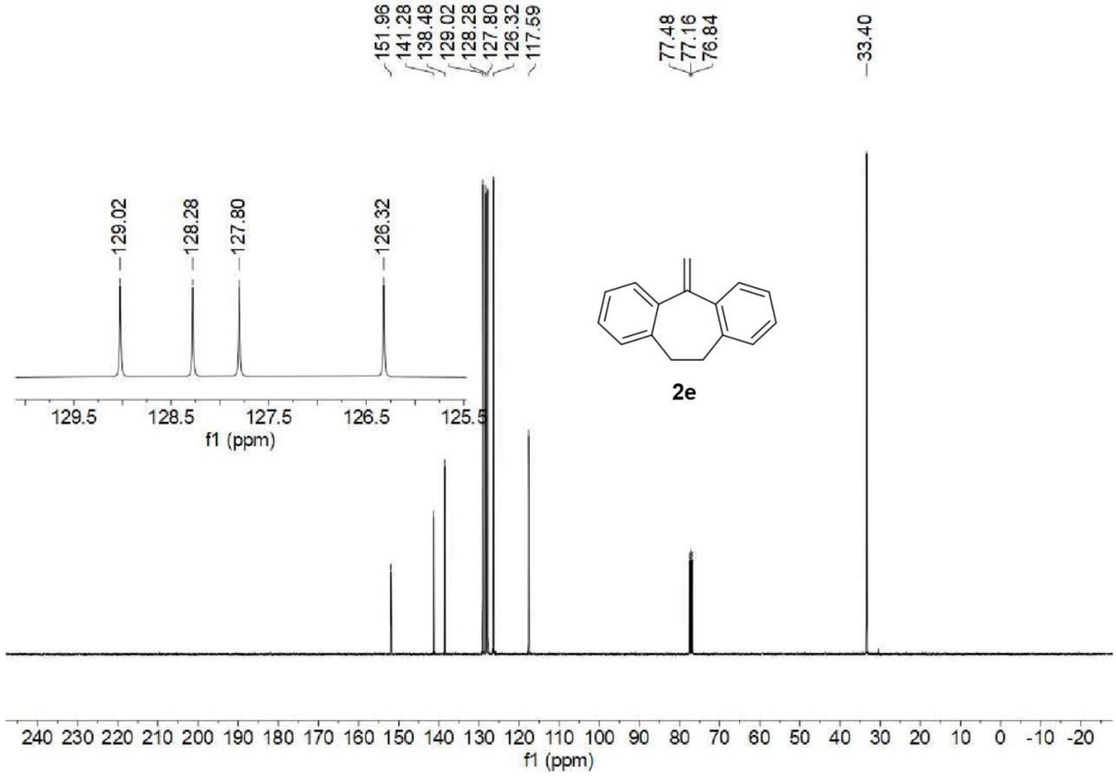
151.96
141.28
138.48
129.02
128.28
127.80
126.32
117.59
77.48
77.16
76.84
33.40
129.02
128.28
127.80
126.32
f1 (ppm)
2e
f1 (ppm)

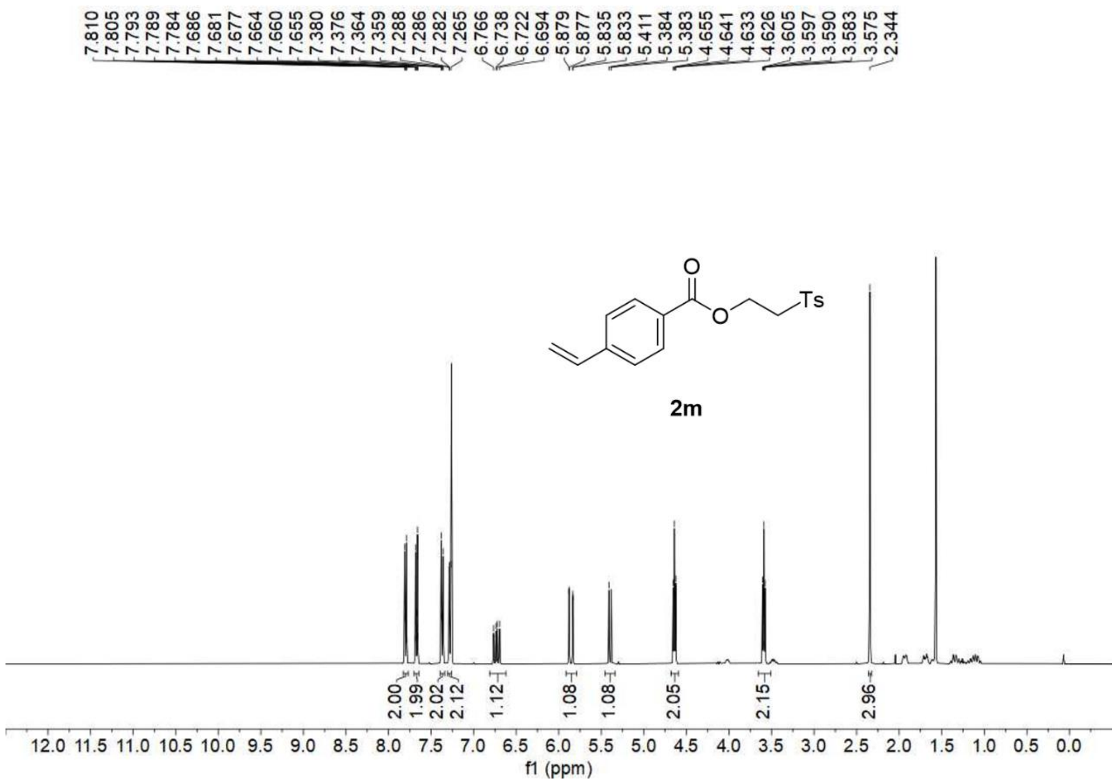
7.810
7.805
7.793
7.789
7.784
7.686
7.681
7.677
7.664
7.660
7.655
7.380
7.376
7.364
7.359
7.288
7.286
7.282
7.265
6.766
6.738
6.722
6.694
5.879
5.877
5.835
5.833
5.411
5.384
5.383
4.655
4.641
4.633
4.626
3.605
3.597
3.590
3.583
3.575
2.344
O
O
Ts
2m
2.00
1.99
2.02
2.12
1.12
1.08
1.08
2.05
2.15
2.96
12.0 11.5 11.0 10.5 10.0 9.5 9.0 8.5 8.0 7.5 7.0 6.5 6.0 5.5 5.0 4.5 4.0 3.5 3.0 2.5 2.0 1.5 1.0 0.5 0.0
f1 (ppm)

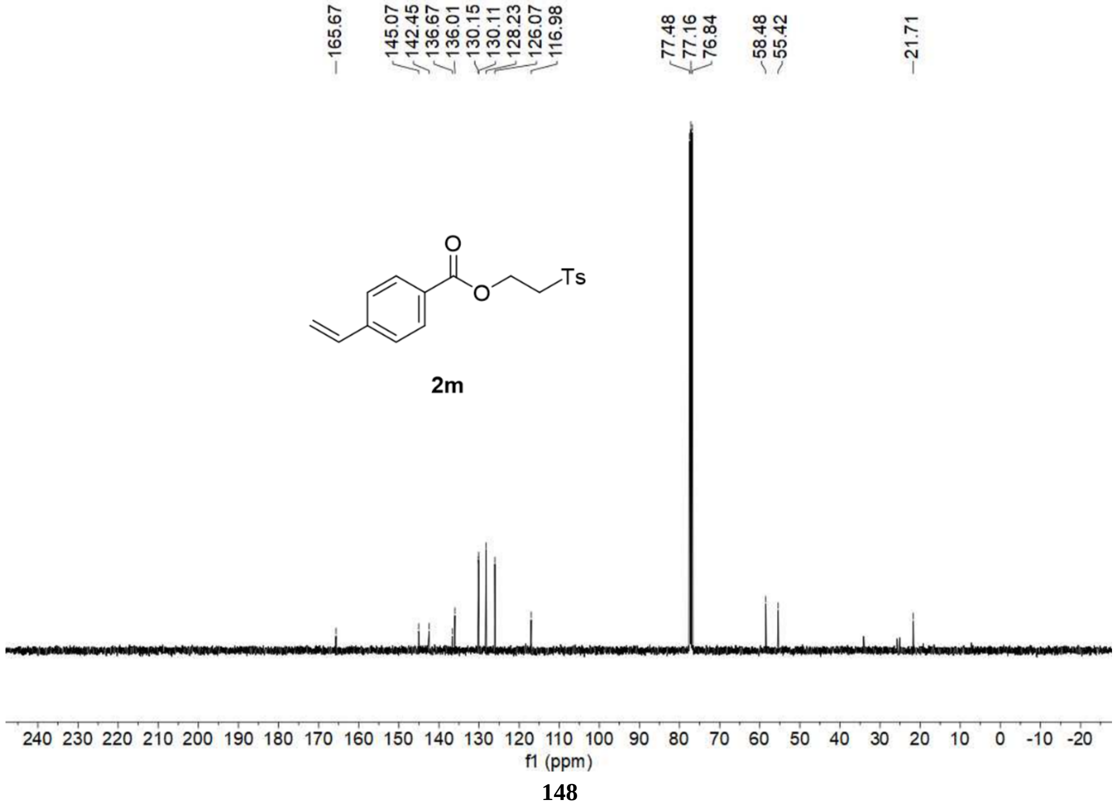
165.67
145.07
142.45
136.67
136.01
130.15
130.11
128.23
126.07
116.98
77.48
77.16
76.84
58.48
55.42
21.71
O
O
Ts
2m
240 230 220 210 200 190 180 170 160 150 140 130 120 110 100 90 80 70 60 50 40 30 20 10 0 -10 -20
f1 (ppm)

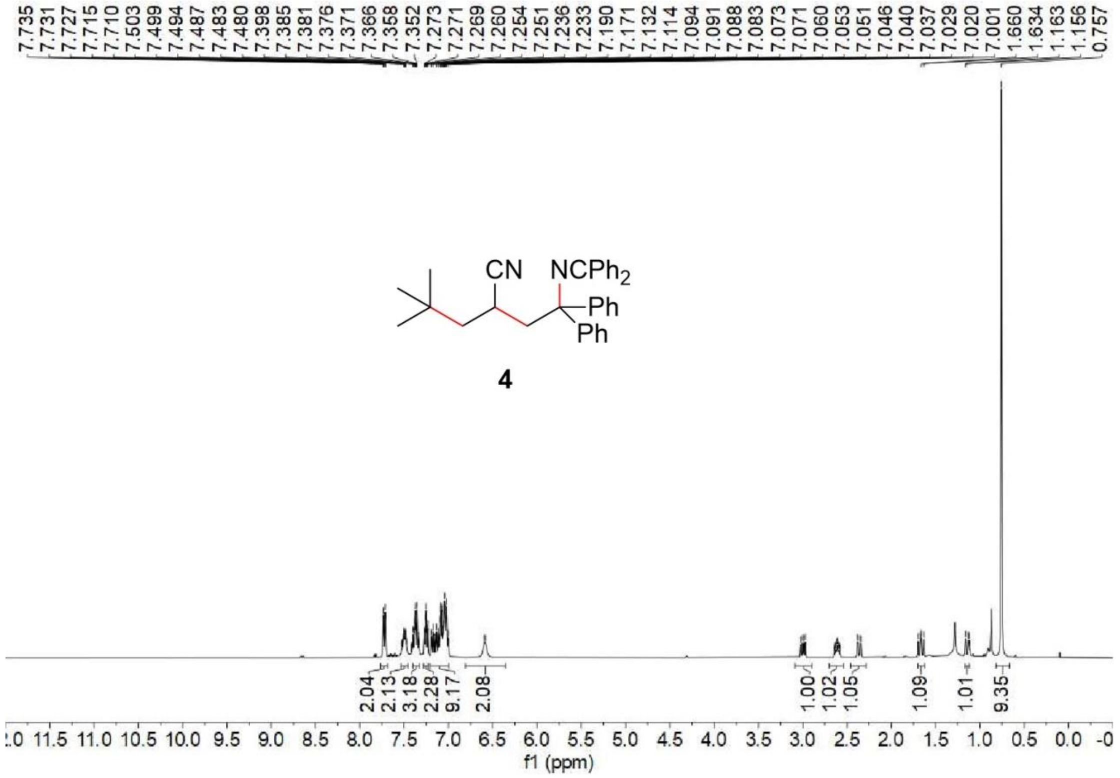
7.735 7.731 7.727 7.715 7.710 7.503 7.499 7.494 7.487 7.483 7.480 7.398 7.385 7.381 7.376 7.371 7.366 7.358 7.352 7.273 7.271 7.269 7.260 7.254 7.251 7.236 7.233 7.190 7.171 7.132 7.114 7.094 7.091 7.088 7.083 7.071 7.060 7.053 7.051 7.046 7.040 7.037 7.029 7.020 7.001 1.660 1.634 1.163 1.156 0.757
CN NCPh2
Ph
Ph
4
2.04 2.13 3.18 2.28 9.17 2.08 1.00 1.02 1.05 1.09 1.01 9.35
f1 (ppm)

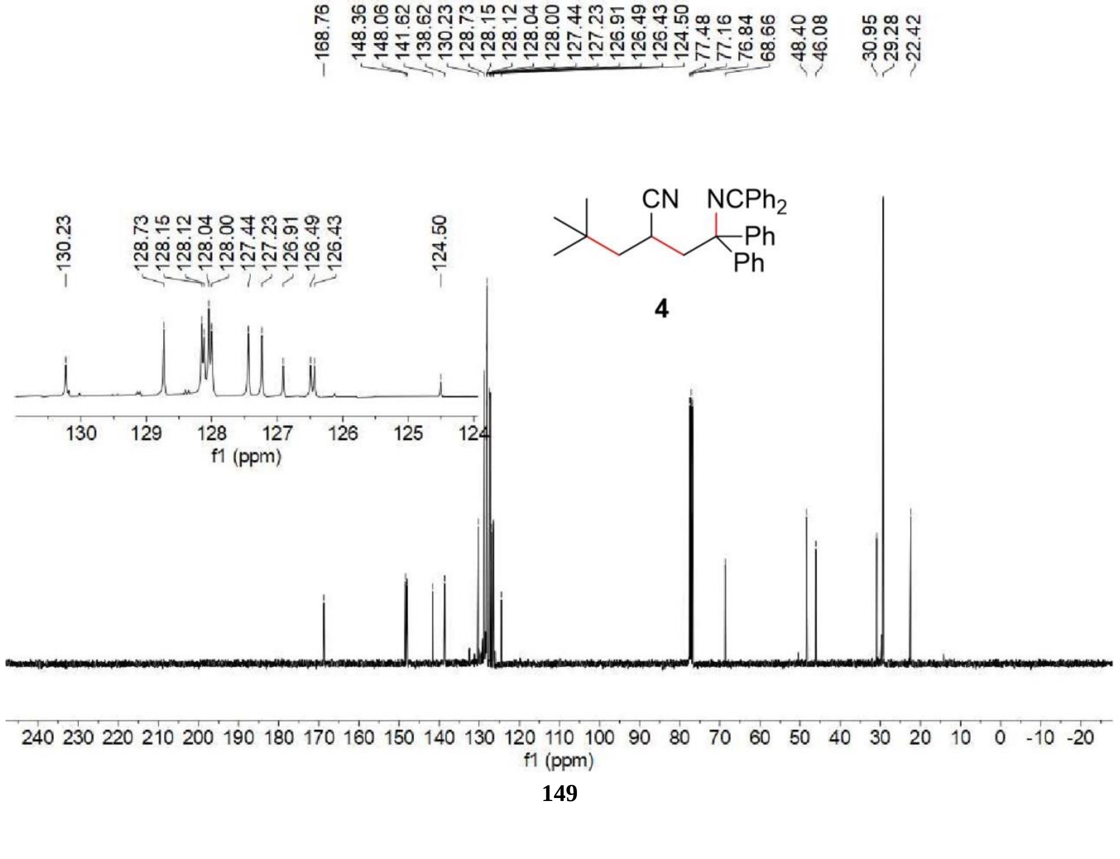
168.76
148.36 148.06 141.62 138.62 130.23 128.73 128.15 128.12 128.04 128.00 127.44 127.23 126.91 126.49 126.43 124.50 77.48 77.16 76.84 68.66
48.40 46.08
30.95 29.28 22.42
130.23 128.73 128.15 128.12 128.04 128.00 127.44 127.23 126.91 126.49 126.43 124.50
CN NCPh2
Ph
Ph
4
f1 (ppm)

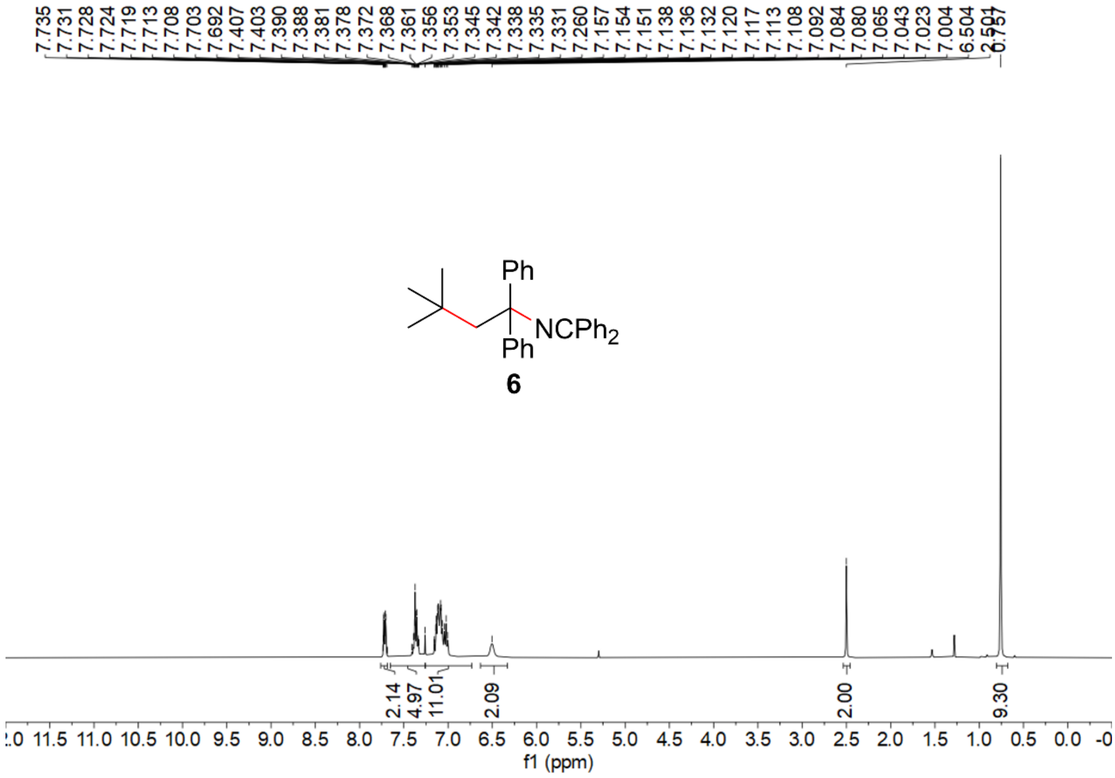

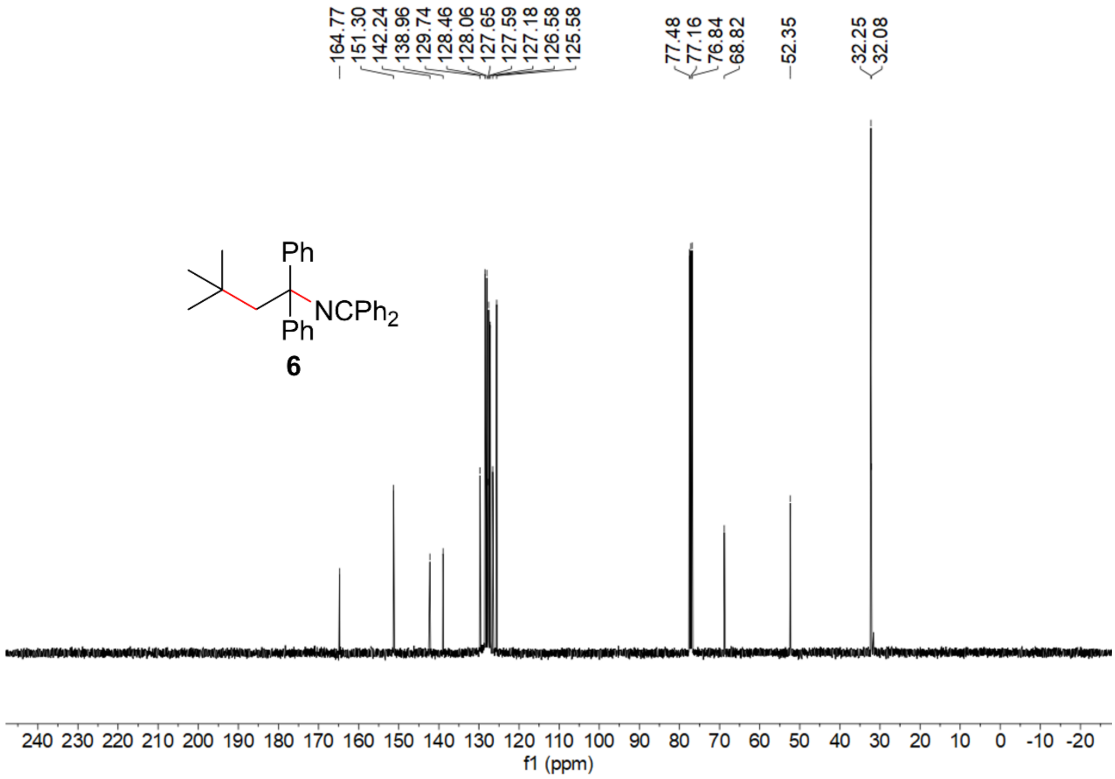

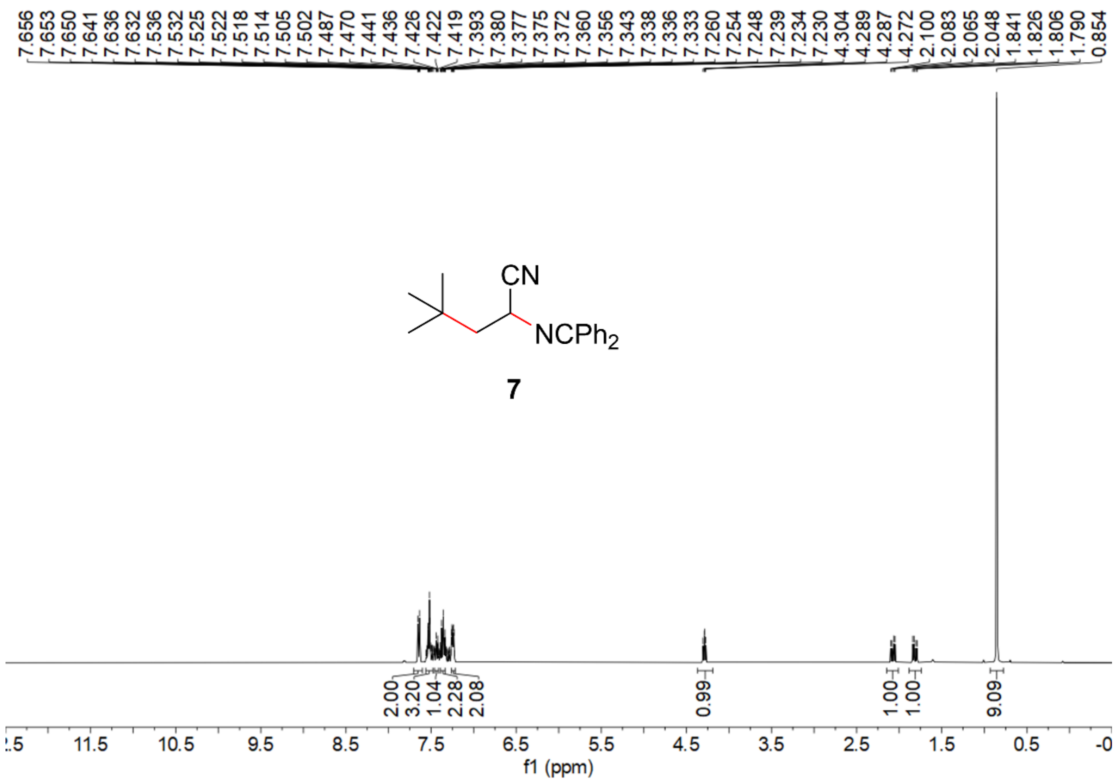
7.656
7.653
7.650
7.641
7.636
7.632
7.536
7.532
7.525
7.522
7.518
7.514
7.505
7.502
7.487
7.470
7.441
7.436
7.426
7.422
7.419
7.393
7.380
7.377
7.375
7.372
7.360
7.356
7.343
7.338
7.336
7.333
7.260
7.254
7.248
7.239
7.234
7.230
4.304
4.289
4.287
4.272
2.100
2.083
2.065
2.048
1.841
1.826
1.806
1.790
0.854
CN
NCPh2
7
2.00
3.20
1.04
2.28
2.08
0.99
1.00
1.00
9.09
11.5
10.5
9.5
8.5
7.5
6.5
5.5
4.5
3.5
2.5
1.5
0.5
f1 (ppm)

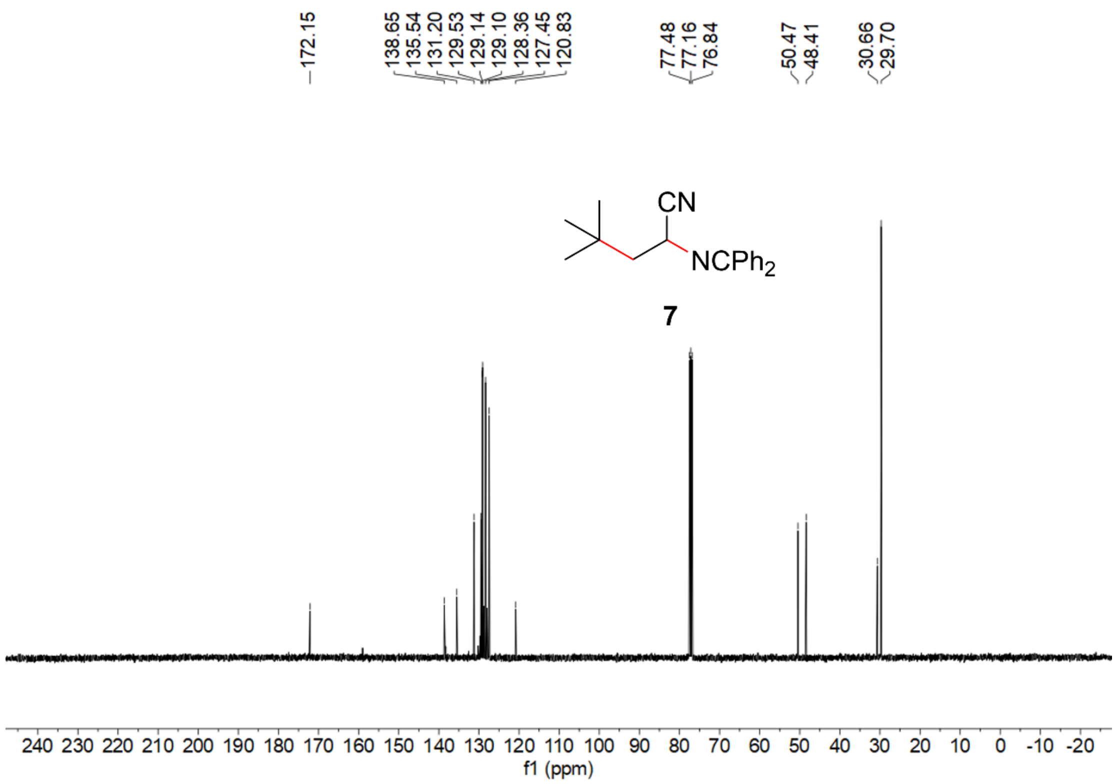
172.15
138.65
135.54
131.20
129.53
129.14
129.10
128.36
127.45
120.83
77.48
77.16
76.84
50.47
48.41
30.66
29.70
CN
NCPh2
7
240 230 220 210 200 190 180 170 160 150 140 130 120 110 100 90 80 70 60 50 40 30 20 10 0 -10 -20
f1 (ppm)

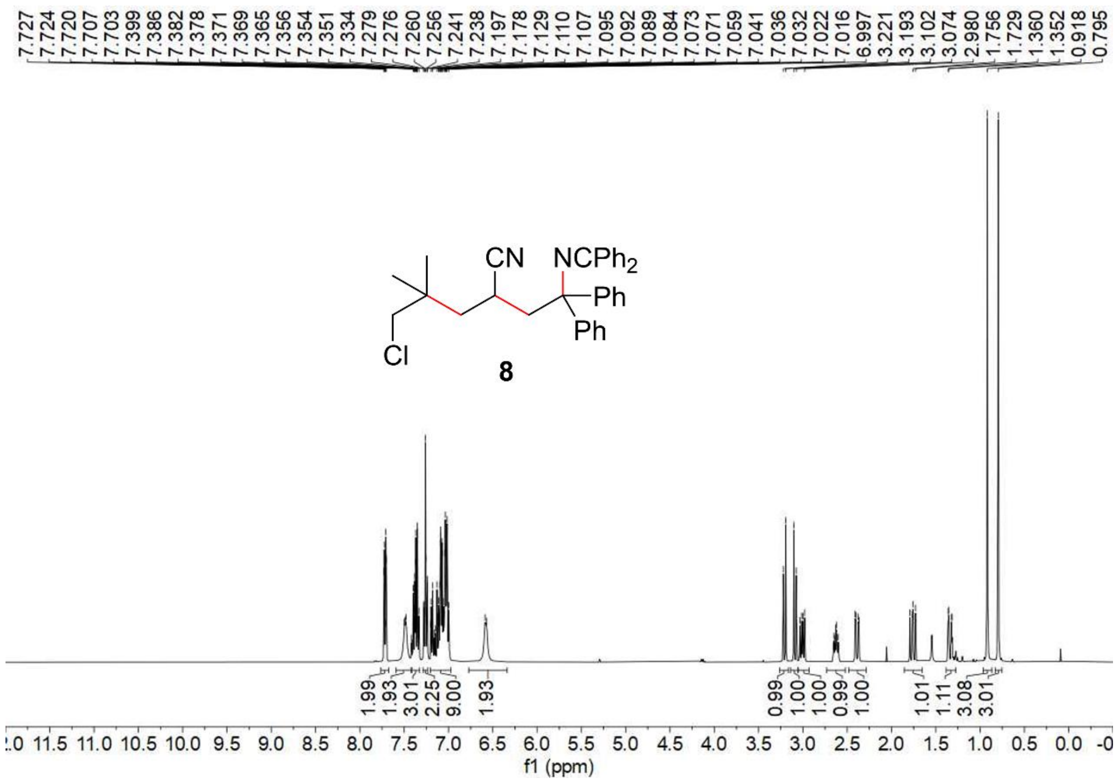
7.727 7.724 7.720 7.707 7.703 7.399 7.386 7.382 7.378 7.371 7.369 7.365 7.356 7.354 7.351 7.334 7.279 7.276 7.260 7.256 7.241 7.238 7.197 7.178 7.129 7.110 7.107 7.095 7.092 7.089 7.084 7.073 7.071 7.059 7.041 7.036 7.032 7.022 7.016 6.997 3.221 3.193 3.102 3.074 2.980 1.756 1.729 1.360 1.352 0.918 0.795
CN
NCPh2
Ph
Ph
Cl
8
1.99 1.93 3.01 2.25 9.00 1.93
0.99 1.00 1.00 0.99 1.00
1.01 1.11 3.08 3.01
f1 (ppm)

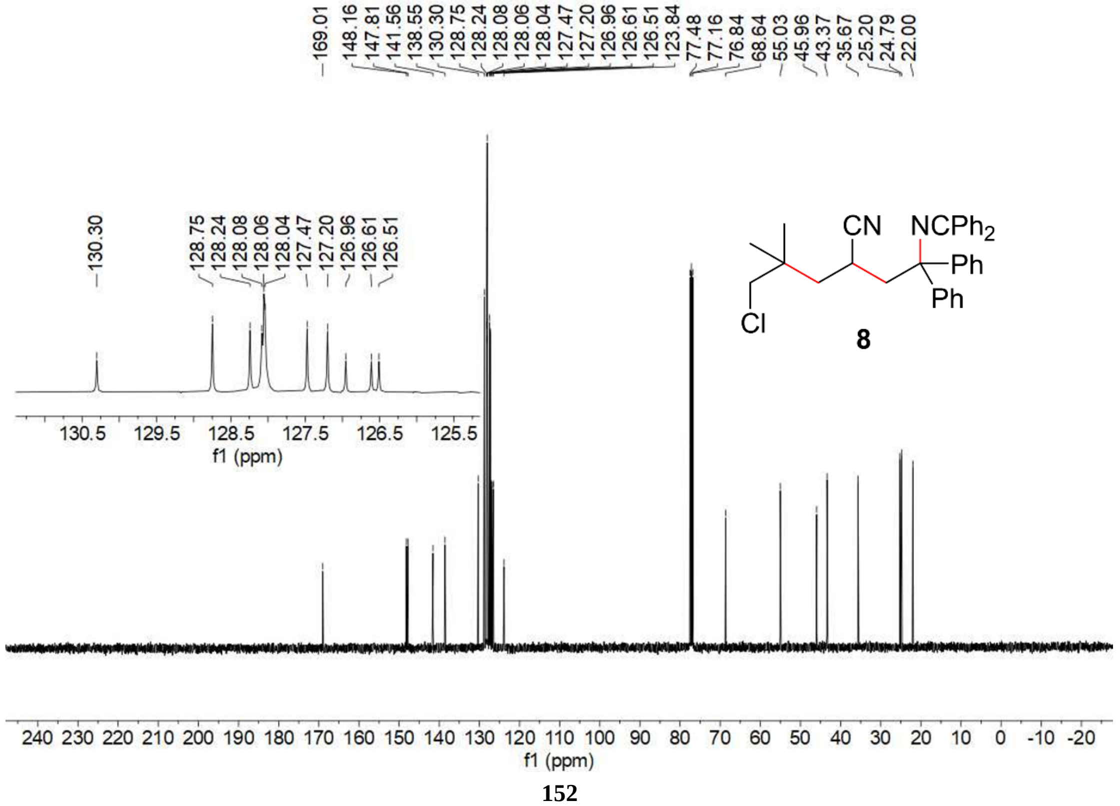
169.01 148.16 147.81 141.56 138.55 130.30 128.75 128.24 128.08 128.06 128.04 127.47 127.20 126.96 126.61 126.51 123.84 77.48 77.16 76.84 68.64 55.03 45.96 43.37 35.67 25.20 24.79 22.00
130.30 128.75 128.24 128.08 128.06 128.04 127.47 127.20 126.96 126.61 126.51
f1 (ppm)
CN
NCPh2
Ph
Ph
Cl
8
f1 (ppm)

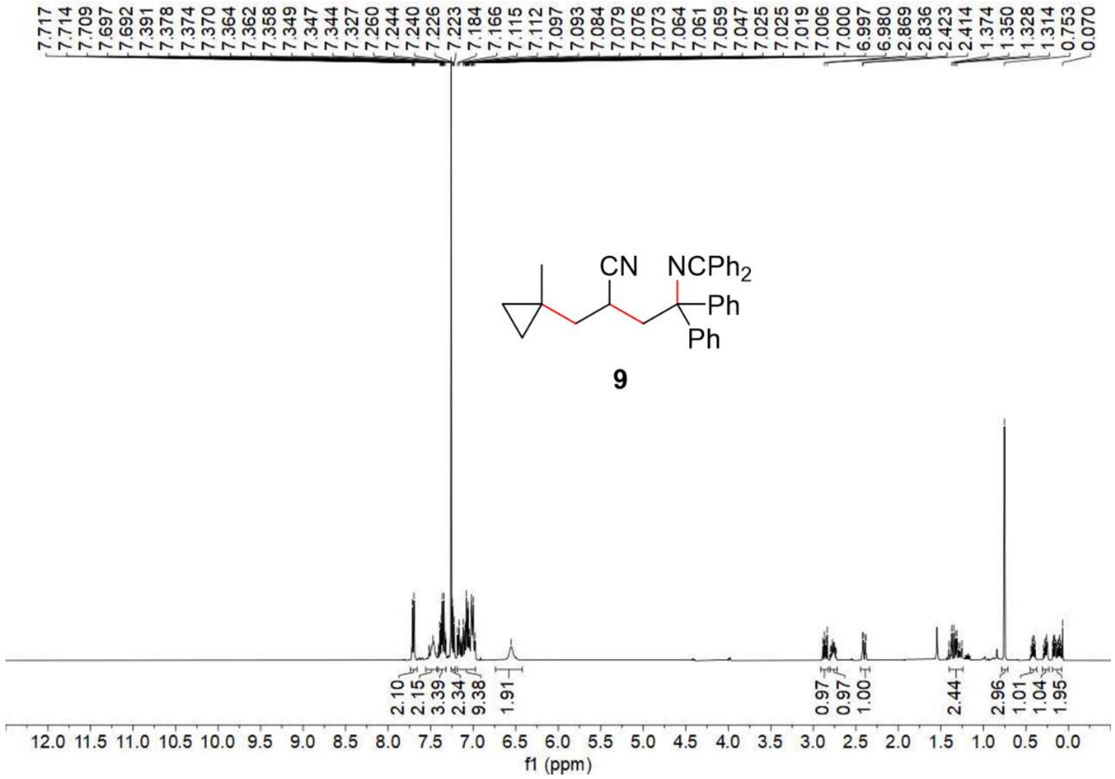
7.717
7.714
7.709
7.697
7.692
7.391
7.378
7.374
7.370
7.364
7.362
7.358
7.349
7.347
7.344
7.327
7.260
7.244
7.240
7.226
7.223
7.184
7.166
7.115
7.112
7.097
7.093
7.084
7.079
7.076
7.073
7.064
7.061
7.059
7.047
7.025
7.025
7.019
7.006
7.000
6.997
6.980
2.869
2.836
2.423
2.414
1.374
1.350
1.328
1.314
0.753
0.070
CN
NCPh2
Ph
Ph
9
2.10
2.15
3.39
2.34
9.38
1.91
0.97
0.97
1.00
2.44
2.96
1.01
1.04
1.95
12.0 11.5 11.0 10.5 10.0 9.5 9.0 8.5 8.0 7.5 7.0 6.5 6.0 5.5 5.0 4.5 4.0 3.5 3.0 2.5 2.0 1.5 1.0 0.5 0.0
f1 (ppm)

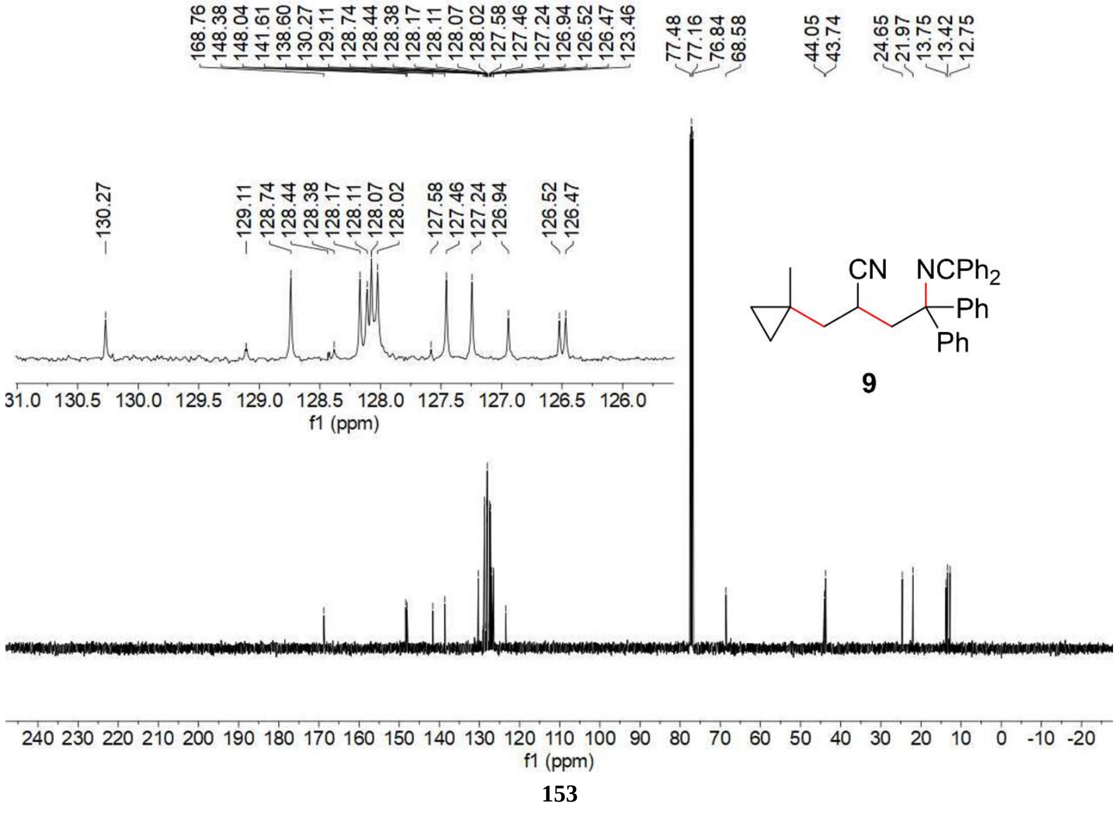
168.76
148.38
148.04
141.61
138.60
130.27
129.11
128.74
128.44
128.38
128.17
128.11
128.07
128.02
127.58
127.46
127.24
126.94
126.52
126.47
123.46
77.48
77.16
76.84
68.58
44.05
43.74
24.65
21.97
13.75
13.42
12.75
130.27
129.11
128.74
128.44
128.38
128.17
128.11
128.07
128.02
127.58
127.46
127.24
126.94
126.52
126.47
31.0 130.5 130.0 129.5 129.0 128.5 128.0 127.5 127.0 126.5 126.0
f1 (ppm)
CN
NCPh2
Ph
Ph
9
240 230 220 210 200 190 180 170 160 150 140 130 120 110 100 90 80 70 60 50 40 30 20 10 0 -10 -20
f1 (ppm)

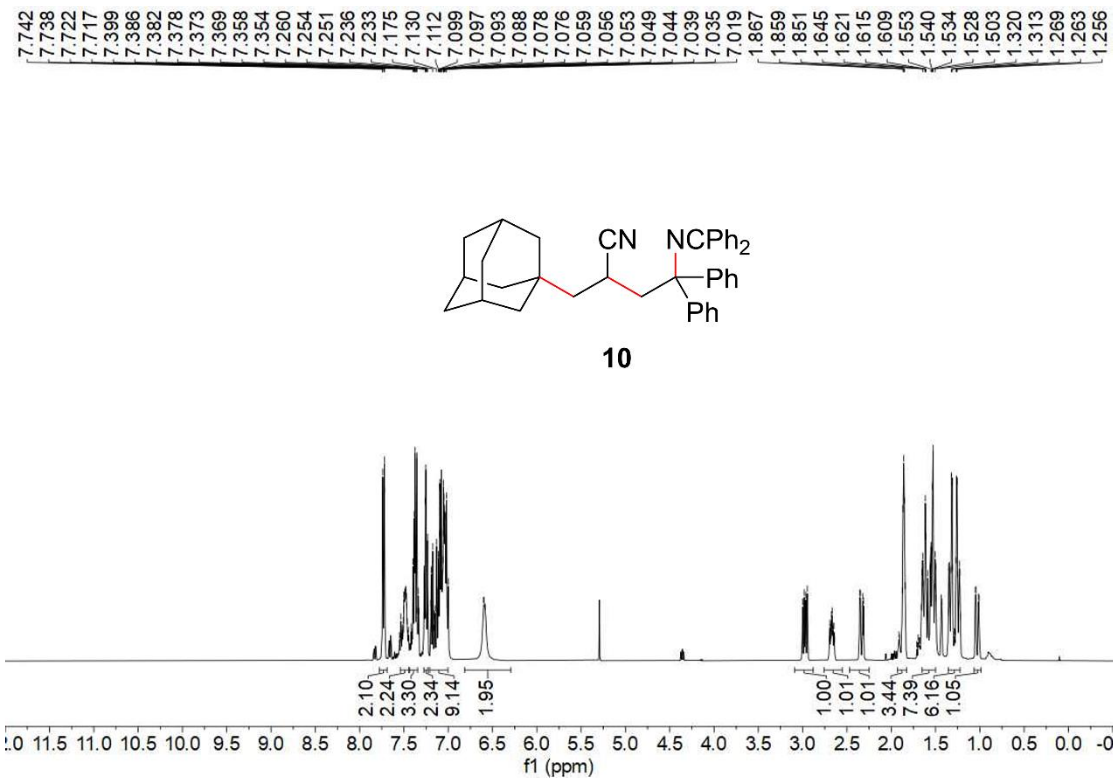

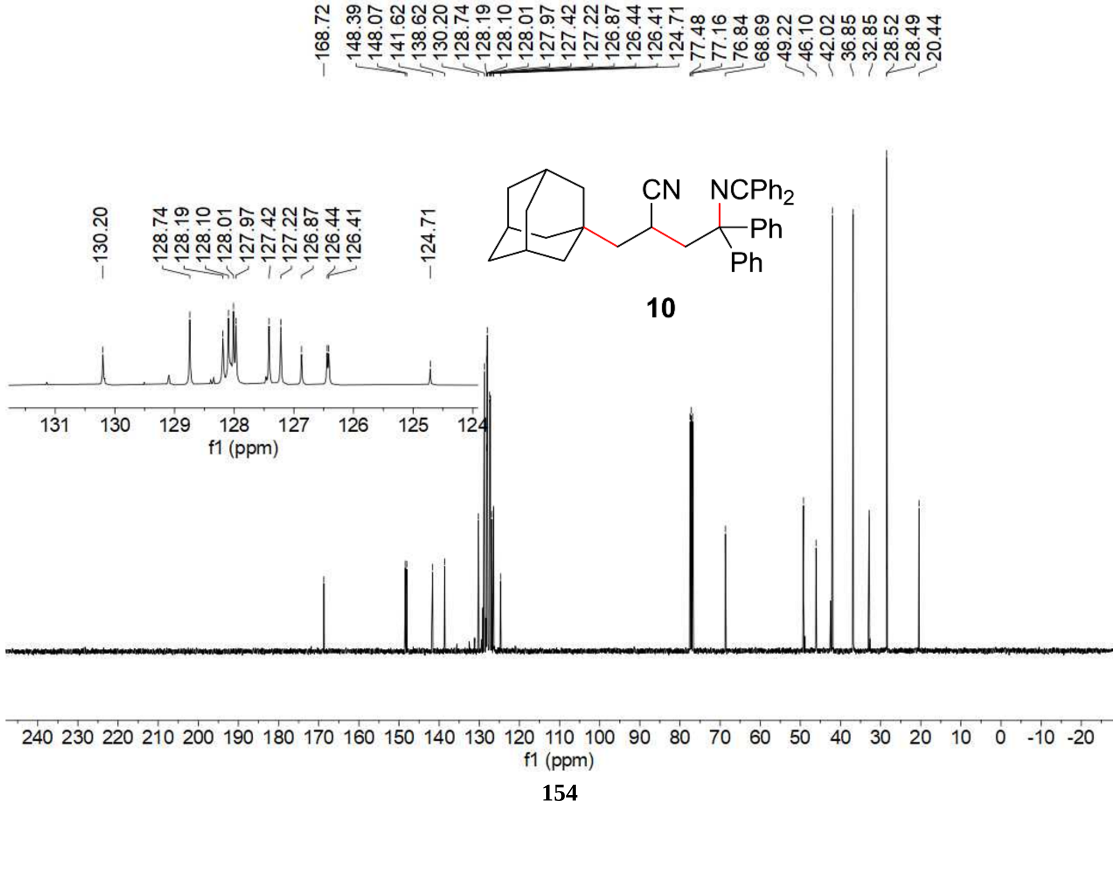

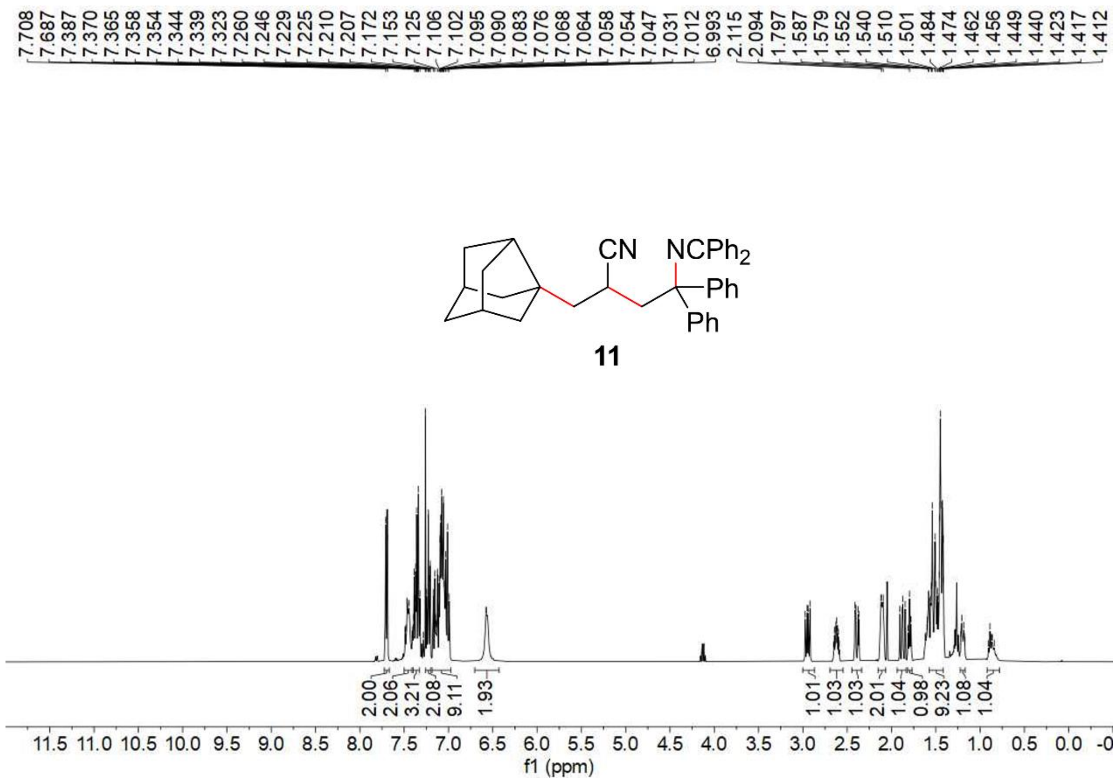
7.708 7.687 7.387 7.370 7.365 7.358 7.354 7.344 7.339 7.323 7.260 7.246 7.229 7.225 7.210 7.207 7.172 7.153 7.125 7.106 7.102 7.095 7.090 7.083 7.076 7.068 7.064 7.058 7.054 7.047 7.031 7.012 6.993 2.115 2.094 1.797 1.587 1.579 1.552 1.540 1.510 1.501 1.484 1.474 1.462 1.456 1.449 1.440 1.423 1.417 1.412
CN
NCPh$_2$
Ph
Ph
11
2.00 2.06 3.21 2.08 9.11 1.93 1.01 1.03 1.03 2.01 1.04 0.98 9.23 1.08 1.04
f1 (ppm)

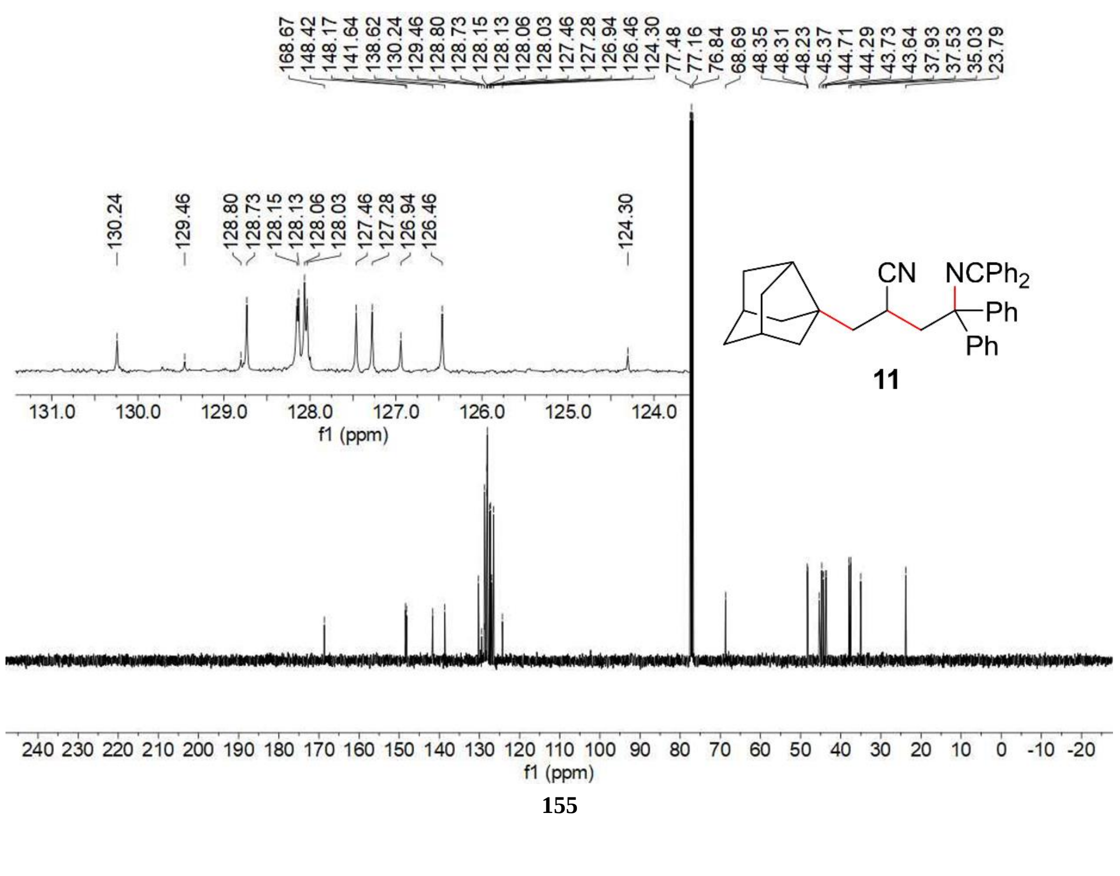
168.67 148.42 148.17 141.64 138.62 130.24 129.46 128.80 128.73 128.15 128.13 128.06 128.03 127.46 127.28 126.94 126.46 124.30 77.48 77.16 76.84 68.69 48.35 48.31 48.23 45.37 44.71 44.29 43.73 43.64 37.93 37.53 35.03 23.79
130.24 129.46 128.80 128.73 128.15 128.13 128.06 128.03 127.46 127.28 126.94 126.46 124.30
f1 (ppm)
CN
NCPh$_2$
Ph
Ph
11
f1 (ppm)

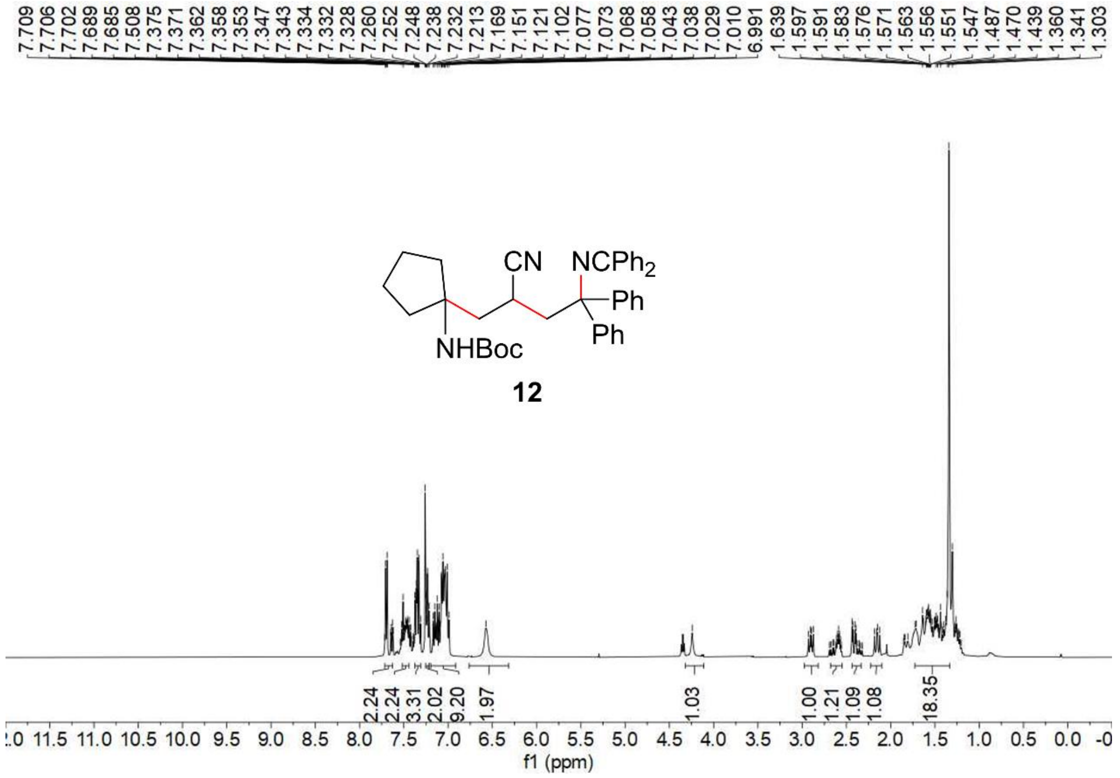
7.709 7.706 7.702 7.689 7.685 7.508 7.375 7.371 7.362 7.358 7.353 7.347 7.343 7.334 7.332 7.328 7.260 7.252 7.248 7.238 7.232 7.213 7.169 7.151 7.121 7.102 7.077 7.073 7.068 7.058 7.043 7.038 7.029 7.010 6.991 1.639 1.597 1.591 1.583 1.576 1.571 1.563 1.556 1.551 1.547 1.487 1.470 1.439 1.360 1.341 1.303
CN
NCPh2
Ph
Ph
NHBoc
12
2.24 2.24 3.31 2.02 9.20 1.97 1.03 1.00 1.21 1.09 1.08 18.35
f1 (ppm)

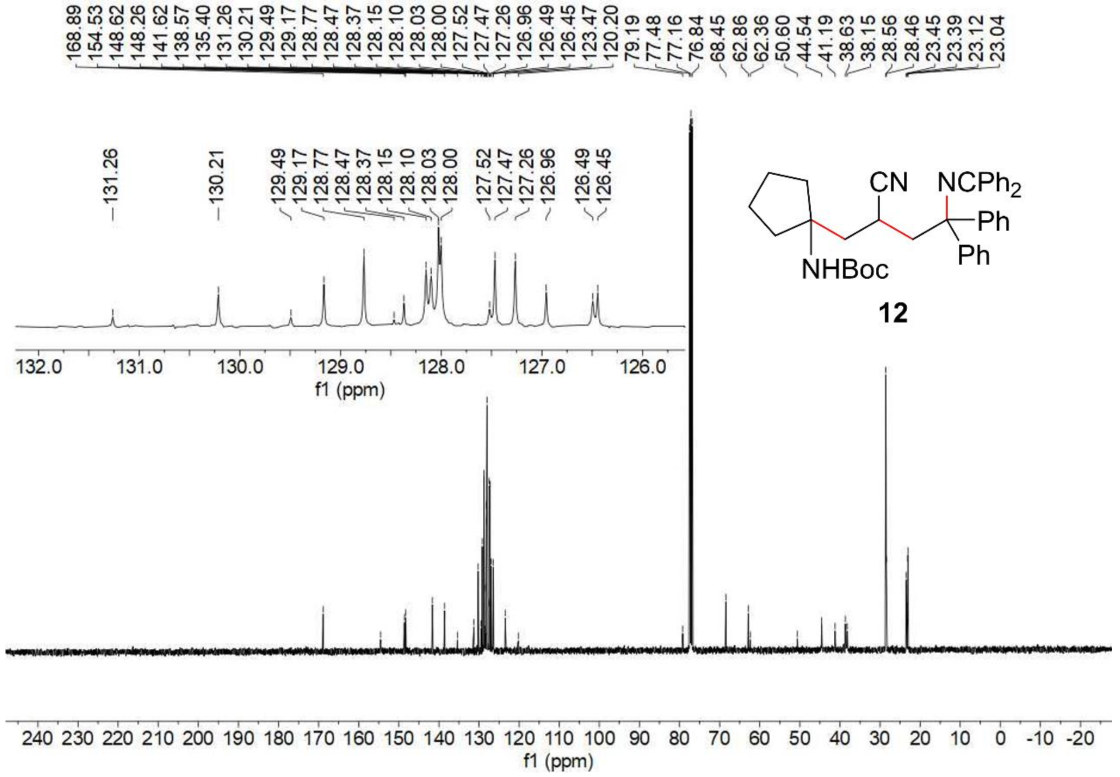
168.89 154.53 148.62 148.26 141.62 138.57 135.40 131.26 130.21 129.49 129.17 128.77 128.47 128.37 128.15 128.10 128.03 128.00 127.52 127.47 127.26 126.96 126.49 126.45 123.47 120.20 79.19 77.48 77.16 76.84 68.45 62.86 62.36 50.60 44.54 41.19 38.63 38.15 28.56 28.46 23.45 23.39 23.12 23.04
131.26 130.21 129.49 129.17 128.77 128.47 128.37 128.15 128.10 128.03 128.00 127.52 127.47 127.26 126.96 126.49 126.45
f1 (ppm)
CN
NCPh2
Ph
Ph
NHBoc
12
f1 (ppm)

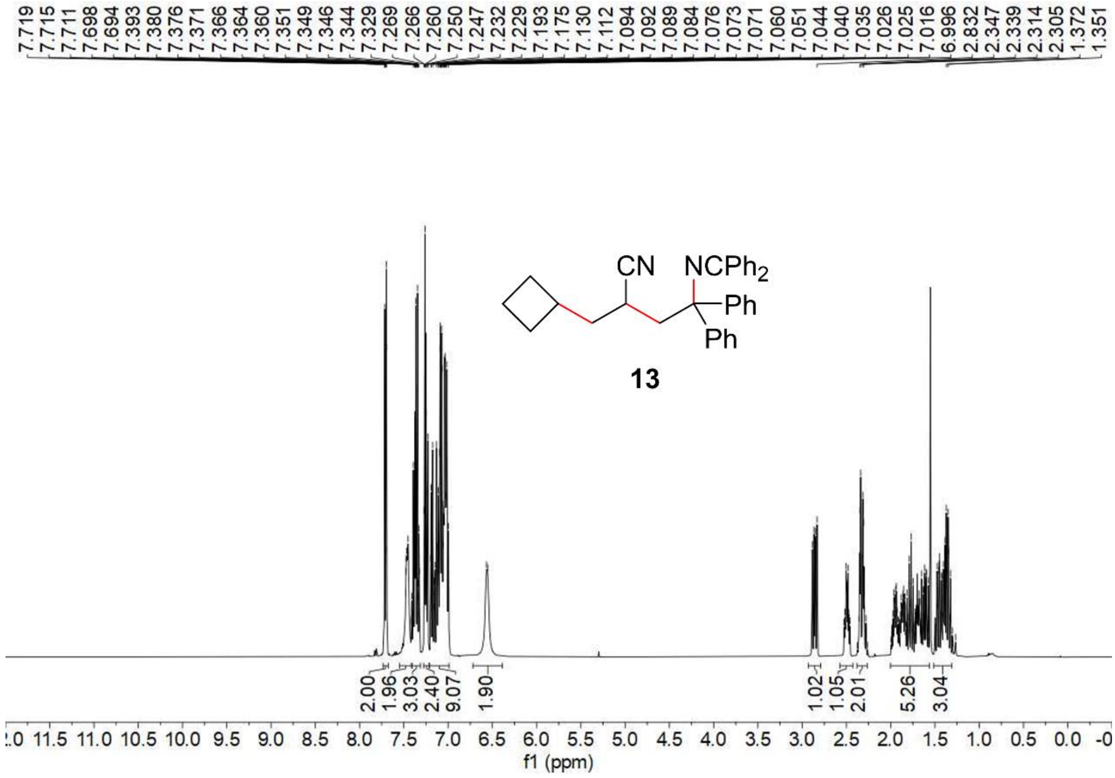
7.719 7.715 7.711 7.698 7.694 7.393 7.380 7.376 7.371 7.366 7.364 7.360 7.351 7.349 7.346 7.344 7.329 7.269 7.266 7.260 7.250 7.247 7.232 7.229 7.193 7.175 7.130 7.112 7.094 7.092 7.089 7.084 7.076 7.073 7.071 7.060 7.051 7.044 7.040 7.035 7.026 7.025 7.016 6.996 2.832 2.347 2.339 2.314 2.305 1.372 1.351
CN
NCPh2
Ph
Ph
13
2.00 1.96 3.03 2.40 9.07 1.90 1.02 1.05 2.01 5.26 3.04
f1 (ppm)

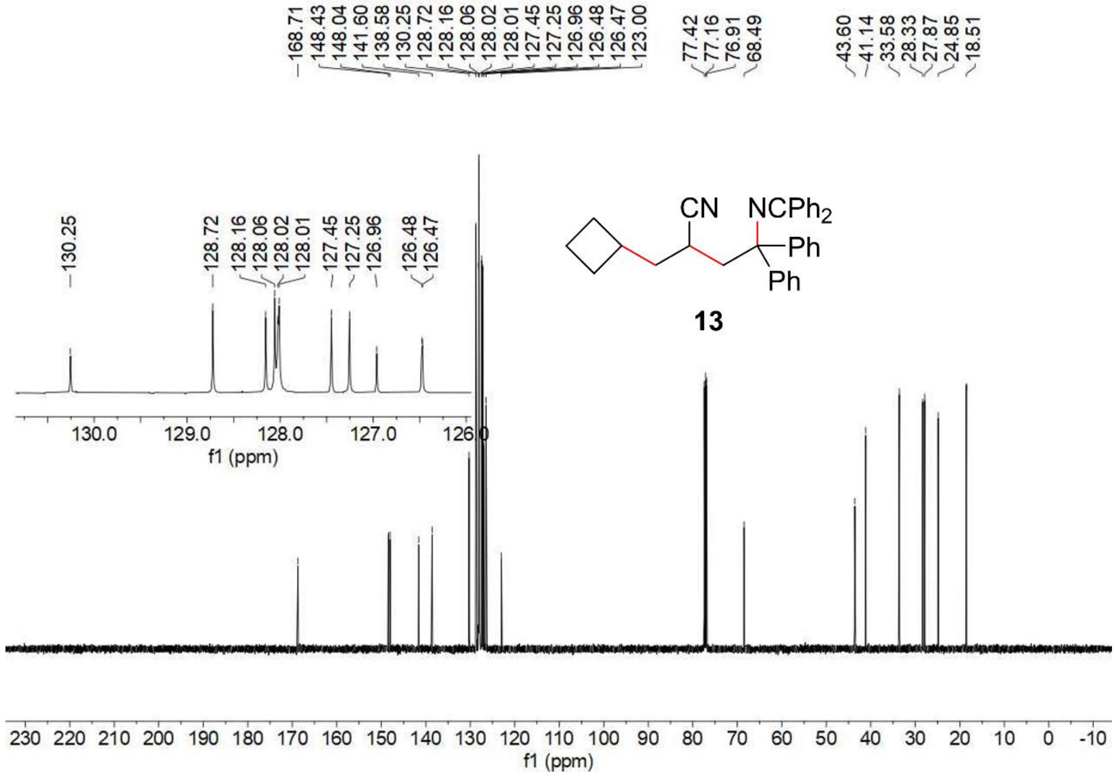
168.71 148.43 148.04 141.60 138.58 130.25 128.72 128.16 128.06 128.02 128.01 127.45 127.25 126.96 126.48 126.47 123.00
77.42 77.16 76.91 68.49
43.60 41.14 33.58 28.33 27.87 24.85 18.51
130.25 128.72 128.16 128.06 128.02 128.01 127.45 127.25 126.96 126.48 126.47
f1 (ppm)
CN
NCPh2
Ph
Ph
13
f1 (ppm)

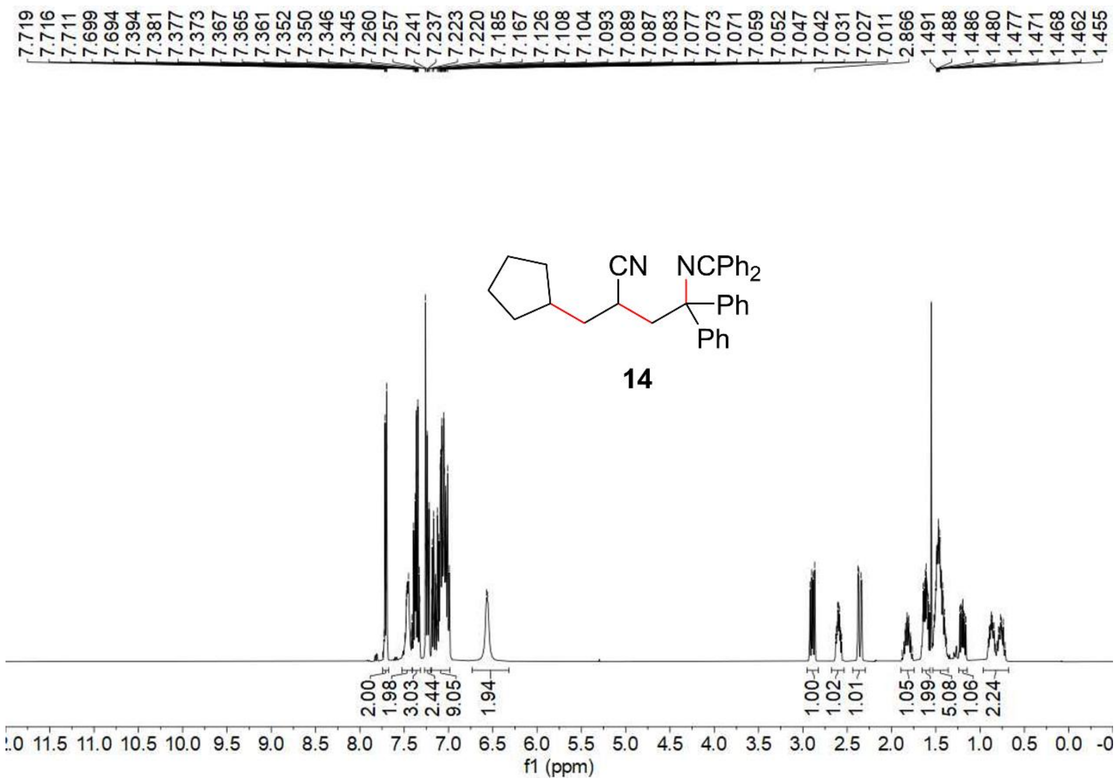

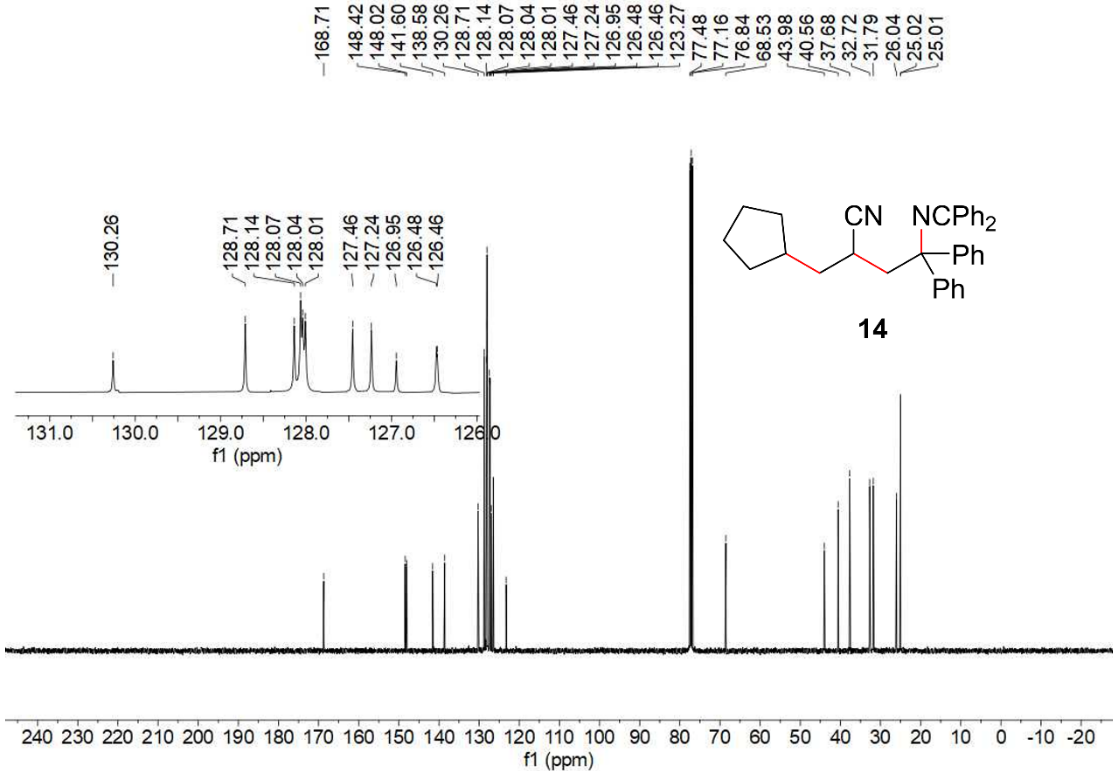

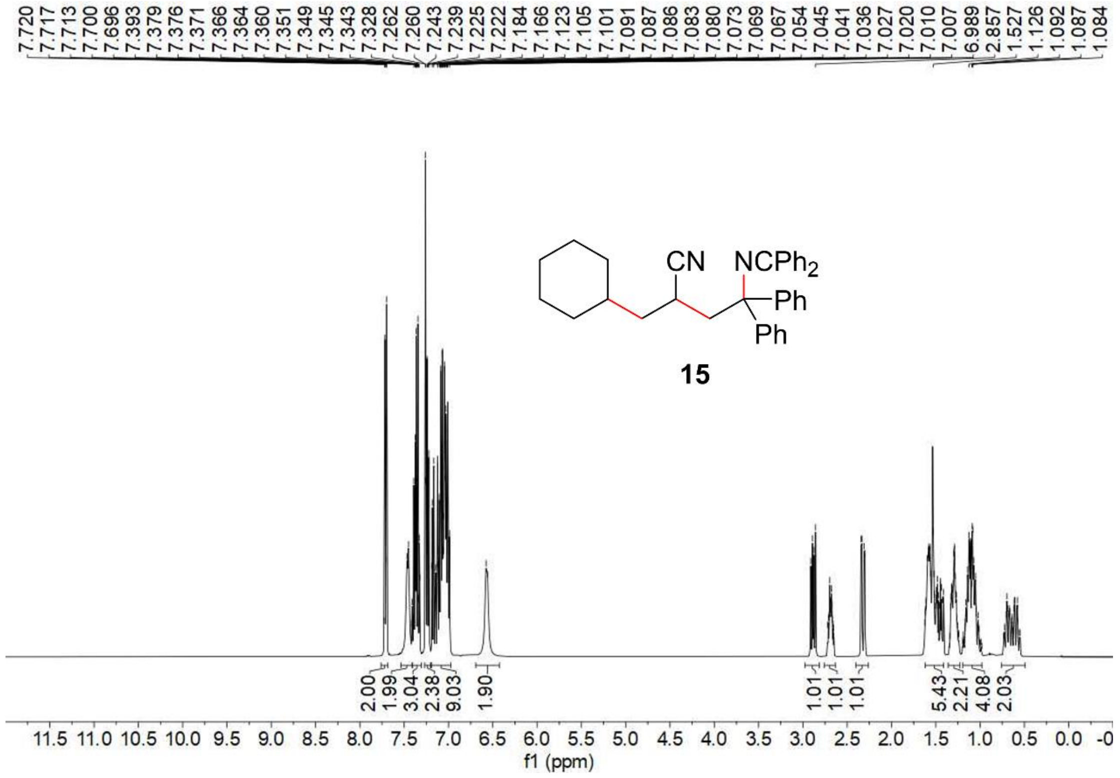

7.720
7.717
7.713
7.700
7.696
7.393
7.379
7.376
7.371
7.366
7.364
7.360
7.351
7.349
7.345
7.343
7.328
7.262
7.260
7.243
7.239
7.225
7.222
7.184
7.166
7.123
7.105
7.101
7.091
7.087
7.086
7.083
7.080
7.073
7.069
7.067
7.054
7.045
7.041
7.036
7.027
7.020
7.010
7.007
6.989
2.857
1.527
1.126
1.092
1.087
1.084
CN
NCPh2
Ph
Ph
15
2.00
1.99
3.04
2.38
9.03
1.90
1.01
1.01
1.01
5.43
2.21
4.08
2.03
f1 (ppm)

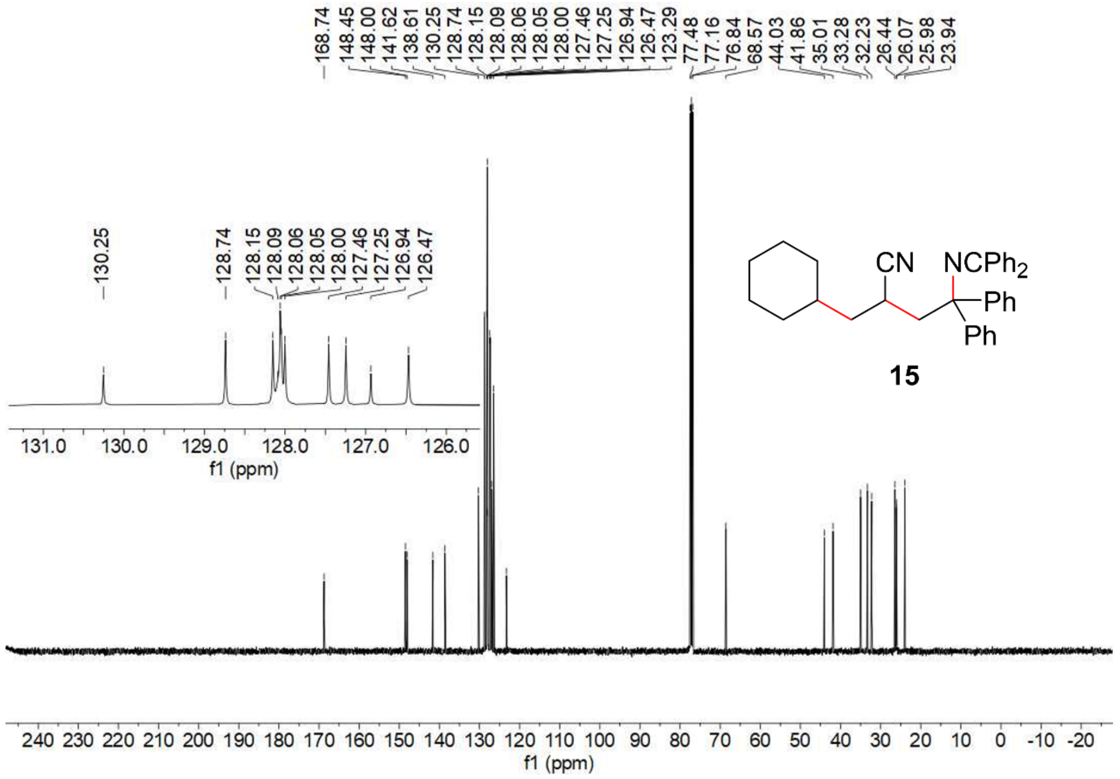

168.74
148.45
148.00
141.62
138.61
130.25
128.74
128.15
128.09
128.06
128.05
128.00
127.46
127.25
126.94
126.47
123.29
77.48
77.16
76.84
68.57
44.03
41.86
35.01
33.28
32.23
26.44
26.07
25.98
23.94
CN
NCPh2
Ph
Ph
15
f1 (ppm)
f1 (ppm)

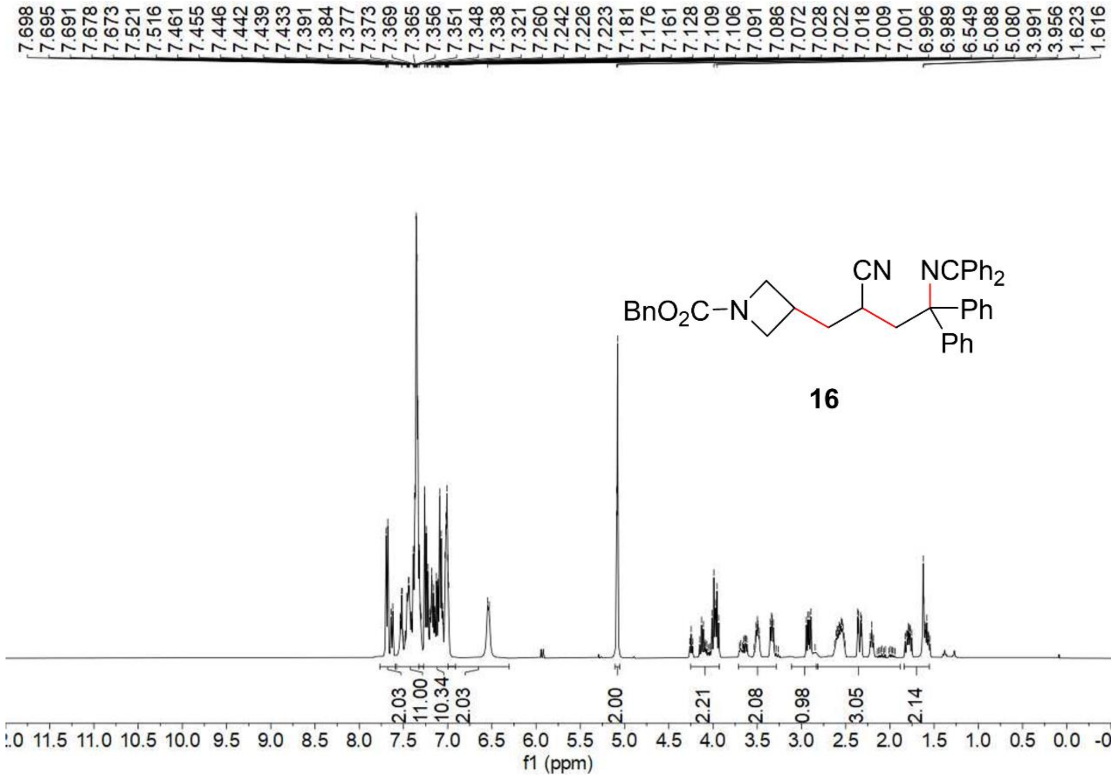
BnO2C–N CN NCPh2 Ph Ph
16
f1 (ppm)

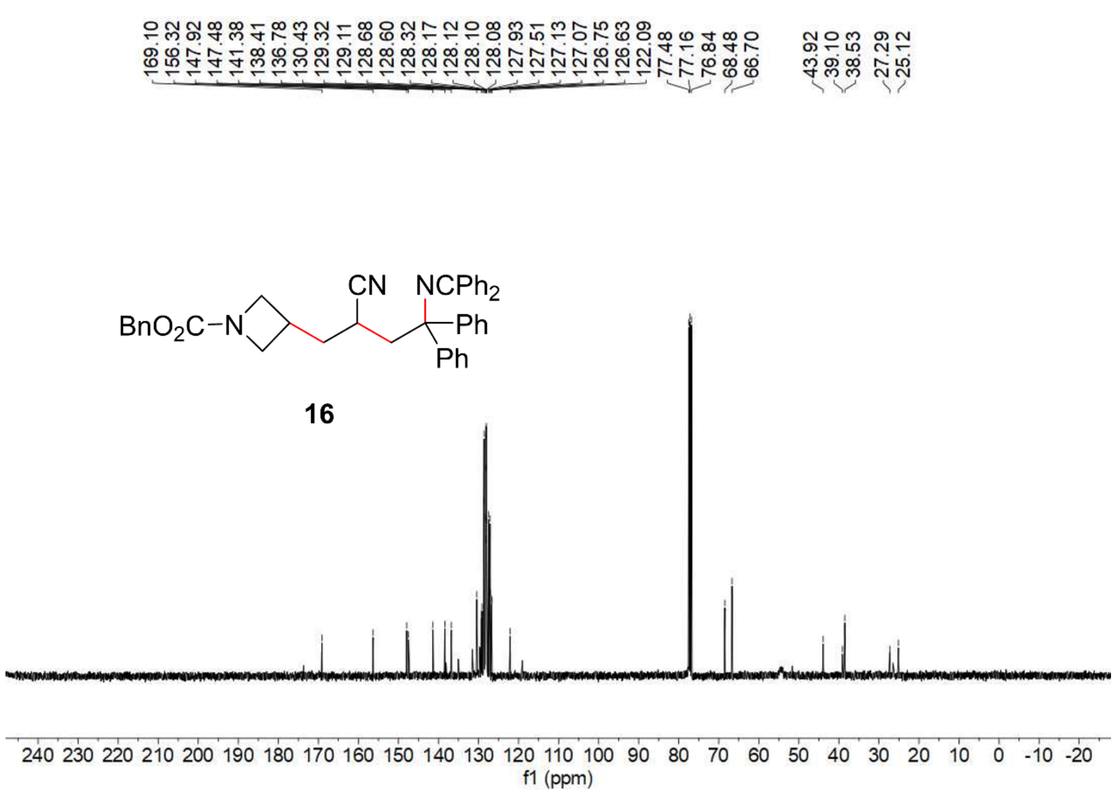
BnO2C–N CN NCPh2 Ph Ph
16
f1 (ppm)

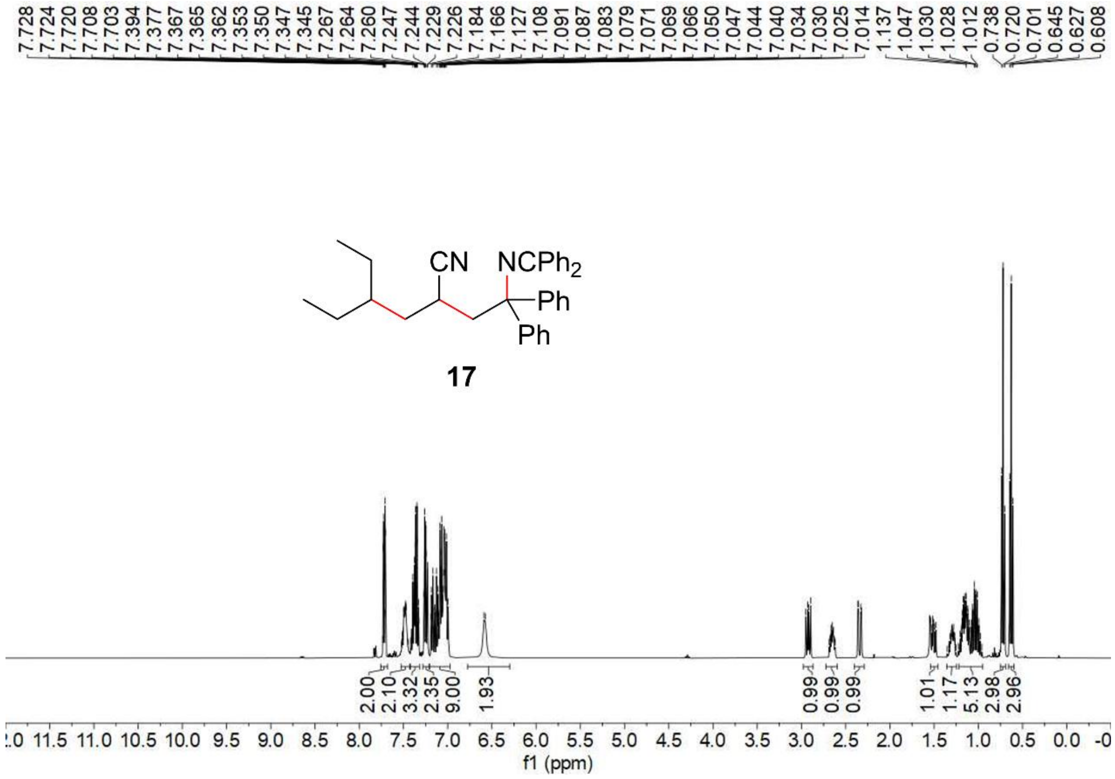
7.728 7.724 7.720 7.708 7.703 7.394 7.377 7.367 7.365 7.362 7.353 7.350 7.347 7.345 7.267 7.264 7.260 7.247 7.244 7.229 7.226 7.184 7.166 7.127 7.108 7.091 7.087 7.083 7.079 7.071 7.069 7.066 7.050 7.047 7.044 7.040 7.034 7.030 7.025 7.014 1.137 1.047 1.030 1.028 1.012 0.738 0.720 0.701 0.645 0.627 0.608
CN
NCPh2
Ph
Ph
17
2.00 2.10 3.32 2.35 9.00 1.93 0.99 0.99 0.99 1.01 1.17 5.13 2.98 2.96
f1 (ppm)

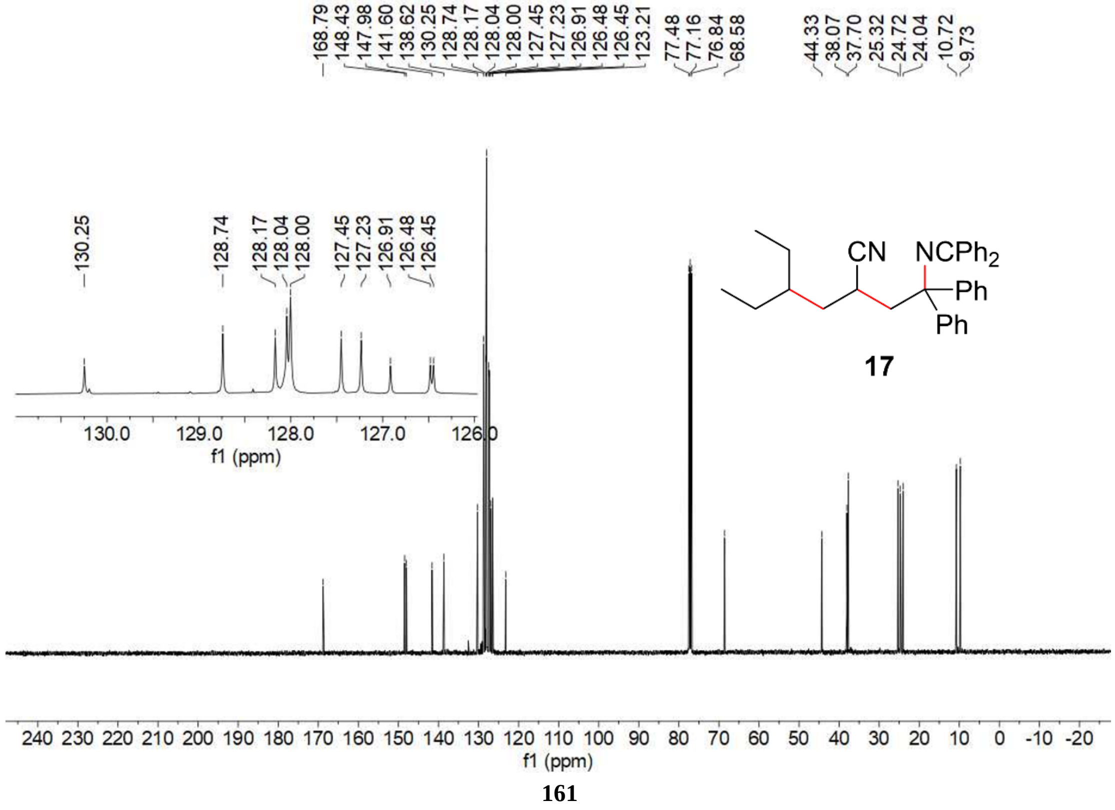
168.79 148.43 147.98 141.60 138.62 130.25 128.74 128.17 128.04 128.00 127.45 127.23 126.91 126.48 126.45 123.21 77.48 77.16 76.84 68.58 44.33 38.07 37.70 25.32 24.72 24.04 10.72 9.73
130.25 128.74 128.17 128.04 128.00 127.45 127.23 126.91 126.48 126.45
f1 (ppm)
CN
NCPh2
Ph
Ph
17
f1 (ppm)

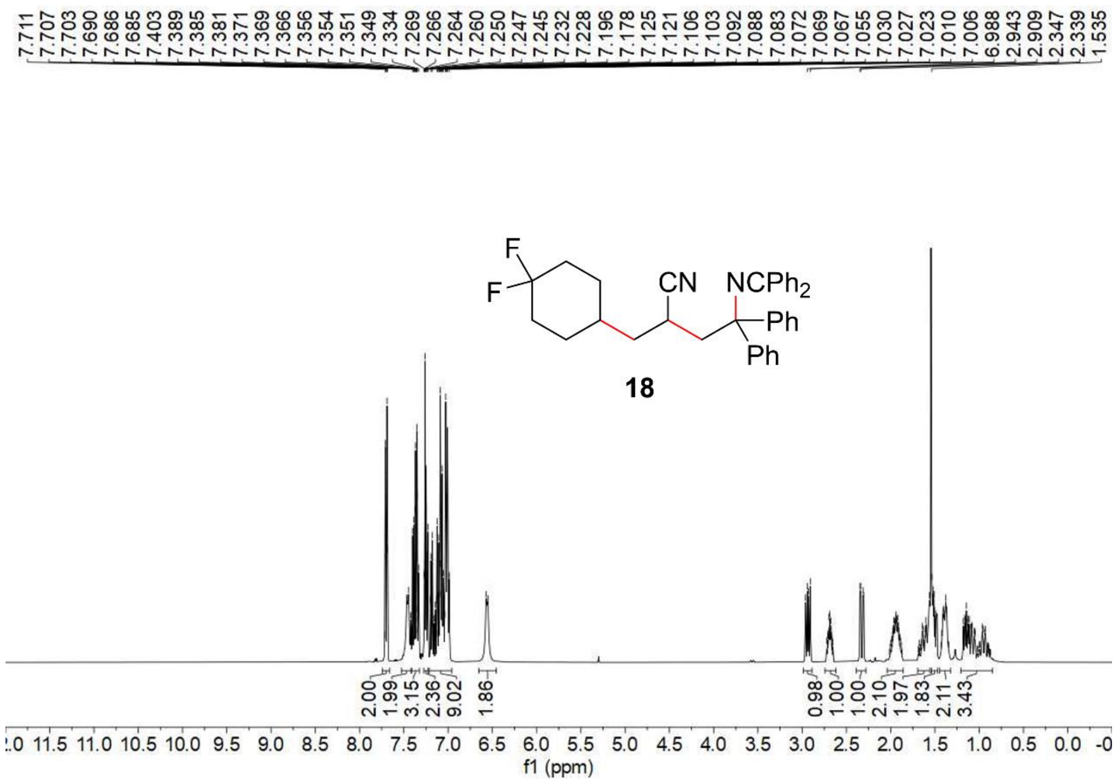
7.711 7.707 7.703 7.690 7.686 7.685 7.403 7.389 7.385 7.381 7.371 7.369 7.366 7.356 7.354 7.351 7.349 7.334 7.269 7.266 7.264 7.260 7.250 7.247 7.245 7.232 7.228 7.196 7.178 7.125 7.121 7.106 7.103 7.092 7.088 7.083 7.072 7.069 7.067 7.055 7.030 7.027 7.023 7.010 7.006 6.988 2.943 2.909 2.347 2.339 1.535
F
F
CN
NCPh2
Ph
Ph
18
2.00 1.99 3.15 2.36 9.02 1.86
0.98 1.00 1.00 2.10 1.97 1.83 2.11 3.43
f1 (ppm)

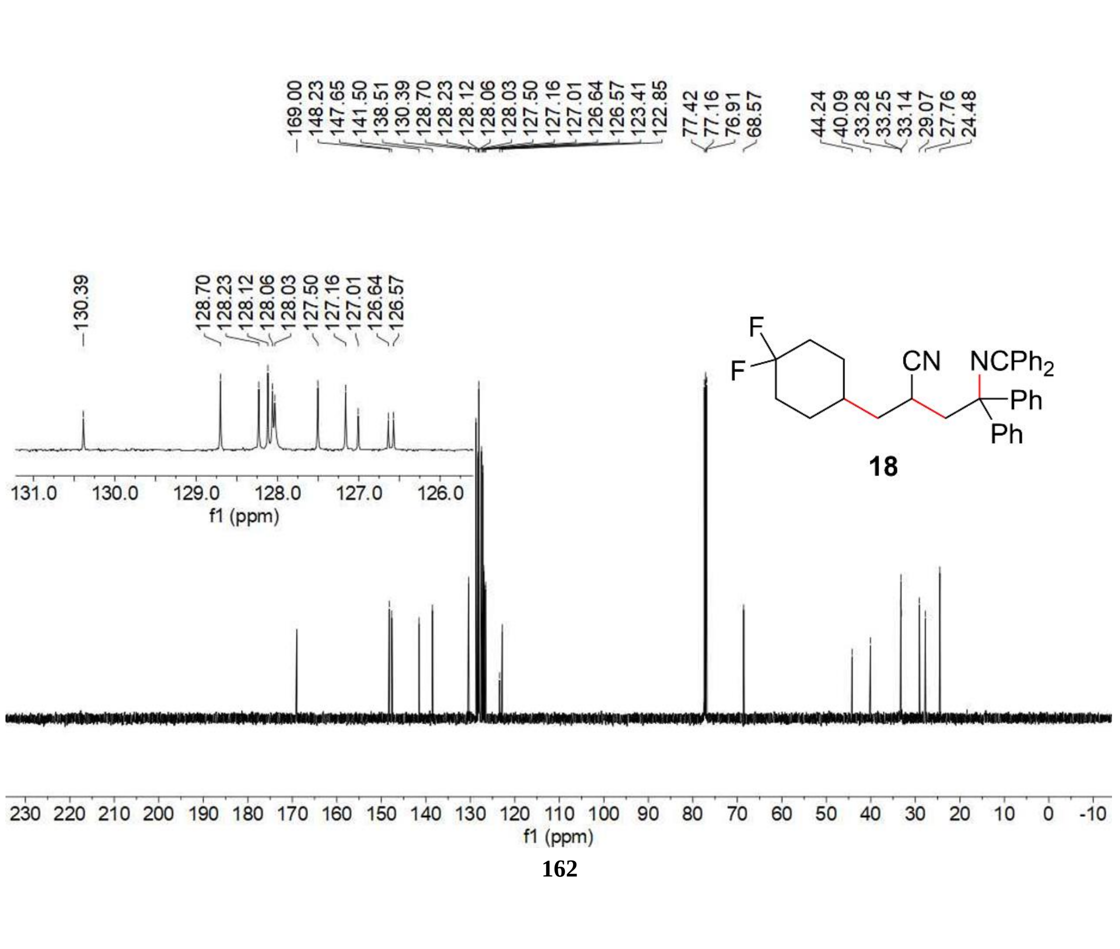
169.00 148.23 147.65 141.50 138.51 130.39 128.70 128.23 128.12 128.06 128.03 127.50 127.16 127.01 126.64 126.57 123.41 122.85
77.42 77.16 76.91 68.57
44.24 40.09 33.28 33.25 33.14 29.07 27.76 24.48
130.39
128.70 128.23 128.12 128.06 128.03 127.50 127.16 127.01 126.64 126.57
f1 (ppm)
F
F
CN
NCPh2
Ph
Ph
18
f1 (ppm)

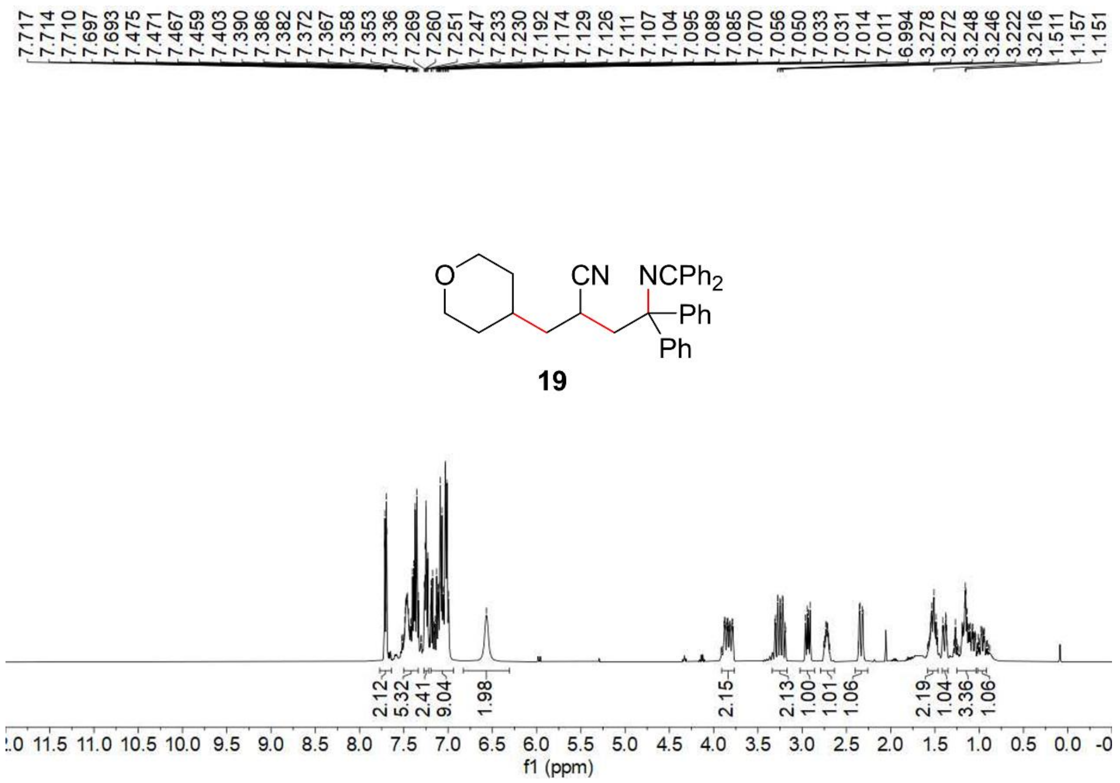

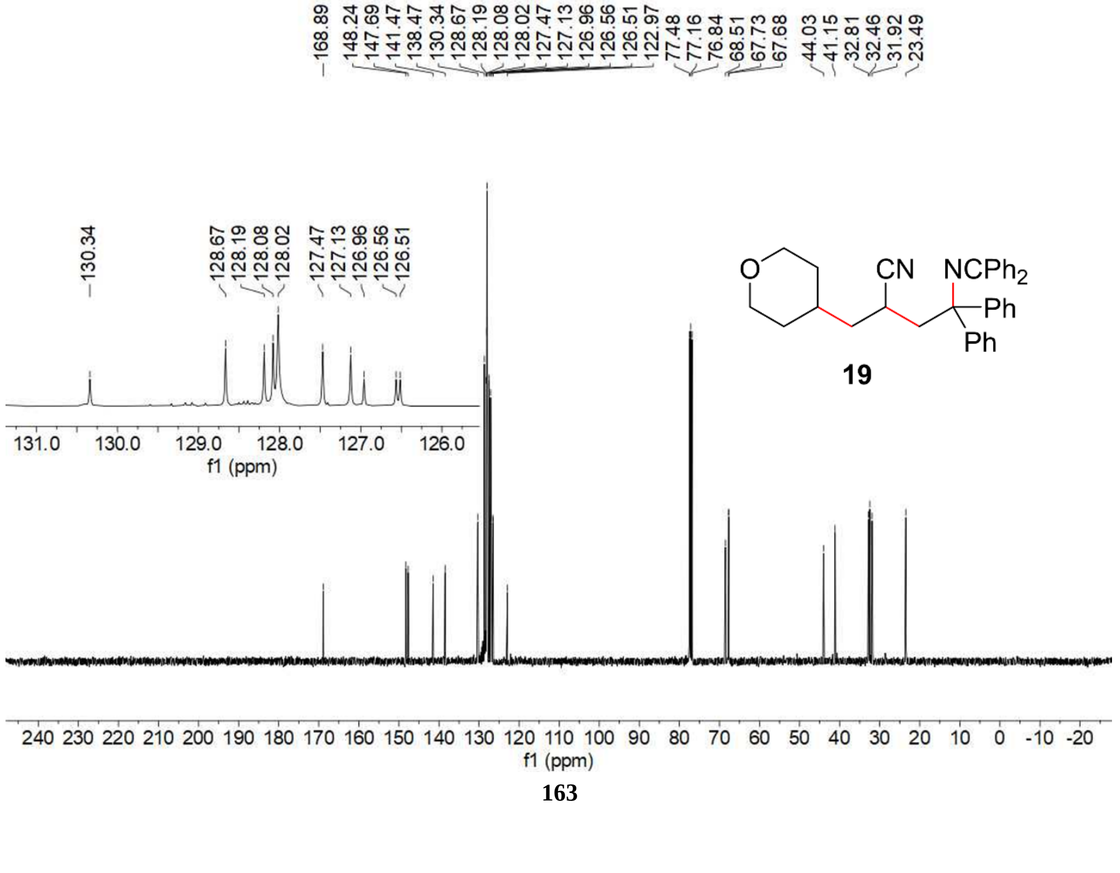

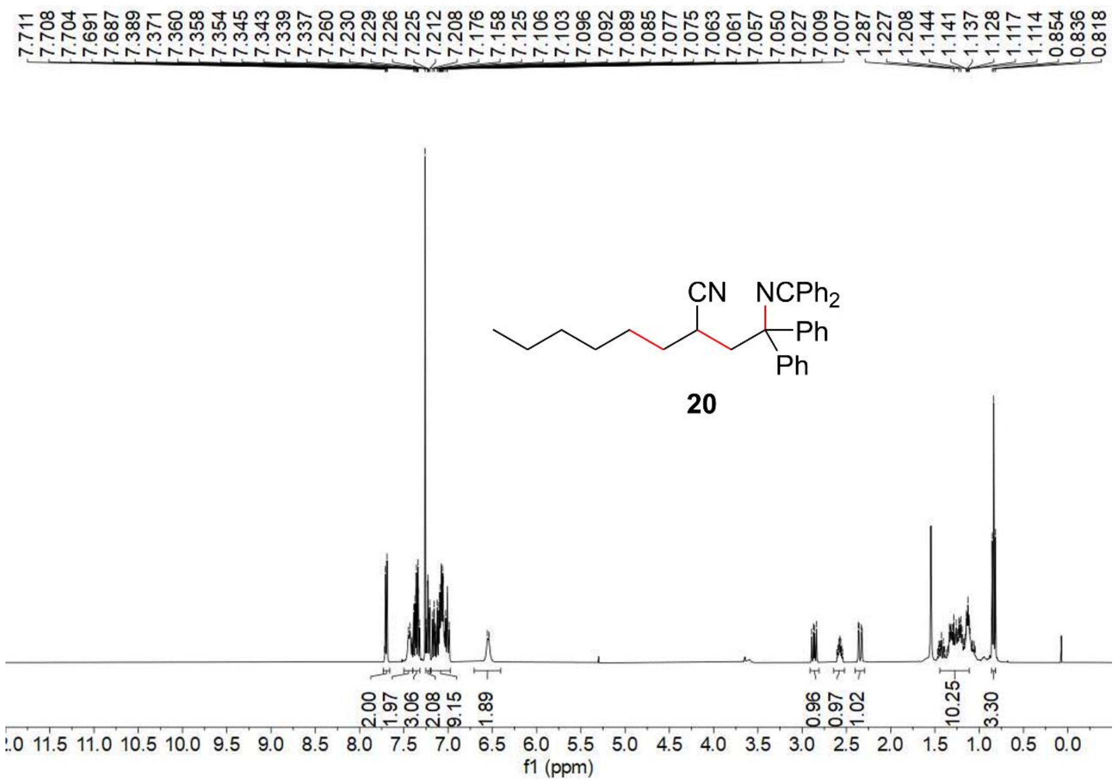

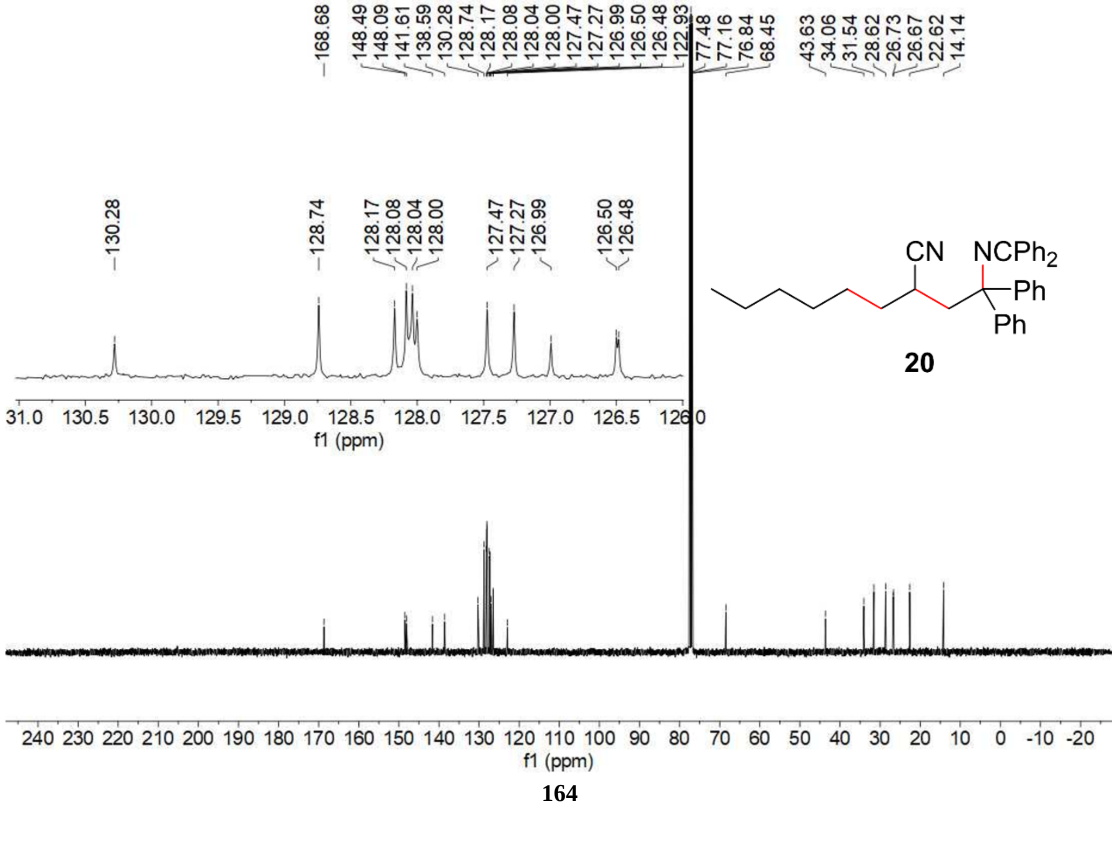

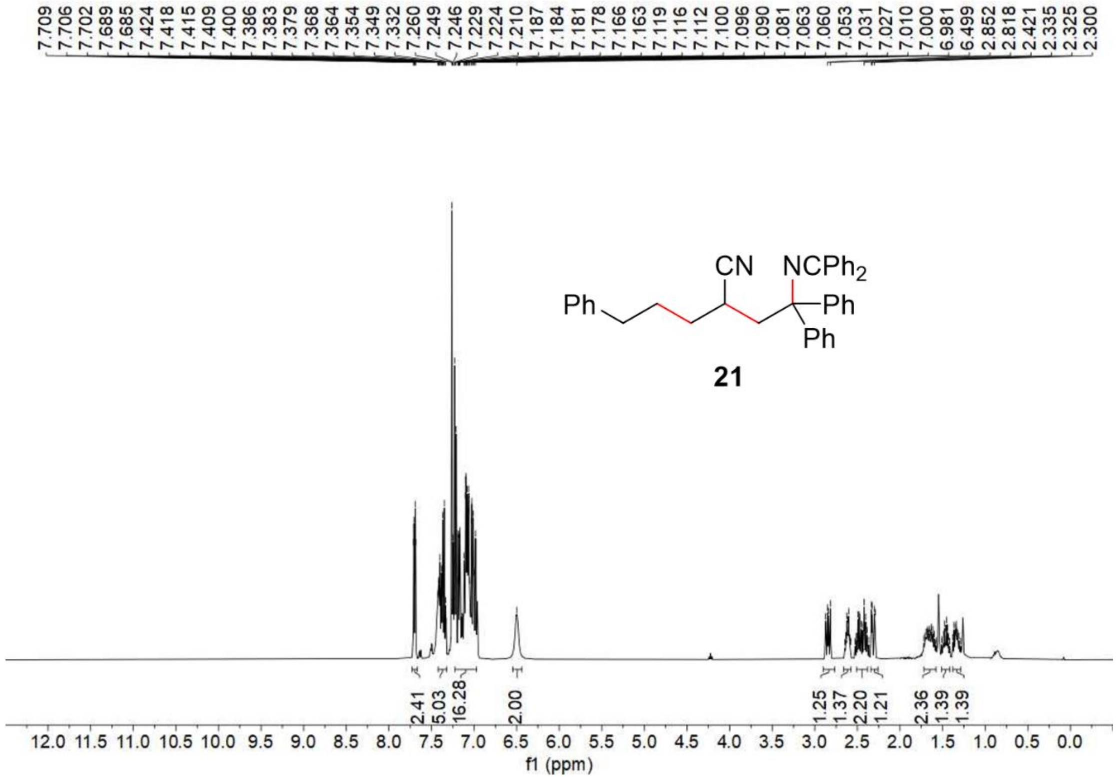
7.709
7.706
7.702
7.689
7.685
7.424
7.418
7.415
7.409
7.400
7.386
7.383
7.379
7.368
7.364
7.354
7.349
7.332
7.260
7.249
7.246
7.229
7.224
7.210
7.187
7.184
7.181
7.178
7.166
7.163
7.119
7.116
7.112
7.100
7.096
7.090
7.081
7.063
7.060
7.053
7.031
7.027
7.010
7.000
6.981
6.499
2.852
2.818
2.421
2.335
2.325
2.300
CN
NCPh2
Ph
Ph
Ph
21
2.41
5.03
16.28
2.00
1.25
1.37
2.20
1.21
2.36
1.39
1.39
f1 (ppm)

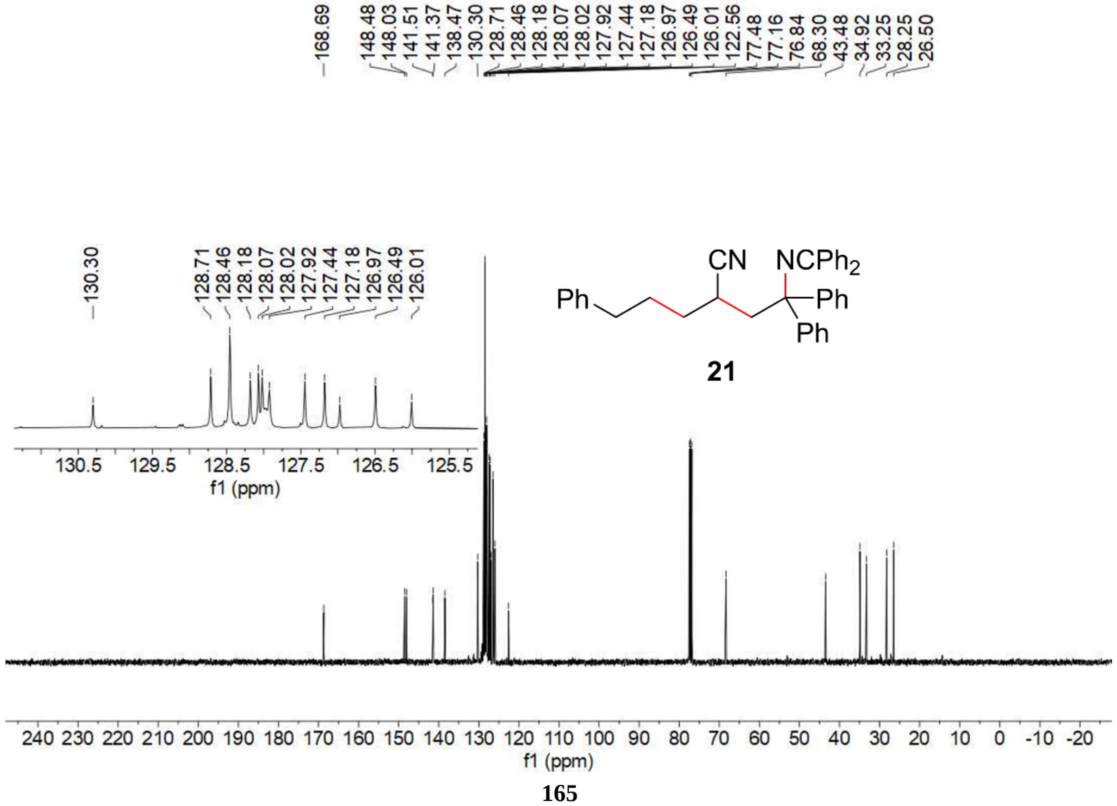
168.69
148.48
148.03
141.51
141.37
138.47
130.30
128.71
128.46
128.18
128.07
128.02
127.92
127.44
127.18
126.97
126.49
126.01
122.56
77.48
77.16
76.84
68.30
43.48
34.92
33.25
28.25
26.50
CN
NCPh2
Ph
Ph
Ph
21
f1 (ppm)

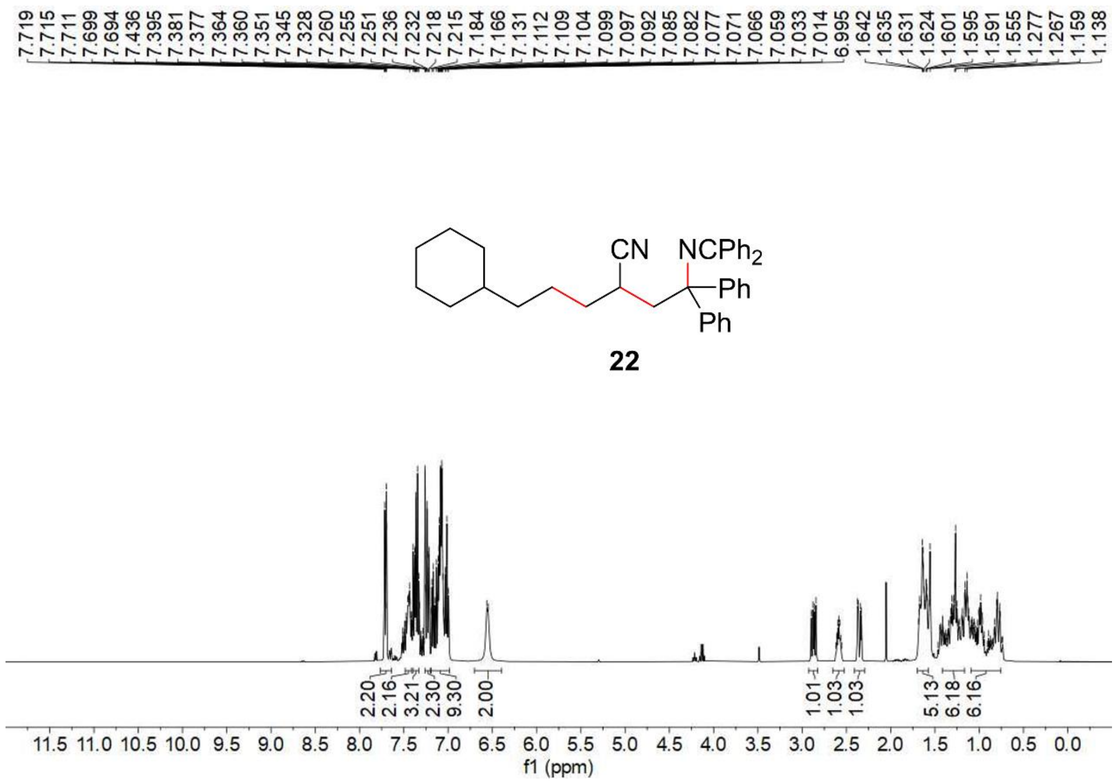
7.719 7.715 7.711 7.699 7.694 7.436 7.395 7.381 7.377 7.364 7.360 7.351 7.345 7.328 7.260 7.255 7.251 7.236 7.232 7.218 7.215 7.184 7.166 7.131 7.112 7.109 7.104 7.099 7.097 7.092 7.085 7.082 7.077 7.071 7.066 7.059 7.033 7.014 6.995 1.642 1.635 1.631 1.624 1.601 1.595 1.591 1.555 1.277 1.267 1.159 1.138
CN
NCPh2
Ph
Ph
22
2.20 2.16 3.21 2.30 9.30 2.00 1.01 1.03 1.03 5.13 6.18 6.16
11.5 11.0 10.5 10.0 9.5 9.0 8.5 8.0 7.5 7.0 6.5 6.0 5.5 5.0 4.5 4.0 3.5 3.0 2.5 2.0 1.5 1.0 0.5 0.0
f1 (ppm)

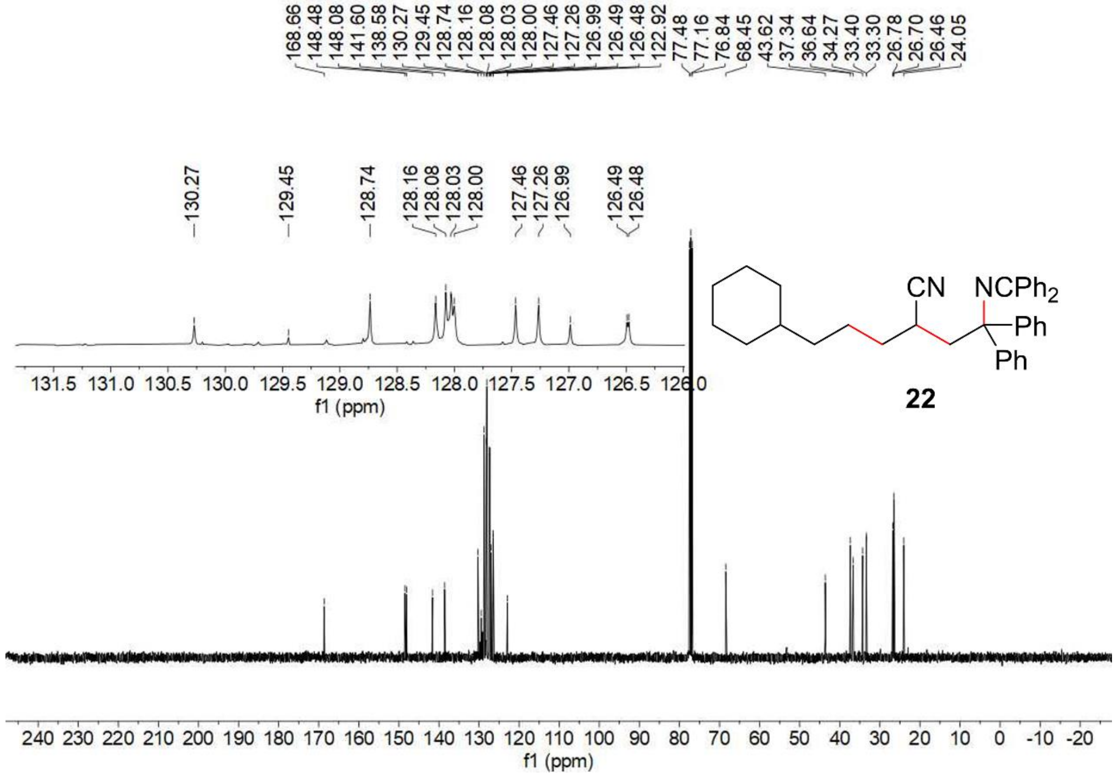
168.66 148.48 148.08 141.60 138.58 130.27 129.45 128.74 128.16 128.08 128.03 128.00 127.46 127.26 126.99 126.49 126.48 122.92 77.48 77.16 76.84 68.45 43.62 37.34 36.64 34.27 33.40 33.30 26.78 26.70 26.46 24.05
130.27 129.45 128.74 128.16 128.08 128.03 128.00 127.46 127.26 126.99 126.49 126.48
131.5 131.0 130.5 130.0 129.5 129.0 128.5 128.0 127.5 127.0 126.5 126.0
f1 (ppm)
CN
NCPh2
Ph
Ph
22
240 230 220 210 200 190 180 170 160 150 140 130 120 110 100 90 80 70 60 50 40 30 20 10 0 -10 -20
f1 (ppm)

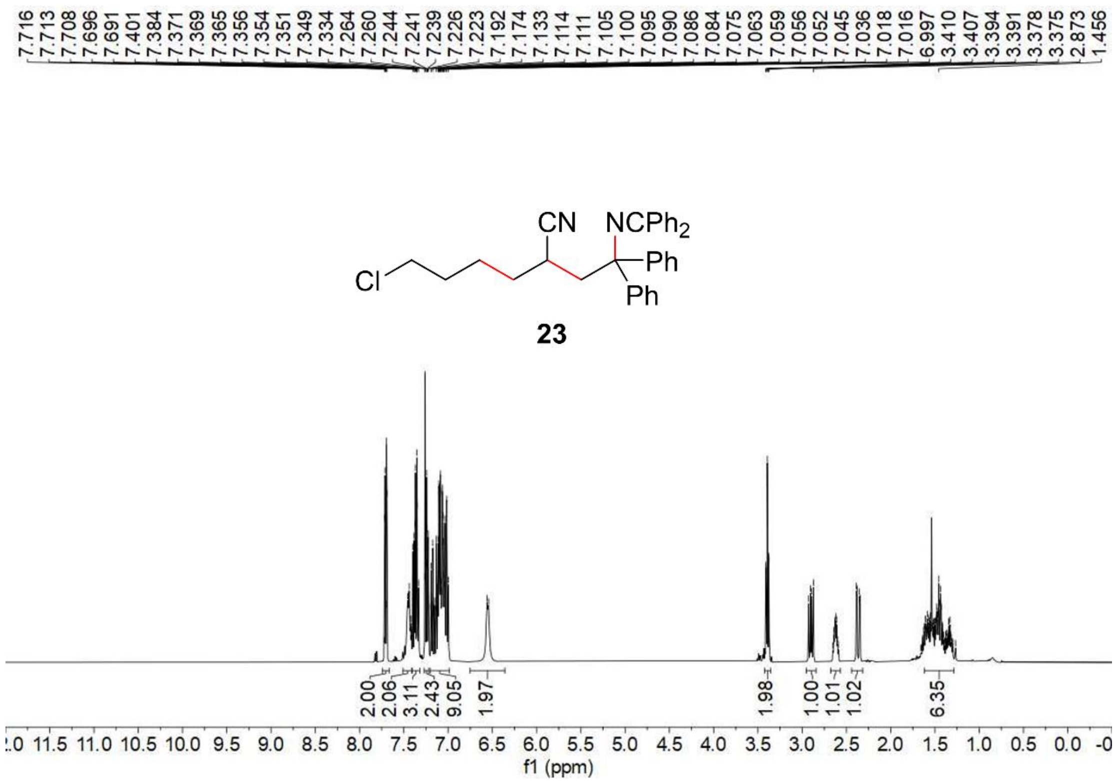
7.716 7.713 7.708 7.696 7.691 7.401 7.384 7.371 7.369 7.365 7.356 7.354 7.351 7.349 7.334 7.264 7.260 7.244 7.241 7.239 7.226 7.223 7.192 7.174 7.133 7.114 7.111 7.105 7.100 7.095 7.090 7.086 7.084 7.075 7.063 7.059 7.056 7.052 7.045 7.036 7.018 7.016 6.997 3.410 3.407 3.394 3.391 3.378 3.375 2.873 1.456
CN
NCPh2
Ph
Cl
Ph
23
2.00 2.06 3.11 2.43 9.05 1.97 1.98 1.00 1.01 1.02 6.35
f1 (ppm)

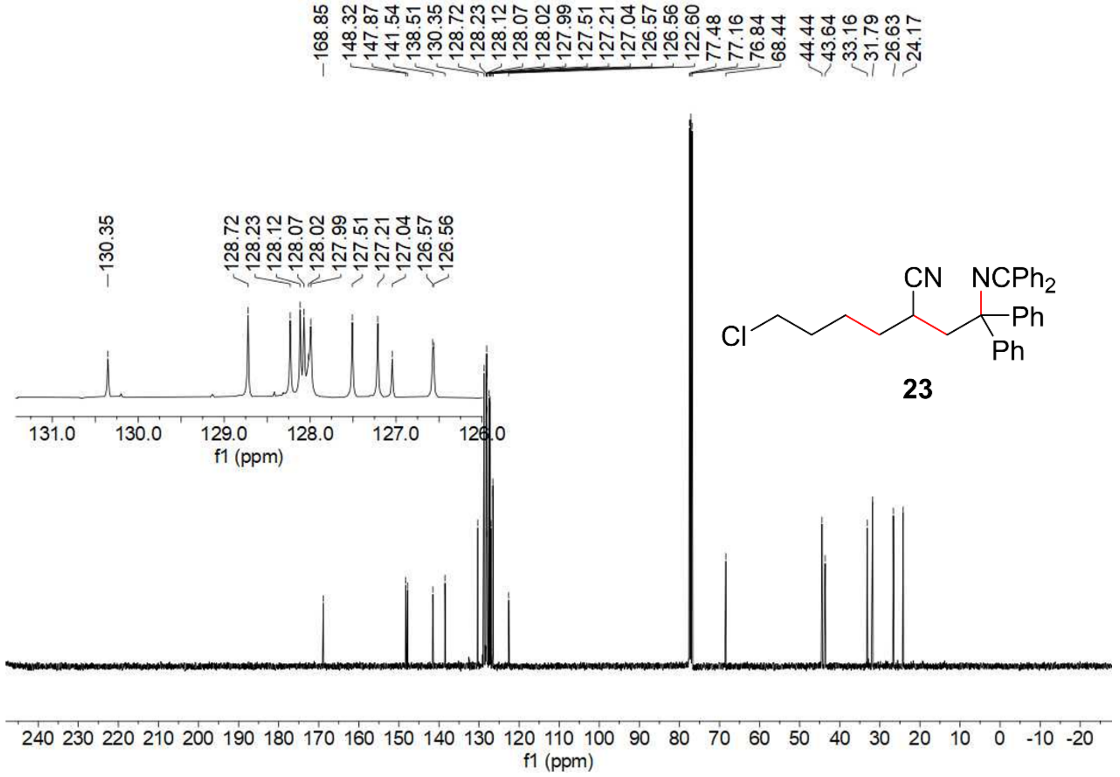
168.85 148.32 147.87 141.54 138.51 130.35 128.72 128.23 128.12 128.07 128.02 127.99 127.51 127.21 127.04 126.57 126.56 122.60 77.48 77.16 76.84 68.44 44.44 43.64 33.16 31.79 26.63 24.17
130.35
128.72 128.23 128.12 128.07 128.02 127.99 127.51 127.21 127.04 126.57 126.56
f1 (ppm)
CN
NCPh2
Ph
Cl
Ph
23
f1 (ppm)

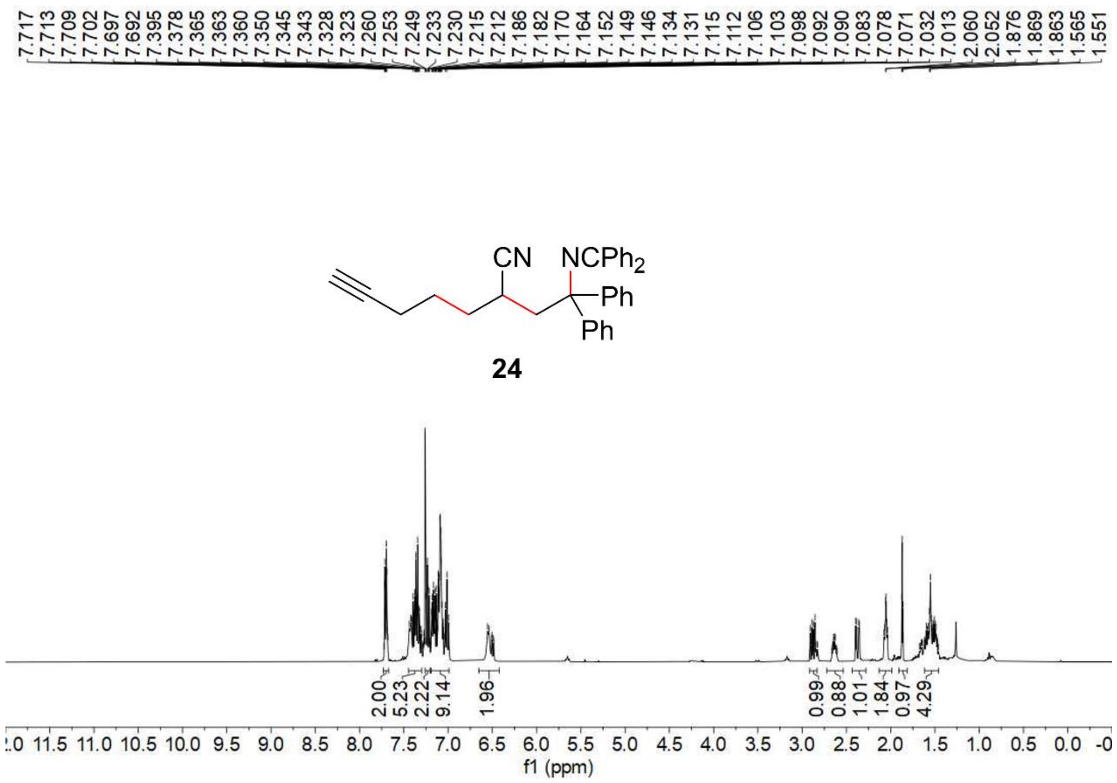
7.717 7.713 7.709 7.702 7.697 7.692 7.395 7.378 7.365 7.363 7.360 7.350 7.345 7.343 7.328 7.323 7.260 7.253 7.249 7.233 7.230 7.215 7.212 7.186 7.182 7.170 7.164 7.152 7.149 7.146 7.134 7.131 7.115 7.112 7.106 7.103 7.098 7.092 7.090 7.083 7.078 7.071 7.032 7.013 2.060 2.052 1.876 1.869 1.863 1.565 1.551
CN
NCPh2
Ph
Ph
24
2.00 5.23 2.22 9.14 1.96 0.99 0.88 1.01 1.84 0.97 4.29
f1 (ppm)

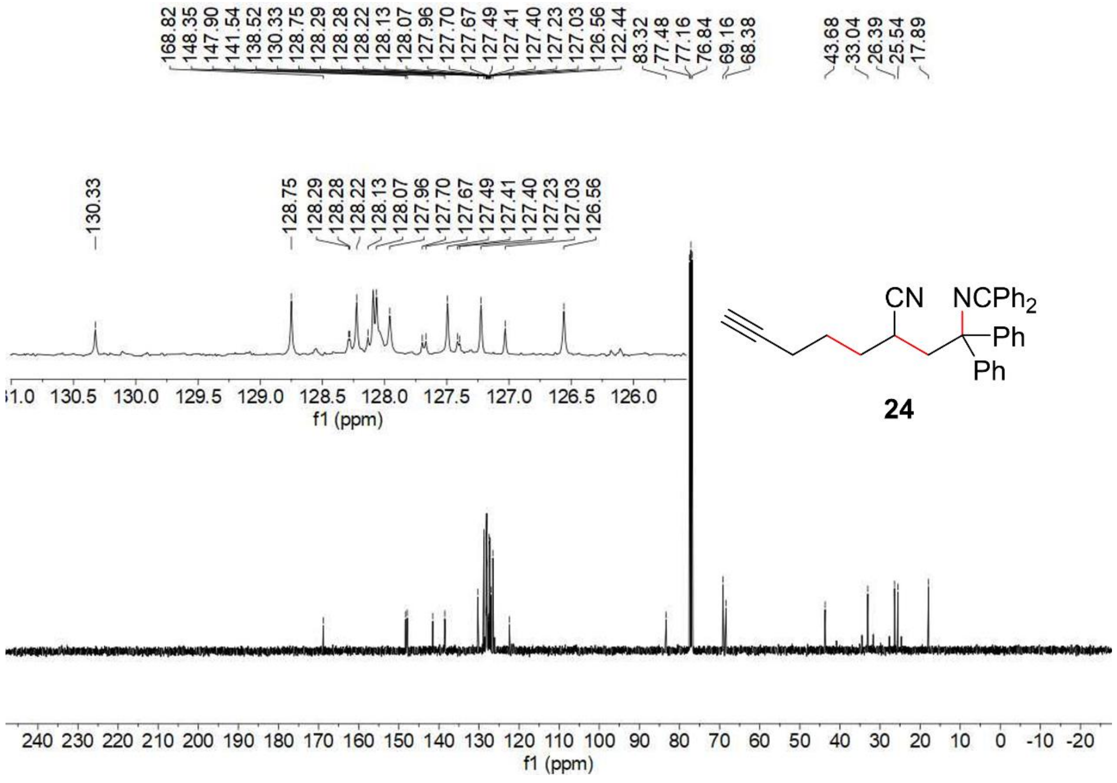
168.82 148.35 147.90 141.54 138.52 130.33 128.75 128.29 128.28 128.22 128.13 128.07 127.96 127.70 127.67 127.49 127.41 127.40 127.23 127.03 126.56 122.44 83.32 77.48 77.16 76.84 69.16 68.38 43.68 33.04 26.39 25.54 17.89
130.33
128.75 128.29 128.28 128.22 128.13 128.07 127.96 127.70 127.67 127.49 127.41 127.40 127.23 127.03 126.56
f1 (ppm)
CN
NCPh2
Ph
Ph
24
f1 (ppm)

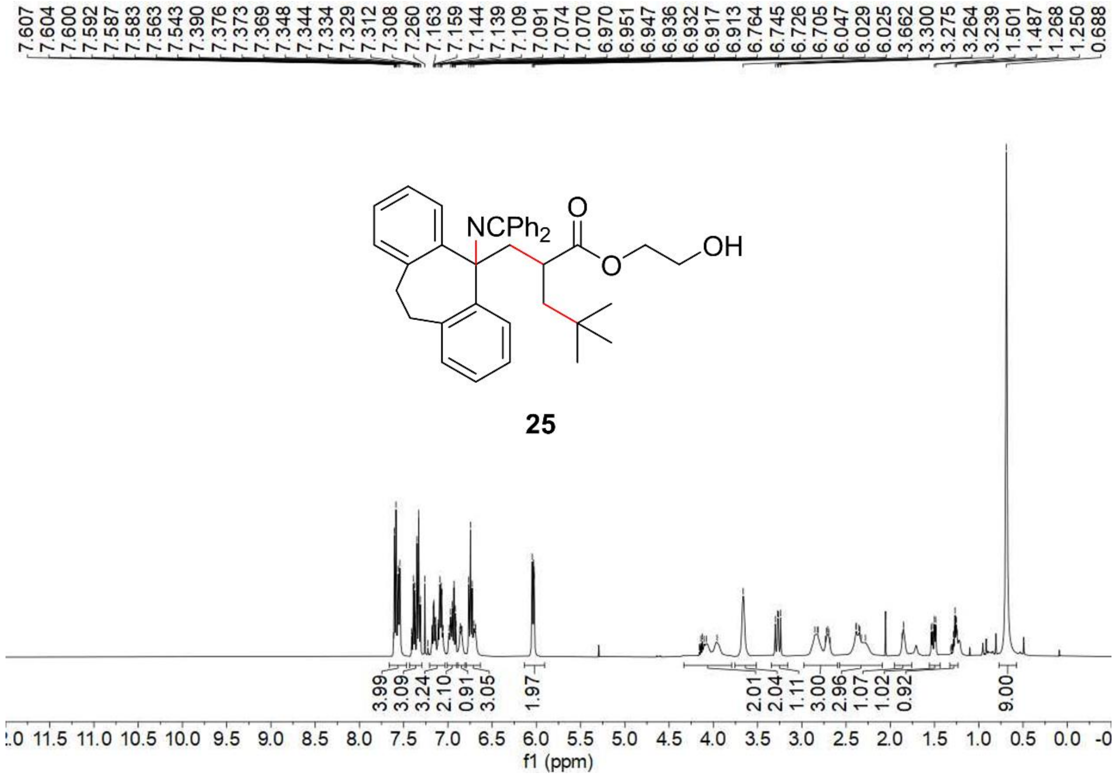
7.607 7.604 7.600 7.592 7.587 7.583 7.563 7.543 7.390 7.376 7.373 7.369 7.348 7.344 7.334 7.329 7.312 7.308 7.260 7.163 7.159 7.144 7.139 7.109 7.091 7.074 7.070 6.970 6.951 6.947 6.936 6.932 6.917 6.913 6.764 6.745 6.726 6.705 6.047 6.029 6.025 3.662 3.300 3.275 3.264 3.239 1.501 1.487 1.268 1.250 0.688
NCPh2
O
OH
3.99 3.09 3.24 2.10 0.91 3.05 1.97 2.01 2.04 1.11 3.00 2.96 1.07 1.02 0.92 9.00
f1 (ppm)

25

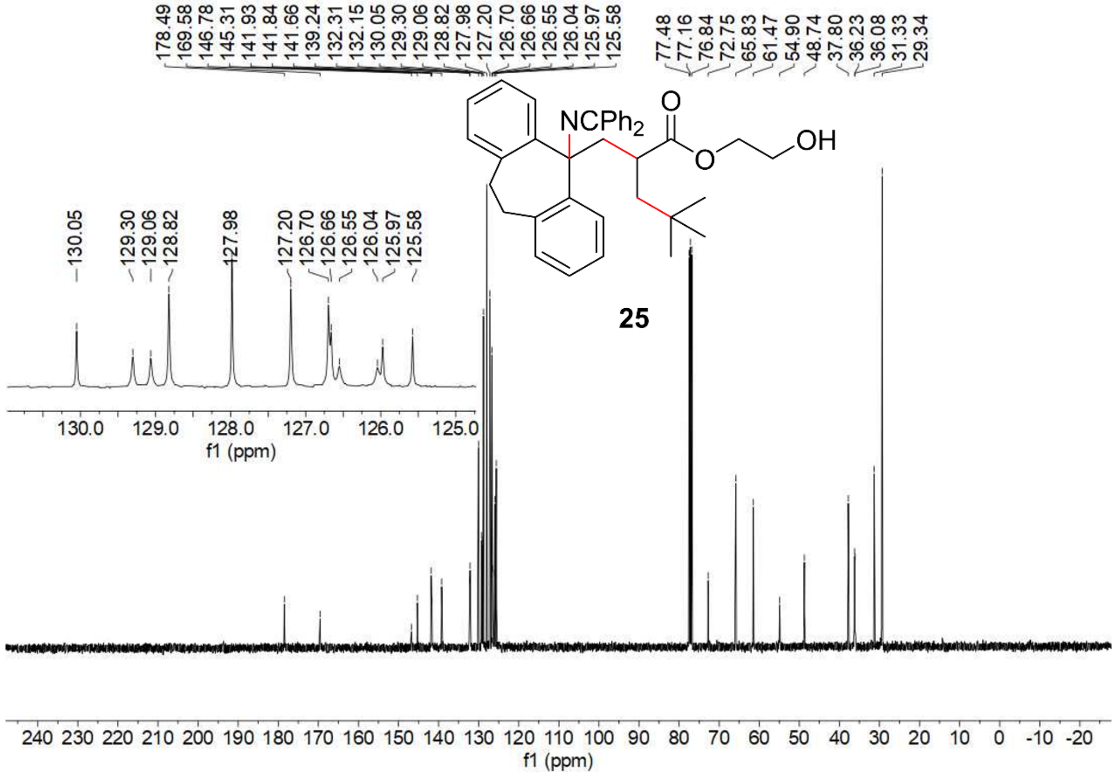
178.49 169.58 146.78 145.31 141.93 141.84 141.66 139.24 132.31 132.15 130.05 129.30 129.06 128.82 127.98 127.20 126.70 126.66 126.55 126.04 125.97 125.58
77.48 77.16 76.84 72.75 65.83 61.47 54.90 48.74 37.80 36.23 36.08 31.33 29.34
NCPh2
O
OH
130.05 129.30 129.06 128.82 127.98 127.20 126.70 126.66 126.55 126.04 125.97 125.58
f1 (ppm)
f1 (ppm)

25

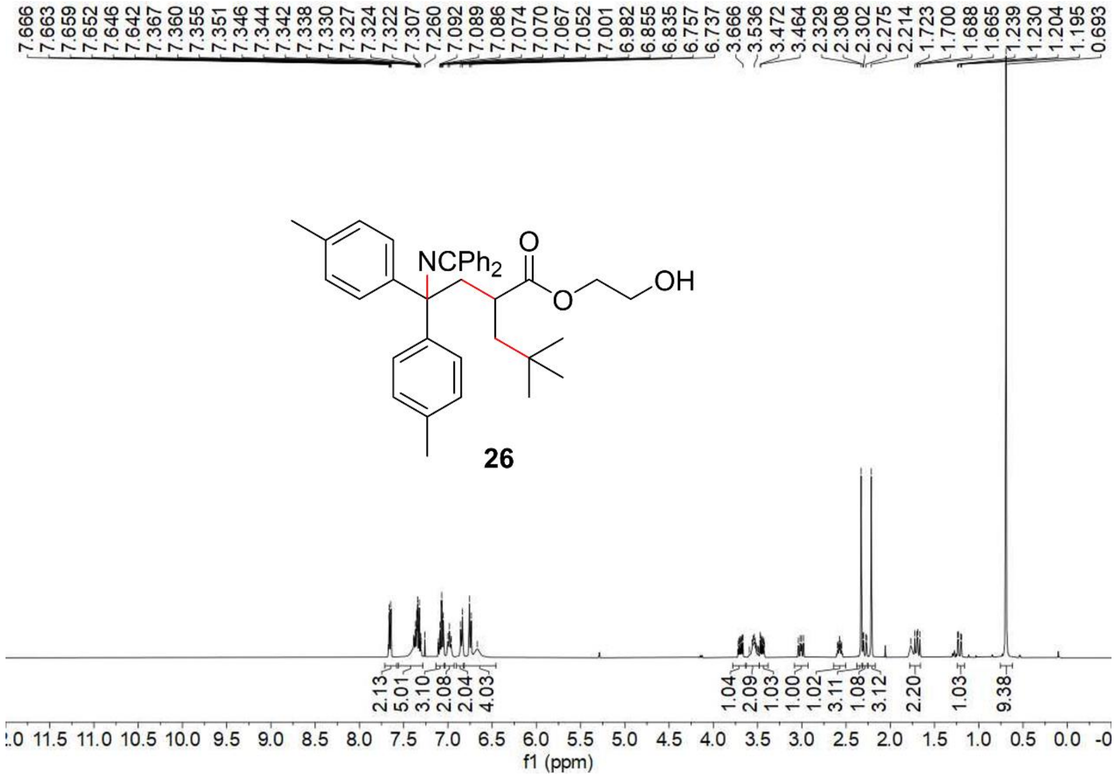
7.666 7.663 7.659 7.652 7.646 7.642 7.367 7.360 7.355 7.351 7.346 7.344 7.342 7.338 7.330 7.327 7.324 7.322 7.307 7.260 7.092 7.089 7.086 7.074 7.070 7.067 7.052 7.001 6.982 6.855 6.835 6.757 6.737 3.666 3.536 3.472 3.464 2.329 2.308 2.302 2.275 2.214 1.723 1.700 1.688 1.665 1.239 1.230 1.204 1.195 0.693
NCPh2
O
O
OH
26
2.13 5.01 3.10 2.08 2.04 4.03 1.04 2.09 1.03 1.00 1.02 3.11 1.08 3.12 2.20 1.03 9.38
f1 (ppm)

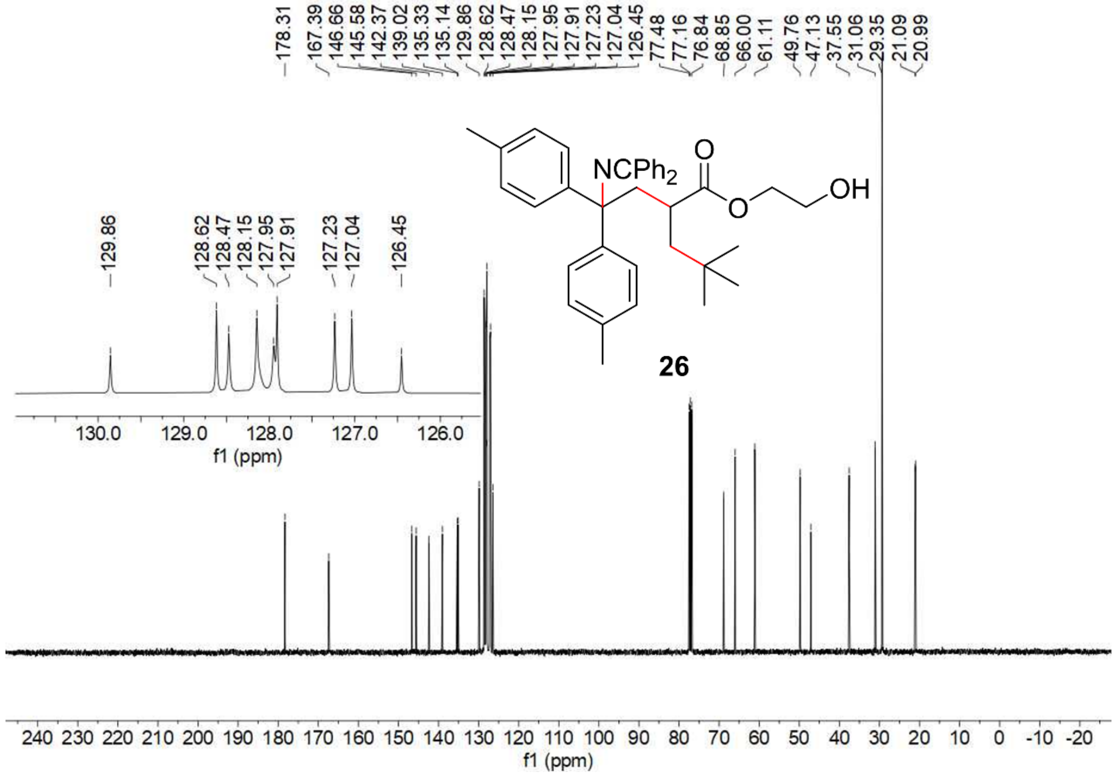
178.31 167.39 146.66 145.58 142.37 139.02 135.33 135.14 129.86 128.62 128.47 128.15 127.95 127.91 127.23 127.04 126.45 77.48 77.16 76.84 68.85 66.00 61.11 49.76 47.13 37.55 31.06 29.35 21.09 20.99
129.86 128.62 128.47 128.15 127.95 127.91 127.23 127.04 126.45
NCPh2
O
O
OH
26
f1 (ppm)
f1 (ppm)

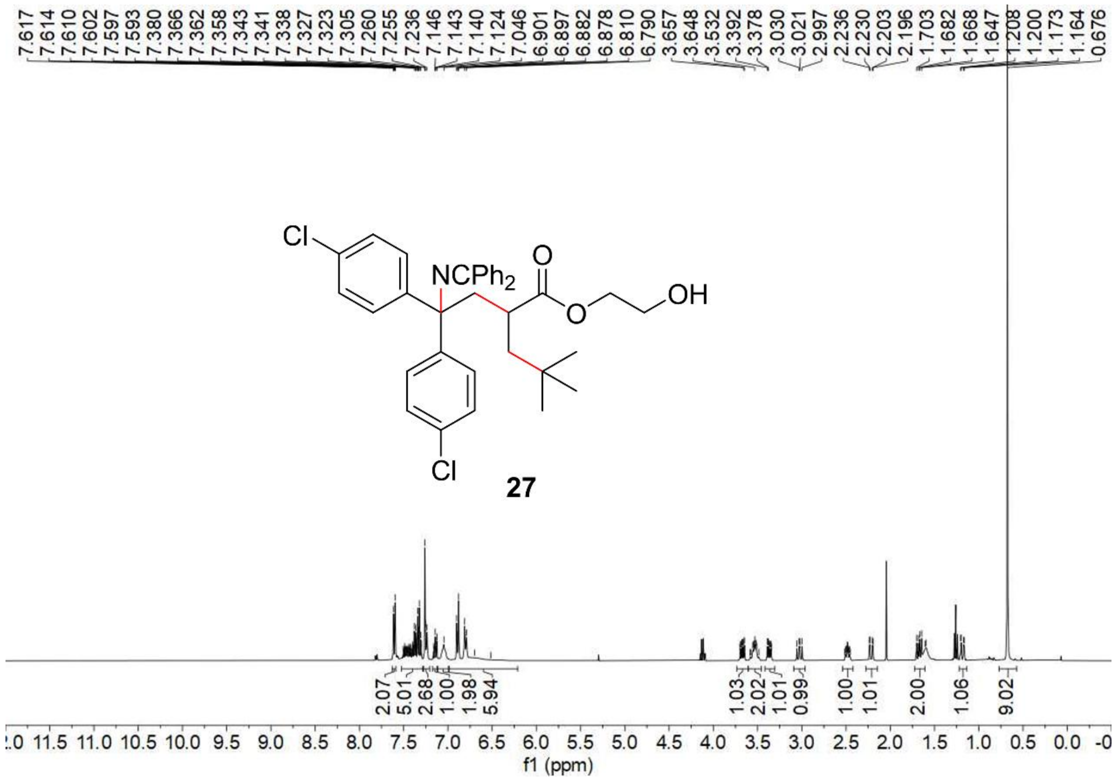

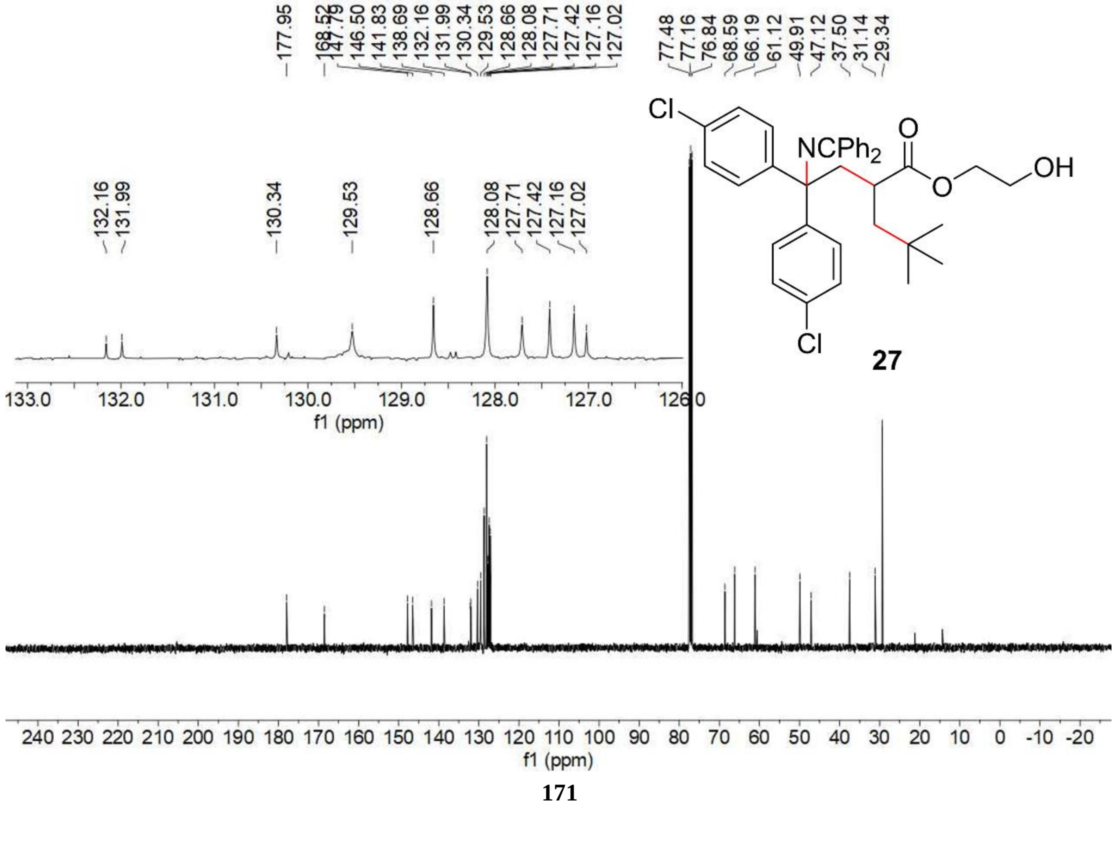

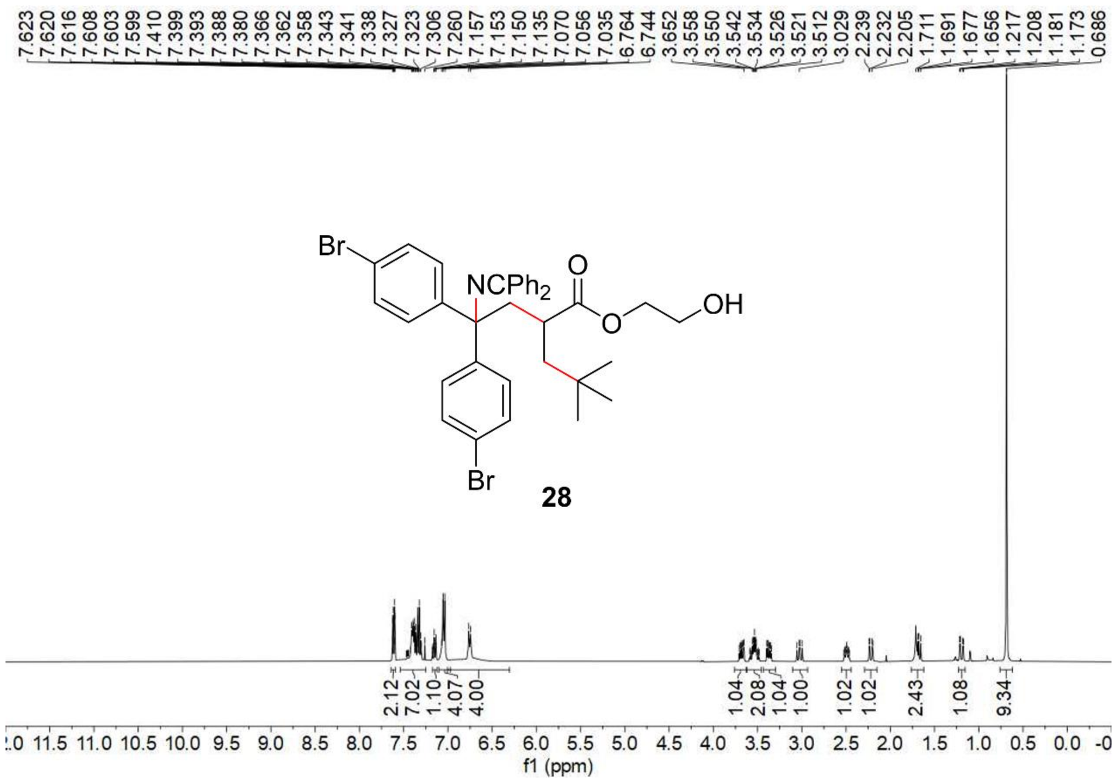
7.623 7.620 7.616 7.608 7.603 7.599 7.410 7.399 7.393 7.388 7.380 7.366 7.362 7.358 7.343 7.341 7.338 7.327 7.323 7.306 7.260 7.157 7.153 7.150 7.135 7.070 7.056 7.035 6.764 6.744 3.652 3.558 3.550 3.542 3.534 3.526 3.521 3.512 3.029 2.239 2.232 2.205 1.711 1.691 1.677 1.656 1.217 1.208 1.181 1.173 0.686
Br
NCPh2
O
O
OH
Br
28
2.12 7.02 1.10 4.07 4.00 1.04 2.08 1.04 1.00 1.02 1.02 2.43 1.08 9.34
f1 (ppm)

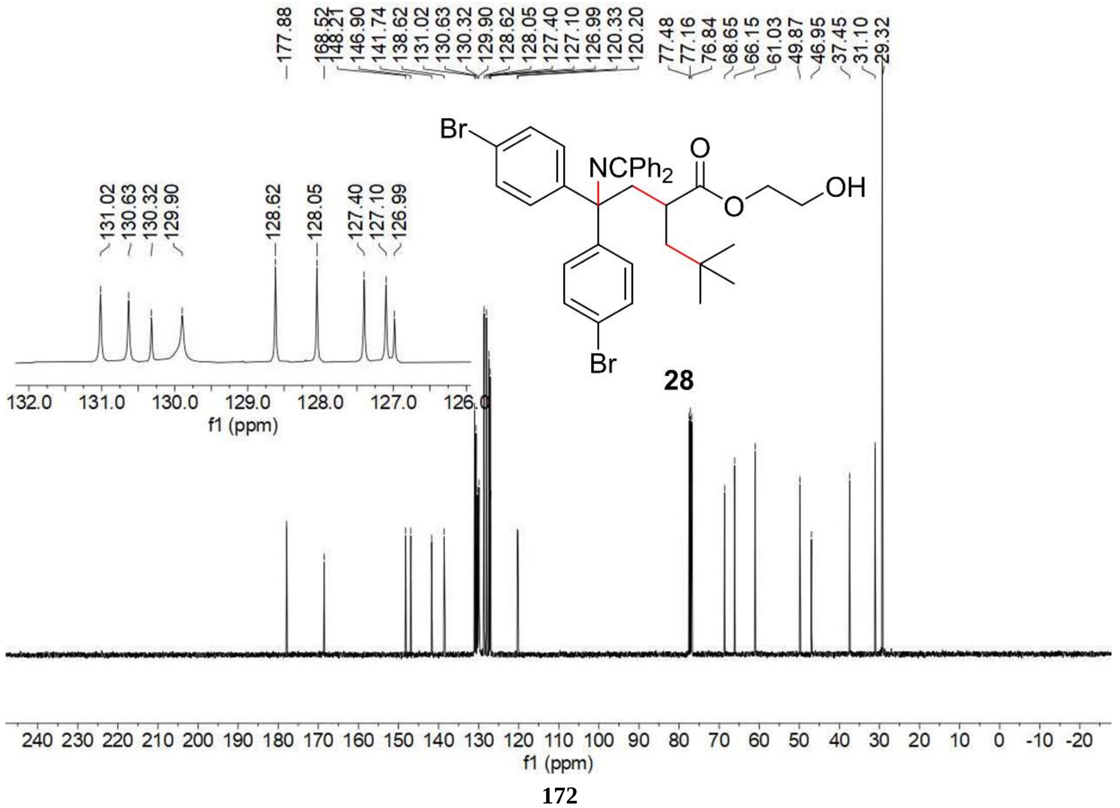
177.88 168.52 148.21 146.90 141.74 138.62 131.02 130.63 130.32 129.90 128.62 128.05 127.40 127.10 126.99 120.33 120.20 77.48 77.16 76.84 68.65 66.15 61.03 49.87 46.95 37.45 31.10 29.32
131.02 130.63 130.32 129.90 128.62 128.05 127.40 127.10 126.99
f1 (ppm)
Br
NCPh2
O
O
OH
Br
28
f1 (ppm)

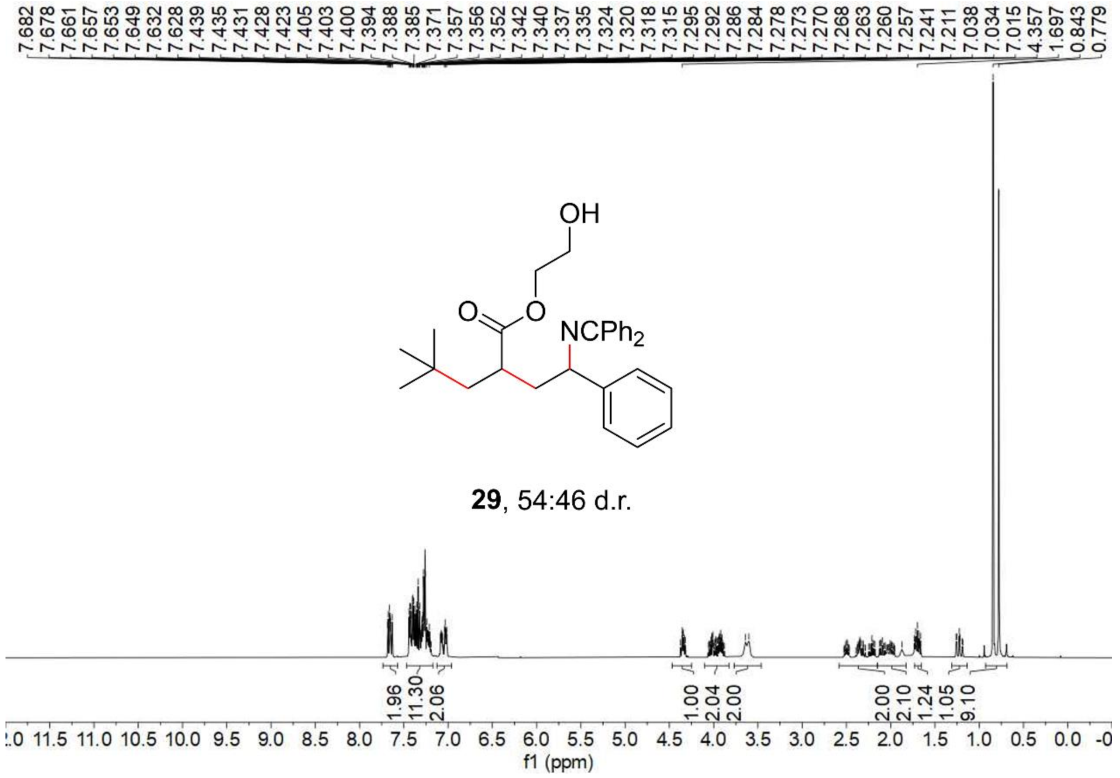
7.682 7.678 7.661 7.657 7.653 7.649 7.632 7.628 7.439 7.435 7.431 7.428 7.423 7.405 7.403 7.400 7.394 7.388 7.385 7.371 7.357 7.356 7.352 7.342 7.340 7.337 7.335 7.324 7.320 7.318 7.315 7.295 7.292 7.286 7.284 7.278 7.273 7.270 7.268 7.263 7.260 7.257 7.241 7.211 7.038 7.034 7.015 4.357 1.697 0.843 0.779
OH
O
O
NCPh2
29, 54:46 d.r.
1.96 11.30 2.06 1.00 2.04 2.00 2.00 2.10 1.24 1.05 9.10
f1 (ppm)

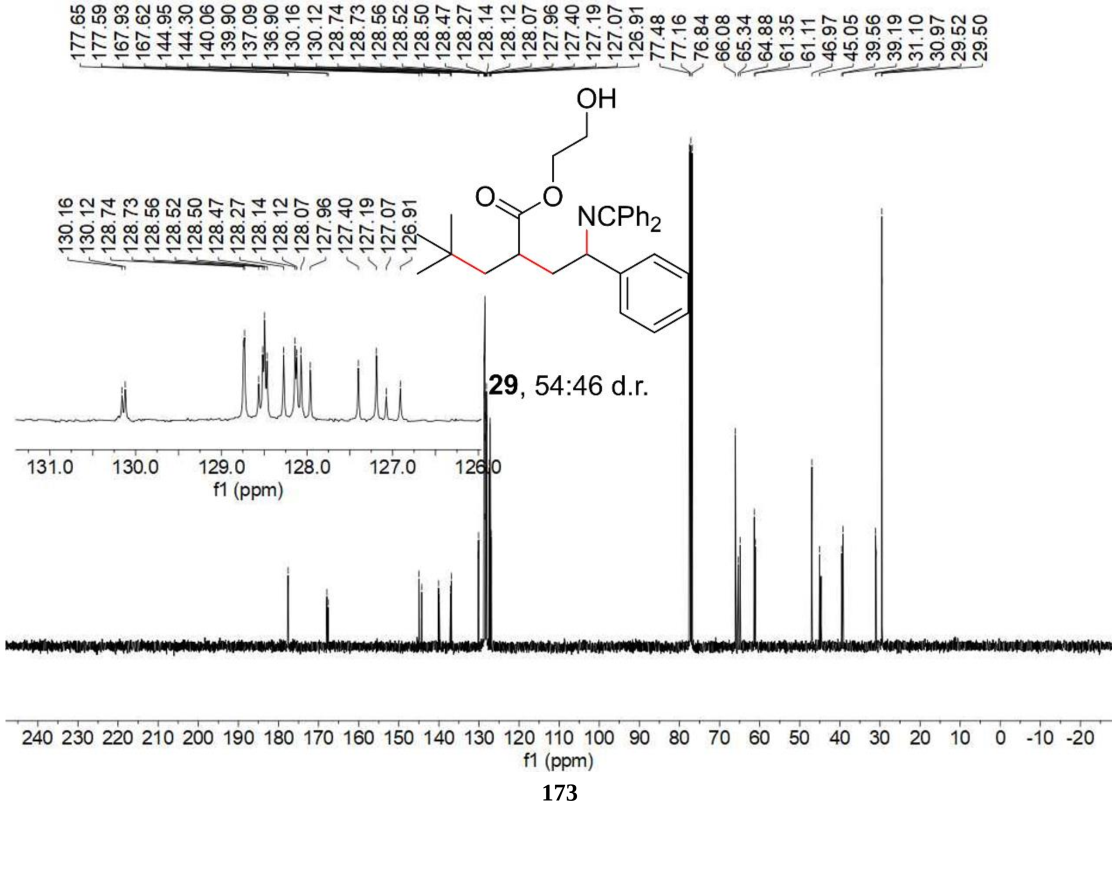
177.65 177.59 167.93 167.62 144.95 144.30 140.06 139.90 137.09 136.90 130.16 130.12 128.74 128.73 128.56 128.52 128.50 128.47 128.27 128.14 128.12 128.07 127.96 127.40 127.19 127.07 126.91 77.48 77.16 76.84 66.08 65.34 64.88 61.35 61.11 46.97 45.05 39.56 39.19 31.10 30.97 29.52 29.50
OH
O
O
NCPh2
29, 54:46 d.r.
130.16 130.12 128.74 128.73 128.56 128.52 128.50 128.47 128.27 128.14 128.12 128.07 127.96 127.40 127.19 127.07 126.91
f1 (ppm)
f1 (ppm)

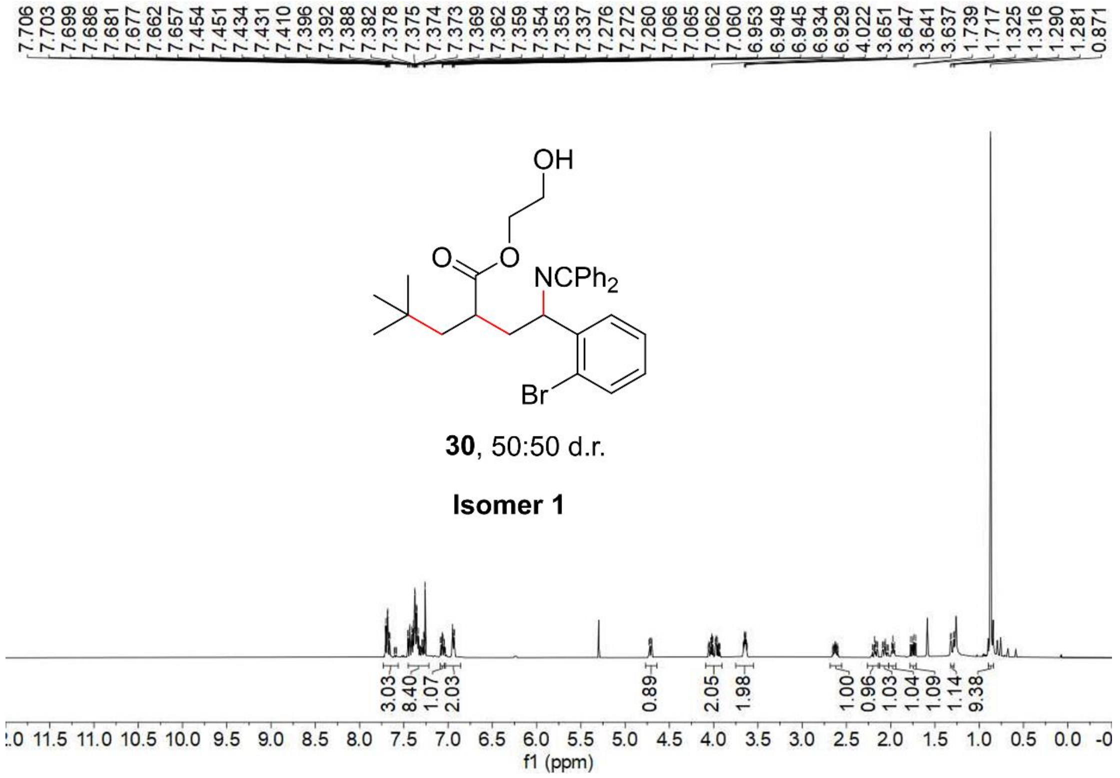
7.706 7.703 7.699 7.686 7.681 7.677 7.662 7.657 7.454 7.451 7.434 7.431 7.410 7.396 7.392 7.388 7.382 7.378 7.375 7.374 7.373 7.369 7.362 7.359 7.354 7.353 7.337 7.276 7.272 7.260 7.066 7.065 7.062 7.060 6.953 6.949 6.945 6.934 6.929 4.022 3.651 3.647 3.641 3.637 1.739 1.717 1.325 1.316 1.290 1.281 0.871
OH
O
O
NCPh2
Br
30, 50:50 d.r.
Isomer 1
3.03 8.40 1.07 2.03 0.89 2.05 1.98 1.00 0.96 1.03 1.04 1.09 1.14 9.38
f1 (ppm)

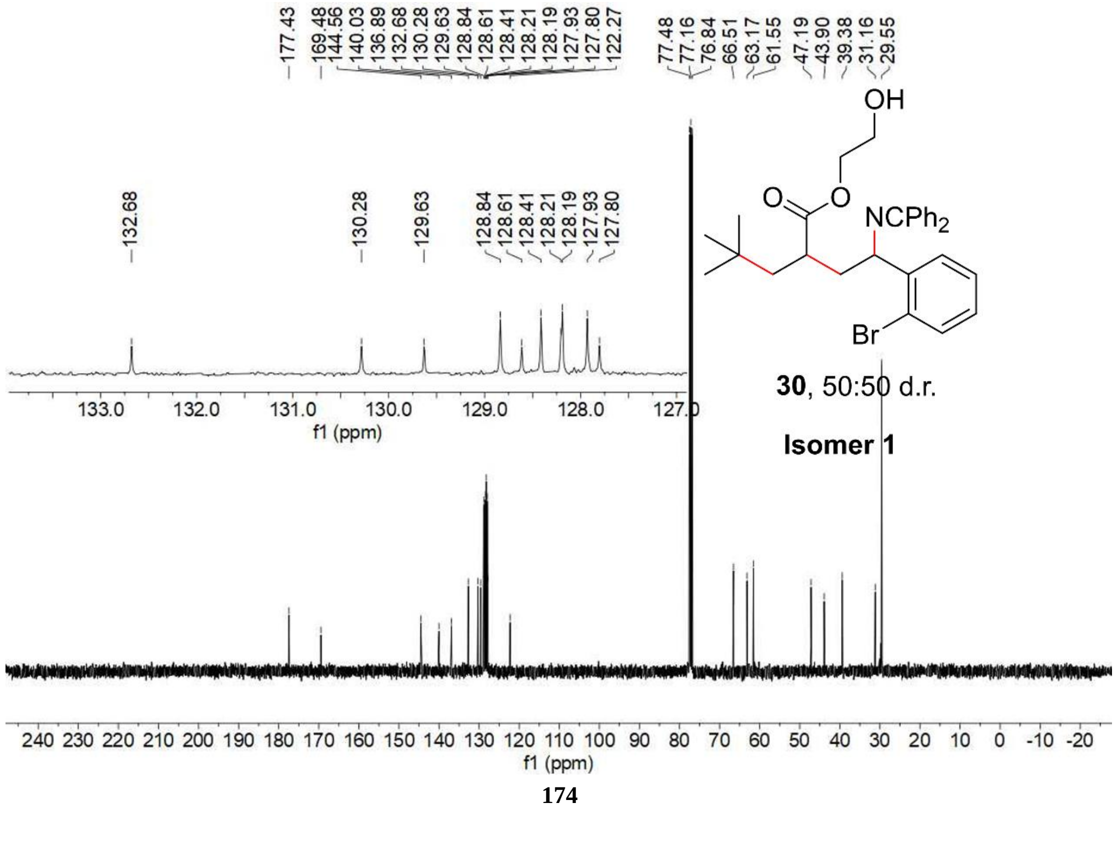
177.43 169.48 144.56 140.03 136.89 132.68 130.28 129.63 128.84 128.61 128.41 128.21 128.19 127.93 127.80 122.27 77.48 77.16 76.84 66.51 63.17 61.55 47.19 43.90 39.38 31.16 29.55
132.68 130.28 129.63 128.84 128.61 128.41 128.21 128.19 127.93 127.80
f1 (ppm)
OH
O
O
NCPh2
Br
30, 50:50 d.r.
Isomer 1
f1 (ppm)

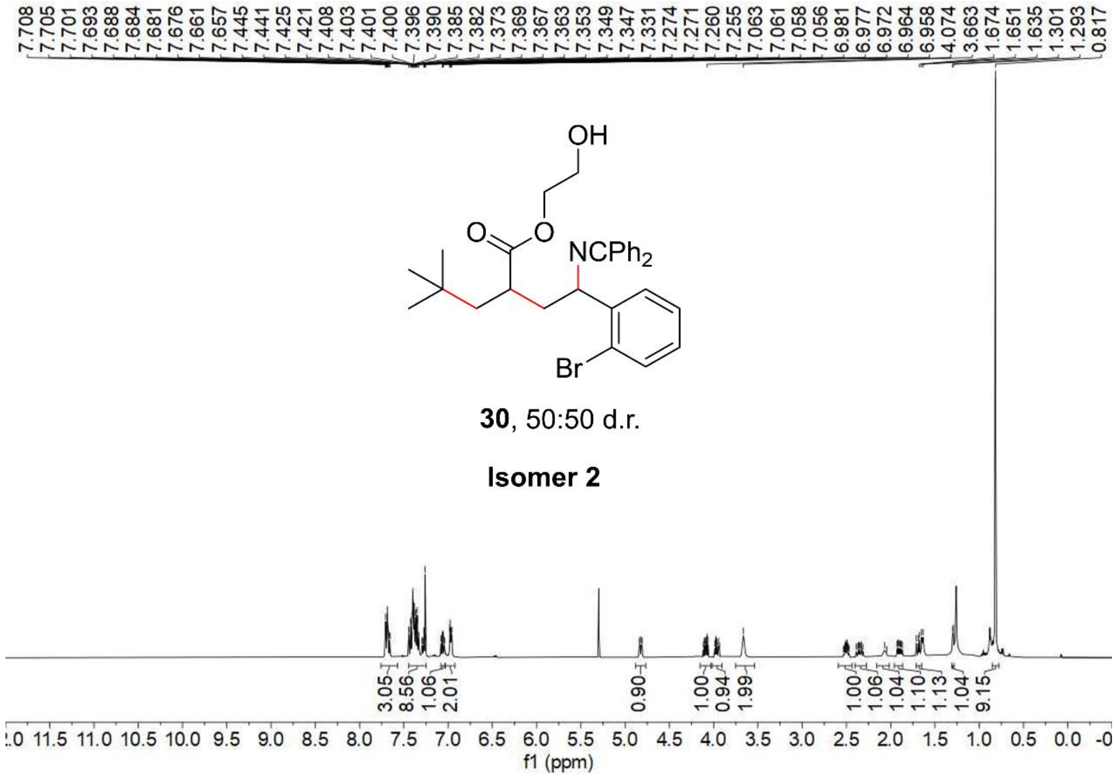
7.708 7.705 7.701 7.693 7.688 7.684 7.681 7.676 7.661 7.657 7.445 7.441 7.425 7.421 7.408 7.403 7.401 7.400 7.396 7.390 7.385 7.382 7.373 7.369 7.367 7.363 7.353 7.349 7.347 7.331 7.274 7.271 7.260 7.255 7.063 7.061 7.058 7.056 6.981 6.977 6.972 6.964 6.958 4.074 3.663 1.674 1.651 1.635 1.301 1.293 0.817
OH
O
O
NCPh2
Br
30, 50:50 d.r.
Isomer 2
3.05 8.56 1.06 2.01 0.90 1.00 0.94 1.99 1.00 1.06 1.04 1.10 1.13 1.04 9.15
f1 (ppm)

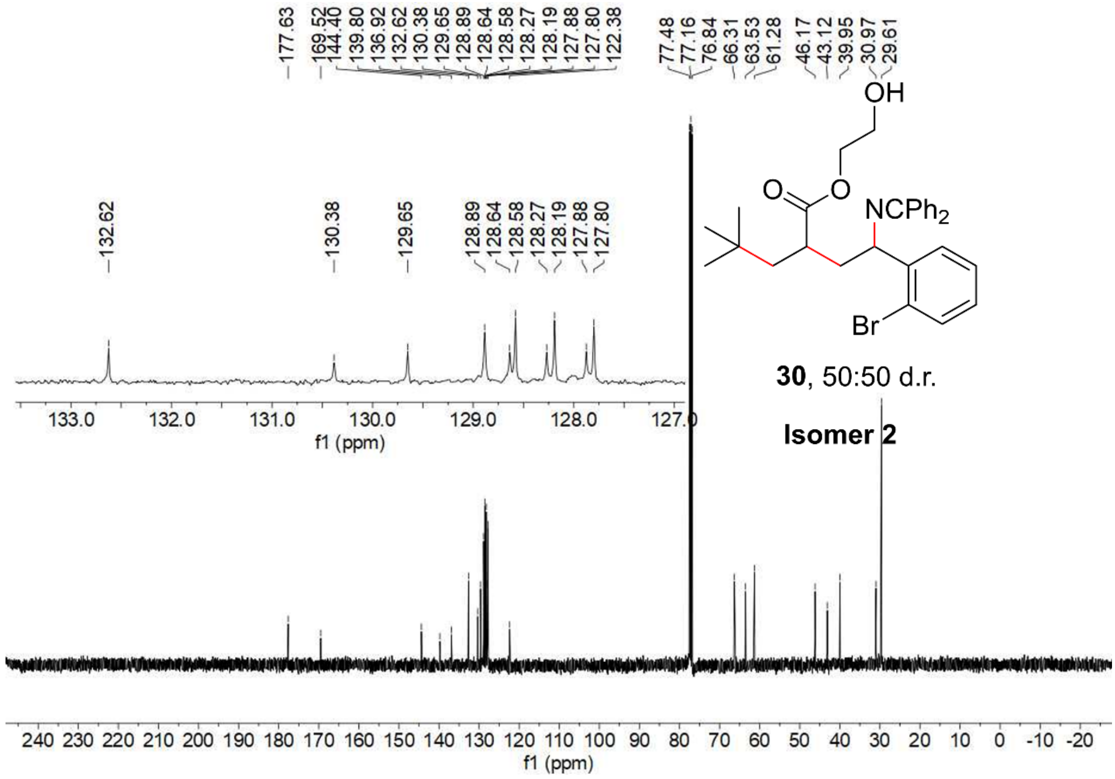
177.63 169.52 144.40 139.80 136.92 132.62 130.38 129.65 128.89 128.64 128.58 128.27 128.19 127.88 127.80 122.38 77.48 77.16 76.84 66.31 63.53 61.28 46.17 43.12 39.95 30.97 29.61
132.62 130.38 129.65 128.89 128.64 128.58 128.27 128.19 127.88 127.80
f1 (ppm)
OH
O
O
NCPh2
Br
30, 50:50 d.r.
Isomer 2
f1 (ppm)

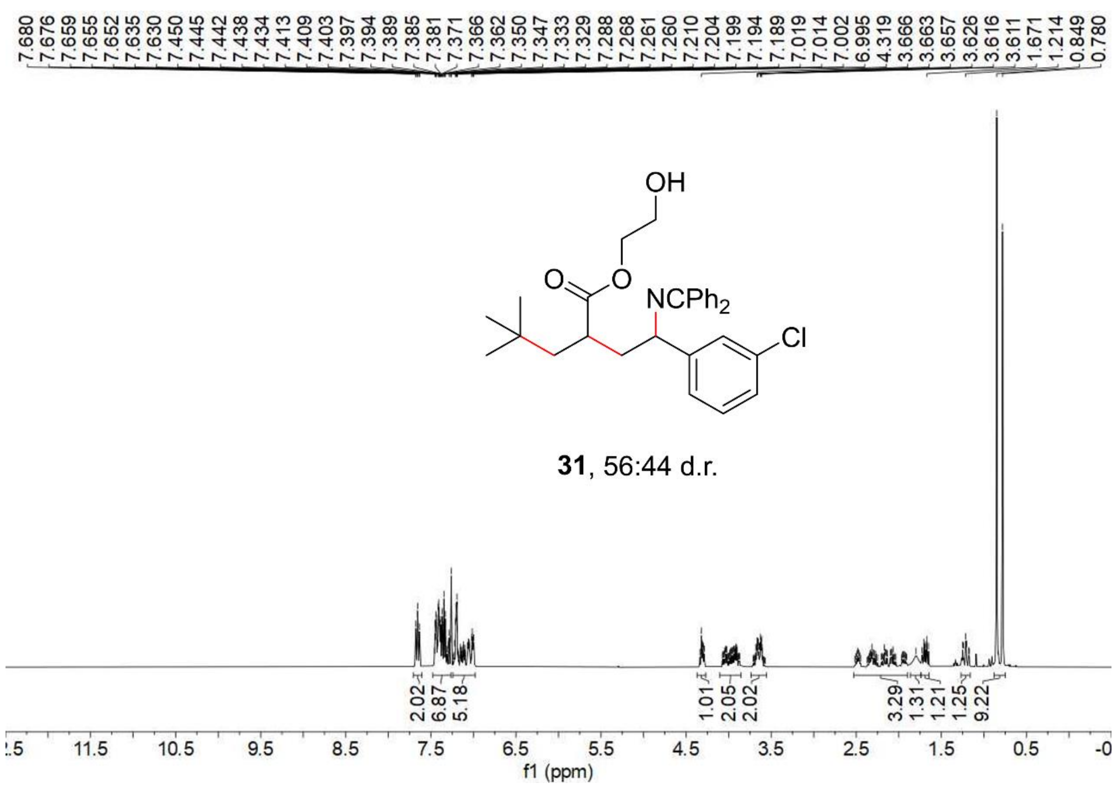
7.680 7.676 7.659 7.655 7.652 7.635 7.630 7.450 7.445 7.442 7.438 7.434 7.413 7.409 7.403 7.397 7.394 7.389 7.385 7.381 7.371 7.366 7.362 7.350 7.347 7.333 7.329 7.288 7.268 7.261 7.260 7.210 7.204 7.199 7.194 7.189 7.019 7.014 7.002 6.995 4.319 3.666 3.663 3.657 3.626 3.616 3.611 1.671 1.214 0.849 0.780
OH
O
O
NCPh2
Cl
31, 56:44 d.r.
2.02 6.87 5.18 1.01 2.05 2.02 3.29 1.31 1.21 1.25 9.22
f1 (ppm)

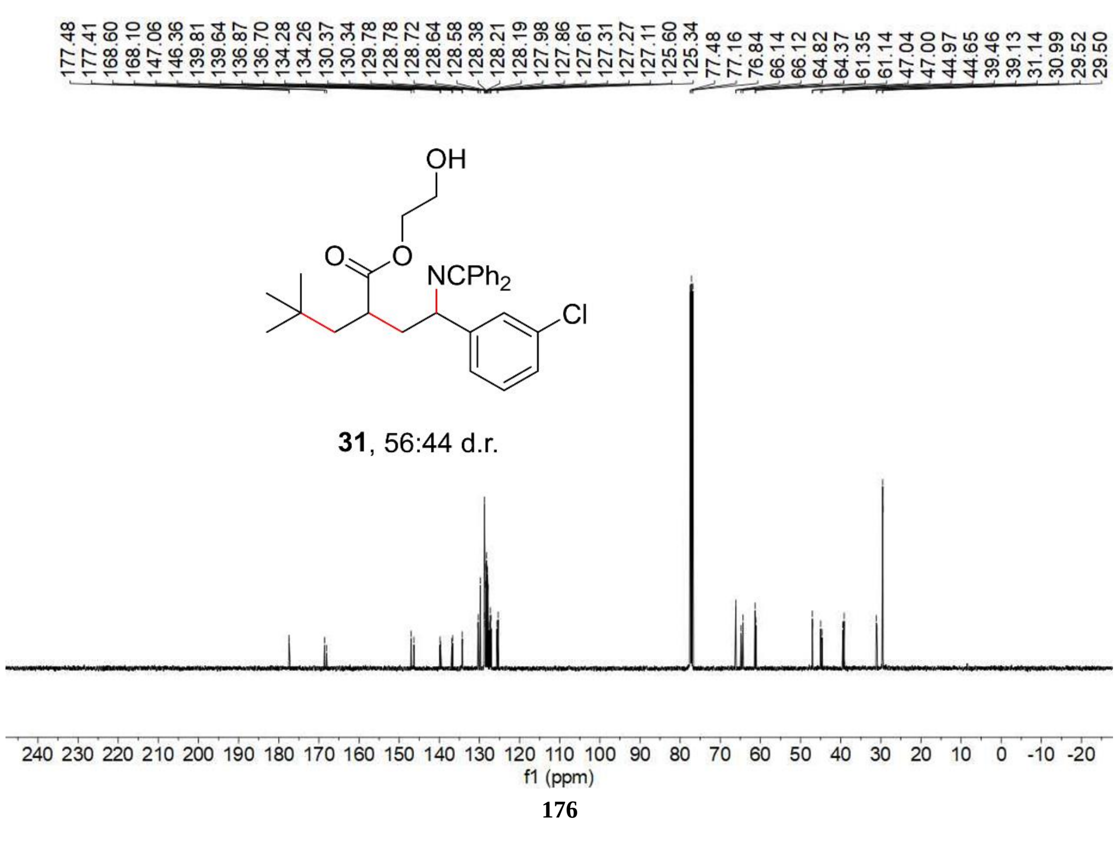
177.48 177.41 168.60 168.10 147.06 146.36 139.81 139.64 136.87 136.70 134.28 134.26 130.37 130.34 129.78 128.78 128.72 128.64 128.58 128.38 128.21 128.19 127.98 127.86 127.61 127.31 127.27 127.11 125.60 125.34 77.48 77.16 76.84 66.14 66.12 64.82 64.37 61.35 61.14 47.04 47.00 44.97 44.65 39.46 39.13 31.14 30.99 29.52 29.50
OH
O
O
NCPh2
Cl
31, 56:44 d.r.
f1 (ppm)

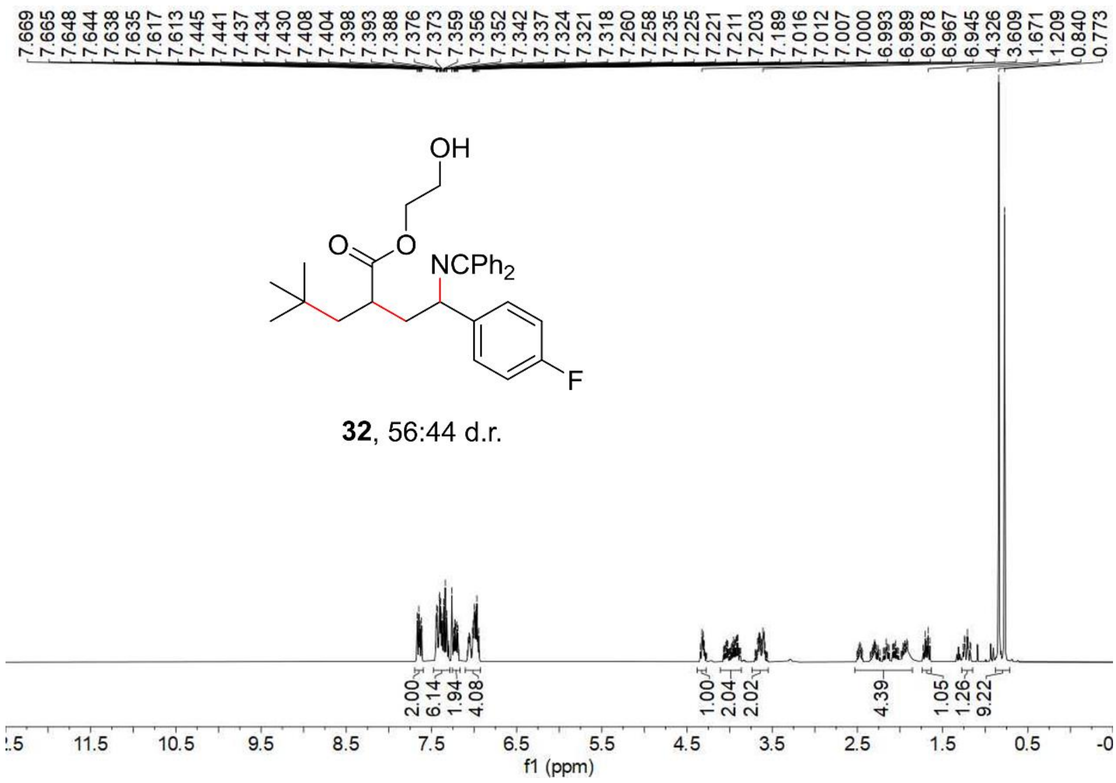
OH
NCPh2
F
32, 56:44 d.r.
f1 (ppm)

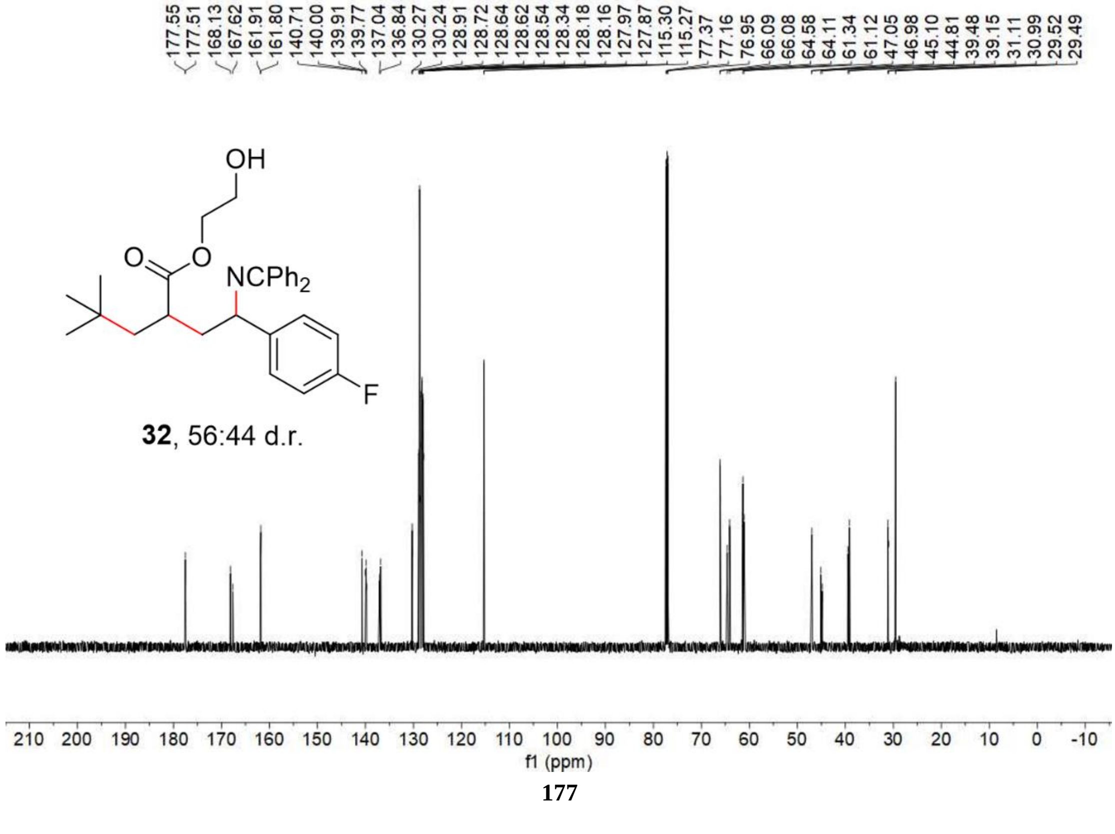
OH
NCPh2
F
32, 56:44 d.r.
f1 (ppm)

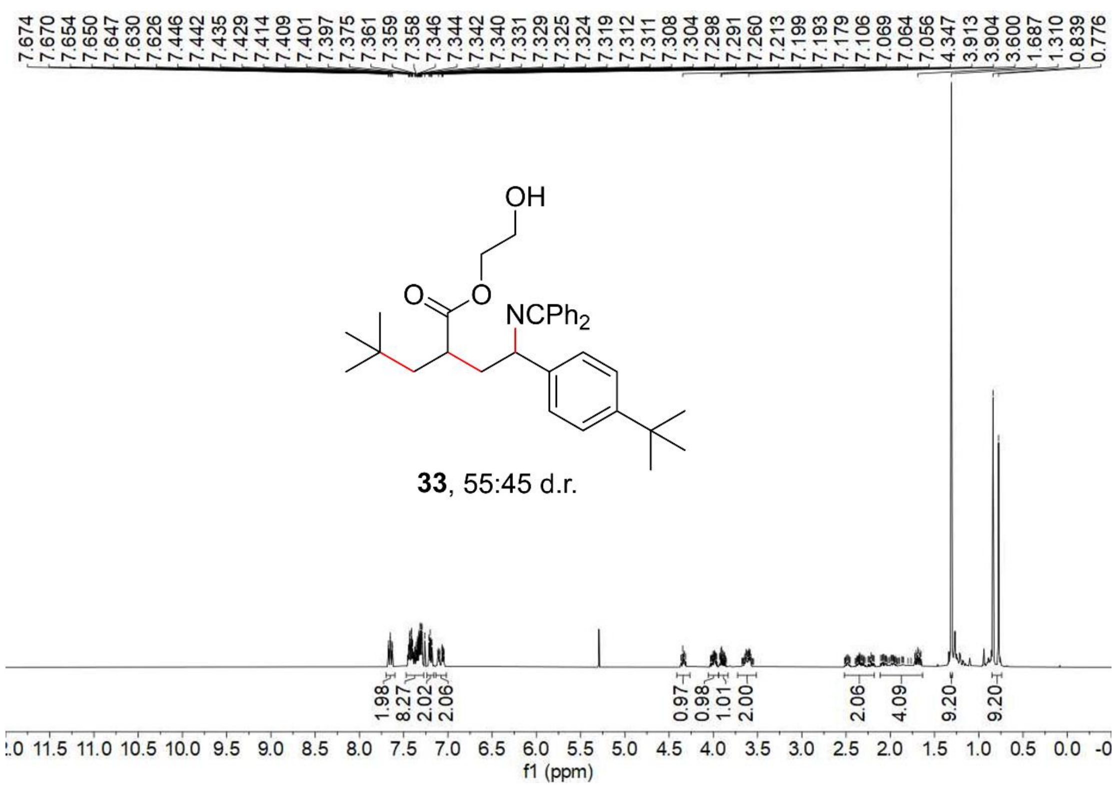

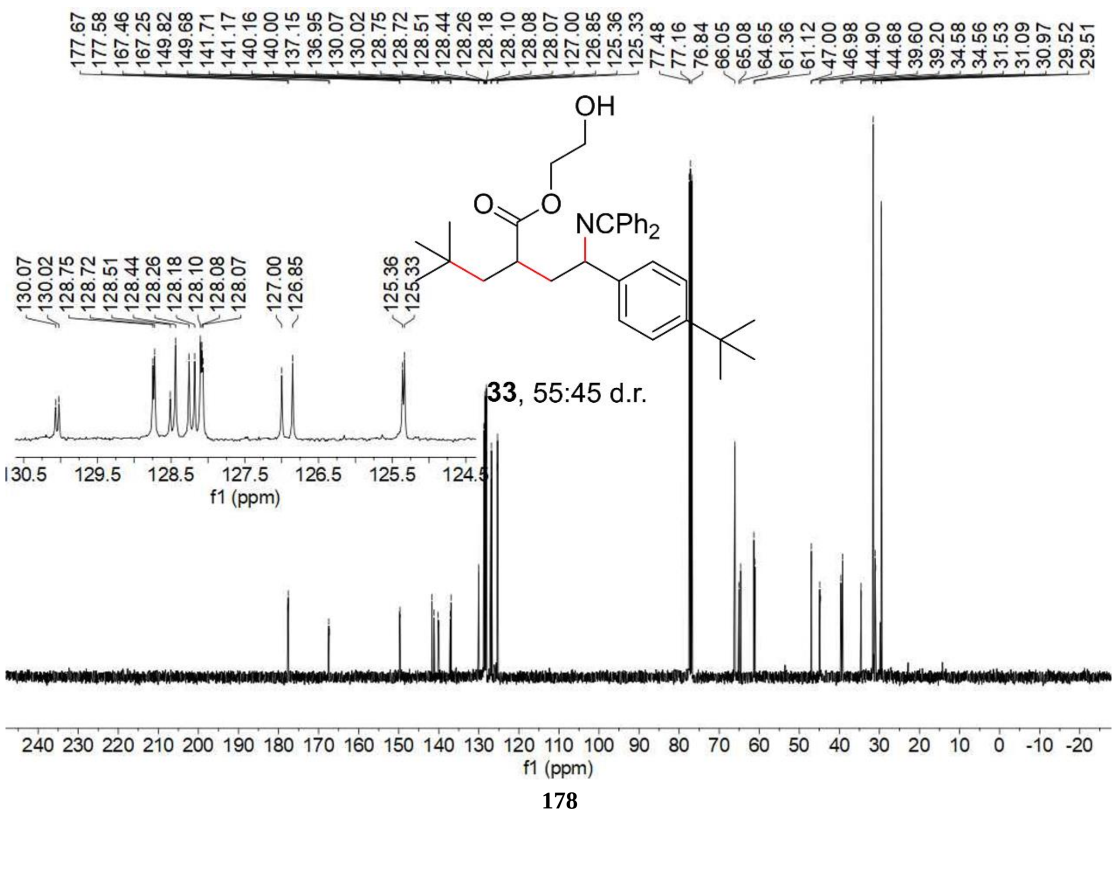

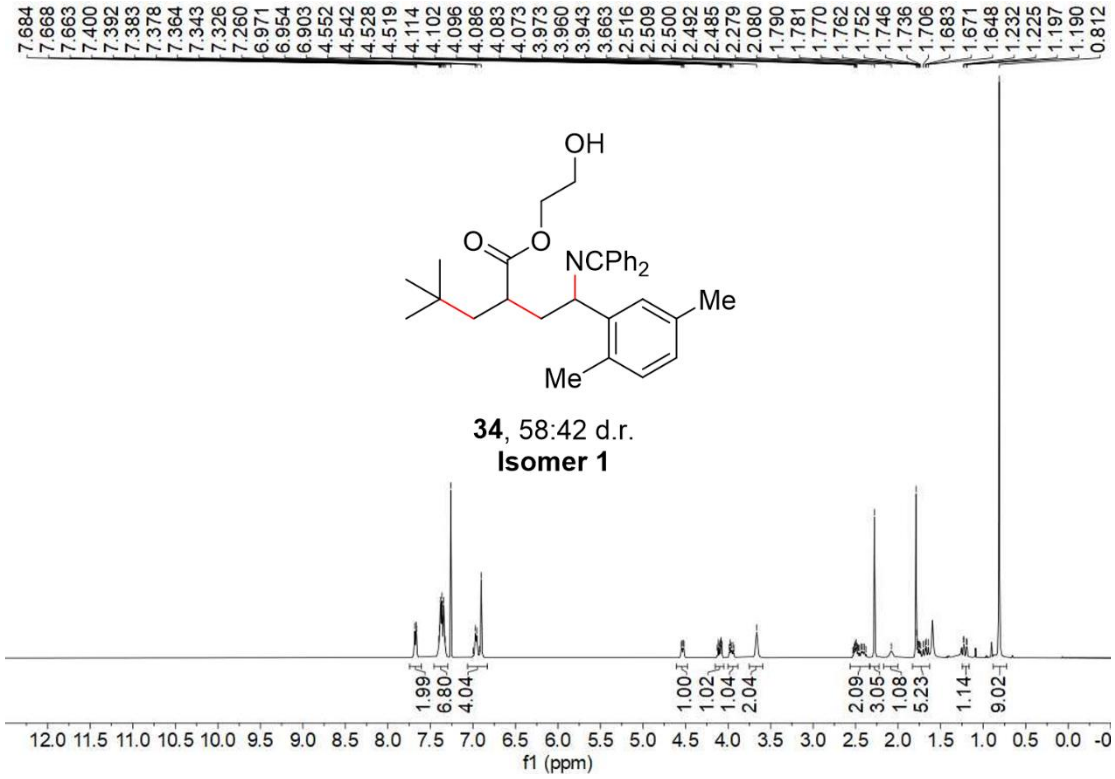

7.684
7.668
7.663
7.400
7.392
7.383
7.378
7.364
7.343
7.326
7.260
6.971
6.954
6.903
4.552
4.542
4.528
4.519
4.114
4.102
4.096
4.086
4.083
4.073
3.973
3.960
3.943
3.663
2.516
2.509
2.500
2.492
2.485
2.279
2.080
1.790
1.781
1.770
1.762
1.752
1.746
1.736
1.706
1.683
1.671
1.648
1.232
1.225
1.197
1.190
0.812
OH
O
O
NCPh2
Me
Me
34, 58:42 d.r.
Isomer 1
1.99
6.80
4.04
1.00
1.02
1.04
2.04
2.09
3.05
1.08
5.23
1.14
9.02
f1 (ppm)

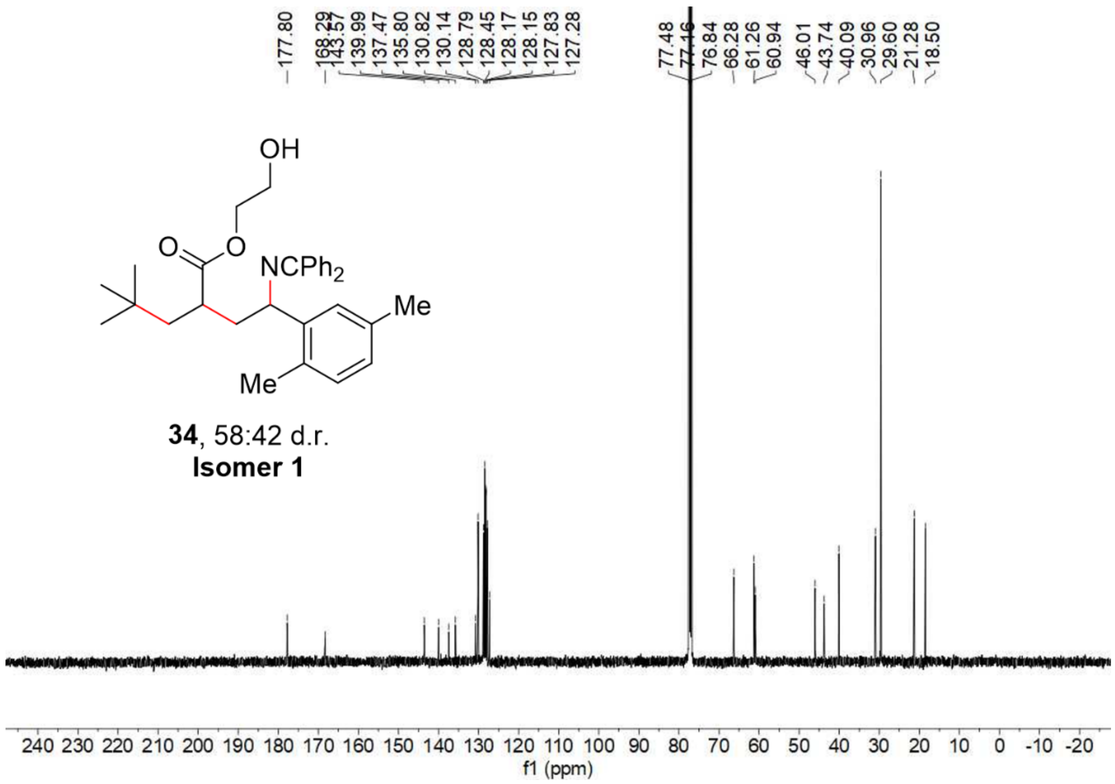

177.80
168.29
143.57
139.99
137.47
135.80
130.82
130.14
128.79
128.45
128.17
128.15
127.83
127.28
77.48
77.16
76.84
66.28
61.26
60.94
46.01
43.74
40.09
30.96
29.60
21.28
18.50
OH
O
O
NCPh2
Me
Me
34, 58:42 d.r.
Isomer 1
f1 (ppm)

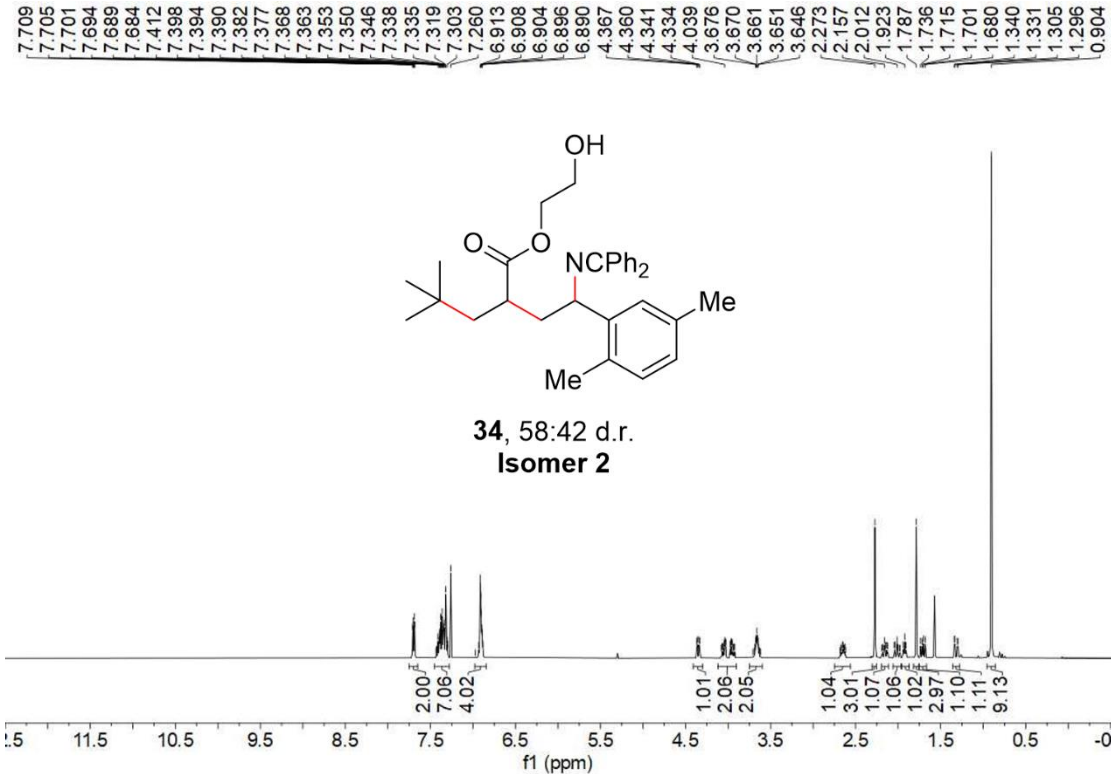
7.709 7.705 7.701 7.694 7.689 7.684 7.412 7.398 7.394 7.390 7.382 7.377 7.368 7.363 7.353 7.350 7.346 7.338 7.335 7.319 7.303 7.260 6.913 6.908 6.904 6.896 6.890 4.367 4.360 4.341 4.334 4.039 3.676 3.670 3.661 3.651 3.646 2.273 2.157 2.012 1.923 1.787 1.736 1.715 1.701 1.680 1.340 1.331 1.305 1.296 0.904
OH
O
O
NCPh2
Me
Me
34, 58:42 d.r.
Isomer 2
2.00 7.06 4.02 1.01 2.06 2.05 1.04 3.01 1.07 1.06 1.02 2.97 1.10 1.11 9.13
11.5 10.5 9.5 8.5 7.5 6.5 5.5 4.5 3.5 2.5 1.5 0.5
f1 (ppm)

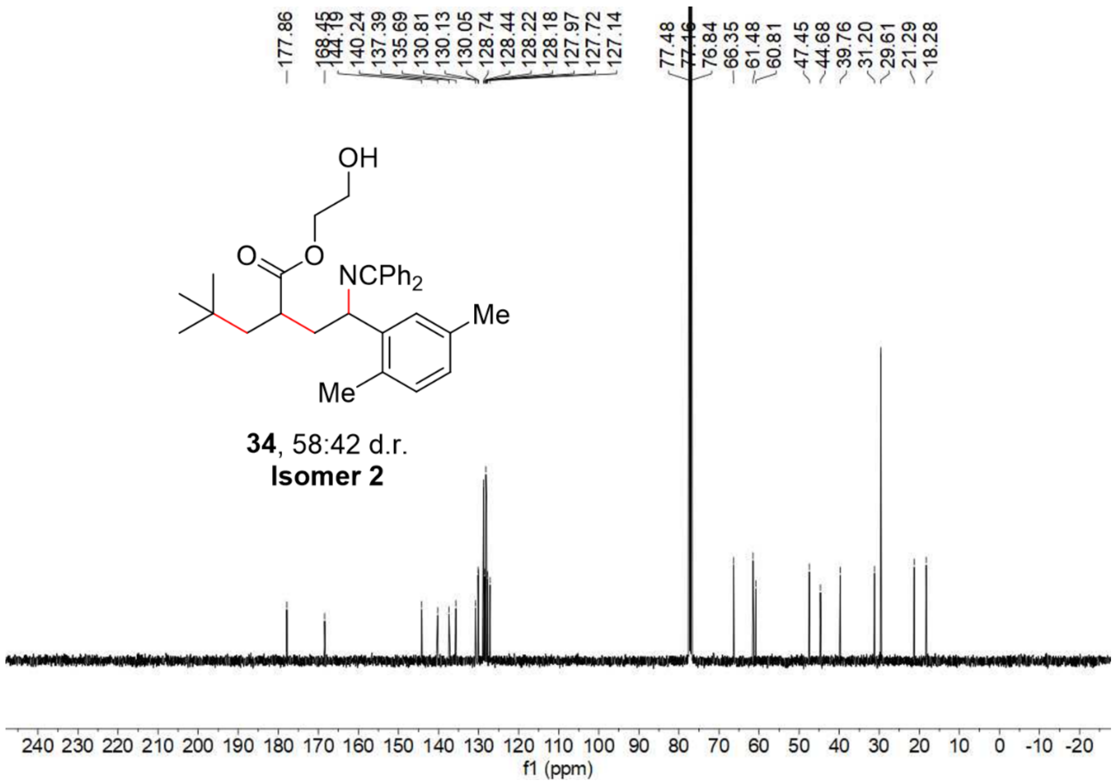
177.86 168.45 144.19 140.24 137.39 135.69 130.81 130.13 130.05 128.74 128.44 128.22 128.18 127.97 127.72 127.14 77.48 77.16 76.84 66.35 61.48 60.81 47.45 44.68 39.76 31.20 29.61 21.29 18.28
OH
O
O
NCPh2
Me
Me
34, 58:42 d.r.
Isomer 2
240 230 220 210 200 190 180 170 160 150 140 130 120 110 100 90 80 70 60 50 40 30 20 10 0 -10 -20
f1 (ppm)

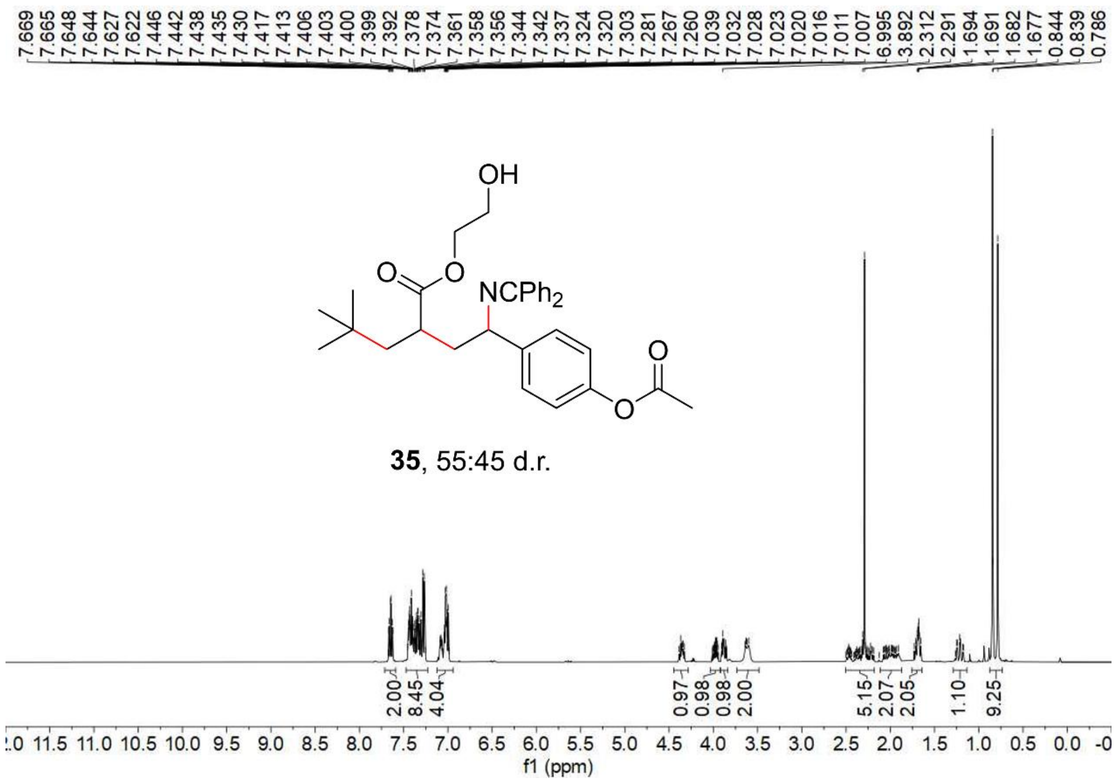
7.669 7.665 7.648 7.644 7.627 7.622 7.446 7.442 7.438 7.435 7.430 7.417 7.413 7.406 7.403 7.400 7.399 7.392 7.378 7.374 7.361 7.358 7.356 7.344 7.342 7.337 7.324 7.320 7.303 7.281 7.267 7.260 7.039 7.032 7.028 7.023 7.020 7.016 7.011 7.007 6.995 3.892 2.312 2.291 1.694 1.691 1.682 1.677 0.844 0.839 0.786
OH
O
O
NCPh2
O
O
35, 55:45 d.r.
2.00 8.45 4.04 0.97 0.98 0.98 2.00 5.15 2.07 2.05 1.10 9.25
f1 (ppm)

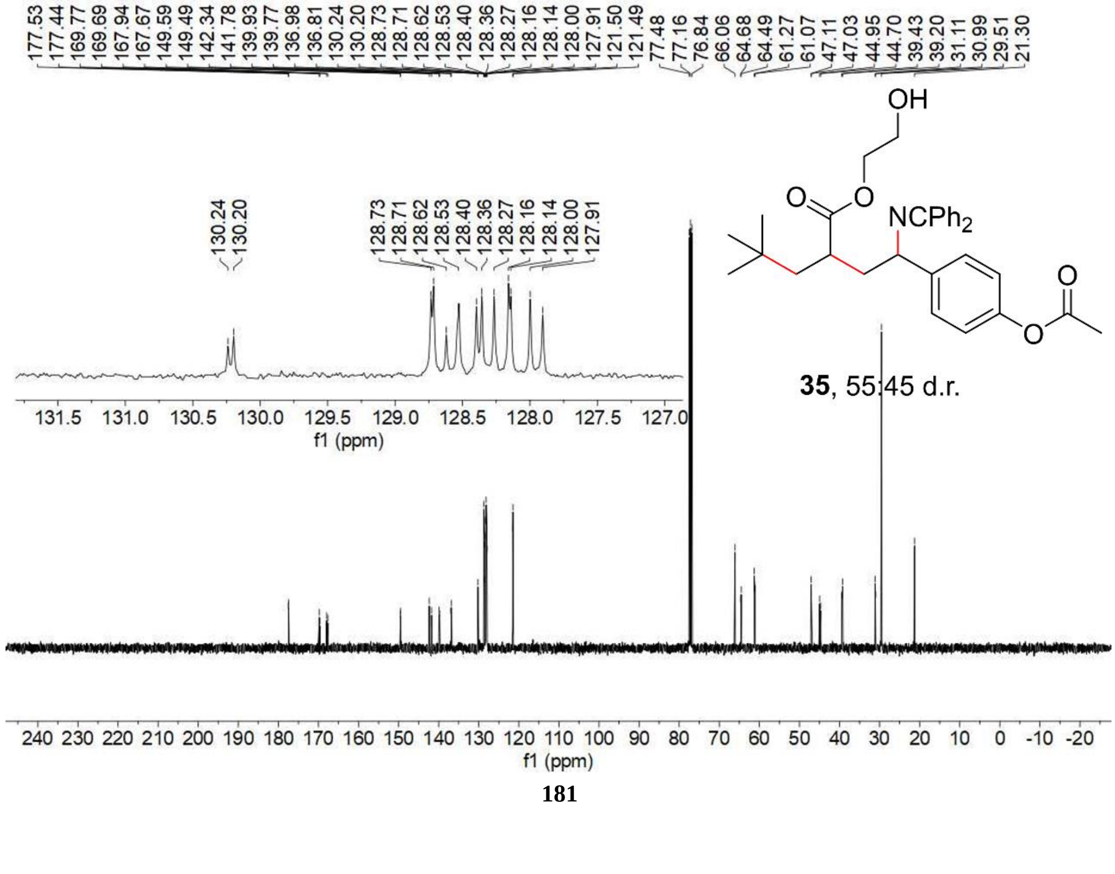
177.53 177.44 169.77 169.69 167.94 167.67 149.59 149.49 142.34 141.78 139.93 139.77 136.98 136.81 130.24 130.20 128.73 128.71 128.62 128.53 128.40 128.36 128.27 128.16 128.14 128.00 127.91 121.50 121.49 77.48 77.16 76.84 66.06 64.68 64.49 61.27 61.07 47.11 47.03 44.95 44.70 39.43 39.20 31.11 30.99 29.51 21.30
130.24 130.20
128.73 128.71 128.62 128.53 128.40 128.36 128.27 128.16 128.14 128.00 127.91
f1 (ppm)
OH
O
O
NCPh2
O
O
35, 55:45 d.r.
f1 (ppm)

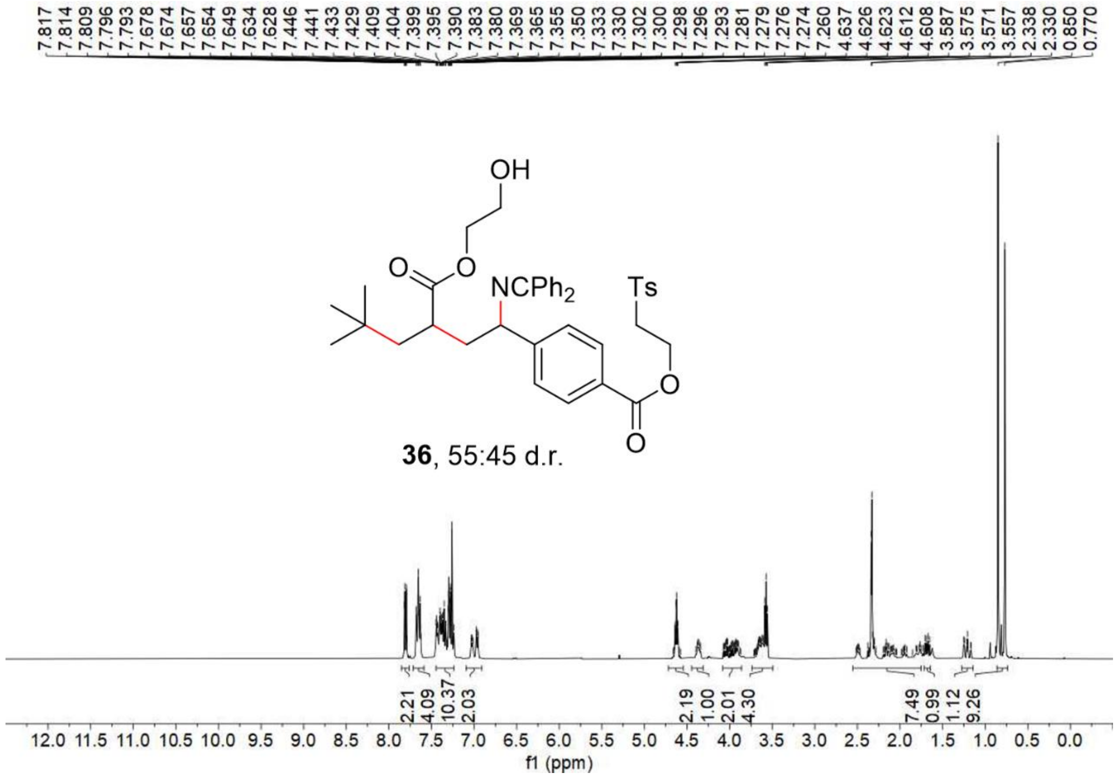

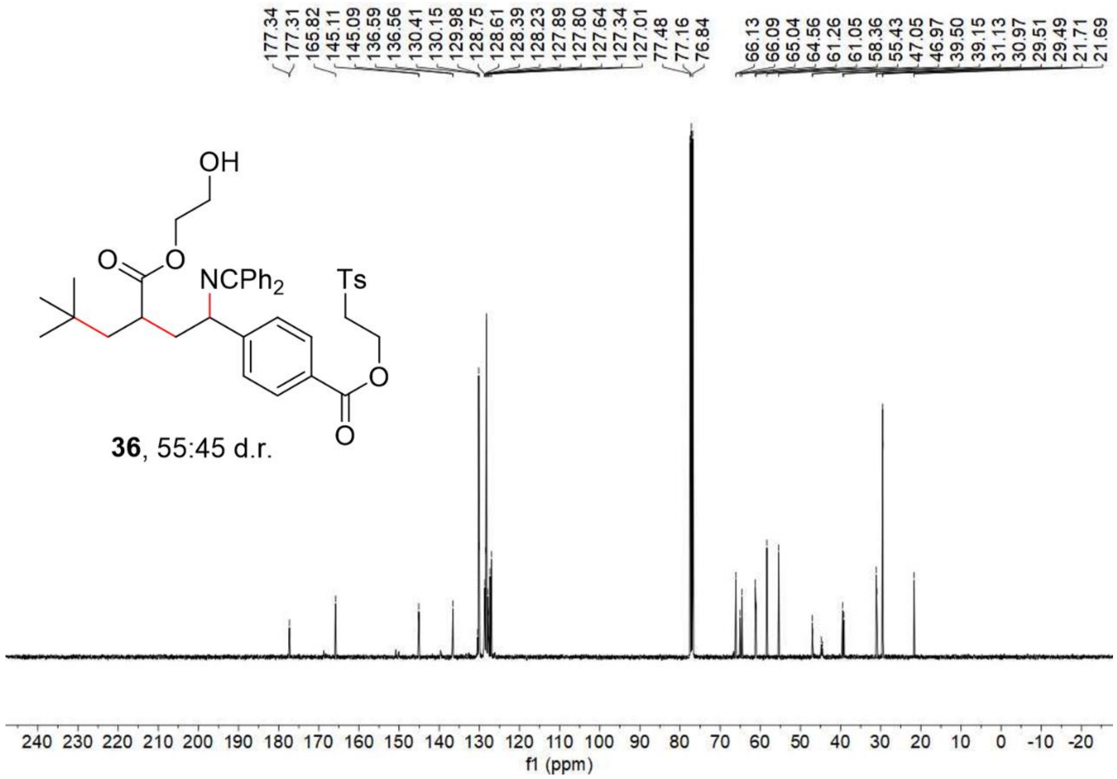

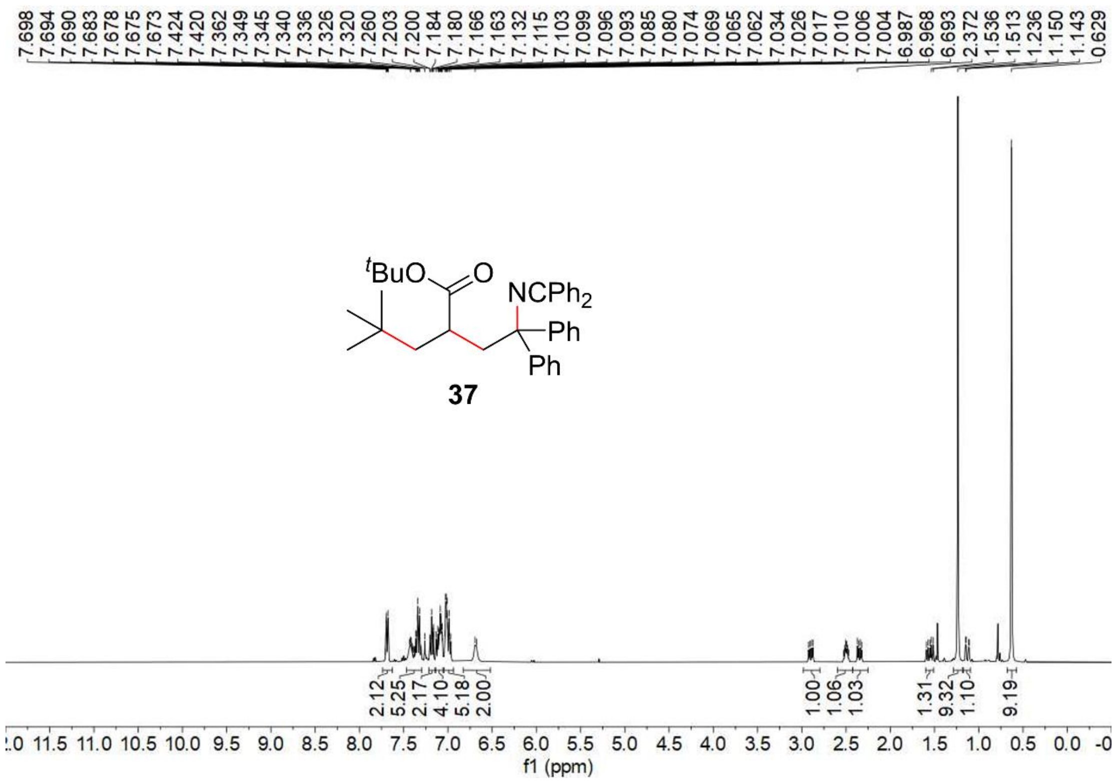

^{t}BuO
O
NCPh$_2$
Ph
Ph
37
f1 (ppm)

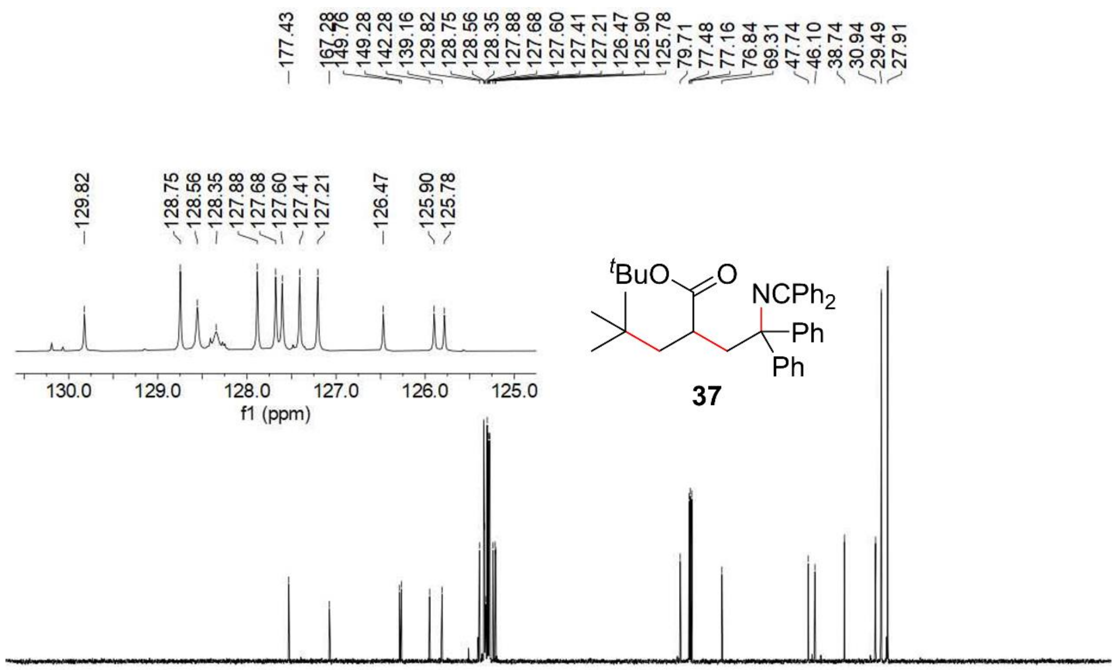

^{t}BuO
O
NCPh$_2$
Ph
Ph
37
f1 (ppm)

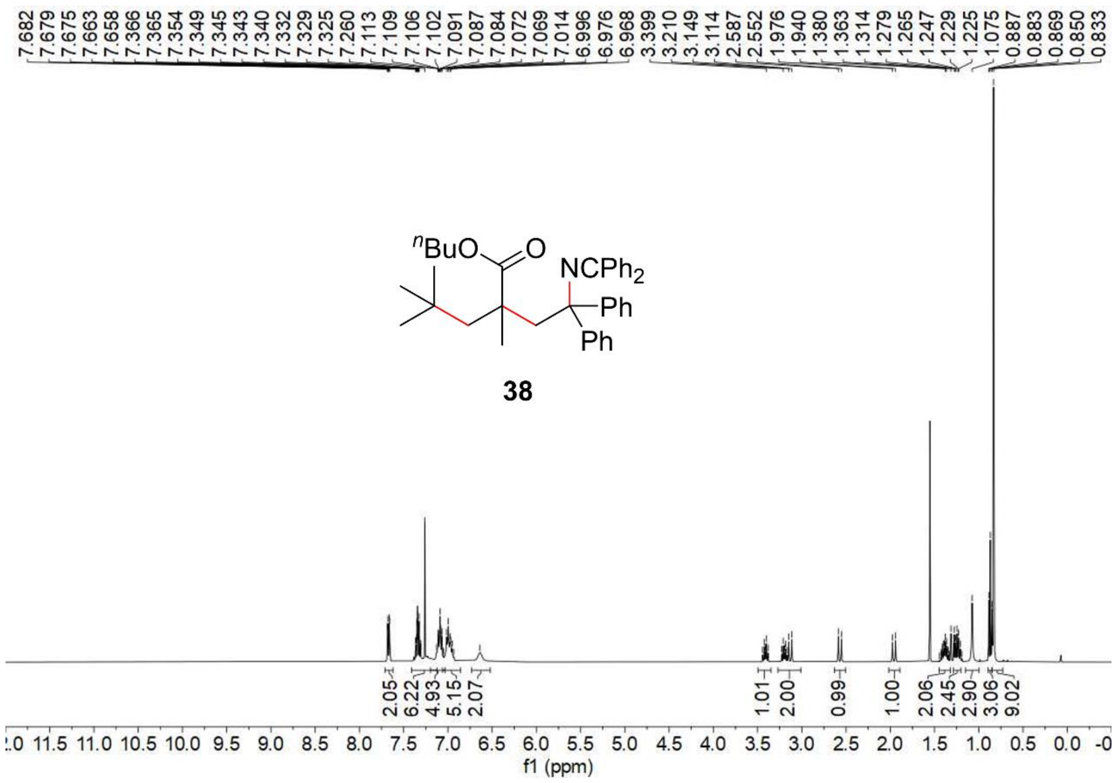
7.682 7.679 7.675 7.663 7.658 7.366 7.365 7.354 7.349 7.345 7.343 7.340 7.332 7.329 7.325 7.260 7.113 7.109 7.106 7.102 7.091 7.087 7.084 7.072 7.069 7.014 6.996 6.976 6.968 3.399 3.210 3.149 3.114 2.587 2.552 1.976 1.940 1.380 1.363 1.314 1.279 1.265 1.247 1.229 1.225 1.075 0.887 0.883 0.869 0.850 0.833
^{n}BuO
O
NCPh$_2$
Ph
Ph
2.05 6.22 4.93 5.15 2.07 1.01 2.00 0.99 1.00 2.06 2.45 2.90 3.06 9.02
f1 (ppm)

38

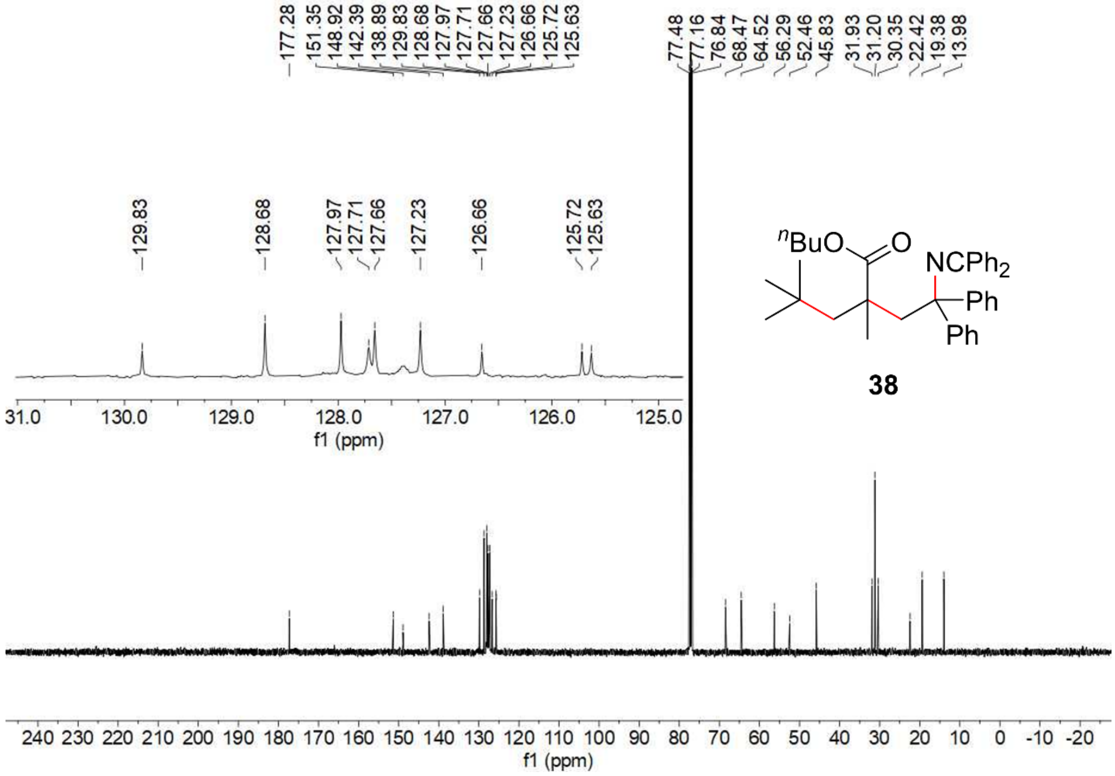
177.28 151.35 148.92 142.39 138.89 129.83 128.68 127.97 127.71 127.66 127.23 126.66 125.72 125.63
77.48 77.16 76.84 68.47 64.52 56.29 52.46 45.83 31.93 31.20 30.35 22.42 19.38 13.98
129.83 128.68 127.97 127.71 127.66 127.23 126.66 125.72 125.63
f1 (ppm)
^{n}BuO
O
NCPh$_2$
Ph
Ph
f1 (ppm)

38

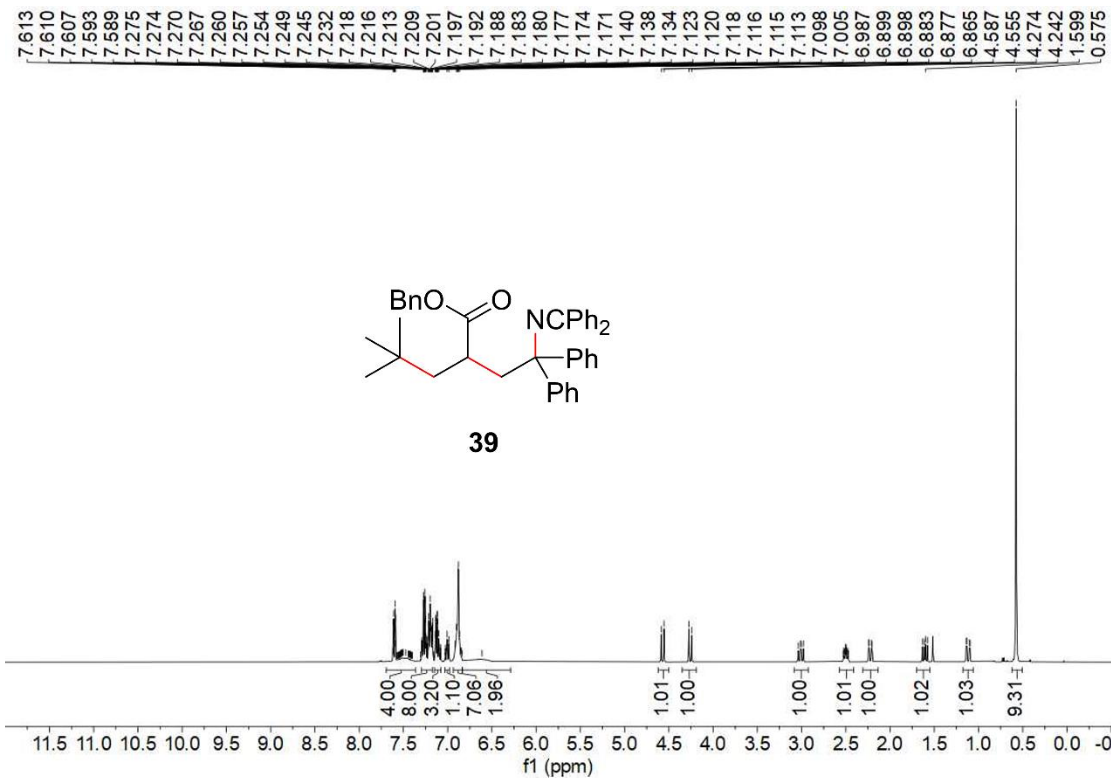

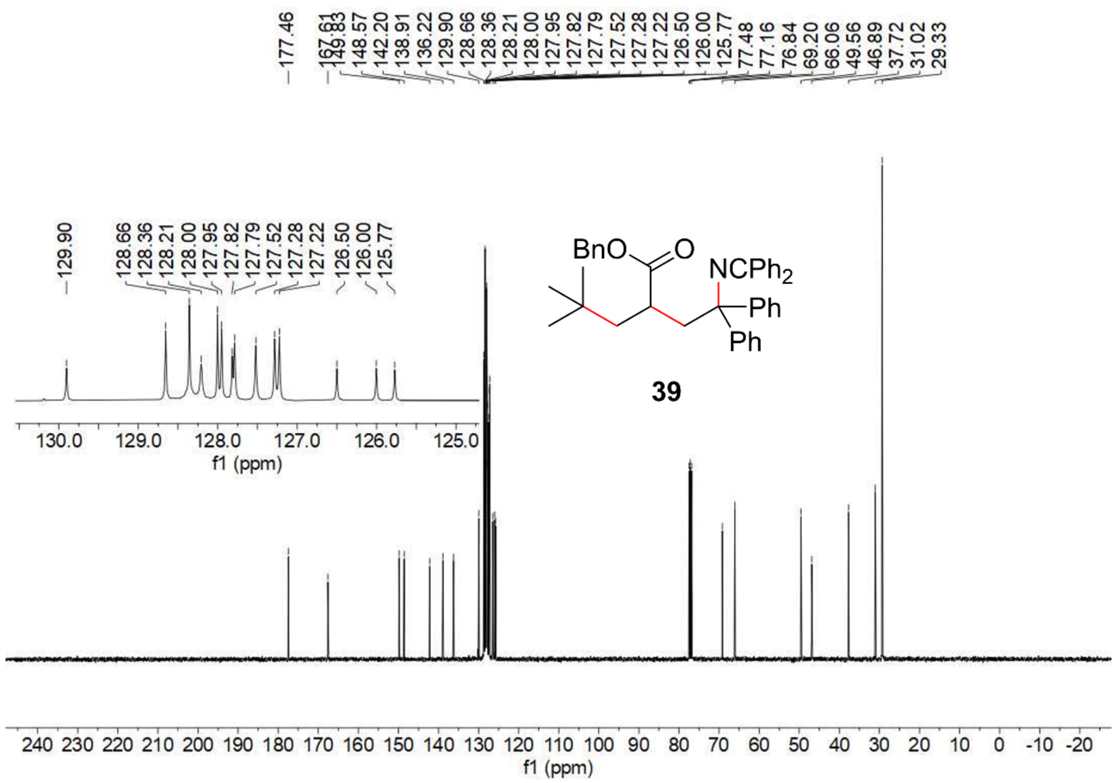

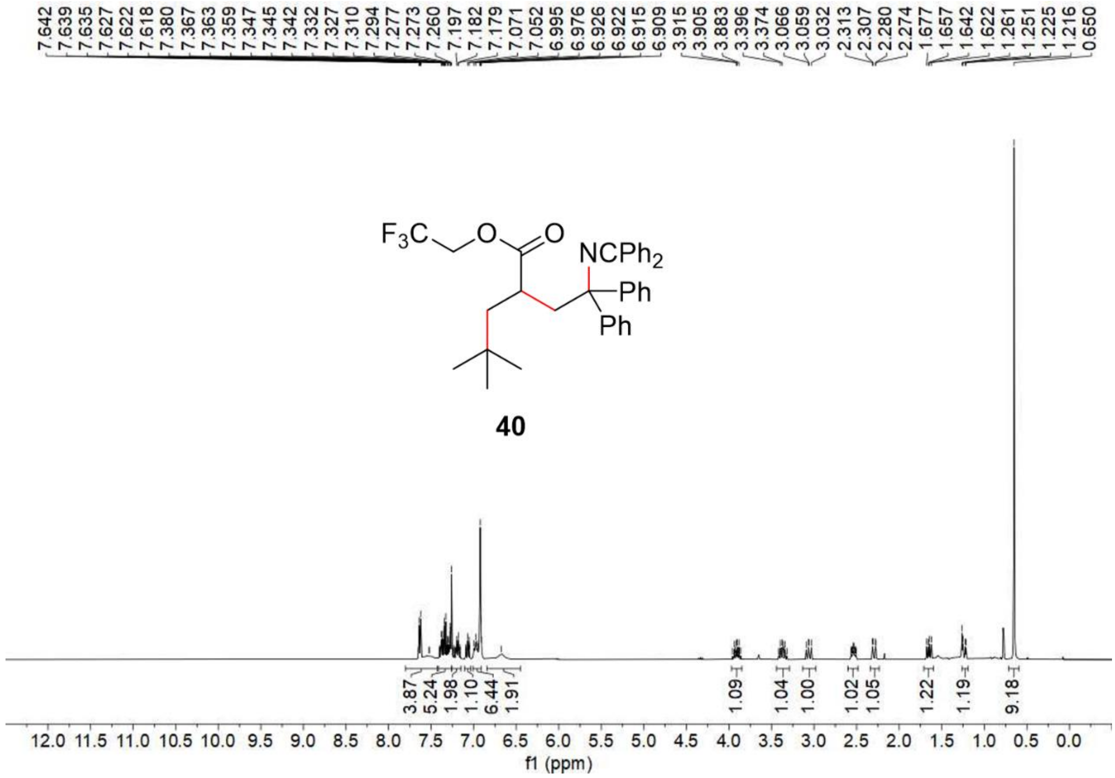
7.642 7.639 7.635 7.627 7.622 7.618 7.380 7.367 7.363 7.359 7.347 7.345 7.342 7.332 7.327 7.310 7.294 7.277 7.273 7.260 7.197 7.182 7.179 7.071 7.052 6.995 6.976 6.926 6.922 6.915 6.909 3.915 3.905 3.883 3.396 3.374 3.066 3.059 3.032 2.313 2.307 2.280 2.274 1.677 1.657 1.642 1.622 1.261 1.251 1.225 1.216 0.650
F3C O O NCPh2 Ph Ph
40
3.87 5.24 1.98 1.10 6.44 1.91 1.09 1.04 1.00 1.02 1.05 1.22 1.19 9.18
12.0 11.5 11.0 10.5 10.0 9.5 9.0 8.5 8.0 7.5 7.0 6.5 6.0 5.5 5.0 4.5 4.0 3.5 3.0 2.5 2.0 1.5 1.0 0.5 0.0
f1 (ppm)

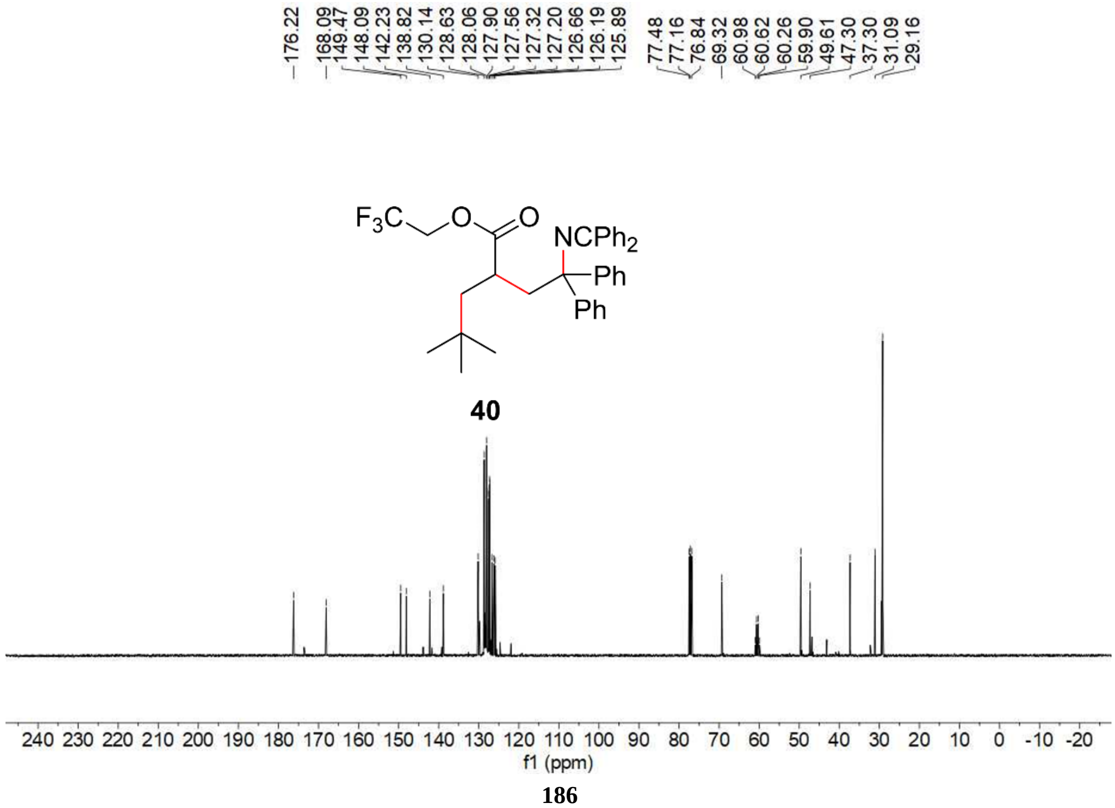
176.22 168.09 149.47 148.09 142.23 138.82 130.14 128.63 128.06 127.90 127.56 127.32 127.20 126.66 126.19 125.89 77.48 77.16 76.84 69.32 60.98 60.62 60.26 59.90 49.61 47.30 37.30 31.09 29.16
F3C O O NCPh2 Ph Ph
40
240 230 220 210 200 190 180 170 160 150 140 130 120 110 100 90 80 70 60 50 40 30 20 10 0 -10 -20
f1 (ppm)

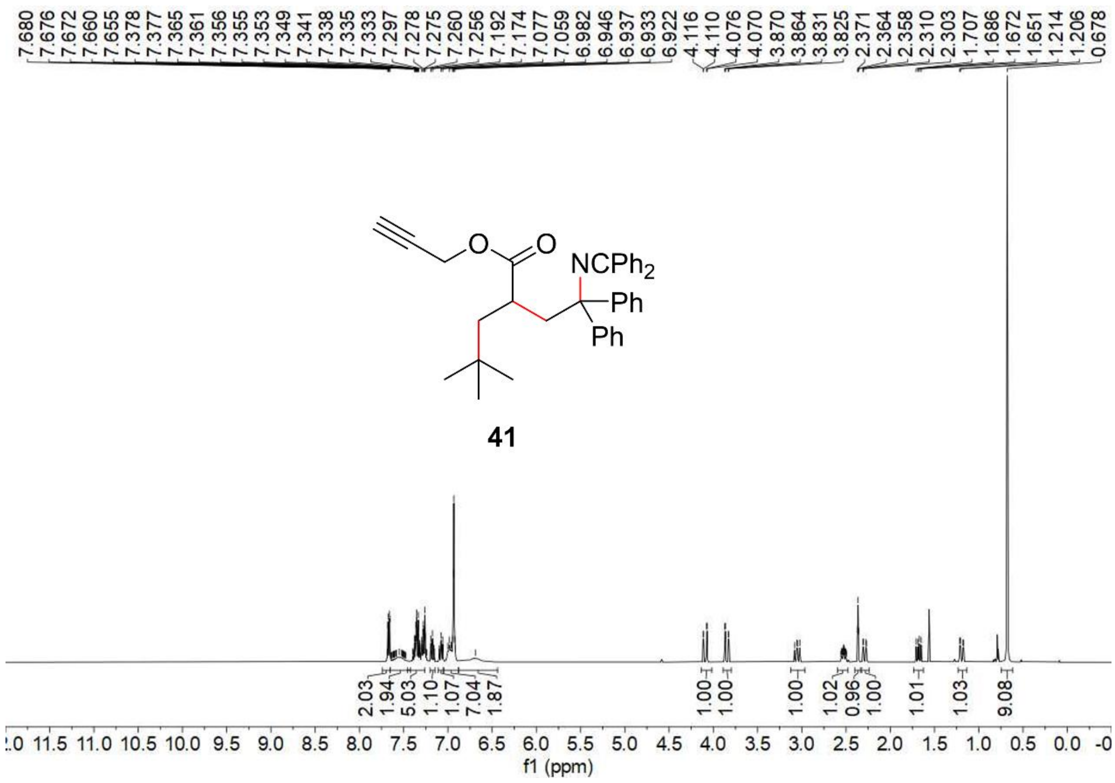

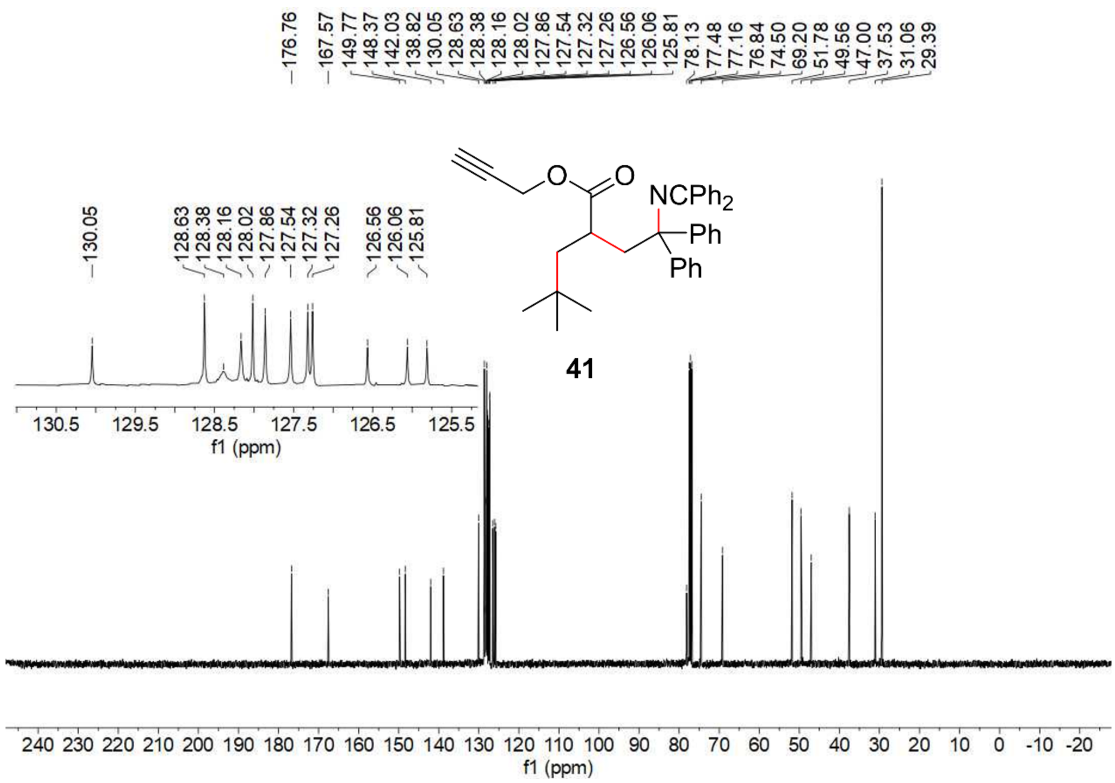

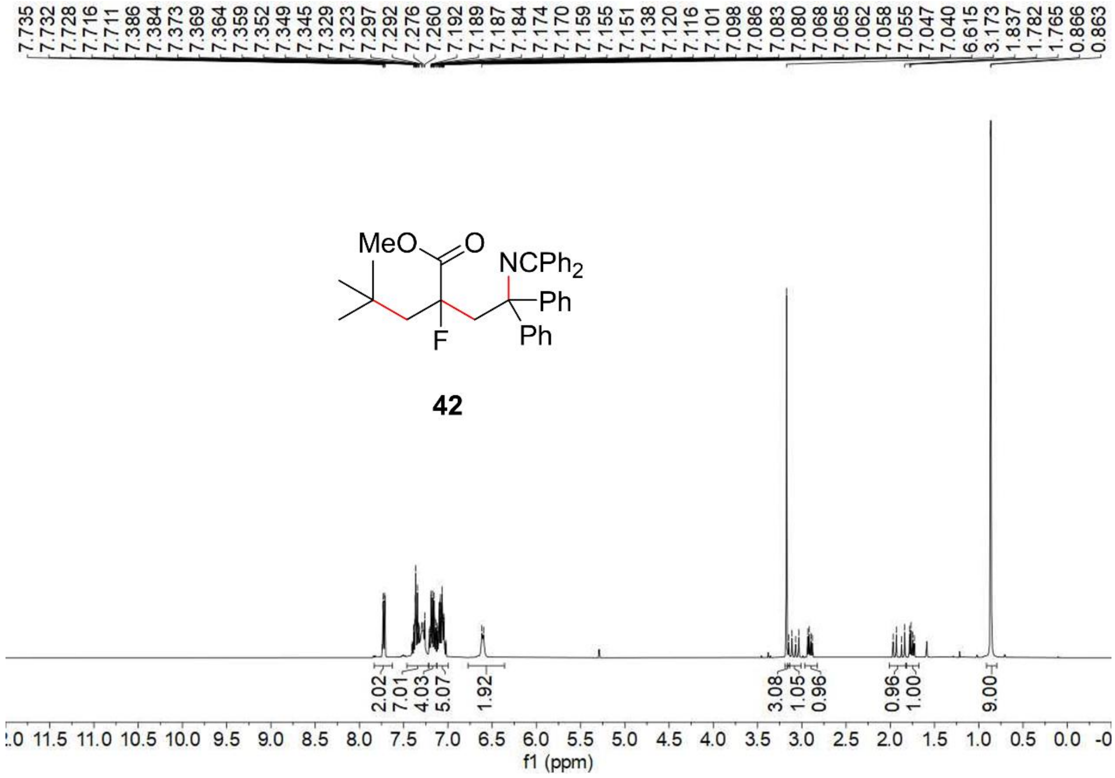
7.735 7.732 7.728 7.716 7.711 7.386 7.384 7.373 7.369 7.364 7.359 7.352 7.349 7.345 7.329 7.323 7.297 7.292 7.276 7.260 7.192 7.189 7.187 7.184 7.174 7.170 7.159 7.155 7.151 7.138 7.120 7.116 7.101 7.098 7.086 7.083 7.080 7.068 7.065 7.062 7.058 7.055 7.047 7.040 6.615 3.173 1.837 1.782 1.765 0.866 0.863
MeO O NCPh2 Ph Ph F
42
2.02 7.01 4.03 5.07 1.92 3.08 1.05 0.96 0.96 1.00 9.00
f1 (ppm)

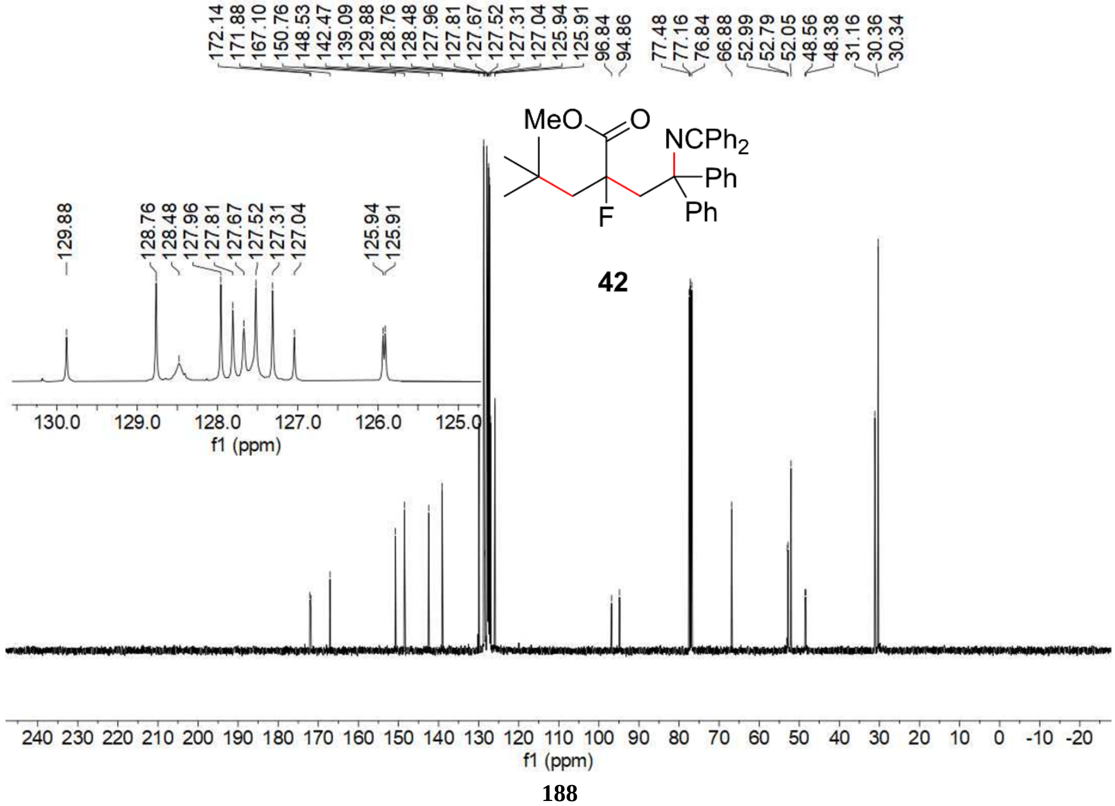
172.14 171.88 167.10 150.76 148.53 142.47 139.09 129.88 128.76 128.48 127.96 127.81 127.67 127.52 127.31 127.04 125.94 125.91 96.84 94.86 77.48 77.16 76.84 66.88 52.99 52.79 52.05 48.56 48.38 31.16 30.36 30.34
MeO O NCPh2 Ph Ph F
42
129.88 128.76 128.48 127.96 127.81 127.67 127.52 127.31 127.04 125.94 125.91
f1 (ppm)
f1 (ppm)

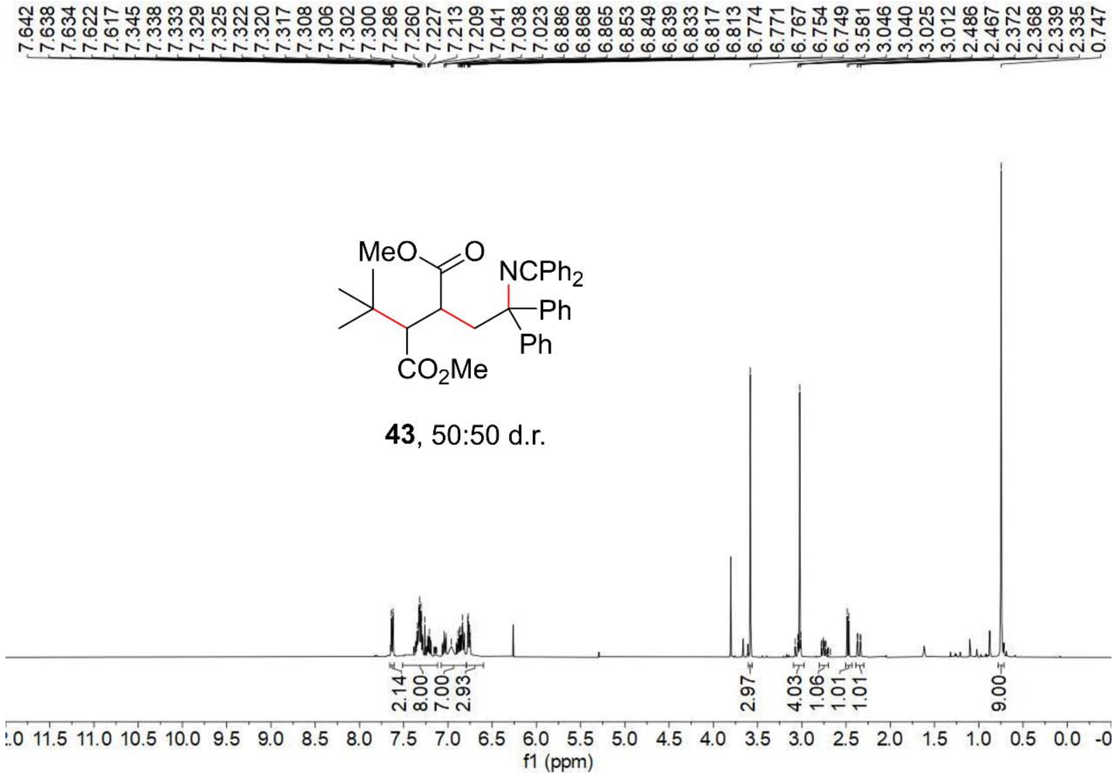
7.642 7.638 7.634 7.622 7.617 7.345 7.338 7.333 7.329 7.325 7.322 7.320 7.317 7.308 7.306 7.302 7.300 7.286 7.260 7.227 7.213 7.209 7.041 7.038 7.023 6.886 6.868 6.865 6.853 6.849 6.839 6.833 6.817 6.813 6.774 6.771 6.767 6.754 6.749 3.581 3.046 3.040 3.025 3.012 2.486 2.467 2.372 2.368 2.339 2.335 0.747
MeO
O
NCPh2
Ph
Ph
CO2Me
43, 50:50 d.r.
2.14
8.00
7.00
2.93
2.97
4.03
1.06
1.01
1.01
9.00
f1 (ppm)

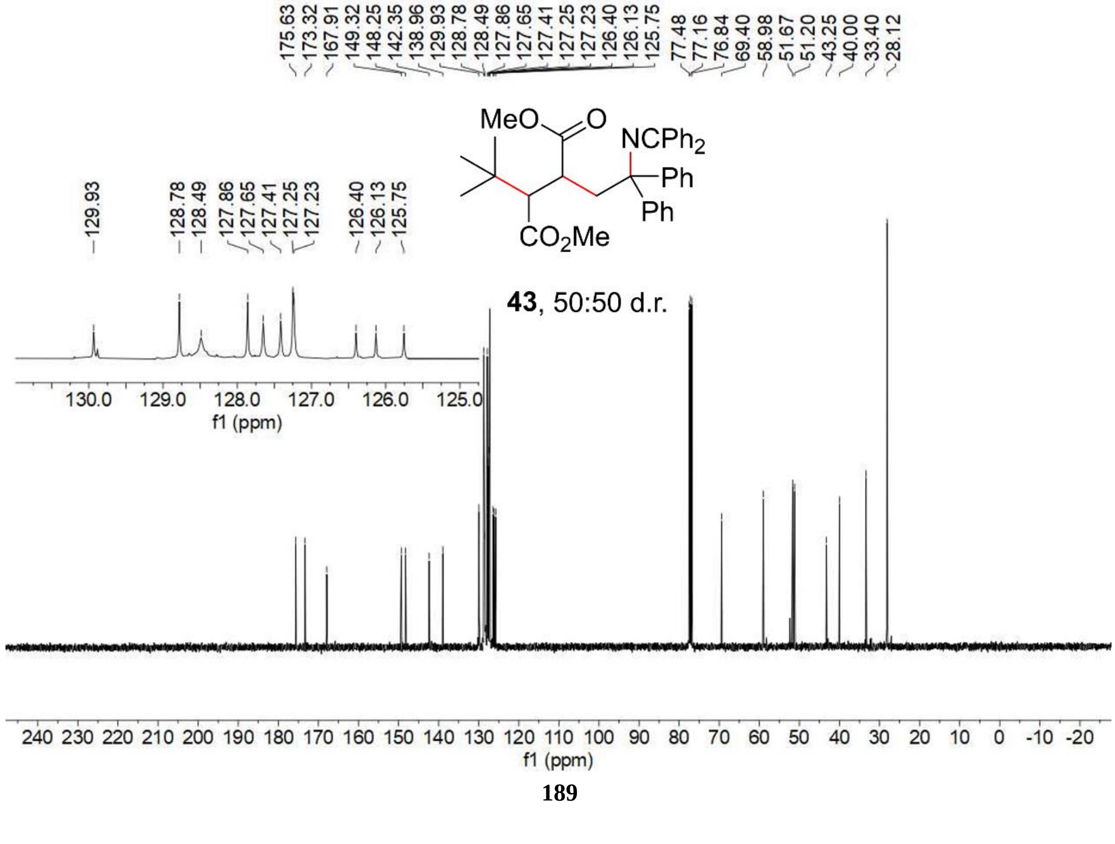
175.63 173.32 167.91 149.32 148.25 142.35 138.96 129.93 128.78 128.49 127.86 127.65 127.41 127.25 127.23 126.40 126.13 125.75 77.48 77.16 76.84 69.40 58.98 51.67 51.20 43.25 40.00 33.40 28.12
129.93
128.78
128.49
127.86
127.65
127.41
127.25
127.23
126.40
126.13
125.75
MeO
O
NCPh2
Ph
Ph
CO2Me
43, 50:50 d.r.
f1 (ppm)
f1 (ppm)

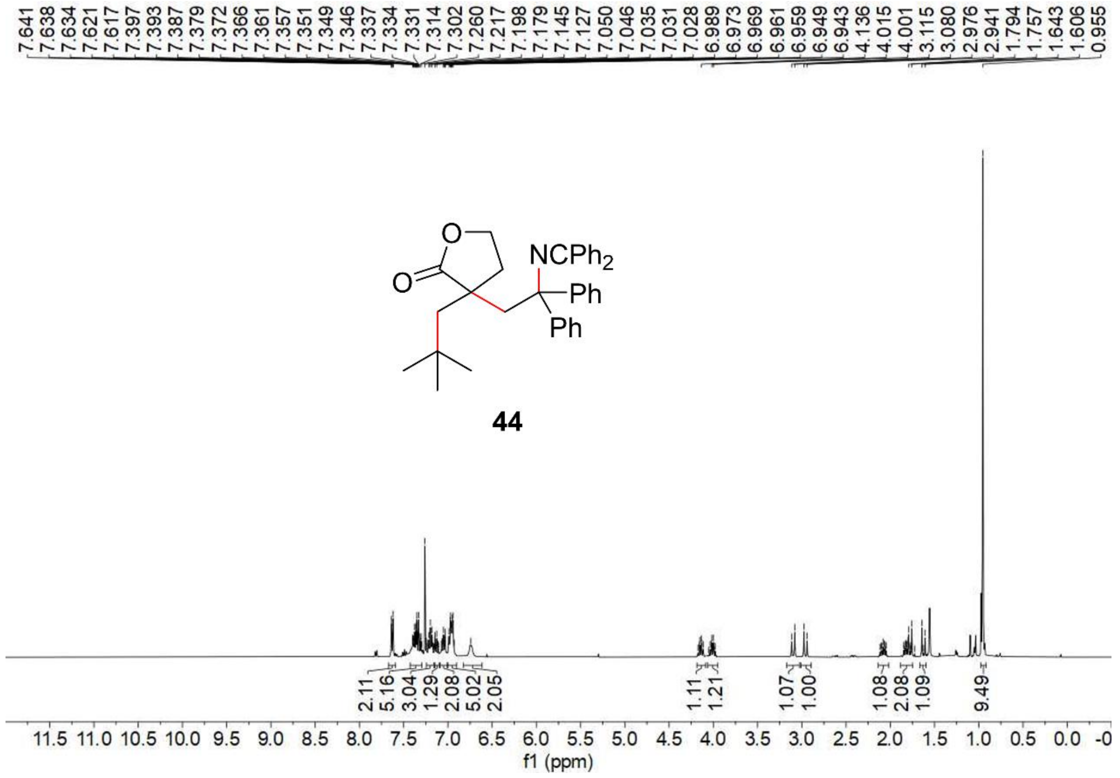
7.641 7.638 7.634 7.621 7.617 7.397 7.393 7.387 7.379 7.372 7.366 7.361 7.357 7.351 7.349 7.346 7.337 7.334 7.331 7.314 7.302 7.260 7.217 7.198 7.179 7.145 7.127 7.050 7.046 7.035 7.031 7.028 6.989 6.973 6.969 6.961 6.959 6.949 6.943 4.136 4.015 4.001 3.115 3.080 2.976 2.941 1.794 1.757 1.643 1.606 0.955
NCPh2
Ph
Ph
O
O
44
2.11 5.16 3.04 1.29 2.08 5.02 2.05 1.11 1.21 1.07 1.00 1.08 2.08 1.09 9.49
11.5 11.0 10.5 10.0 9.5 9.0 8.5 8.0 7.5 7.0 6.5 6.0 5.5 5.0 4.5 4.0 3.5 3.0 2.5 2.0 1.5 1.0 0.5 0.0 -0
f1 (ppm)

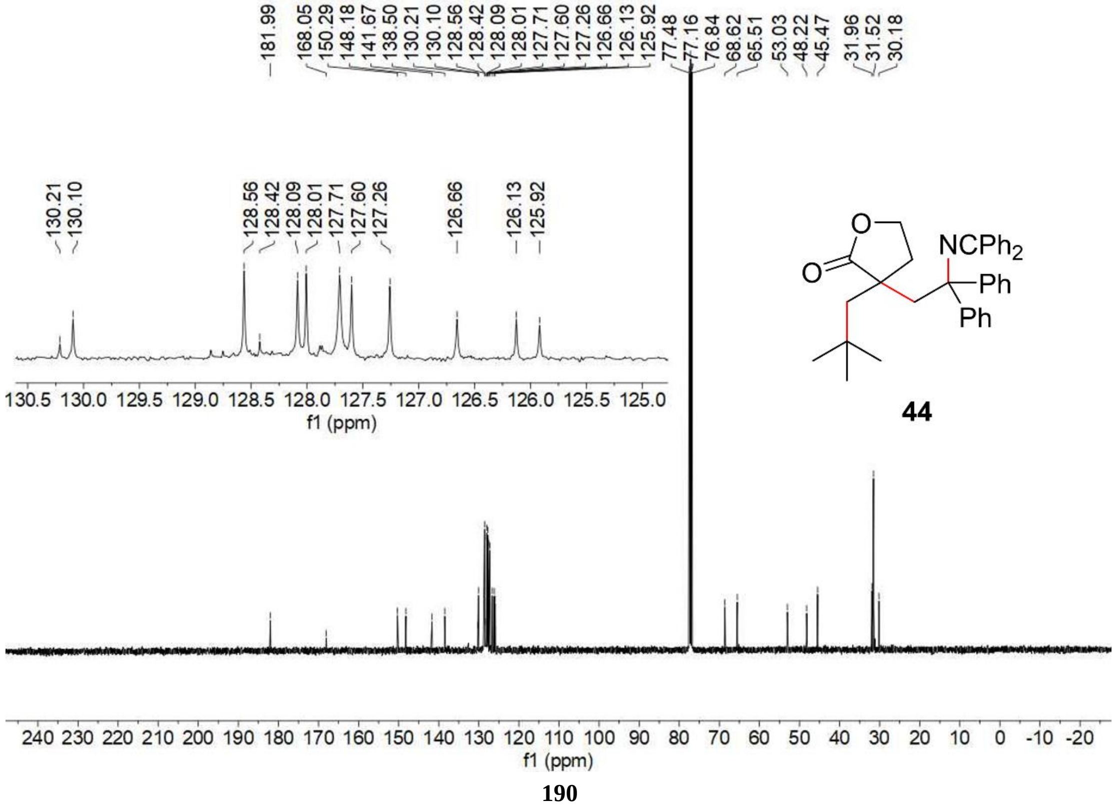
181.99 168.05 150.29 148.18 141.67 138.50 130.21 130.10 128.56 128.42 128.09 128.01 127.71 127.60 127.26 126.66 126.13 125.92 77.48 77.16 76.84 68.62 65.51 53.03 48.22 45.47 31.96 31.52 30.18
130.21 130.10 128.56 128.42 128.09 128.01 127.71 127.60 127.26 126.66 126.13 125.92
130.5 130.0 129.5 129.0 128.5 128.0 127.5 127.0 126.5 126.0 125.5 125.0
f1 (ppm)
NCPh2
Ph
Ph
O
O
44
240 230 220 210 200 190 180 170 160 150 140 130 120 110 100 90 80 70 60 50 40 30 20 10 0 -10 -20
f1 (ppm)

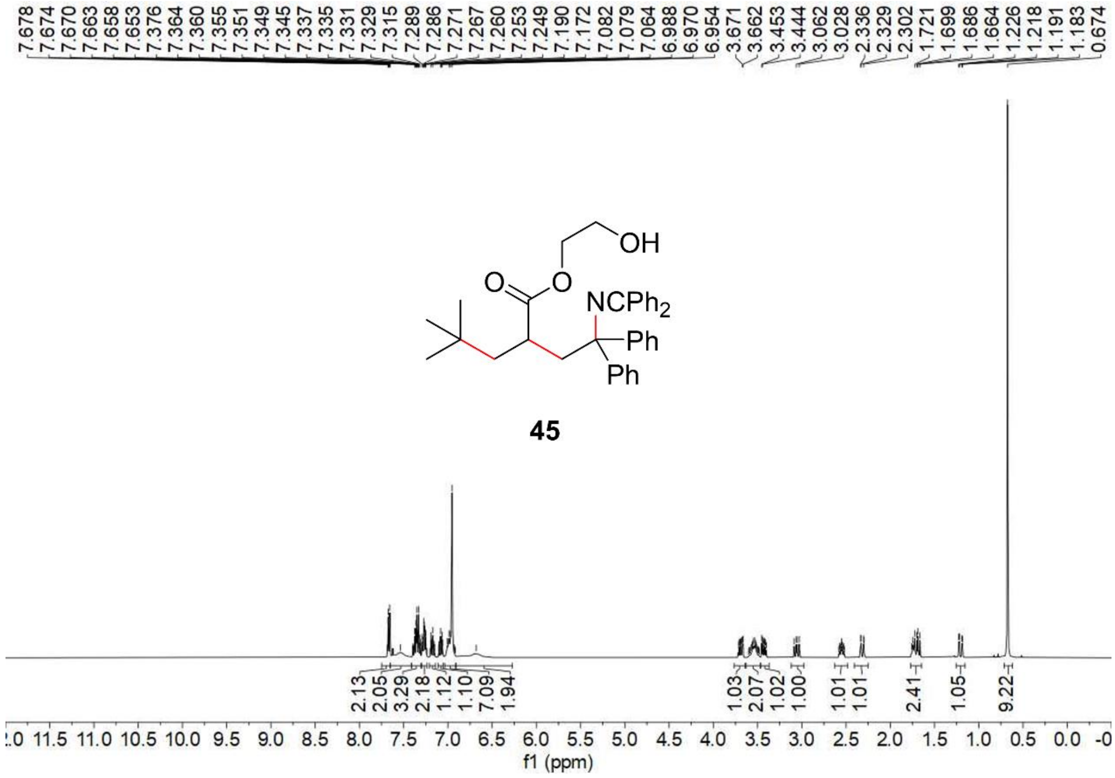

45

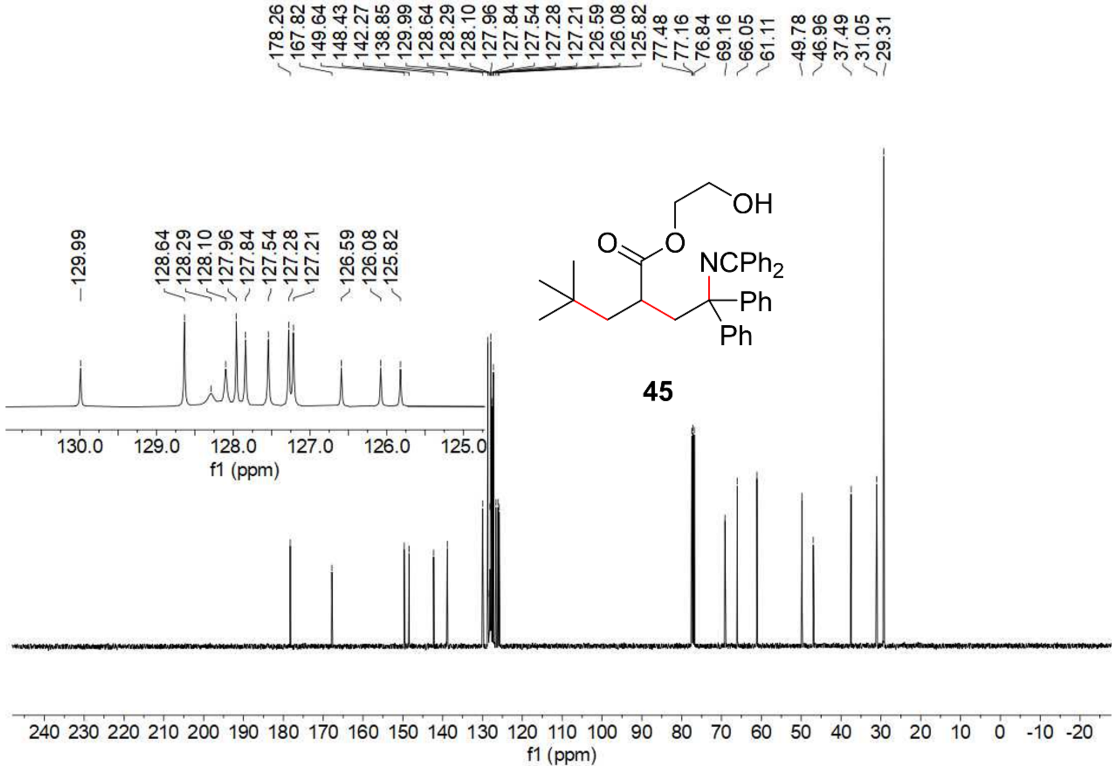

45

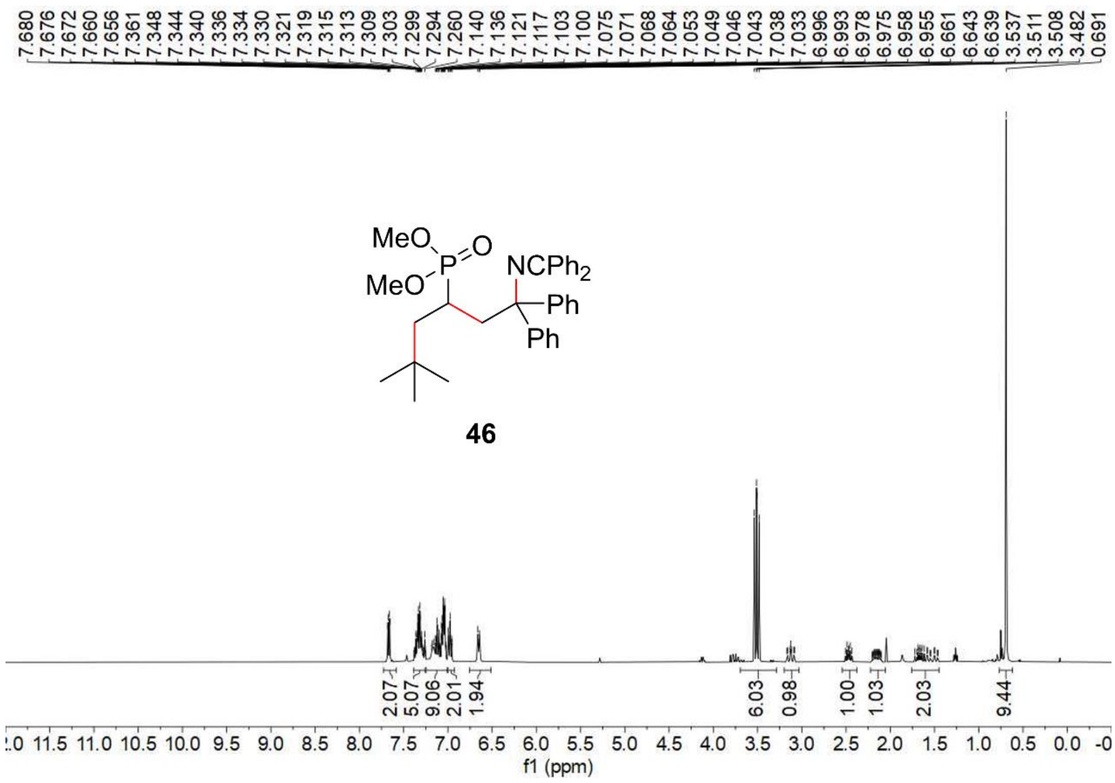
7.680 7.676 7.672 7.660 7.656 7.361 7.348 7.344 7.340 7.336 7.334 7.330 7.321 7.319 7.315 7.313 7.309 7.303 7.299 7.294 7.260 7.140 7.136 7.121 7.117 7.103 7.100 7.075 7.071 7.068 7.064 7.053 7.049 7.046 7.043 7.038 7.033 6.996 6.993 6.978 6.975 6.958 6.955 6.661 6.643 6.639 3.537 3.511 3.508 3.482 0.691
MeO
MeO
P
O
NCPh$_2$
Ph
Ph
46
2.07 5.07 9.06 2.01 1.94 6.03 0.98 1.00 1.03 2.03 9.44
f1 (ppm)

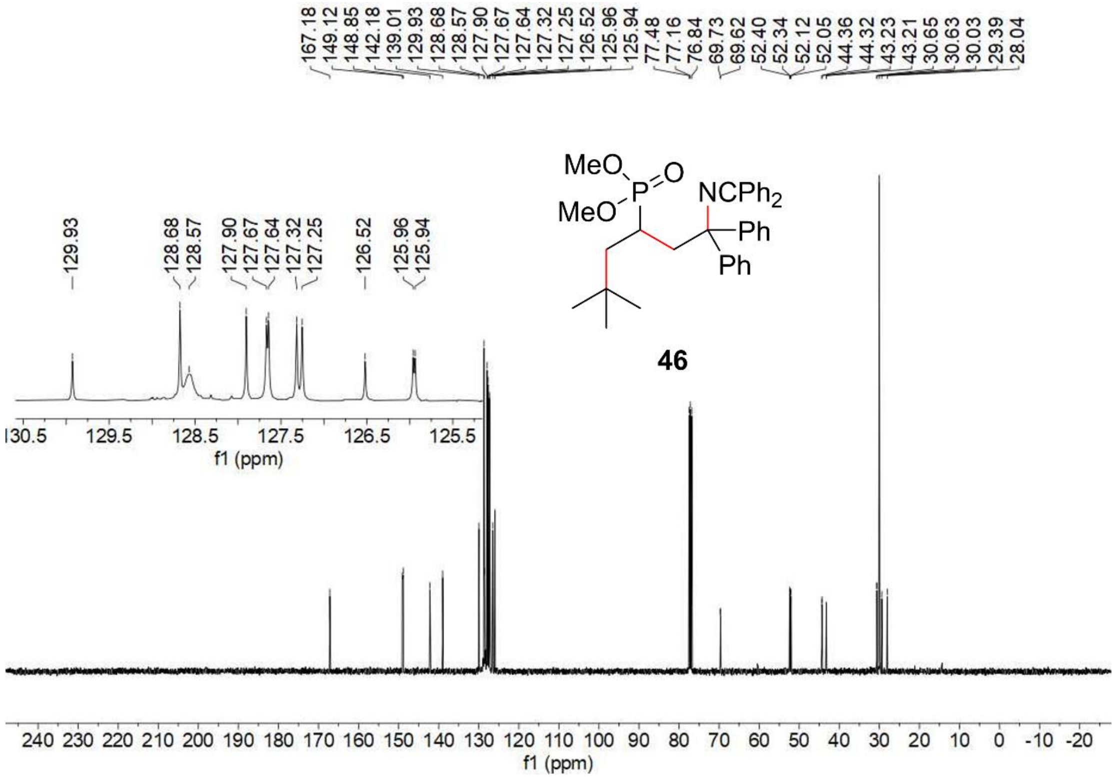
167.18 149.12 148.85 142.18 139.01 129.93 128.68 128.57 127.90 127.67 127.64 127.32 127.25 126.52 125.96 125.94 77.48 77.16 76.84 69.73 69.62 52.40 52.34 52.12 52.05 44.36 44.32 43.23 43.21 30.65 30.63 30.03 29.39 28.04
MeO
MeO
P
O
NCPh$_2$
Ph
Ph
46
f1 (ppm)

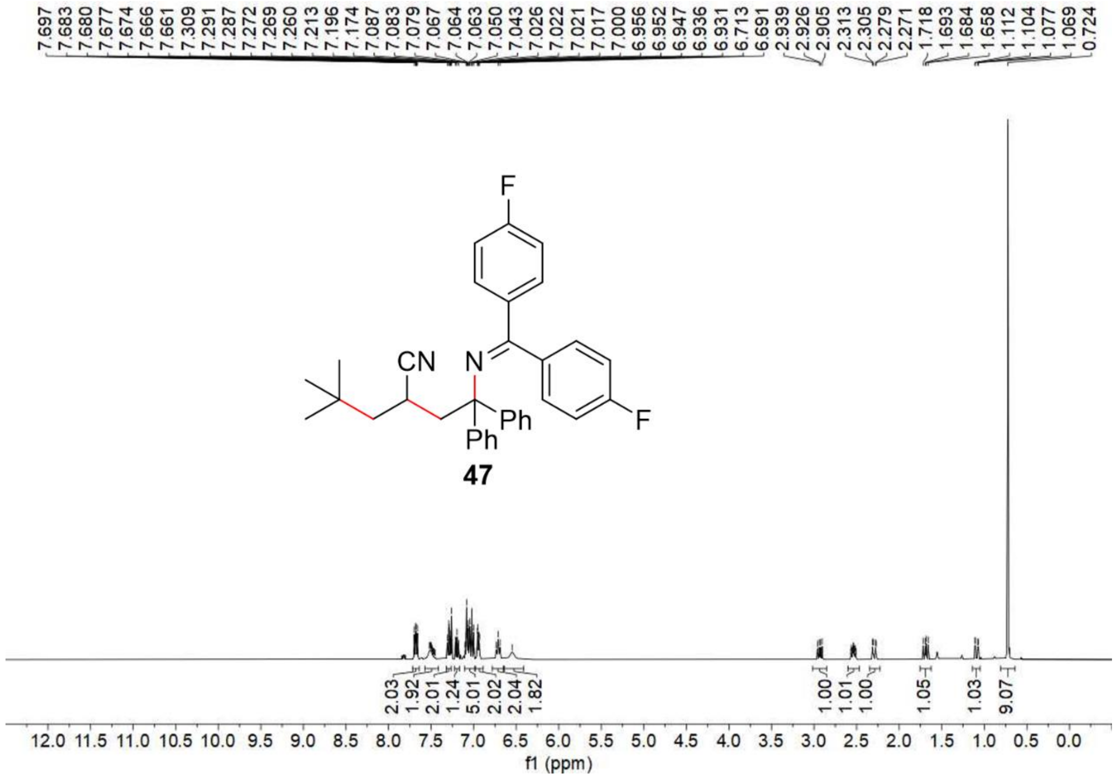
7.697 7.683 7.680 7.677 7.674 7.666 7.661 7.309 7.291 7.287 7.272 7.269 7.260 7.213 7.196 7.174 7.087 7.083 7.079 7.067 7.064 7.063 7.050 7.043 7.026 7.022 7.021 7.017 7.000 6.956 6.952 6.947 6.936 6.931 6.713 6.691 2.939 2.926 2.905 2.313 2.305 2.279 2.271 1.718 1.693 1.684 1.658 1.112 1.104 1.077 1.069 0.724
F
CN
N
Ph
Ph
F
47
2.03 1.92 2.01 1.24 5.01 2.02 2.04 1.82
1.00 1.01 1.00 1.05 1.03 9.07
12.0 11.5 11.0 10.5 10.0 9.5 9.0 8.5 8.0 7.5 7.0 6.5 6.0 5.5 5.0 4.5 4.0 3.5 3.0 2.5 2.0 1.5 1.0 0.5 0.0
f1 (ppm)

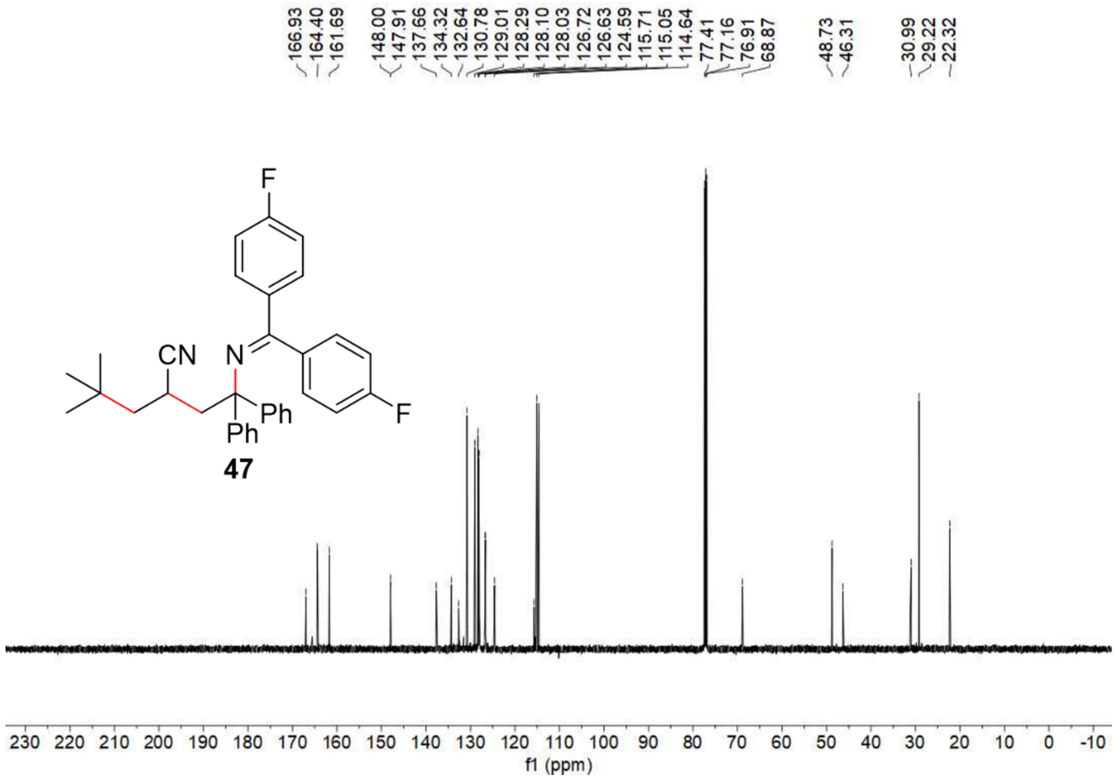
166.93 164.40 161.69 148.00 147.91 137.66 134.32 132.64 130.78 129.01 128.29 128.10 128.03 126.72 126.63 124.59 115.71 115.05 114.64 77.41 77.16 76.91 68.87 48.73 46.31 30.99 29.22 22.32
F
CN
N
Ph
Ph
F
47
230 220 210 200 190 180 170 160 150 140 130 120 110 100 90 80 70 60 50 40 30 20 10 0 -10
f1 (ppm)

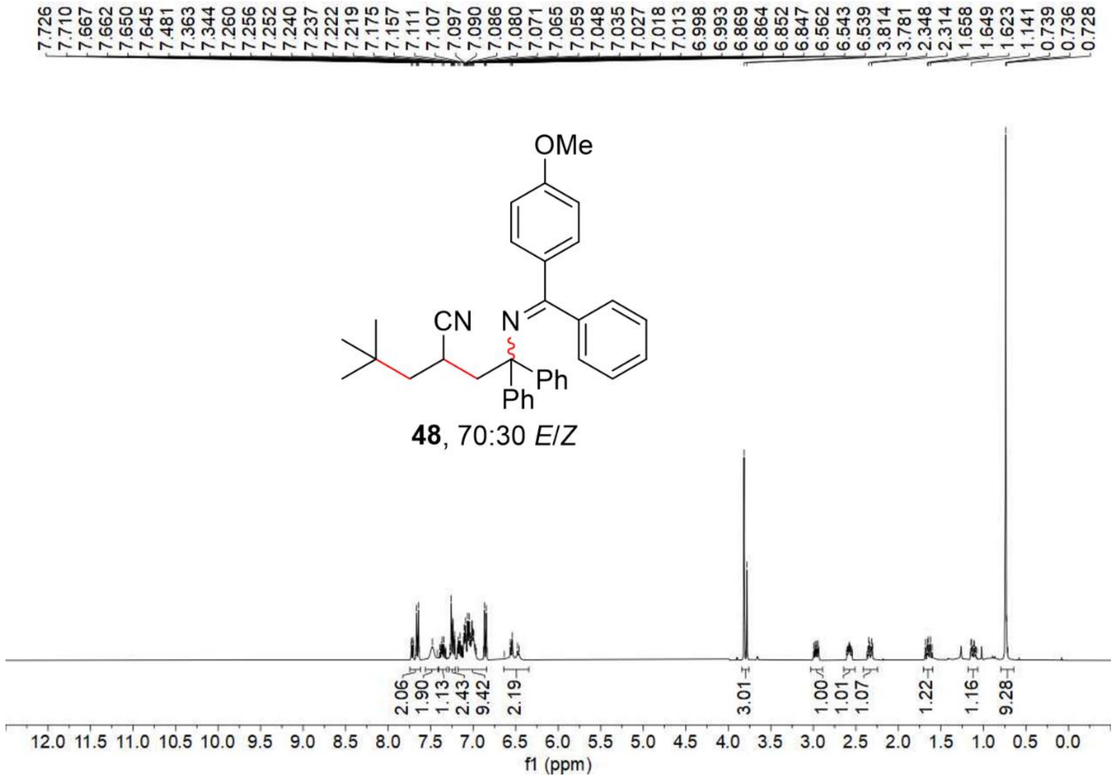
7.726 7.710 7.667 7.662 7.650 7.645 7.481 7.363 7.344 7.260 7.256 7.252 7.240 7.237 7.222 7.219 7.175 7.157 7.111 7.107 7.097 7.090 7.086 7.080 7.071 7.065 7.059 7.048 7.035 7.027 7.018 7.013 6.998 6.993 6.869 6.864 6.852 6.847 6.562 6.543 6.539 3.814 3.781 2.348 2.314 1.658 1.649 1.623 1.141 0.739 0.736 0.728
OMe
CN
N
Ph
Ph
48, 70:30 E/Z
2.06 1.90 1.13 2.43 9.42 2.19 3.01 1.00 1.01 1.07 1.22 1.16 9.28
12.0 11.5 11.0 10.5 10.0 9.5 9.0 8.5 8.0 7.5 7.0 6.5 6.0 5.5 5.0 4.5 4.0 3.5 3.0 2.5 2.0 1.5 1.0 0.5 0.0
f1 (ppm)

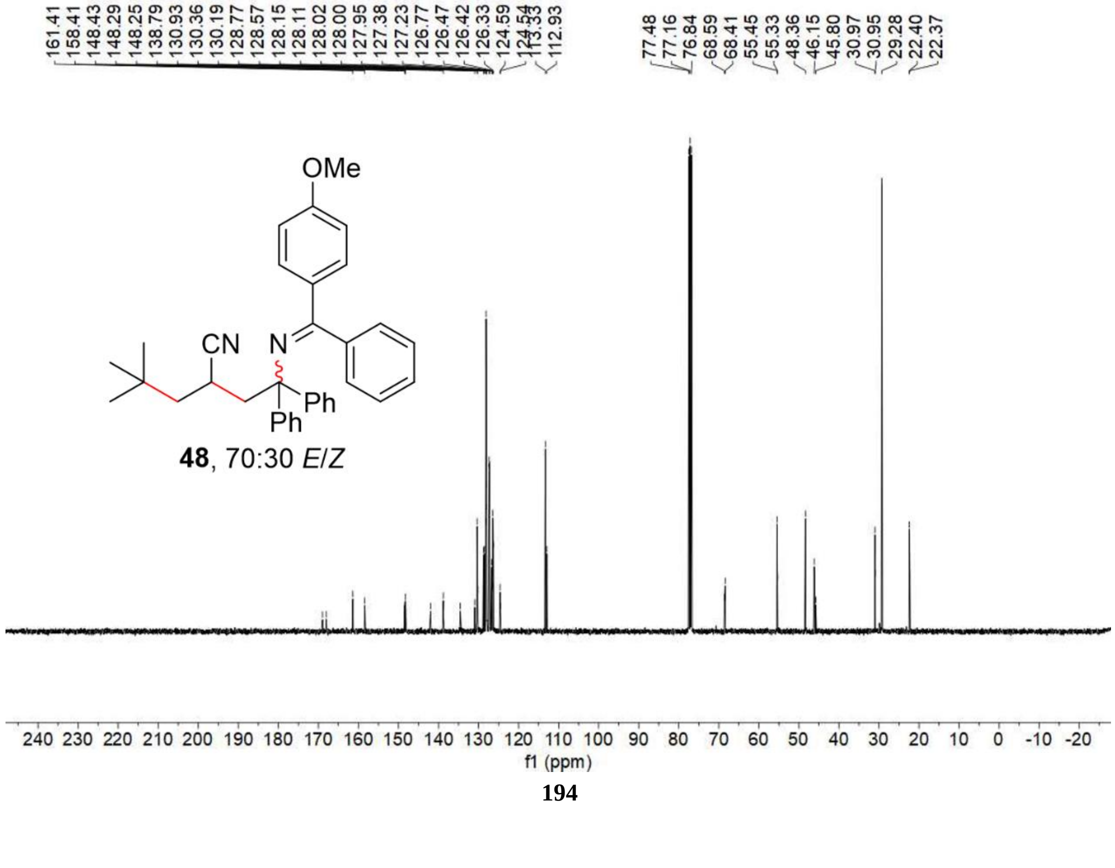
161.41 158.41 148.43 148.29 148.25 138.79 130.93 130.36 130.19 128.77 128.57 128.15 128.11 128.02 128.00 127.95 127.38 127.23 126.77 126.47 126.42 126.33 124.59 124.54 113.33 112.93
77.48 77.16 76.84 68.59 68.41 55.45 55.33 48.36 46.15 45.80 30.97 30.95 29.28 22.40 22.37
OMe
CN
N
Ph
Ph
48, 70:30 E/Z
240 230 220 210 200 190 180 170 160 150 140 130 120 110 100 90 80 70 60 50 40 30 20 10 0 -10 -20
f1 (ppm)

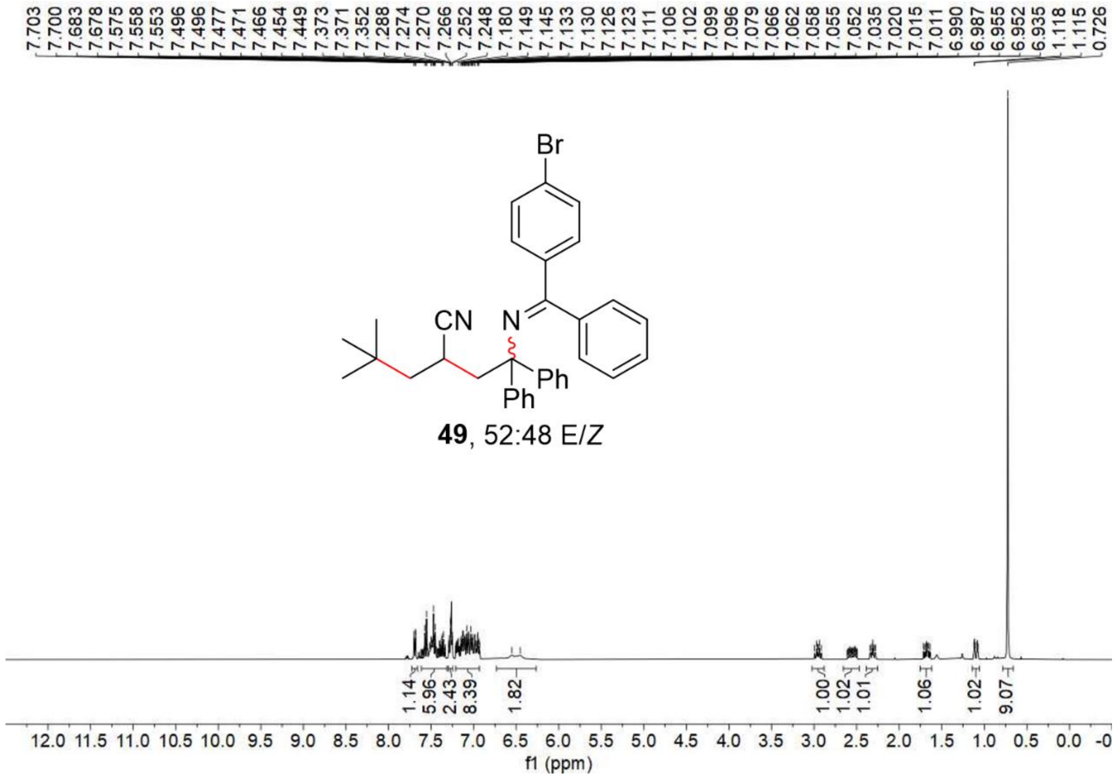
7.703 7.700 7.683 7.678 7.575 7.558 7.553 7.496 7.496 7.477 7.471 7.466 7.454 7.449 7.373 7.371 7.352 7.288 7.274 7.270 7.266 7.252 7.248 7.180 7.149 7.145 7.133 7.130 7.126 7.123 7.111 7.106 7.102 7.099 7.096 7.079 7.066 7.062 7.058 7.055 7.052 7.035 7.020 7.015 7.011 6.990 6.987 6.955 6.952 6.935 1.118 1.115 0.726
Br
CN
N
Ph
Ph
49, 52:48 E/Z
1.14 5.96 2.43 8.39 1.82 1.00 1.02 1.01 1.06 1.02 9.07
12.0 11.5 11.0 10.5 10.0 9.5 9.0 8.5 8.0 7.5 7.0 6.5 6.0 5.5 5.0 4.5 4.0 3.5 3.0 2.5 2.0 1.5 1.0 0.5 0.0 -0
f1 (ppm)

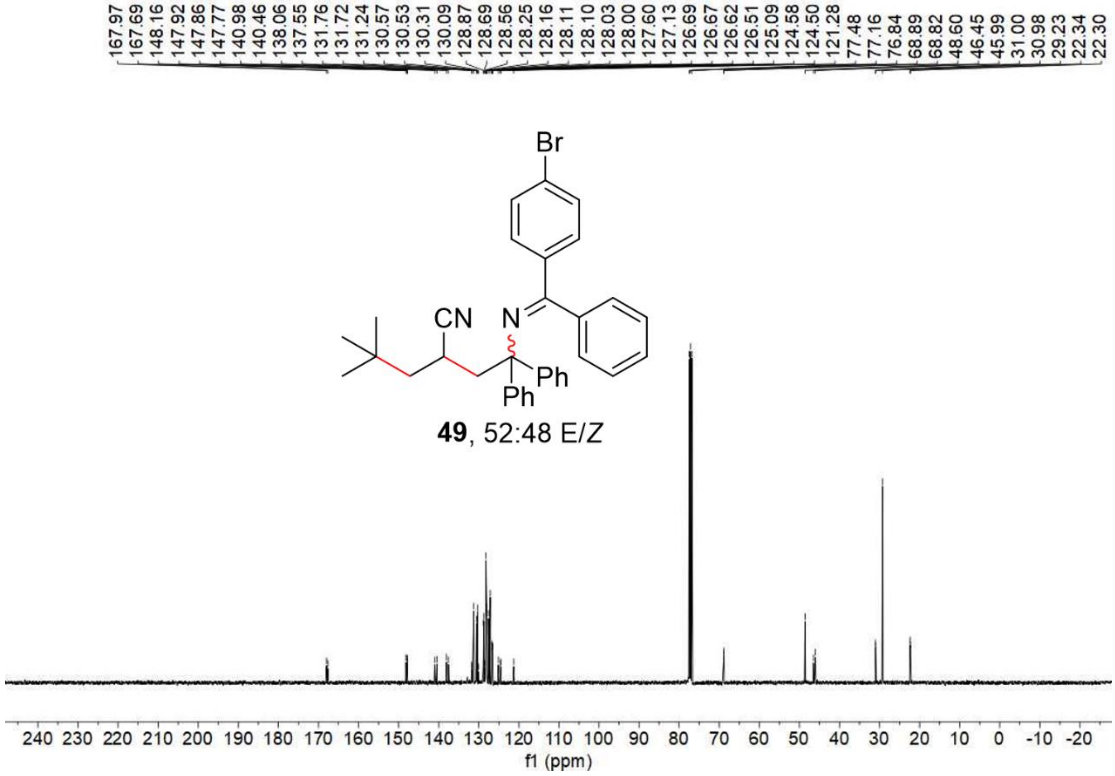
167.97 167.69 148.16 147.92 147.86 147.77 140.98 140.46 138.06 137.55 131.76 131.72 131.24 130.57 130.53 130.31 130.09 128.87 128.69 128.56 128.25 128.16 128.11 128.10 128.03 128.00 127.60 127.13 126.69 126.67 126.62 126.51 125.09 124.58 124.50 121.28 77.48 77.16 76.84 68.89 68.82 48.60 46.45 45.99 31.00 30.98 29.23 22.34 22.30
Br
CN
N
Ph
Ph
49, 52:48 E/Z
240 230 220 210 200 190 180 170 160 150 140 130 120 110 100 90 80 70 60 50 40 30 20 10 0 -10 -20
f1 (ppm)

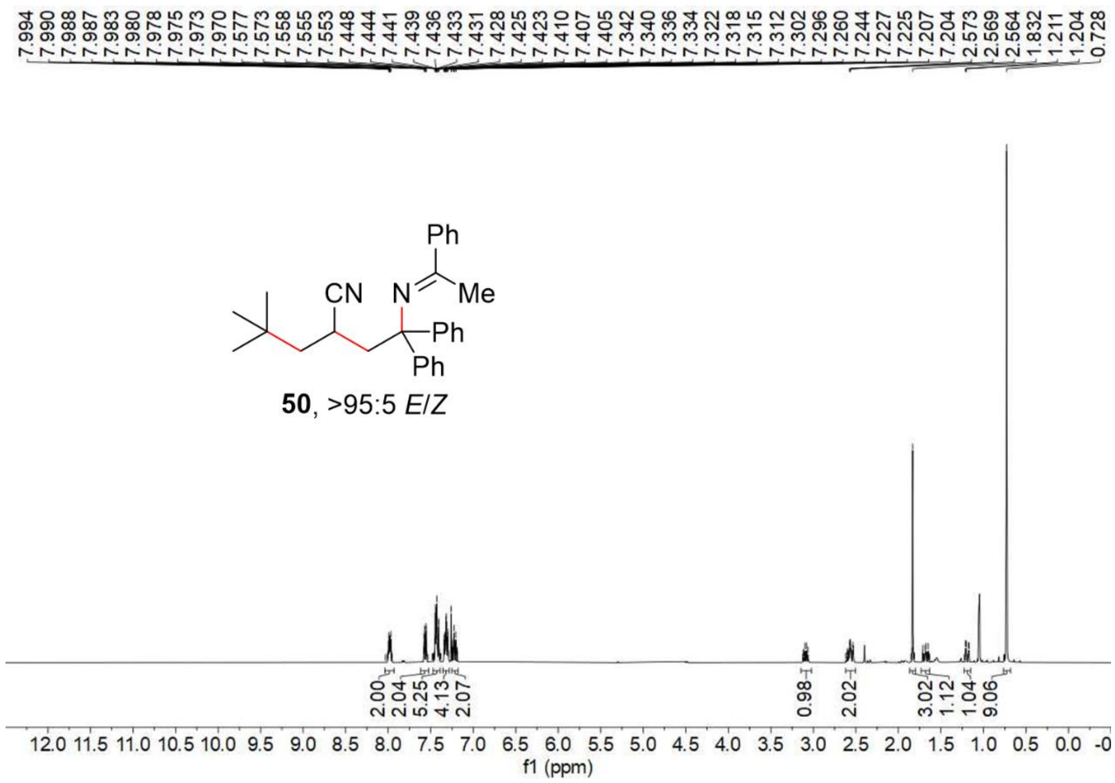

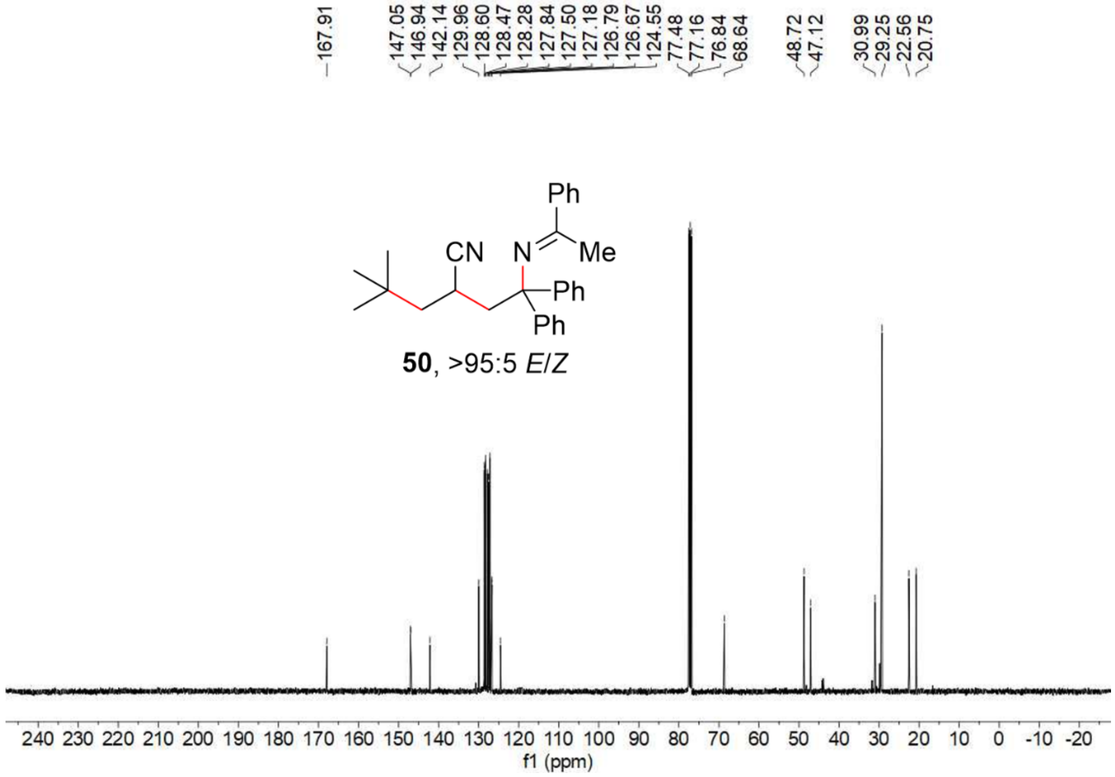

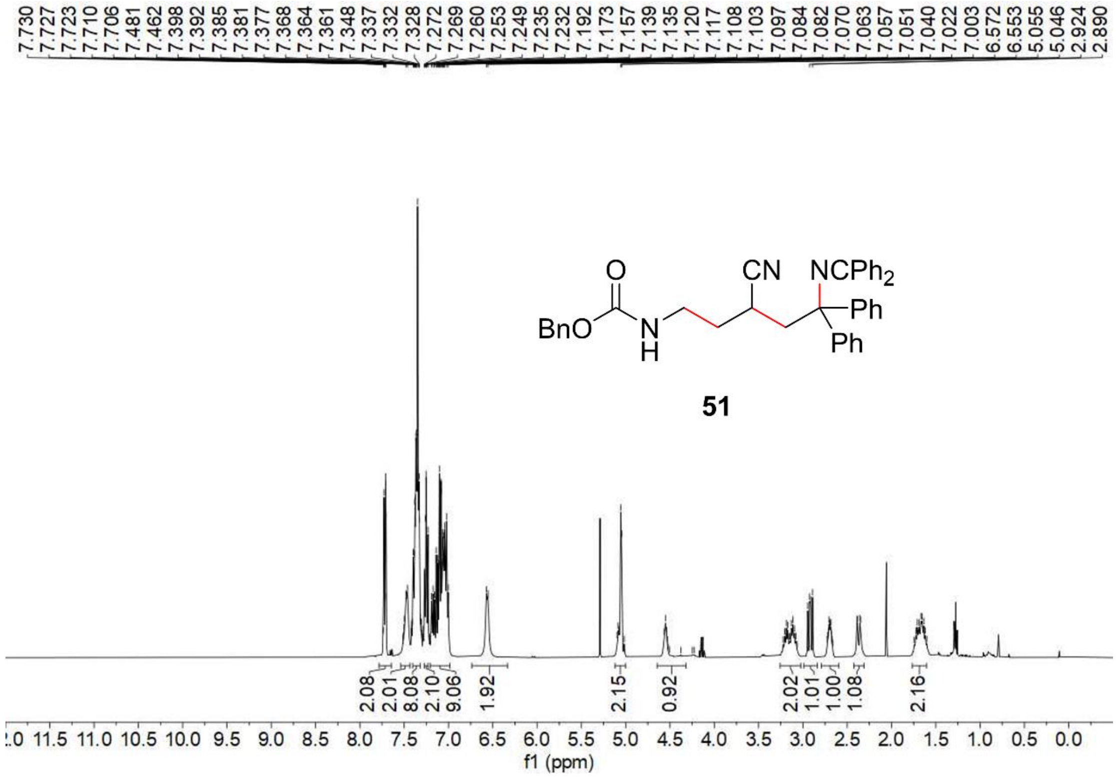
7.730 7.727 7.723 7.710 7.706 7.481 7.462 7.398 7.392 7.385 7.381 7.377 7.368 7.364 7.361 7.348 7.337 7.332 7.328 7.272 7.269 7.260 7.253 7.249 7.235 7.232 7.192 7.173 7.157 7.139 7.135 7.120 7.117 7.108 7.103 7.097 7.084 7.082 7.070 7.063 7.057 7.051 7.040 7.022 7.003 6.572 6.553 5.055 5.046 2.924 2.890
O
CN
NCPh2
BnO
N
H
Ph
Ph
51
2.08 2.01 8.08 2.10 9.06 1.92 2.15 0.92 2.02 1.01 1.00 1.08 2.16
12.0 11.5 11.0 10.5 10.0 9.5 9.0 8.5 8.0 7.5 7.0 6.5 6.0 5.5 5.0 4.5 4.0 3.5 3.0 2.5 2.0 1.5 1.0 0.5 0.0
f1 (ppm)

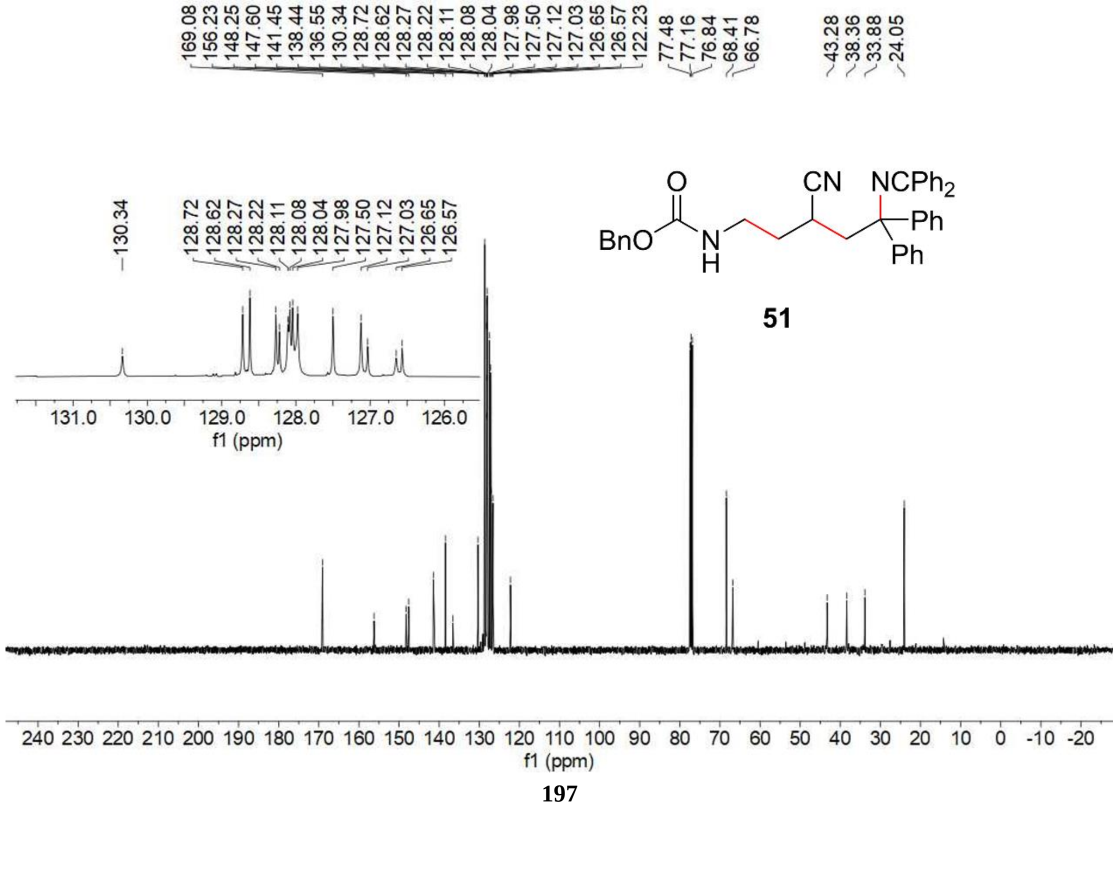
169.08 156.23 148.25 147.60 141.45 138.44 136.55 130.34 128.72 128.62 128.27 128.22 128.11 128.08 128.04 127.98 127.50 127.12 127.03 126.65 126.57 122.23
77.48 77.16 76.84 68.41 66.78
43.28 38.36 33.88 24.05
130.34
128.72 128.62 128.27 128.22 128.11 128.08 128.04 127.98 127.50 127.12 127.03 126.65 126.57
131.0 130.0 129.0 128.0 127.0 126.0
f1 (ppm)
O
CN
NCPh2
BnO
N
H
Ph
Ph
51
240 230 220 210 200 190 180 170 160 150 140 130 120 110 100 90 80 70 60 50 40 30 20 10 0 -10 -20
f1 (ppm)

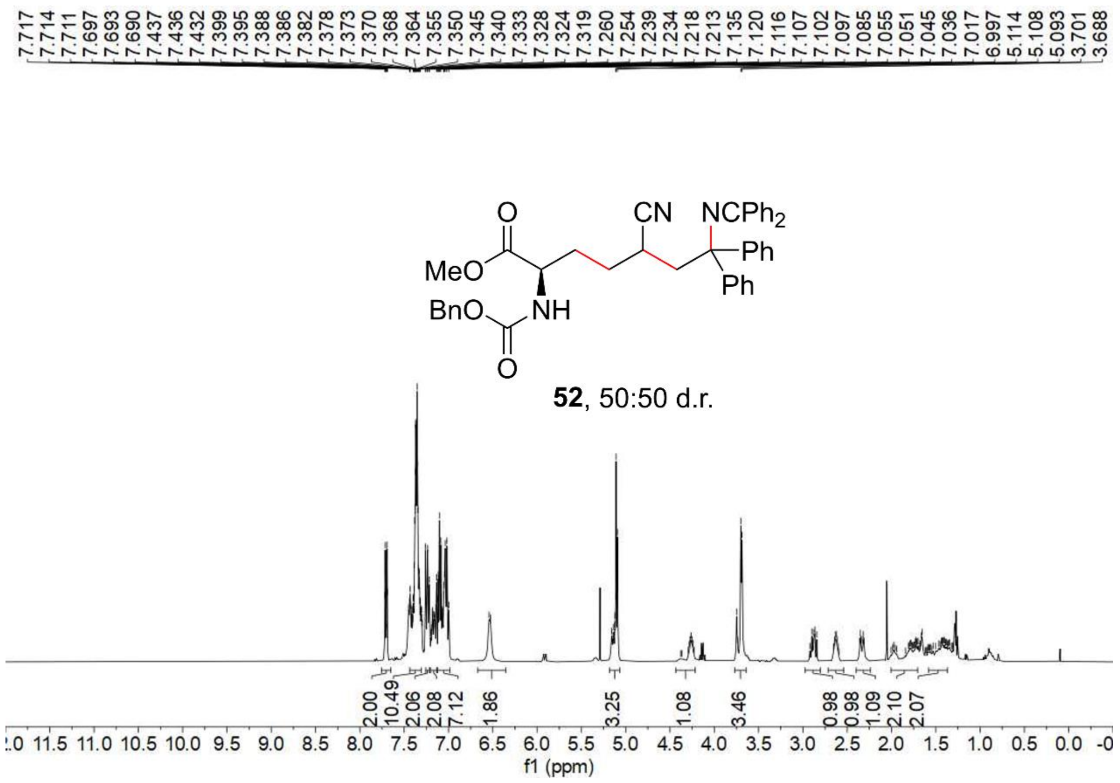
7.717 7.714 7.711 7.697 7.693 7.690 7.437 7.436 7.432 7.399 7.395 7.388 7.386 7.382 7.378 7.373 7.370 7.368 7.364 7.355 7.350 7.345 7.340 7.333 7.328 7.324 7.319 7.260 7.254 7.239 7.234 7.218 7.213 7.135 7.120 7.116 7.107 7.102 7.097 7.085 7.055 7.051 7.045 7.036 7.017 6.997 5.114 5.108 5.093 3.701 3.688
O
CN
NCPh2
MeO
Ph
Ph
BnO
NH
O
52, 50:50 d.r.
2.00 10.49 2.06 2.08 7.12 1.86 3.25 1.08 3.46 0.98 0.98 1.09 2.10 2.07
f1 (ppm)

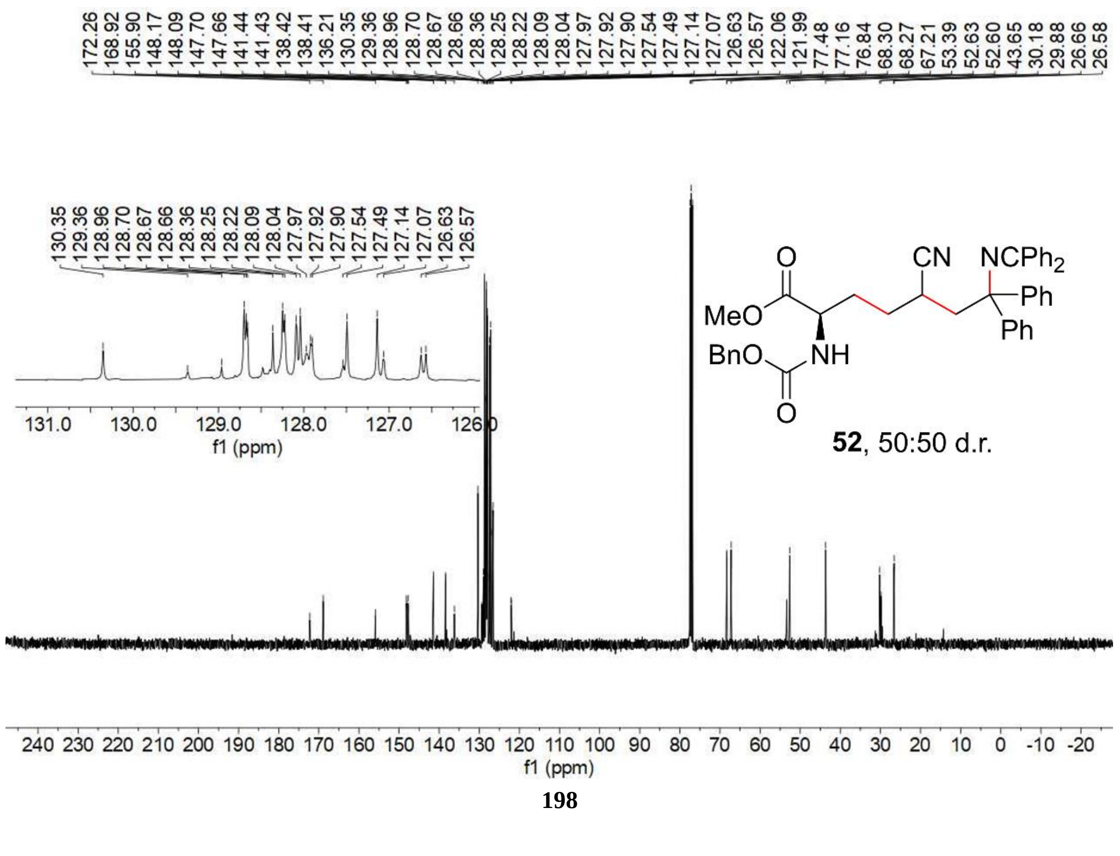
172.26 168.92 155.90 148.17 148.09 147.70 147.66 141.44 141.43 138.42 138.41 136.21 130.35 129.36 128.96 128.70 128.67 128.66 128.36 128.25 128.22 128.09 128.04 127.97 127.92 127.90 127.54 127.49 127.14 127.07 126.63 126.57 122.06 121.99 77.48 77.16 76.84 68.30 68.27 67.21 53.39 52.63 52.60 43.65 30.18 29.88 26.66 26.58
130.35 129.36 128.96 128.70 128.67 128.66 128.36 128.25 128.22 128.09 128.04 127.97 127.92 127.90 127.54 127.49 127.14 127.07 126.63 126.57
f1 (ppm)
O
CN
NCPh2
MeO
Ph
Ph
BnO
NH
O
52, 50:50 d.r.
f1 (ppm)

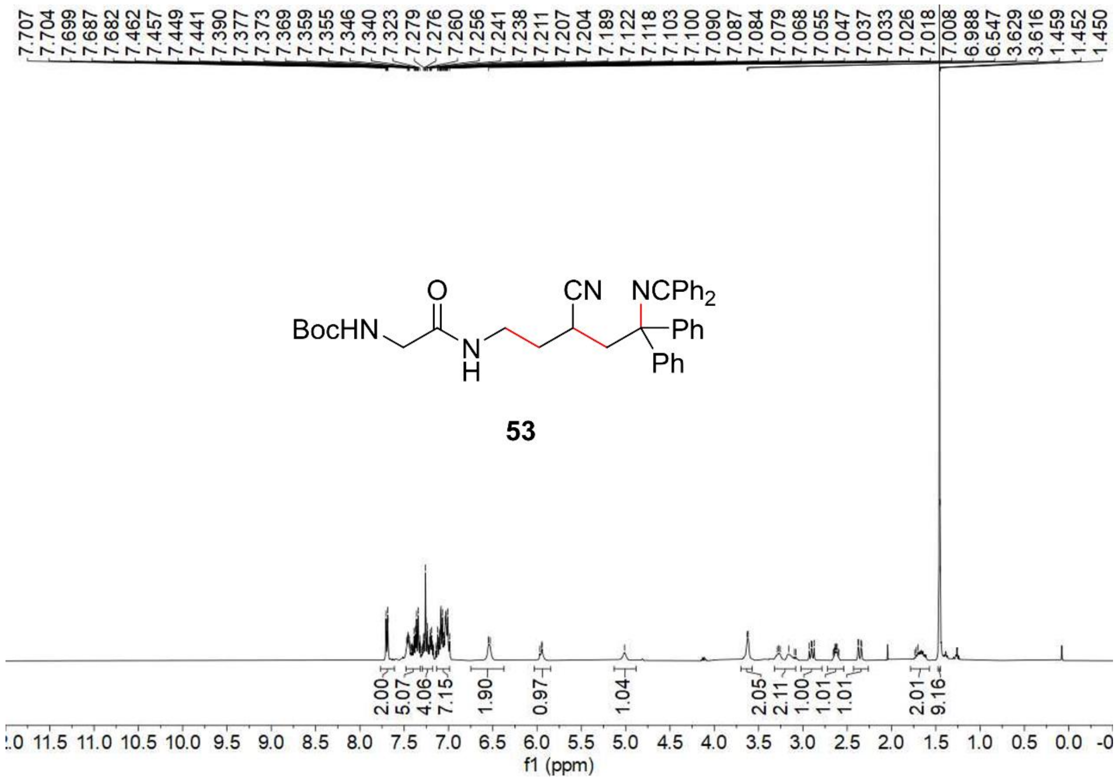
7.707 7.704 7.699 7.687 7.682 7.462 7.457 7.449 7.441 7.390 7.377 7.373 7.369 7.359 7.355 7.346 7.340 7.323 7.279 7.276 7.260 7.256 7.241 7.238 7.211 7.207 7.204 7.189 7.122 7.118 7.103 7.100 7.090 7.087 7.084 7.079 7.068 7.055 7.047 7.037 7.033 7.026 7.018 7.008 6.988 6.547 3.629 3.616 1.459 1.452 1.450
BocHN
O
N
H
CN
NCPh2
Ph
Ph
53
2.00 5.07 4.06 7.15 1.90 0.97 1.04 2.05 2.11 1.00 1.01 1.01 2.01 9.16
f1 (ppm)

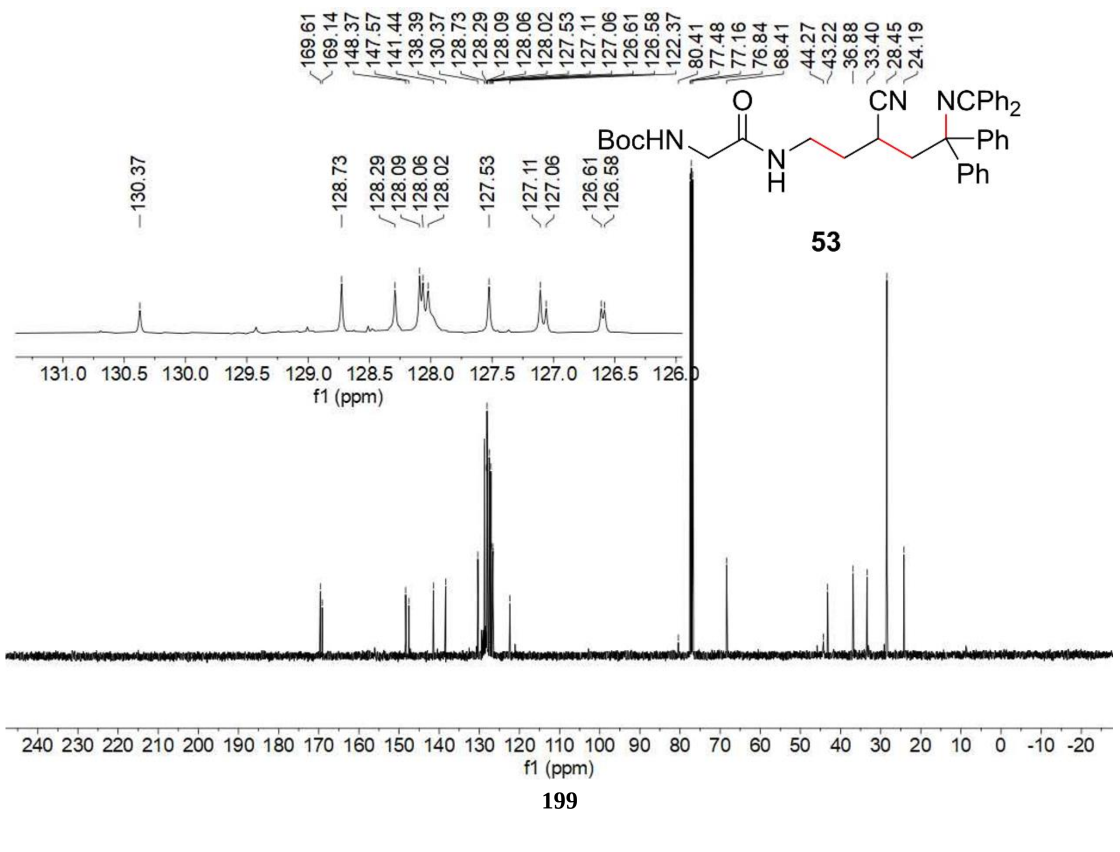
169.61 169.14 148.37 147.57 141.44 138.39 130.37 128.73 128.29 128.09 128.06 128.02 127.53 127.11 127.06 126.61 126.58 122.37 80.41 77.48 77.16 76.84 68.41 44.27 43.22 36.88 33.40 28.45 24.19
BocHN
O
N
H
CN
NCPh2
Ph
Ph
53
130.37 128.73 128.29 128.09 128.06 128.02 127.53 127.11 127.06 126.61 126.58
f1 (ppm)
f1 (ppm)

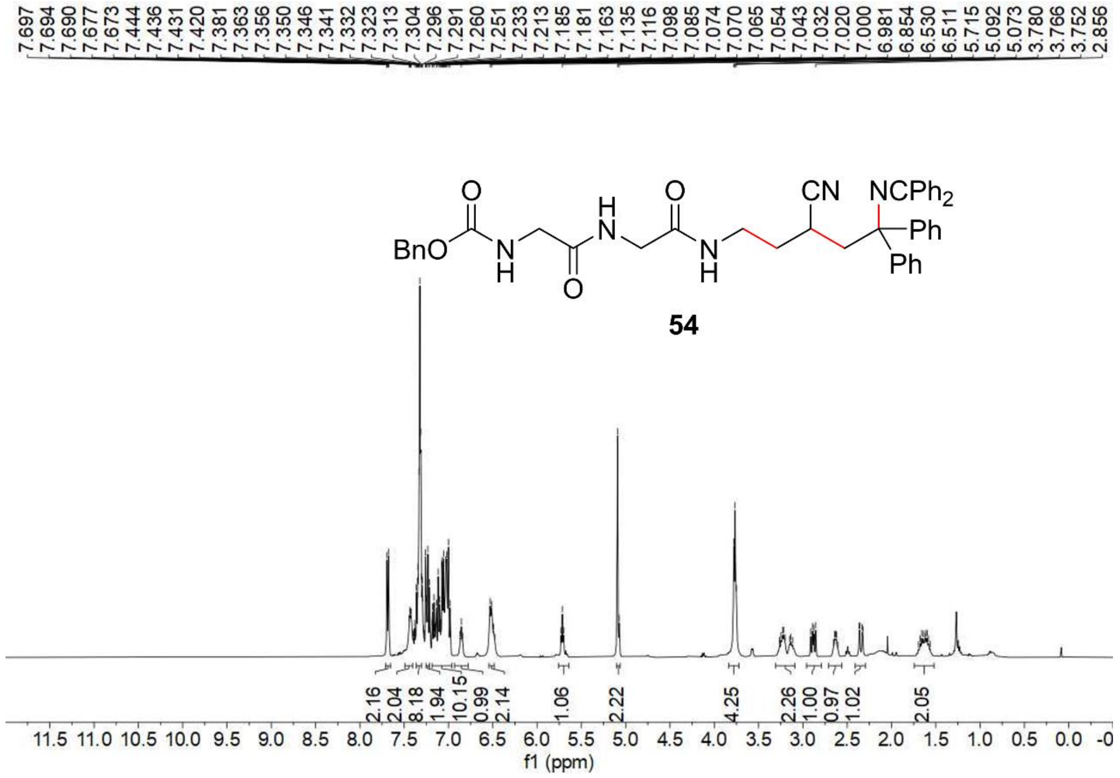
7.697 7.694 7.690 7.677 7.673 7.444 7.436 7.431 7.420 7.381 7.363 7.356 7.350 7.346 7.341 7.332 7.323 7.313 7.304 7.296 7.291 7.260 7.251 7.233 7.213 7.185 7.181 7.163 7.135 7.116 7.098 7.085 7.074 7.070 7.065 7.054 7.043 7.032 7.020 7.000 6.981 6.854 6.530 6.511 5.715 5.092 5.073 3.780 3.766 3.752 2.856
BnO
NCPh2
CN
Ph
54
2.16 2.04 8.18 1.94 10.15 0.99 2.14 1.06 2.22 4.25 2.26 1.00 0.97 1.02 2.05
f1 (ppm)

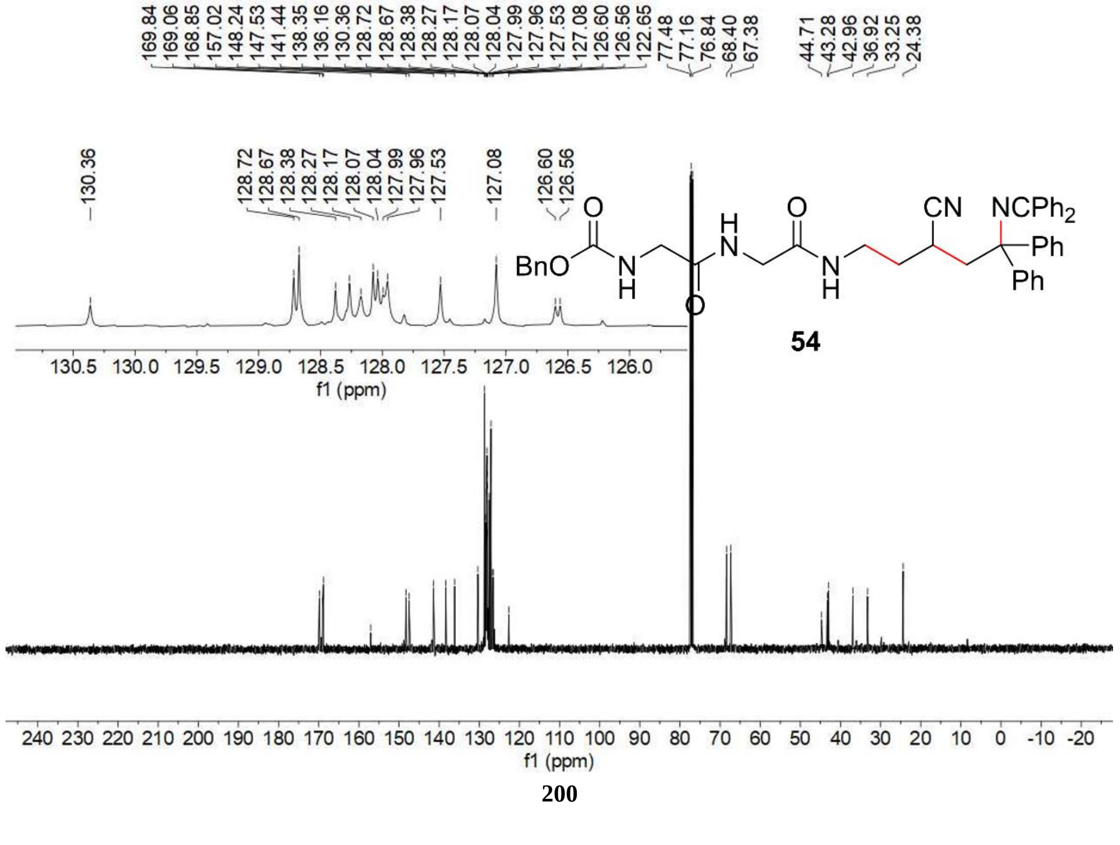
169.84 169.06 168.85 157.02 148.24 147.53 141.44 138.35 136.16 130.36 128.72 128.67 128.38 128.27 128.17 128.07 128.04 127.99 127.96 127.53 127.08 126.60 126.56 122.65 77.48 77.16 76.84 68.40 67.38 44.71 43.28 42.96 36.92 33.25 24.38
BnO
NCPh2
CN
Ph
54
f1 (ppm)

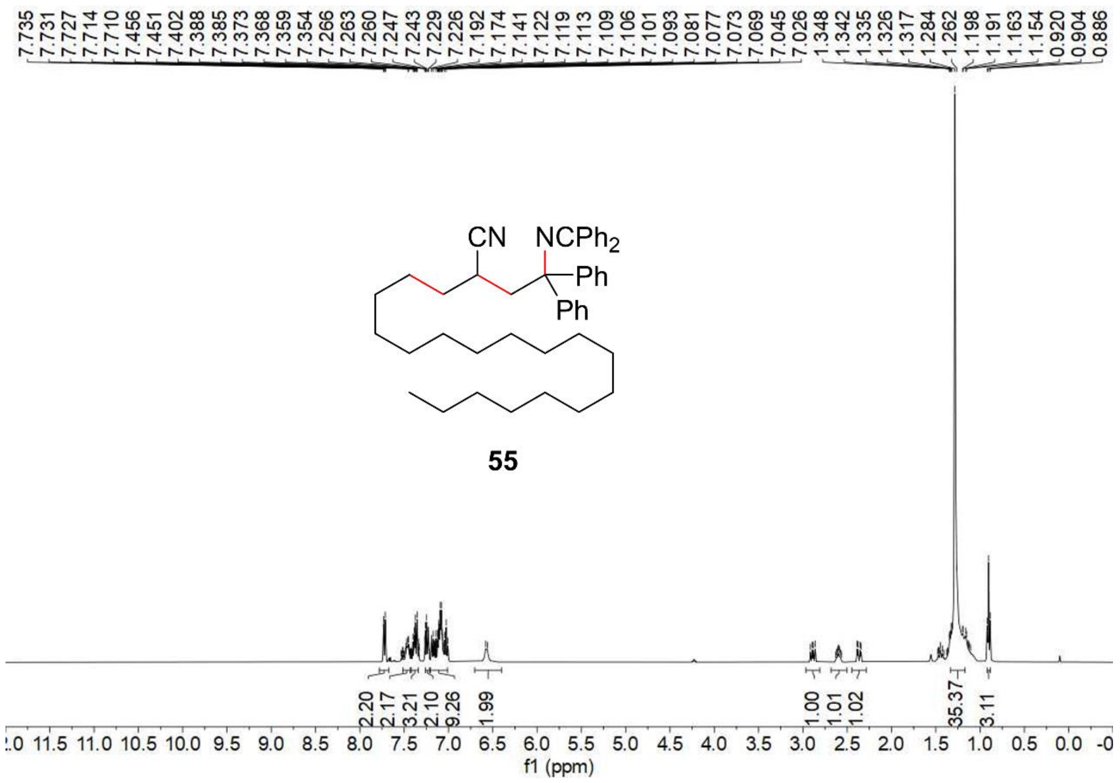
7.735
7.731
7.727
7.714
7.710
7.456
7.451
7.402
7.388
7.385
7.373
7.368
7.359
7.354
7.266
7.263
7.260
7.247
7.243
7.229
7.226
7.192
7.174
7.141
7.122
7.119
7.113
7.109
7.106
7.101
7.093
7.081
7.077
7.073
7.069
7.045
7.026
1.348
1.342
1.335
1.326
1.317
1.284
1.262
1.198
1.191
1.163
1.154
0.920
0.904
0.886
CN
NCPh2
Ph
Ph
55
2.20
2.17
3.21
2.10
9.26
1.99
1.00
1.01
1.02
35.37
3.11
f1 (ppm)

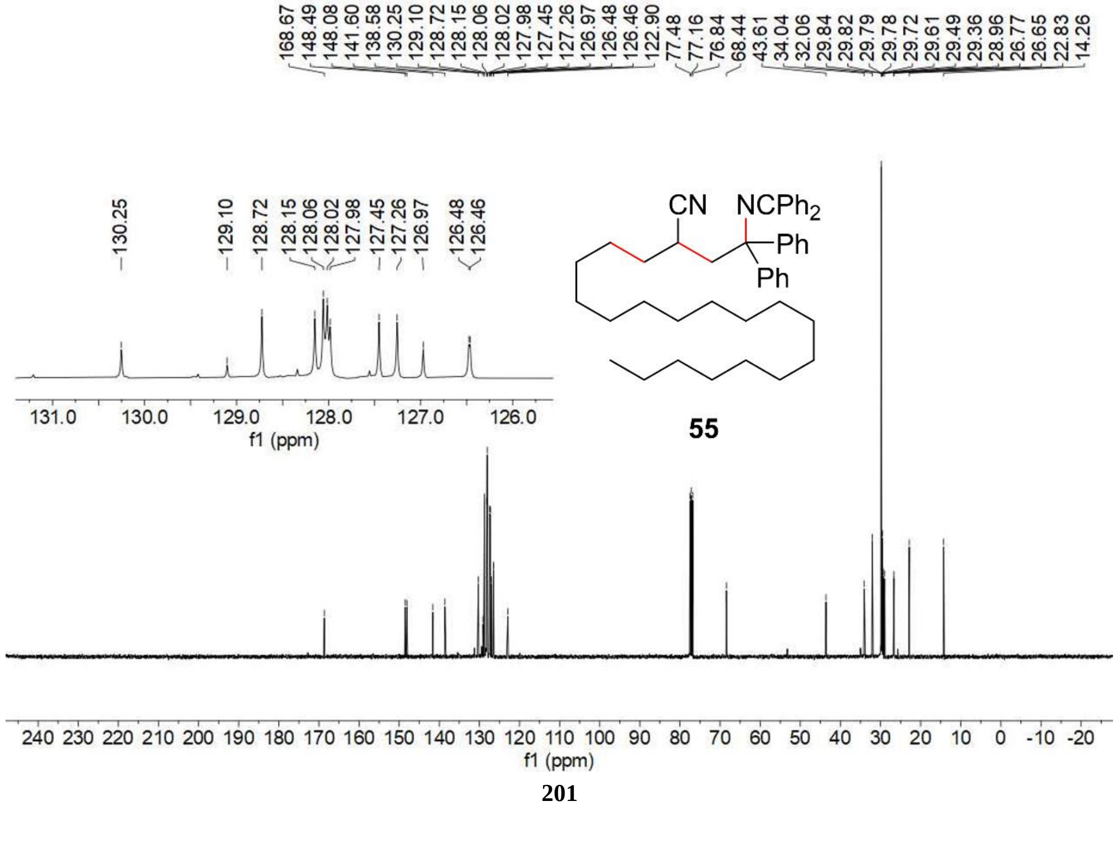
168.67
148.49
148.08
141.60
138.58
130.25
129.10
128.72
128.15
128.06
128.02
127.98
127.45
127.26
126.97
126.48
126.46
122.90
77.48
77.16
76.84
68.44
43.61
34.04
32.06
29.84
29.82
29.79
29.78
29.72
29.61
29.49
29.36
28.96
26.77
26.65
22.83
14.26
CN
NCPh2
Ph
Ph
55
f1 (ppm)

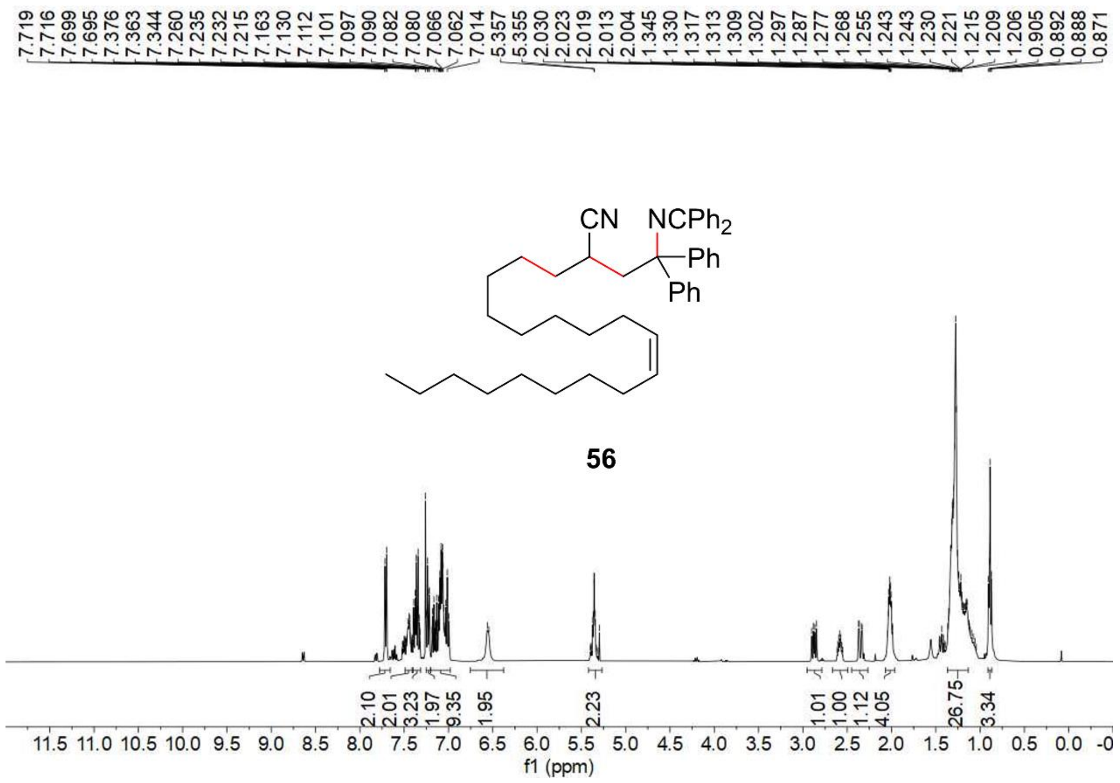

56

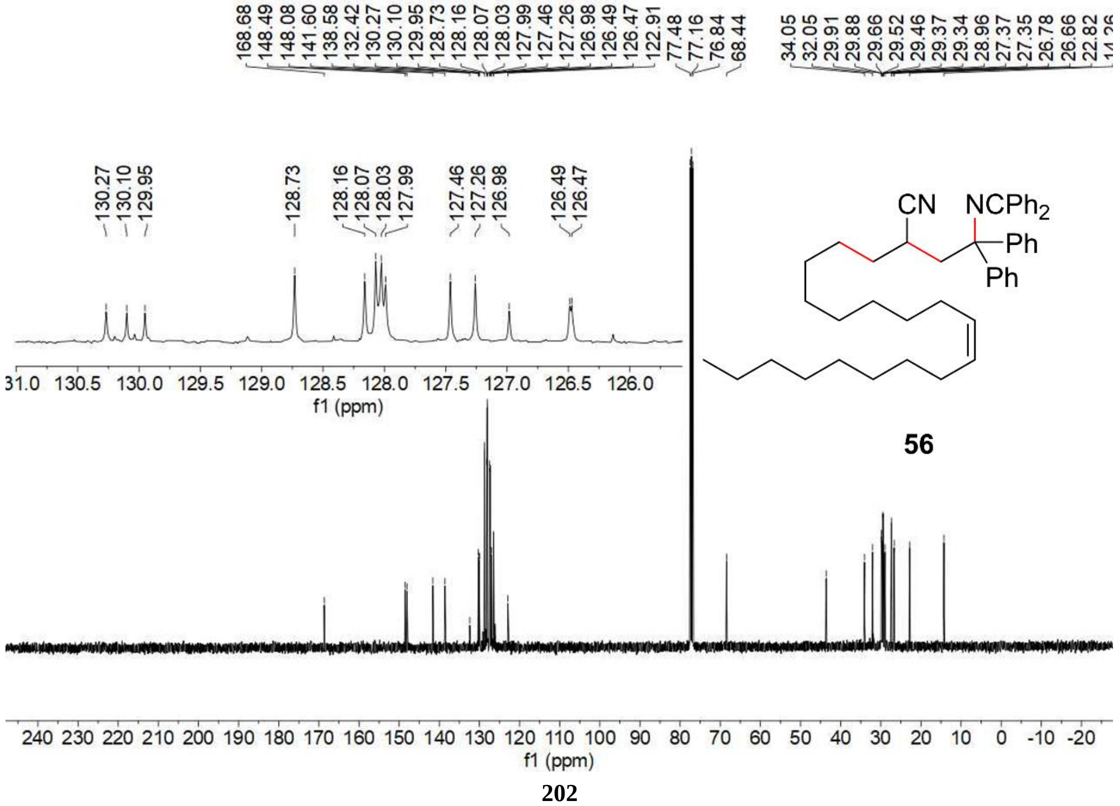

56

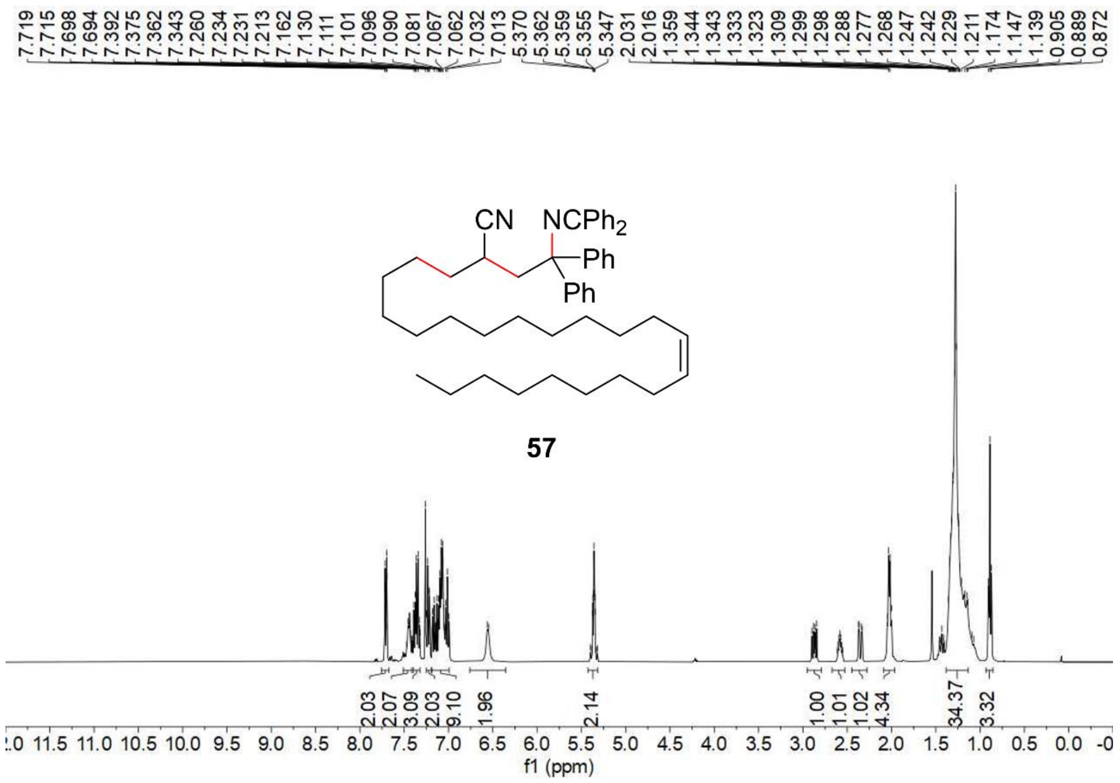
7.719 7.715 7.698 7.694 7.392 7.375 7.362 7.343 7.260 7.234 7.231 7.213 7.162 7.130 7.111 7.101 7.096 7.090 7.081 7.067 7.062 7.032 7.013 5.370 5.362 5.359 5.355 5.347 2.031 2.016 1.359 1.344 1.343 1.333 1.323 1.309 1.299 1.298 1.288 1.277 1.268 1.247 1.242 1.229 1.211 1.174 1.147 1.139 0.905 0.889 0.872
CN
NCPh$_2$
Ph
Ph
57
2.03 2.07 3.09 2.03 9.10 1.96 2.14 1.00 1.01 1.02 4.34 34.37 3.32
f1 (ppm)

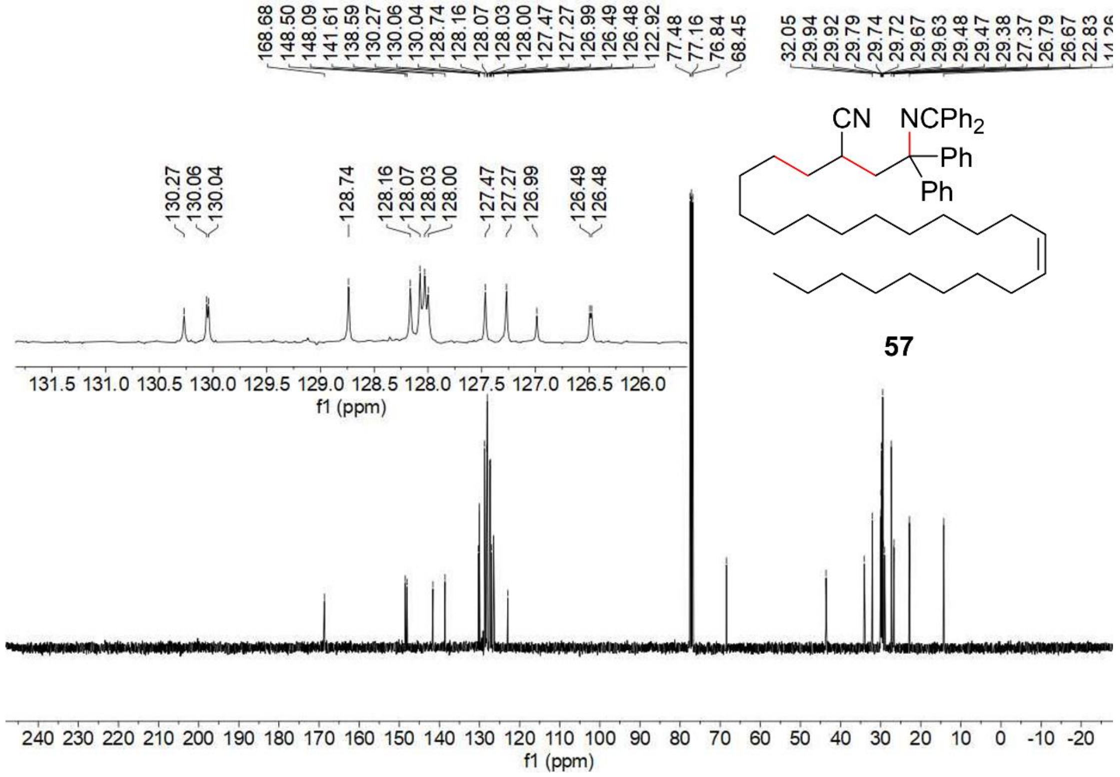
168.68 148.50 148.09 141.61 138.59 130.27 130.06 130.04 128.74 128.16 128.07 128.03 128.00 127.47 127.27 126.99 126.49 126.48 122.92 77.48 77.16 76.84 68.45 32.05 29.94 29.92 29.79 29.74 29.72 29.67 29.63 29.48 29.47 29.38 27.37 26.79 26.67 22.83
130.27 130.06 130.04 128.74 128.16 128.07 128.03 128.00 127.47 127.27 126.99 126.49 126.48
f1 (ppm)
CN
NCPh$_2$
Ph
Ph
57
f1 (ppm)

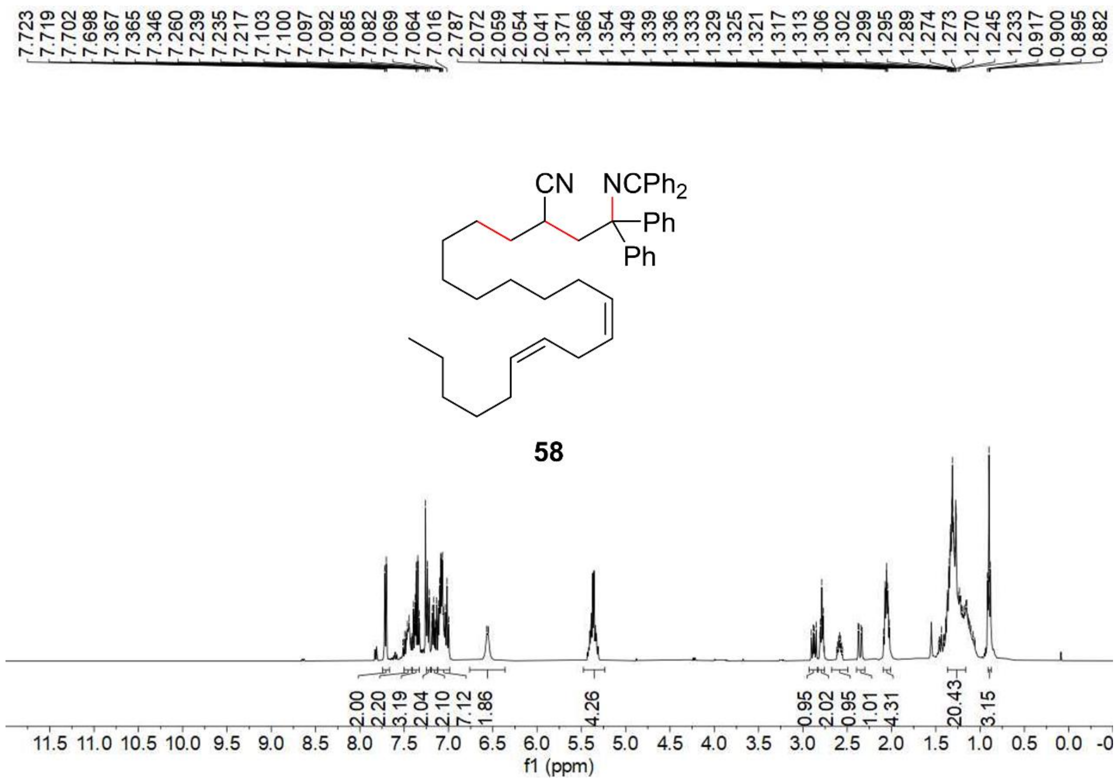
7.723 7.719 7.702 7.367 7.365 7.346 7.260 7.239 7.235 7.217 7.103 7.100 7.097 7.092 7.085 7.082 7.069 7.064 7.016 2.787 2.072 2.059 2.054 2.041 1.371 1.366 1.354 1.349 1.339 1.336 1.333 1.329 1.325 1.321 1.317 1.313 1.306 1.302 1.299 1.295 1.289 1.274 1.273 1.270 1.245 1.233 0.917 0.900 0.895 0.882
CN
NCPh2
Ph
Ph
58
2.00 2.20 3.19 2.04 2.10 7.12 1.86 4.26 0.95 2.02 0.95 1.01 4.31 20.43 3.15
f1 (ppm)

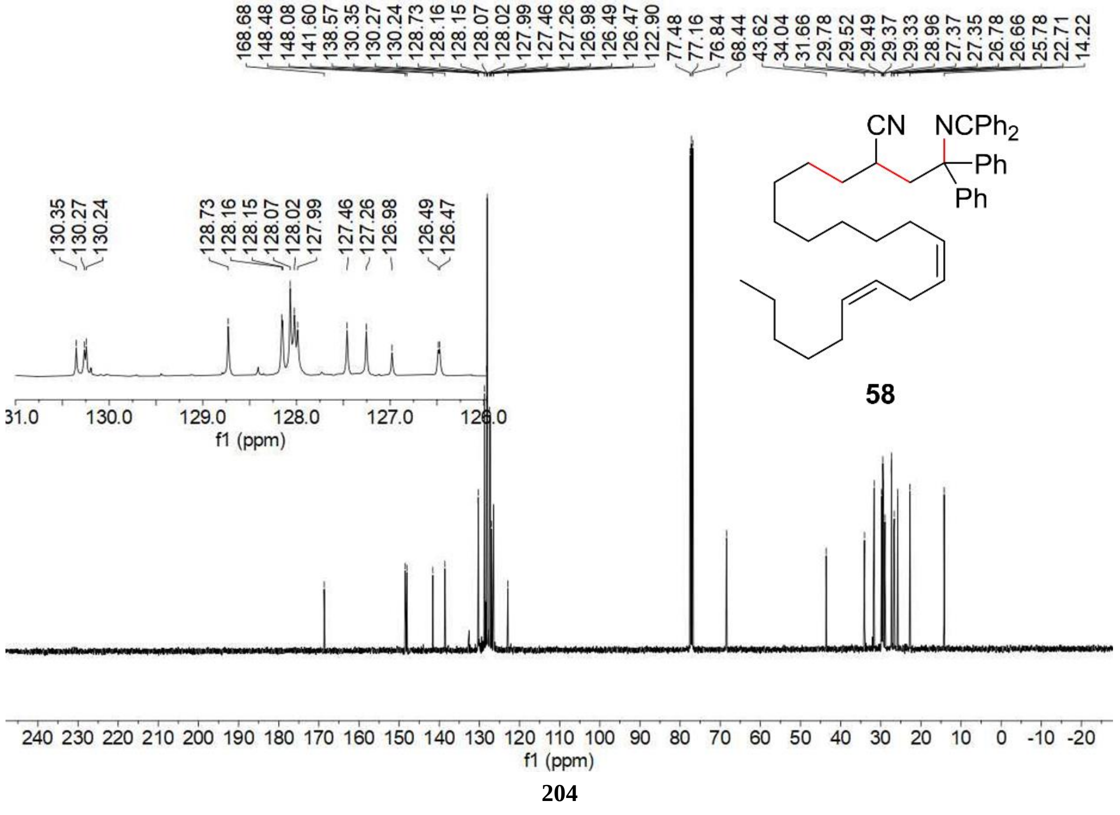
168.68 148.48 148.08 141.60 138.57 130.35 130.27 130.24 128.73 128.16 128.15 128.07 128.02 127.99 127.46 127.26 126.98 126.49 126.47 122.90 77.48 77.16 76.84 68.44 43.62 34.04 31.66 29.78 29.52 29.49 29.37 29.33 28.96 27.37 27.35 26.78 26.66 25.78 22.71 14.22
130.35 130.27 130.24 128.73 128.16 128.15 128.07 128.02 127.99 127.46 127.26 126.98 126.49 126.47
f1 (ppm)
CN
NCPh2
Ph
Ph
58
f1 (ppm)

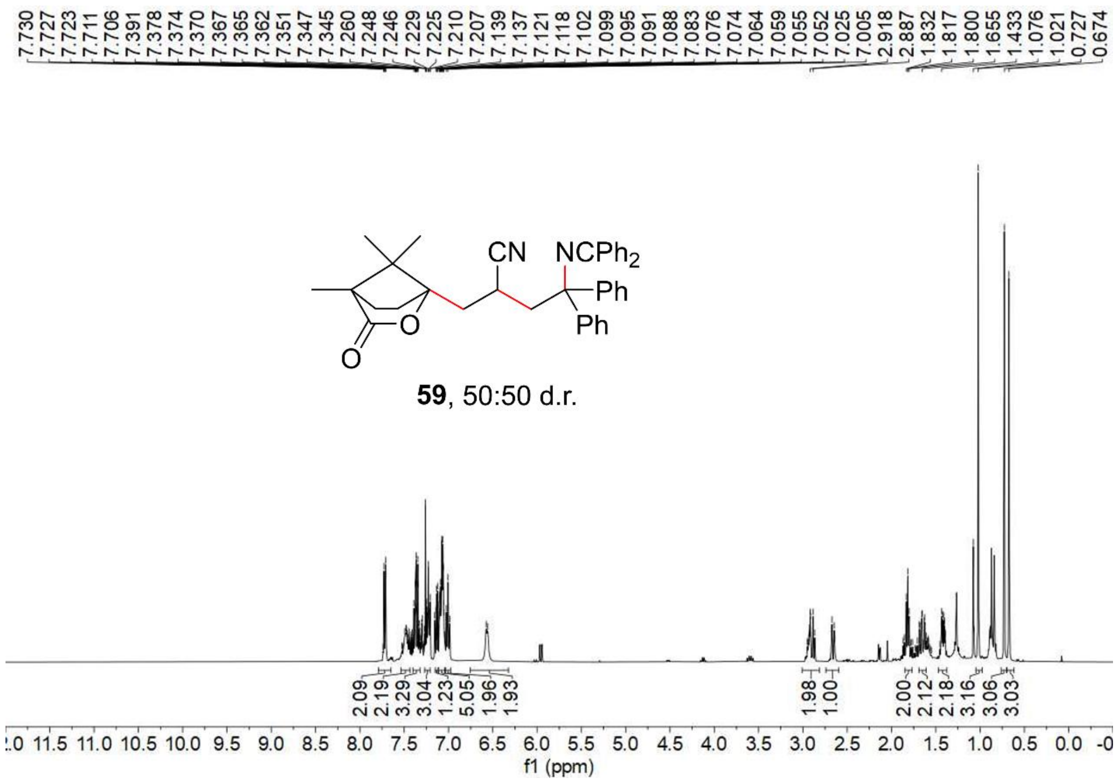
7.730 7.727 7.723 7.711 7.706 7.391 7.378 7.374 7.370 7.367 7.365 7.362 7.351 7.347 7.345 7.260 7.248 7.246 7.229 7.225 7.210 7.207 7.139 7.137 7.121 7.118 7.102 7.099 7.095 7.091 7.088 7.083 7.076 7.074 7.064 7.059 7.055 7.052 7.025 7.005 2.918 2.887 1.832 1.817 1.800 1.655 1.433 1.076 1.021 0.727 0.674
CN
NCPh2
Ph
Ph
O
O
59, 50:50 d.r.
2.09 2.19 3.29 3.04 1.23 5.05 1.96 1.93 1.98 1.00 2.00 2.12 2.18 3.16 3.06 3.03
f1 (ppm)

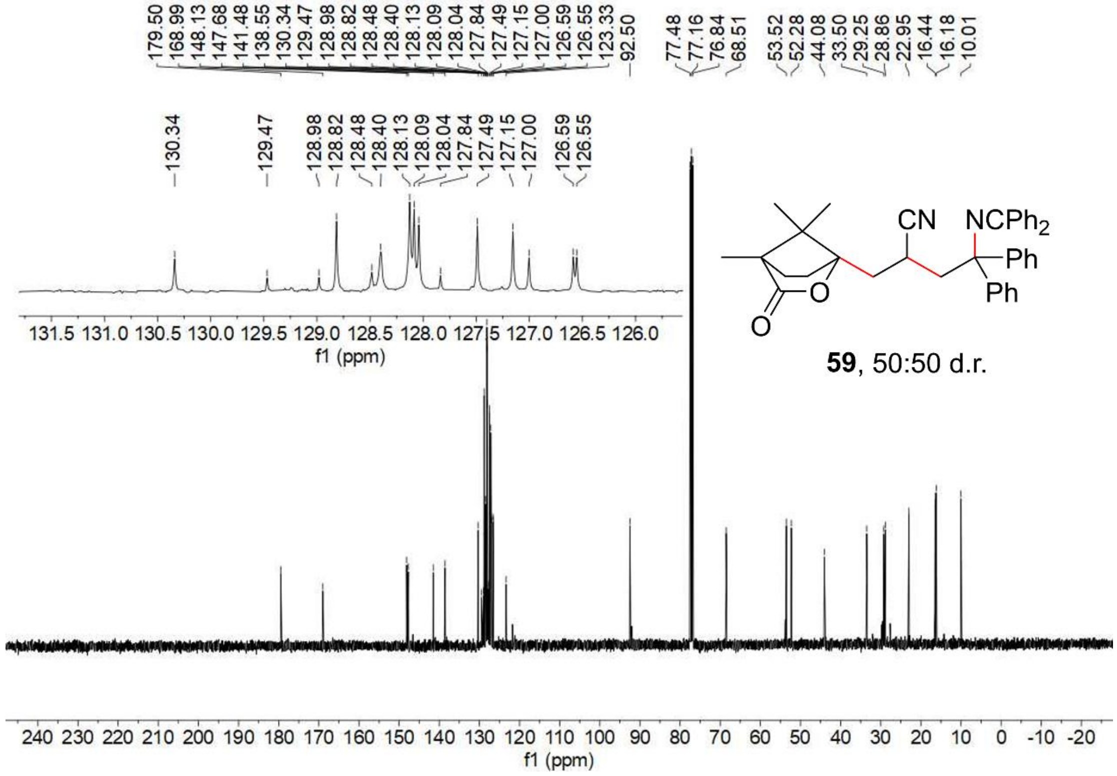
179.50 168.99 148.13 147.68 141.48 138.55 130.34 129.47 128.98 128.82 128.48 128.40 128.13 128.09 128.04 127.84 127.49 127.15 127.00 126.59 126.55 123.33 92.50 77.48 77.16 76.84 68.51 53.52 52.28 44.08 33.50 29.25 28.86 22.95 16.44 16.18 10.01
130.34 129.47 128.98 128.82 128.48 128.40 128.13 128.09 128.04 127.84 127.49 127.15 127.00 126.59 126.55
f1 (ppm)
CN
NCPh2
Ph
Ph
O
O
59, 50:50 d.r.
f1 (ppm)

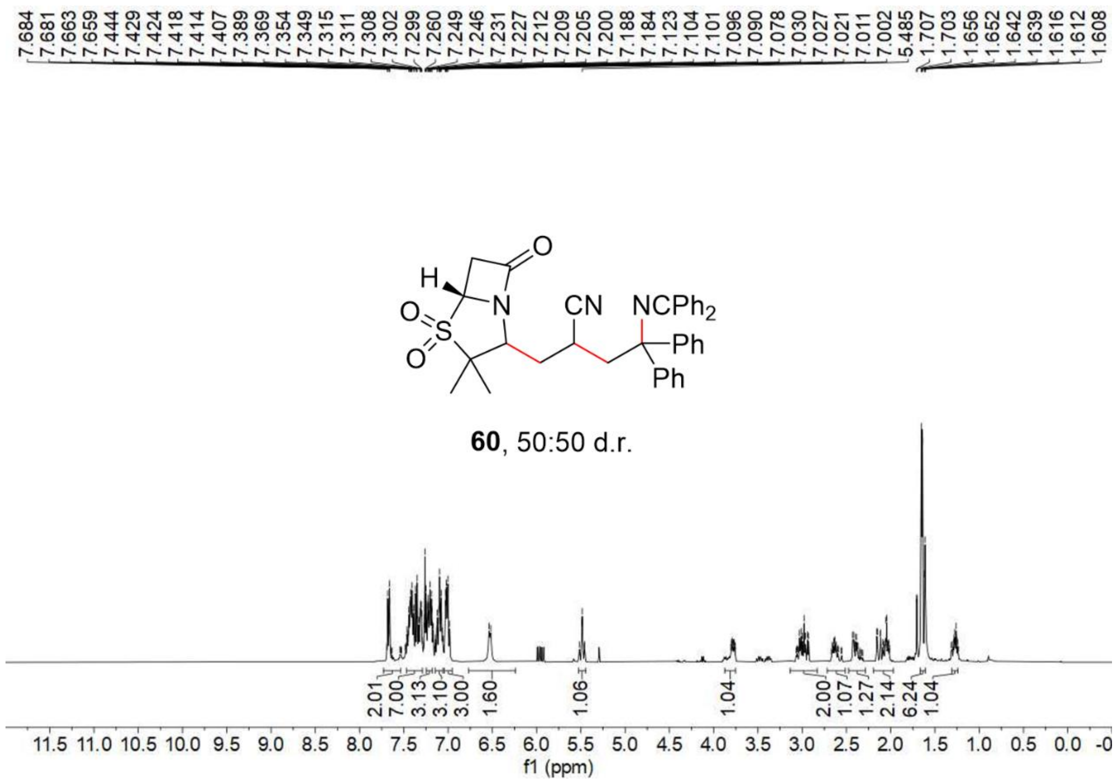
7.684 7.681 7.663 7.659 7.444 7.429 7.424 7.418 7.414 7.407 7.389 7.369 7.354 7.349 7.315 7.311 7.308 7.302 7.299 7.260 7.249 7.246 7.231 7.227 7.212 7.209 7.205 7.200 7.188 7.184 7.123 7.104 7.101 7.096 7.090 7.078 7.030 7.027 7.021 7.011 7.002 5.485 1.707 1.703 1.656 1.652 1.642 1.639 1.616 1.612 1.608
O
H
N
CN
NCPh2
O
S
O
Ph
Ph
60, 50:50 d.r.
2.01 7.00 3.13 3.10 3.00 1.60 1.06 1.04 2.00 1.07 1.27 2.14 6.24 1.04
11.5 11.0 10.5 10.0 9.5 9.0 8.5 8.0 7.5 7.0 6.5 6.0 5.5 5.0 4.5 4.0 3.5 3.0 2.5 2.0 1.5 1.0 0.5 0.0 -0
f1 (ppm)

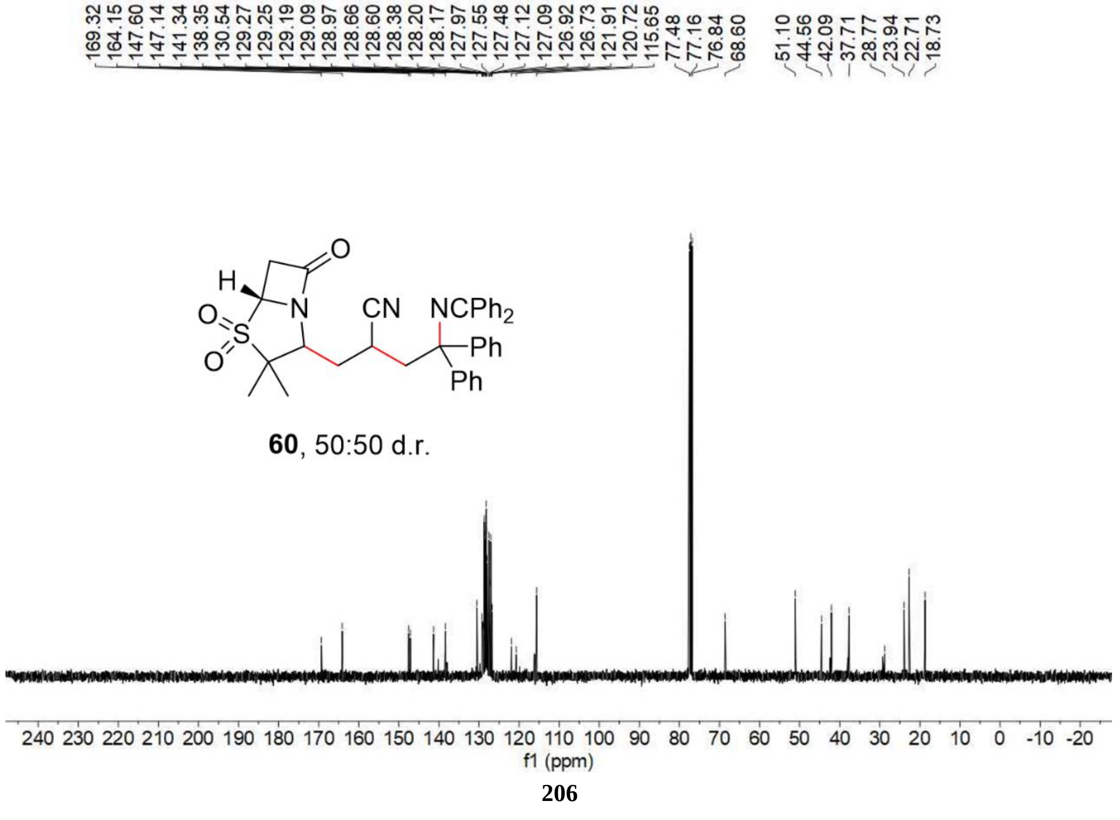
169.32 164.15 147.60 147.14 141.34 138.35 130.54 129.27 129.25 129.19 129.09 128.97 128.66 128.60 128.38 128.20 128.17 127.97 127.55 127.48 127.12 127.09 126.92 126.73 121.91 120.72 115.65 77.48 77.16 76.84 68.60 51.10 44.56 42.09 37.71 28.77 23.94 22.71 18.73
O
H
N
CN
NCPh2
O
S
O
Ph
Ph
60, 50:50 d.r.
240 230 220 210 200 190 180 170 160 150 140 130 120 110 100 90 80 70 60 50 40 30 20 10 0 -10 -20
f1 (ppm)

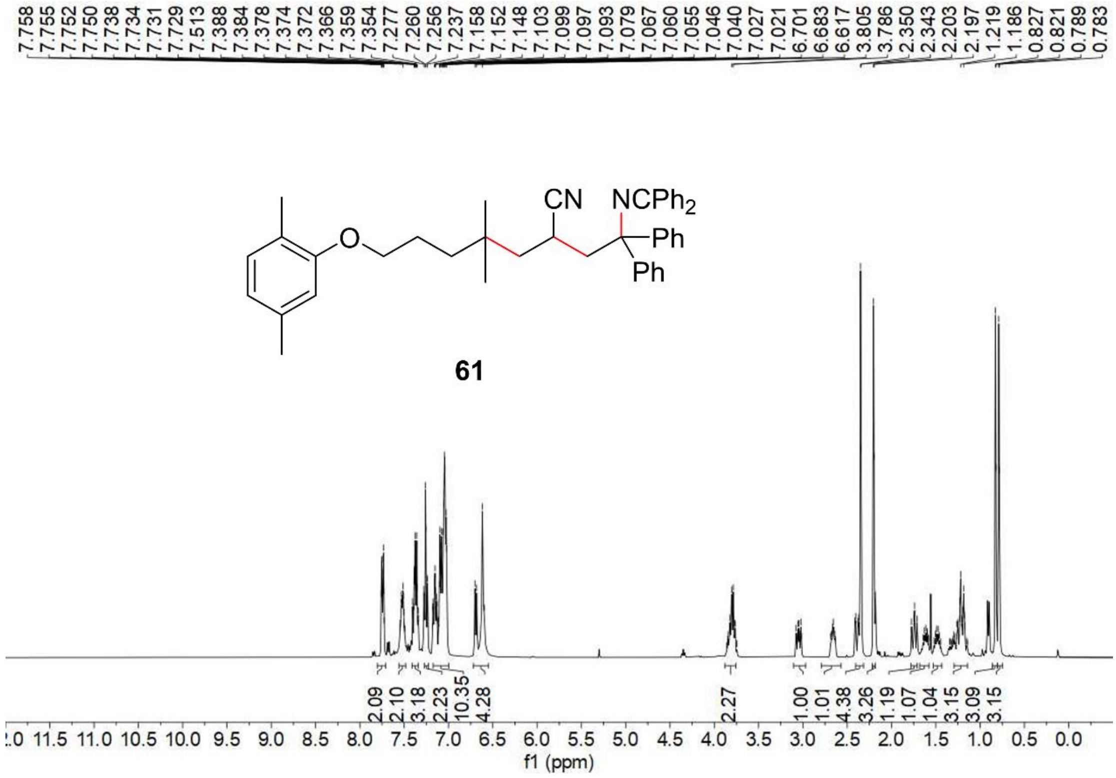
CN NCPh2
O
Ph
Ph
61
f1 (ppm)

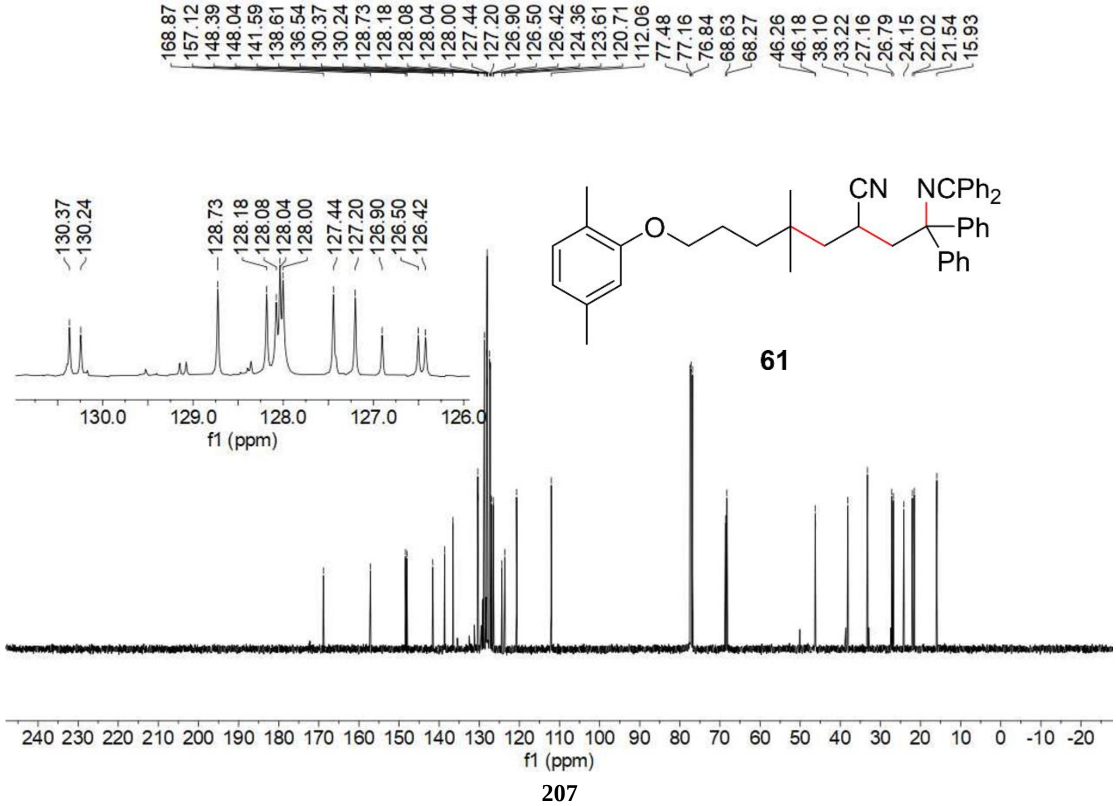
CN NCPh2
O
Ph
Ph
61
f1 (ppm)

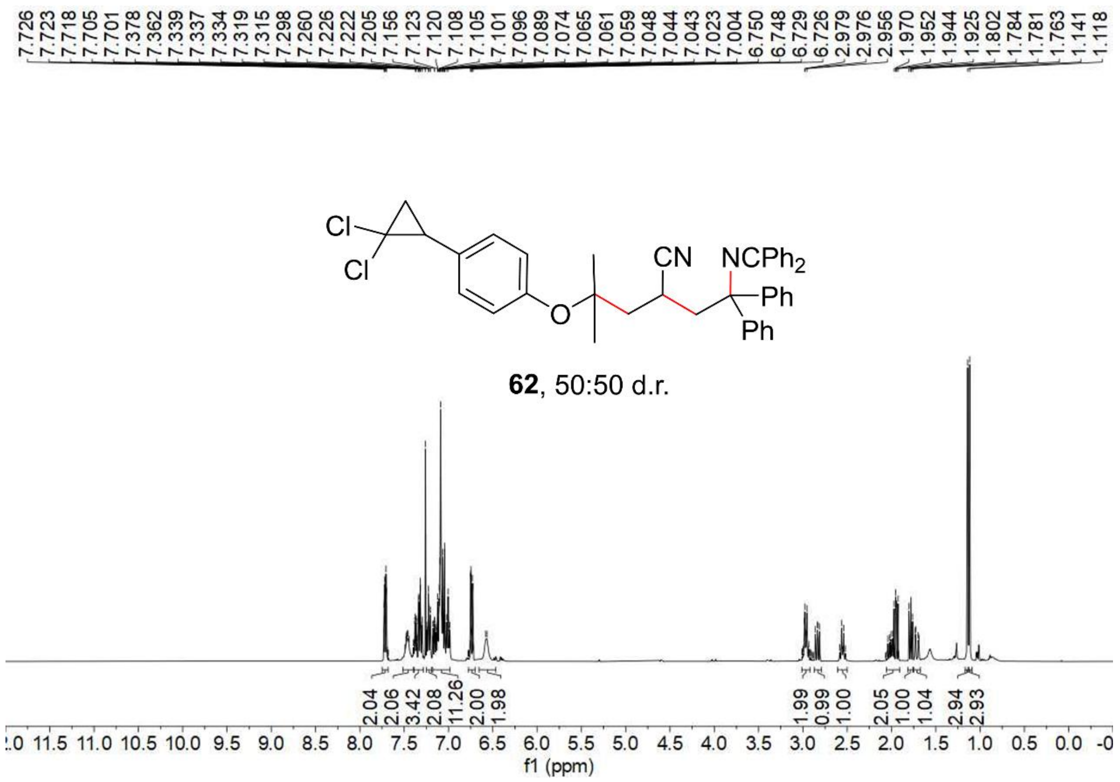
7.726 7.723 7.718 7.705 7.701 7.378 7.362 7.339 7.337 7.334 7.319 7.315 7.298 7.260 7.226 7.222 7.205 7.156 7.123 7.120 7.108 7.105 7.101 7.096 7.089 7.074 7.065 7.061 7.059 7.048 7.044 7.043 7.023 7.004 6.750 6.748 6.729 6.726 2.979 2.976 2.956 1.970 1.952 1.944 1.925 1.802 1.784 1.781 1.763 1.141 1.118
Cl
Cl
O
CN
NCPh$_2$
Ph
Ph
62, 50:50 d.r.
2.04 2.06 3.42 2.08 11.26 2.00 1.98
1.99 0.99 1.00 2.05 1.00 1.04 2.94 2.93
f1 (ppm)

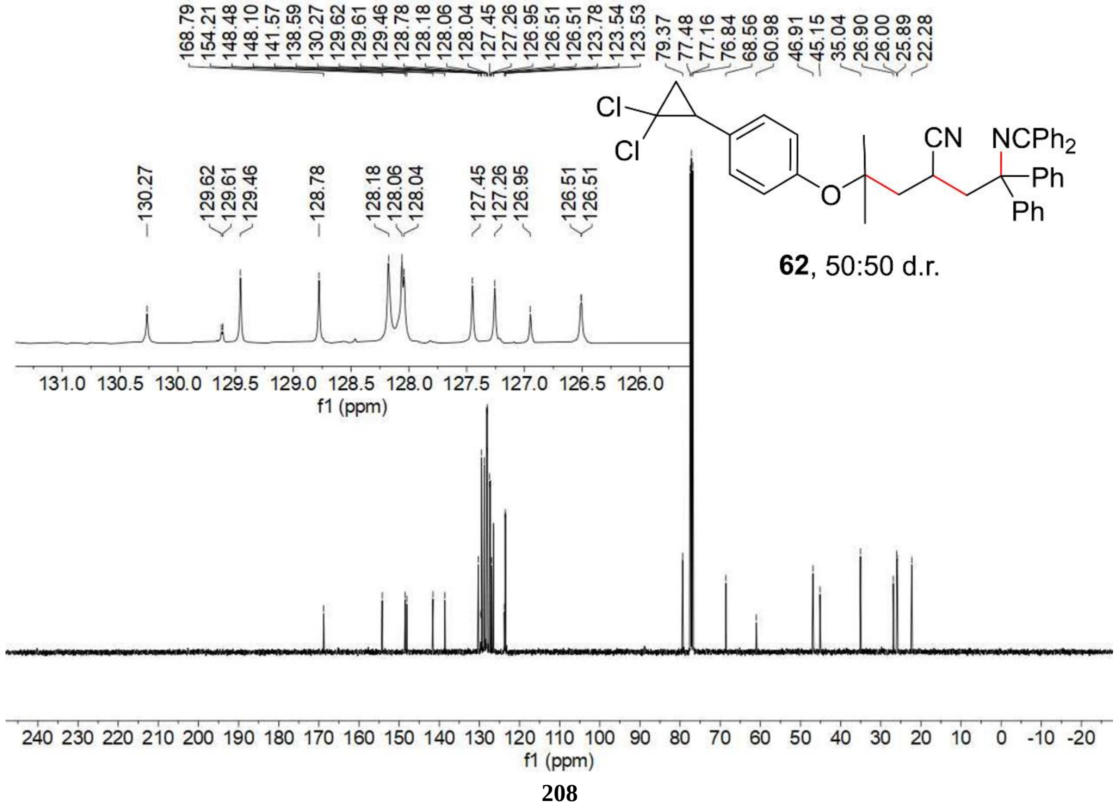
168.79 154.21 148.48 148.10 141.57 138.59 130.27 129.62 129.61 129.46 128.78 128.18 128.06 128.04 127.45 127.26 126.95 126.51 126.51 123.78 123.54 123.53 79.37 77.48 77.16 76.84 68.56 60.98 46.91 45.15 35.04 26.90 26.00 25.89 22.28
130.27 129.62 129.61 129.46 128.78 128.18 128.06 128.04 127.45 127.26 126.95 126.51 126.51
f1 (ppm)
Cl
Cl
O
CN
NCPh$_2$
Ph
Ph
62, 50:50 d.r.
f1 (ppm)

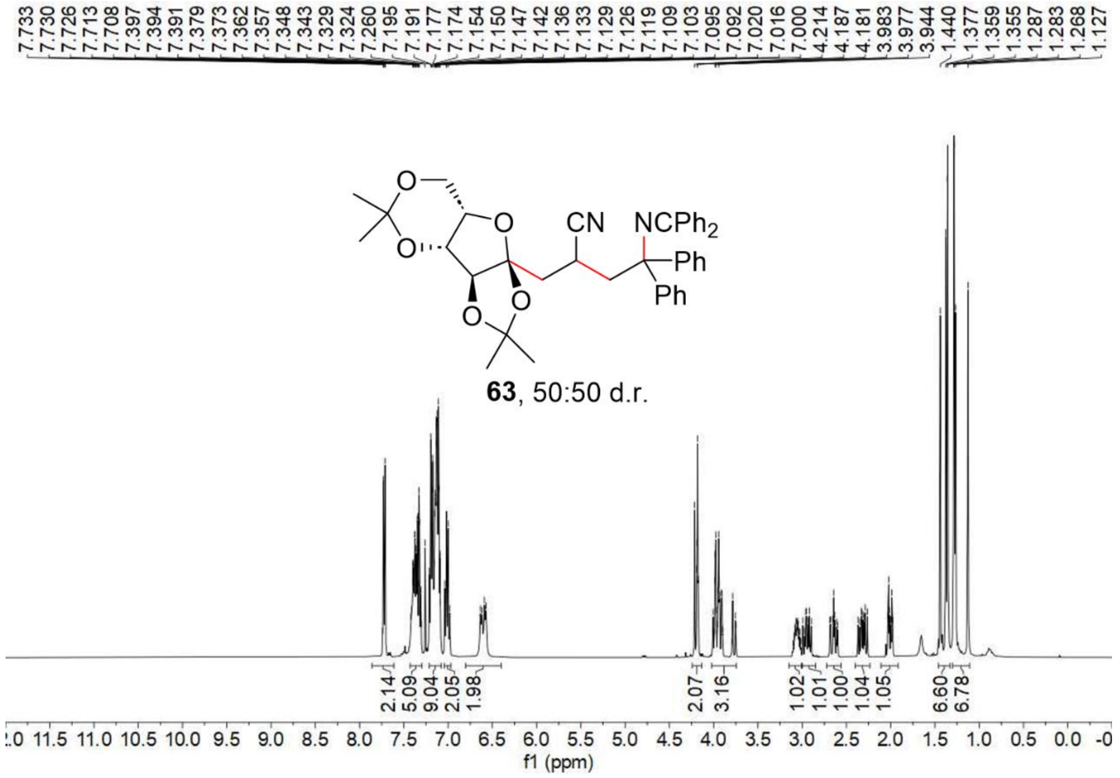

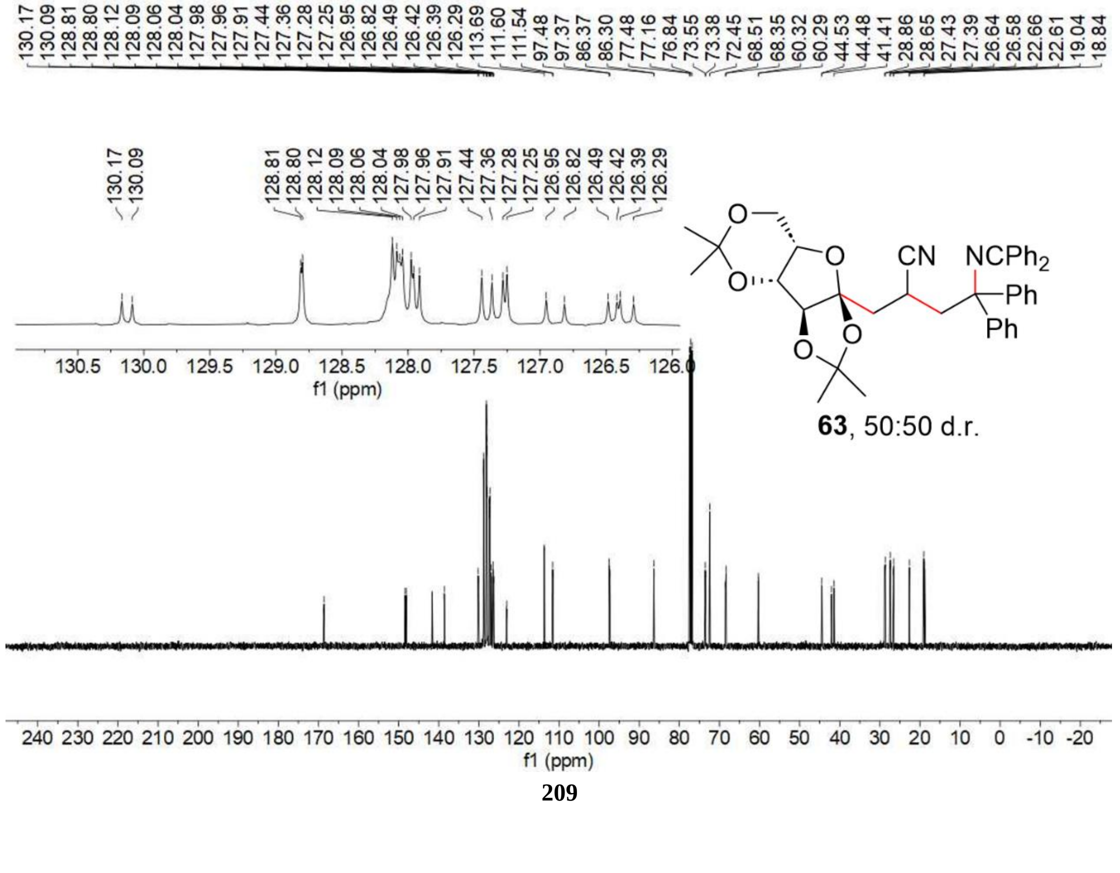

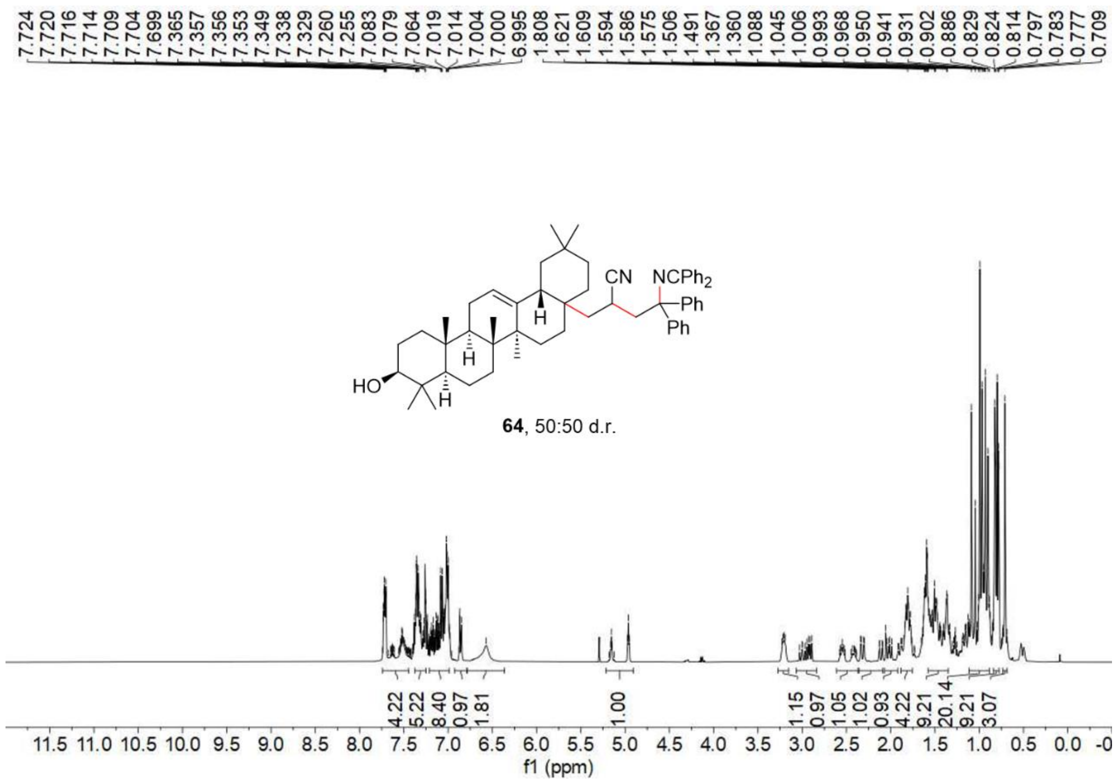
7.724 7.720 7.716 7.714 7.709 7.704 7.699 7.365 7.357 7.356 7.353 7.349 7.338 7.329 7.260 7.255 7.083 7.079 7.064 7.019 7.014 7.004 7.000 6.995 1.808 1.621 1.609 1.594 1.586 1.575 1.506 1.491 1.367 1.360 1.088 1.045 1.006 0.993 0.968 0.950 0.941 0.931 0.902 0.886 0.829 0.824 0.814 0.797 0.783 0.777 0.709
CN
NCPh2
Ph
Ph
HO
H
H
H
64, 50:50 d.r.
4.22 5.22 8.40 0.97 1.81 1.00 1.15 0.97 1.05 1.02 0.93 4.22 9.21 20.14 9.21 3.07
11.5 11.0 10.5 10.0 9.5 9.0 8.5 8.0 7.5 7.0 6.5 6.0 5.5 5.0 4.5 4.0 3.5 3.0 2.5 2.0 1.5 1.0 0.5 0.0
f1 (ppm)

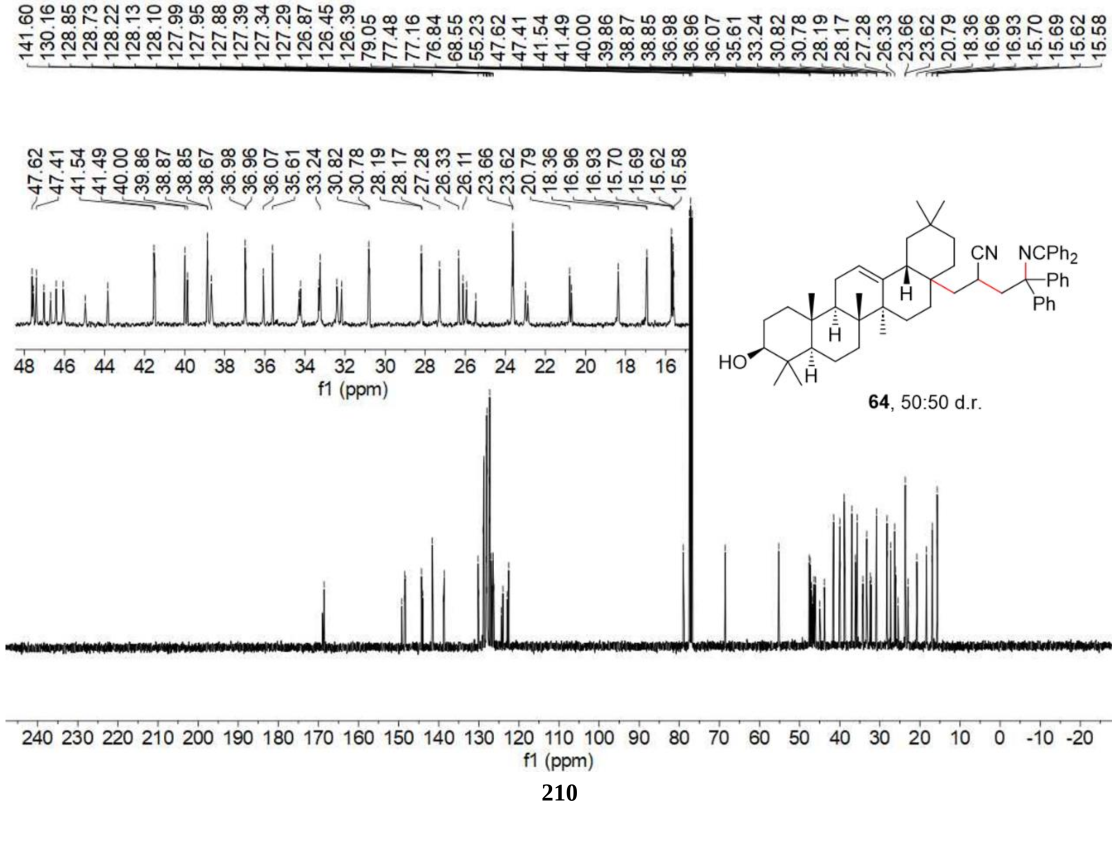
141.60 130.16 128.85 128.73 128.22 128.13 128.10 127.99 127.95 127.88 127.39 127.34 127.29 126.87 126.45 126.39 79.05 77.48 77.16 76.84 68.55 55.23 47.62 47.41 41.54 41.49 40.00 39.86 38.87 38.85 36.98 36.96 36.07 35.61 33.24 30.82 30.78 28.19 28.17 27.28 26.33 23.66 23.62 20.79 18.36 16.96 16.93 15.70 15.69 15.62 15.58
47.62 47.41 41.54 41.49 40.00 39.86 38.87 38.85 38.67 36.98 36.96 36.07 35.61 33.24 30.82 30.78 28.19 28.17 27.28 26.33 26.11 23.66 23.62 20.79 18.36 16.96 16.93 15.70 15.69 15.62 15.58
48 46 44 42 40 38 36 34 32 30 28 26 24 22 20 18 16
f1 (ppm)
CN
NCPh2
Ph
Ph
HO
H
H
H
64, 50:50 d.r.
240 230 220 210 200 190 180 170 160 150 140 130 120 110 100 90 80 70 60 50 40 30 20 10 0 -10 -20
f1 (ppm)

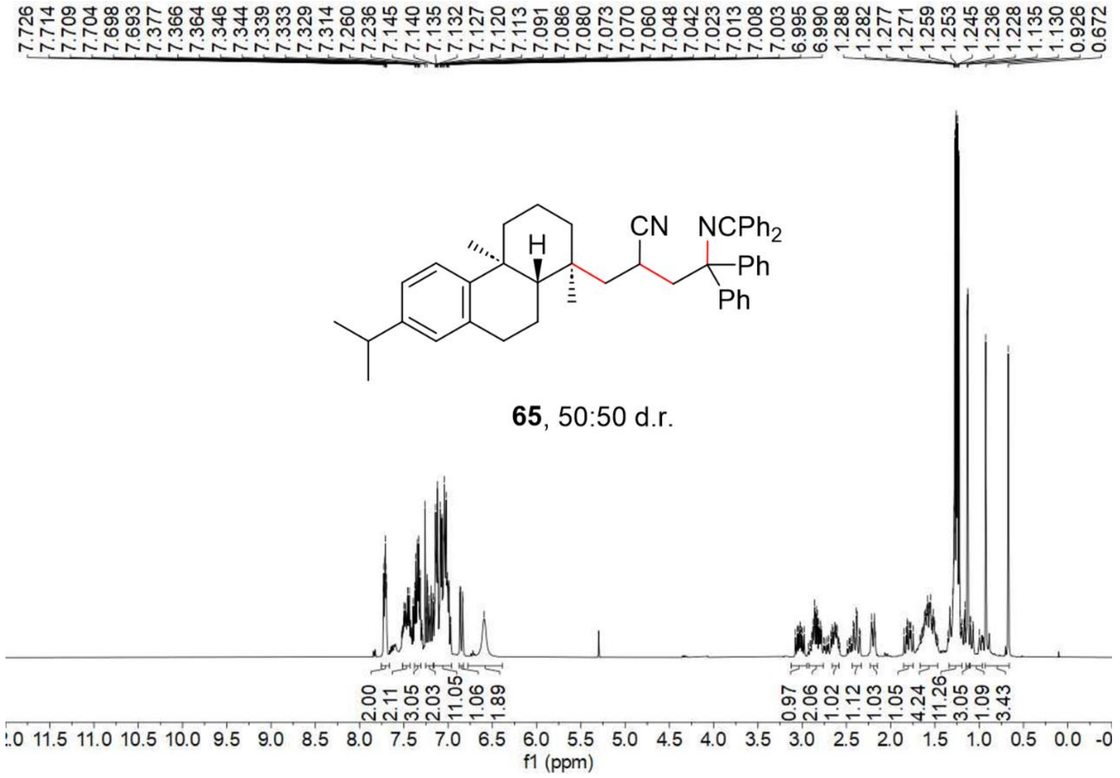
CN
NCPh$_2$
Ph
Ph
H
65, 50:50 d.r.
f1 (ppm)

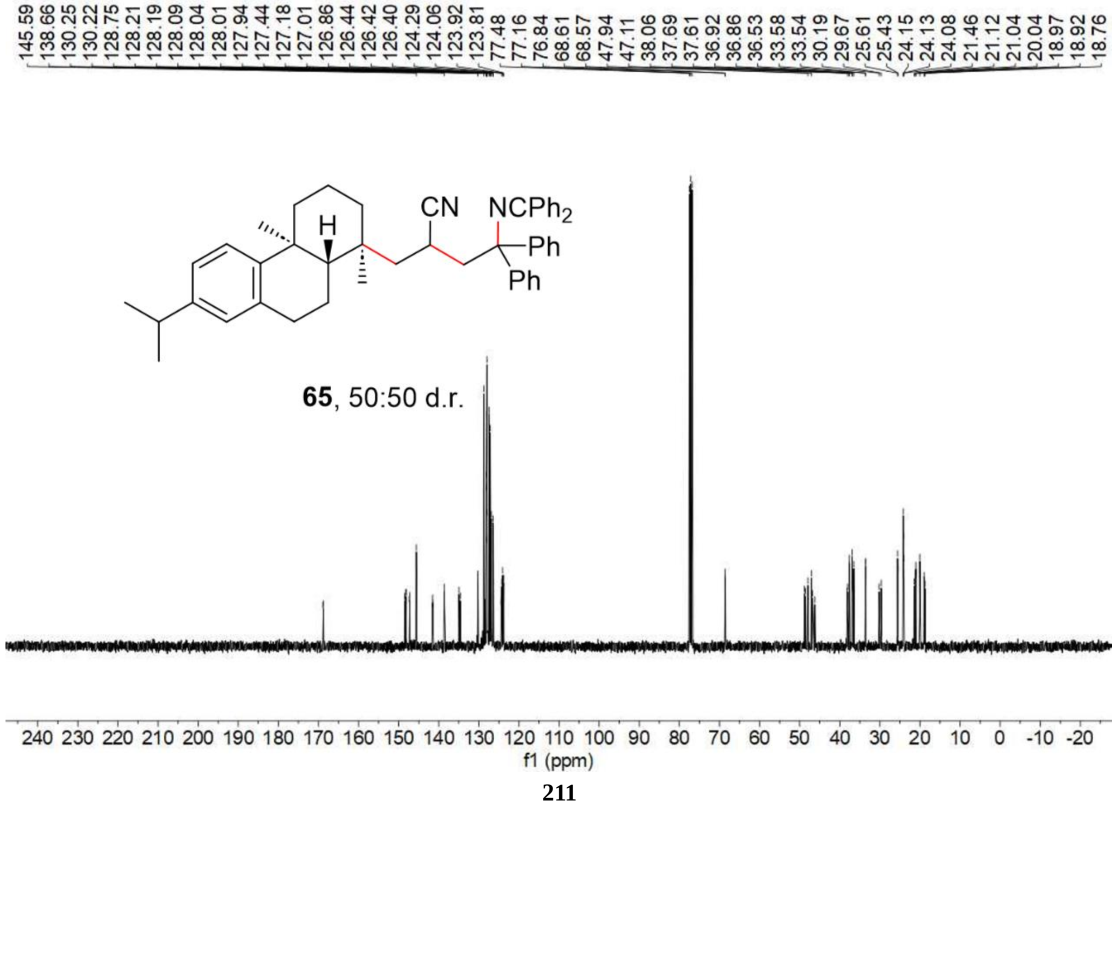
CN
NCPh$_2$
Ph
Ph
H
65, 50:50 d.r.
f1 (ppm)

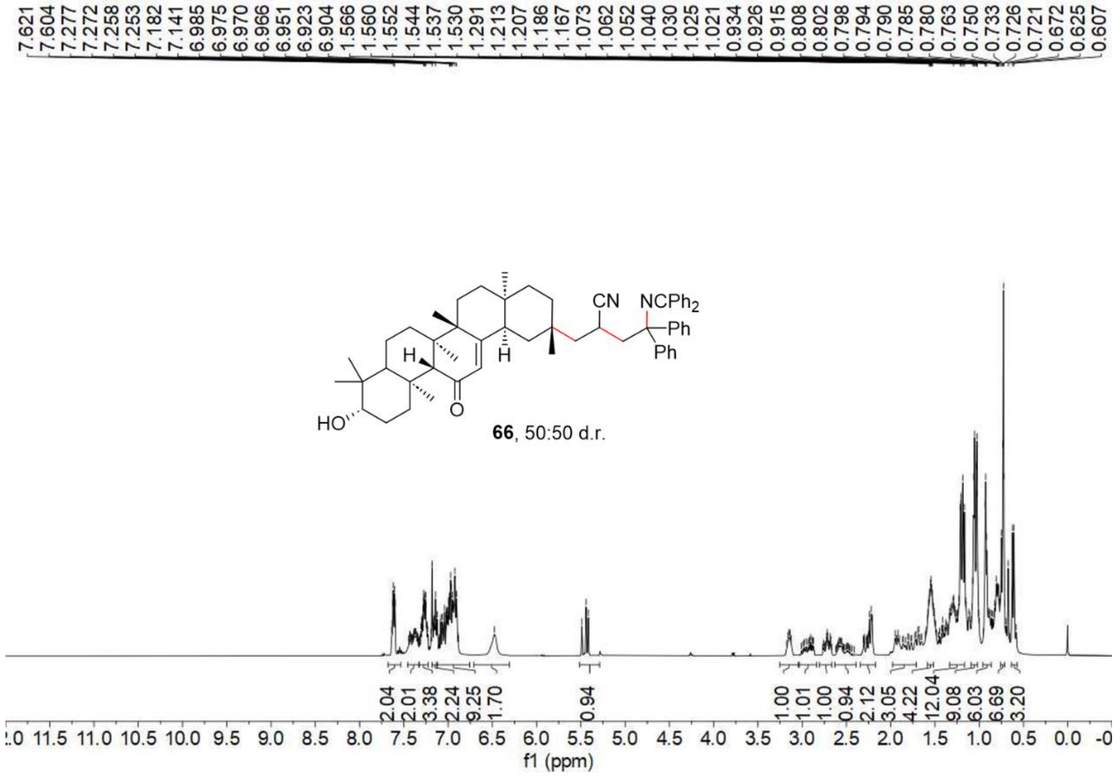
7.621
7.604
7.277
7.272
7.258
7.253
7.182
7.141
6.985
6.975
6.970
6.966
6.951
6.923
6.904
1.566
1.560
1.552
1.544
1.537
1.530
1.291
1.213
1.207
1.186
1.167
1.073
1.062
1.052
1.040
1.030
1.025
1.021
0.934
0.926
0.915
0.808
0.802
0.798
0.794
0.790
0.785
0.780
0.763
0.750
0.733
0.726
0.721
0.672
0.625
0.607
CN
NCPh2
Ph
Ph
H
H
O
HO
66, 50:50 d.r.
2.04
2.01
3.38
2.24
9.25
1.70
0.94
1.00
1.01
1.00
0.94
2.12
3.05
4.22
12.04
9.08
6.03
6.69
3.20
f1 (ppm)

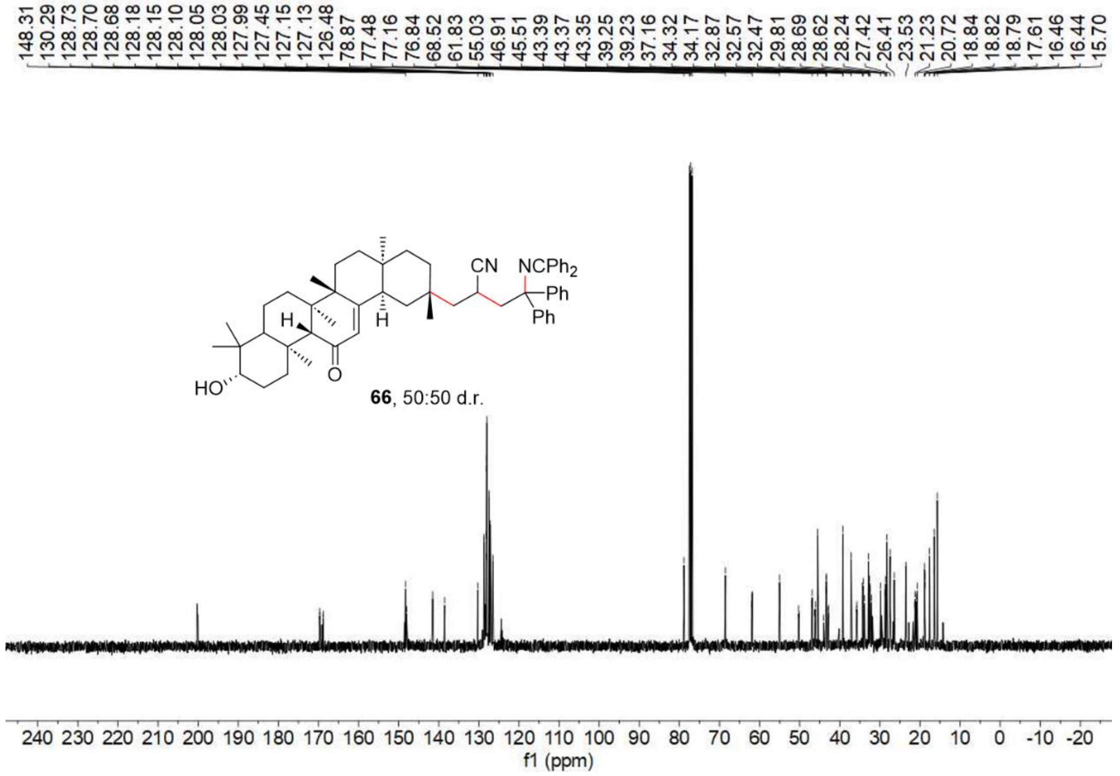
148.31
130.29
128.73
128.70
128.68
128.18
128.15
128.10
128.05
128.03
127.99
127.45
127.15
127.13
126.48
78.87
77.48
77.16
76.84
68.52
61.83
55.03
46.91
45.51
43.39
43.37
43.35
39.25
39.23
37.16
34.32
34.17
32.87
32.57
32.47
29.81
28.69
28.62
28.24
27.42
26.41
23.53
21.23
20.72
18.84
18.82
18.79
17.61
16.46
16.44
15.70
CN
NCPh2
Ph
Ph
H
H
O
HO
66, 50:50 d.r.
240 230 220 210 200 190 180 170 160 150 140 130 120 110 100 90 80 70 60 50 40 30 20 10 0 -10 -20
f1 (ppm)

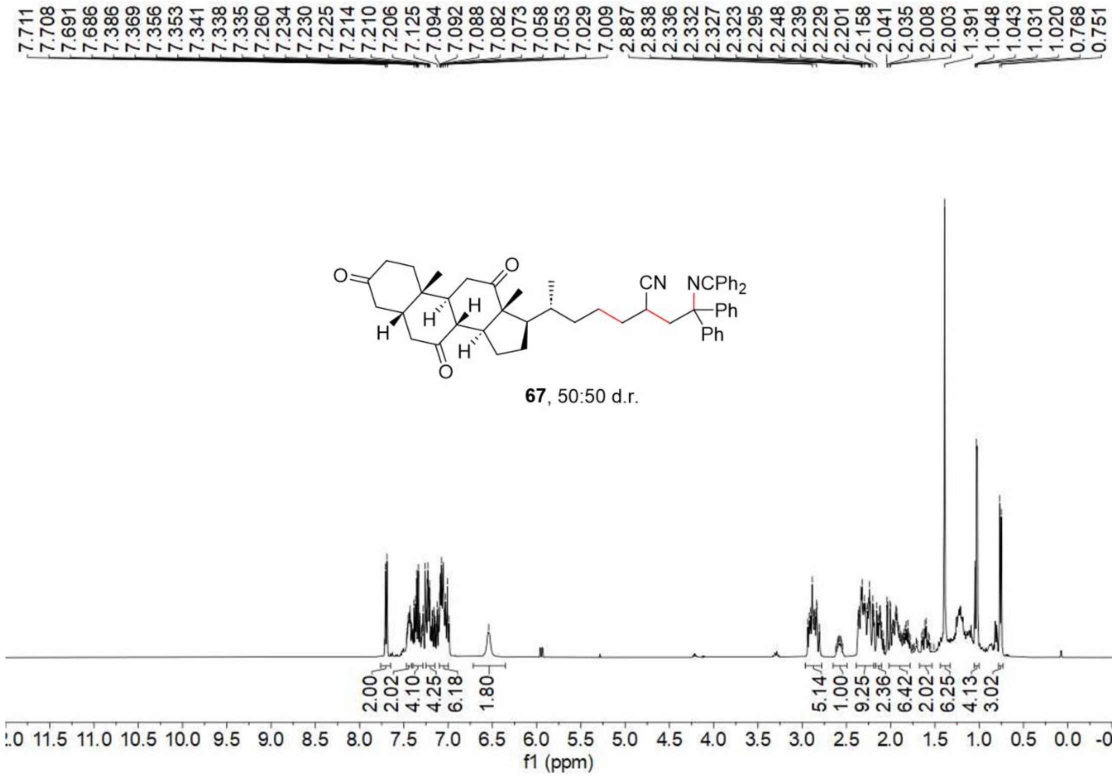
7.711 7.708 7.691 7.686 7.386 7.369 7.356 7.353 7.341 7.338 7.335 7.260 7.234 7.230 7.225 7.214 7.210 7.206 7.125 7.094 7.092 7.088 7.082 7.073 7.058 7.053 7.029 7.009 2.887 2.838 2.336 2.332 2.327 2.323 2.295 2.248 2.239 2.229 2.201 2.158 2.041 2.035 2.008 2.003 1.391 1.048 1.043 1.031 1.020 0.768 0.751
CN
NCPh2
Ph
Ph
67, 50:50 d.r.
2.00 2.02 4.10 4.25 6.18 1.80
5.14 1.00 9.25 2.36 6.42 2.02 6.25 4.13 3.02
f1 (ppm)

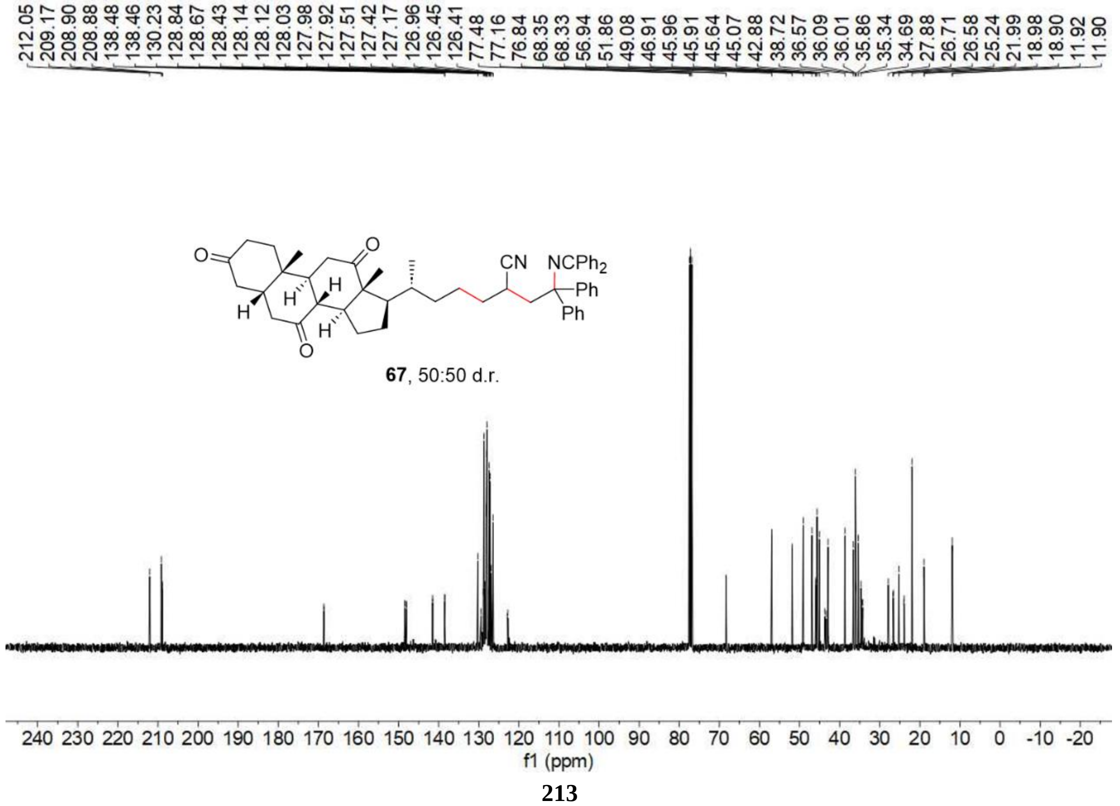
212.05 209.17 208.90 208.88 138.48 138.46 130.23 128.84 128.67 128.43 128.14 128.12 128.03 127.98 127.92 127.51 127.42 127.17 126.96 126.45 126.41 77.48 77.16 76.84 68.35 68.33 56.94 51.86 49.08 46.91 45.96 45.91 45.64 45.07 42.88 38.72 36.57 36.09 36.01 35.86 35.34 34.69 27.88 26.71 26.58 25.24 21.99 18.98 18.90 11.92 11.90
CN
NCPh2
Ph
Ph
67, 50:50 d.r.
f1 (ppm)

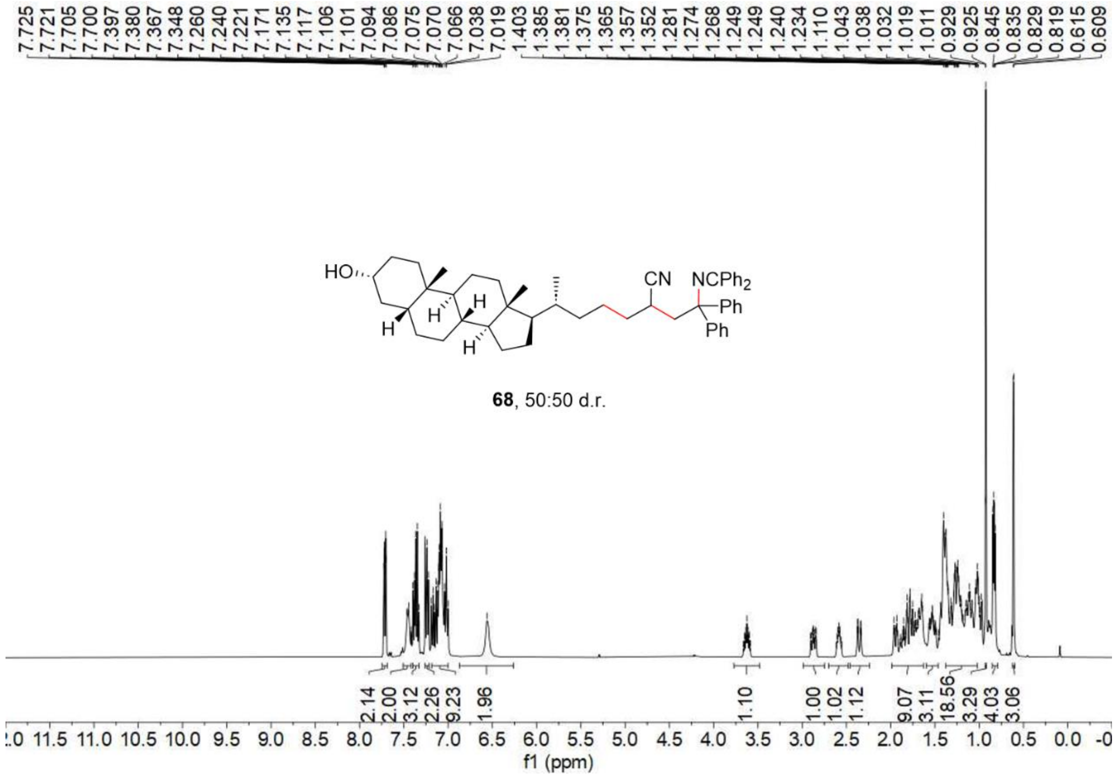
7.725 7.721 7.705 7.700 7.397 7.380 7.367 7.348 7.260 7.240 7.221 7.171 7.135 7.117 7.106 7.101 7.094 7.086 7.075 7.070 7.066 7.038 7.019 1.403 1.385 1.381 1.375 1.365 1.357 1.352 1.281 1.274 1.268 1.249 1.249 1.240 1.234 1.110 1.043 1.038 1.032 1.019 1.011 0.929 0.925 0.845 0.835 0.829 0.819 0.615 0.609
HO
H
H
H
H
CN
NCPh2
Ph
Ph
68, 50:50 d.r.
2.14 2.00 3.12 2.26 9.23 1.96 1.10 1.00 1.02 1.12 9.07 3.11 18.56 3.29 4.03 3.06
f1 (ppm)

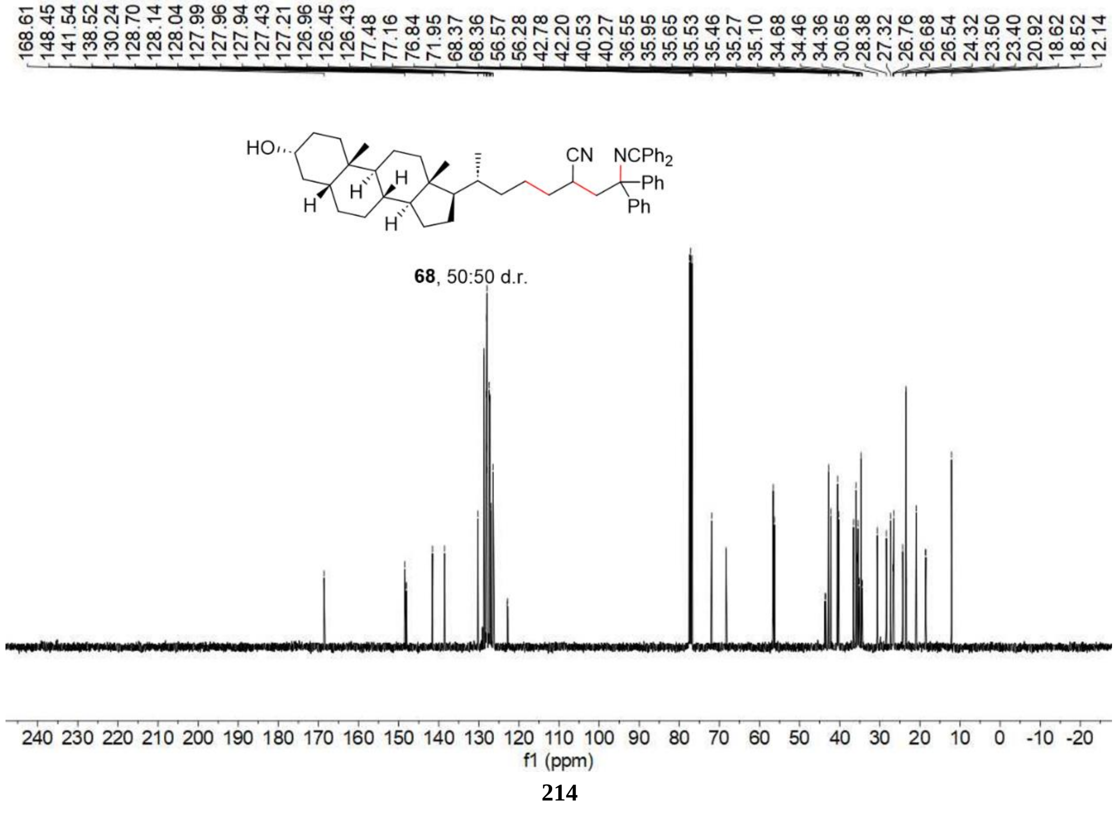
168.61 148.45 141.54 138.52 130.24 128.70 128.14 128.04 127.99 127.96 127.94 127.43 127.21 126.96 126.45 126.43 77.48 77.16 76.84 71.95 68.37 68.36 56.57 56.28 42.78 42.20 40.53 40.27 36.55 35.95 35.65 35.53 35.46 35.27 35.10 34.68 34.46 34.36 30.65 28.38 27.32 26.76 26.68 26.54 24.32 23.50 23.40 20.92 18.62 18.52 12.14
HO
H
H
H
H
CN
NCPh2
Ph
Ph
68, 50:50 d.r.
f1 (ppm)

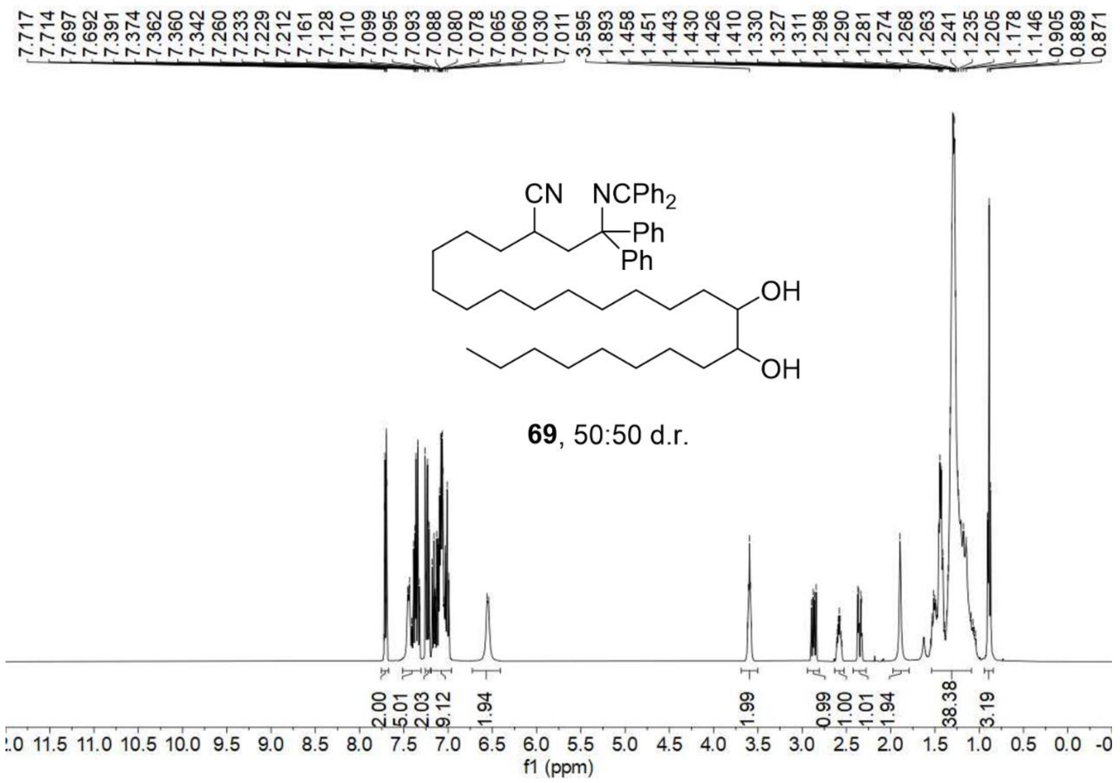
CN
NCPh2
Ph
Ph
OH
OH
69, 50:50 d.r.
f1 (ppm)

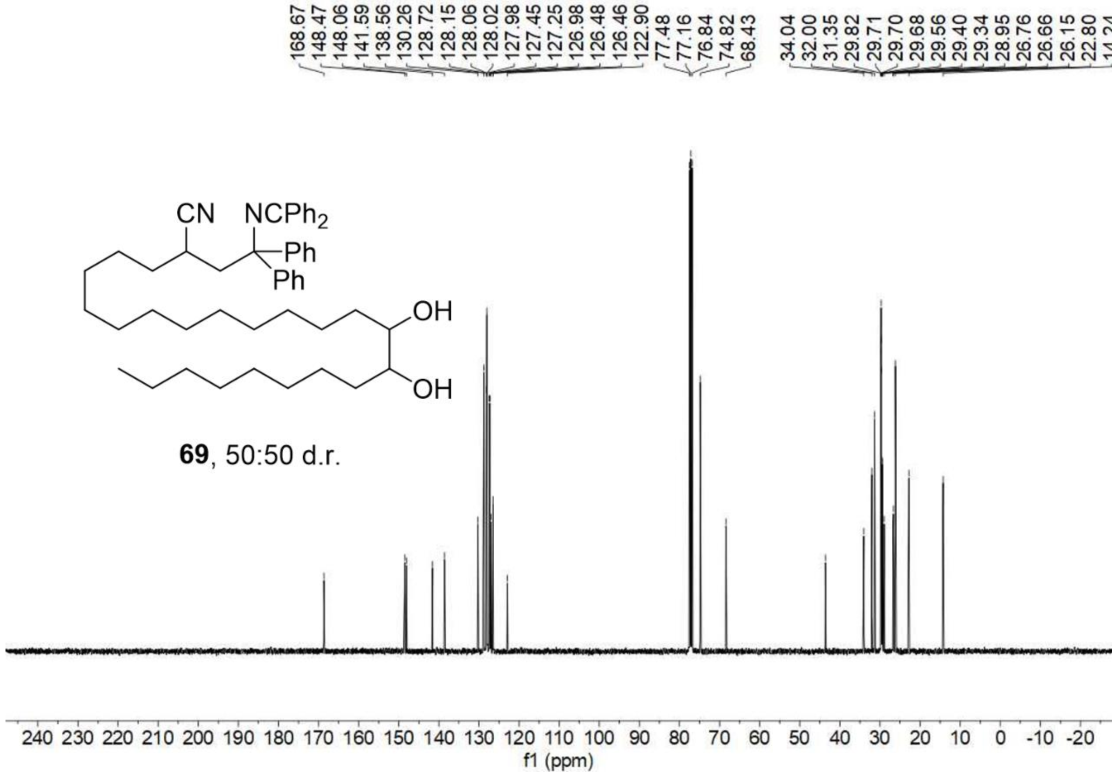
CN
NCPh2
Ph
Ph
OH
OH
69, 50:50 d.r.
f1 (ppm)

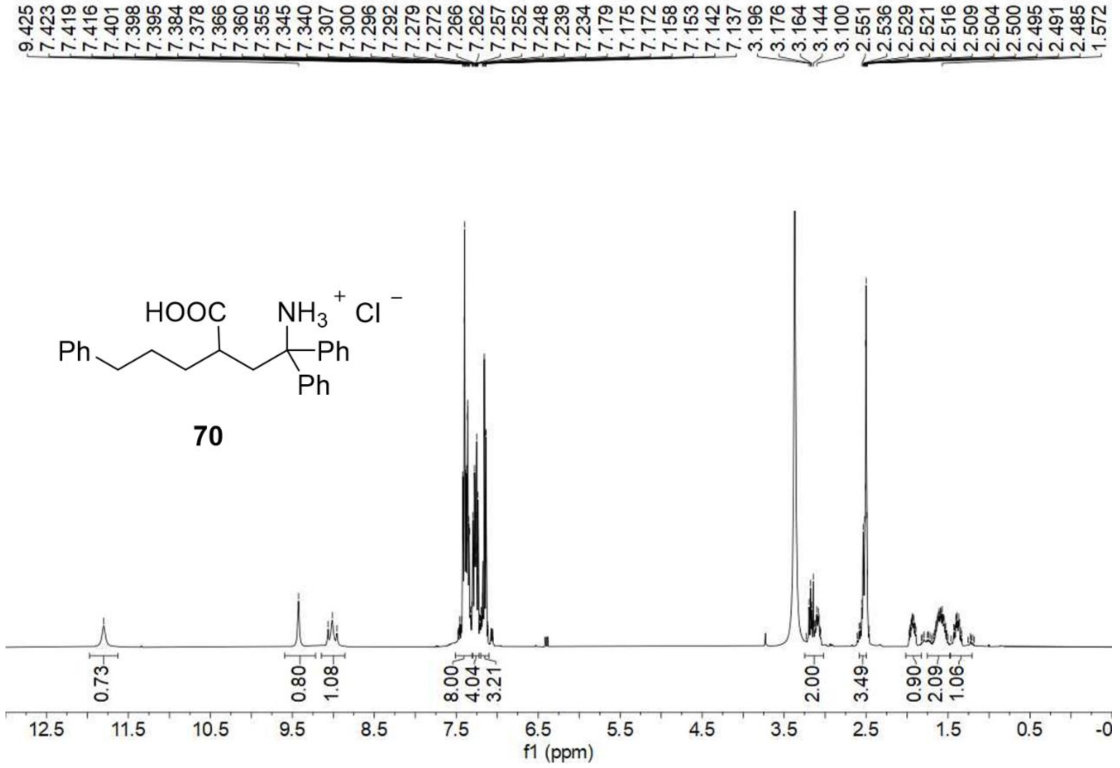
9.425
7.423
7.419
7.416
7.401
7.398
7.395
7.384
7.378
7.366
7.360
7.355
7.345
7.340
7.307
7.300
7.296
7.292
7.279
7.272
7.266
7.262
7.257
7.252
7.248
7.239
7.234
7.179
7.175
7.172
7.158
7.153
7.142
7.137
3.196
3.176
3.164
3.144
3.100
2.551
2.536
2.529
2.521
2.516
2.509
2.504
2.500
2.495
2.491
2.485
1.572
HOOC
NH3 + Cl −
Ph
Ph
Ph
70
0.73
0.80
1.08
8.00
4.04
3.21
2.00
3.49
0.90
2.09
1.06
12.5 11.5 10.5 9.5 8.5 7.5 6.5 5.5 4.5 3.5 2.5 1.5 0.5 -0
f1 (ppm)

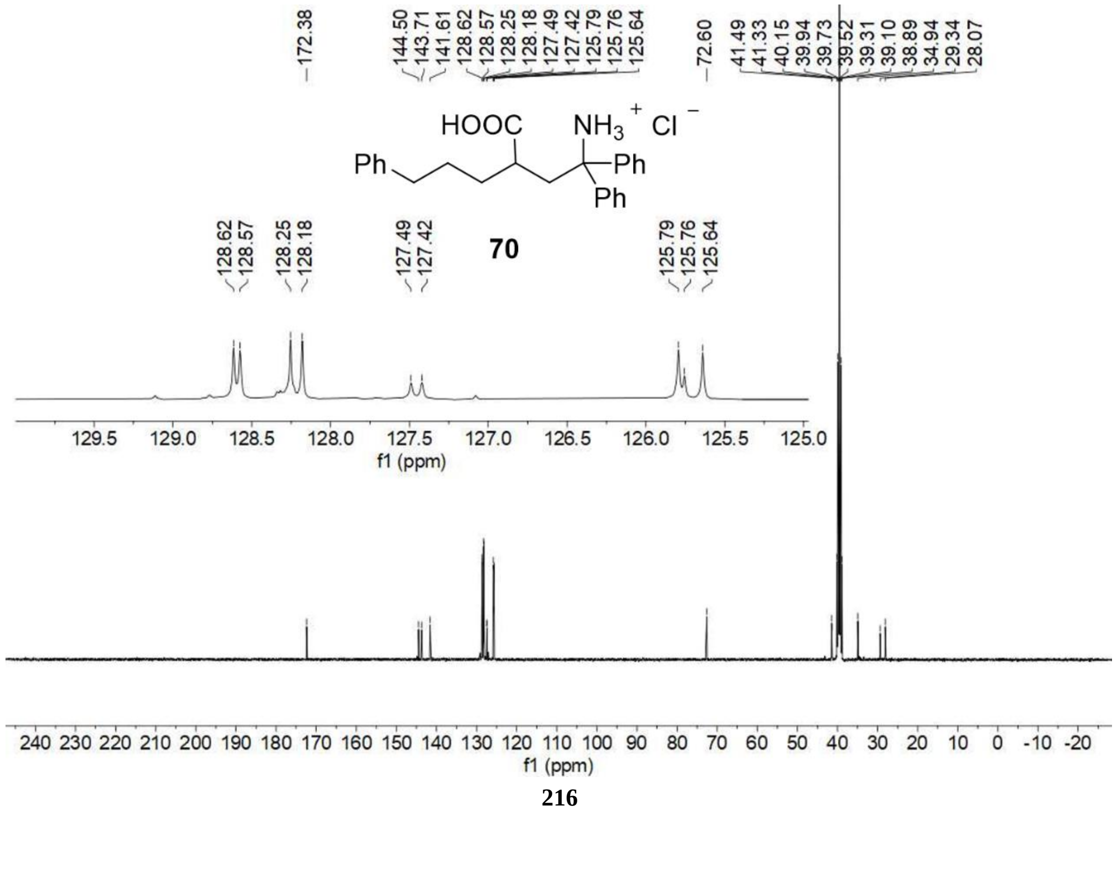
172.38
144.50
143.71
141.61
128.62
128.57
128.25
128.18
127.49
127.42
125.79
125.76
125.64
72.60
41.49
41.33
40.15
39.94
39.73
39.52
39.31
39.10
38.89
34.94
29.34
28.07
HOOC
NH3 + Cl −
Ph
Ph
Ph
70
128.62
128.57
128.25
128.18
127.49
127.42
125.79
125.76
125.64
129.5 129.0 128.5 128.0 127.5 127.0 126.5 126.0 125.5 125.0
f1 (ppm)
240 230 220 210 200 190 180 170 160 150 140 130 120 110 100 90 80 70 60 50 40 30 20 10 0 -10 -20
f1 (ppm)

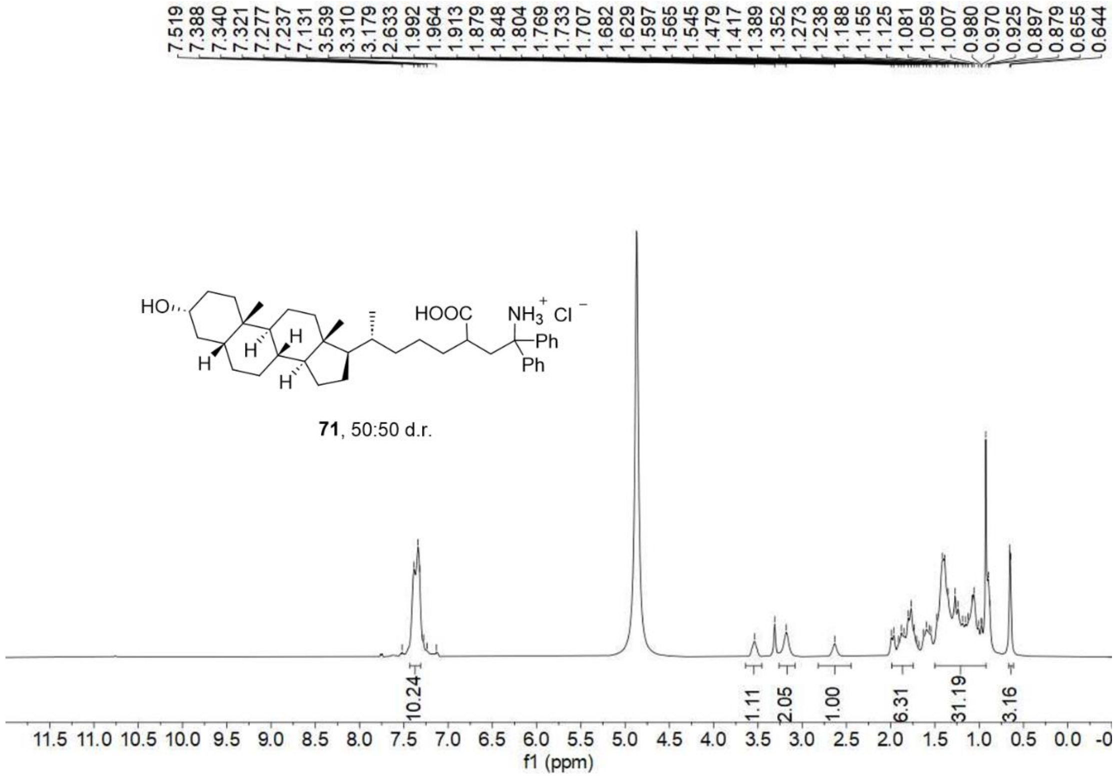
7.519 7.388 7.340 7.321 7.277 7.237 7.131 3.539 3.310 3.179 2.633 1.992 1.964 1.913 1.879 1.848 1.804 1.769 1.733 1.707 1.682 1.629 1.597 1.565 1.545 1.479 1.417 1.389 1.352 1.273 1.238 1.188 1.155 1.125 1.081 1.059 1.007 0.980 0.970 0.925 0.897 0.879 0.655 0.644
HO
HOOC
NH_3^+ Cl$^-$
Ph
Ph
H
H
H
H
71, 50:50 d.r.
10.24
1.11
2.05
1.00
6.31
31.19
3.16
11.5 11.0 10.5 10.0 9.5 9.0 8.5 8.0 7.5 7.0 6.5 6.0 5.5 5.0 4.5 4.0 3.5 3.0 2.5 2.0 1.5 1.0 0.5 0.0 -0
f1 (ppm)

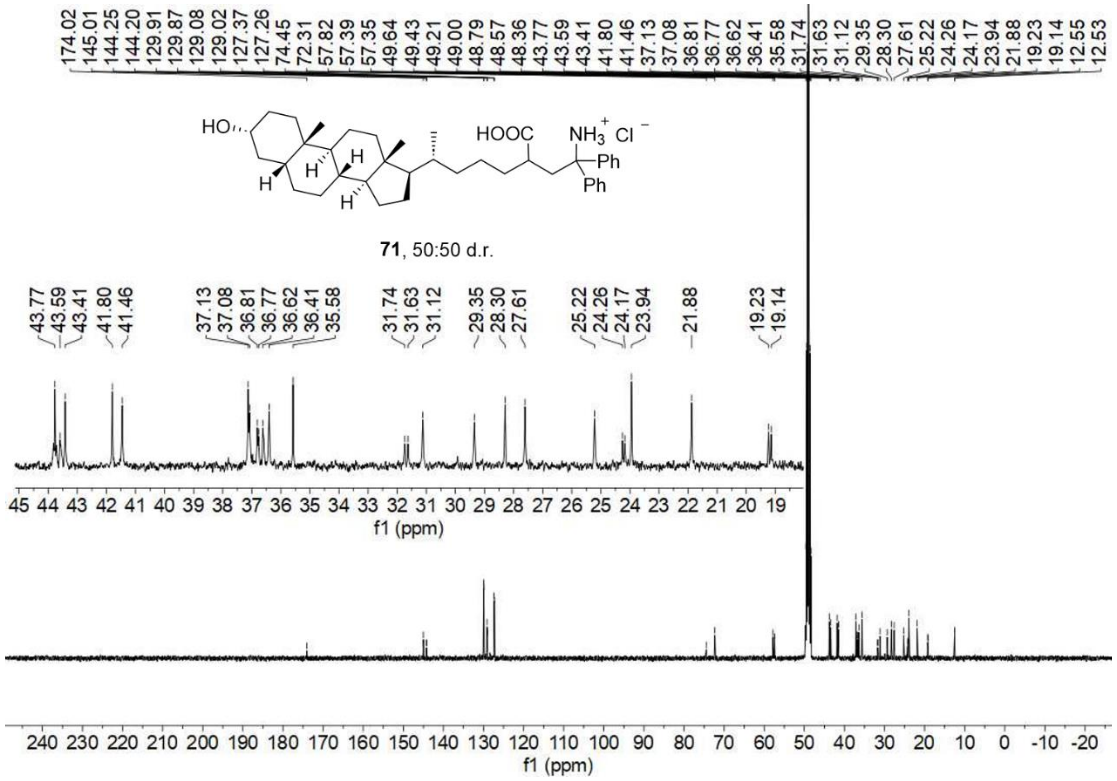
174.02 145.01 144.25 144.20 129.91 129.87 129.08 129.02 127.37 127.26 74.45 72.31 57.82 57.39 57.35 49.64 49.43 49.21 49.00 48.79 48.57 48.36 43.77 43.59 43.41 41.80 41.46 37.13 37.08 36.81 36.77 36.62 36.41 35.58 31.74 31.63 31.12 29.35 28.30 27.61 25.22 24.26 24.17 23.94 21.88 19.23 19.14 12.55 12.53
HO
HOOC
NH_3^+ Cl$^-$
Ph
Ph
H
H
H
H
71, 50:50 d.r.
43.77 43.59 43.41 41.80 41.46 37.13 37.08 36.81 36.77 36.62 36.41 35.58 31.74 31.63 31.12 29.35 28.30 27.61 25.22 24.26 24.17 23.94 21.88 19.23 19.14
45 44 43 42 41 40 39 38 37 36 35 34 33 32 31 30 29 28 27 26 25 24 23 22 21 20 19
f1 (ppm)
240 230 220 210 200 190 180 170 160 150 140 130 120 110 100 90 80 70 60 50 40 30 20 10 0 -10 -20
f1 (ppm)
